# Enantioselective Cu-catalyzed double hydroboration of alkynes to access chiral gem-diborylalkanes

**DOI:** 10.1038/s41467-022-31234-2

**Published:** 2022-06-20

**Authors:** Shengnan Jin, Jinxia Li, Kang Liu, Wei-Yi Ding, Shuai Wang, Xiujuan Huang, Xue Li, Peiyuan Yu, Qiuling Song

**Affiliations:** 1 Institute of Next Generation Matter Transformation, College of Material Sciences Engineering, Huaqiao University, Xiamen, Fujian 361021 China; 2 Department of Chemistry and Shenzhen Grubbs Institute, Southern University of Science and Technology, Shenzhen, 518055 China; 3 School of Chemistry and Chemical Engineering, Henan Normal University, Xinxiang, Henan 453007 China; 4 State Key Laboratory of Elemento-Organic Chemistry, Nankai University, Tianjin, China

**Keywords:** Synthetic chemistry methodology, Chemical synthesis

## Abstract

Chiral organoborons are of great value in asymmetric synthesis, functional materials, and medicinal chemistry. The development of chiral bis(boryl) alkanes, especially optically enriched 1,1-diboron compounds, has been greatly inhibited by the lack of direct synthetic protocols. Therefore, it is very challenging to develop a simple and effective strategy to obtain chiral 1,1-diborylalkanes. Herein, we develop an enantioselective copper-catalyzed cascade double hydroboration of terminal alkynes and highly enantioenriched *gem*-diborylalkanes were readily obtained. Our strategy uses simple terminal alkynes and two different boranes to construct valuable chiral *gem*-bis(boryl) alkanes with one catalytic and one ligand pattern, which represents the simplest and most straightforward strategy for constructing such chiral *gem*-diborons.

## Abstract (web summary)

Optically enriched 1,1-diboron compounds are significantly underdeveloped because of the paucity of the straightforward synthetic protocols. Herein, the authors report an enantioselective copper-catalyzed cascade double hydroboration of terminal alkynes to access highly enantioenriched gem-diborylalkanes.

## Introduction

Chiral organoborons are of great value in asymmetric synthesis and functional materials as well as bioactive molecules^1–8^. Myriads of protocols have been developed to construct chiral mono-organoborons with carbon-stereogenic centers^9–13^, which have been proven to be very valuable chiral building blocks in organic synthesis^14,15^. Compared with chiral mono-organoboron compounds, enantioenriched bis(boryl) ones might provide the feasibility for selective and multiple C-C or C-heteroatom bonds constructions to generate new valuable chiral compounds. However, chiral bis(boryl) compounds are underdeveloped due to the paucity of straightforward synthetic strategies. The development of efficient synthetic protocols to access various chiral bis(boryl)alkanes is highly desirable for investigating their fundamental properties and exploring their potential applications.

**Fig. 1:**
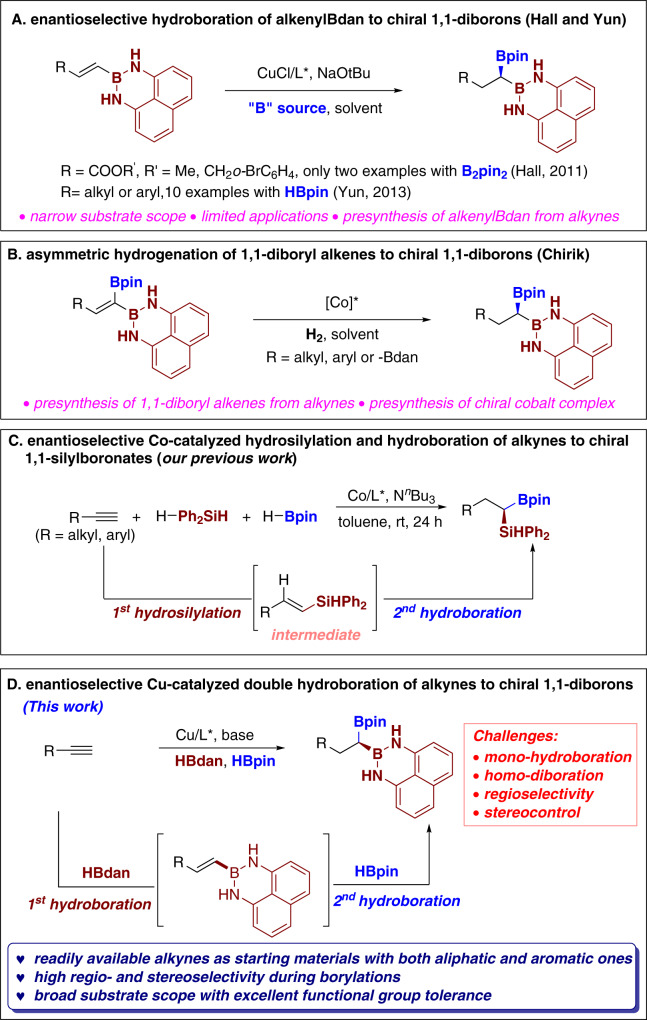
Enantioselective approaches to access chiral bis(boryl)alkanes. **A** Enantioselective hydroboration of alkenylBdan to chiral 1,1-diborons. **B** Asymmetric hydrogenation of 1,1-diboryl alkenes to chiral 1,1-diboryl alkanes. **C** Enantioselective Co-catalyzed hydrosilylation and hydroboration of alkynes to chiral 1,1-silyboronates (our previous work). **D** Our strategy for enantioselective copper-catalyzed double hydroboration of alkynes to chiral 1,1-diborons (this work).

Enantioselective hydroboration of widely existing alkenes is one of most important and efficient means to construct chiral organoboron compounds, yet which usually only leads to chiral mono-organoborons, sequential double hydroboration of readily available alkynes was considered as one of the most ideal strategies for synthesizing bis(boryl)alkanes, however, because of the inherent challenges on controlling the chemo-, regio-, and enantioselectivities on the hydrofunctionalizations of alkynes and multicomponent reactions, enantioenriched double hydroborations of alkynes have been scarcely reported^9–13^. In 2009, Hoveyda presented an enantioselective copper-catalyzed 1,2-hydroboration of aliphatic terminal alkynes for the synthesis of 1,2-bis(boryl) alkanes with B_2_pin_2_ as boron source and MeOH as hydrogen donor^16^; in 2012, Yun and coworkers disclosed a highly regio- and stereoselective Cu-catalyzed double hydroboration of silylalkynes to render *syn*-vicinal diboronates, once again with B_2_pin_2_/MeOH system^13^. Compared to enantioselective 1,2-double hydroboration of alkynes, chiral 1,1-diborylalkanes which contain two different boryl units on the same carbon atom are very rare, the seminal example came from the Hall group in 2011^17^, in which chiral 1,1-diborons were generated from enantioselective Cu-catalyzed hydroboration of alkenylBdan substrates (dan, 1,8-diaminonaphthalenyl-) with B_2_pin_2_ as boron source. Two years later, Yun and coworkers disclosed an enantioselective hydroboration of alkenylBdan via Cu-catalysis with HBpin as boron source (Fig. 1A)^2^. Despite as the sporadic seminal two methods on the preparation of chiral 1,1-diboryl alkanes, Hall’s strategy and Yun’s method have significant shortcomings, including the requirement to prepare the alkenylBdan as starting material through a one- or two-step synthesis, narrow substrate scope with very few examples, and limited synthetic applications employed for the chiral 1,1-diboryl alkanes. Recently, the Chirik group developed an asymmetric hydrogenation of unsymmetric 1,1-diboryl alkenes to render chiral 1,1-diboryl alkanes (Fig. 1B)^18^. Once again, the starting materials in this strategy have to be presynthesized from alkynes and the chiral cobalt complexes are presynthesized as well. Given the prevalence and ready accessibility of alkynes and the principals of green chemistry on trimming down the synthetic steps, we envision alkynes could be a very good starting point for the construction of chiral 1,1-diboryl alkanes based on our previous experience on the synthesis of racemic 1,1-diborons. In order to build the optically pure 1,1-diborons, transition-metal catalyzed hydroboration of alkynes with HBR_2_ as both boron source and hydrogen source should be the ideal solution for this goal. However, there are several significant challenges in our proposed protocols: mono-hydroboration might be the dominant one^19^; homo-diboration might occur and will be a trouble for purification^20,21^; regioselectivity is always an issue on the functionalization of alkynes^22^; last but not the least, the stereocontrol will be a great challenge to surpass with suitable metal catalyst and appropriate chiral ligands. Our previous experience on transition metal-free multi-functionalization of alkynes^23–33^ and cobalt-catalyzed cascade hydrosilylation and hydroboration of alkynes to access enantioenriched 1,1-silylboryl alkanes (Fig. 1C)^34^ led to speculation that the incorporation of transition metal with appropriate choice of double hydroboration partners will be crucial to overcome the aforementioned issues.

Here, we show a general and efficient strategy to construct enantioenriched 1,1-diboryl alkanes via a cascade copper-catalyzed double hydroborations of readily available alkynes (Fig. 1D). The strategy offers a straightforward method and great value in streamlining access to the important chiral 1,1-diboryl alkanes in a manner that complements the existing method. This system takes advantage of the in situ formation of the key terminal alkenylBdan intermediate, followed by second enantioenriched hydroboration with HBpin (Bpin, pinacolato) with two steps sharing one catalytic system. This reaction is compatible with terminal alkynes with a variety of substituents (including aliphatic and aromatic ones)^35–39^ and demonstrates high regio- and stereoselectivity during diborylations. Further mechanistic studies and DFT calculations help us to gain insights on the mechanism of beautiful regio- and stereoselectivity.

## Results and discussion

### Optimization studies of racemic process

Our studies began to investigate the reaction of racemic process by using 4-phenyl-1-butyne (**1**) as the testing substrate. A series of conditions with 2,3-dihydro-1H-naphtho[1,8-de][1–3]diazaborinine (HBdan) as the borylation source, pinacolborane (HBpin) as another borylation reagent, NEt_3_ as the base, Co(acac)_2_ as catalyst and Xantphos as ligand were examined, which was successfully rendering 68% yield of target product **4** (Table 1, entry 1). Subsequently, various metal catalysts were investigated, Co(acac)_2_ emerged as the optimal catalyst (Table 1, entries 2-3). After exploration of the other parameters like ligands, solvents and additives, the concentration of the reaction and the reactants adding-orders (Table 1, entries 4-10, also see Supplementary Tables 1 to 5 for details), the best reaction conditions were identified as 0.2 mmol of alkyne, 0.24 mmol of HBdan, 0.6 mmol of HBpin, 4 mol % of Co(acac)_2_, 4 mol% of Xantphos and 3.0 equiv of NEt_3_ in 0.2 mL of cyclohexane for 12 h (Table 1, entry 9).

**Table 1:** Discovery and parameter evaluation of the Co-catalyzed racemic cascade reaction^a,b^.

Entry	Catalyst	Ligand	Solvent	Additive	Yield (%)^b^
1	Co(acac)_2_	Xantphos	heptane	NEt_3_	68
2	Co(acac)_3_	Xantphos	heptane	NEt_3_	63
3	Cu(acac)_2_	Xantphos	heptane	NEt_3_	60
4	Co(acac)_2_	1.10-Phen	heptane	NEt_3_	N.D.
5	Co(acac)_2_	Xantphos	heptane	NHEt_2_	67
6	Co(acac)_2_	Xantphos	heptane	2,6-lutidine	66
7^c^	Co(acac)_2_	Xantphos	heptane	NEt_3_	70
8^c^	Co(acac)_2_	Xantphos	THF	NEt_3_	17
9^c^	Co(acac)_2_	Xantphos	cyclohexane	NEt_3_	76
10^c, d^	Co(acac)_2_	Xantphos	cyclohexane	NEt_3_	70

^a^After Co(acac)_2_ (4 mol%), ligand (4 mol%), HBdan (0.24 mmol) and amine (0.2 mmol) were mixed in 0.2 mL of solvent at room temperature for 15 mins, HBpin (0.3 mmol) and **1** (0.2 mmol) were added subsequently at room temperature, then the resulting mixture was stirred at ambient temperature for 12 h, ^b^Isolated yield, ^c^NEt_3_ (0.6 mmol), ^d^HBpin and **1** add after 8 mins.

### Substrate scope for racemic process

**Fig. 2:**
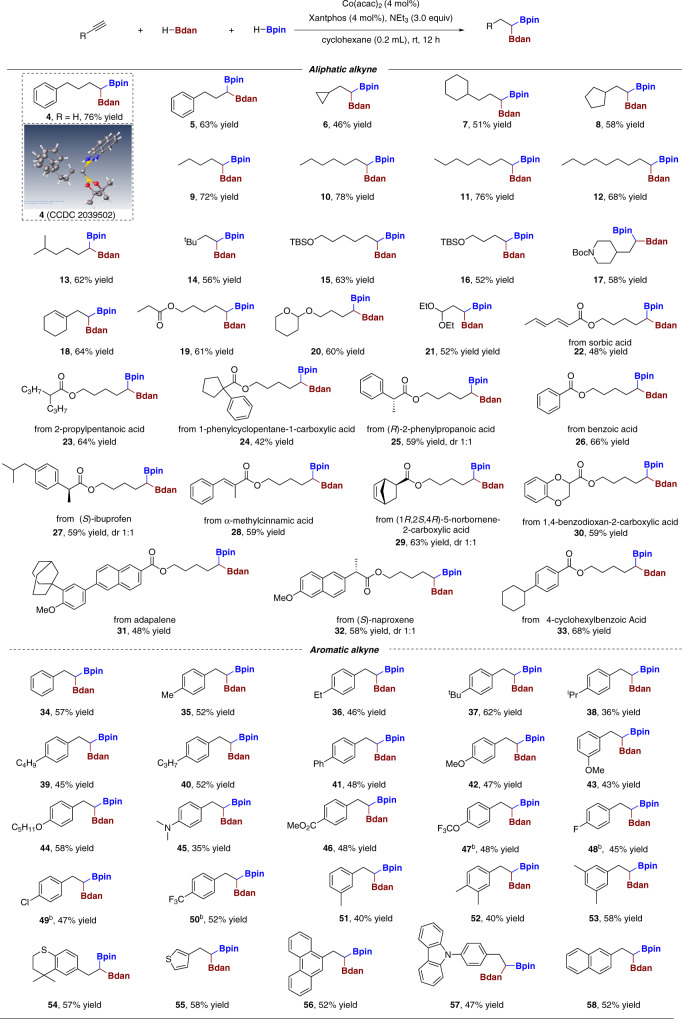
Scope of the racemic reaction^a^. Reaction conditions: ^a^After Co(acac)_2_ (4 mol%), Xantphos (4 mol%), HBdan (0.24 mmol) and NEt_3_ (0.6 mmol) were mixed in cyclohexane (0.2 mL, 1 M) at room temperature for 10 min, HBpin (0.3 mmol) and **1** (0.2 mmol) were added subsequently at room temperature, then the resulting mixture was stirred at ambient temperature for 12 h. ^b^50 °C.

Achieving the optimal conditions with non-chiral transformation, we started to evaluate the universality of the optimized conditions (Table 1, entry 9) in preparing racemic 1,1-diborons, the substrate scope was illustrated Fig. 2. Various unactivated aliphatic or aromatic alkynes were investigated to afford the corresponding racemic 1,1-diborons with ideal reaction outcomes. The molecular structure of diboron **4** was confirmed by X-ray crystallographic analysis (CCDC 2039502, see Supplementary Fig. 15 for details). The influence of different carbon chain length of aliphatic alkynes was studied and the products **4**–**14** were obtained in decent yields respectively. The substrates bearing bulky isopropyl and cycloalkyl groups were also good candidates to deliver the targeted products in satisfactory yields (**6**,**8**). Alkynes which bear various functional groups, such as silyl-protected alcohol, diethoxypropyl, and ester were all compatible and the respective products (**15**–**16, 18**–**33**) were obtained in good yields. It is worthwhile to find that *N*-Boc-4-ethynylpiperidine can also react smoothly under our standard conditions (**17**). Of note, the olefin moiety (**18**) does not interfere with the reaction and remains intact, indicating that the reaction has good chemical selectivity and ideal regional selectivity. High functional compatibility forced us to extrapolate our strategy to bioactive or therapeutic agents (Fig. 2). Various bioactive molecules or medicinal agents were derived into the corresponding aliphatic alkynes, all of them could render the respective targeted products in very good response under our standard conditions (for instance, sorbic acid (**22**), 2-propylpentanoic acid (**23**), 1-phenylcyclopentane-1-carboxylic acid (**24**), (R)-2-phenylpropanoic acid (**25**), benzoic acid (**26**), (S)-ibuprofen (**27**), α-methylcinnamic acid (**28**), (1 S,2 S,4 R)-5-norbornene-2-carboxylic acid (**29**), 1,4-benzodioxan-2-carboxylic acid (**30**), adapalene (**31**), naproxene (**32**) and gemfibrozil (**33**)). Gratifyingly, C-C double bond could well retain in our system, which also proved that this method has excellent chemoselectivity (**22**, **28** and **29**).

Not only aliphatic terminal alkynes gave very good response, the double hydroborations could also apply to versatile aromatic acetylenes. Overall, the standard conditions tolerated both electron-rich and electron-deficient functional groups on the aromatic rings (Fig. 2, bottom). When the aromatic ring bearing different substituents like Me, Et, ^*t*^Bu, ^*i*^Pr, C_4_H_9_, C_3_H_7_, Ph, OMe, OC_5_H_11_, NMe_2_, COOEt, the reaction could proceed smoothly and the expected products **35**–**46** were obtained in decent yields. It is noteworthy that the lower yields afforded in reactions with aryl alkynes bearing halogens on the *para* position, such substrates could be rectified by increasing the reaction temperature to 50 °C (**47**–**53**). Moreover, alkynes bearing other aromatic substituents such as dimethylthiochroman (**54**), thiophene (**55**), phenanthrene (**56**), 4-carbazolebenzene (**57**) and 2-naphthyl (**58**) were good candidates as well, rendering the targeted products in nice yields.

### Optimization studies of asymmetric process

Inspired by the success in racemic synthesis of 1,1-diborons, we decided to interprete this racemic transformation to an enantioselective process. Initially, we used Co(acac)_2_ as the catalyst and evaluated the chiral diphosphine ligands, and it turned out that Walphos was the best one by a series of trials. However, after extensively screening, we still struggled the low yields and unsatisfactory ee values. Given the influence of metals on the reaction outcomes, we decided to reevaluate metal catalysts (such as Co(acac)_2_, Co(acac)_3_, Cu(acac)_2_)., we were pleasantly surprised that Cu(acac)_2_ could provide the optimal result with the enantioenriched 1,1-diborons **59** in 76:24 er (Table 2, entry 4). Encouraged by the desired performance of Xantphos in reactions above, a series of structurally relevant chiral diphosphine ligands were investigated (Table 2, entries 4-8, see Supplementary Table 8 for more details), whereas (R,R)-Me-Duphos and (R,R)-Me-Ferrocelane were found to be ineffective to our reaction (Table 2, entries 7-8). After screening a series of solvents (see Supplementary Table 9 for details), no better results were obtained than cyclohexane (1.0 mL) (Table 2, entry 9). After determining the best solvent, we screened the (R,R)-Walphos with different substitutions, and found that (R,R)-Walphos was still the best ligand. Then we did more research and testing about additives, the amount of catalysis and ligands, different adding-ordering and reaction time (see Supplementary Tables 10, 13 and 14 for details).

**Table 2:** Discovery and evaluation of the Cu-catalyzed asymmetric cascade process^a^.

Entry	Catalyst	Ligand	Solvent (mL)	Additive (equiv)	Yield (%)^b^	er ^c^
1	Co(acac)_2_	Xantphos	THF (0.2)	—	76	—
2	Co(acac)_2_	(R,R)-Walphos	THF (0.2)	—	33	39:61
3	Co(acac)_3_	(R,R)-Walphos	THF (0.2)	—	28	35:65
4	Cu(acac)_2_	(R,R)-Walphos	THF (0.2)	—	20	76:24
5	Cu(acac)_2_	(R,S)-Josiphos	THF (0.2)	—	25	32:68
6	Cu(acac)_2_	(R)-tol-BINAP	THF (0.2)	—	15	45:55
7	Cu(acac)_2_	(S,S)-Me-duphos	THF (0.2)	—	n.d.	—
8	Cu(acac)_2_	(R,R)-Me-ferrocelane	THF (0.2)	—	n.d.	—
9	Cu(acac)_2_	(R,R)-Walphos	Cyclohexane (1.0)	—	33	88:12
10	Cu(acac)_2_	(R,R)-CH_3_-Walphos	Cyclohexane (1.0)	—	16	72:28
11	Cu(acac)_2_	(R,R)-^t^Bu-Walphos	Cyclohexane (1.0)	—	17	62:38
12	Cu(acac)_2_	(R,R)-CF_3_-Walphos	Cyclohexane (1.0)	—	18	86:14
13	Cu(acac)_2_	(R,R)-Nap-Walphos	THF (0.2)	—	23	84:16
14^d^	Cu(acac)_2_	(R,R)-Walphos	Cyclohexane (1.0)	PMHS (1.0)	78	94:6

^a^After catalyst (4 mol%), ligand (4 mol%), HBdan (0.24 mmol) were mixed in 0.2 mL of THF at room temperature for 15 min, HBpin (0.3 mmol) and **1** (0.2 mmol) were added subsequently at room temperature, then the resulting mixture was stirred at ambient temperature for 24 h.

^b^Isolated yield.

^c^The enantioselectivity was determined by Chiral HPLC.

^d^After catalyst (6 mol%), ligand (6 mol%), HBdan (0.24 mmol) and PMHS (1.0 equiv) were mixed in 1.0 mL of cyclohexane at room temperature for 10 min, HBpin (0.3 mmol) and **1** (0.2 mmol) were added subsequently at room temperature, then the resulting mixture was stirred at ambient temperature for 60 h.

We found the reaction in the absence of PMHS, and the desired product could be obtained as well, however the yield and ee value were not satisfactory (33% yield with 88:12 er). Inspired by Engle’s work^*[10]*^, we studied the amount of PMHS and other base (see Supplementary Table 11 for details), and we found that the addition of PMHS is very important to the success of our transformation, and one equivalent of PMHS is required in order to get good yield of product, however, PMHS has little influence on the selectivity of our transformation^40^. Based on our previous work (Fig. 1C)^34^, we realized that the sequence and the adding time will influence the efficiency of the transformation, therefore, we performed control experiments careful to figure out the length of adding time (see Supplementary Table 15 for details). It clearly demonstrated the importance of the time control in this transformation, and 10 mins after HBdan and PMHS addition with catalyst and ligand led to desired product with both good yield and good enantioselectivity. We think that the result might stem from the initial formation of Cu-H species from Cu catalyst and HBdan and PMHS. Further DFT calculations also suggest that the formation of intermediate alkenyl-Bdan is very important, otherwise, 1,1-diBpin will be an inevitable byproduct which origins from the intermediate alkenyl-Bpin, therefore, the addition of HBdan prior to HBpin is crucial to the good yields and enantioselectivity. Finally, we identified the best conditions as: After Cu(acac)_2_ (6 mol%), Walphos (6 mol%), HBdan (0.24 mmol) and PMHS (1.0 equiv) were mixed in 1.0 mL of cyclohexane at room temperature for 10 min, HBpin (0.3 mmol) and **1** (0.2 mmol) were added subsequently at room temperature, then the resulting mixture was stirred at ambient temperature for 60 h. The desired chiral product **59** was obtained in 78% yield with 94:6 er (Table 2, entry 14).

### Substrate scope in asymmetric transformation

**Fig. 3:**
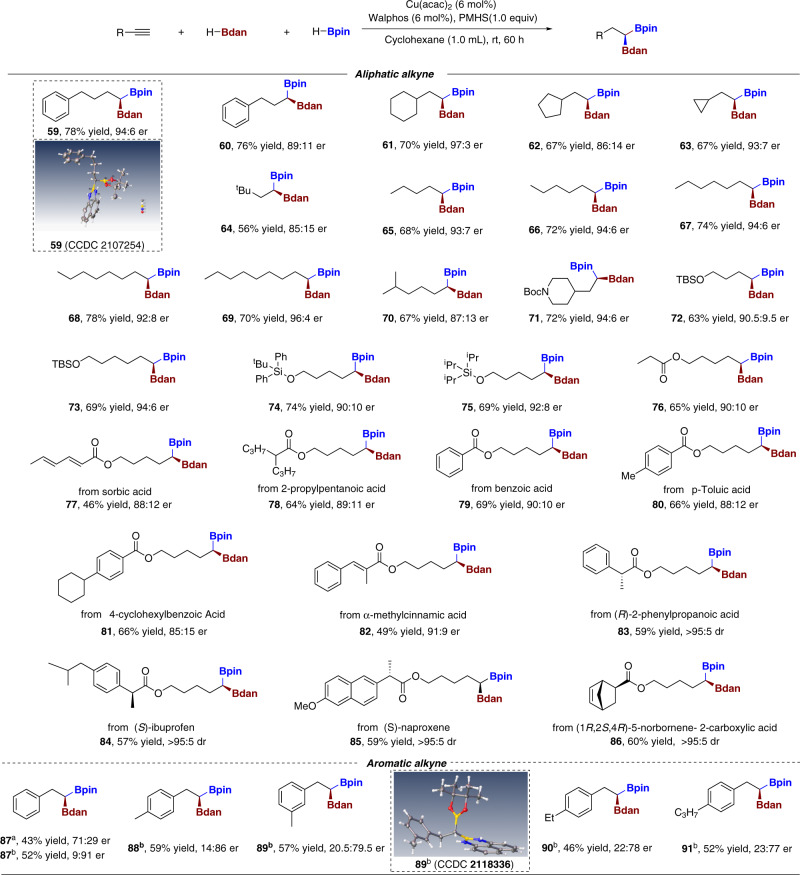
Scope of the chiral reaction^a^. Reaction conditions: ^a^After Cu(acac)_2_ (6 mol%), Walphos (6 mol%), HBdan (0.24 mmol) and PMHS (0.2 mmol) were mixed in cyclohexane (1.0 mL) at room temperature for 10 min, HBpin (0.3 mmol) and **1** (0.2 mmol) were added subsequently at room temperature, then the resulting mixture was stirred at ambient temperature for 60 h. ^b^Josiphos (6 mol%).

Having established the conditions for enantioselective reaction, we began to examine the reaction range and the results were depicted in Fig. 3. To our delight, like their racemic congeners, various unactivated aliphatic and aromatic alkynes were investigated and smoothly deliver the corresponding chiral 1,1-diborons with desired reaction outcomes. Aliphatic alkynes with different carbon chain length were also examined to afford the desired products in good yields with high levels of stereoinduction (**59**–**70**). The molecular structure of asymmetric diboron **59** was confirmed by X-ray crystallographic analysis (CCDC 2107254, see Supplementary Fig. 16 for details). Of note, this asymmetric reaction could tolerate a variety of functionalities installed on the carbon chain such as Boc-protected amine (**71**), silyl-protected alcohol (**72**–**75**) and esters (**76**–**87**), this high functionality compatibility encouraged us to expand our synthetic scheme to the late stage elaborations of bioactive or pharmaceutic agents. Upon treatment with the standard conditions, a series of alkynes derived from sorbic acid, 2-propylpentanoic acid, benzoic acid, *p*-toluic acid, 4-cyclohexylbenzoic acid, α-methylcpiinnamic, gemfibrozil, (R)-2-phenylpropanoic acid, (S)-ibuprofen, (S)-naproxen and (1 S,2 S,4 R)-5-norbornene-2-carboxylic acid all rendered corresponding enantioenriched chiral *gem*-diborylalkanes in decent yields with both high regio- and stereoselectivities (**77**–**86**). The successful elaborations of these bioactive or therapeutic agents certified the mildness and broad functional group compatibility of this asymmetric transformation.

Of note, compared with the insensitivity to structural variation for aliphatic alkynes on enantioselectivity in the asymmetric conditions, aryl alkynes varied significantly. When we use phenylacetylene as substrate under the aforementioned standard condition, we can only achieve the target product **87** in 43% yield and 71:29 er. Through a series of optimization studies of process, we identified Josiphos as the best chiral diphosphine ligand which could provide 52% yield and 91:9 er in target product **87**. The molecular structure of asymmetric diboron **89** was confirmed by X-ray crystallographic analysis (CCDC 2118336, see Supplementary Fig. 17 for details). Chiral functionalized products with Me, Et, C_3_H_7_ and substituents were prepared in useful yields and good er values (**88**–**91**).

### Mechanistic studies

**Fig. 4:**
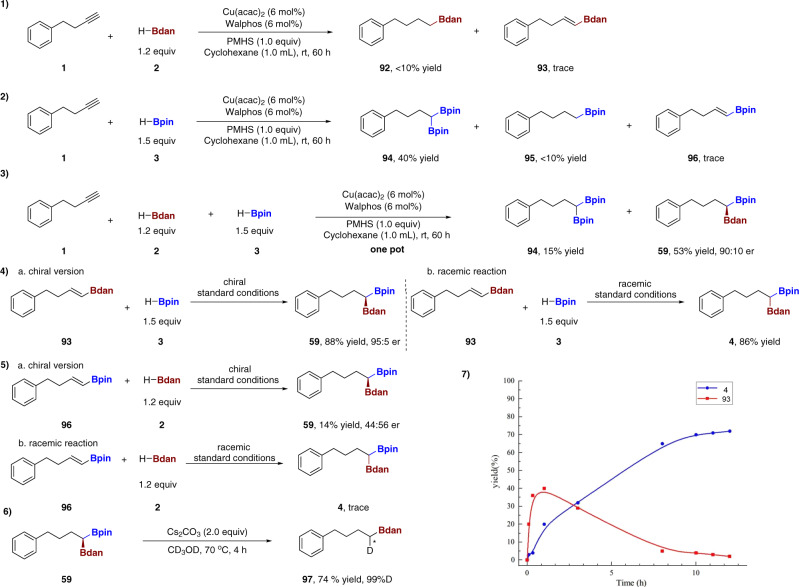
Control experiments. (1) **1** was exposed to HBdan in the absence of HBpin, under the standard reaction conditions. (2) **1** was exposed to HBpin in the absence of HBdan, under the standard reaction conditions. (3) One-pot reaction to the target product **59**. (4) alkenyl-Bdan **93** and HBpin was exposed under the standard reaction conditions. (5) alkenyl-Bpin **96** and HBdan was exposed under the standard reaction conditions. (6) Deuterium experiment. (7) the yield changes of alkenyl-Bdan **93** and targeted product **4** over the reaction time.

Since our experimental results show that the reaction is advantageous to the formation of 1,1- bifunctionalized product over other byproducts, we then studied the origin of significant regioselectivity and stereoselectivity in this transformation. When **1** was subjected to HBdan in the absence of HBpin, trace amount of alkenyl-Bdan **93** was detected by gas chromatography (GC) analysis with dodecane as internal standard under the standard reaction conditions (Fig. 4, eq. 1). Of note, when only HBpin was added, 1,1-diboron alkane **94** was detected in 40% yield by GC analysis, meanwhile, alkyl Bpin **95** was detected with trace amount of alkenyl-Bpin **96** (Fig. 4, eq. 2). Moreover, where HBdan, HBpin and alkyne were added simultaneously at the beginning of reaction, the target product **59** was detected in 53% yield with 90:10 er with 15% yield of 1,1-diboron alkane **94** detected (Fig. 4, eq. 3). These outcomes indicated two feasible routes, that is whether alkenyl-Bdan **93** or alkenyl-Bpin **96** was built first for our transformation. To figure out the plausible pathway, we prepared the two possible intermediates alkenyl-Bdan **93** and alkenyl-Bpin **96** using the standard conditions for asymmetric synthesis. Under chiral standard conditions, alkenyl-Bdan **93** reacted with HBpin smoothly to afford **59** in 88% yield and 95:5 er (Fig. 4, eq. 4a) but the reaction of alkenyl Bpin **96** with HBdan only in 14% yield and 44:56 er to give diboron product **59** (Fig. 4, eq. 5a). When exposed to the racemic standard conditions, similar results were obtained: alkenyl-Bdan **93** reacted with HBpin smoothly to afford **4** in 86% yield (Fig. 4, eq. 4b) but the reaction of alkenyl-Bpin **96** with HBdan did not proceed and only afforded trace amount of targeted product **4** (Fig. 4, eq. 5b). Exposure of **59** under basic conditions in the presence of a large excess of CD_3_OD (0.5 mL) led to the selective protodeboration of the Bpin moiety, affording the Bdan-substituted alkane **97** in 74% yield and 99% D (Fig. 4, eq. 6)^41^.

### Mechanistic investigations

**Fig. 5:**
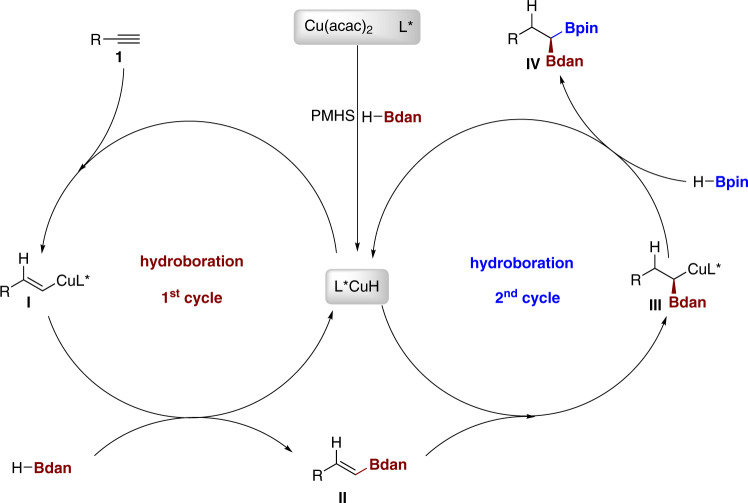
The proposed mechanisms. The reaction proceeds through 1^st^ hydroboration cycle with HBdan and 2^nd^ hydroboration cycle with HBpin.

According to the previous report^2,9–12,16,13,17–19,40,42–44^ and control experiments, we put forward the possible reaction mechanism about the copper-catalyzed enantioselective double hydroboration of alkynes (Fig. 5). The first hydroboration is started with the formation of copper hydride species from Cu(acac)_2_, ligand, HBdan and PMHS. Alkyne **1** reacts with the copper hydride species by Cu–H bond insertion to generate vinyl copper species **I**, which undertakes a σ-bond metathesis with HBdan to deliver vinyl Bdan **II** and release Cu-H species for finishing the first cycle and starting the second catalytic cycle. Vinyl Bdan **II** reacts with Cu–H species once again to generate alkyl copper species **III**, which further undertakes σ-bond metathesis with HBpin. The release of product **IV** and regeneration of Cu–H complex fulfill the catalytic cycle for the second hydroboration.

**Fig. 6:**
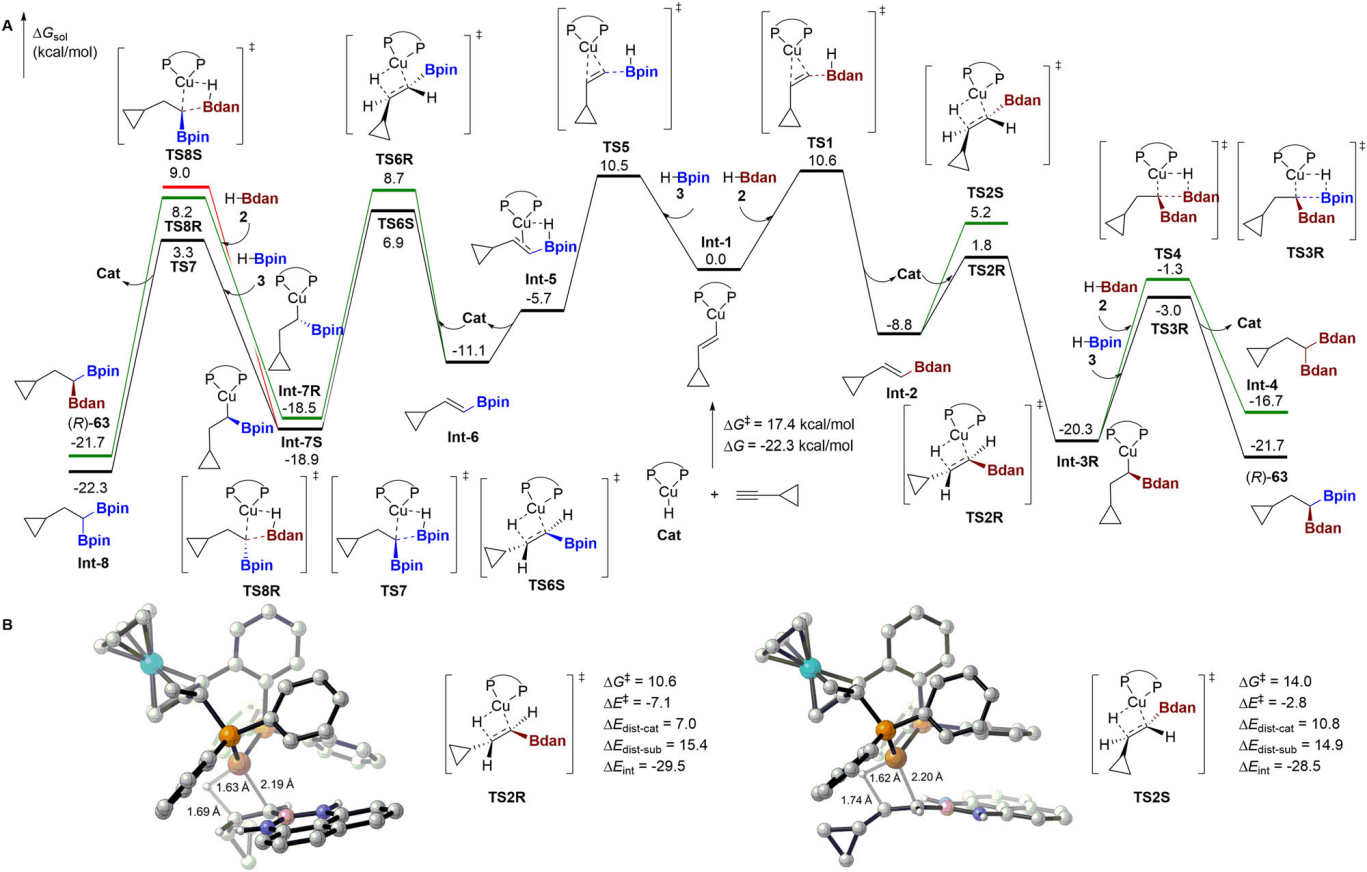
DFT calculations on the proposed reaction pathway. **A** Free energy profile for the competing hydroboration cycles. **B** Stereoselectivity-determining transition state structures.

We next performed density functional theory (DFT) calculations to further validate our proposed mechanism (SMD-M06L/6-311+G(d,p)/SDD(Cu,Fe)//B3LYP-D3/6-31G(d)/SDD(Cu,Fe), computational details can be found in the Supplementary information 1). The structurally simplest cyclopropyl alkyne was chosen as the model substrate and the results are shown in Fig. 6. The hydroboration cycles begin with the coordination of the alkyne substrate to the copper hydride (**Cat**), which undergoes regioselective alkyne insertion into the Cu–H bond via a four-membered transition state (structure not shown here, see Supplementary Fig. 1 for details) to generate vinyl copper species **Int-1**. The computed free energy of activation (Δ*G*^*‡*^) for this step is 17.4 kcal/mol and the free energy of reaction (Δ*G*) is -22.3 kcal/mol, in accordance with previous computational results^10^. **Int-1** could competatively react with H–Bdan **2** or H–Bpin **3**, undergoing either concerted or stepwise σ-bond metathesis processes via **TS1** or **TS5** to afford key alkenyl boron intermediates **Int-2** or **Int-6**, respectively, and regenerate the active copper hydride catalyst. Alkenyl Bdan **Int-2** can subsequently react with the copper hydride catalyst (**Cat**) using either of its two faces (via **TS2R** or **TS2S**) to generate chiral alkyl copper intermediates **Int-3R** or **Int-3S**, respectively. **TS2R** that ultimately leads to the major enantiomeric product (*R*)-**63** is energetically favored than **TS2S**. **Int-3R** then undergoes chemoselective σ-bond metathesis with H–Bpin **3** via **TS3R** with retention of stereochemistry to furnish the catalytic cycle. The competing reaction of H–Bdan **2** with **Int-3R** via **TS4** is higher in energy both kinetically and thermodynamically, in agreement with the experimental observation that **Int-4** is not formed. In the competing pathway that initially forms alkenylBpin **Int-6**, the subsequent 2^nd^ hydroboration favors the ultimate formation of 1,1-diboron species **Int-8** via **TS7**, which is the main side product observed experimentally. The reaction between **Int-7** with H–Bdan **2** is disfavored with a much higher barrier and poor enantioselectivity. This result agrees well with the control experiment that the reaction between alkenyl boron **96** and H–Bdan **2** only afforded the corresponding product with 14% yield and 44:56 er. Overall, although the energies of the competing first hydroboration are comparable between alkenyl copper species **Int-1** with H–Bpin **3** or H–Bdan **2**, its subsequent barrier via **TS6S** (18.0 kcal/mol) is higher than that of **TS2R** (10.6 kcal/mol). Taken together with the computed chemo- and enantioselectivities, and the results from the corresponding control experiments, the proposed mechanism for the formation of the main product is supported. The computational results also predicted the unavoidable formation of the side product, which rationalized the moderate yields of these reactions. To further probe the origin of the enantioselectivity for the hydrocupration process, we performed distortion/interaction analysis^45^ on **TS2R** and **TS2S** (Fig. 6B). The transition state structures were separated into two fragments that correspond to the Cu-H catalyst and the vinyl Bdan substrate, respectively. We found that the distortion energy of the catalyst (Δ*E*_dist-cat_) is the main contributor to the enantioselectivity. The catalyst pocket accommodates one face of the approaching vinyl Bdan substrate better than the other. The relatively large and rigid structure of the planar Bdan group causes the phenyl group on the phosphine ligand to distort upwards in the disfavored **TS2S**, resulting in a larger distortion energy of the catalyst (10.8 kcal/mol) than that of **TS2R** (7.0 kcal/mol).

In summary, we disclose a Cu-catalyzed enantioselective 1,1-diboration reaction from two different boranes and readily accessible terminal alkynes. This method demonstrates commendable regio-, chemo- and enantioselectivity, and could deliver valuable chiral *gem*-bis(boryl)alkanes with one catalysis and one ligand pattern from simple terminal alkynes and two different borane, which represents the most simple and straightforward strategy for the construction of such chiral *gem*-diborons. We think that this research will encourage and intrigue efforts for the synthesis of derived enantioenriched borylalkanes, and provide new avenue for the asymmetric bifunctionalization of alkynes. DFT calculation illustrates the beautiful regioselectivity, chemoselectivity and enantioselectivity.

## Methods

### General procedure A for synthesis of racemic *gem*-diborylalkane

A tube was charged with Co(acac)_2_ (4 mol%) and Xantphos (4 mol%) under N_2_ atmosphere, then cyclohexane (0.2 mL), HBdan (0.24 mmol) and NEt_3_ (0.6 mmol) were added subsequently. The reaction mixture was stirred at room temperature for 15 mins. Then HBpin (0.3 mmol) and **1** (0.2 mmol) were added subsequently at room temperature, then the resulting mixture was stirred at room temperature for 12 h. The residue was purified by column chromatography to afford the corresponding *gem*-diborylalkanes **4**.

### General procedure B for synthesis of racemic *gem*-diborylalkane

A tube was charged with Co(acac)_2_ (4 mol%) and Xantphos (4 mol%) under N_2_ atmosphere, then cyclohexane (0.2 mL), HBdan (0.24 mmol) and NEt_3_ (0.6 mmol) were added subsequently. The reaction mixture was stirred at room temperature for 15 mins. Then HBpin (0.3 mmol) and phenylacetylene containing halogen (0.2 mmol) were added subsequently at room temperature, then the resulting mixture was stirred at 50 °C for 12 h. The residue was purified by column chromatography to afford the corresponding product *gem*-diborylalkanes **47**.

### General procedure C for synthesis of chiral *gem*-diborylalkane

A tube was charged with Cu(acac)_2_ (6 mol%) and Walphos (6 mol%) under N_2_ atmosphere, then cyclohexane (1.0 mL), HBdan (0.24 mmol) and PMHS (1.0 equiv) were added subsequently. The reaction mixture was stirred at room temperature for 10 mins. Then HBpin (0.3 mmol) and **1** (0.2 mmol) were added subsequently at room temperature, then the resulting mixture was stirred at ambient temperature for 60 h. The residue was purified by column chromatography to afford the corresponding product **(R)-59**.

### General procedure D for synthesis of chiral *gem*-diborylalkane

A tube was charged with Cu(acac)_2_ (6 mol%) and Josiphos (6 mol%) under N_2_ atmosphere, then cyclohexane (1.0 mL), HBdan (0.24 mmol) and PMHS (1.0 equiv) were added subsequently. The reaction mixture was stirred at room temperature for 10 mins. Then HBpin (0.3 mmol) and phenylacetylene (0.2 mmol) were added subsequently at room temperature, then the resulting mixture was stirred at ambient temperature for 60 h. The residue was purified by column chromatography to afford the corresponding product **(S)-87**.

## Supplementary information

Supplementary information

## Data Availability

All data generated or analyzed during this study are included in this Article and its Supplementary Information. Crystallographic data have been deposited at the Cambridge Crystallographic Data Centre (CCDC) as CCDC 2039502 (**4**), 2107254 (**59**) and 2118336 (**89**) can be obtained free of charge from the CCDC via www.ccdc.cam.ac.uk/getstructures. Experimental procedures, characterization of new compounds, and DFT calculations (see Supplementary Data 1 for XYZ coordiantes) are available in the Supplementary information.
