# Publisher Correction: Enantioselective Cu-catalyzed double hydroboration of alkynes to access chiral gem-diborylalkanes

**DOI:** 10.1038/s41467-022-32433-7

**Published:** 2022-08-22

**Authors:** Shengnan Jin, Jinxia Li, Kang Liu, Wei-Yi Ding, Shuai Wang, Xiujuan Huang, Xue Li, Peiyuan Yu, Qiuling Song

**Affiliations:** 1 Institute of Next Generation Matter Transformation, College of Material Sciences Engineering, Huaqiao University, Xiamen, Fujian 361021 China; 2 Department of Chemistry and Shenzhen Grubbs Institute, Southern University of Science and Technology, Shenzhen, 518055 China; 3 School of Chemistry and Chemical Engineering, Henan Normal University, Xinxiang, Henan 453007 China; 4 State Key Laboratory of Elemento-Organic Chemistry, Nankai University, Tianjin, China

**Keywords:** Synthetic chemistry methodology, Chemical synthesis

Correction to: *Nature Communications* 10.1038/s41467-022-31234-2 published online 20 June 2022

The original version of this Article contained an error in Fig. 6, in which the labelling of Int-1 and its relative energy are unclear.

The correct version of Fig. 6 is:
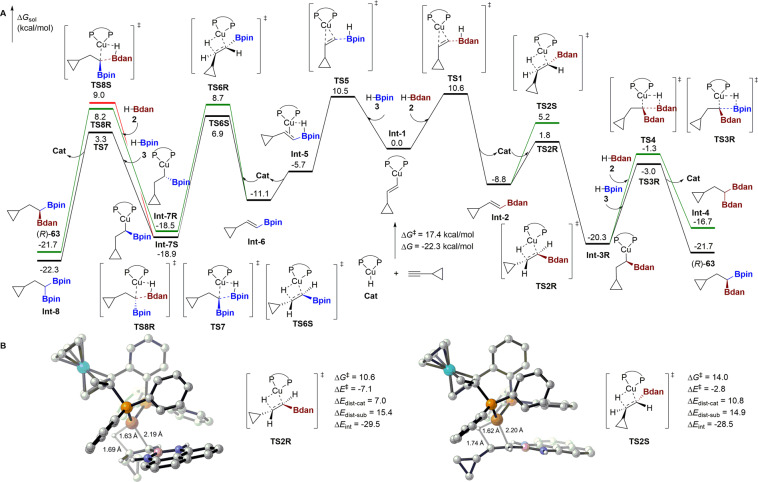


which replaces the previous incorrect version:
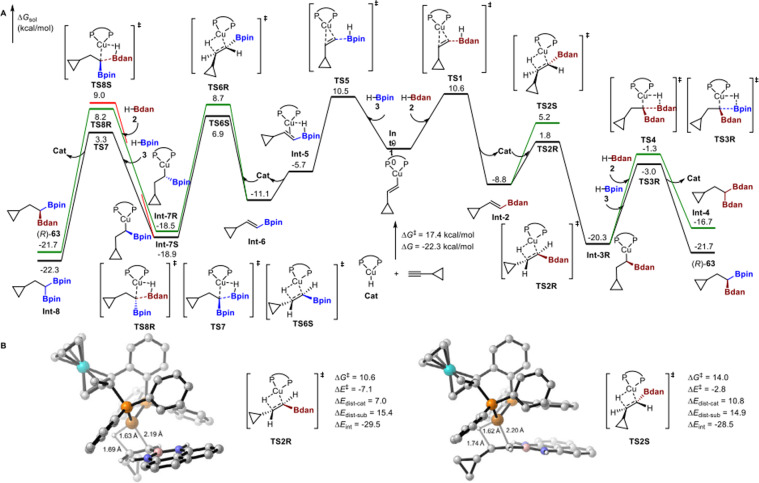


This has been corrected in both the PDF and HTML versions of the Article.

