# Supplementary material for: Enantioselective Cu-catalyzed double hydroboration of alkynes to access chiral gem-diborylalkanes
Source: Nat Commun. 2022 Jun 20;13:3524. doi: 10.1038/s41467-022-31234-2 (PMC9209482; doi:10.1038/s41467-022-31234-2)
Supplement: Supplementary file 1 — Supplementary information [file 41467_2022_31234_MOESM1_ESM.pdf]

Supplementary Information

Enantioselective Cu-catalyzed double hydroboration of alkynes to access chiral gem-diborylalkanes

Q. Song et al

Table of Contents

1. Supplementary Methods……………………………………..….…………………….…..……...3

1.1 General information……………………………………………………………………………3

2. Supplementary Disscussion…………………….……………………...4

2.1 Optimization of the reaction condition for racemic gem-diborylalkanes……………………………4

2.2 Optimization of the reaction conditions for asymmetric gem-diborylalkanes……………………..……6

2.3 General Process for the Synthesis gem-Diborylalkanes.…………………………..……............11

3. Supplementary Notes…….…………………….…………………............…….….…….........13

3.1 Mechanism Studies…….……….………………………………………….…………….. ……13

3.2. ^{31}P NMR Studies…….……….……………………………………….……………..…… …16

3.3 Crystal Data of **4**, **59** and **89**.……….…………………. ……………….…….……...… ……..…18

3.4 Characterization Data ……………...…………………………………………..……… ……....21

3.5 DFT Data………………………………………………………………………………..……57

4. Supplementary Figures…………………………………………………………………………….58

4.1. NMR Spectroscopic Data….……………………………………………………......…......58

4.2. Chiral HPLC charts….…………………………………………………………….......195

5. Supplementary References……………… ……………………………………………….......222

1. Supplementary Methods

1.1 General information

All reagents and solvents were purchased from Adamas Reagent, Energy Chemical Company, Bide Pharmatech Ltd., and Tansoole, and were used without further purification. Unless otherwise stated, all reactions were accomplished in Schlenk tubes under N_2 atmosphere. The reactions were monitored by thin layer chromatography (TLC) or gas chromatography-mass spectrometry (GC-MS). Flash column chromatography was performed over silica gel (200−300 mesh). ^{1}H NMR spectra were recorded on a Bruker Avance III 500 MHz (or 400 MHz) NMR spectrometer, and the chemical shifts (in ppm) were referred to $CDCl_3$ (δ = 7.26 ppm) as an internal standard. ^{13}C NMR spectra were obtained by using the same NMR spectrometer and were calibrated with $CDCl_3$ (δ = 77.0 ppm). ^{11}B NMR spectra were acquired with accessories on the same NMR spectrometer using $CDCl_3$. ^{19}F NMR spectra were acquired with accessories on the same NMR spectrometer using $CDCl_3$, too. The following abbreviations were used to illuminate the diversities: δ = chemical shifts, J = coupling constant, s = singlet, d = doublet, t = triplet, q = quartet, m = multiplet. High-resolution mass spectra (HRMS; (ESI)) were acquired with quadrupole and time-of-flight (TOF) mass spectrometers. All reagents and solvents were obtained from commercial suppliers and used without further purification. Reactions were monitored by thin-layer chromatography (TLC). The products were purified by column chromatography on silica gel using petroleum ether and ethyl acetate as the eluent. Analytical chiral HPLC was performed on an Agilent 1600 Infinity instrument with Daicel Chiralcel OD-H column, or Daicel Chiralpak AD-H, IA-3, IC-3 columns. Optical rotations were measured on an Aton Paar MCP 150 polarimeter.

2. Supplementary Discussion

2.1 Optimization of the racemic gem-diborylalkanes reaction condition

Supplementary Table 1 Catalysis Screening. Changing the catalysis with other parameters as the same.

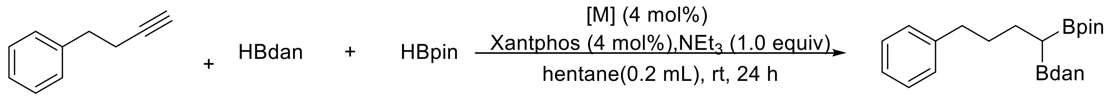

1   2 (1.2 equiv)   3 (1.5 equiv)   4

Entry	[M] (4.0 mol%))	Yield
1	$Co(acac)_2$	68
2	$Co(acac)_3$	63
3	$Cu(acac)_2$	60
4	$Co_2(CO)_8$	N.D.
5	Cobalt bis(2-ethylhexanoate)	N.D.
6	CoC	N.D.
7	$Co(TMHD)_3$	N.D.
8	Co_2CO_3	N.D.
9	$Co(acacF_6)_2$	50
10	Dicarbonylcyclopentadienylcobalt	N.D.

Supplementary Table 2 Ligand evaluations. Changing ligands with other parameters as the same.

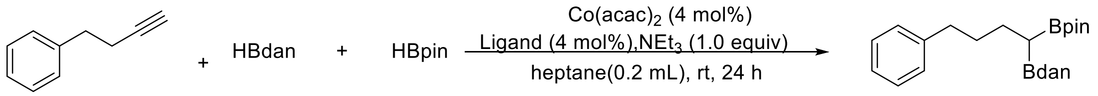

1   2 (1.2 equiv)   3 (1.5 equiv)   4

Entry	Ligand	Yield (%)
1	2,2'-Bipyridine	N.D.
2	1,10-Phen	N.D.
3	PPh_3	12
4	PPh_3O	trace
5	$P(OMe)_3$	N.D.
6	P^tBu_3	15
7	PPh_2O	trace
8	Xantphos	68

Supplementary Table 3 Additive examinations. Changing additives with other parameters as the same.

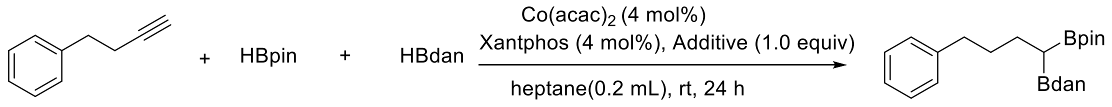

1a 1.5 equiv 1.2 equiv

Entry	Additive (1.0 equiv)	Yield (%)
1	Morpholine	16
2	Quinoline	<10
3	Pyridine	43
4	$HNEt_2$	67
5	NEt_3	68
6	N,N-Diethylaniline	65
7	Cyclohexylamine	52
8	N^tBu_3	67
9	N^iPr_3	35
10	2,6-Lutidine	66
11	NEt_3 (3.0)	70
12	NEt_3 (5.0)	35
13	NEt_3 (0.5)	57

Supplementary Table 4 Solvent screening. Changing solvents with other parameters as the same.

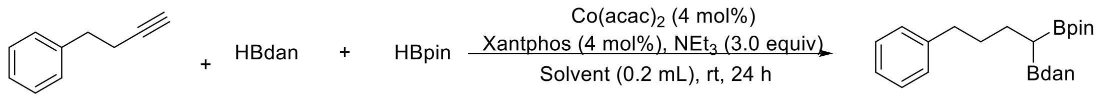

1 **2** 1.2 equiv **3** 1.5 equiv **4**

Entry	Solvent (0.2 mL)	Yield (%)
1	THF	17
2	Toluene	trace
3	Et_2O	16
4	1,4-dioxane	30
5	n-hexane	42
6	n-octane	62
7	heptane	70
8	pentane	59
9	Cyclopentane	52
10	Cyclohexane	76
11	Cycloheptane	65
12	Cyclooctane	67
10	Cyclohexane (1.0 mL)	70

Supplementary Table 5 Adding time of HBpin. Testing the reactions with different adding time of HBpin.

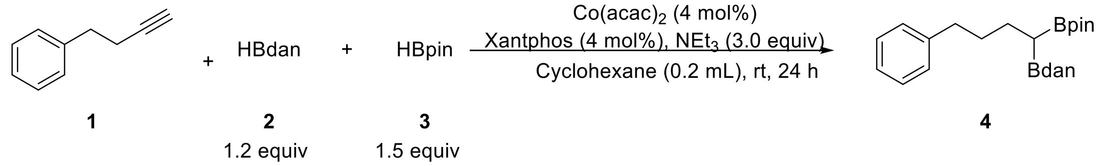

Entry	variation from standard conditions	Yield (%)
1	HBpin added together with 1 and HBdan	65
2	HBpin added after 8min	70
3	HBpin added after 15 min	76
4	HBpin added after 20 min	73

2.2 Optimization of the reaction conditions for chiral gem-diborylalkanes

Supplementary Table 6 Chiral ligand evaluations. Changing chiral ligands with other parameters as the same.

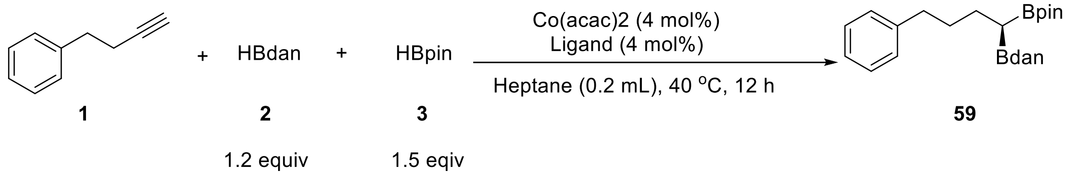

Entry	Ligand	Yield (%)	er (%)
1	(R)-binap	13	53:47
2	(R)-Tol-binap	<5	40:60
3	(R)-Xyl-binap	48	45:55
4	(S)-Segphos	8	55:45
5	(S)-DM-Segphos	8	40:60
6	(S)-DTBM-Segphos	24	40.5:59.5
7	(R,S)-josiphos	<5	—
8	(R)-(R)-Walphos	43	23:77
9	(S)-Sunphos	26	44.5:55.5
10	(R)-Difluorphos(TM)	trace	—

Supplementary Table 7 Catalysis examinations. Changing the catalysis with other parameters as the same.

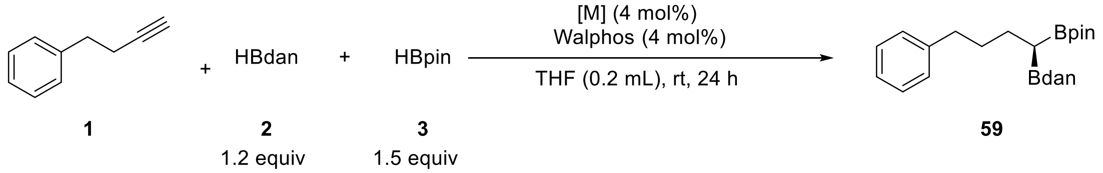

Entry	[M] (4.0 mol%)	Yield (%)	er (%)
1	$Co(acac)_2$	33	39:61
6	$Co(acac)_3$	28	35:65
7	$Cu(acac)_2$	20	76:24

Supplementary Table 8 Chiral ligand evaluations. Changing chiral ligands with other parameters as the same.

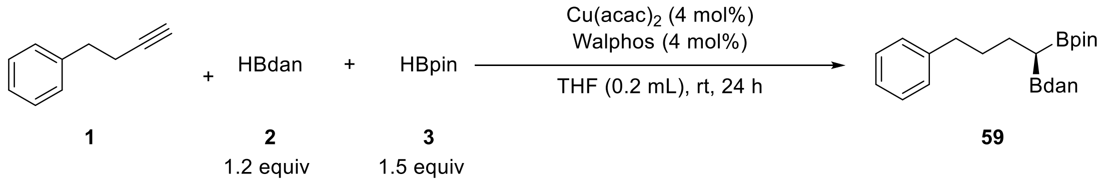

Entry	Ligand	Yield (%)	er
1	(R)-tol-binap	15	45:55
2	(R)-xyl-binap	12	42:58
3	(R)-DM-Segphos	13	48:52
4	(R)-Segphos	14	47:53
5	(S,S)-Ph-BPE	8	56:44
6	(R,R)-BDDP	20	64:36
7	(R,R)-quinoxP*	16	34:66
8	(S)-Sunphos	43	74:26
9	(R)-Difluorduphos	9	61:39
10	(R,R)-Me-ferrocelane	—	—
11	(R)-DTBM-MeOBiphep	8	37:63
12	(R,R)-Me-duphos	—	—
13	(R,S)-josiphos	25	32:68
14	(R)-(R)-Walphos	20	76:24

Supplementary Table 9 Solvent screening. Changing solvents with other parameters as the same.

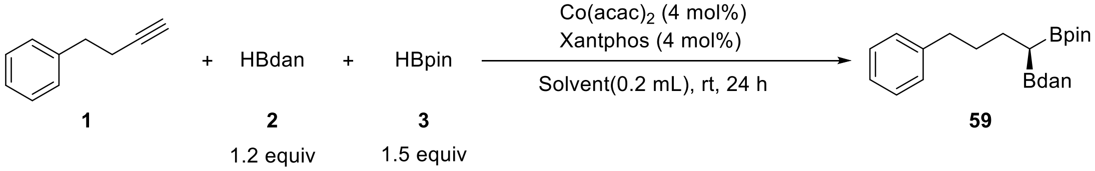

Entry	Solvent (0.2 mL)	Yield (%)	er
1	THF	20	76:24
2	Toluene	28	82:18
3	Et_2O	30	78:22
4	1,4-dioxane	32	83:17
5	Pentane	25	82.5:17.5
6	Hexane	30	80:20
7	n-octane	27	82:18
8	Heptane	32	83:17
9	cyclohexane	33	87:13
10	cyclohexane (1.0 mL)	30	88:12

Supplementary Table 10 Additive examinations. Changing additives with other parameters as the same.

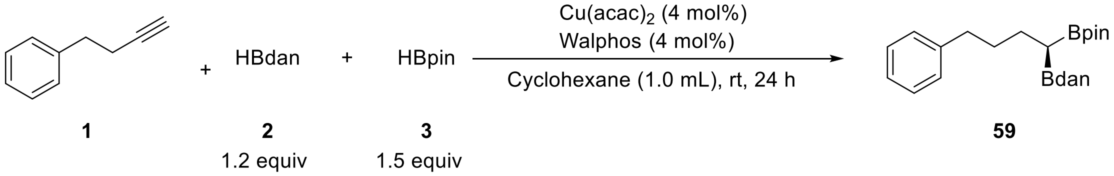

Entry	Additive (1.0 equiv)	Yield (%)	er
1	—	33	88:12
2	MeOH (1.0) + NaO^tBu (2.0)	n.d.	—
3	$(EtO)_2SiMeH$	70	89:11
4	$(MeO)_2SiMeH$	67	89:11
5	2,6-Lutidine	43	86:14
6	N^nBu_3	13	49:51
7	NEt_3	18	79:21
8	PMHS (1.0)	76	91:9
9	PMHS (3.0)	72	91:9

Supplementary Table 11 The amount of PMHS. Changing the amount of PMHS with other parameters as the same.

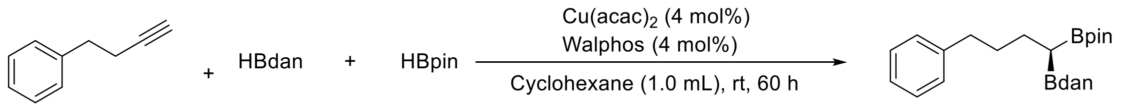

1 2 1.2 equiv 3 1.5 equiv 59

Entry	PMHS (equiv)	Yield (%)[a]	Yield (%)[b]	er (%)
1	—	40	33	88:12
2	0.2	51	46	89:11
3	0.5	60	50	91:9
4	1.0	80	76	91:9
5	2.0	83	73	91:9
6	3.0	85	72	91:9

[a] the yield of product **59** was determined with gas chromatography (GC) analysis with Dodecane as internal standard; [b] isolated yield; [c] The enantioselectivity was determined by Chiral HPLC.

Supplementary Table 12 The additive of PMHS and base. Changing the additive and base with other parameters as the same.

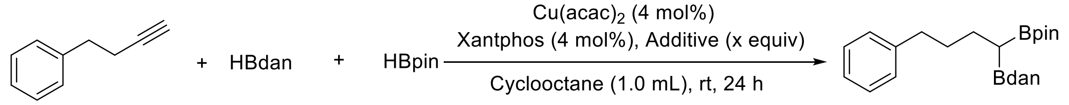

1a 2 1.2 equiv 3 1.5 equiv 59

Entry	Additive (equiv)	Yield (%)	er
1	PMHS (1.0)	76	91:9
2	PMHS (1.0) + LiO^{t}Bu (2.0)	32	88:12
3	PMHS (1.0) + NaO^{t}Bu (2.0)	10	54:46
4	PMHS (1.0) + KO^{t}Bu (2.0)	12	66:34
5	PMHS (1.0) +LiOMe (2.0)	17	81:19

Isolated yield.

Supplementary Table 13 The amount of catalysis and ligand. Changing the amount of catalysis and ligand with other parameters as the same.

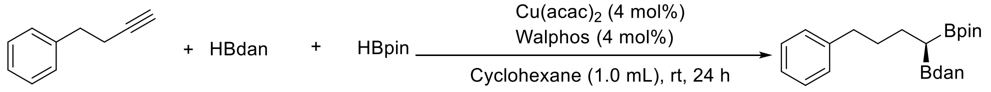

1 2 1.2 equiv 3 1.5 equiv 5 9

Entry	Cu(acac)$_2$ (mol%)	Walphos (mol%)	yield (%)	er
1	4	4	76	91:9
2	6	6	78	92:8
3	8	8	65	86:14
4	4	6	52	77:23

Supplementary Table 14 Adding sequence and spans examinations. Changing the adding sequence and spans with other parameters as the same.

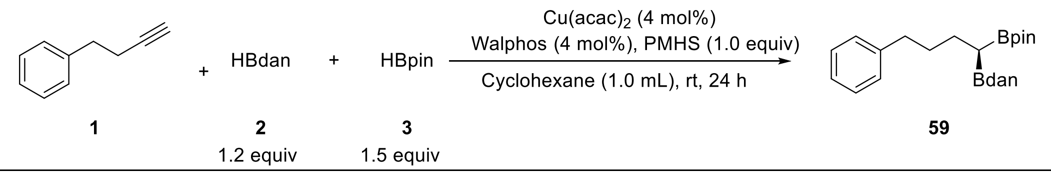

Entry	variation from standard conditions	yield (%)	er
1	**1** and HBpin added after 10 min	76	91:9
2	**1** and HBpin added after 15 min	73	89:11
3	**1** and HBpin added after 20 min	78	88:12
4	**1** and HBpin added after 37 min	80	85:15
5	**1** and HBpin added after 15 min (dry Cyclooctane)	74	85:15
6	one pot	53	88:12
7	HBpin added after 10 min	66	89:11

Supplementary Table 15 Time screening. Changing the reaction time with other parameters as the same.

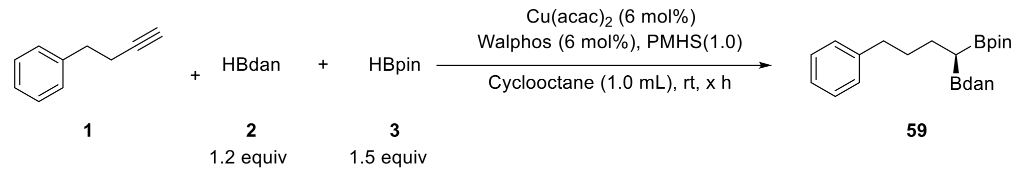

Entry	time (h)	yield (%)	er
1	24	78	92:8
2	36	77	92:8
3	48	76	92:8
4	60	78	94:6
5	72	78	92:8

2.3. General Process for the Synthesis gem-Diborylalkanes

General Procedure A:

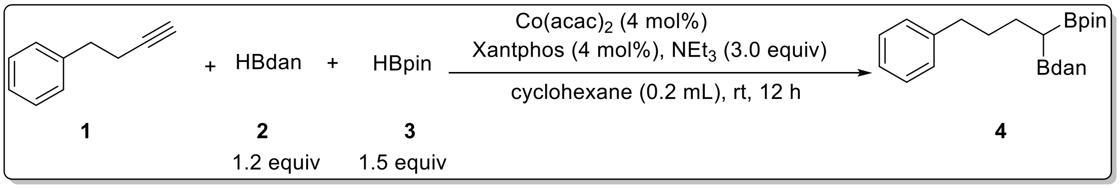

Supplementary Figure 1. Synthetic methods for gem-diborylalkanes

A tube was charged with $Co(acac)_2$ (4 mol%) and Xantphos (4 mol%) under N_2 atmosphere, then cyclohexane (0.2 mL), HBdan (0.24 mmol) and NEt_3 (0.6 mmol) were added subsequently. The reaction mixture was stirred at room temperature for 15 mins. Then HBpin (0.3 mmol) and **1** (0.2 mmol) were added subsequently at room temperature, then the resulting mixture was stirred at room temperature for 12 h. The residue was purified by column chromatography to afford the corresponding *gem*-diborylalkanes **4**.

General Procedure B:

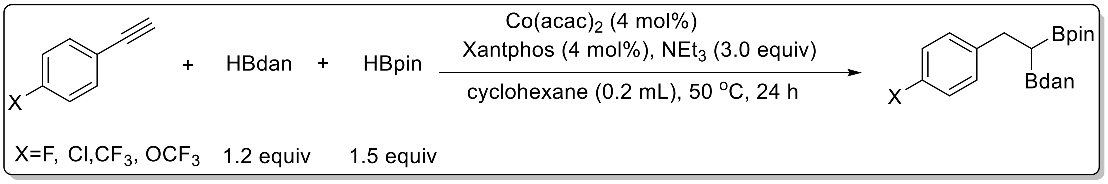

Supplementary Figure 2. Synthetic methods for gem-diborylalkanes

A tube was charged with $Co(acac)_2$ (4 mol%) and Xantphos (4 mol%) under N_2 atmosphere, then cyclohexane (0.2 mL), HBdan (0.24 mmol) and NEt_3 (0.6 mmol) were added subsequently. The reaction mixture was stirred at room temperature for 15 mins. Then HBpin (0.3 mmol) and Phenylacetylene containing halogen (0.2 mmol) were added subsequently at room temperature, then the resulting mixture was stirred at 50 ºC for 24 h. The residue was purified by column chromatography to afford the corresponding product *gem*-diborylalkanes.

General Procedure C:

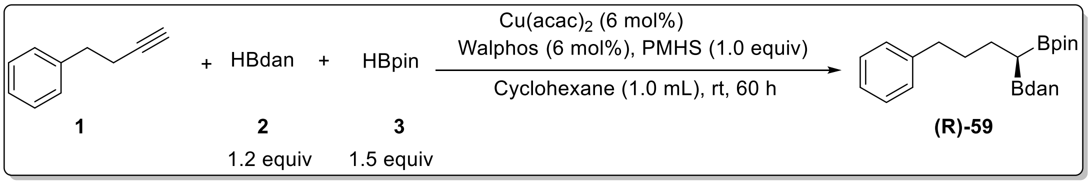

Supplementary Figure 3. Synthetic methods for chiral gem-diborylalkanes

A tube was charged with $Cu(acac)_2$ (6 mol%) and Walphos (6 mol%) under N_2 atmosphere, then cyclohexane (1.0 mL), HBdan (0.24 mmol) and PMHS (0.2 mmol) were added subsequently. The reaction mixture was stirred at room temperature for 10 mins. Then HBpin (0.3 mmol) and **1** (0.2 mmol) were added subsequently at room temperature, then the resulting mixture was stirred at ambient temperature for 60 h. The residue was purified by column chromatography to afford the corresponding product **(R)-59.**

General Procedure D:

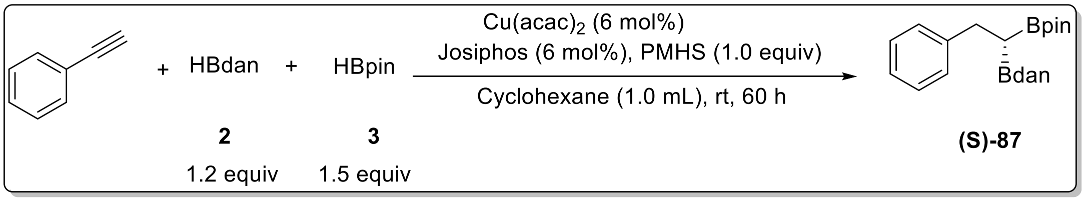

Supplementary Figure 4. Synthetic methods for chiral gem-Diborylalkanes

A tube was charged with $Cu(acac)_2$ (6 mol%) and Josiphos (6 mol%) under N_2 atmosphere, then cyclohexane (1.0 mL), HBdan (0.24 mmol) and PMHS (0.2 mmol) were added subsequently. The reaction mixture was stirred at room temperature for 10 mins. Then HBpin (0.3 mmol) and Phenylacetylene (0.2 mmol) were added subsequently at room temperature, then the resulting mixture was stirred at ambient temperature for 60 h. The residue was purified by column chromatography to afford the corresponding product **(S)-88.**

General Procedure E to Complex Alkynes:

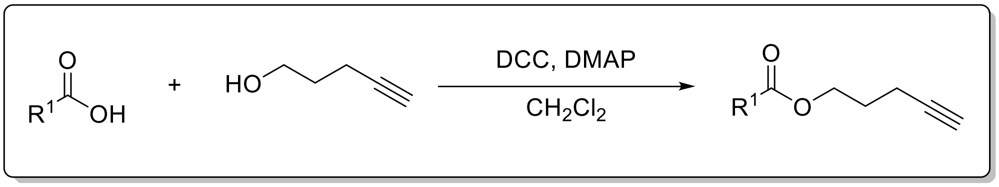

Supplementary Figure 5. Synthetic methods for Complex Alkynes

A tube was charged with 4-Pentyn-1-ol (3.6 mmol), acid (3.6 mmol), DCC (735 mg, 3.6 mmol) and DMAP (2 mg) in dry DCM (3 mL) and the solution was stirred for 6 h at room temperature under N_2 atmosphere. The reaction mixture was diluted with DCM (20 mL) and filtered. The residue was purified by column chromatography to afford the corresponding product.

3. Supplementary Notes

3.1 Control experiments:

1)

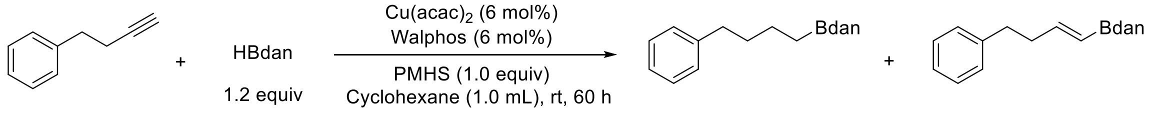

1 2 **92**, <10% yield **93**, trace

Supplementary Figure 6. Control experiments. 1 was exposed to HBdan in the absence of HBpin, under the standard reaction conditions.

A tube was charged with $Cu(acac)_2$ (6 mol%) and Walphos (6 mol%) under N_2 atmosphere, then cyclohexane (1.0 mL), HBdan (0.24 mmol), PMHS (1.0 equiv) and **1** (0.2 mmol) were added subsequently at room temperature, then the resulting mixture was stirred at ambient temperature for 60 h. The yields of products were determined with gas chromatography (GC) analysis with dodecane as internal standard.

2)

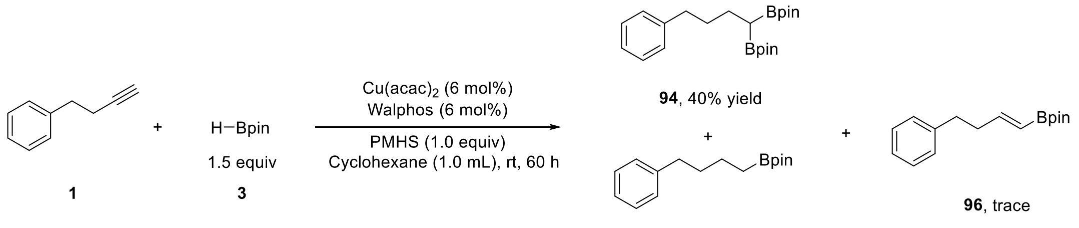

95, <10% yield

Supplementary Figure 7. Control experiments. 1 was exposed to HBpin in the absence of HBdan, under the standard reaction conditions.

A tube was charged with $Cu(acac)_2$ (6 mol%) and Walphos (4 mol%) under N_2 atmosphere, then cyclohexane (1.0 mL), HBpin (0.3 mmol), PMHS (1.0 equiv) and **1a** (0.2 mmol) were added subsequently at room temperature, then the resulting mixture was stirred at ambient temperature for 60 h. The yields of products were determined with gas chromatog- raphy (GC) analysis with Dodecane as internal standard. The yields of products were determined with gas chromatography (GC) analysis with dodecane as internal standard.

3)

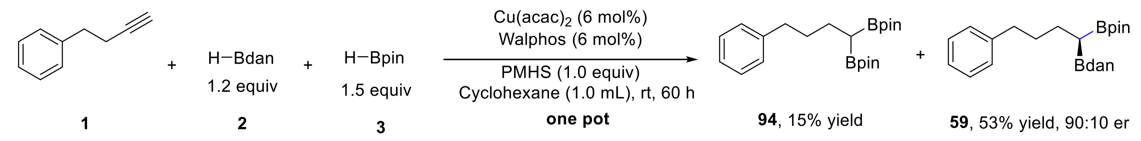

Supplementary Figure 8. Control experiments. One-pot reaction to the target product **59**.

A tube was charged with $Cu(acac)_2$ (6 mol%) and Walphos (6 mol%) under N_2 atmosphere, then cyclohexane (1.0 mL), HBdan (0.24 mmol), HBpin (0.3 mmol), PMHS (1.0 equiv) and **1** (0.2 mmol) were added subsequently at room temperature in one pot, then the resulting mixture was stirred at ambient temperature for 60 h. The residue was purified by column chromatography to afford the corresponding product**.**

4)

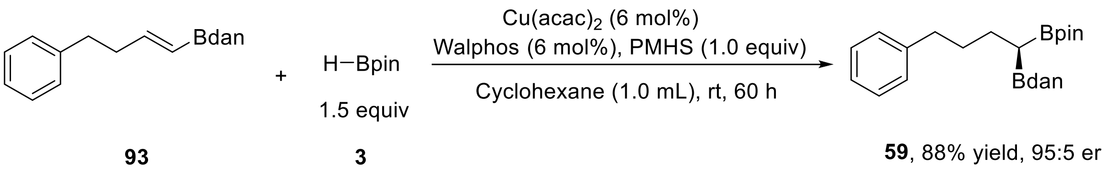

Supplementary Figure 9. Control experiments. alkenyl-Bdan **93** and HBpin was exposed under the standard asymmetric reaction conditions.

A tube was charged with $Cu(acac)_2$ (6 mol%) and Walphos (4 mol%) under N_2 atmosphere, then cyclohexane (1.0 mL), HBpin (0.3 mmol), PMHS (1.0 equiv) and **94** (0.2 mmol) were added subsequently at room temperature in one pot, then the resulting mixture was stirred at ambient temperature for 60 h. The residue was purified by column chromatography to afford the corresponding product **59**.

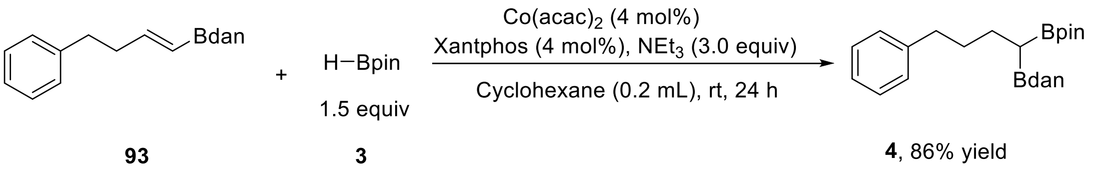

Supplementary Figure 10. Control experiments. alkenyl-Bdan **93** and HBpin was exposed under the standard racemic reaction conditions.

A tube was charged with $Cu(acac)_2$ (6 mol%) and Walphos (4 mol%) under N_2 atmosphere, then cyclohexane (1.0 mL), HBpin (0.3 mmol), PMHS (1.0 equiv) and **94** (0.2 mmol) were added subsequently at room temperature in one pot, then the resulting mixture was stirred at ambient temperature for 24 h. The residue was purified by column chromatography to afford the corresponding product **4**.

5)

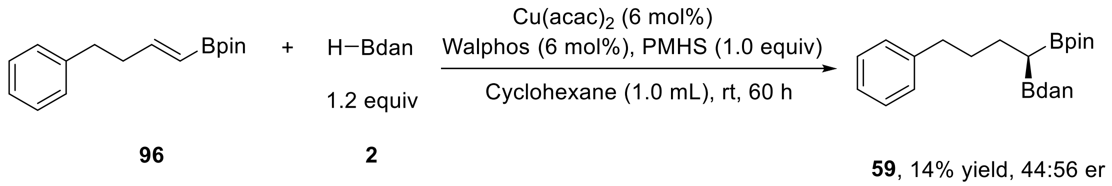

Supplementary Figure 11. Control experiments. alkenyl-Bpin **96** and HBdan was exposed under the standard asymmetric reaction conditions.

A tube was charged with $Cu(acac)_2$ (6 mol%) and Walphos (6 mol%) under N_2 atmosphere, then cyclohexane (1.0 mL), HBdan (0.24 mmol), PMHS (1.0 equiv) and **97** (0.2 mmol) were added subsequently at room temperature in one pot, then the resulting mixture was stirred at ambient temperature for 60 h. The residue was purified by column chromatography to afford the corresponding product**.**

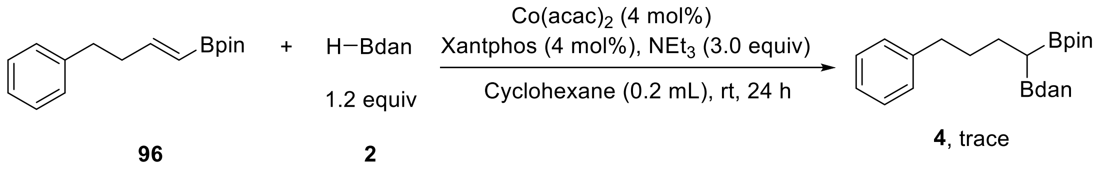

Supplementary Figure 12. Control experiments. alkenyl-Bpin **96** and HBdan was exposed under the standard racemic reaction conditions.

A tube was charged with $Co(acac)_2$ (6 mol%) and Xantphos (6 mol%) under N_2 atmosphere, then cyclohexane (1.0 mL), HBdan (0.24 mmol), NEt_3 (3.0 equiv) and **97** (0.2 mmol) were added subsequently at room temperature in one pot, then the resulting mixture was stirred at ambient temperature for 24 h. The crude mixture was detected by GC-MS.

6)

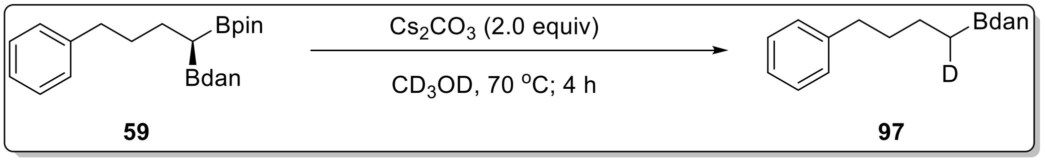

Supplementary Figure 13. Control experiments. deuterium experiment.

To a 25 mL Schlenk tube, was added gem-diborylalkanes **59** (0.2 mmol, 1.0 equiv) followed by 0.4 mmol of Cs_2CO_3 (2 equiv) under air. Then CD_3OD (0.5 mL) was added at rt and the reaction mixture was stirred at 70 °C for 4 h. When over, the reaction mixture was extracted with EA/H_2O. Subsequently, the organic layers

were dried with Na_2SO_4, and concentrated to dryness. The combined filtrates were concentrated and crude product was purified by flash column chromatography using PE/EtOAc = 100/1 as the eluent to give the corresponding product **97** as a colorless oil[1].

3.2. ^{31}P NMR Studies

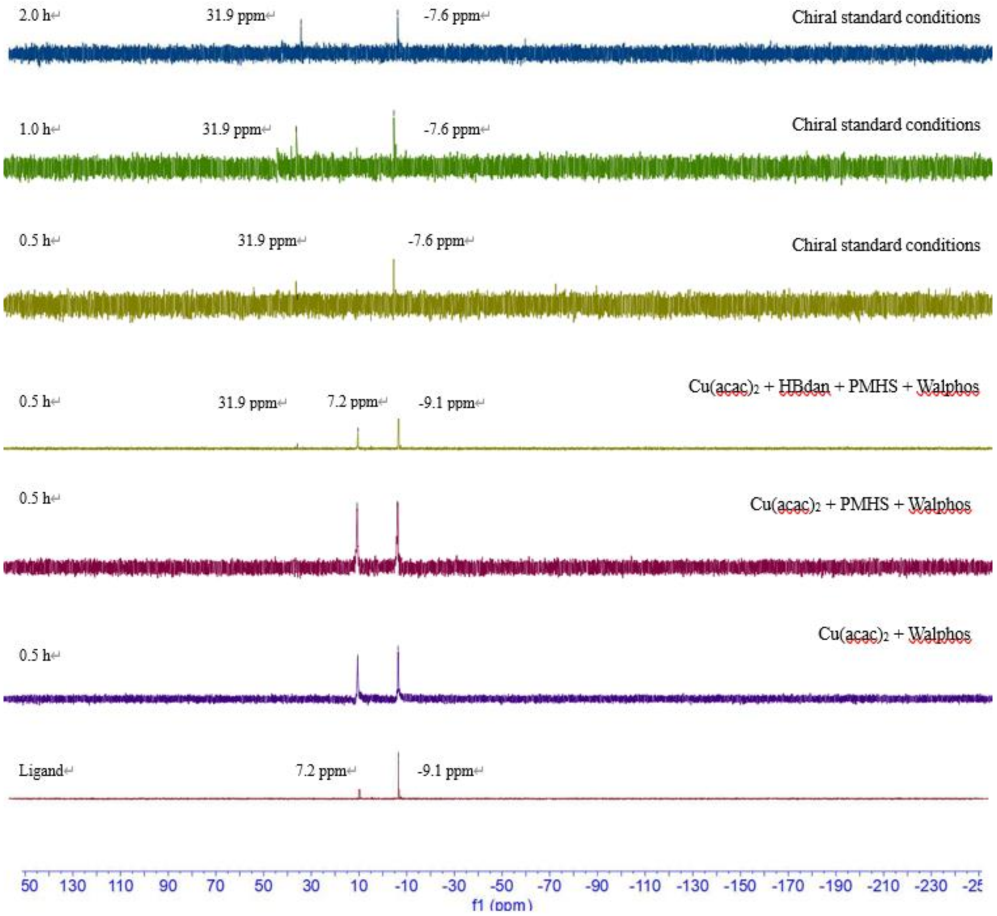

Supplementary Figure 14. ^{31}P NMR Studies. ^{31}P NMR spectra along with different reaction times.

We monitored the changes in yields over time, and we found that the yield of **4** gradually increased as the time went on during the reaction; on the other hand, the yields of alkenyl-Bdan **94** increased at the first 2 h and decreased gradually after that. These results validated the intermediacy of alkenyl-Bdan species and the first hydroboration should precede with HBdan and the second hydroboration should be with HBpin in this catalytic process. This is further supported by ^{31}P NMR analysis of the hydroboration cycle: When we use chiral standard conditions, free Walphos still exited after 5 mins, as time went by, the typical peaks of the

free Walphos disappeared while new peaks at δ = –7.46 ppm and δ = –31.9 ppm showed up, which suggested that the ligand started to function with copper salt to form a new complex in the solution. In order to understand the type of complex, we further carried out several control experiments by stepwise addition of copper salt as well as PMHS and HBdan into the solution of Walphos, we found that that the ligand started to function with copper salt to form a new complex in the solution.

3.3 Crystal Data of 4, 59 and 89[b]

Crystallographic data for compound **4** (CCDC-2039502) has been deposited with the Cambridge Crystallographic Data Centre, Copies of the data can be obtained, free of charge, on application to CCDC (Email: deposit@ccdc.cam.ac.uk).

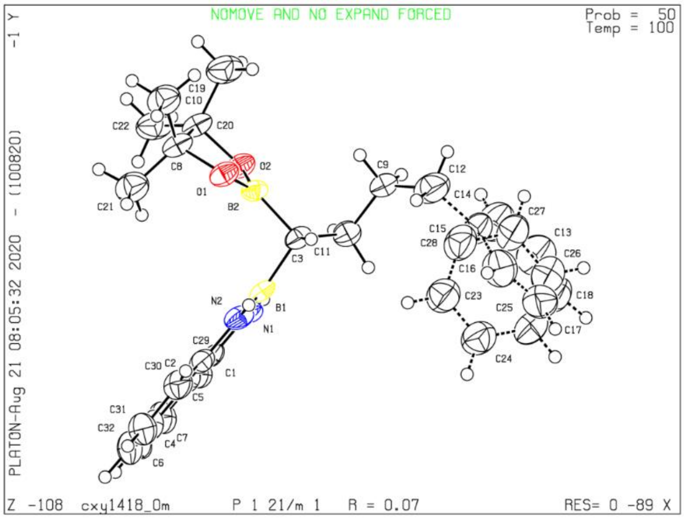

Supplementary Figure 15. Crystal structure of compound 4

```
Bond precision:  = 0.0000 A             Wavelength=1.54178

Cell:            a=10.0572(15)    b=10.3891(16)       c=12.520(2)
                 alpha=90         beta=105.820(17)    gamma=90
Temperature:     100 K

                 Calculated                Reported
Volume           1258.6(4)                 1258.6(4)
Space group      P 21/m                    P 1 21/m 1
Hall group       -P 2yb                    -P 2yb
Moiety formula   C26 H32 B2 N2 O2          C24.39 H30.66 B2 N2 O2,
                                           0.27(C6 H5)
Sum formula      C26 H32 B2 N2 O2          C26 H32 B2 N2 O2
Mr               426.16                    426.15
Dx,g cm-3        1.125                     1.124
Z                2                         2
Mu (mm-1)        0.540                     0.540
F000             456.0                     456.0
F000'            457.22
h,k,lmax         11,12,14                  11,12,14
Nref             2344                      2338
Tmin,Tmax        0.801,0.841               0.703,0.753
Tmin'            0.801

Correction method= # Reported T Limits: Tmin=0.703 Tmax=0.753
AbsCorr = MULTI-SCAN

Data completeness= 0.997          Theta(max)= 66.412

R(reflections)= 0.0695( 2059)     wR2(reflections)= 0.1801( 2338)

S = 1.055                 Npar= 298
```

Crystallographic data for compound **59** (CCDC-2107254) has been deposited with the Cambridge Crystallographic Data Centre, Copies of the data can be obtained, free of charge, on application to CCDC (Email: deposit@ccdc.cam.ac.uk).

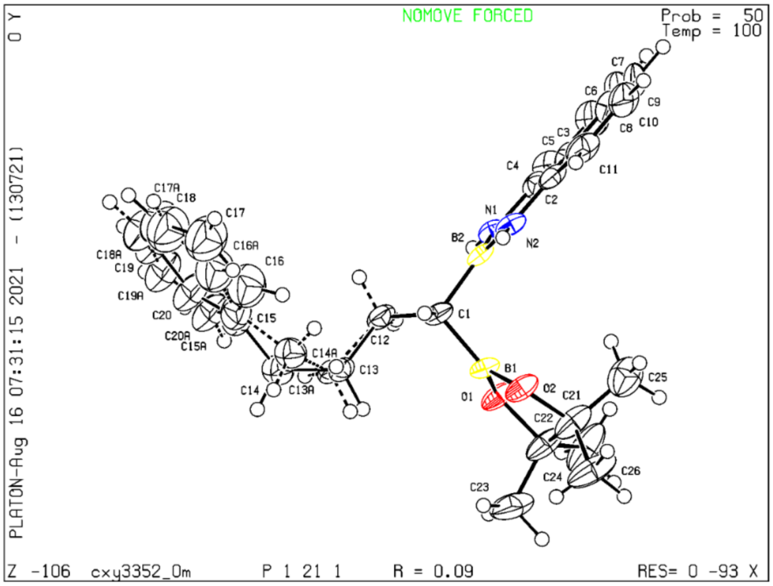

Supplementary Figure 16. Crystal structure of compound 59

Bond precision: C-C = 0.0086 A Wavelength=1.54178

Cell:	a=9.9834(5)	b=10.1836(5)	c=12.3364(6)
	alpha=90	beta=105.559(2)	gamma=90
Temperature:	100 K		

	Calculated	Reported
Volume	1208.24(10)	1208.24(10)
Space group	P 21	P 1 21 1
Hall group	P 2yb	P 2yb
Moiety formula	C26 H32 B2 N2 O2	C26 H32 B2 N2 O2
Sum formula	C26 H32 B2 N2 O2	C26 H32 B2 N2 O2
Mr	426.16	426.15
Dx,g cm-3	1.171	1.171
Z	2	2
Mu (mm-1)	0.562	0.562
F000	456.0	456.0
F000'	457.22	
h,k,lmax	12,12,14	12,12,14
Nref	4454[2362]	4404
Tmin,Tmax	0.856,0.889	0.673,0.753
Tmin'	0.840	

Correction method= # Reported T Limits: Tmin=0.673 Tmax=0.753
AbsCorr = MULTI-SCAN

Data completeness= 1.86/0.99 Theta(max)= 68.526

R(reflections)= 0.0902(4314) wR2(reflections)= 0.2909(4404)

S = 1.488 Npar= 306

Crystallographic data for compound **89**[b] (CCDC- 2118336) has been deposited with the Cambridge Crystallographic Data Centre, Copies of the data can be obtained, free of charge, on application to CCDC (Email: deposit@ccdc.cam.ac.uk).

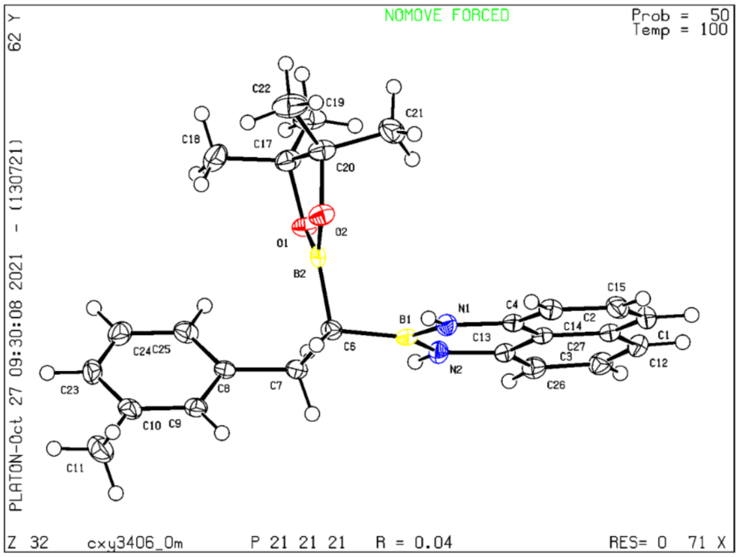

Supplementary Figure 17. Crystal structure of compound 89

```
Bond precision:   C-C = 0.0031 A          Wavelength=1.54178

Cell:              a=6.4367(3)     b=11.4196(5)     c=30.5689(15)
                   alpha=90        beta=90          gamma=90
Temperature:       100 K

                 Calculated                Reported
Volume           2246.95(18)               2246.95(18)
Space group      P 21 21 21                P 21 21 21
Hall group       P 2ac 2ab                 P 2ac 2ab
Moiety formula   C25 H30 B2 N2 O2          C25 H30 B2 N2 O2
Sum formula      C25 H30 B2 N2 O2          C25 H30 B2 N2 O2
Mr               412.13                    412.13
Dx,g cm-3        1.218                     1.218
Z                4                         4
Mu (mm-1)        0.588                     0.588
F000             880.0                     880.0
F000'            882.38
h,k,lmax         7,13,36                   7,13,36
Nref             4125[ 2400]               4120
Tmin,Tmax        0.881,0.910               0.872,1.000
Tmin'            0.863

Correction method= # Reported T Limits: Tmin=0.872 Tmax=1.000
AbsCorr = MULTI-SCAN

Data completeness= 1.72/1.00        Theta(max)= 68.383

                                                    wR2(reflections)=
R(reflections)= 0.0360( 3970)                       0.0890( 4120)

S = 1.083                  Npar= 285
```

3.4 Characterization Data

2-(4-phenyl-1-(4,4,5,5-tetramethyl-1,3,2-dioxaborolan-2-yl)butyl)-2,3-dihydro-1H-naphtho[1,8-de][1,3,2]diazaborinine (4)

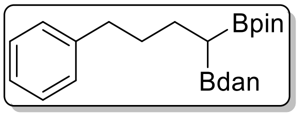

4

The reaction was performed following the general procedure A. The residue was purified by flash column chromatograph to give the product as a crystalline solid (64.7 mg, 76%). **^{1}H NMR** (500 MHz, $CDCl_3$) δ 7.31 – 7.27 (m, 2H), 7.21 – 7.18 (m, 3H), 7.11 – 7.07 (m, 2H), 6.99 (d, J = 8.3 Hz, 2H), 6.27 (d, J = 7.3 Hz, 2H), 5.76 (s, 2H), 2.65 (t, J = 6.9 Hz, 2H), 1.71 – 1.66 (m, 2H), 1.62 – 1.56 (m, 2H), 1.25 (d, J = 6.0 Hz, 12H), 0.78 (dd, J = 8.2, 6.0 Hz, 1H). **^{13}C NMR** (126 MHz, $CDCl_3$) δ 142.6, 141.3, 136.3, 128.4, 128.3, 127.56, 125.7 119.5, 117.3, 105.5, 83.2, 36.0, 34.0, 26.0, 25.1, 24.5. **^{11}B NMR** (160 MHz, $CDCl_3$) δ 34.18, 33.79. **HRMS** (ESI, m/z) calcd for $C_{26}H_{33}B_2N_2O_3$ [M+H] $^+$: 427.2723; found: 427.2726.

2-(3-phenyl-1-(4,4,5,5-tetramethyl-1,3,2-dioxaborolan-2-yl)propyl)-2,3-dihydro-1H-naphtho[1,8-de][1,3,2]diazaborinine (5)

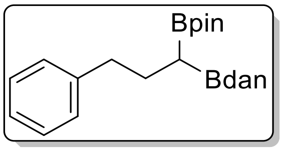

5

The reaction was performed following the general procedure A. The residue was purified by flash column chromatograph to give the product as a crystalline solid (51.9 mg, 63%). **^{1}H NMR** (500 MHz, $CDCl_3$) δ 7.32 – 7.27 (m, 2H), 7.21 (dd, J = 13.2, 7.0 Hz, 3H), 7.10 (t, J = 7.8 Hz, 2H), 7.00 (d, J = 8.2 Hz, 2H), 6.29 (d, J = 7.3 Hz, 2H), 5.80 (s, 2H), 2.78 – 2.70 (m, 1H), 2.64 – 2.60 (m, 1H), 2.03 – 1.94 (m, 1H), 1.88 – 1.83 (m, 1H), 1.28 (s, 6H), 1.27 (s, 6H), 0.84 (dd, J = 9.8, 5.6 Hz, 1H). **^{13}C NMR** (126 MHz, $CDCl_3$) δ 142.6, 141.3, 136.3, 128.5, 128.33 (s), 127.6, 125.8, 119.5, 117.3, 105.5, 83.3, 38.5, 28.6, 25.1, 24.5. **^{11}B NMR** (160 MHz, $CDCl_3$) δ 34.23, 34.02. **HRMS** (ESI, m/z) calcd for $C_{25}H_{31}B_2N_2O_2$ [M+H] $^+$: 413.2566; found: 413.2561.

2-(2-cyclopropyl-1-(4,4,5,5-tetramethyl-1,3,2-dioxaborolan-2-yl)ethyl)-2,3-dihydro-1H-naphtho[1,8-de][1,3,2]diazaborinine (6)

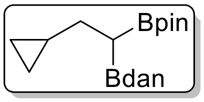

6

The reaction was performed following the general procedure A. The residue was purified by flash column chromatograph to give the product as a crystalline solid (33.3 mg, 46%). **^{1}H NMR** (500 MHz, $CDCl_3$) δ 7.08 (t, J = 7.8 Hz, 2H), 6.99 (d, J = 8.2 Hz, 2H), 6.28 (d, J = 7.3 Hz, 2H), 5.80 (s, 2H), 1.57 – 1.52 (m, 1H), 1.47 – 1.43 (m, 1H), 1.25 (d, J = 4.6 Hz, 12H), 0.90 (dd, J = 9.5, 6.1 Hz, 1H), 0.76 (dt, J = 12.3, 5.1 Hz, 1H), 0.41 (d, J = 7.9 Hz, 2H), 0.13 (dd, J = 8.8, 3.9 Hz, 1H), 0.07 – 0.04 (m, 1H). **^{13}C NMR** (126 MHz, $CDCl_3$) δ 141.3,

136.3, 127.5, 119.5, 117.2, 105.4, 83.2, 31.6, 25.0, 24.8, 24.6, 13.4, 5.1, 4.8. **^{11}B NMR** (160 MHz, $CDCl_3$) δ 34.58, 34.03. **HRMS** (ESI, m/z) calcd for $C_{21}H_{29}B_2N_2O_2$ [M+H] $^+$: 363.2410; found: 363.2401.

2-(3-cyclohexyl-1-(4,4,5,5-tetramethyl-1,3,2-dioxaborolan-2-yl)propyl)-2,3-dihydro-1H-naphtho[1,8-de][1,3,2]diazaborinine (7)

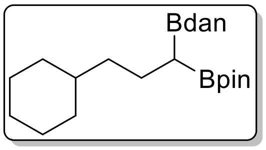

7

The reaction was performed following the general procedure A. The residue was purified by flash column chromatograph to give the product as a crystalline solid (51.0 mg, 51%). **^{1}H NMR** (500 MHz, $CDCl_3$) δ 7.12 – 7.07 (m, 2H), 7.00 (d, J = 8.2 Hz, 2H), 6.29 (d, J = 7.2 Hz, 2H), 5.80 (s, 2H), 1.75 – 1.63 (m, 8H), 1.56 (d, J = 4.3 Hz, 2H), 1.24 (s, 12H), 1.18 (dd, J = 18.8, 6.8 Hz, 3H), 0.89 (d, J = 11.3 Hz, 2H), 0.73 – 0.69 (m, 1H). **^{13}C NMR** (126 MHz, $CDCl_3$) δ 141.4, 136.3, 127.5, 119.5, 117.2, 105.4, 83.1, 40.3, 37.8, 33.5, 33.4, 33.3, 26.8, 26.4, 25.1, 24.9, 24.5, 24.5, 23.7. **^{11}B NMR** (160 MHz, $CDCl_3$) δ 35.43, 34.63. **HRMS** (ESI, m/z) calcd for $C_{25}H_{37}B_2N_2O_2$ [M+H] $^+$: 419.3036; found: 419.3032.

2-(2-cyclopentyl-1-(4,4,5,5-tetramethyl-1,3,2-dioxaborolan-2-yl)ethyl)-2,3-dihydro-1H-naphtho[1,8-de][1,3,2]diazaborinine (8)

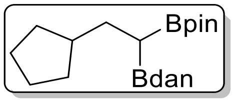

8

The reaction was performed following the general procedure A. The residue was purified by flash column chromatograph to give the product as a crystalline solid (45.3 mg, 58%). **^{1}H NMR** (500 MHz, $CDCl_3$) δ 7.09 (t, J = 7.8 Hz, 2H), 6.99 (d, J = 8.2 Hz, 2H), 6.29 (d, J = 7.3 Hz, 2H), 5.78 (s, 2H), 1.79 – 1.69 (m, 4H), 1.61 – 1.56 (m, 3H), 1.52 – 1.48 (m, 2H), 1.24 (d, J = 5.7 Hz, 12H), 1.09 (dt, J = 10.7, 3.3 Hz, 2H), 0.83 (dd, J = 9.1, 6.2 Hz, 1H). **^{13}C NMR** (126 MHz, $CDCl_3$) δ 141.4, 136.3, 127.5, 119.5, 117.2, 105.4, 83.2, 82.9, 42.4, 32.8, 32.6, 32.52, 32.50, 25.21, 25.18, 25.0, 24.8, 24.60, 24.57. **^{11}B NMR** (160 MHz, $CDCl_3$) δ 34.38, 33.90. **HRMS** (ESI, m/z) calcd for $C_{23}H_{32}B_2N_2NaO_2$ [M+Na] $^+$: 413.2542; found: 413.2534.

2-(1-(4,4,5,5-tetramethyl-1,3,2-dioxaborolan-2-yl)pentyl)-2,3-dihydro-1H-naphtho[1,8-de][1,3,2]diazaborinine (9)

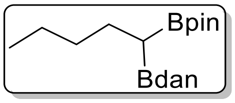

9

The reaction was performed following the general procedure A. The residue was purified by flash column chromatograph to give the product as a crystalline solid (52.5 mg, 72%). **^{1}H NMR** (500 MHz, $CDCl_3$) δ 7.09 (t, J = 7.8 Hz, 2H), 6.99 (d, J = 8.2 Hz, 2H), 6.29 (d, J = 7.3 Hz, 2H), 5.80 (s, 2H), 1.67 – 1.59 (m, 1H), 1.54

(dd, J = 13.9, 7.4 Hz, 1H), 1.37 – 1.31 (m, 4H), 1.25 (d, J = 6.1 Hz, 12H), 0.91 (t, J = 6.9 Hz, 3H), 0.75 (dd, J = 9.3, 6.3 Hz, 1H). **^{13}C NMR** (126 MHz, $CDCl_3$) δ 141.4, 136.3, 127.5, 119.5, 117.2, 105.4, 83.1, 34.9, 34.6, 26.1, 25.0, 24.9, 24.5, 24.5, 22.8, 14.1. **^{11}B NMR** (160 MHz, $CDCl_3$) δ 34.66, 34.34. **HRMS** (ESI, m/z) calcd for $C_{21}H_{31}B_2N_2O_2$ [M+H] $^+$: 365.2566; found: 365.2571.

2-(1-(4,4,5,5-tetramethyl-1,3,2-dioxaborolan-2-yl)heptyl)-2,3-dihydro-1H-naphtho[1,8-de][1,3,2]diazaborinine (10)

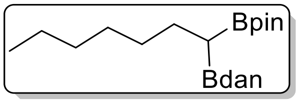

10

The reaction was performed following the general procedure A. The residue was purified by flash column chromatograph to give the product as a crystalline solid (61.2 mg, 78%). **^{1}H NMR** (500 MHz, $CDCl_3$) δ 7.10 – 7.07 (m, 2H), 6.98 (d, J = 7.3 Hz, 2H), 6.28 (d, J = 7.3 Hz, 2H), 5.78 (s, 2H), 1.54 (d, J = 7.3 Hz, 2H), 1.24 (s, 8H), 1.23 (s, 6H), 1.22 (s, 6H), 0.87 (d, J = 3.2 Hz, 3H), 0.74 – 0.72 (m, 1H). **^{13}C NMR** (126 MHz, $CDCl_3$) δ 141.4, 136.3, 127.5, 119.5, 117.2, 105.4, 83.1, 82.9, 32.6, 32.4, 31.8, 29.4, 29.3, 26.4, 25.7, 25.0, 24.9, 24.53, 24.50, 22.7, 22.6, 14.1. **^{11}B NMR** (160 MHz, $CDCl_3$) δ 34.88, 34.11. **HRMS** (ESI, m/z) calcd for $C_{23}H_{35}B_2N_2O_2$ [M+H] $^+$: 393.2879; found: 393.2874.

2-(1-(4,4,5,5-tetramethyl-1,3,2-dioxaborolan-2-yl)octyl)-2,3-dihydro-1H-naphtho[1,8-de][1,3,2]diazaborinine (11)

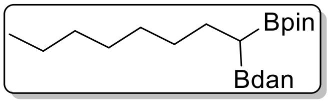

11

The reaction was performed following the general procedure A. The residue was purified by flash column chromatograph to give the product as a crystalline solid (61.8 mg, 76%). **^{1}H NMR** (500 MHz, $CDCl_3$) δ 7.10 – 7.06 (m, 2H), 6.98 (d, J = 8.2 Hz, 2H), 6.28 (d, J = 7.3 Hz, 2H), 5.79 (s, 2H), 1.57 – 1.41 (m, 2H), 1.27 (dd, J = 7.6, 2.5 Hz, 10H), 1.25 (s, 6H), 1.24 (s, 6H), 0.88 (t, J = 6.8 Hz, 3H), 0.75 – 0.72 (m, 1H). **^{13}C NMR** (126 MHz, $CDCl_3$) δ 141.4, 136.3, 127.5, 119.5, 117.2, 105.4, 83.1, 32.4, 31.9, 29.7, 29.2, 26.4, 25.0, 24.9, 24.5, 22.7, 14.1. **^{11}B NMR** (160 MHz, $CDCl_3$) δ 34.23, 33.23. **HRMS** (ESI, m/z) calcd for $C_{24}H_{36}B_2N_2NaO_2$ [M+Na] $^+$: 429.2855; found: 429.2859.

2-(1-(4,4,5,5-tetramethyl-1,3,2-dioxaborolan-2-yl)octyl)-2,3-dihydro-1H-naphtho[1,8-de][1,3,2]diazaborinine (12)

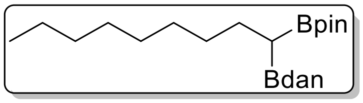

12

The reaction was performed following the general procedure A. The residue was purified by flash column chromatograph to give the product as a crystalline solid (57.2 mg, 68%). **^{1}H NMR** (500 MHz, $CDCl_3$) δ 7.10 (t, J = 7.8 Hz, 2H), 7.00 (d, J = 8.2 Hz, 2H), 6.30 (d, J = 7.3 Hz, 2H), 5.81 (s, 2H), 1.67 – 1.61 (m, 1H), 1.55

– 1.50 (m, 1H), 1.34 – 1.27 (m, 12H), 1.26 (d, *J* = 6.2 Hz, 12H), 0.90 (t, *J* = 6.8 Hz, 3H), 0.76 (dd, *J* = 9.4, 6.0 Hz, 1H). **^{13}C NMR** (126 MHz, $CDCl_3$) δ 141.4, 136.3, 127.6, 119.5, 117.2, 105.4, 83.2, 32.4, 31.9, 29.7, 29.5, 29.3, 26.4, 25.0, 24.5, 22.7, 14.1. **^{11}B NMR** (160 MHz, $CDCl_3$) δ 34.45, 34.19. **HRMS** (ESI, m/z) calcd for $C_{25}H_{39}B_2N_2O_2$ [M+H]$^+$: 421.3192; found: 421.3195.

2-(5-methyl-1-(4,4,5,5-tetramethyl-1,3,2-dioxaborolan-2-yl)hexyl)-2,3-dihydro-1H-naphtho[1,8-de][1,3,2]diazaborinine (13)

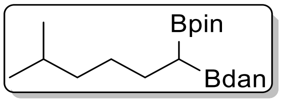

13

The reaction was performed following the general procedure A. The residue was purified by flash column chromatograph to give the product as a crystalline solid (48.6 mg, 62%). **^{1}H NMR** (500 MHz, $CDCl_3$) δ 7.09 (t, *J* = 7.8 Hz, 2H), 6.99 (d, *J* = 8.2 Hz, 2H), 6.29 (d, *J* = 7.2 Hz, 2H), 5.80 (s, 2H), 1.64 – 1.60 (m, 1H), 1.53 (td, *J* = 8.1, 7.4, 4.5 Hz, 2H), 1.37 (qd, *J* = 8.3, 5.5 Hz, 4H), 1.26 (s, 3H), 1.25 (s, 5H), 1.24 (s, 4H), 1.23 (s, 3H), 0.88 (d, *J* = 6.6 Hz, 3H), 0.85 (d, *J* = 6.5 Hz, 3H), 0.76 (dt, *J* = 10.1, 5.2 Hz, 1H). **^{13}C NMR** (126 MHz, $CDCl_3$) δ 141.4, 136.3, 127.5, 119.5, 117.2, 105.4, 83.1, 39.1, 30.1, 27.8, 26.6, 25.0, 24.9, 24.5, 24.5, 22.7, 22.6. **^{11}B NMR** (160 MHz, $CDCl_3$) δ 34.33, 33.33. **HRMS** (ESI, m/z) calcd for $C_{23}H_{34}B_2N_2NaO_2$ [M+H]$^+$: 415.2699; found: 415.2699.

2-(3,3-dimethyl-1-(4,4,5,5-tetramethyl-1,3,2-dioxaborolan-2-yl)butyl)-2,3-dihydro-1H-naphtho[1,8-de][1,3,2]diazaborinine (14)

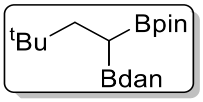

14

The reaction was performed following the general procedure A. The residue was purified by flash column chromatograph to give the product as a crystalline solid (42.4 mg, 56%). **^{1}H NMR** (500 MHz, $CDCl_3$) δ 7.11 – 7.06 (m, 2H), 7.00 (dd, *J* = 8.3, 1.8 Hz, 2H), 6.26 (d, *J* = 7.2 Hz, 2H), 5.60 (d, *J* = 18.6 Hz, 2H), 1.35 – 1.27 (m, 12H), 1.26 (s, 2H), 1.24 – 1.17 (m, 9H), 0.91 – 0.86 (m, 1H). **^{13}C NMR** (126 MHz, $CDCl_3$) δ 141.3, 127.5, 117.2, 105.4, 83.2, 82.9, 40.3, 39.23 31.8, 31.6, 29.3, 29.2, 24.9, 24.8, 24.7, 24.6. **^{11}B NMR** (160 MHz, $CDCl_3$) δ 34.35, 34.06. **HRMS** (ESI, m/z) calcd for $C_{22}H_{33}B_2N_2O_2$ [M+H]$^+$: 379.2723; found: 379.2723.

2-(6-((tert-butyldimethylsilyl)oxy)-1-(4,4,5,5-tetramethyl-1,3,2-dioxaborolan-2-yl)hexyl)-2,3-dihydro-1H-naphtho[1,8-de][1,3,2]diazaborinine (15)

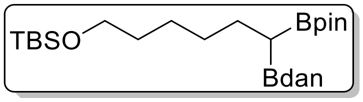

15

The reaction was performed following the general procedure A. The residue was purified by flash column chromatograph to give the product as a crystalline solid (64.1 mg, 63%). **^{1}H NMR** (500 MHz, $CDCl_3$) δ 7.08 (t, *J* = 7.8 Hz, 2H), 6.98 (d, *J* = 8.2 Hz, 2H), 6.28 (d, *J* = 7.3 Hz, 2H), 5.79 (s, 2H), 3.59 (d, *J* = 6.6 Hz, 2H),

1.55 – 1.48 (m, 4H), 1.35 (dd, J = 12.2, 6.6 Hz, 4H), 1.25 (s, 6H), 1.23 (s, 6H), 0.89 (s, 9H), 0.75 – 0.71 (m, 1H), 0.04 (s, 6H). **^{13}C NMR** (126 MHz, $CDCl_3$) δ 141.3, 136.3, 127.5, 119.5, 117.2, 105.4, 83.2, 63.3, 32.8, 32.2, 26.4, 26.0, 25.9, 25.0, 24.9, 24.5, 24.52,24.50, 18.4, -5.2. **^{11}B NMR** (160 MHz, $CDCl_3$) δ 33.92, 33.51. **HRMS** (ESI, m/z) calcd for $C_{28}H_{47}B_2N_2O_3Si$ [M+H] $^+$: 509.3537; found: 509.3530.

2-(4-((tert-butyldimethylsilyl)oxy)-1-(4,4,5,5-tetramethyl-1,3,2-dioxaborolan-2-yl)butyl)-2,3-dihydro-1H-naphtho[1,8-de][1,3,2]diazaborinine (16)

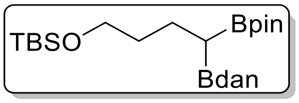

16

The reaction was performed following the general procedure A. The residue was purified by flash column chromatograph to give the product as a crystalline solid (50.0 mg, 52%) **^{1}H NMR (**500 MHz, $CDCl_3$) δ 7.11 (dd, J = 8.2, 7.3 Hz, 2H), 7.01 (dd, J = 8.3, 1.0 Hz, 2H), 6.30 (dd, J = 7.4, 1.0 Hz, 2H), 5.86 (s, 2H), 3.68 – 3.64 (m, 2H), 1.66 (dd, J = 13.6, 5.3 Hz, 2H), 1.63 – 1.56 (m, 2H), 1.26 (d, J = 5.3 Hz, 12H), 0.94 (s, 9H), 0.78 (dd, J = 8.7, 5.6 Hz, 1H), 0.09 (d, J = 1.0 Hz, 6H). **^{13}C NMR** (126 MHz, $CDCl_3$) δ 141.3, 136.3, 127.5, 119.5, 117.2, 105.4, 83.2, 82.9, 63.2, 35.4, 25.1, 24.9, 24.54, 24.52, 22.4, 18.4, -5.2. **^{11}B NMR** (160 MHz, $CDCl_3$) δ 34.08, 33.91. **HRMS** (ESI, m/z) calcd for $C_{26}H_{43}B_2N_2O_3Si$ [M+H] $^+$: 481.3224; found: 481.3221.

tert-butyl 4-(2-(1H-naphtho[1,8-de][1,3,2]diazaborinin-2(3H)-yl)-2-(4,4,5,5-tetramethyl-1,3,2-dioxaborolan-2-yl)ethyl)piperidine-1-carboxylate (17)

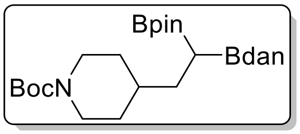

17

The reaction was performed following the general procedure A. The residue was purified by flash column chromatograph to give the product as a crystalline solid (58.6 mg, 58%). **^{1}H NMR** (500 MHz, $CDCl_3$) δ 7.13 – 7.07 (m, 2H), 7.04 – 6.96 (m, 2H), 6.33 – 6.27 (m, 2H), 5.76 (s, 2H), 4.08 (s, 4H), 2.65 (s, 4H), 1.45 (d, J = 3.5 Hz, 9H), 1.24 (s, 12H), 1.06 (d, J = 12.2 Hz, 2H), 0.85 (s, 1H), 0.81 – 0.71 (m, 1H). **^{13}C NMR** (126 MHz, $CDCl_3$) δ 155.2, 141.2, 136.3, 117.4, 105.5, 83.3, 79.3, 38.0, 33.0, 28.5, 25.0, 24.8, 24.5. **^{11}B NMR** (160 MHz, $CDCl_3$) δ 33.92, 33.33. **HRMS** (ESI, m/z) calcd for $C_{21}H_{31}B_2N_2O_2$ [M+H] $^+$: 365.2566; found: 365.2571.

2-(2-(cyclohex-1-en-1-yl)-1-(4,4,5,5-tetramethyl-1,3,2-dioxaborolan-2-yl)ethyl)-2,3-dihydro-1H-naphtho[1,8-de][1,3,2]diazaborinine (18)

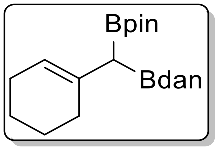

18

The reaction was performed following the general procedure A. The residue was purified by flash column chromatograph to give the product as a crystalline solid (51.5 mg, 64%). **^{1}H NMR** (500 MHz, $CDCl_3$) δ 7.10 – 7.08 (m, 2H), 6.98 (d, *J* = 4.8 Hz, 2H), 6.31 (d, *J* = 8.2 Hz, 2H), 6.05 (s, 2H), 6.00 (s, 1H), 2.26 (s, 1H), 2.19 (s, 1H), 1.67 (t, *J* = 6.1 Hz, 2H), 1.62 – 1.60 (m, 2H), 1.36 (s, 12H), 1.24 (d, *J* = 4.6 Hz, 4H), 0.90 – 0.80 (m, 1H). **^{13}C NMR** (126 MHz, $CDCl_3$) δ 153.6, 136.4, 134.3, 127.6, 117.2, 105.6, 83.8, 26.5, 26.2, 25.0, 24.8, 24.60, 22.4, 22.1. **^{11}B NMR** (160 MHz, $CDCl_3$) δ 32.80, 31.92. **HRMS** (ESI, m/z) calcd for $C_{24}H_{33}B_2N_2O_2$ [M+H] $^+$: 403.2723; found: 403.2725.

5-(1H-naphtho[1,8-de][1,3,2]diazaborinin-2(3H)-yl)-5-(4,4,5,5-tetramethyl-1,3,2-dioxaborolan-2-yl)pentyl propionate (19)

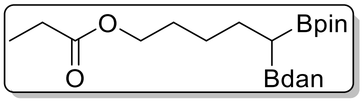

19

The reaction was performed following the general procedure A. The residue was purified by flash column chromatograph to give the product as a crystalline solid (53.2 mg, 61%). **^{1}H NMR** (500 MHz, $CDCl_3$) δ 7.11 – 7.06 (m, 2H), 6.99 (d, *J* = 8.2 Hz, 2H), 6.29 (d, *J* = 7.3 Hz, 2H), 5.79 (s, 2H), 4.06 (dt, *J* = 9.5, 5.2 Hz, 2H), 2.33 – 2.31 (m, 1H), 2.31 – 2.28 (m, 1H), 1.61 – 1.51 (m, 2H), 1.48 – 1.37 (m, 2H), 1.36 – 1.26 (m, 2H), 1.25 (s, 6H), 1.23 (s, 6H), 1.12 (t, *J* = 7.6 Hz, 3H), 0.75 (dd, *J* = 9.3, 6.1 Hz, 1H). **^{13}C NMR** (126 MHz, $CDCl_3$) δ 174.6, 141.3, 136.3, 127.5, 119.5, 117.3, 105.5, 83.2, 64.5, 64.3, 28.6, 28.55, 27.6, 26.0, 25.0, 24.9, 24.5, 9.2. **^{11}B NMR** (160 MHz, $CDCl_3$) δ 34.34, 34.09. **HRMS** (ESI, m/z) calcd for $C_{24}H_{35}B_2N_2O_4$ [M+H] $^+$: 437.2777; found: 437.2774.

2-(4-((tetrahydro-2H-pyran-2-yl)oxy)-1-(4,4,5,5-tetramethyl-1,3,2-dioxaborolan-2-yl)butyl)-2,3-dihydro-1H-naphtho[1,8-de][1,3,2]diazaborinine (20)

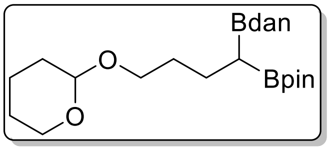

20

The reaction was performed following the general procedure A. The residue was purified by flash column chromatograph to give the product as a crystalline solid (54.0 mg, 60%). **^{1}H NMR** (500 MHz, $CDCl_3$) δ 7.09 (t, *J* = 7.8 Hz, 2H), 6.99 (d, *J* = 8.2 Hz, 2H), 6.29 (dd, *J* = 7.3, 1.0 Hz, 2H), 5.87 (d, *J* = 3.9 Hz, 2H), 4.59 (td, *J* = 4.5, 2.9 Hz, 1H), 3.91 – 3.86 (m, 1H), 3.81 – 3.75 (m, 1H), 3.53 – 3.49 (m, 1H), 3.43 (dd, *J* = 9.7, 4.8 Hz, 1H), 1.88 – 1.83 (m, 1H), 1.77 – 1.59 (m, 7H), 1.54 (d, *J* = 6.3 Hz, 2H), 1.25 (d, *J* = 5.0 Hz, 12H), 0.80 – 0.76 (m, 1H). **^{13}C NMR** (126 MHz, CDCl3) δ 141.4, 136.3, 127.5, 119.5, 117.2, 105.4, 99.0, 83.2, 67.57, 67.55, 62.49, 62.47, 32.2, 32.1, 30.8, 25.5, 25.0, 24.9, 24.5, 22.9, 22.8, 19.81, 19.78. **^{11}B NMR** (160 MHz, $CDCl_3$) δ 33.89, 33.50. **HRMS** (ESI, m/z) calcd for $C_{25}H_{37}B_2N_2O_4$ [M+H] +: 451.2934; found: 451.2937.

2-(3,3-diethoxy-1-(4,4,5,5-tetramethyl-1,3,2-dioxaborolan-2-yl)propyl)-2,3-dihydro-1H-naphtho[1,8-de][1,3,2]diazaborinine (21)

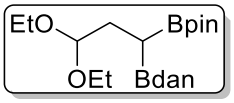

21

The reaction was performed following the general procedure A. The residue was purified by flash column chromatograph to give the product as a crystalline solid (44.1 mg, 52%). **^{1}H NMR** (500 MHz, $CDCl_3$) δ 7.10 – 7.06 (m, 2H), 6.98 (d, *J* = 8.3 Hz, 2H), 6.29 – 6.25 (m, 2H), 5.92 (d, *J* = 31.5 Hz, 2H), 3.57 – 3.36 (m, 2H), 1.97 – 1.87 (m, 0H), 1.82 (ddt, *J* = 13.4, 9.3, 6.6 Hz, 0H), 1.25 (d, *J* = 4.0 Hz, 6H), 1.21 (dt, *J* = 7.0, 3.6 Hz, 3H), 0.89 – 0.84 (m, 1H). **^{13}C NMR** (126 MHz, $CDCl_3$) δ 141.37, 141.35, 136.3, 127.5, 117.2, 105.4, 104.2, 61.6, 61.4, 30.2, 26.4, 25.07, 25.03, 24.54, 24.52, 15.40, 15.37. **^{11}B NMR** (160 MHz, $CDCl_3$) δ 33.51, 32.45. **HRMS** (ESI, m/z) calcd for $C_{23}H_{34}B_2N_2NaO_4$ [M+Na] $^+$: 447.2597; found: 447.2593.

5-(1H-naphtho[1,8-de][1,3,2]diazaborinin-2(3H)-yl)-5-(4,4,5,5-tetramethyl-1,3,2-dioxaborolan-2-yl)pentyl (2E,4E)-hexa-2,4-dienoate (22)

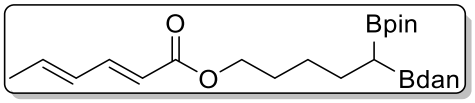

22

The reaction was performed following the general procedure A. The residue was purified by flash column chromatograph to give the product as a crystalline solid (45.5 mg, 48%). **^{1}H NMR** (500 MHz, $CDCl_3$) δ 7.08 (dd, *J* = 8.3, 7.3 Hz, 2H), 6.99 (dd, *J* = 8.3, 1.0 Hz, 2H), 6.29 (dd, *J* = 7.3, 1.0 Hz, 2H), 5.79 (s, 2H), 5.60 – 5.27 (m, 4H), 4.07 (dt, *J* = 6.6, 3.4 Hz, 2H), 2.35 – 2.32 (m, 2H), 2.30 – 2.27 (m, 2H), 1.63 (d, *J* = 1.2 Hz, 3H), 1.45 – 1.36 (m, 2H), 1.26 (s, 3H), 1.25 (s, 6H), 1.23 (s, 3H), 0.74 (dd, *J* = 9.3, 6.2 Hz, 1H). **^{13}C NMR** (126 MHz, $CDCl_3$) δ 173.4, 141.3, 136.3, 129.2, 127.6, 126.1, 117.3, 105.5, 83.2, 64.3, 34.4, 28.8, 28.6, 27.9, 26.0, 25.0, 24.8, 24.5, 17.9. **^{11}B NMR** (160 MHz, $CDCl_3$) δ 34.45, 33.68. **HRMS** (ESI, m/z) calcd for $C_{27}H_{37}B_2N_2O_4$ [M+H] $^+$: 475.2934; found: 475.2929.

5-(1H-naphtho[1,8-de][1,3,2]diazaborinin-2(3H)-yl)-5-(4,4,5,5-tetramethyl-1,3,2-dioxaborolan-2-yl)pentyl 2-propylpentanoate (23)

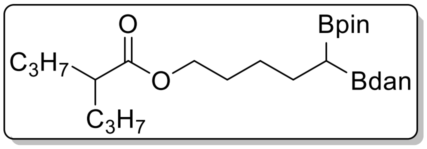

23

The reaction was performed following the general procedure A. The residue was purified by flash column chromatograph to give the product as a crystalline solid (64.8 mg, 64%). **^{1}H NMR** (500 MHz, $CDCl_3$) δ 7.08 (t, *J* = 7.8 Hz, 2H), 6.98 (d, *J* = 8.2 Hz, 2H), 6.29 (d, *J* = 7.3 Hz, 2H), 5.79 (s, 2H), 4.07 – 4.03 (m, 2H), 2.35 – 2.32 (m, 1H), 1.66 (d, *J* = 7.1 Hz, 2H), 1.57 (dd, *J* = 10.5, 4.9 Hz, 4H), 1.41 – 1.36 (m, 4H), 1.27 (d, *J* = 7.7 Hz, 4H), 1.24 (s, 3H), 1.23 (d, *J* = 2.4 Hz, 6H), 1.22 (s, 3H), 0.88 (d, *J* = 5.4 Hz, 6H), 0.75 – 0.72 (m, 1H). **^{13}C NMR** (126 MHz, $CDCl_3$) δ 176.7, 141.3, 136.3, 127.5, 119.5, 117.3, 105.5, 83.2, 83.0, 64.0, 45.4,

34.7, 28.9, 28.8, 28.6, 26.1, 25.0, 24.9, 24.5, 20.6, 14.0. **^{11}B NMR** (160 MHz, $CDCl_3$) δ 34.80, 34.45. **HRMS** (ESI, m/z) calcd for $C_{29}H_{45}B_2N_2O_4$ [M+H] $^+$: 507.3560; found: 507.3559.

5-(1H-naphtho[1,8-de][1,3,2]diazaborinin-2(3H)-yl)-5-(4,4,5,5-tetramethyl-1,3,2-dioxaborolan-2-yl)pentyl 1-phenylcyclopentane-1-carboxylate (24)

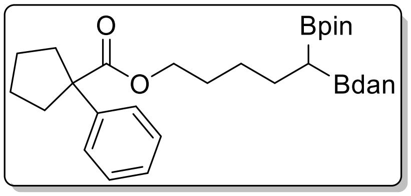

24

The reaction was performed following the general procedure A. The residue was purified by flash column chromatograph to give the product as a crystalline solid (46.4 mg, 42%).**^{1}H NMR** (500 MHz, $CDCl_3$) δ 7.32 (d, J = 7.8 Hz, 3H), 7.25 (s, 1H), 7.17 (d, J = 7.4 Hz, 1H), 7.07 (t, J = 7.8 Hz, 2H), 6.97 (d, J = 8.3 Hz, 2H), 6.27 (d, J = 7.3 Hz, 2H), 5.74 (s, 2H), 3.98 (d, J = 5.3 Hz, 2H), 2.62 (dd, J = 12.6, 6.2 Hz, 2H), 1.85 (d, J = 11.0 Hz, 2H), 1.70 – 1.65 (m, 4H), 1.57 – 1.49 (m, 4H), 1.25 (s, 2H), 1.21 (d, J = 7.6 Hz, 12H), 0.65 (t, J = 7.6 Hz, 1H). **^{13}C NMR** (126 MHz, $CDCl_3$) δ 176.0, 143.5, 141.3, 136.3, 128.2, 127.5, 126.9, 126.6, 117.3, 105.5, 83.2, 64.8, 59.2, 36.1, 28.7, 28.5, 26.0, 24.99, 24.52, 23.6. **^{11}B NMR** (160 MHz, $CDCl_3$) δ 35.15, 34.45. **HRMS** (ESI, m/z) calcd for $C_{33}H_{43}B_2N_2O_4$ [M+H] $^+$: 553.3403; found: 553.3398.

5-(1H-naphtho[1,8-de][1,3,2]diazaborinin-2(3H)-yl)-5-(4,4,5,5-tetramethyl-1,3,2-dioxaborolan-2-yl)pentyl (2R)-2-phenylpropanoate (25)

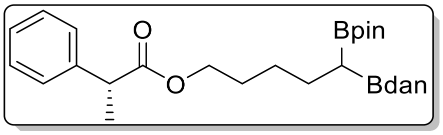

25

The reaction was performed following the Condition A. The residue was purified by flash column chromatograph (PE:EA=30:1) to give the product as a yellow oil liquid (60.4 mg, 59%). **^{1}H NMR** (500 MHz, $CDCl_3$) δ 7.34 – 7.30 (m, 3H), 7.24 (ddt, J = 8.4, 5.9, 3.4 Hz, 2H), 7.10 (t, J = 7.8 Hz, 2H), 7.00 (d, J = 8.3 Hz, 2H), 6.30 (d, J = 7.2 Hz, 2H), 5.79 (s, 2H), 4.08 (t, J = 7.0 Hz, 2H), 3.70 (d, J = 7.2 Hz, 1H), 1.61 (d, J = 7.3 Hz, 2H), 1.49 (d, J = 7.2 Hz, 3H), 1.36 – 1.27 (m, 4H), 1.25 (s, 6H), 1.23 (s, 6H), 0.71 (dd, J = 9.5, 6.0 Hz, 1H). **^{13}C NMR** (126 MHz, $CDCl_3$) δ 174.6, 141.3, 140.7, 136.3, 128.6, 127.6, 127.5, 127.1, 119.5, 117.3, 105.5, 83.2, 83.0, 64.7, 45.6, 28.7, 28.5, 26.0, 25.98, 25.0, 24.9, 24.54, 24.52, 18.5. **^{11}B NMR** (160 MHz, $CDCl_3$) δ 34.44, 33.92. **HRMS** (ESI, m/z) calcd for $C_{30}H_{39}B_2N_2O_4$ [M+H] $^+$: 513.3090; found: 513.3094. **HPLC analysis**: HPLC DAICEL CHIRALCEL OD, hexane/isopropanol = 90/10, 0.5 mL/min, λ = 280 nm, t_R (major) = 14.5 min, t_R (minor) = 16.2 min, dr ≈ 1:1.

5-(1H-naphtho[1,8-de][1,3,2]diazaborinin-2(3H)-yl)-5-(4,4,5,5-tetramethyl-1,3,2-dioxaborolan-2-yl)pentyl benzoate (26)

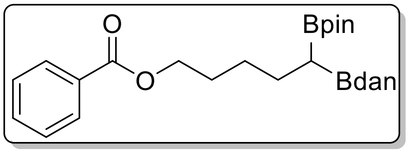

26

The reaction was performed following the general procedure A. The residue was purified by flash column chromatograph to give the product as a crystalline solid (63.9 mg, 66%). **^{1}H NMR** (500 MHz, $CDCl_3$) δ 8.06 (d, *J* = 1.4 Hz, 1H), 8.05 (d, *J* = 1.7 Hz, 1H), 7.55 – 7.52 (m, 1H), 7.41 (t, *J* = 7.8 Hz, 2H), 7.10 (t, *J* = 7.8 Hz, 2H), 7.01 (d, *J* = 8.3 Hz, 2H), 6.30 (d, *J* = 7.3 Hz, 2H), 5.84 (s, 2H), 4.35 (t, *J* = 6.6 Hz, 2H), 1.82 (t, *J* = 7.0 Hz, 2H), 1.67 – 1.47 (m, 4H), 1.25 (s, 6H), 1.23 (s, 6H), 0.80 (dd, *J* = 9.4, 5.7 Hz, 1H). **^{13}C NMR** (126 MHz, $CDCl_3$) δ 166.7, 141.3, 136.3, 132.8, 130.5, 129.6, 128.3, 127.6, 119.6, 117.3, 105.5, 83.2, 64.9, 28.9, 28.7, 26.1, 25.0, 24.87, 24.54, 24.51. **^{11}B NMR** (160 MHz, $CDCl_3$) δ 33.92, 32.98. **HRMS** (ESI, m/z) calcd for $C_{28}H_{35}B_2N_2O_4$ [M+H] $^+$:485.2777; found: 485.2773.

5-(1H-naphtho[1,8-de][1,3,2]diazaborinin-2(3H)-yl)-5-(4,4,5,5-tetramethyl-1,3,2-dioxaborolan-2-yl)pentyl (2S)-2-(4-isobutylphenyl)propanoate (27)

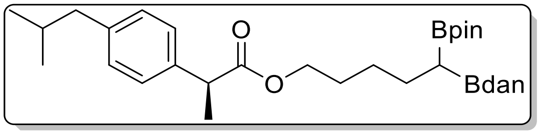

27

The reaction was performed following the general procedure A. The residue was purified by flash column chromatograph to give the product as a crystalline solid (67.1 mg, 59%). **^{1}H NMR** (500 MHz, $CDCl_3$) δ 7.18 (d, *J* = 8.0 Hz, 2H), 7.08 (dd, *J* = 9.3, 1.8 Hz, 4H), 7.00 (d, *J* = 7.7 Hz, 2H), 6.30 (d, *J* = 7.3 Hz, 2H), 5.78 (s, 2H), 4.06 (dd, *J* = 11.4, 4.7 Hz, 2H), 3.67 (d, *J* = 7.1 Hz, 1H), 2.43 (d, *J* = 7.2 Hz, 2H), 1.83 (dd, *J* = 11.1, 4.4 Hz, 1H), 1.63 – 1.54 (m, 4H), 1.48 – 1.46 (m, 3H), 1.36 – 1.31 (m, 2H), 1.24 (d, *J* = 7.3 Hz, 12H), 0.90 (d, *J* = 1.0 Hz, 6H), 0.71 (dd, *J* = 8.0, 4.7 Hz, 1H). **^{13}C NMR** (126 MHz, $CDCl_3$) δ 174.9, 141.3, 140.4, 137.8, 136.3, 129.3, 127.5, 127.1, 119.5, 117.3, 105.5, 83.2, 83.0, 64.6, 45.2, 45.0, 30.2, 28.7, 28.5, 26.0, 25.0, 24.9, 24.5, 24.5 22.4, 18.5. **^{11}B NMR** (160 MHz, $CDCl_3$) δ 34.62, 34.09. **HRMS** (ESI, m/z) calcd for $C_{34}H_{47}B_2N_2O_4$ [M+H] $^+$: 569.3716; found: 569.3713. **HPLC analysis**: HPLC DAICEL CHIRALCEL OD, hexane/isopropanol = 90/10, 1.0 mL/min, λ = 280 nm, t_R (minor) = 5.6 min, t_R (major) = 6.4 min, dr ≈1:1.

5-(1H-naphtho[1,8-de][1,3,2]diazaborinin-2(3H)-yl)-5-(4,4,5,5-tetramethyl-1,3,2-dioxaborolan-2-yl)pentyl (E)-2-methyl-3-phenylacrylate (28)

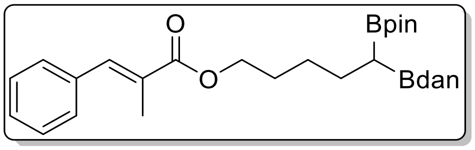

28

The reaction was performed following the general procedure A. The residue was purified by flash column chromatograph to give the product as a crystalline solid (61.9 mg, 59%). **^{1}H NMR** (500 MHz, $CDCl_3$) δ 7.69

(s, 1H), 7.41 – 7.37 (m, 4H), 7.32 (q, J = 5.2, 4.4 Hz, 1H), 7.08 (t, J = 7.7 Hz, 2H), 7.00 (d, J = 8.3 Hz, 2H), 6.29 (d, J = 7.2 Hz, 2H), 5.81 (s, 2H), 4.24 (td, J = 6.6, 1.9 Hz, 2H), 2.11 (s, 3H), 1.76 (q, J = 7.1 Hz, 2H), 1.65 – 1.43 (m, 4H), 1.24 (d, J = 6.7 Hz, 12H), 0.90 (t, J = 6.8 Hz, 1H). **^{13}C NMR** (126 MHz, $CDCl_3$) δ 168.8, 141.3, 138.7, 136.3, 135.96, 129.65, 128.36, 128.25, 127.56, 119.52, 117.30, 105.50, 83.25, 64.93, 28.87, 28.77, 26.12, 25.04, 24.51, 14.08. **^{11}B NMR** (160 MHz, $CDCl_3$) δ 33.24, 33.15. **HRMS** (ESI, m/z) calcd for $C_{31}H_{39}B_2N_2O_4$ [M+H] $^+$: 525.3090; found: 525.3092.

5-(1H-naphtho[1,8-de][1,3,2]diazaborinin-2(3H)-yl)-5-(4,4,5,5-tetramethyl-1,3,2-dioxaborolan-2-yl)pentyl (1R,2S,4R)-bicyclo[2.2.1]hept-5-ene-2-carboxylate (29)

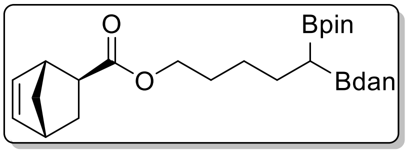

29

The reaction was performed following the general procedure A. The residue was purified by flash column chromatograph to give the product as a crystalline solid (63.0 mg, 63%). **^{1}H NMR** (500 MHz, $CDCl_3$) δ 7.09 (t, J = 7.7 Hz, 2H), 6.99 (d, J = 8.2 Hz, 2H), 6.29 (d, J = 7.2 Hz, 2H), 6.12 (dd, J = 5.7, 3.1 Hz, 1H), 6.09 – 6.06 (m, 1H), 5.79 (s, 2H), 4.11 – 4.07 (m, 2H), 3.02 (s, 1H), 2.89 (s, 1H), 2.22 – 2.19 (m, 1H), 1.93 – 1.89 (m, 1H), 1.67 (dd, J = 12.3, 5.1 Hz, 4H), 1.50 (s, 1H), 1.39 – 1.31 (m, 4H), 1.24 (d, J = 6.6 Hz, 12H), 0.75 (dd, J = 9.4, 6.1 Hz, 1H). **^{13}C NMR** (126 MHz, $CDCl_3$) δ 175.1, 141.3, 136.3, 127.5, 117.3, 105.5, 83.2, 64.2, 46.1, 40.5, 40.1, 37.0, 31.9, 29.1, 28.9, 28.64, 28.62, 26.1, 25.02, 25.01, 24.9, 24.8, 24.53, 24.51. **^{11}B NMR** (160 MHz, $CDCl_3$) δ 35.22, 34.45. **HRMS** (ESI, m/z) calcd for $C_{29}H_{38}{}^{10}B_2KN_2O_4$ [M+K] $^+$: 537.2722; found: 537.2725. **HPLC analysis**: HPLC DAICEL CHIRALCEL AD, hexane/isopropanol = 97/3, 0.5 mL/min, λ = 280 nm, t_R (major) = 19.4 min, t_R (minor) = 21.0 min, dr ≈1:1.

5-(1H-naphtho[1,8-de][1,3,2]diazaborinin-2(3H)-yl)-5-(4,4,5,5-tetramethyl-1,3,2-dioxaborolan-2-yl)pentyl 2,3-dihydrobenzo[b][1,4]dioxine-2-carboxylate (30)

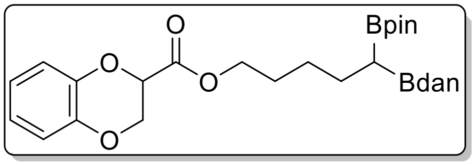

30

The reaction was performed following the general procedure A. The residue was purified by flash column chromatograph to give the product as a crystalline solid (66.6 mg, 59%). **^{1}H NMR** (500 MHz, $CDCl_3$) δ 7.09 (t, J = 7.7 Hz, 2H), 6.99 (d, J = 7.6 Hz, 3H), 6.86 (d, J = 1.6 Hz, 3H), 6.30 (d, J = 7.3 Hz, 2H), 5.79 (s, 2H), 4.81 (dd, J = 3.9, 2.8 Hz, 1H), 4.39 – 4.32 (m, 2H), 4.22 (dt, J = 13.8, 5.0 Hz, 2H), 1.69 – 1.65 (m, 2H), 1.60 (d, J = 5.7 Hz, 1H), 1.56 – 1.49 (m, 1H), 1.41 – 1.33 (m, 2H), 1.24 (d, J = 7.2 Hz, 12H), 0.72 (dd, J = 9.0, 6.3 Hz, 1H). **^{13}C NMR** (126 MHz, $CDCl_3$) δ 168.1, 143.0, 142.4, 141.3, 136.3, 127.6, 126.2, 122.2, 121.8,

119.8, 119.5, 117.4, 117.34, 117.28, 111.6, 105.5, 83.3, 83.0, 72.1, 65.9, 65.9, 65.0, 31.6, 28.6, 28.4, 28.3, 26.0, 25.9, 25.0, 24.9, 24.5, 22.7, 14.1. **^{11}B NMR** (160 MHz, $CDCl_3$) δ 33.33, 32.34. **HRMS** (ESI, m/z) calcd for $C_{30}H_{36}B_2N_2NaO_6$ [M+Na] $^+$: 565.2652; found: 565.2644.

5-(1H-naphtho[1,8-de][1,3,2]diazaborinin-2(3H)-yl)-5-(4,4,5,5-tetramethyl-1,3,2-dioxaborolan-2-yl)pentyl 6-(3-(bicyclo[3.3.1]nonan-1-yl)-4-methoxyphenyl)-2-naphthoate (31)

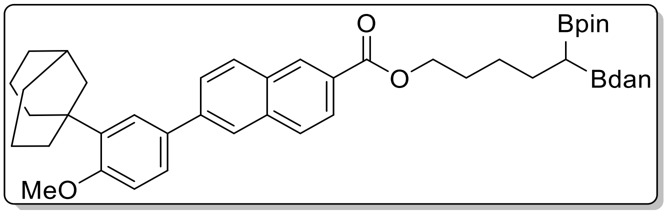

31

The reaction was performed following the general procedure A. The residue was purified by flash column chromatograph to give the product as a crystalline solid (73.2 mg, 48%). **^{1}H NMR** (500 MHz, $CDCl_3$) δ 8.63 (d, *J* = 1.7 Hz, 1H), 8.09 (dd, *J* = 8.6, 1.8 Hz, 1H), 8.08 – 7.73 (m, 5H), 7.64 (d, *J* = 2.4 Hz, 1H), 7.56 (dd, *J* = 8.4, 2.3 Hz, 1H), 7.10 (dd, *J* = 8.3, 7.3 Hz, 2H), 7.02 – 7.02 (m, 1H), 7.01 – 7.00 (m, 1H), 6.30 (dd, *J* = 7.2, 1.0 Hz, 2H), 5.85 (s, 2H), 4.44 (td, *J* = 6.5, 1.5 Hz, 2H), 3.92 (s, 3H), 2.23 (d, *J* = 3.0 Hz, 6H), 2.16 – 2.12 (m, 3H), 1.89 (t, *J* = 7.0 Hz, 2H), 1.84 (s, 6H), 1.78 – 1.75 (m, 1H), 1.68 – 1.60 (m, 2H), 1.26 (d, *J* = 7.3 Hz, 12H), 0.94 – 0.90 (m, 1H), 0.84 (dd, *J* = 9.5, 5.4 Hz, 1H). **^{13}C NMR** (126 MHz, $CDCl_3$) δ 166.9, 159.0, 141.3, 139.0, 136.3, 136.0, 132.6, 131.3, 130.8, 129.7, 128.2, 127.6, 127.3, 126.5, 126.0, 125.8, 125.6, 124.7, 119.6, 117.3, 112.2, 105.6, 83.3, 65.1, 55.2, 40.7, 37.3, 37.2, 31.6, 29.2, 29.0, 28.9, 26.2, 25.1, 24.5, 22.7, 14.2. **^{11}B NMR** (160 MHz, $CDCl_3$) δ 36.03, 35.68. **HRMS** (ESI, m/z) calcd for $C_{48}H_{57}B_2N_2O_5$ [M+H] $^+$: 763.4448; found: 763.4443.

5-(1H-naphtho[1,8-de][1,3,2]diazaborinin-2(3H)-yl)-5-(4,4,5,5-tetramethyl-1,3,2-dioxaborolan-2-yl)pentyl (2S)-2-(6-methoxynaphthalen-2-yl)propanoate (32)

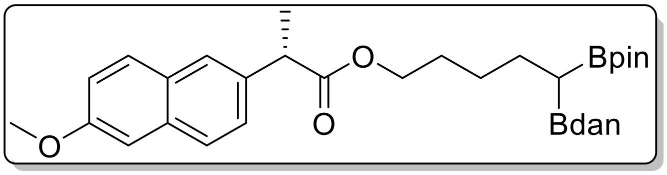

32

The reaction was performed following the general procedure A. The residue was purified by flash column chromatograph to give the product as a crystalline solid (68.7 mg, 58%). **^{1}H NMR** (500 MHz, $CDCl_3$) δ 7.71 – 7.67 (m, 3H), 7.42 (dd, *J* = 8.5, 1.9 Hz, 1H), 7.17 – 7.12 (m, 2H), 7.11 – 7.10 (m, 2H), 7.02 (d, *J* = 8.2 Hz, 2H), 6.31 (d, *J* = 7.3 Hz, 2H), 5.78 (s, 2H), 4.11 (t, *J* = 6.8 Hz, 2H), 3.90 (d, *J* = 5.2 Hz, 3H), 3.85 (d, *J* = 7.1 Hz, 1H), 1.63 (d, *J* = 7.4 Hz, 2H), 1.59 (d, *J* = 7.1 Hz, 3H), 1.50 (ddt, *J* = 16.8, 12.9, 6.9 Hz, 2H), 1.32 – 1.29 (m, 2H), 1.25 (s, 6H), 1.23 (s, 6H), 0.85 (dd, 1H). **^{13}C NMR** (126 MHz, $CDCl_3$) δ 174.8, 157.6, 141.3, 136.3, 135.9, 133.7, 129.3, 129.0, 127.6, 127.1, 126.3, 125.9, 119.6, 119.0, 117.3, 105.5, 83.2, 64.7, 55.3, 45.6, 29.8,

28.7, 28.5, 25.97, 25.95, 25.0, 24.9, 24.5, 18.5. **^{11}B NMR** (160 MHz, $CDCl_3$) δ 34.62, 33.51. **HRMS** (ESI, m/z) calcd for $C_{35}H_{43}B_2N_2O_5$ [M+H] $^+$:593.3353; found: 593.3346. **HPLC analysis**: HPLC DAICEL CHIRALCEL OD, hexane/isopropanol = 90/10, 0.5 mL/min, λ = 250 nm, t_R (minor) = 9.4 min, t_R (major) = 11.8 min, dr = 0.94:1.

5-(1H-naphtho[1,8-de][1,3,2]diazaborinin-2(3H)-yl)-5-(4,4,5,5-tetramethyl-1,3,2-dioxaborolan-2-yl)pentyl 4-cyclohexylbenzoate (33)

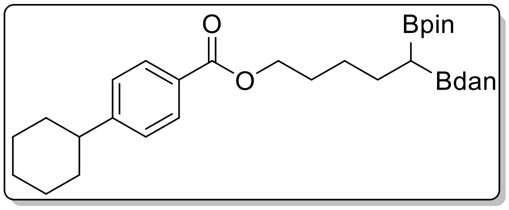

33

The reaction was performed following the general procedure A. The residue was purified by flash column chromatograph to give the product as a crystalline solid (77.0 mg, 68%). **^{1}H NMR** (500 MHz, $CDCl_3$) δ 7.96 (s, 1H), 7.95 (s, 1H), 7.22 (d, *J* = 8.3 Hz, 2H), 7.11 – 7.07 (m, 2H), 7.00 (d, *J* = 8.2 Hz, 2H), 6.29 (d, *J* = 7.3 Hz, 2H), 5.82 (s, 2H), 4.32 (t, *J* = 6.5 Hz, 2H), 2.56 – 2.52 (m, 1H), 1.87 – 1.85 (m, 4H), 1.79 (d, *J* = 6.9 Hz, 2H), 1.76 (t, *J* = 3.7 Hz, 2H), 1.66 – 1.58 (m, 2H), 1.57 – 1.50 (m, 2H), 1.40 (dd, *J* = 8.5, 3.7 Hz, 4H), 1.24 (s, 6H), 1.22 (s, 6H), 0.79 (ddd, *J* = 9.5, 6.2, 2.8 Hz, 1H). **^{13}C NMR** (126 MHz, $CDCl_3$) δ 166.8, 141.3, 129.7, 127.6, 126.8, 117.3, 105.5, 83.2, 64.7, 44.7, 34.2, 28.9, 28.8, 26.8, 26.1, 25.0, 24.9, 24.5, 24.5. **^{11}B NMR** (160 MHz, $CDCl_3$) δ 34.71, 33.02. **HRMS** (ESI, m/z) calcd for $C_{34}H_{45}B_2N_2O_4$ [M+H] $^+$:567.3560; found: 567.3560.

2-(2-phenyl-1-(4,4,5,5-tetramethyl-1,3,2-dioxaborolan-2-yl)ethyl)-2,3-dihydro-1H-naphtho[1,8-de][1,3,2]diazaborinine (34)

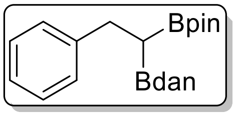

34

The reaction was performed following the general procedure A. The residue was purified by flash column chromatograph to give the product as a crystalline solid (45.4 mg, 57%). **^{1}H NMR** (500 MHz, $CDCl_3$) δ 7.25 (s, 4H), 7.17 – 7.14 (m, 1H), 7.08 (t, *J* = 7.8 Hz, 2H), 6.99 (d, *J* = 8.2 Hz, 2H), 6.27 (d, *J* = 7.3 Hz, 2H), 5.81 (s, 2H), 2.96 (dd, *J* = 14.1, 9.6 Hz, 1H), 2.91 – 2.86 (m, 1H), 1.28 – 1.25 (m, 1H), 1.18 (s, 6H), 1.16 (s, 6H). **^{13}C NMR** (126 MHz, $CDCl_3$) δ 143.7, 141.2, 136.3, 128.3, 128.2, 127.5, 125.7, 119.5, 117.3, 105.5, 83.4, 32.0, 24.9, 24.5. **^{11}B NMR** (160 MHz, $CDCl_3$) δ 34.27, 33.68. **HRMS** (ESI, m/z) calcd for $C_{24}H_{29}B_2N_2O_2$ [M+H] $^+$: 399.2410; found: 399.2418.

2-(1-(4,4,5,5-tetramethyl-1,3,2-dioxaborolan-2-yl)-2-(p-tolyl)ethyl)-2,3-dihydro-1H-naphtho[1,8-de][1,3,2]diazaborinine (35)

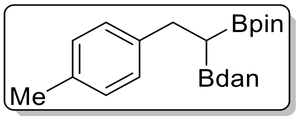

35

The reaction was performed following the general procedure A. The residue was purified by flash column chromatograph to give the product as a crystalline solid (42.9 mg, 52%). **^{1}H NMR** (500 MHz, $CDCl_3$) δ 7.17 (d, *J* = 7.8 Hz, 2H), 7.11 – 7.07 (m, 4H), 7.00 (d, *J* = 8.2 Hz, 2H), 6.28 (d, *J* = 7.3 Hz, 2H), 5.83 (s, 2H), 2.95 (dd, *J* = 14.2, 9.3 Hz, 1H), 2.87 (t, *J* = 4.7 Hz, 1H), 2.32 (s, 3H), 1.20-1.26 (m, 1H), 1.21 (s, 6H), 1.19 (s, 6H). **^{13}C NMR** (126 MHz, $CDCl_3$) δ 141.3, 140.6, 136.3, 135.1, 129.0, 128.1, 127.6, 119.6, 117.3, 105.5, 83.4, 31.5, 29.7, 25.0, 24.8, 24.5, 21.0. **^{11}B NMR** (160 MHz, $CDCl_3$) δ 34.27, 34.09. **HRMS** (ESI, m/z) calcd for $C_{25}H_{30}B_2N_2NaO_2$ $[M+Na]^+$: 435.2386; found: 435.2381.

2-(2-(4-ethylphenyl)-1-(4,4,5,5-tetramethyl-1,3,2-dioxaborolan-2-yl)ethyl)-2,3-dihydro-1H-naphtho[1,8-de][1,3,2]diazaborinine (36)

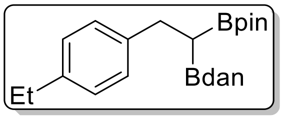

36

The reaction was performed following the general procedure A. The residue was purified by flash column chromatograph to give the product as a crystalline solid (37.9 mg, 46%). **^{1}H NMR** (500 MHz, $CDCl_3$) δ 7.14 (d, *J* = 7.9 Hz, 2H), 7.10 – 7.05 (m, 4H), 6.98 (d, *J* = 8.2 Hz, 2H), 6.26 (d, *J* = 7.3 Hz, 2H), 5.81 (s, 2H), 2.93 (dd, *J* = 14.2, 9.3 Hz, 1H), 2.84 (dd, *J* = 14.2, 6.8 Hz, 1H), 2.30 (s, 3H), 1.26 (s, 2H), 1.18 (d, *J* = 8.4 Hz, 12H), 1.15 – 1.13 (m, 1H). **^{13}C NMR** (126 MHz, $CDCl_3$) δ 141.2 (s), 140.6 (s), 135.1 (s), 128.9 (s), 128.0 (s), 127.5 (s), 117.3 (s), 105.5 (s), 83.3 (s), 31.5 (s), 25.0 (s), 24.5 (s), 21.0 (s). **^{11}B NMR** (160 MHz, $CDCl_3$) δ 34.45, 34.09. **HRMS** (ESI, m/z) calcd for $C_{26}H_{33}B_2N_2O_2$ $[M+H]^+$: 427.2723; found: 427.2722.

2-(2-(4-(tert-butyl)phenyl)-1-(4,4,5,5-tetramethyl-1,3,2-dioxaborolan-2-yl)ethyl)-2,3-dihydro-1H-naphtho[1,8-de][1,3,2]diazaborinine (37)

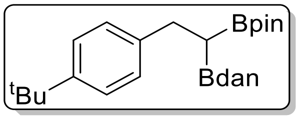

37

The reaction was performed following the general procedure A. The residue was purified by flash column chromatograph to give the product as a crystalline solid (56.3 mg, 62%). **^{1}H NMR** (500 MHz, $CDCl_3$) δ 7.31 – 7.28 (m, 2H), 7.20 (d, *J* = 8.3 Hz, 2H), 7.09 (dd, *J* = 8.3, 7.3 Hz, 2H), 7.00 (dd, *J* = 8.4, 1.0 Hz, 2H), 6.27 (d, *J* = 7.3 Hz, 2H), 5.82 (s, 2H), 2.97 – 2.85 (m, 2H), 1.30 (s, 9H), 1.24 (d, *J* = 4.3 Hz, 1H), 1.19 (s, 6H), 1.17 (s, 6H). **^{13}C NMR** (126 MHz, $CDCl_3$) δ 141.3, 140.6, 136.3, 127.8, 127.5, 125.1, 117.3, 105.5, 83.3, 34.3, 31.4, 24.9, 24.5. **^{11}B NMR** (160 MHz, $CDCl_3$) δ 34.62, 33.92. **HRMS** (ESI, m/z) calcd for $C_{28}H_{36}B_2KN_2O_2$ $[M+K]^+$: 493.2594; found: 493.2588.

2-(2-(4-isopropylphenyl)-1-(4,4,5,5-tetramethyl-1,3,2-dioxaborolan-2-yl)ethyl)-2,3-dihydro-1H-

naphtho[1,8-de][1,3,2]diazaborinine (38)

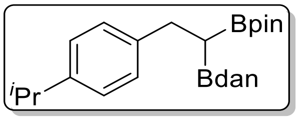

38

The reaction was performed following the general procedure A. The residue was purified by flash column chromatograph to give the product as a crystalline solid (31.7 mg, 36%). **^{1}H NMR** (500 MHz, $CDCl_3$) δ 7.19 (d, *J* = 8.2 Hz, 2H), 7.13 (d, *J* = 8.2 Hz, 2H), 7.09 (dd, *J* = 8.3, 7.3 Hz, 2H), 7.00 (dd, *J* = 8.4, 1.0 Hz, 2H), 6.27 (dd, *J* = 7.3, 1.0 Hz, 2H), 5.82 (s, 2H), 2.94 (dd, *J* = 14.2, 9.3 Hz, 1H), 2.87 (ddd, *J* = 13.7, 6.9, 2.1 Hz, 2H), 1.23 (d, *J* = 6.9 Hz, 6H), 1.19 (s, 1H), 1.18 (d, *J* = 6.9 Hz, 12H). **^{13}C NMR** (126 MHz, $CDCl_3$) δ 141.2, 141.0, 136.3, 1281 127.5, 126.3, 117.3, 105.5, 83.3, 33.7, 31.5, 24.9, 24.5, 24.1. **^{11}B NMR** (160 MHz, $CDCl_3$) δ 33.33, 32.10. **HRMS** (ESI, m/z) calcd for $C_{27}H_{34}B_2N_2NaO_2$ [M+Na] $^{+}$: 463.2699; found: 463.2706.

2-(2-(4-butylphenyl)-1-(4,4,5,5-tetramethyl-1,3,2-dioxaborolan-2-yl)ethyl)-2,3-dihydro-1H-naphtho[1,8-de][1,3,2]diazaborinine (39)

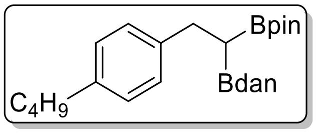

39

The reaction was performed following the general procedure A. The residue was purified by flash column chromatograph to give the product as a crystalline solid (40.9 mg, 45 %). ^{1}H NMR (500 MHz, $CDCl_3$) δ 7.17 (d, *J* = 8.0 Hz, 2H), 7.08 (t, *J* = 7.8 Hz, 4H), 6.99 (d, *J* = 8.2 Hz, 2H), 6.26 (d, *J* = 7.3 Hz, 2H), 5.81 (s, 2H), 2.93 (dd, *J* = 14.1, 9.4 Hz, 1H), 2.86 (dd, *J* = 14.2, 6.9 Hz, 1H), 2.58 – 2.54 (m, 2H), 1.58 – 1.53 (m, 2H), 1.36 – 1.31 (m, 2H), 1.27 (d, *J* = 7.4 Hz, 1H), 1.18 (s, 6H), 1.17 (s, 6H), 0.91 (t, *J* = 7.3 Hz, 3H). **^{13}C NMR (**126 MHz, $CDCl_3$) δ 141.2, 140.8, 140.2, 136.3, 128.3, 128.0, 127.5 119.5, 117.3, 105.5, 83.3, 35.2, 33.7, 31.6, 24.9, 24.5, 22.3, 14.0. ^{11}B NMR (160 MHz, $CDCl_3$) δ 34.09, 33.51.

HRMS (ESI, m/z) calcd for $C_{28}H_{36}B_2KN_2O_2$ [M+K] $^{+}$: 493.2594; found: 493.2598.

2-(2-(4-propylphenyl)-1-(4,4,5,5-tetramethyl-1,3,2-dioxaborolan-2-yl)ethyl)-2,3-dihydro-1H-naphtho[1,8-de][1,3,2]diazaborinine (40)

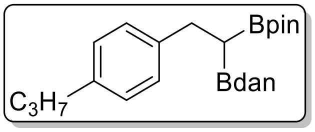

40

The reaction was performed following the general procedure A. The residue was purified by flash column chromatograph to give the product as a crystalline solid (45.8 mg, 52%). **^{1}H NMR** (500 MHz, $CDCl_3$) δ 7.19 (d, *J* = 7.8 Hz, 2H), 7.10 (t, *J* = 7.8 Hz, 4H), 7.01 (dd, *J* = 8.3, 1.0 Hz, 2H), 6.28 (dd, *J* = 7.3, 1.0 Hz, 2H), 5.84 (s, 2H), 2.96 (dd, *J* = 14.2, 9.4 Hz, 1H), 2.88 (dd, *J* = 14.1, 6.9 Hz, 1H), 2.58 – 2.55 (m, 2H), 1.66 – 1.61 (m, 2H), 1.24 – 1.22 (m, 1H), 1.20 (d, *J* = 7.8 Hz, 12H), 0.94 (t, *J* = 7.3 Hz, 3H). **^{13}C NMR** (126 MHz, $CDCl_3$)

δ 141.3, 140.9, 140.0, 136.3, 128.4, 128.1, 127.6, 119.6, 117.3, 105.5, 83.4, 37.7, 31.6, 24.9, 24.7, 24.5, 13.8. **^{11}B NMR** (160 MHz, $CDCl_3$) δ 33.68, 32.10. **HRMS** (ESI, m/z) calcd for $C_{27}H_{35}B_2N_2O_2$ $[M+H]^+$: 441.2879; found: 441.2878.

2-(2-([1,1'-biphenyl]-4-yl)-1-(4,4,5,5-tetramethyl-1,3,2-dioxaborolan-2-yl)ethyl)-2,3-dihydro-1H-naphtho[1,8-de][1,3,2]diazaborinine (41)

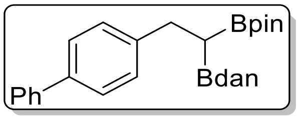

41

The reaction was performed following the general procedure A. The residue was purified by flash column chromatograph to give the product as a crystalline solid (45.6 mg, 48%). **^{1}H NMR** (500 MHz, $CDCl_3$) δ 7.58 (dd, *J* = 7.0, 1.6 Hz, 2H), 7.51 (d, *J* = 8.2 Hz, 2H), 7.44 – 7.41 (m, 3H), 7.35 (s, 1H), 7.33 (d, *J* = 3.7 Hz, 1H), 7.09 (dd, *J* = 8.3, 7.3 Hz, 2H), 7.00 (d, *J* = 8.3 Hz, 2H), 6.29 (dd, *J* = 7.3, 1.1 Hz, 2H), 5.84 (s, 2H), 3.03 – 2.98 (m, 1H), 2.96 – 2.91 (m, 1H), 1.22 (s, 1H), 1.20 (s, 6H), 1.18 (s, 6H). **^{13}C NMR** (126 MHz, $CDCl_3$) δ 142.9, 141.2, 138.7, 136.3, 128.7, 128.6, 127.6, 127.0, 127.0, 117.4, 105.6, 83.4, 31.7, 29.7, 25.0, 24.5. **^{11}B NMR** (160 MHz, $CDCl_3$) δ 34.09, 33.51. **HRMS** (ESI, m/z) calcd for $C_{30}H_{33}B_2N_2O_2$ $[M+H]^+$: 475.2723; found: 475.2723.

2-(2-(4-methoxyphenyl)-1-(4,4,5,5-tetramethyl-1,3,2-dioxaborolan-2-yl)ethyl)-2,3-dihydro-1H-naphtho[1,8-de][1,3,2]diazaborinine (42)

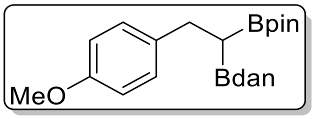

42

The reaction was performed following the general procedure A. The residue was purified by flash column chromatograph to give the product as a crystalline solid (40.3 mg, 47%). **^{1}H NMR** (500 MHz, $CDCl_3$) δ 7.19 (d, *J* = 8.6 Hz, 2H), 7.11 – 7.08 (m, 2H), 7.00 (d, *J* = 8.2 Hz, 2H), 6.83 – 6.80 (m, 2H), 6.28 (d, *J* = 7.3 Hz, 2H), 5.83 (s, 2H), 3.78 (s, 3H), 2.92 (dd, *J* = 14.1, 9.5 Hz, 1H), 2.85 (s, 1H), 1.31 – 1.26 (m, 1H), 1.20 (s, *J* = 1.3 Hz, 6H), 1.19 (s, 3H), 1.18 (s, 3H). **^{13}C NMR** (126 MHz, $CDCl_3$) δ 157.7, 141.2, 136.3, 135.9, 129.1, 127.6, 117.3, 113.7, 105.5, 83.4, 55.3, 31.1, 25.0, 24.8, 24.55 (s), 24.5. **^{11}B NMR** (160 MHz, $CDCl_3$) δ 34.97, 34.45. **HRMS** (ESI, m/z) calcd for $C_{25}H_{31}B_2N_2O_3$ $[M+H]^+$: 429.2515; found: 429.2508.

2-(2-(3-methoxyphenyl)-1-(4,4,5,5-tetramethyl-1,3,2-dioxaborolan-2-yl)ethyl)-2,3-dihydro-1H-naphtho[1,8-de][1,3,2]diazaborinine (43)

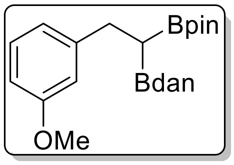

43

The reaction was performed following the general procedure A. The residue was purified by flash column chromatograph to give the product as a crystalline solid (36.8 mg, 43%). **^{1}H NMR** (500 MHz, $CDCl_3$) δ 7.17 (t, *J* = 7.9 Hz, 1H), 7.07 (d, *J* = 7.4 Hz, 2H), 7.00 – 6.96 (m, 2H), 6.85 (d, *J* = 7.5 Hz, 1H), 6.81 – 6.78 (m, 1H), 6.70 (dd, *J* = 8.1, 2.3 Hz, 1H), 6.26 (dd, *J* = 7.3, 0.8 Hz, 2H), 5.81 (s, 2H), 3.77 (s, 3H), 2.93 (dd, *J* = 14.1, 9.5 Hz, 1H), 2.86 (d, *J* = 8.0 Hz, 1H), 1.27 – 1.25 (m, 1H), 1.18 (s, 6H), 1.17 (d, *J* = 1.7 Hz, 6H). **^{13}C NMR** (126 MHz, $CDCl_3$) δ 159.6, 146.1, 145.4, 141.2, 136.3, 129.2, 128.9, 127.5, 120.8, 120.6, 119.5, 117.3, 116.8, 113.9, 113.8, 111.3, 111.2, 105.5, 83.4 55.1, 32.0, 31.4, 25.0, 24.8, 24.6, 24.5. **^{11}B NMR** (160 MHz, $CDCl_3$) δ 33.92, 33.68. **HRMS** (ESI, m/z) calcd for $C_{25}H_{31}B_2N_2O_3$ [M+H] $^+$: 429.2515; found: 429.2512.

2-(2-(4-(pentyloxy)phenyl)-1-(4,4,5,5-tetramethyl-1,3,2-dioxaborolan-2-yl)ethyl)-2,3-dihydro-1H-naphtho[1,8-de][1,3,2]diazaborinine (44)

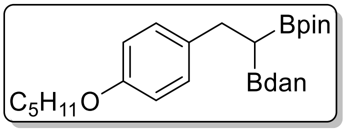

44

The reaction was performed following the general procedure A. The residue was purified by flash column chromatograph to give the product as a crystalline solid (56.2 mg, 58%). **^{1}H NMR** (500 MHz, $CDCl_3$) δ 7.16 (d, *J* = 8.5 Hz, 2H), 7.11 – 7.06 (m, 2H), 6.99 (d, *J* = 8.2 Hz, 2H), 6.80 (d, *J* = 8.6 Hz, 2H), 6.27 (d, *J* = 7.3 Hz, 2H), 5.81 (s, 2H), 3.92 (t, *J* = 6.6 Hz, 2H), 2.90 (dd, *J* = 14.1, 9.4 Hz, 1H), 2.85 – 2.81 (m, 1H), 1.81 – 1.72 (m, 3H), 1.43 – 1.37 (m, 4H), 1.19 (s, 6H), 1.18 (s, 6H), 1.14 (dd, *J* = 6.8, 2.4 Hz, 1H), 0.93 (d, *J* = 5.1 Hz, 3H). **^{13}C NMR** (126 MHz, $CDCl_3$) δ 157.3, 141.2, 136.3, 135.7, 129.2, 129.0, 127.5, 119.5, 117.3, 114.4, 114.1, 105.5, 83.3, 68.1, 31.1, 29.1, 28.2, 25.0, 24.8 24.54, 24.52, 22.5, 14.0. **^{11}B NMR** (160 MHz, $CDCl_3$) δ 34.27, 34.09. **HRMS** (ESI, m/z) calcd for $C_{29}H_{39}B_2N_2O_3$ [M+H] $^+$: 485.3141; found: 485.3147.

4-(2-(1H-naphtho[1,8-de][1,3,2]diazaborinin-2(3H)-yl)-2-(4,4,5,5-tetramethyl-1,3,2-dioxaborolan-2-yl)ethyl)-N,N-dimethylaniline (45)

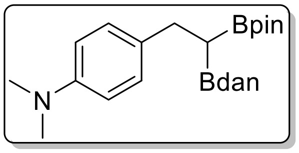

45

The reaction was performed following the general procedure A. The residue was purified by flash column chromatograph to give the product as a crystalline solid (30.9 mg, 35%). **^{1}H NMR (**500 MHz, $CDCl_3$) δ 7.15 (d, *J* = 8.6 Hz, 2H), 7.11 – 7.07 (m, 2H), 7.00 (d, *J* = 8.2 Hz, 2H), 6.70 (d, *J* = 8.6 Hz, 2H), 6.27 (d, *J* = 7.3 Hz, 2H), 5.84 (s, 2H), 2.91 (s, 6H), 2.89 – 2.79 (m, 2H), 1.21 (d, *J* = 6.4 Hz, 12H), 1.18 – 1.14 (m, 1H). **^{13}C NMR** (126 MHz, $CDCl_3$) δ 149.1, 141.3, 136.3, 128.7, 127.5, 119.5, 117.2, 113.1, 105.5, 83.3, 41.0, 30.9, 25.0, 24.6. **^{11}B NMR** (160 MHz, $CDCl_3$) δ 33.92, 32.45. **HRMS** (ESI, m/z) calcd for $C_{26}H_{34}B_2N_3O_2$ [M+H] $^+$: 442.2832; found: 442.2828.

Methyl 4-(2-(1H-naphtho[1,8-de] [1,3,2]diazaborinin-2(3H)-yl) -2-(4,4,5,5-tetramethy l-1,3,2 -

dioxaborolan-2-yl)ethyl)benzoate (46)

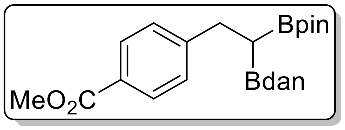

46

The reaction was performed following the general procedure A. The residue was purified by flash column chromatograph to give the product as a crystalline solid (43.8 mg, 48%). ^{1}**H NMR** (500 MHz, $CDCl_3$) δ 7.97 (s, 1H), 7.93 (d, J = 8.3 Hz, 1H), 7.32 (s, 1H), 7.29 (s, 1H), 7.09 (dd, J = 8.3, 7.3 Hz, 2H), 7.01 (dd, J = 8.3, 1.0 Hz, 2H), 6.27 (dd, J = 7.3, 1.0 Hz, 2H), 5.58 (s, 2H), 3.91 (s, 3H), 2.85 (d, J = 8.2 Hz, 1H), 2.80 (s, 1H), 1.40 – 1.37 (m, 1H), 1.21 (s, 12H). ^{13}**C NMR** (126 MHz, $CDCl_3$) δ 167.3, 149.4, 140.9, 136.3, 129.9, 129.6, 128.1, 128.0, 127.6, 117.6, 105.6, 83.2, 52.0, 31.0, 30.0, 24.8. ^{11}**B NMR** (160 MHz, $CDCl_3$) δ 34.80, 34.27. **HRMS** (ESI, m/z) calcd for $C_{26}H_{31}B_2N_2O_4$ [M+H] $^{+}$:457.2464; found: 457.2461.

2-(1-(4,4,5,5-tetramethyl-1,3,2-dioxaborolan-2-yl)-2-(4-(trifluoromethoxy)phenyl)ethyl)-2,3-dihydro-1H-naphtho[1,8-de][1,3,2]diazaborinine (47)

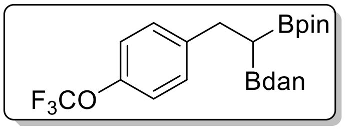

47

The reaction was performed following the general procedure B. The residue was purified by flash column chromatograph to give the product as a crystalline solid (46.3 mg, 48%). ^{1}**H NMR** (500 MHz, $CDCl_3$) δ 7.29 (d, J = 2.7 Hz, 1H), 7.27 (d, J = 3.1 Hz, 1H), 7.13 – 7.11 (m, 2H), 7.10 – 7.08 (m, 2H), 7.01 (dd, J = 8.3, 2.8 Hz, 2H), 6.30 (d, J = 7.4 Hz, 2H), 5.80 (s, 2H), 2.93 – 2.87 (m, 2H), 1.27 (s, 1H), 1.17 (s, 12H). ^{13}**C NMR** (126 MHz, $CDCl_3$) δ 147.3, 142.6, 141.1, 136.3, 129.5, 127.6, 120.8, 120.4 (q, J =119.7), 119.5, 117.5, 105.6, 83.5, 31.4, 24.9, 24.4. ^{11}**B NMR** (160 MHz, $CDCl_3$) δ 33.92, 33.15. ^{19}**F NMR** (471 MHz, $CDCl_3$) δ -57.96. **HRMS** (ESI, m/z) calcd for $C_{25}H_{28}B_2F_3N_2O_3$ [M+H] $^{+}$:483.2233; found: 483.2229.

2-(2-(4-fluorophenyl)-1-(4,4,5,5-tetramethyl-1,3,2-dioxaborolan-2-yl)ethyl)-2,3-dihydro-1H-naphtho[1,8-de][1,3,2]diazaborinine (48)

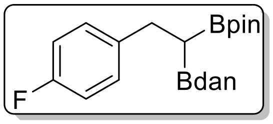

48

The reaction was performed following the general procedure B. The residue was purified by flash column chromatograph to give the product as a crystalline solid (37.5 mg, 45%). ^{1}**H NMR** (500 MHz, $CDCl_3$) δ 7.22 (dd, J = 8.4, 5.5 Hz, 2H), 7.10 (t, J = 7.8 Hz, 2H), 7.01 (d, J = 8.2 Hz, 2H), 6.95 (t, J = 8.7 Hz, 2H), 6.29 (d,

J = 7.3 Hz, 2H), 5.81 (s, 2H), 2.94 – 2.84 (m, 2H), 1.33 – 1.21 (m, 1H), 1.17 (d, J = 12.5 Hz, 12H). **^{13}C NMR** (126 MHz, $CDCl_3$) δ 161.2 (d, J = 243.4 Hz), 141.1, 139.4 (d, J = 3.1 Hz), 136.3, 129.6 (d, J = 7.9 Hz), 127.6, 119.5, 117.4, 114.9 (d, J = 21.3 Hz), 105.6, 83.4, 31.2, 25.0, 24.5. **^{11}B NMR** (160 MHz, $CDCl_3$) δ 34.54, 34.34. **^{19}F NMR** (471 MHz, $CDCl_3$) δ -117.92. **HRMS** (ESI, m/z) calcd for $C_{24}H_{28}B_2FN_2O_2$ [M+H] $^+$: 417.2315; found: 417.2307.

2-(2-(4-chlorophenyl)-1-(4,4,5,5-tetramethyl-1,3,2-dioxaborolan-2-yl)ethyl)-2,3-dihydro-1H-naphtho[1,8-de][1,3,2]diazaborinine (49)

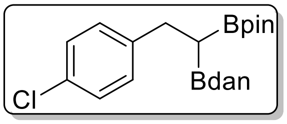

49

The reaction was performed following the general procedure B. The residue was purified by flash column chromatograph to give the product as a crystalline solid (40.6 mg, 47%). **^{1}H NMR** (500 MHz, $CDCl_3$) δ 7.23 (d, J = 8.3 Hz, 2H), 7.19 (d, J = 8.4 Hz, 2H), 7.09 (t, J = 7.8 Hz, 2H), 7.00 (d, J = 8.2 Hz, 2H), 6.28 (d, J = 7.3 Hz, 2H), 5.80 (s, 2H), 2.91 (dd, J = 14.1, 9.8 Hz, 1H), 2.85 (t, J = 3.9 Hz, 1H), 1.27 (d, J = 6.0 Hz, 1H), 1.19 (s, 6H), 1.17 (s, 3H), 1.16 (s, 3H). **^{13}C NMR** (126 MHz, $CDCl_3$) δ 142.2, 141.1, 136.3, 131.4, 129.7, 129.6, 128.3, 128.0, 127.6, 119.5, 117.5, 105.6, 83.5, 31.4, 30.7, 25.0, 24.8, 24.7, 24.5, 24.5. **^{11}B NMR** (160 MHz, $CDCl_3$) δ 34.25, 33.50. **HRMS** (ESI, m/z) calcd for $C_{24}H_{28}B_2ClN_2O_2$ [M+H] $^+$:433.2020; found: 433.2017.

2-(1-(4,4,5,5-tetramethyl-1,3,2-dioxaborolan-2-yl)-2-(4-(trifluoromethyl)phenyl)ethyl)-2,3-dihydro-1H-naphtho[1,8-de][1,3,2]diazaborinine (50)

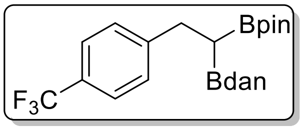

50

The reaction was performed following the general procedure B. The residue was purified by flash column chromatograph to give the product as a crystalline solid (48.5 mg, 52%). **^{1}H NMR** (500 MHz, $CDCl_3$) δ 7.51 (s, 2H), 7.37 (d, J = 9.1 Hz, 2H), 7.09 (d, J = 8.4 Hz, 2H), 7.01 (d, J = 9.1 Hz, 2H), 6.30 (d, J = 8.2 Hz, 2H), 5.81 (s, 2H), 2.95 (dd, J = 16.9, 9.6 Hz, 2H), 1.18 (s, 6H), 1.15 (s, 6H), 0.88 (s, 1H). **^{13}C NMR** (126 MHz, $CDCl_3$) δ 147.9, 141.0, 136.3, 128.5, 128.0, 127.6, 125.2 (q, J = 3.7 Hz, 1H), 119.5, 117.6, 105.6, 83.5, 31.9, 24.9, 24.44. **^{11}B NMR** (160 MHz, $CDCl_3$) δ 34.80, 34.27. **^{19}F NMR** (471 MHz, $CDCl_3$) δ -62.27. **HRMS** (ESI, m/z) calcd for $C_{25}H_{27}B_2F_3N_2NaO_2$ [M+H] $^+$: 489.2103; found: 489.2108.

2-(1-(4,4,5,5-tetramethyl-1,3,2-dioxaborolan-2-yl)-2-(m-tolyl)ethyl)-2,3-dihydro-1H-naphtho[1,8-de][1,3,2]diazaborinine (51)

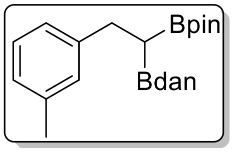

51

The reaction was performed following the general procedure A. The residue was purified by flash column chromatograph to give the product as a crystalline solid (43.0 mg, 40%). **^{1}H NMR** (500 MHz, $CDCl_3$) δ 7.14 (d, *J* = 7.5 Hz, 1H), 7.08 (t, *J* = 7.8 Hz, 5H), 6.99 (d, *J* = 8.2 Hz, 3H), 6.27 (d, *J* = 7.3 Hz, 2H), 5.81 (s, 2H), 2.92 (dd, *J* = 14.0, 9.7 Hz, 1H), 2.84 (dd, *J* = 14.1, 6.5 Hz, 1H), 2.31 (s, 3H), 1.21 (s, 1H), 1.19 (s, 6H), 1.17 (s, 6H). **^{13}C NMR** (126 MHz, $CDCl_3$) δ 143.7 (s), 141.2 (s), 137.7 (s), 129.0 (s), 128.2 (s), 127.5 (s), 126.4 (s), 125.2 (s), 117.3 (s), 105.5 (s), 83.3 (s), 31.9 (s), 25.0 (s), 24.5 (s), 21.4 (s). **^{11}B NMR** (160 MHz, $CDCl_3$) δ 34.32, 34.09. **HRMS** (ESI, m/z) calcd for $C_{25}H_{30}B_2N_2NaO_2$ [M+Na] $^+$: 435.2386; found: 435.2391.

2-(2-(3,4-dimethylphenyl)-1-(4,4,5,5-tetramethyl-1,3,2-dioxaborolan-2-yl)ethyl)-2,3-dihydro-1H-naphtho[1,8-de][1,3,2]diazaborinine (52)

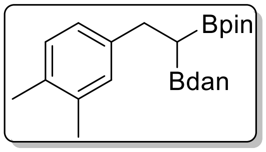

52

The reaction was performed following the general procedure A. The residue was purified by flash column chromatograph to give the product as a crystalline solid (34.1mg, 40%). **^{1}H NMR** (500 MHz, $CDCl_3$) δ 7.09 (td, *J* = 7.9, 7.5, 2.0 Hz, 2H), 7.05 – 6.98 (m, 5H), 6.27 (d, *J* = 7.4 Hz, 2H), 5.83 (s, 2H), 2.92 (ddd, *J* = 14.0, 9.3, 2.5 Hz, 1H), 2.82 (ddd, *J* = 14.1, 6.8, 2.5 Hz, 1H), 2.23 (dd, *J* = 4.6, 2.1 Hz, 6H), 1.20 (dd, *J* = 6.4, 2.0 Hz, 12H), 1.16 – 1.13 (m, 1H). **^{13}C NMR** (126 MHz, $CDCl_3$) δ 141.3, 141.2, 136.3, 136.2, 133.7, 129.5, 127.5, 125.5, 119.5, 105.5, 83.3, 31.5, 29.7, 25.0, 24.5, 19.8, 19.3. **^{11}B NMR** (160 MHz, $CDCl_3$) δ 32.27, 31.57. **HRMS** (ESI, m/z) calcd for $C_{26}H_{32}B_2KN_2O_2$ [M+K] $^+$: 465.2281; found: 465.2288.

2-(2-(3,5-dimethylphenyl)-1-(4,4,5,5-tetramethyl-1,3,2-dioxaborolan-2-yl)ethyl)-2,3-dihydro-1H-naphtho[1,8-de][1,3,2]diazaborinine (53)

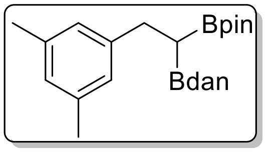

53

The reaction was performed following the general procedure A. The residue was purified by flash column chromatograph to give the product as a crystalline solid (49.4 mg, 58%). **^{1}H NMR** (500 MHz, $CDCl_3$) δ 7.09 (dd, *J* = 8.3, 7.3 Hz, 2H), 6.99 (dd, *J* = 8.2, 1.0 Hz, 2H), 6.89 (d, *J* = 1.5 Hz, 2H), 6.80 (s, 1H), 6.27 (dd, *J* = 7.2, 1.0 Hz, 2H), 5.82 (s, 2H), 2.90 (dd, *J* = 13.9, 9.9 Hz, 1H), 2.80 (dd, *J* = 13.9, 6.2 Hz, 1H), 2.28 (s, 6H), 1.19 (d, *J* = 6.1 Hz, 12H), 1.14 (d, *J* = 3.7 Hz, 1H). **^{13}C NMR** (126 MHz, $CDCl_3$) δ 143.7, 141.3, 137.6, 136.3,

127.6, 127.3, 126.1, 119.5, 117.3, 105.5, 83.3, 31.8, 29.7, 25.0, 24.5, 21.3. **^{11}B NMR** (160 MHz, $CDCl_3$) δ 34.09, 32.63. **HRMS** (ESI, m/z) calcd for $C_{26}H_{33}B_2N_2O_2$ [M+H] $^+$: 427.2723; found: 427.2725.

2-(2-(4,4-dimethylthiochroman-7-yl)-1-(4,4,5,5-tetramethyl-1,3,2-dioxaborolan-2-yl)ethyl)-2,3-dihydro-1H-naphtho[1,8-de][1,3,2]diazaborinine (54)

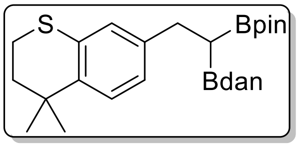

54

The reaction was performed following the general procedure A. The residue was purified by flash column chromatograph to give the product as a crystalline solid (56.8 mg, 57%). **^{1}H NMR** (500 MHz, $CDCl_3$) δ 7.25 (d, J = 1.6 Hz, 1H), 7.08 (dd, J = 8.2, 7.4 Hz, 2H), 7.01 – 6.97 (m, 3H), 6.94 (dd, J = 8.0, 1.8 Hz, 1H), 6.26 (dd, J = 7.3, 0.8 Hz, 2H), 5.81 (s, 2H), 3.01 – 2.98 (m, 2H), 2.91 – 2.79 (m, 2H), 1.94 – 1.91 (m, 2H), 1.29 (s, 3H), 1.27 (s, 3H), 1.19 (s, 6H), 1.17 (s, 6H), 1.15 – 1.12 (m, 1H). **^{13}C NMR** (126 MHz, $CDCl_3$) δ 141.8, 141.2, 139.5, 136.3, 128.4, 127.5, 126.5, 126.4, 126.0, 119.5, 117.3, 105.5, 83.34, 37.7, 33.0, 31.7, 30.31, 30.26, 25.0, 24.6, 23.1. **^{11}B NMR** (160 MHz, $CDCl_3$) δ 33.15, 32.27. **HRMS** (ESI, m/z) calcd for $C_{29}H_{37}B_2N_2O_2S$ [M+H] $^+$: 499.2756; found: 499.2754.

2-(1-(4,4,5,5-tetramethyl-1,3,2-dioxaborolan-2-yl)-2-(thiophen-3-yl)ethyl)-2,3-dihydro-1H-naphtho[1,8-de][1,3,2]diazaborinine (55)

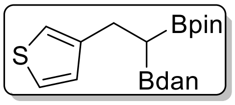

55

The reaction was performed following the general procedure A. The residue was purified by flash column chromatograph to give the product as a crystalline solid (46.9 mg, 58%). **^{1}H NMR** (500 MHz, $CDCl_3$) δ 7.56 (d, J = 3.0 Hz, 1H), 7.50 (s, 1H), 7.41 (dd, J = 5.1, 1.3 Hz, 1H), 7.10 (d, J = 7.8 Hz, 2H), 7.00 (d, J = 8.3 Hz, 2H), 6.34 (d, J = 7.3 Hz, 2H), 6.21 (s, 2H), 1.38 (s, 12H), 1.26 (d, J = 6.5 Hz, 2H), 1.20 (s, 1H). **^{13}C NMR** (126 MHz, $CDCl_3$) δ 144.6, 141.5, 136.4, 127.9, 127.6, 126.1, 125.4, 117.3, 105.7, 83.9, 53.4, 25.0, 24.8. **^{11}B NMR** (160 MHz, $CDCl_3$) δ 32.08, 31.80. **HRMS** (ESI, m/z) calcd for $C_{22}H_{27}B_2N_2O_2S$ [M+H] $^+$:405.1974; found: 405.1974.

2-(2-(1H-phenalen-5-yl)-1-(4,4,5,5-tetramethyl-1,3,2-dioxaborolan-2-yl)ethyl)-2,3-dihydro-1H-naphtho[1,8-de][1,3,2]diazaborinine (56)

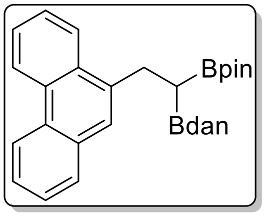

56

The reaction was performed following the general procedure A. The residue was purified by flash column chromatograph to give the product as a crystalline solid (50.6 mg, 52%). **^{1}H NMR** (500 MHz, $CDCl_3$) δ 8.77 – 8.75 (m, 1H), 8.66 (s, 1H), 8.17 (dt, *J* = 7.8, 3.0 Hz, 1H), 7.84 – 7.82 (m, 1H), 7.74 (s, 1H), 7.67 (s, 1H), 7.65 – 7.63 (m, 1H), 7.60 – 7.57 (m, 2H), 7.13 (t, *J* = 7.8 Hz, 2H), 7.05 (d, *J* = 8.2 Hz, 2H), 6.34 (d, *J* = 7.3 Hz, 2H), 5.97 (s, 2H), 3.45 (s, 2H), 1.23 (s, 3H), 1.23 (s, 3H), 1.19 (s, 3H), 1.18 (s, 3H), 0.92 (s, 1H). **^{13}C NMR** (126 MHz, $CDCl_3$) δ 141.2, 137.6, 136.4, 131.9, 131.3, 129.7, 128.1, 128.0, 127.6, 126.62, 126.60, 126.4, 126.2, 126.0, 125.7, 125.5, 125.3, 124.7, 124.4, 123.3, 123.0, 122.5, 122.4, 119.6, 117.5, 105.7, 83.5, 83.2, 31.6, 29.2, 28.6, 25.1, 24.9, 24.6, 24.5, 22.7, 14.2. **^{11}B NMR** (160 MHz, $CDCl_3$) δ 34.62, 34.09. **HRMS** (ESI, m/z) calcd for $C_{32}H_{32}B_2N_{2Na}O_2$ [M+Na] $^+$:521.2542; found: 521.2539.

2-(2-(4-(9H-carbazol-9-yl)phenyl)-1-(4,4,5,5-tetramethyl-1,3,2-dioxaborolan-2-yl)ethyl)-2,3-dihydro-1H-naphtho[1,8-de][1,3,2]diazaborinine (57)

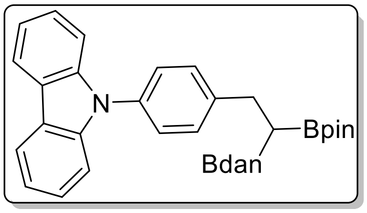

57

The reaction was performed following the general procedure A. The residue was purified by flash column chromatograph to give the product as a crystalline solid (53.0 mg, 47%). **^{1}H NMR** (500 MHz, $CDCl_3$) δ 8.16 (d, *J* = 7.8 Hz, 2H), 7.51 (d, *J* = 8.4 Hz, 2H), 7.48 – 7.46 (m, 2H), 7.43 – 7.40 (m, 2H), 7.40 – 7.37 (m, 2H), 7.30 – 7.28 (m, 2H), 7.15 – 7.11 (m, 2H), 7.04 (dd, *J* = 8.3, 1.0 Hz, 2H), 6.34 (dd, *J* = 7.3, 1.0 Hz, 2H), 5.88 (s, 2H), 3.11 – 3.02 (m, 2H), 1.24 (s, 6H), 1.22 (s, 6H), 0.89 – 0.88 (m, 1H). **^{13}C NMR** (126 MHz, $CDCl_3$) δ 143.3, 141.2, 141.1, 136.3, 135.3, 129.7, 127.6, 127.0, 125.9, 123.3, 120.3, 119.8, 117.5, 109.8, 105.7, 83.5, 31.8, 25.0, 24.6. **^{11}B NMR** (160 MHz, $CDCl_3$) δ 34.27, 33.51. **HRMS** (ESI, m/z) calcd for $C_{36}H_{36}B_2N_3O_2$ [M+H] $^+$: 564.2988; found: 564.2986.

2-(2-(naphthalen-2-yl)-1-(4,4,5,5-tetramethyl-1,3,2-dioxaborolan-2-yl)ethyl)-2,3-dihydro-1H-naphtho[1,8-de][1,3,2]diazaborinine (58)

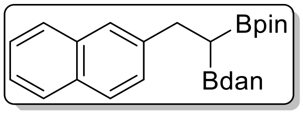

58

The reaction was performed following the general procedure A. The residue was purified by flash column chromatograph to give the product as a crystalline solid (42.6 mg, 52%). **^{1}H NMR** (500 MHz, $CDCl_3$) δ 7.81 (d, *J* = 7.6 Hz, 1H), 7.79 – 7.77 (m, 2H), 7.72 (s, 1H), 7.46 – 7.42 (m, 3H), 7.11 (t, *J* = 7.7 Hz, 2H), 7.03 (s, 2H), 6.30 (d, *J* = 7.3 Hz, 2H), 5.88 (s, 2H), 3.16 (dd, *J* = 14.2, 9.9 Hz, 1H), 3.07 (dd, *J* = 14.2, 6.2 Hz, 1H), 1.31 (d, *J* = 6.3 Hz, 1H), 1.18 (d, *J* = 9.7 Hz, 12H). **^{13}C NMR** (126 MHz, $CDCl_3$) δ 141.4, 141.2, 133.6, 127.9, 127.6, 127.5, 127.3, 126.0, 125.8, 125.1, 117.4, 117.3, 105.6, 83.4, 32.2, 29.7, 25.00, 24.8, 24.73, 24.71, 24.6, 24.5. **^{11}B NMR** (160 MHz, $CDCl_3$) δ 33.15, 32.27. **HRMS** (ESI, m/z) calcd for $C_{28}H_{31}B_2N_2O_2$ [M+H] $^+$: 449.2566; found: 449.2560.

(R)-2-(4-phenyl-1-(4,4,5,5-tetramethyl-1,3,2-dioxaborolan-2-yl)butyl)-2,3-dihydro-1H-naphtho[1,8-de][1,3,2]diazaborinine (59)

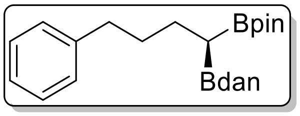

59

The reaction was performed following the general procedure C. The residue was purified by flash column chromatograph to give the product as a crystalline solid (66.5 mg, 78%). **^{1}H NMR** (500 MHz, $CDCl_3$) δ 7.31 – 7.27 (m, 2H), 7.21 – 7.18 (m, 3H), 7.11 – 7.07 (m, 2H), 6.99 (d, *J* = 8.3 Hz, 2H), 6.27 (d, *J* = 7.3 Hz, 2H), 5.76 (s, 2H), 2.65 (t, *J* = 6.9 Hz, 2H), 1.71 – 1.66 (m, 2H), 1.62 – 1.56 (m, 2H), 1.25 (d, *J* = 6.0 Hz, 12H), 0.78 (dd, *J* = 8.2, 6.0 Hz, 1H). **^{13}C NMR** (126 MHz, $CDCl_3$) δ 142.6, 141.3, 136.3, 128.4, 128.3, 127.56, 125.7 119.5, 117.3, 105.5, 83.2, 36.0, 34.0, 26.0, 25.1, 24.5. **^{11}B NMR** (160 MHz, $CDCl_3$) δ 34.18, 33.79. **HRMS** (ESI, m/z) calcd for $C_{26}H_{33}B_2N_2O_3$ [M+H] $^+$: 427.2723; found: 427.2726. **Specific rotation:** $[\alpha]^{25}_{D}$= -8.78° (c = 0.82, $CHCl_3$). **HPLC analysis**: HPLC DAICEL CHIRALCEL AD, hexane/isopropanol = 90/10, 0.5 mL/min, λ = 280 nm, t_R (minor) = 9.8 min, t_R (major) = 10.4 min, er = 94:6.

(R)-2-(3-phenyl-1-(4,4,5,5-tetramethyl-1,3,2-dioxaborolan-2-yl)propyl)-2,3-dihydro-1H-naphtho[1,8-de][1,3,2]diazaborinine (60)

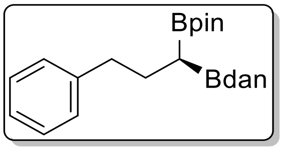

60

The reaction was performed following the general procedure C. The residue was purified by flash column chromatograph to give the product as a crystalline solid (62.6 mg, 76%). **^{1}H NMR** (500 MHz, $CDCl_3$) δ 7.32 – 7.27 (m, 2H), 7.21 (dd, *J* = 13.2, 7.0 Hz, 3H), 7.10 (t, *J* = 7.8 Hz, 2H), 7.00 (d, *J* = 8.2 Hz, 2H), 6.29 (d, *J* = 7.3 Hz, 2H), 5.80 (s, 2H), 2.78 – 2.70 (m, 1H), 2.64 – 2.60 (m, 1H), 2.03 – 1.94 (m, 1H), 1.88 – 1.83 (m, 1H), 1.28 (s, 6H), 1.27 (s, 6H), 0.84 (dd, *J* = 9.8, 5.6 Hz, 1H). **^{13}C NMR** (126 MHz, $CDCl_3$) δ 142.6, 141.3, 136.3, 128.5, 128.33 (s), 127.6, 125.8, 119.5, 117.3, 105.5, 83.3, 38.5, 28.6, 25.1, 24.5. **^{11}B NMR** (160 MHz, $CDCl_3$) δ 34.23, 34.02. **HRMS** (ESI, m/z) calcd for $C_{25}H_{31}B_2N_2O_2$ [M+H] $^+$: 413.2566; found: 413.2561. **Specific rotation:** $[\alpha]^{25}_{D}$= -4.08° (c = 0.76, $CHCl_3$). **HPLC analysis**: HPLC DAICEL CHIRALCEL OD, hexane/isopropanol = 99/1, 1.0 mL/min, λ = 280 nm, t_R (minor) = 14.5 min, t_R (major) = 16.4 min, er = 89:11.

(R)-2-(2-cyclohexyl-1-(4,4,5,5-tetramethyl-1,3,2-dioxaborolan-2-yl)ethyl)-2,3-dihydro-1H-naphtho[1,8-de][1,3,2]diazaborinine (61)

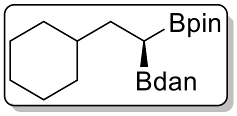

61

The reaction was performed following the general procedure C. The residue was purified by flash column chromatograph to give the product as a crystalline solid (56.6 mg, 70%). **^{1}H NMR** (500 MHz, $CDCl_3$) δ 7.12 – 7.07 (m, 2H), 7.00 (d, *J* = 8.2 Hz, 2H), 6.29 (d, *J* = 7.2 Hz, 2H), 5.80 (s, 2H), 1.75 – 1.63 (m, 8H), 1.56 (d, *J* = 4.3 Hz, 2H), 1.24 (s, 12H), 1.18 (dd, *J* = 18.8, 6.8 Hz, 3H), 0.89 (d, *J* = 11.3 Hz, 2H), 0.73 – 0.69 (m, 1H). **^{13}C NMR** (126 MHz, $CDCl_3$) δ 141.4, 136.3, 127.5, 119.5, 117.2, 105.4, 83.1, 40.3, 37.8, 33.5, 33.4, 33.3, 26.8, 26.4, 25.1, 24.9, 24.5, 24.5, 23.7. **^{11}B NMR** (160 MHz, $CDCl_3$) δ 34.26, 34.14. **HRMS** (ESI, m/z) calcd for $C_{24}H_{35}B_2N_2O_2$ $[M+H]^+$: 405.2879; found: 405.2873. **Specific rotation:** $[\alpha]^{25}_{D}$= -6.3° (c = 1.02, $CHCl_3$). **HPLC analysis**: HPLC DAICEL CHIRALCEL OD, hexane/isopropanol = 99/1, 0.5 mL/min, λ = 280 nm, t_R (major) = 20.4 min, t_R (minor) = 24.8 min, er = 97:3.

(R)-2-(2-cyclopentyl-1-(4,4,5,5-tetramethyl-1,3,2-dioxaborolan-2-yl)ethyl)-2,3-dihydro-1H-naphtho[1,8-de][1,3,2]diazaborinine (62)

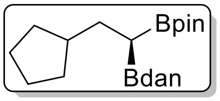

62

The reaction was performed following the general procedure C. The residue was purified by flash column chromatograph to give the product as a crystalline solid (52.3 mg, 67%). **^{1}H NMR** (500 MHz, $CDCl_3$) δ 7.09 (t, *J* = 7.8 Hz, 2H), 6.99 (d, *J* = 8.2 Hz, 2H), 6.29 (d, *J* = 7.3 Hz, 2H), 5.78 (s, 2H), 1.79 – 1.69 (m, 4H), 1.61 – 1.56 (m, 3H), 1.52 – 1.48 (m, 2H), 1.24 (d, *J* = 5.7 Hz, 12H), 1.09 (dt, *J* = 10.7, 3.3 Hz, 2H), 0.83 (dd, *J* = 9.1, 6.2 Hz, 1H). **^{13}C NMR** (126 MHz, $CDCl_3$) δ 141.4, 136.3, 127.5, 119.5, 117.2, 105.4, 83.2, 82.9, 42.4, 32.8, 32.6, 32.52, 32.50, 25.21, 25.18, 25.0, 24.8, 24.60, 24.57. **^{11}B NMR** (160 MHz, $CDCl_3$) δ 34.38, 33.90. **HRMS** (ESI, m/z) calcd for $C_{23}H_{32}B_2N_2NaO_2$ $[M+Na]^+$: 413.2542; found: 413.2534. **Specific rotation:** $[\alpha]^{25}_{D}$= -8.44° (c = 1.28, $CHCl_3$). **HPLC analysis**: HPLC DAICEL CHIRALCEL OD, hexane/isopropanol = 90/10, 1.0 mL/min, λ = 280 nm, t_R (major) = 6.8 min, t_R (minor) = 7.9 min, er = 86:14.

(R)-2-(2-cyclopropyl-1-(4,4,5,5-tetramethyl-1,3,2-dioxaborolan-2-yl)ethyl)-2,3-dihydro-1H-naphtho[1,8-de][1,3,2]diazaborinine (63)

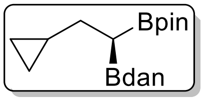

63

The reaction was performed following the general procedure C. The residue was purified by flash column chromatograph to give the product as a crystalline solid (48.5 mg, 67%). **^{1}H NMR** (500 MHz, $CDCl_3$) δ 7.08

(t, J = 7.8 Hz, 2H), 6.99 (d, J = 8.2 Hz, 2H), 6.28 (d, J = 7.3 Hz, 2H), 5.80 (s, 2H), 1.57 – 1.52 (m, 1H), 1.47 – 1.43 (m, 1H), 1.25 (d, J = 4.6 Hz, 12H), 0.90 (dd, J = 9.5, 6.1 Hz, 1H), 0.76 (dt, J = 12.3, 5.1 Hz, 1H), 0.41 (d, J = 7.9 Hz, 2H), 0.13 (dd, J = 8.8, 3.9 Hz, 1H), 0.07 – 0.04 (m, 1H). **^{13}C NMR** (126 MHz, $CDCl_3$) δ 141.3, 136.3, 127.5, 119.5, 117.2, 105.4, 83.2, 31.6, 25.0, 24.8, 24.6, 13.4, 5.1, 4.8. **^{11}B NMR** (160 MHz, $CDCl_3$) δ 34.58, 34.03. **HRMS** (ESI, m/z) calcd for $C_{21}H_{29}B_2N_2O_2$ [M+H] $^+$: 363.2410; found: 363.2401. **Specific rotation:** $[\alpha]^{25}_D$= -14.69° (c = 0.49, $CHCl_3$). **HPLC analysis**: HPLC DAICEL CHIRALCEL OD, hexane/isopropanol = 95/5, 0.5 mL/min, λ = 280 nm, t_R (major) = 26.8 min, t_R (minor) = 29.5 min, er = 93:7.

(R)-2-(3,3-dimethyl-1-(4,4,5,5-tetramethyl-1,3,2-dioxaborolan-2-yl)butyl)-2,3-dihydro-1H-naphtho[1,8-de][1,3,2]diazaborinine (64)

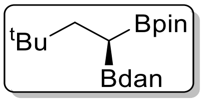

64

The reaction was performed following the general procedure C. The residue was purified by flash column chromatograph to give the product as a crystalline solid (42.4 mg, 56%). **^{1}H NMR** (500 MHz, $CDCl_3$) δ 7.11 – 7.06 (m, 2H), 7.00 (dd, J = 8.3, 1.8 Hz, 2H), 6.26 (d, J = 7.2 Hz, 2H), 5.60 (d, J = 18.6 Hz, 2H), 1.35 – 1.27 (m, 12H), 1.26 (s, 2H), 1.24 – 1.17 (m, 9H), 0.91 – 0.86 (m, 1H). **^{13}C NMR** (126 MHz, $CDCl_3$) δ 141.3, 127.5, 117.2, 105.4, 83.2, 82.9, 40.3, 39.23 31.8, 31.6, 29.3, 29.2, 24.9, 24.8, 24.7, 24.6. **^{11}B NMR** (160 MHz, $CDCl_3$) δ 34.35, 34.06. **HRMS** (ESI, m/z) calcd for $C_{22}H_{33}B_2N_2O_2$ [M+H] $^+$: 379.2723; found: 379.2723. **Specific rotation:** $[\alpha]^{25}_D$= -15.45° (c = 0.44, $CHCl_3$). **HPLC analysis**: HPLC DAICEL CHIRALCEL OD, hexane/isopropanol = 99.0/1.0, 1.0 mL/min, λ = 280 nm, t_R (major) = 5.8 min, t_R (minor) = 7.5 min, er = 85:15.

(R)-2-(1-(4,4,5,5-tetramethyl-1,3,2-dioxaborolan-2-yl)pentyl)-2,3-dihydro-1H-naphtho[1,8-de][1,3,2]diazaborinine (65)

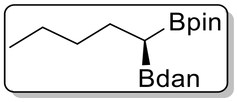

65

The reaction was performed following the general procedure C. The residue was purified by flash column chromatograph to give the product as a crystalline solid (49.5 mg, 68%). **^{1}H NMR** (500 MHz, $CDCl_3$) δ 7.09 (t, J = 7.8 Hz, 2H), 6.99 (d, J = 8.2 Hz, 2H), 6.29 (d, J = 7.3 Hz, 2H), 5.80 (s, 2H), 1.67 – 1.59 (m, 1H), 1.54 (dd, J = 13.9, 7.4 Hz, 1H), 1.37 – 1.31 (m, 4H), 1.25 (d, J = 6.1 Hz, 12H), 0.91 (t, J = 6.9 Hz, 3H), 0.75 (dd, J = 9.3, 6.3 Hz, 1H). **^{13}C NMR** (126 MHz, $CDCl_3$) δ 141.4, 136.3, 127.5, 119.5, 117.2, 105.4, 83.1, 34.9, 34.6, 26.1, 25.0, 24.9, 24.5, 24.5, 22.8, 14.1. **^{11}B NMR** (160 MHz, $CDCl_3$) δ 34.66, 34.34. **HRMS** (ESI, m/z) calcd for $C_{21}H_{31}B_2N_2O_2$ [M+H] $^+$: 365.2566; found: 365.2571. **Specific rotation:** $[\alpha]^{25}_D$= -11.27° (c = 0.55, $CHCl_3$). **HPLC analysis**: HPLC DAICEL CHIRALCEL AD, hexane/isopropanol = 99.5/0.5, 1.4 mL/min, λ = 280 nm, t_R (minor) = 5.2 min, t_R (major) = 6.3 min, er = 93:7.

(R)-2-(1-(4,4,5,5-tetramethyl-1,3,2-dioxaborolan-2-yl)hexyl)-2,3-dihydro-1H-naphtho[1,8-de][1,3,2]diazaborinine (66)

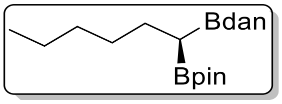

66

The reaction was performed following the general procedure C. The residue was purified by flash column chromatograph to give the product as a crystalline solid (54.4 mg, 72%). **^{1}H NMR** (500 MHz, $CDCl_3$) δ 7.10 (dd, *J* = 8.3, 7.3 Hz, 2H), 7.00 (dd, *J* = 8.3, 1.0 Hz, 2H), 6.30 (dd, *J* = 7.3, 1.0 Hz, 2H), 5.81 (s, 2H), 1.66 – 1.61 (m, 1H), 1.55 – 1.51 (m, 1H), 1.37 – 1.30 (m, 6H), 1.26 (d, *J* = 6.3 Hz, 12H), 0.93 – 0.89 (m, 3H), 0.76 (dd, *J* = 9.5, 6.0 Hz, 1H). **^{13}C NMR** (126 MHz, $CDCl_3$) δ 141.4, 136.3, 127.6, 119.5, 117.2, 105.4, 83.2, 32.1, 31.9, 26.4, 25.0, 24.5, 22.6, 14.1. **^{11}B NMR** (160 MHz, $CDCl_3$) δ 34.80, 33.92. **HRMS** (ESI, m/z) calcd for $C_{22}H_{32}B_2N_2NaO_2$ $[M+Na]^+$: 401.2542; found: 401.2540. **Specific rotation:** $[\alpha]^{25}_{D}$= -7.72° (c = 1.32, $CHCl_3$). **HPLC analysis**: HPLC DAICEL CHIRALCEL AD, hexane/isopropanol = 99.5/0.5, 1.4 mL/min, λ = 280 nm, t_R (minor) = 6.2 min, t_R (major) = 7.0 min, er = 94:6.

(R)-2-(1-(4,4,5,5-tetramethyl-1,3,2-dioxaborolan-2-yl)heptyl)-2,3-dihydro-1H-naphtho[1,8-de][1,3,2]diazaborinine (67)

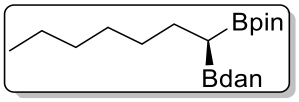

67

The reaction was performed following the general procedure C. The residue was purified by flash column chromatograph to give the product as a crystalline solid (58.1 mg, 74%). **^{1}H NMR** (500 MHz, $CDCl_3$) δ 7.10 – 7.07 (m, 2H), 6.98 (d, *J* = 7.3 Hz, 2H), 6.28 (d, *J* = 7.3 Hz, 2H), 5.78 (s, 2H), 1.54 (d, *J* = 7.3 Hz, 2H), 1.24 (s, 8H), 1.23 (s, 6H), 1.22 (s, 6H), 0.87 (d, *J* = 3.2 Hz, 3H), 0.74 – 0.72 (m, 1H). **^{13}C NMR** (126 MHz, $CDCl_3$) δ 141.4, 136.3, 127.5, 119.5, 117.2, 105.4, 83.1, 82.9, 32.6, 32.4, 31.8, 29.4, 29.3, 26.4, 25.7, 25.0, 24.9, 24.53, 24.50, 22.7, 22.6, 14.1**^{11}B NMR** (160 MHz, $CDCl_3$) δ 34.88, 34.11. **HRMS** (ESI, m/z) calcd for $C_{23}H_{35}B_2N_2O_2$ $[M+H]^+$: 393.2879; found: 393.2874. **Specific rotation:** $[\alpha]^{25}_{D}$= -10.7° (c = 1.43, $CHCl_3$). **HPLC analysis**: HPLC DAICEL CHIRALCEL AD, hexane/isopropanol = 99.5/0.5, 0.5 mL/min, λ = 280 nm, t_R (minor) = 12.5 min, t_R (major) = 12.9 min, er = 94:6.

(R)-2-(1-(4,4,5,5-tetramethyl-1,3,2-dioxaborolan-2-yl)octyl)-2,3-dihydro-1H-naphtho[1,8-de][1,3,2]diazaborinine (68)

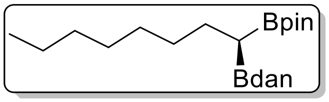

68

The reaction was performed following the general procedure C. The residue was purified by flash column chromatograph to give the product as a crystalline solid (63.4 mg, 78%). **^{1}H NMR** (500 MHz, $CDCl_3$) δ 7.10 – 7.06 (m, 2H), 6.98 (d, *J* = 8.2 Hz, 2H), 6.28 (d, *J* = 7.3 Hz, 2H), 5.79 (s, 2H), 1.57 – 1.41 (m, 2H), 1.27 (dd,

J = 7.6, 2.5 Hz, 10H), 1.25 (s, 6H), 1.24 (s, 6H), 0.88 (t, J = 6.8 Hz, 3H), 0.75 – 0.72 (m, 1H). **^{13}C NMR** (126 MHz, $CDCl_3$) δ 141.4, 136.3, 127.5, 119.5, 117.2, 105.4, 83.1, 32.4, 31.9, 29.7, 29.2, 26.4, 25.0, 24.9, 24.5, 22.7, 14.1. **^{11}B NMR** (160 MHz, $CDCl_3$) δ 34.23, 33.23. **HRMS** (ESI, m/z) calcd for $C_{24}H_{36}B_2N_2NaO_2$ $[M+Na]^+$: 429.2855; found: 429.2859. **Specific rotation:** $[\alpha]^{25}_D$= -8.29° (c = 0.70, $CHCl_3$). **HPLC analysis**: HPLC DAICEL CHIRALCEL AD, hexane/isopropanol = 99.5/0.5, 0.5 mL/min, λ = 280 nm, t_R (minor) = 15.9 min, t_R (major) = 16.3 min, er = 92:8.

(R)-2-(1-(4,4,5,5-tetramethyl-1,3,2-dioxaborolan-2-yl)octyl)-2,3-dihydro-1H-naphtho[1,8-de][1,3,2]diazaborinine (69)

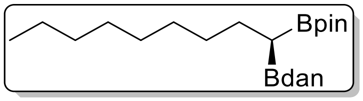

69

The reaction was performed following the general procedure C. The residue was purified by flash column chromatograph to give the product as a crystalline solid (58.8 mg, 70%). **^{1}H NMR** (500 MHz, $CDCl_3$) δ 7.10 (t, J = 7.8 Hz, 2H), 7.00 (d, J = 8.2 Hz, 2H), 6.30 (d, J = 7.3 Hz, 2H), 5.81 (s, 2H), 1.67 – 1.61 (m, 1H), 1.55 – 1.50 (m, 1H), 1.34 – 1.27 (m, 12H), 1.26 (d, J = 6.2 Hz, 12H), 0.90 (t, J = 6.8 Hz, 3H), 0.76 (dd, J = 9.4, 6.0 Hz, 1H). **^{13}C NMR** (126 MHz, $CDCl_3$) δ 141.4, 136.3, 127.6, 119.5, 117.2, 105.4, 83.2, 32.4, 31.9, 29.7, 29.5, 29.3, 26.4, 25.0, 24.5, 22.7, 14.1. **^{11}B NMR** (160 MHz, $CDCl_3$) δ 34.45, 34.19. **HRMS** (ESI, m/z) calcd for $C_{25}H_{39}B_2N_2O_2$ $[M+H]^+$: 421.3192; found: 421.3195. **Specific rotation:** $[\alpha]^{25}_D$= -21.97° (c = 1.22, $CHCl_3$). **HPLC analysis**: HPLC DAICEL CHIRALCEL AD, hexane/isopropanol = 99.5/0.5, 0.5 mL/min, λ = 280 nm, t_R (minor) = 14.4 min, t_R (major) = 14.9 min, er = 96:4.

(R)-2-(5-methyl-1-(4,4,5,5-tetramethyl-1,3,2-dioxaborolan-2-yl)hexyl)-2,3-dihydro-1H-naphtho[1,8-de][1,3,2]diazaborinine (70)

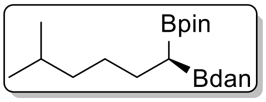

70

The reaction was performed following the general procedure C. The residue was purified by flash column chromatograph to give the product as a crystalline solid (52.3 mg, 67%). **^{1}H NMR** (500 MHz, $CDCl_3$) δ 7.09 (t, J = 7.8 Hz, 2H), 6.99 (d, J = 8.2 Hz, 2H), 6.29 (d, J = 7.2 Hz, 2H), 5.80 (s, 2H), 1.64 – 1.60 (m, 1H), 1.53 (td, J = 8.1, 7.4, 4.5 Hz, 2H), 1.37 (qd, J = 8.3, 5.5 Hz, 4H), 1.26 (s, 3H), 1.25 (s, 5H), 1.24 (s, 4H), 1.23 (s, 3H), 0.88 (d, J = 6.6 Hz, 3H), 0.85 (d, J = 6.5 Hz, 3H), 0.76 (dt, J = 10.1, 5.2 Hz, 1H). **^{13}C NMR** (126 MHz, $CDCl_3$) δ 141.4, 136.3, 127.5, 119.5, 117.2, 105.4, 83.1, 39.1, 30.1, 27.8, 26.6, 25.0, 24.9, 24.5, 24.5, 22.7, 22.6. **^{11}B NMR** (160 MHz, $CDCl_3$) δ 34.33, 33.33. **HRMS** (ESI, m/z) calcd for $C_{23}H_{34}B_2N_2NaO_2$ $[M+Na]^+$: 415.2699; found: 415.2699. **Specific rotation:** $[\alpha]^{25}_D$= -4.04° (c = 0.99, $CHCl_3$). **HPLC analysis**: HPLC DAICEL CHIRALCEL AD, hexane/isopropanol = 99.4/0.6, 1.4 mL/min, λ = 280 nm, t_R (minor) = 5.0 min, t_R (major) = 5.2 min, er = 87:13.

(R)-tert-butyl 4-(2-(1H-naphtho[1,8-de][1,3,2]diazaborinin-2(3H)-yl)-2-(4,4,5,5-tetramethyl-1,3,2-

dioxaborolan-2-yl)ethyl)piperidine-1-carboxylate (71)

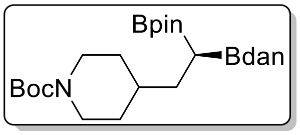

71

The reaction was performed following the general procedure C. The residue was purified by flash column chromatograph to give the product as a crystalline solid (72.8 mg, 72%). **^{1}H NMR** (500 MHz, $CDCl_3$) δ 7.13 – 7.07 (m, 2H), 7.04 – 6.96 (m, 2H), 6.33 – 6.27 (m, 2H), 5.76 (s, 2H), 4.08 (s, 4H), 2.65 (s, 4H), 1.45 (d, *J* = 3.5 Hz, 9H), 1.24 (s, 12H), 1.06 (d, *J* = 12.2 Hz, 2H), 0.85 (s, 1H), 0.81 – 0.71 (m, 1H). **^{13}C NMR** (126 MHz, $CDCl_3$) δ 155.2, 141.2, 136.3, 117.4, 105.5, 83.3, 79.3, 38.0, 33.0, 28.5, 25.0, 24.8, 24.5. **^{11}B NMR** (160 MHz, $CDCl_3$) δ 33.92, 33.33. **HRMS** (ESI, m/z) calcd for $C_{21}H_{31}B_2N_2O_2$ $[M+H]^+$: 365.2566; found: 365.2571. **Specific rotation:** $[\alpha]^{25}_D$= -5.32° (c = 1.09, $CHCl_3$). **HPLC analysis**: HPLC DAICEL CHIRALCEL AD, hexane/isopropanol = 90/10, 0.5 mL/min, λ = 280 nm, t_R (minor) = 14.5 min, t_R (major) = 15.6 min, er = 94:6.

(R)-2-(4-((tert-butyldimethylsilyl)oxy)-1-(4,4,5,5-tetramethyl-1,3,2-dioxaborolan-2-yl)butyl)-2,3-dihydro-1H-naphtho[1,8-de][1,3,2]diazaborinine (72)

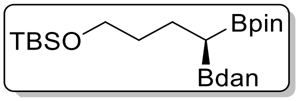

72

The reaction was performed following the general procedure C. The residue was purified by flash column chromatograph to give the product as a crystalline solid (60.5 mg, 63%). **^{1}H NMR (**500 MHz, $CDCl_3$) δ 7.11 (dd, *J* = 8.2, 7.3 Hz, 2H), 7.01 (dd, *J* = 8.3, 1.0 Hz, 2H), 6.30 (dd, *J* = 7.4, 1.0 Hz, 2H), 5.86 (s, 2H), 3.68 – 3.64 (m, 2H), 1.66 (dd, *J* = 13.6, 5.3 Hz, 2H), 1.63 – 1.56 (m, 2H), 1.26 (d, *J* = 5.3 Hz, 12H), 0.94 (s, 9H), 0.78 (dd, *J* = 8.7, 5.6 Hz, 1H), 0.09 (d, *J* = 1.0 Hz, 6H). **^{13}C NMR** (126 MHz, $CDCl_3$) δ 141.3, 136.3, 127.5, 119.5, 117.2, 105.4, 83.2, 82.9, 63.2, 35.4, 25.1, 24.9, 24.54, 24.52, 22.4, 18.4, -5.2. **^{11}B NMR** (160 MHz, $CDCl_3$) δ 34.08, 33.91. **HRMS** (ESI, m/z) calcd for $C_{26}H_{43}B_2N_2O_3Si$ $[M+H]^+$: 481.3224; found: 481.3221. **Specific rotation:** $[\alpha]^{25}_D$= -2.30° (c = 0.87, $CHCl_3$). **HPLC analysis**: HPLC DAICEL CHIRALCEL OD, hexane/isopropanol = 95/5, 1.0 mL/min, λ = 280 nm, t_R (minor) = 3.9 min, t_R (major) = 4.3 min, er = 90.5:9.5.

(R)-2-(6-((tert-butyldimethylsilyl)oxy)-1-(4,4,5,5-tetramethyl-1,3,2-dioxaborolan-2-yl)hexyl)-2,3-dihydro-1H-naphtho[1,8-de][1,3,2]diazaborinine (73)

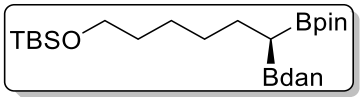

73

The reaction was performed following the general procedure C. The residue was purified by flash column chromatograph to give the product as a crystalline solid (70.2 mg, 69%). **^{1}H NMR** (500 MHz, $CDCl_3$) δ 7.08 (t, *J* = 7.8 Hz, 2H), 6.98 (d, *J* = 8.2 Hz, 2H), 6.28 (d, *J* = 7.3 Hz, 2H), 5.79 (s, 2H), 3.59 (d, *J* = 6.6 Hz, 2H), 1.55 – 1.48 (m, 4H), 1.35 (dd, *J* = 12.2, 6.6 Hz, 4H), 1.25 (s, 6H), 1.23 (s, 6H), 0.89 (s, 9H), 0.75 – 0.71 (m,

1H), 0.04 (s, 6H). **^{13}C NMR** (126 MHz, $CDCl_3$) δ 141.3, 136.3, 127.5, 119.5, 117.2, 105.4, 83.2, 63.3, 32.8, 32.2, 26.4, 26.0, 25.9, 25.0, 24.9, 24.5, 24.52,24.50, 18.4, -5.2. **^{11}B NMR** (160 MHz, $CDCl_3$) δ 33.92, 33.51. **HRMS** (ESI, m/z) calcd for $C_{28}H_{47}B_2N_2O_3Si$ [M+H] $^+$: 509.3537; found: 509.3530. **Specific rotation:** $[\alpha]^{25}_D$= -6.25° (c = 0.64, $CHCl_3$). **HPLC analysis**: HPLC DAICEL CHIRALCEL OD, hexane/isopropanol = 99/1, 0.3 mL/min, λ = 280 nm, t_R (minor) = 26.5 min, t_R (major) = 29.3 min, er = 94:6.

(R)-2-(5-((tert-butyldiphenylsilyl)oxy)-1-(4,4,5,5-tetramethyl-1,3,2-dioxaborolan-2-yl)pentyl)-2,3-dihydro-1H-naphtho[1,8-de][1,3,2]diazaborinine (74)

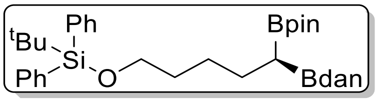

74

The reaction was performed following the general procedure C. The residue was purified by flash column chromatograph to give the product as a crystalline solid (91.5 mg, 74%). **^{1}H NMR** (500 MHz, $CDCl_3$) δ 7.70 – 7.67 (m, 4H), 7.40 (ddt, *J* = 12.1, 6.6, 3.5 Hz, 6H), 7.10 (td, *J* = 7.8, 2.2 Hz, 2H), 7.01 (dd, *J* = 8.4, 2.4 Hz, 2H), 6.29 (dd, *J* = 7.4, 2.2 Hz, 2H), 5.79 (d, *J* = 3.2 Hz, 2H), 3.71 – 3.67 (m, 2H), 1.62 (dd, *J* = 7.7, 3.6 Hz, 2H), 1.51 (h, *J* = 4.0, 3.4 Hz, 2H), 1.42 – 1.39 (m, 1H), 1.31 (d, *J* = 2.8 Hz, 1H), 1.25 (dd, *J* = 8.3, 2.4 Hz, 12H), 1.06 (d, *J* = 3.5 Hz, 9H), 0.75 (dd, *J* = 8.1, 3.8 Hz, 1H). **^{13}C NMR** (126 MHz, $CDCl_3$) δ 141.4, 136.3, 135.6, 134.2, 129.5, 127.6, 127.6, 119.5, 117.2, 105.5, 83.2, 64.0, 32.8, 28.7, 26.9, 26.3, 25.0, 24.6, 19.2. **^{11}B NMR** (160 MHz, $CDCl_3$) δ 34.42, 34.10. **HRMS** (ESI, m/z) calcd for $C_{37}H_{48}B_2KN_2O_3Si$ [M+K] $^+$: 657.3252; found: 657.3262. **Specific rotation:** $[\alpha]^{25}_D$= -4.97° (c = 1.45, $CHCl_3$). **HPLC analysis**: HPLC DAICEL CHIRALCEL OD, hexane/isopropanol = 99/1, 1.0 mL/min, λ = 250 nm, t_R (minor) = 22.1 min, t_R (major) = 26.8 min, er = 90:10.

(R)-2-(1-(4,4,5,5-tetramethyl-1,3,2-dioxaborolan-2-yl)-5-((triisopropylsilyl)oxy)pentyl)-2,3-dihydro-1H-naphtho[1,8-de][1,3,2]diazaborinine (75)

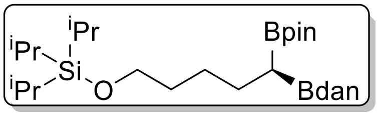

75

The reaction was performed following the general procedure C. The residue was purified by flash column chromatograph to give the product as a crystalline solid (74.0 mg, 69%). **^{1}H NMR** (500 MHz, $CDCl_3$) δ 7.12 – 7.07 (m, 2H), 7.00 (dd, *J* = 8.3, 0.9 Hz, 2H), 6.30 (dd, *J* = 7.2, 1.0 Hz, 2H), 5.81 (s, 2H), 3.70 (t, *J* = 6.5 Hz, 2H), 1.72 – 1.64 (m, 1H), 1.62 – 1.55 (m, 3H), 1.51 – 1.45 (m, 1H), 1.26 (d, *J* = 5.5 Hz, 12H), 1.24 (s, 2H), 1.13 – 1.10 (m, 2H), 1.08 (s, 13H), 0.78 (dd, *J* = 9.5, 5.8 Hz, 1H). **^{13}C NMR** (126 MHz, $CDCl_3$) δ 141.4, 136.3, 127.5, 119.5, 117.2, 105.5, 83.2, 63.5, 33.3, 26.4, 25.0, 24.5, 18.1, 12.1. **^{11}B NMR** (160 MHz, $CDCl_3$) δ 34.32, 33.75. **HRMS** (ESI, m/z) calcd for $C_{30}H_{50}B_2KN_2O_3Si$ [M+K] $^+$:575.3408; found: 575.3411. **Specific rotation:** $[\alpha]^{25}_D$= -7.84° (c =1.16, $CHCl_3$). **HPLC analysis**: HPLC DAICEL CHIRALCEL AD, hexane/isopropanol = 99.7/0.3, 0.5 mL/min, λ = 280 nm, t_R (minor) = 19.0 min, t_R (major) = 21.1 min, er = 92:8.

(R)-5-(1H-naphtho[1,8-de][1,3,2]diazaborinin-2(3H)-yl)-5-(4,4,5,5-tetramethyl-1,3,2-dioxaborolan-2-yl)pentyl propionate (76)

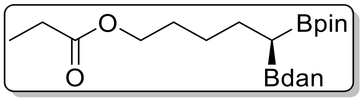

76

The reaction was performed following the general procedure C. The residue was purified by flash column chromatograph to give the product as a crystalline solid (56.7 mg, 65%). **^{1}H NMR** (500 MHz, $CDCl_3$) δ 7.11 – 7.06 (m, 2H), 6.99 (d, *J* = 8.2 Hz, 2H), 6.29 (d, *J* = 7.3 Hz, 2H), 5.79 (s, 2H), 4.06 (dt, *J* = 9.5, 5.2 Hz, 2H), 2.33 – 2.31 (m, 1H), 2.31 – 2.28 (m, 1H), 1.61 – 1.51 (m, 2H), 1.48 – 1.37 (m, 2H), 1.36 – 1.26 (m, 2H), 1.25 (s, 6H), 1.23 (s, 6H), 1.12 (t, *J* = 7.6 Hz, 3H), 0.75 (dd, *J* = 9.3, 6.1 Hz, 1H). **^{13}C NMR** (126 MHz, $CDCl_3$) δ 174.6, 141.3, 136.3, 127.5, 119.5, 117.3, 105.5, 83.2, 64.5, 64.3, 28.6, 28.55, 27.6, 26.0, 25.0, 24.9, 24.5, 9.2. **^{11}B NMR** (160 MHz, $CDCl_3$) δ 34.34, 34.09. **HRMS** (ESI, m/z) calcd for $C_{24}H_{35}B_2N_2O_4$ $[M+H]^+$: 437.2777; found: 437.2774. **Specific rotation:** $[\alpha]^{25}_D$= -11.62° (c = 0.37, $CHCl_3$). **HPLC analysis**: HPLC DAICEL CHIRALCEL OD, hexane/isopropanol = 97/3, 0.5 mL/min, λ = 280 nm, t_R (major) =23.0 min, t_R (minor) = 26.1 min, er = 90:10.

(R)-5-(1H-naphtho[1,8-de][1,3,2]diazaborinin-2(3H)-yl)-5-(4,4,5,5-tetramethyl-1,3,2-dioxaborolan-2-yl)pentyl (2E,4E)-hexa-2,4-dienoate (77)

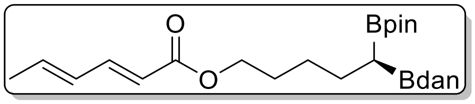

77

The reaction was performed following the general procedure C. The residue was purified by flash column chromatograph to give the product as a crystalline solid (43.6 mg, 46%). **^{1}H NMR** (500 MHz, $CDCl_3$) δ 7.08 (dd, *J* = 8.3, 7.3 Hz, 2H), 6.99 (dd, *J* = 8.3, 1.0 Hz, 2H), 6.29 (dd, *J* = 7.3, 1.0 Hz, 2H), 5.79 (s, 2H), 5.60 – 5.27 (m, 4H), 4.07 (dt, *J* = 6.6, 3.4 Hz, 2H), 2.35 – 2.32 (m, 2H), 2.30 – 2.27 (m, 2H), 1.63 (d, *J* = 1.2 Hz, 3H), 1.45 – 1.36 (m, 2H), 1.26 (s, 3H), 1.25 (s, 6H), 1.23 (s, 3H), 0.74 (dd, *J* = 9.3, 6.2 Hz, 1H). **^{13}C NMR** (126 MHz, $CDCl_3$) δ 173.4, 141.3, 136.3, 129.2, 127.6, 126.1, 117.3, 105.5, 83.2, 64.3, 34.4, 28.8, 28.6, 27.9, 26.0, 25.0, 24.8, 24.5, 17.9. **^{11}B NMR** (160 MHz, $CDCl_3$) δ 34.45, 33.68. **HRMS** (ESI, m/z) calcd for $C_{27}H_{37}B_2N_2O_4$ $[M+H]^+$: 475.2934; found: 475.2929. **Specific rotation:** $[\alpha]^{25}_D$= -1.24° (c = 0.86, $CHCl_3$). **HPLC analysis**: HPLC DAICEL CHIRALCEL AD, hexane/isopropanol = 90/10, 0.5 mL/min, λ = 280 nm, t_R (major) = 14.4 min, t_R (minor) = 15.7 min, er = 88:12.

(R)-5-(1H-naphtho[1,8-de][1,3,2]diazaborinin-2(3H)-yl)-5-(4,4,5,5-tetramethyl-1,3,2-dioxaborolan-2-yl)pentyl 2-propylpentanoate (78)

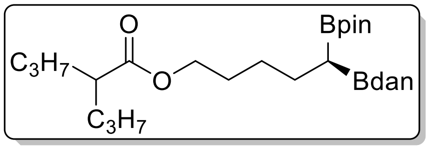

78

The reaction was performed following the general procedure C. The residue was purified by flash column chromatograph to give the product as a crystalline solid (64.8 mg, 64%). **^{1}H NMR** (500 MHz, $CDCl_3$) δ 7.08 (t, *J* = 7.8 Hz, 2H), 6.98 (d, *J* = 8.2 Hz, 2H), 6.29 (d, *J* = 7.3 Hz, 2H), 5.79 (s, 2H), 4.07 – 4.03 (m, 2H), 2.35 – 2.32 (m, 1H), 1.66 (d, *J* = 7.1 Hz, 2H), 1.57 (dd, *J* = 10.5, 4.9 Hz, 4H), 1.41 – 1.36 (m, 4H), 1.27 (d, *J* = 7.7 Hz, 4H), 1.24 (s, 3H), 1.23 (d, *J* = 2.4 Hz, 6H), 1.22 (s, 3H), 0.88 (d, *J* = 5.4 Hz, 6H), 0.75 – 0.72 (m, 1H). **^{13}C NMR** (126 MHz, $CDCl_3$) δ 176.7, 141.3, 136.3, 127.5, 119.5, 117.3, 105.5, 83.2, 83.0, 64.0, 45.4, 34.7, 28.9, 28.8, 28.6, 26.1, 25.0, 24.9, 24.5, 20.6, 14.0. **^{11}B NMR** (160 MHz, $CDCl_3$) δ 34.80, 34.45. **HRMS** (ESI, m/z) calcd for $C_{29}H_{45}B_2N_2O_4$ [M+H] $^+$: 507.3560; found: 507.3559. **Specific rotation:** $[\alpha]^{25}_D$= 4.57° (c = 0.46, $CHCl_3$). **HPLC analysis**: HPLC DAICEL CHIRALCEL OD, hexane/isopropanol = 97/3, 0.5 mL/min, λ = 280 nm, t_R (major) = 12.8 min, t_R (minor) = 14.3 min, er = 89:11.

(R)-5-(1H-naphtho[1,8-de][1,3,2]diazaborinin-2(3H)-yl)-5-(4,4,5,5-tetramethyl-1,3,2-dioxaborolan-2-yl)pentyl benzoate (79)

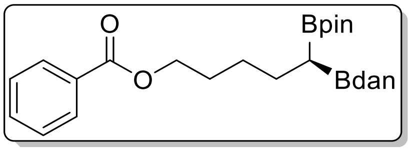

79

The reaction was performed following the general procedure C. The residue was purified by flash column chromatograph to give the product as a crystalline solid (66.8 mg, 69%). **^{1}H NMR** (500 MHz, $CDCl_3$) δ 8.06 (d, *J* = 1.4 Hz, 1H), 8.05 (d, *J* = 1.7 Hz, 1H), 7.55 – 7.52 (m, 1H), 7.41 (t, *J* = 7.8 Hz, 2H), 7.10 (t, *J* = 7.8 Hz, 2H), 7.01 (d, *J* = 8.3 Hz, 2H), 6.30 (d, *J* = 7.3 Hz, 2H), 5.84 (s, 2H), 4.35 (t, *J* = 6.6 Hz, 2H), 1.82 (t, *J* = 7.0 Hz, 2H), 1.67 – 1.47 (m, 4H), 1.25 (s, 6H), 1.23 (s, 6H), 0.80 (dd, *J* = 9.4, 5.7 Hz, 1H). **^{13}C NMR** (126 MHz, $CDCl_3$) δ 166.7, 141.3, 136.3, 132.8, 130.5, 129.6, 128.3, 127.6, 119.6, 117.3, 105.5, 83.2, 64.9, 28.9, 28.7, 26.1, 25.0, 24.87, 24.54, 24.51. **^{11}B NMR** (160 MHz, $CDCl_3$) δ 33.92, 32.98. **HRMS** (ESI, m/z) calcd for $C_{28}H_{35}B_2N_2O_4$ [M+H] $^+$:485.2777; found: 485.2773. **Specific rotation:** $[\alpha]^{25}_D$= -5.6° (c = 0.75, $CHCl_3$). **HPLC analysis**: HPLC DAICEL CHIRALCEL OD, hexane/isopropanol = 95/5 1.0 mL/min, λ = 250 nm, t_R (minor) = 18.4 min, t_R (major) = 29.4 min, er = 90:10.

(R)-5-(1H-naphtho[1,8-de][1,3,2]diazaborinin-2(3H)-yl)-5-(4,4,5,5-tetramethyl-1,3,2-dioxaborolan-2-yl)pentyl 4-methylbenzoate (80)

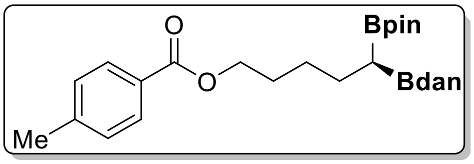

80

The reaction was performed following the general procedure C. The residue was purified by flash column chromatograph to give the product as a crystalline solid (67.9 mg, 66%). **^{1}H NMR** (500 MHz, $CDCl_3$) δ 7.93 (s, 1H), 7.92 (d, *J* = 1.8 Hz, 1H), 7.20 (s, 1H), 7.18 (s, 1H), 7.11 – 7.07 (m, 2H), 6.99 (d, *J* = 8.1 Hz, 2H), 6.28 (d, *J* = 7.3 Hz, 2H), 5.81 (s, 2H), 4.32 (t, *J* = 6.5 Hz, 2H), 2.39 (s, 3H), 1.82 – 1.78 (m, 2H), 1.67 – 1.59 (m, 2H), 1.56 – 1.49 (m, 2H), 1.24 (s, 3H), 1.23 (s, 3H), 1.23 (s, 3H), 1.22 (s, 3H), 0.80 – 0.76 (m, 1H). **^{13}C NMR** (126 MHz, $CDCl_3$) δ 166.8, 143.4, 141.3, 129.6, 129.0, 127.5, 117.3, 105.5, 83.2, 64.7, 28.9, 28.7, 26.1, 25.0, 24.9, 24.53, 24.50, 21.6. **^{11}B NMR** (160 MHz, $CDCl_3$) δ 34.97, 34.27. **HRMS** (ESI, m/z) calcd for $C_{29}H_{37}B_2N_2O_4$ [M+H] $^+$:499.2934; found: 499.2932. **Specific rotation:** $[\alpha]^{25}_D$= -7.65° (c = 0.51, $CHCl_3$). **HPLC analysis**: HPLC DAICEL CHIRALCEL OD, hexane/isopropanol = 95/5 1.0 mL/min, λ = 280 nm, t_R (minor) = 18.9 min, t_R (major) = 21.8 min, er = 88:12.

(R)-5-(1H-naphtho[1,8-de][1,3,2]diazaborinin-2(3H)-yl)-5-(4,4,5,5-tetramethyl-1,3,2-dioxaborolan-2-yl)pentyl 4-cyclohexylbenzoate (81)

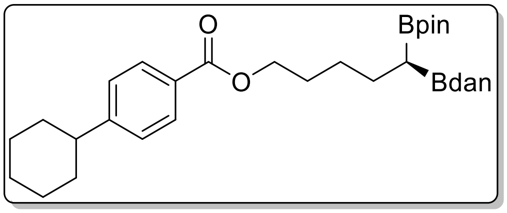

81

The reaction was performed following the general procedure C. The residue was purified by flash column chromatograph to give the product as a crystalline solid (74.8 mg, 66%). **^{1}H NMR** (500 MHz, $CDCl_3$) δ 7.96 (s, 1H), 7.95 (s, 1H), 7.22 (d, *J* = 8.3 Hz, 2H), 7.11 – 7.07 (m, 2H), 7.00 (d, *J* = 8.2 Hz, 2H), 6.29 (d, *J* = 7.3 Hz, 2H), 5.82 (s, 2H), 4.32 (t, *J* = 6.5 Hz, 2H), 2.56 – 2.52 (m, 1H), 1.87 – 1.85 (m, 4H), 1.79 (d, *J* = 6.9 Hz, 2H), 1.76 (t, *J* = 3.7 Hz, 2H), 1.66 – 1.58 (m, 2H), 1.57 – 1.50 (m, 2H), 1.40 (dd, *J* = 8.5, 3.7 Hz, 4H), 1.24 (s, 6H), 1.22 (s, 6H), 0.79 (ddd, *J* = 9.5, 6.2, 2.8 Hz, 1H). **^{13}C NMR** (126 MHz, $CDCl_3$) δ 166.8, 141.3, 129.7, 127.6, 126.8, 117.3, 105.5, 83.2, 64.7, 44.7, 34.2, 28.9, 28.8, 26.8, 26.1, 25.0, 24.9, 24.5, 24.5. **^{11}B NMR** (160 MHz, $CDCl_3$) δ 34.71, 33.02. **HRMS** (ESI, m/z) calcd for $C_{34}H_{45}B_2N_2O_4$ [M+H] $^+$:567.3560; found: 567.3560. **Specific rotation:** $[\alpha]^{25}_D$= -4.66° (c = 0.88, $CHCl_3$). **HPLC analysis**: HPLC DAICEL CHIRALCEL OD, hexane/isopropanol = 95/5, 1.0 mL/min, λ = 280 nm, t_R (major) = 9.2 min, t_R (minor) = 11.3 min, er = 85:15.

(R)-5-(1H-naphtho[1,8-de][1,3,2]diazaborinin-2(3H)-yl)-5-(4,4,5,5-tetramethyl-1,3,2-dioxaborolan-2-yl)pentyl (E)-2-methyl-3-phenylacrylate (82)

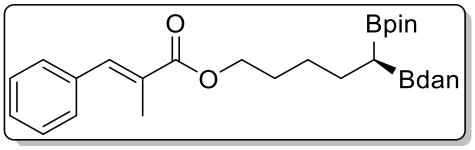

82

The reaction was performed following the general procedure C. The residue was purified by flash column chromatograph to give the product as a crystalline solid (51.4 mg, 49%). **^{1}H NMR** (500 MHz, $CDCl_3$) δ 7.69

(s, 1H), 7.41 – 7.37 (m, 4H), 7.32 (q, J = 5.2, 4.4 Hz, 1H), 7.08 (t, J = 7.7 Hz, 2H), 7.00 (d, J = 8.3 Hz, 2H), 6.29 (d, J = 7.2 Hz, 2H), 5.81 (s, 2H), 4.24 (td, J = 6.6, 1.9 Hz, 2H), 2.11 (s, 3H), 1.76 (q, J = 7.1 Hz, 2H), 1.65 – 1.43 (m, 4H), 1.24 (d, J = 6.7 Hz, 12H), 0.90 (t, J = 6.8 Hz, 1H). **^{13}C NMR** (126 MHz, $CDCl_3$) δ 168.8, 141.3, 138.7, 136.3, 135.96, 129.65, 128.36, 128.25, 127.56, 119.52, 117.30, 105.50, 83.25, 64.93, 28.87, 28.77, 26.12, 25.04, 24.51, 14.08. **^{11}B NMR** (160 MHz, $CDCl_3$) δ 33.24, 33.15. **HRMS** (ESI, m/z) calcd for $C_{31}H_{39}B_2N_2O_4$ [M+H] $^+$: 525.3090; found: 525.3092. **Specific rotation:** $[\alpha]^{25}_D$= -8.48° (c =0.33, $CHCl_3$). **HPLC analysis**: HPLC DAICEL CHIRALCEL AD, hexane/isopropanol = 90/10, 0.5 mL/min, λ = 280 nm, t_R (major) = 13.6 min, t_R (minor) = 14.9 min, er = 91:9.

(R)-5-(1H-naphtho[1,8-de][1,3,2]diazaborinin-2(3H)-yl)-5-(4,4,5,5-tetramethyl-1,3,2-dioxaborolan-2-yl)pentyl (2R)-2-phenylpropanoate (83)

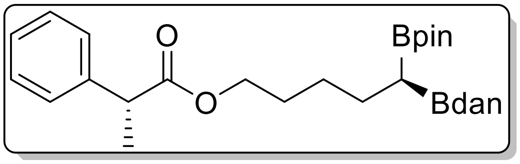

83

The reaction was performed following the **Condition C**. The residue was purified by flash column chromatograph (PE:EA=30:1) to give the product as a yellow oil liquid (60.4 mg, 59%). **^{1}H NMR** (500 MHz, $CDCl_3$) δ 7.32 – 7.28 (m, 5H), 7.10 (t, J = 7.8 Hz, 2H), 7.00 (d, J = 8.3 Hz, 2H), 6.30 (d, J = 7.3 Hz, 2H), 5.78 (s, 2H), 4.07 (t, J = 6.8 Hz, 2H), 3.70 (d, J = 7.3 Hz, 1H), 1.62 (d, J = 7.1 Hz, 2H), 1.49 (d, J = 7.3 Hz, 3H), 1.27 (s, 2H), 1.25 (s, 6H), 1.23 (s, 6H), 1.22 (s, 2H), 0.72 – 0.68 (m, 1H). **^{13}C NMR** (126 MHz, $CDCl_3$) δ 174.6, 141.3, 136.3, 128.6, 127.6, 127.5, 127.1, 117.3, 105.5, 83.2, 64.7, 45.6, 28.7, 28.5, 26.0, 25.0, 24.9, 24.8, 24.54, 24.52, 18.5. **^{11}B NMR** (160 MHz, $CDCl_3$) δ 34.44, 33.92. **HRMS** (ESI, m/z) calcd for $C_{30}H_{39}B_2N_2O_4$ [M+H] $^+$: 513.3090; found: 513.3094. **Specific rotation:** $[\alpha]^{25}_D$=-16.3° (c = 1.00, $CHCl_3$). Dr >95:5.

(R)-5-(1H-naphtho[1,8-de][1,3,2]diazaborinin-2(3H)-yl)-5-(4,4,5,5-tetramethyl-1,3,2-dioxaborolan-2-yl)pentyl (S)-2-(4-isobutylphenyl)propanoate (84)

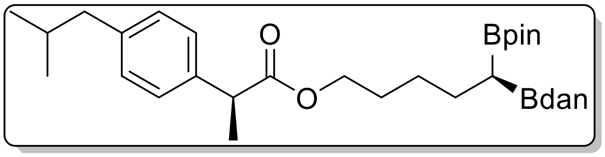

84

The reaction was performed following the general procedure C. The residue was purified by flash column chromatograph to give the product as a crystalline solid (64.8 mg, 57%). **^{1}H NMR** (500 MHz, $CDCl_3$) δ 7.18 (d, J = 7.9 Hz, 2H), 7.08 (dd, J = 10.7, 7.8 Hz, 4H), 7.00 (d, J = 8.2 Hz, 2H), 6.30 (d, J = 7.3 Hz, 2H), 5.78 (s, 2H), 4.09 – 4.04 (m, 2H), 3.67 (d, J = 7.2 Hz, 1H), 2.43 (d, J = 7.3 Hz, 2H), 1.85 – 1.82 (m, 1H), 1.61 (d, J = 6.9 Hz, 2H), 1.47 (d, J = 7.2 Hz, 3H), 1.34 (dq, J = 11.2, 3.6 Hz, 2H), 1.27 (s, 3H), 1.25 (s, 3H), 1.23 (s, 6H), 0.89 (d, J = 6.6 Hz, 6H), 0.72 – 0.69 (m, 1H). **^{13}C NMR** (126 MHz, $CDCl_3$) δ 174.9, 141.3, 140.5, 137.9, 136.3, 129.3, 127.6, 127.2, 119.5, 117.3, 105.5, 64.7, 45.2, 45.0, 30.2, 28.7, 28.5, 26.0, 25.0, 24.9, 24.8, 24.5,

22.4, 18.5. **^{11}B NMR** (160 MHz, $CDCl_3$) δ 34.62, 34.09. **HRMS** (ESI, m/z) calcd for $C_{34}H_{47}B_2N_2O_4$ $[M+H]^+$: 569.3716; found: 569.3713. **Specific rotation:** $[\alpha]^{25}_D$= -3.03° (c = 0.99, $CHCl_3$). Dr >95:5.

(R)-5-(1H-naphtho[1,8-de][1,3,2]diazaborinin-2(3H)-yl)-5-(4,4,5,5-tetramethyl-1,3,2-dioxaborolan-2-yl)pentyl (S)-2-(6-methoxynaphthalen-2-yl)propanoate (85)

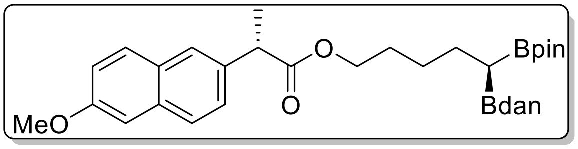

85

The reaction was performed following the general procedure C. The residue was purified by flash column chromatograph to give the product as a crystalline solid (69.9 mg, 59%). **^{1}H NMR** (500 MHz, $CDCl_3$) δ 7.74 – 7.70 (m, 3H), 7.46 (dd, *J* = 8.4, 1.9 Hz, 1H), 7.16 (dd, *J* = 16.1, 8.2 Hz, 4H), 7.06 (d, *J* = 8.2 Hz, 2H), 6.34 (d, *J* = 7.3 Hz, 2H), 5.84 (s, 2H), 4.18 – 4.12 (m, 2H), 3.91 (d, *J* = 5.5 Hz, 3H), 3.88 (d, *J* = 7.2 Hz, 1H), 1.66 (d, *J* = 7.2 Hz, 2H), 1.63 (s, 3H), 1.53 (dt, *J* = 10.1, 6.4 Hz, 2H), 1.38 – 1.33 (m, 2H), 1.28 – 1.26 (m, 12H), 0.73 – 0.68 (m, 1H). **^{13}C NMR** (126 MHz, $CDCl_3$) δ 177.9, 157.0, 141.2, 136.4, 136.3, 130.3, 127.5, 123.6, 120.7, 119.5, 117.3, 112.0, 105.5, 83.2, 68.0, 64.4, 42.1, 37.1, 28.8, 28.6, 26.1, 25.2, 25.0, 24.8, 24.5, 21.4, 15.8. **^{11}B NMR** (160 MHz, $CDCl_3$) δ 34.62, 33.51. **HRMS** (ESI, m/z) calcd for $C_{35}H_{43}B_2N_2O_5$ $[M+H]^+$:593.3353; found: 593.3346. **Specific rotation:** $[\alpha]^{25}_D$= 5.00° (c = 0.50, $CHCl_3$). Dr > 95:5.

(R)-5-(1H-naphtho[1,8-de][1,3,2]diazaborinin-2(3H)-yl)-5-(4,4,5,5-tetramethyl-1,3,2-dioxaborolan-2-yl)pentyl (1R,2S,4R)-bicyclo[2.2.1]hept-5-ene-2-carboxylate (86)

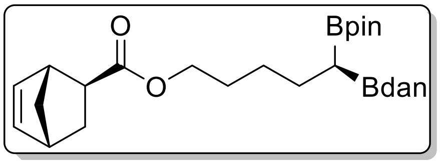

86

The reaction was performed following the general procedure C. The residue was purified by flash column chromatograph to give the product as a crystalline solid (60.0 mg, 60%). **^{1}H NMR** (500 MHz, $CDCl_3$) δ 7.09 (t, *J* = 7.7 Hz, 2H), 6.99 (d, *J* = 8.2 Hz, 2H), 6.29 (d, *J* = 7.2 Hz, 2H), 6.12 (dd, *J* = 5.7, 3.1 Hz, 1H), 6.09 – 6.06 (m, 1H), 5.79 (s, 2H), 4.11 – 4.07 (m, 2H), 3.02 (s, 1H), 2.89 (s, 1H), 2.22 – 2.19 (m, 1H), 1.93 – 1.89 (m, 1H), 1.67 (dd, *J* = 12.3, 5.1 Hz, 4H), 1.51 (d, *J* = 8.4 Hz, 2H), 1.39 – 1.31 (m, 4H), 1.24 (d, *J* = 6.6 Hz, 12H), 0.75 (dd, *J* = 9.4, 6.1 Hz, 1H). **^{13}C NMR** (126 MHz, $CDCl_3$) δ 176.3, 141.,3 138.0, 136.3, 135.8, 127.5, 117.3, 105.5, 64.4, 46.6, 46.4, 43.2, 41.6, 30.3, 28.9, 28.6, 26.1, 25.0, 24.8, 24.5. **^{11}B NMR** (160 MHz, $CDCl_3$) δ 35.22, 34.45. **HRMS** (ESI, m/z) calcd for $C_{29}H_{38}{}^{10}B_2KN_2O_4$ $[M+K]^+$: 537.2722; found: 537.2725. **Specific rotation:** $[\alpha]^{25}_D$= -7.4° (c = 0.50, $CHCl_3$). Dr >95:5.

(R)-2-(2-phenyl-1-(4,4,5,5-tetramethyl-1,3,2-dioxaborolan-2-yl)ethyl)-2,3-dihydro-1H-naphtho[1,8-de][1,3,2]diazaborinine (87[a])

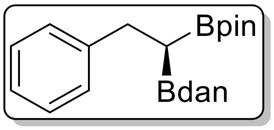

87[a]

The reaction was performed following the general procedure C. The residue was purified by flash column chromatograph to give the product as a crystalline solid (34.2 mg, 43%). **^{1}H NMR** (500 MHz, $CDCl_3$) δ 7.25 (s, 4H), 7.17 – 7.14 (m, 1H), 7.08 (t, J = 7.8 Hz, 2H), 6.99 (d, J = 8.2 Hz, 2H), 6.27 (d, J = 7.3 Hz, 2H), 5.81 (s, 2H), 2.96 (dd, J = 14.1, 9.6 Hz, 1H), 2.91 – 2.86 (m, 1H), 1.28 – 1.25 (m, 1H), 1.18 (s, 6H), 1.16 (s, 6H). **^{13}C NMR** (126 MHz, $CDCl_3$) δ 143.7, 141.2, 136.3, 128.3, 128.2, 127.5, 125.7, 119.5, 117.3, 105.5, 83.4, 32.0, 24.9, 24.5. **^{11}B NMR** (160 MHz, $CDCl_3$) δ 34.27, 33.68. **HRMS** (ESI, m/z) calcd for $C_{24}H_{29}B_2N_2O_2$ $[M+H]^+$: 399.2410; found: 399.2418. **Specific rotation:** $[\alpha]^{25}_{D}$= -0.96° (c = 0.52, $CHCl_3$). **HPLC analysis**: HPLC DAICEL CHIRALCEL OD, hexane/isopropanol = 95/5, 0.5 mL/min, λ = 210 nm, t_R (major) = 17.2 min, t_R (minor) = 19.6 min, er = 71:29.

(S)-2-(2-phenyl-1-(4,4,5,5-tetramethyl-1,3,2-dioxaborolan-2-yl)ethyl)-2,3-dihydro-1H-naphtho[1,8-de][1,3,2]diazaborinine (87[b])

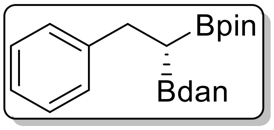

87[b]

The reaction was performed following the general procedure D. The residue was purified by flash column chromatograph to give the product as a crystalline solid (41.4 mg, 52%). **^{1}H NMR** (500 MHz, $CDCl_3$) δ 7.25 (s, 4H), 7.17 – 7.14 (m, 1H), 7.08 (t, J = 7.8 Hz, 2H), 6.99 (d, J = 8.2 Hz, 2H), 6.27 (d, J = 7.3 Hz, 2H), 5.81 (s, 2H), 2.96 (dd, J = 14.1, 9.6 Hz, 1H), 2.91 – 2.86 (m, 1H), 1.28 – 1.25 (m, 1H), 1.18 (s, 6H), 1.16 (s, 6H). **^{13}C NMR** (126 MHz, $CDCl_3$) δ 141.3, 140.6, 136.3, 135.1, 129.0, 128.1, 127.6, 119.6, 117.3, 105.5, 83.4, 31.5, 29.7, 25.0, 24.8, 24.5, 21.0. **^{11}B NMR** (160 MHz, $CDCl_3$) δ 34.27, 33.68. **HRMS** (ESI, m/z) calcd for $C_{24}H_{29}B_2N_2O_2$ $[M+H]^+$: 399.2410; found: 399.2418. **Specific rotation:** $[\alpha]^{25}_{D}$= 3.39° (c = 0.62, $CHCl_3$). **HPLC analysis**: HPLC DAICEL CHIRALCEL OD, hexane/isopropanol = 95/5, 0.5 mL/min, λ = 210 nm, t_R (minor) = 17.0 min, t_R (major) = 19.3 min, er = 9:91.

(S)-2-(1-(4,4,5,5-tetramethyl-1,3,2-dioxaborolan-2-yl)-2-(p-tolyl)ethyl)-2,3-dihydro-1H-naphtho[1,8-de][1,3,2]diazaborinine (88)

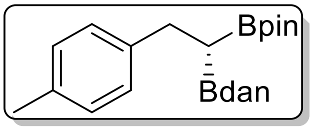

88

The reaction was performed following the general procedure D. The residue was purified by flash column chromatograph to give the product as a crystalline solid (48.6 mg, 59%). **^{1}H NMR** (500 MHz, $CDCl_3$) δ 7.17

(d, J = 7.8 Hz, 2H), 7.11 – 7.07 (m, 4H), 7.00 (d, J = 8.2 Hz, 2H), 6.28 (d, J = 7.3 Hz, 2H), 5.83 (s, 2H), 2.95 (dd, J = 14.2, 9.3 Hz, 1H), 2.87 (t, J = 4.7 Hz, 1H), 2.32 (s, 3H), 1.20-1.26 (m, 1H), 1.21 (s, 6H), 1.19 (s, 6H). **^{13}C NMR** (126 MHz, $CDCl_3$) δ 141.2 (s), 140.6 (s), 136.3 (s), 135.1 (s), 128.9 (s), 128.0 (s), 127.5 (s), 119.5 (s), 117.3 (s), 105.5 (s), 83.3 (s), 31.5 (s), 25.0 (s), 24.5 (s), 21.0 (s). **^{11}B NMR** (160 MHz, $CDCl_3$) δ 34.27, 34.09. **HRMS** (ESI, m/z) calcd for $C_{25}H_{30}B_2N_2NaO_2$ $[M+Na]^+$: 435.2386; found: 435.2381. **Specific rotation:** $[\alpha]^{25}_{D}$= 4.5° (c = 0.22, $CHCl_3$). **HPLC analysis**: HPLC DAICEL CHIRALCEL AD, hexane/isopropanol = 95/5, 0.5 mL/min, λ = 280 nm, t_R (major) = 14.8 min, t_R (minor) = 24.0 min, er = 86:14.

(S)-2-(1-(4,4,5,5-tetramethyl-1,3,2-dioxaborolan-2-yl)-2-(m-tolyl)ethyl)-2,3-dihydro-1H-naphtho[1,8-de][1,3,2]diazaborinine (99)

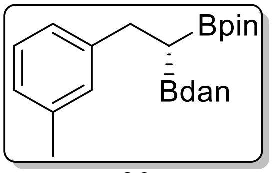

89

The reaction was performed following the general procedure D. The residue was purified by flash column chromatograph to give the product as a crystalline solid (46.9 mg, 57%). **^{1}H NMR** (500 MHz, $CDCl_3$) δ 7.14 (d, J = 7.5 Hz, 1H), 7.08 (t, J = 7.8 Hz, 5H), 6.99 (d, J = 8.2 Hz, 3H), 6.27 (d, J = 7.3 Hz, 2H), 5.81 (s, 2H), 2.92 (dd, J = 14.0, 9.7 Hz, 1H), 2.84 (dd, J = 14.1, 6.5 Hz, 1H), 2.31 (s, 3H), 1.21 (s, 1H), 1.19 (s, 6H), 1.17 (s, 6H). **^{13}C NMR** (126 MHz, $CDCl_3$) δ 143.7 (s), 141.2 (s), 137.7 (s), 129.0 (s), 128.2 (s), 127.5 (s), 126.4 (s), 125.2 (s), 117.3 (s), 105.5 (s), 83.3 (s), 31.9 (s), 25.0 (s), 24.5 (s), 21.4 (s). **^{11}B NMR** (160 MHz, $CDCl_3$) δ 34.32, 34.09. **HRMS** (ESI, m/z) calcd for $C_{25}H_{30}B_2N_2NaO_2$ $[M+Na]^+$: 435.2386; found: 435.2391. **Specific rotation:** $[\alpha]^{25}_{D}$= 5.28° (c = 0.53, $CHCl_3$). **HPLC analysis**: HPLC DAICEL CHIRALCEL AD, hexane/isopropanol = 95/5, 0.5 mL/min, λ = 210 nm, t_R (major) = 11.9 min, t_R (minor) = 14.2 min, er = 79.5:20.5.

(S)-2-(2-(4-ethylphenyl)-1-(4,4,5,5-tetramethyl-1,3,2-dioxaborolan-2-yl)ethyl)-2,3-dihydro-1H-naphtho[1,8-de][1,3,2]diazaborinine (90)

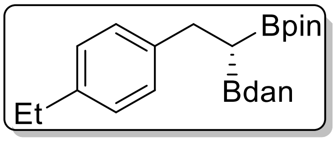

90

The reaction was performed following the general procedure D. The residue was purified by flash column chromatograph to give the product as a crystalline solid (37.9 mg, 46%). **^{1}H NMR** (500 MHz, $CDCl_3$) δ 7.14 (d, J = 7.9 Hz, 2H), 7.10 – 7.05 (m, 4H), 6.98 (d, J = 8.2 Hz, 2H), 6.26 (d, J = 7.3 Hz, 2H), 5.81 (s, 2H), 2.93 (dd, J = 14.2, 9.3 Hz, 1H), 2.84 (dd, J = 14.2, 6.8 Hz, 1H), 2.30 (s, 3H), 1.26 (s, 2H), 1.18 (d, J = 8.4 Hz, 12H), 1.15 – 1.13 (m, 1H). **^{13}C NMR** (126 MHz, $CDCl_3$) δ 141.2 (s), 140.6 (s), 135.1 (s), 128.9 (s), 128.0 (s), 127.5 (s), 117.3 (s), 105.5 (s), 83.3 (s), 31.5 (s), 25.0 (s), 24.5 (s), 21.0 (s). **^{11}B NMR** (160 MHz, $CDCl_3$) δ 34.45, 34.09. **HRMS** (ESI, m/z) calcd for $C_{26}H_{33}B_2N_2O_2$ $[M+H]^+$: 427.2723; found: 427.2722. **Specific**

rotation: [α]$^{25}_{D}$= 7.63° (c = 0.76, $CHCl_3$). **HPLC analysis**: HPLC DAICEL CHIRALCEL AD, hexane/isopropanol = 95/5, 0.5 mL/min, λ = 250 nMMm, t_R (major) = 14.7 min, t_R (minor) = 21.2 min, er = 78:22.

(R)-2-(2-(4-propylphenyl)-1-(4,4,5,5-tetramethyl-1,3,2-dioxaborolan-2-yl)ethyl)-2,3-dihydro-1H-naphtho[1,8-de][1,3,2]diazaborinine (91)

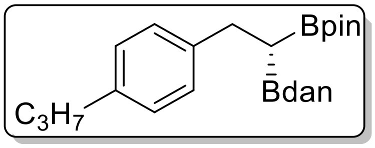

91

The reaction was performed following the general procedure D. The residue was purified by flash column chromatograph to give the product as a crystalline solid (45.8 mg, 52%). **^{1}H NMR** (500 MHz, $CDCl_3$) δ 7.19 (d, *J* = 7.8 Hz, 2H), 7.10 (t, *J* = 7.8 Hz, 4H), 7.01 (dd, *J* = 8.3, 1.0 Hz, 2H), 6.28 (dd, *J* = 7.3, 1.0 Hz, 2H), 5.84 (s, 2H), 2.96 (dd, *J* = 14.2, 9.4 Hz, 1H), 2.88 (dd, *J* = 14.1, 6.9 Hz, 1H), 2.58 – 2.55 (m, 2H), 1.66 – 1.61 (m, 2H), 1.24 – 1.22 (m, 1H), 1.20 (d, *J* = 7.8 Hz, 12H), 0.94 (t, *J* = 7.3 Hz, 3H). **^{13}C NMR** (126 MHz, $CDCl_3$) δ 141.3, 140.9, 140.0, 136.3, 128.4, 128.1, 127.6, 119.6, 117.3, 105.5, 83.4, 37.7, 31.6, 24.9, 24.7, 24.5, 13.8. **^{11}B NMR** (160 MHz, $CDCl_3$) δ 33.68, 32.10. **HRMS** (ESI, m/z) calcd for $C_{27}H_{35}B_2N_2O_2$ [M+H]$^+$: 441.2879; found: 441.2878. **Specific rotation:** [α]$^{25}_{D}$= 8.8° (c = 0.68, $CHCl_3$). **HPLC analysis**: HPLC DAICEL CHIRALCEL AD, hexane/isopropanol = 95/5, 0.5 mL/min, λ = 210 nm, t_R (major) = 13.4 min, t_R (minor) = 17.8 min, er = 77:23.

2-(4-phenylbutyl-1-d)-2,3-dihydro-1H-naphtho[1,8-de][1,3,2]diazaborinine (97)

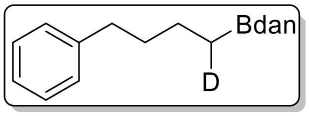

97

The reaction was performed following the general procedure F. The residue was purified by flash column chromatograph to give the product as a crystalline solid (44.5 mg, 74%). **^{1}H NMR** (500 MHz, $CDCl_3$) δ 7.32 – 7.28 (m, 2H), 7.22 – 7.18 (m, 3H), 7.10 (td, *J* = 7.7, 2.8 Hz, 2H), 7.01 (dd, *J* = 8.3, 2.8 Hz, 2H), 6.28 (d, *J* = 10.2 Hz, 2H), 5.57 (s, 2H), 2.66 (dt, *J* = 7.8, 3.9 Hz, 2H), 1.73 – 1.67 (m, 2H), 1.47 (d, *J* = 8.2 Hz, 2H), 0.87 (t, *J* = 9.0 Hz, 1H). **^{13}C NMR** (126 MHz, $CDCl_3$) δ 142.60, 141.16, 136.33, 128.46, 128.33, 127.55, 125.71, 119.57, 117.38, 105.43, 35.78, 34.10, 24.31. **^{11}B NMR** (160 MHz, $CDCl_3$) δ 32.35. **HRMS** (ESI, m/z) calcd for $C_{20}H_{21}DBN_2$ [M+H]$^+$: 302.1933; found: 302.1933.

3.5 DFT Data

Computational Method

Density functional theory (DFT) calculations were carried out with the *Gaussian 16*[3] package. All geometry optimizations were performed using the dispersion-corrected B3LYP1[4-7] functional with Grimme's D3 dispersion correction[8], with a mixed basis set of SDD[9] for Cu and Fe and 6-31G(d) for other atoms. Normal mode vibrational frequency calculations at the same level confirmed that the optimized structures are minima (no imaginary frequency) or transition states (with one imaginary frequency). Single point energies were calculated with the SMD solvation model in cyclohexane ($\varepsilon = 2.0$) with the M06-L[10] functional and a mixed basis set of SDD for Cu and Fe and 6-311+G(d,p) for other atoms[11-12]. Free energies were corrected using Truhlar's quasiharmonic correction, by raising vibrational frequencies that are below 100 cm^{-1} to 100 cm^{-1}.[13] In regard to the standard state change from 1 atm to 1 M at 298.15 K, a correction of $RT\ln(C_s/C_g)$ (= 1.9 kcal/mol) was added to the Gibbs free energy (C_s is the concentration in solution phase and C_g is the concentration in gas phase).[14-16] DFT-optimized structures are illustrated using *CYLView* v1.0 software.[17]

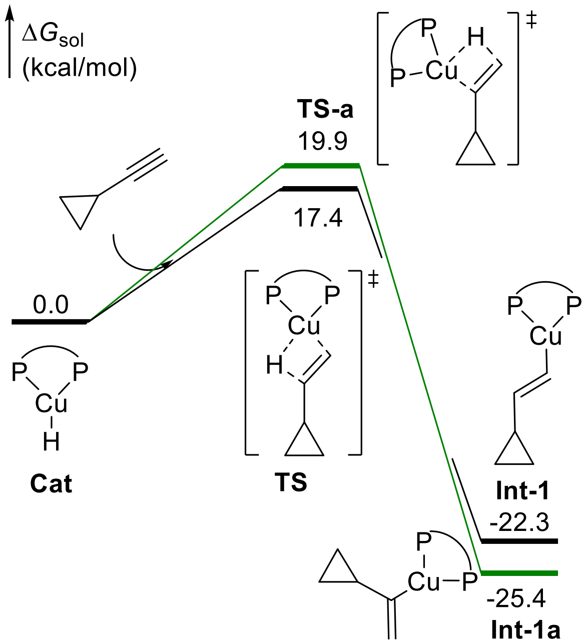

Supplementary Figure 18. DFT calculations on the proposed reaction pathway. Free energy profile for the 1,2- or 2,1-insertion of cyclopropyl alkyne into copper hydride.

4. Supplementary Figures

4.1 NMR Spectroscopic Data

2-(4-phenyl-1-(4,4,5,5-tetramethyl-1,3,2-dioxaborolan-2-yl)butyl)-2,3-dihydro-1H-naphtho[1,8-de][1,3,2]diazaborinine (4)

^{1}H NMR (500 MHz, room temperature, CDCl$_3$)

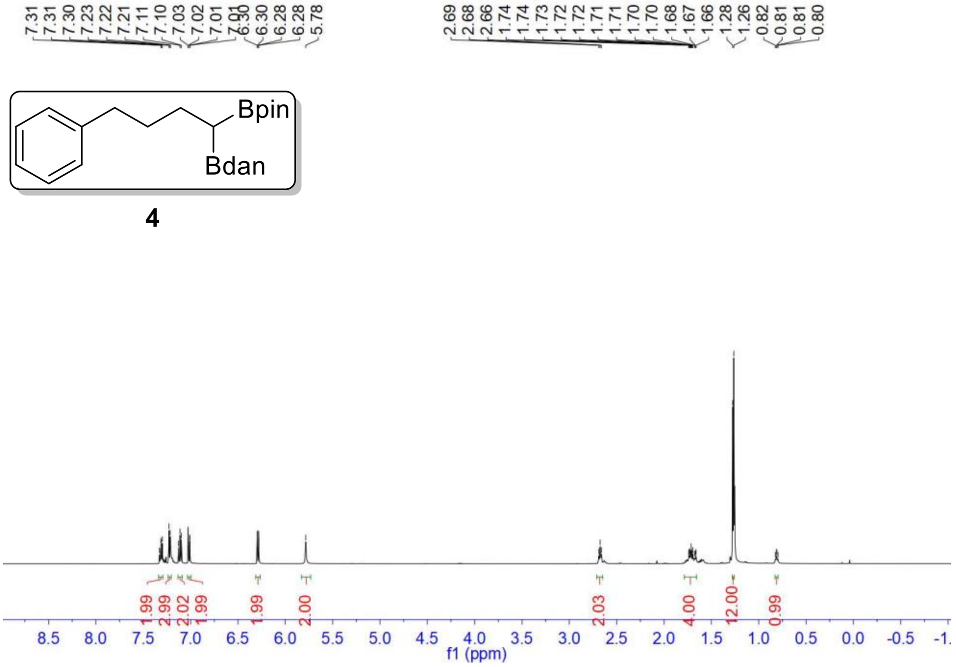

Supplementary Figure 19. ^{1}H NMR spectrum of compound 4

^{13}C NMR (126 MHz, room temperature, CDCl$_3$)

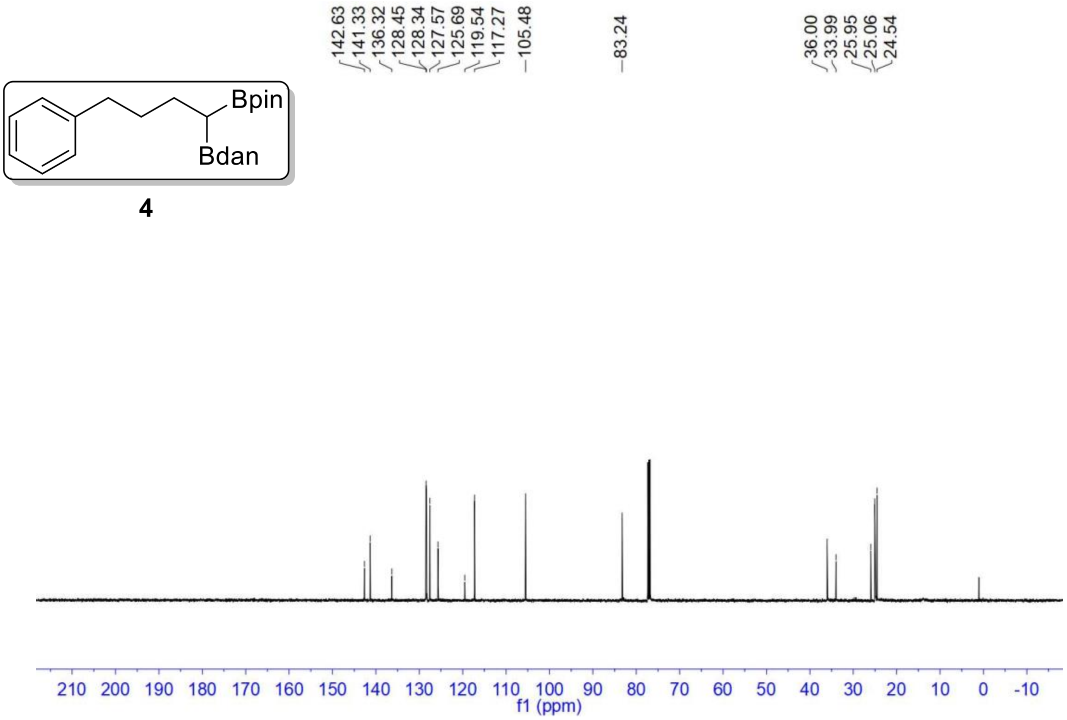

Supplementary Figure 20. ^{13}C NMR spectrum of compound 4

^{11}B NMR (160 MHz, room temperature, $CDCl_3$)

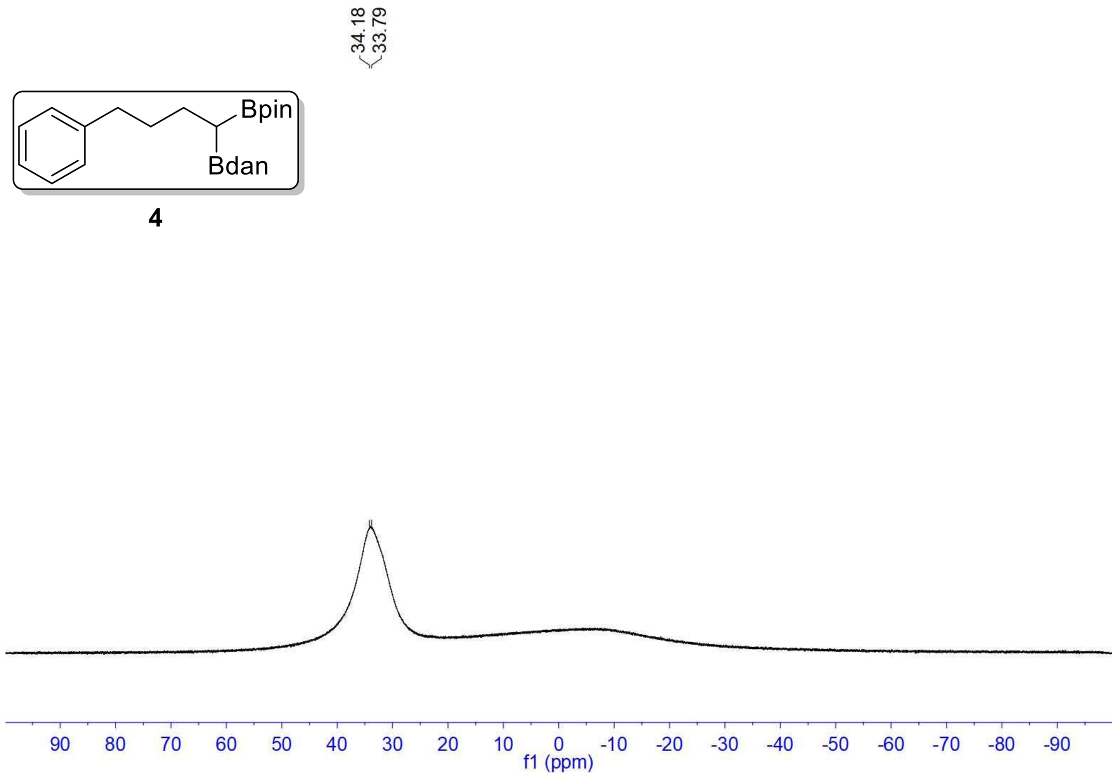

Supplementary Figure 21. ^{11}B NMR spectrum of compound 4

2-(3-phenyl-1-(4,4,5,5-tetramethyl-1,3,2-dioxaborolan-2-yl)propyl)-2,3- dihydro-1H-naphtho[1,8-de][1,3,2]diazaborinine (5)

^{1}H NMR (500 MHz, room temperature, $CDCl_3$)

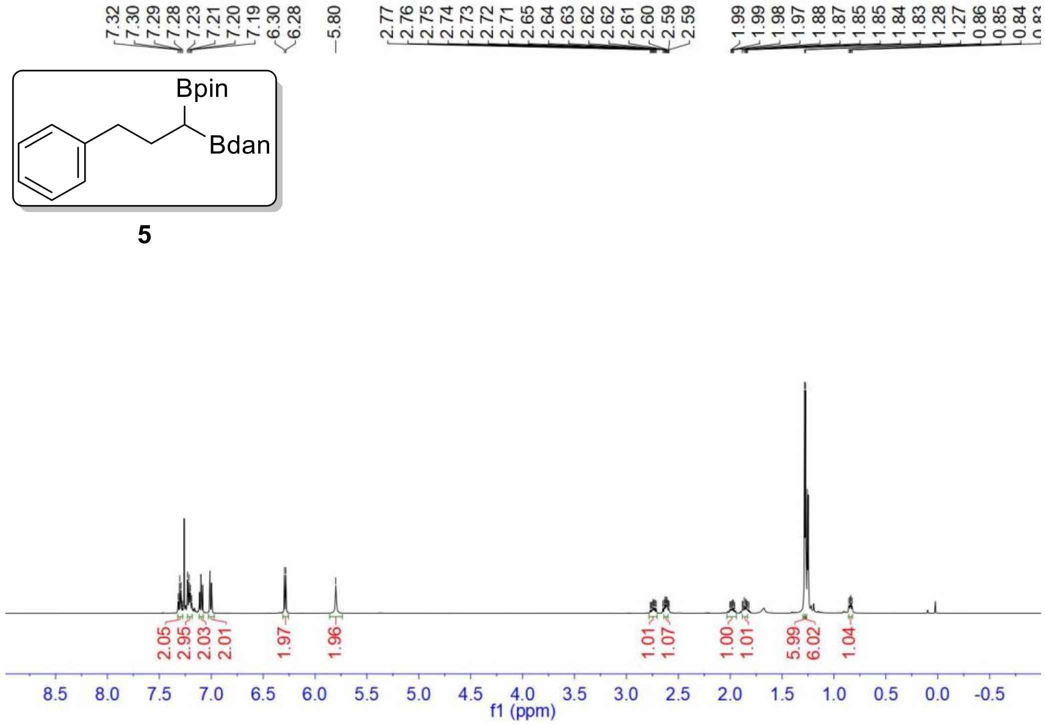

Supplementary Figure 22. ^{1}H NMR spectrum of compound 5

^{13}C NMR (126 MHz, room temperature, CDCl$_3$)

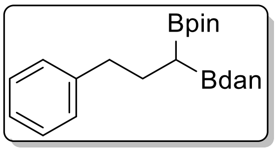

5

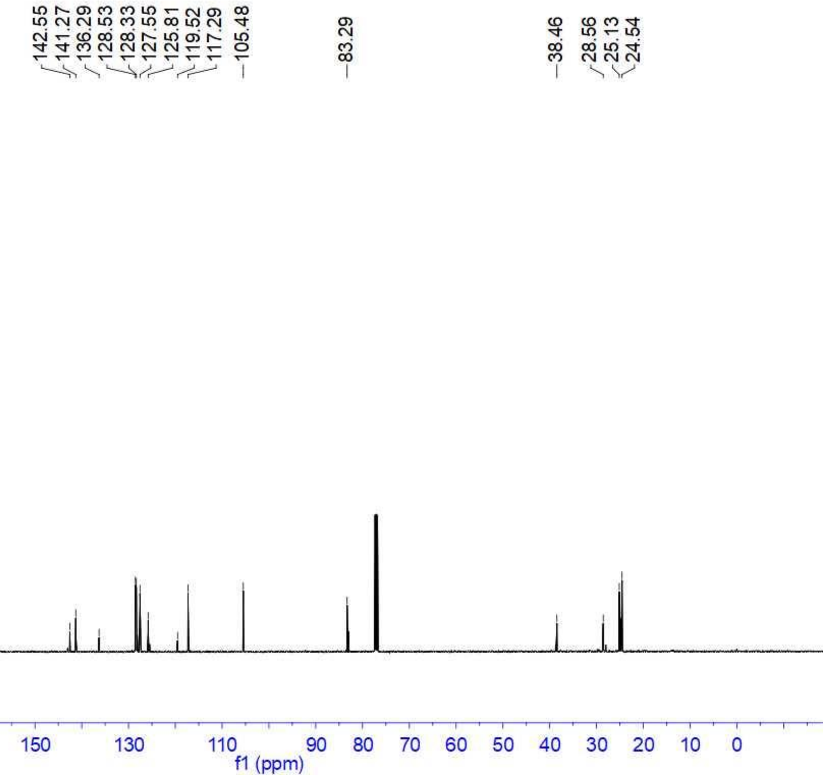

Supplementary Figure 23. ^{13}C NMR spectrum of compound 5

^{11}B NMR (160 MHz, room temperature, CDCl$_3$)

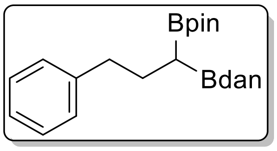

5

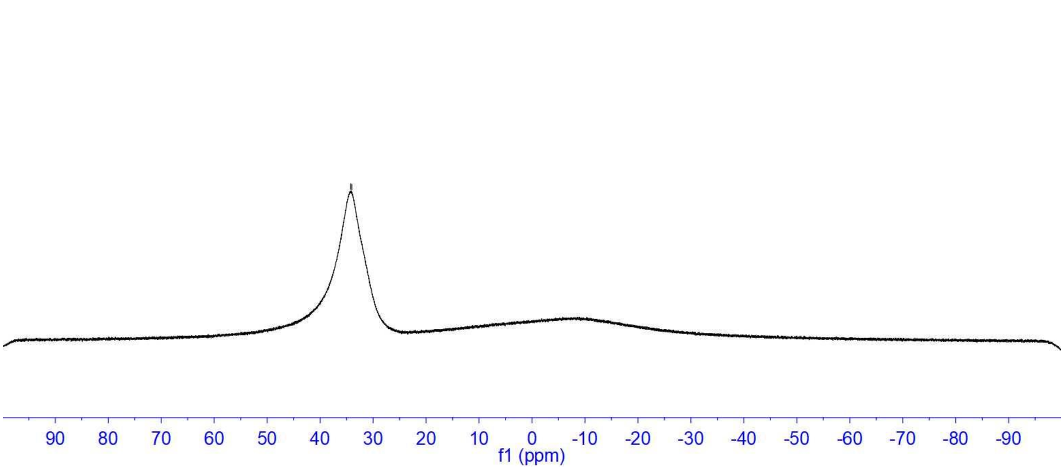

Supplementary Figure 24. ^{11}B NMR spectrum of compound 5

2-(2-cyclopropyl-1-(4,4,5,5-tetramethyl-1,3,2-dioxaborolan-2-yl) ethyl)-2,3-dihydro-1H-naphtho[1,8-de][1,3,2]diazaborinine (6)

^{1}H NMR (500 MHz, room temperature, ($CDCl_3$)

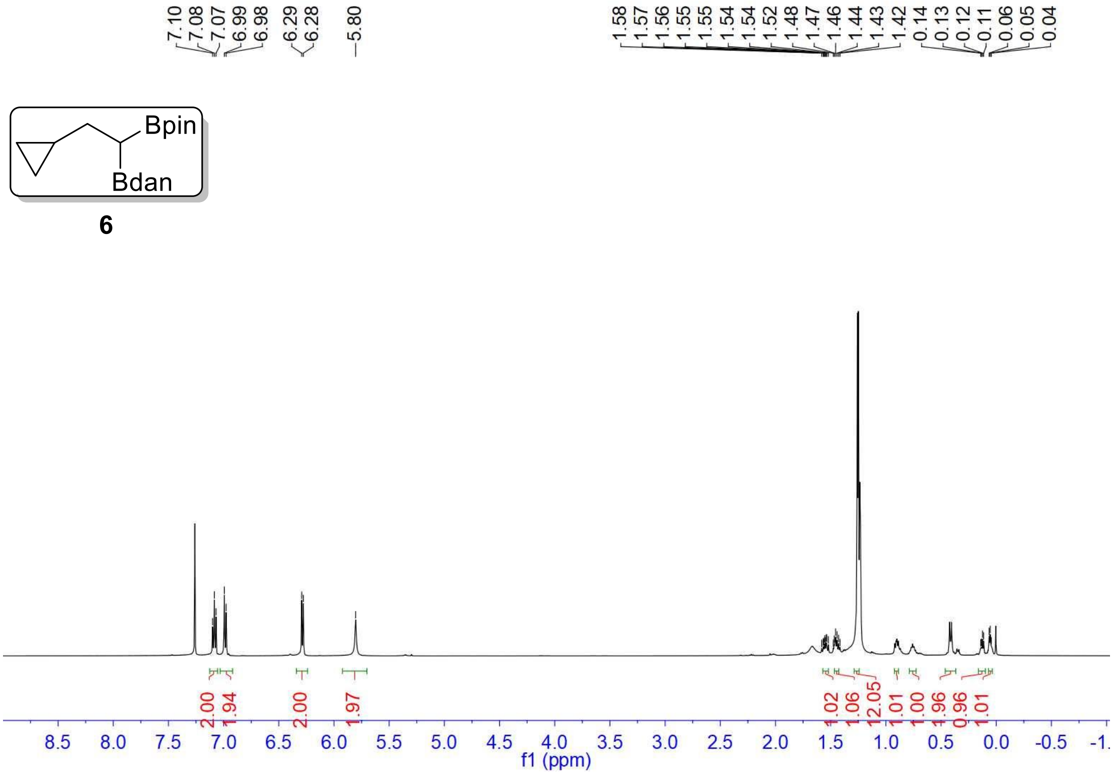

Supplementary Figure 25. ^{1}H NMR spectrum of compound 6

^{13}C NMR (126 MHz, room temperature, $CDCl_3$)

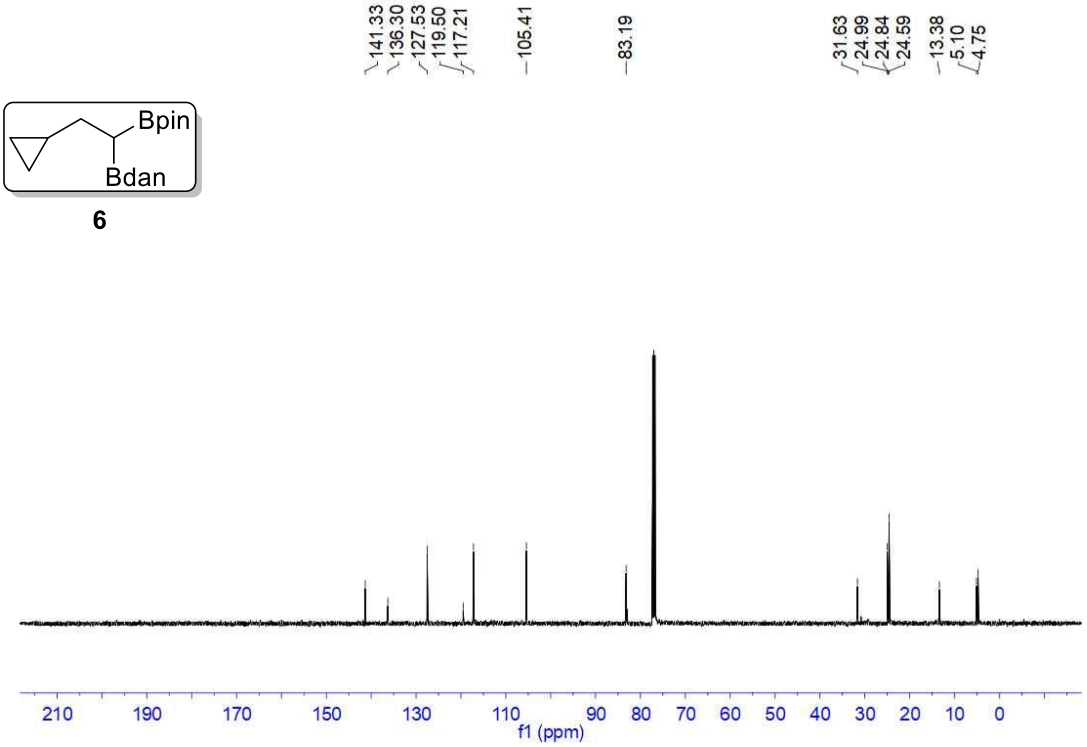

Supplementary Figure 26. ^{13}C NMR spectrum of compound 6

^{11}B NMR (160 MHz, room temperature, $CDCl_3$)

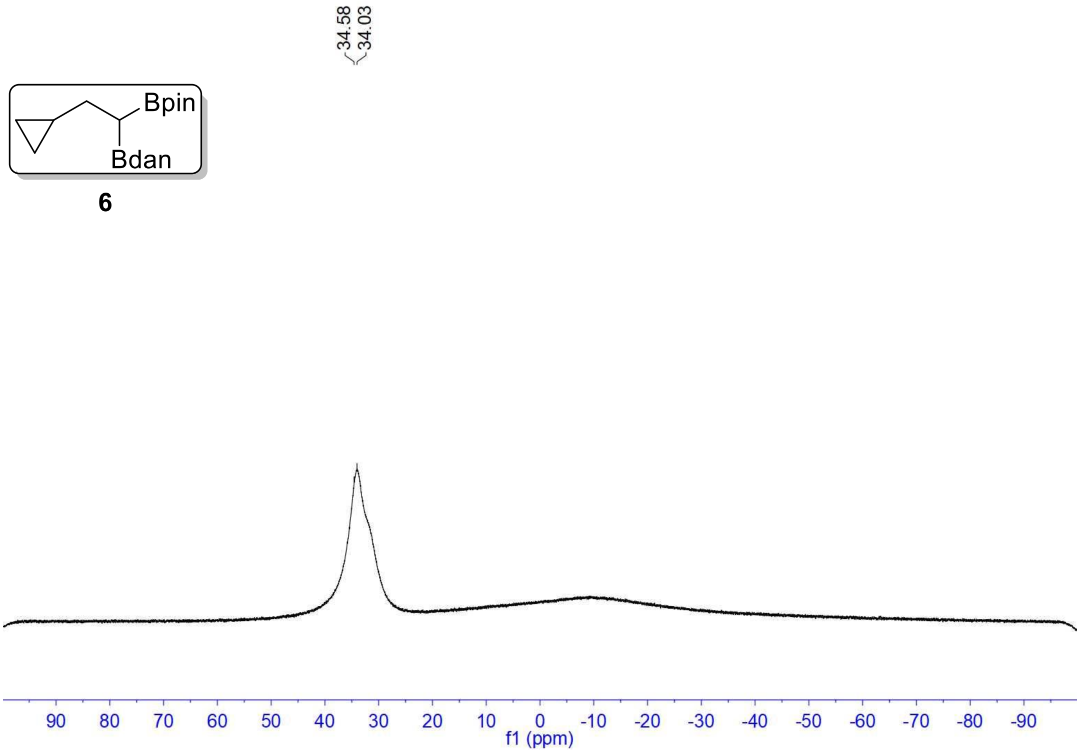

Supplementary Figure 27. ^{11}B NMR spectrum of compound 6

2-(3-cyclohexyl-1-(4,4,5,5-tetramethyl-1,3,2-dioxaborolan-2-yl)propyl) -2,3-dihydro-1H-naphtho[1,8-de][1,3,2]diazaborinine (7)

^{1}H NMR (500 MHz, room temperature, $CDCl_3$)

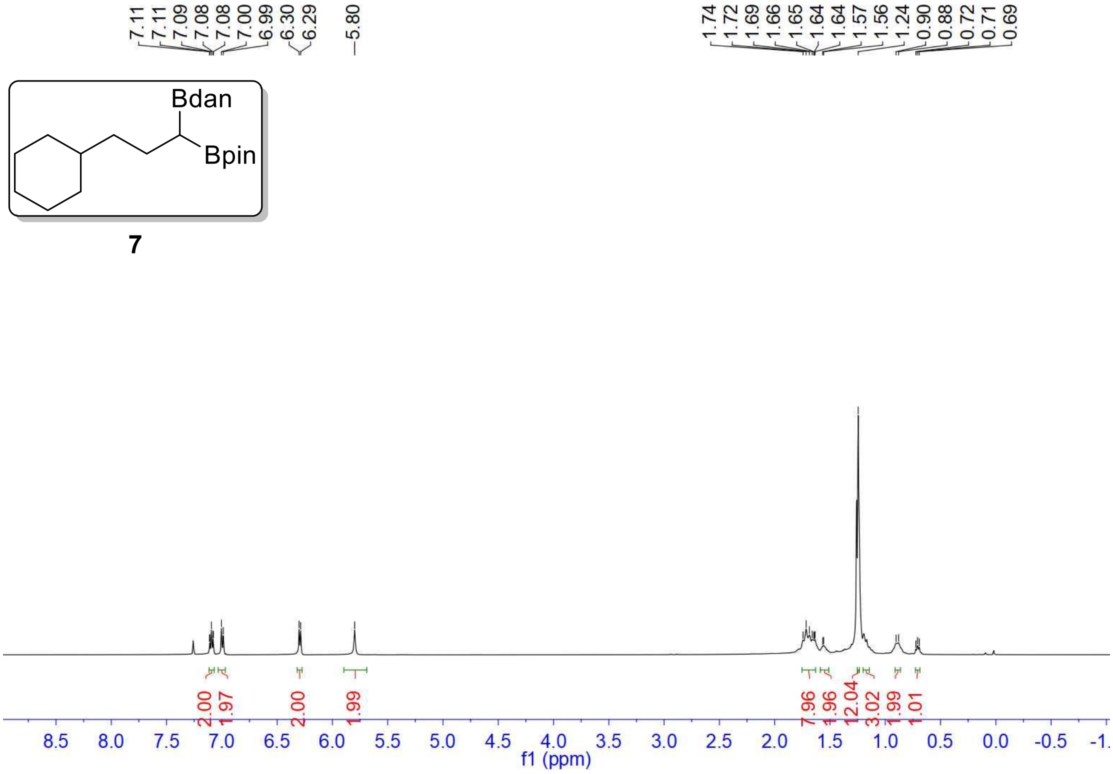

Supplementary Figure 28. ^{1}H NMR spectrum of compound 7

^{13}C NMR (126 MHz, room temperature, $CDCl_3$)

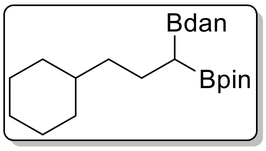

7

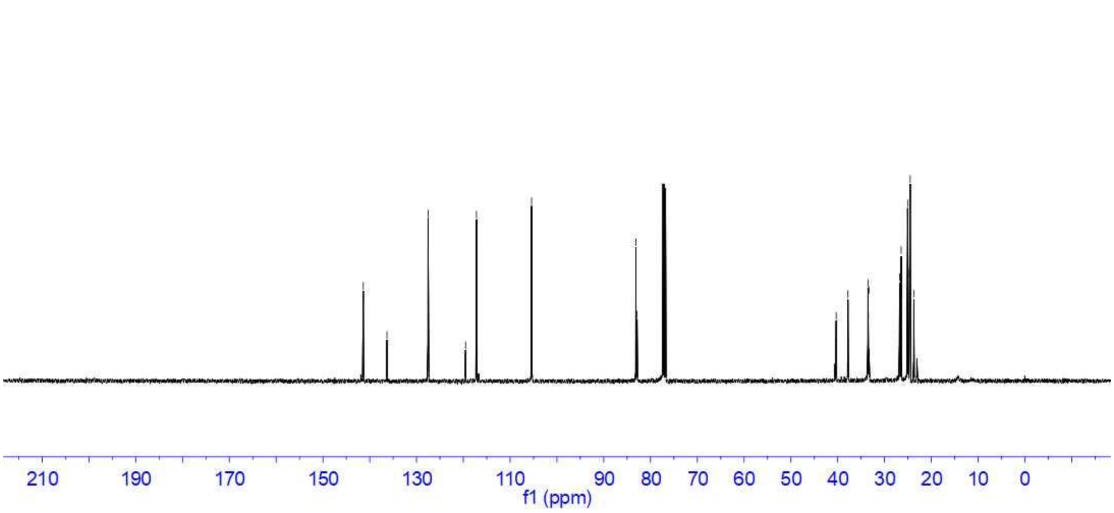

Supplementary Figure 29. ^{13}C NMR spectrum of compound 7

^{11}B NMR (160 MHz, room temperature, $CDCl_3$)

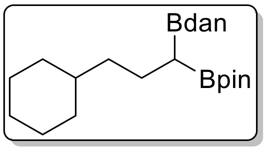

7

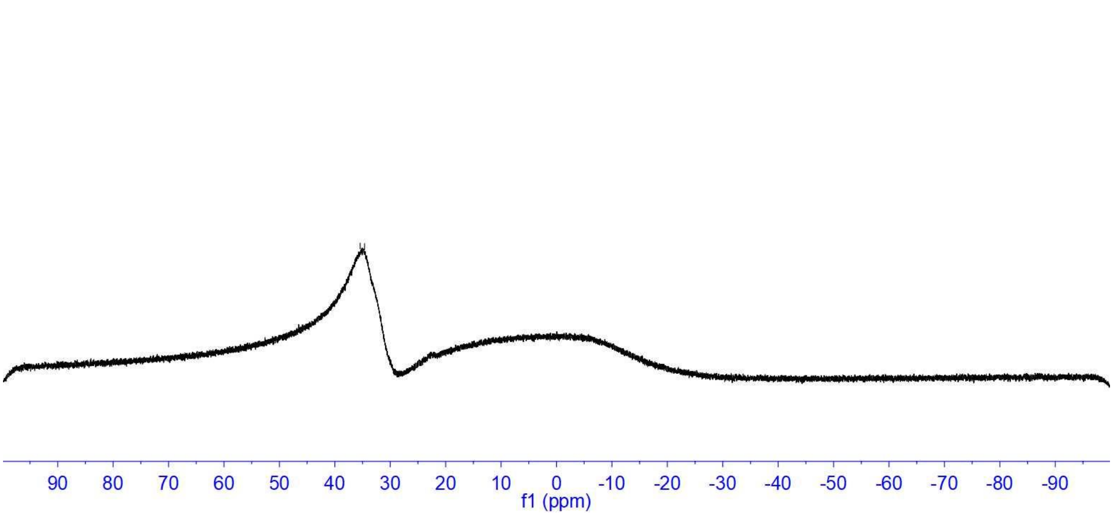

Supplementary Figure 30. ^{11}B NMR spectrum of compound 7

2-(2-cyclopentyl-1-(4,4,5,5-tetramethyl-1,3,2-dioxaborolan-2-yl)ethyl) -2,3-dihydro-1H-naphtho[1,8-de][1,3,2]diazaborinine (8)

^{1}H NMR (500 MHz, room temperature, CDCl$_3$)

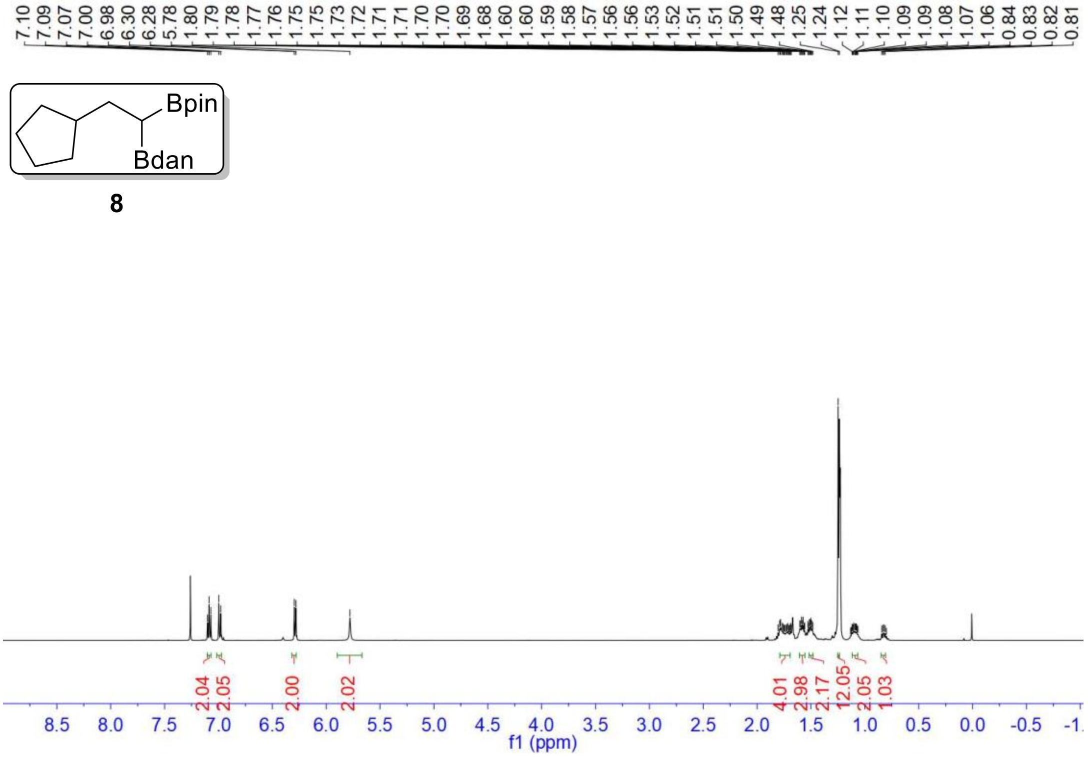

Supplementary Figure 31. ^{1}H NMR spectrum of compound 8

^{13}C NMR (126 MHz, room temperature, CDCl$_3$)

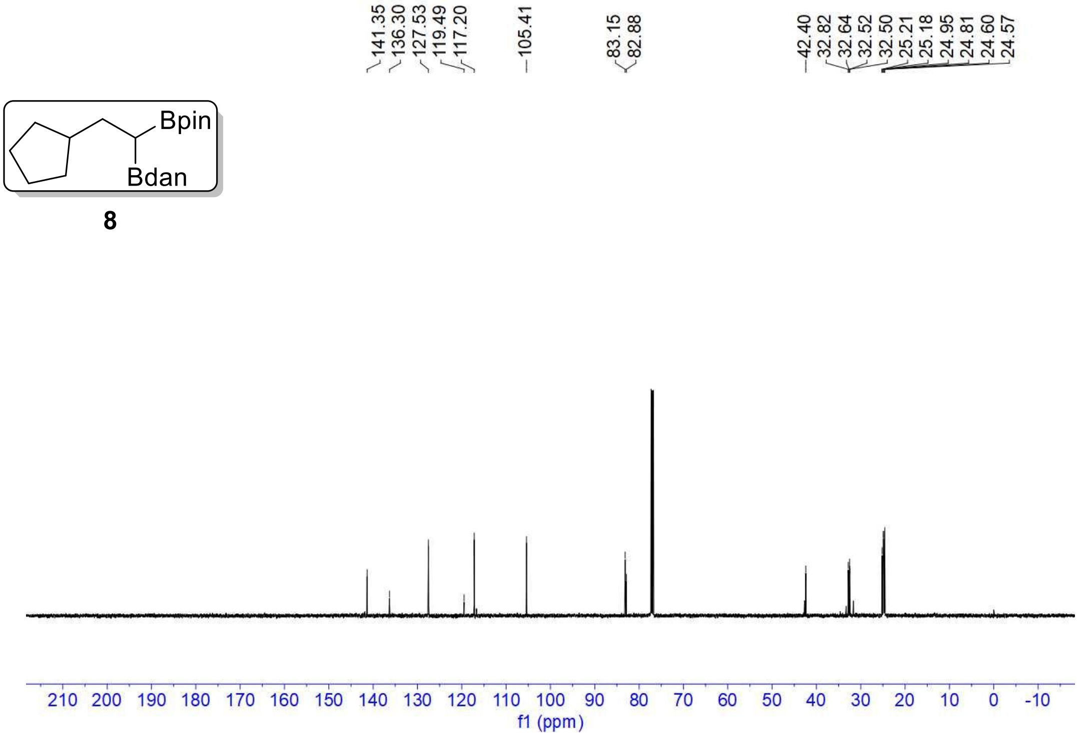

Supplementary Figure 32. ^{13}C NMR spectrum of compound 8

^{11}B NMR (160 MHz, room temperature, $CDCl_3$)

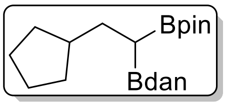

8

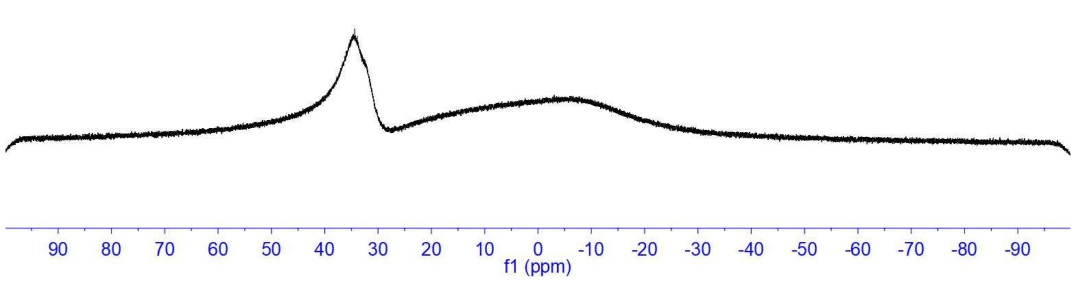

Supplementary Figure 33. ^{11}B NMR spectrum of compound 8

2-(1-(4,4,5,5-tetramethyl-1,3,2-dioxaborolan-2-yl)pentyl) -2,3-dihydro-1H-naphtho[1,8-de][1,3,2]diazaborinine (9)

^{1}H NMR (500 MHz, room temperature, $CDCl_3$)

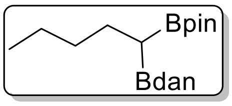

9

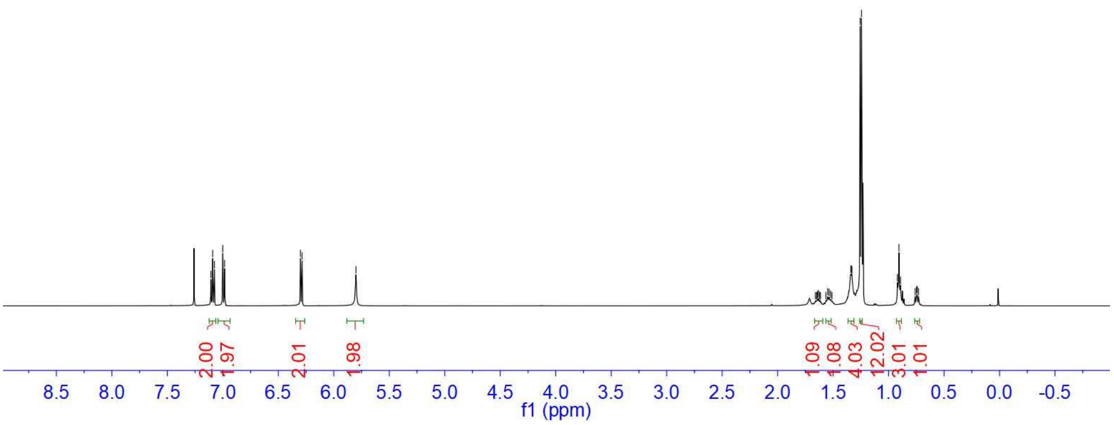

Supplementary Figure 34. ^{1}H NMR spectrum of compound 9

^{13}C NMR (126 MHz, room temperature, $CDCl_3$)

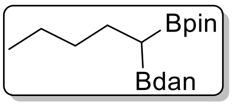

9

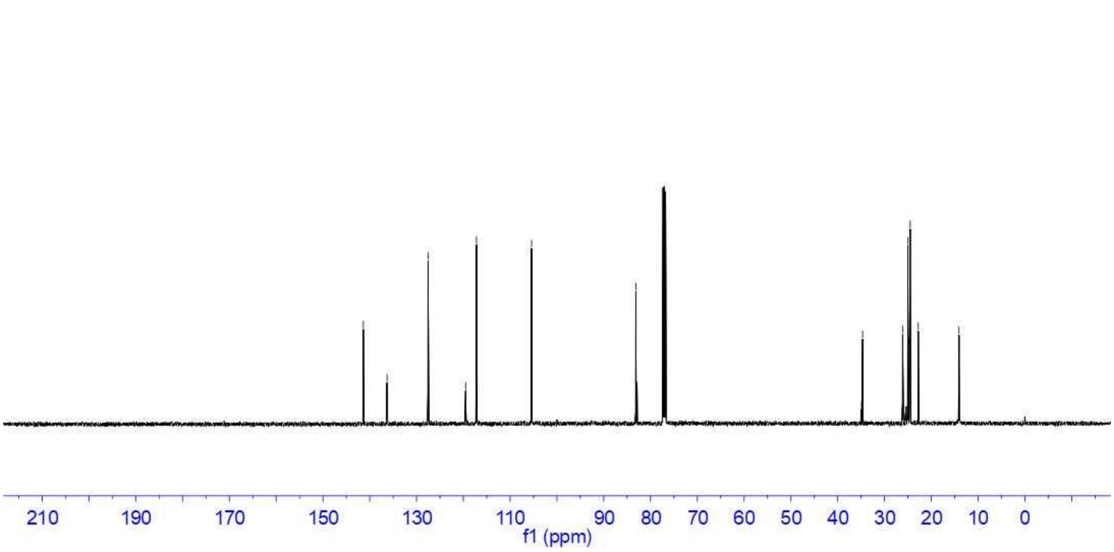

Supplementary Figure 35. ^{13}C NMR spectrum of compound 9

^{11}B NMR (160 MHz, room temperature, $CDCl_3$)

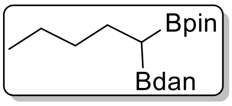

9

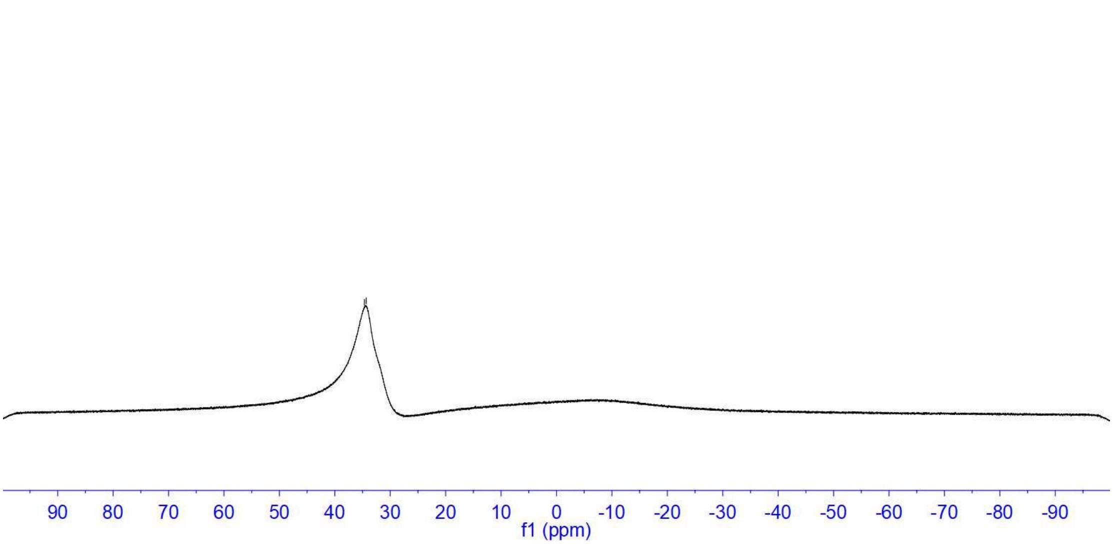

Supplementary Figure 36. ^{11}B NMR spectrum of compound 9

2-(1-(4,4,5,5-tetramethyl-1,3,2-dioxaborolan-2-yl)heptyl)-2,3-dihydro-1H-naphtho[1,8-de][1,3,2]diazaborinine (10)

^{1}H NMR (500 MHz, room temperature, $CDCl_3$)

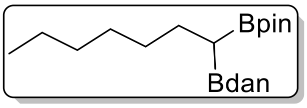

10

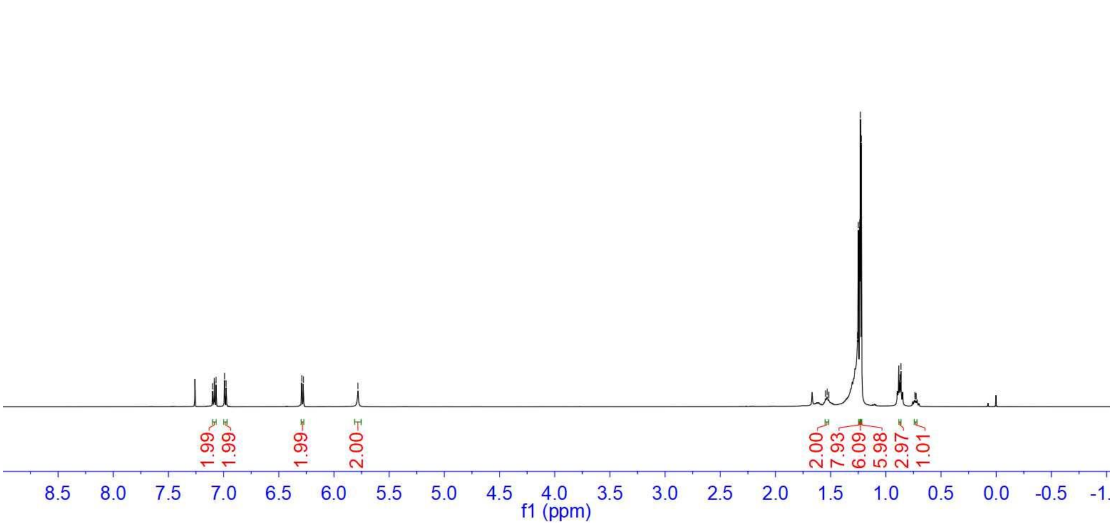

Supplementary Figure 37. ^{1}H NMR spectrum of compound 10

^{13}C NMR (126 MHz, room temperature, $CDCl_3$)

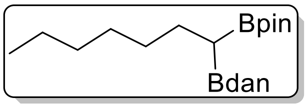

10

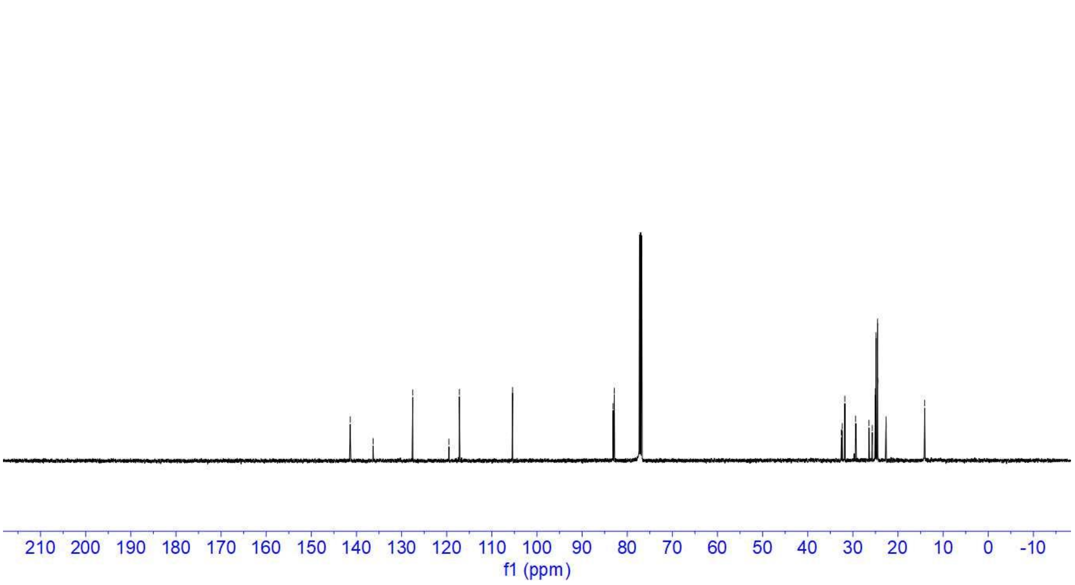

Supplementary Figure 38. ^{13}C NMR spectrum of compound 10

^{11}B NMR (160 MHz, room temperature, CDCl3)

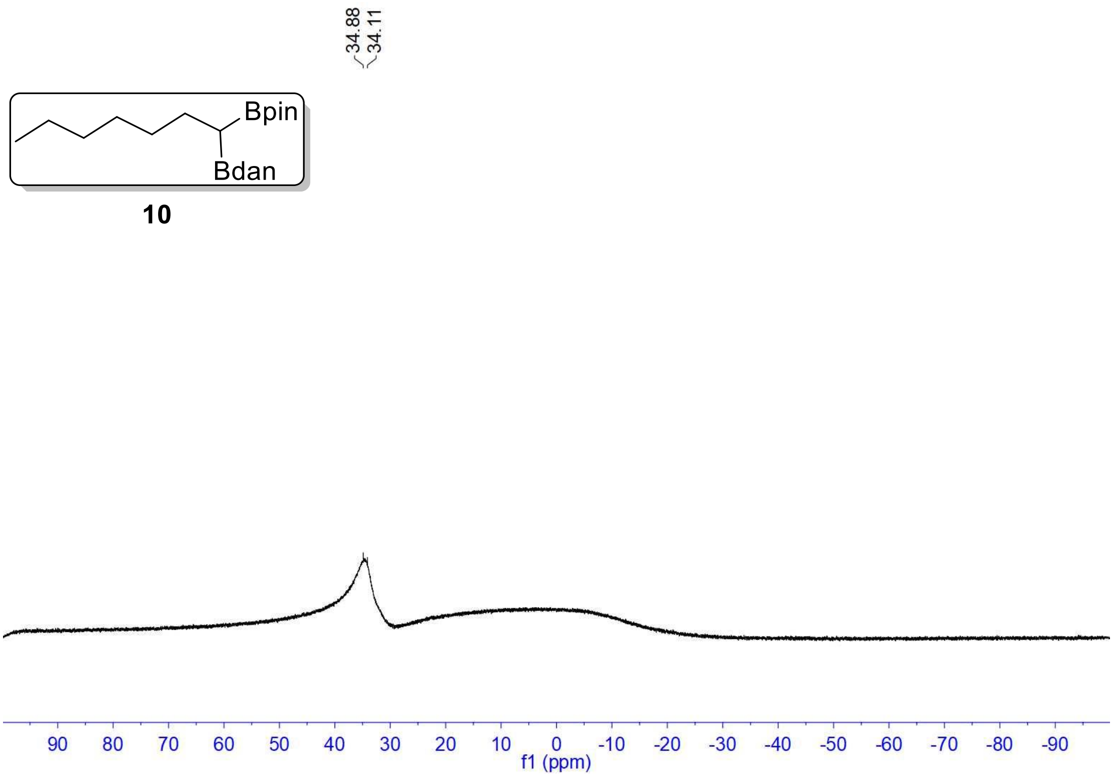

Supplementary Figure 39. ^{11}B NMR spectrum of compound 10

2-(1-(4,4,5,5-tetramethyl-1,3,2-dioxaborolan-2-yl)octyl) -2,3-dihydro-1H-naphtho[1,8-de][1,3,2]diazaborinine (11)

^{1}H NMR (500 MHz, room temperature, CDCl3)

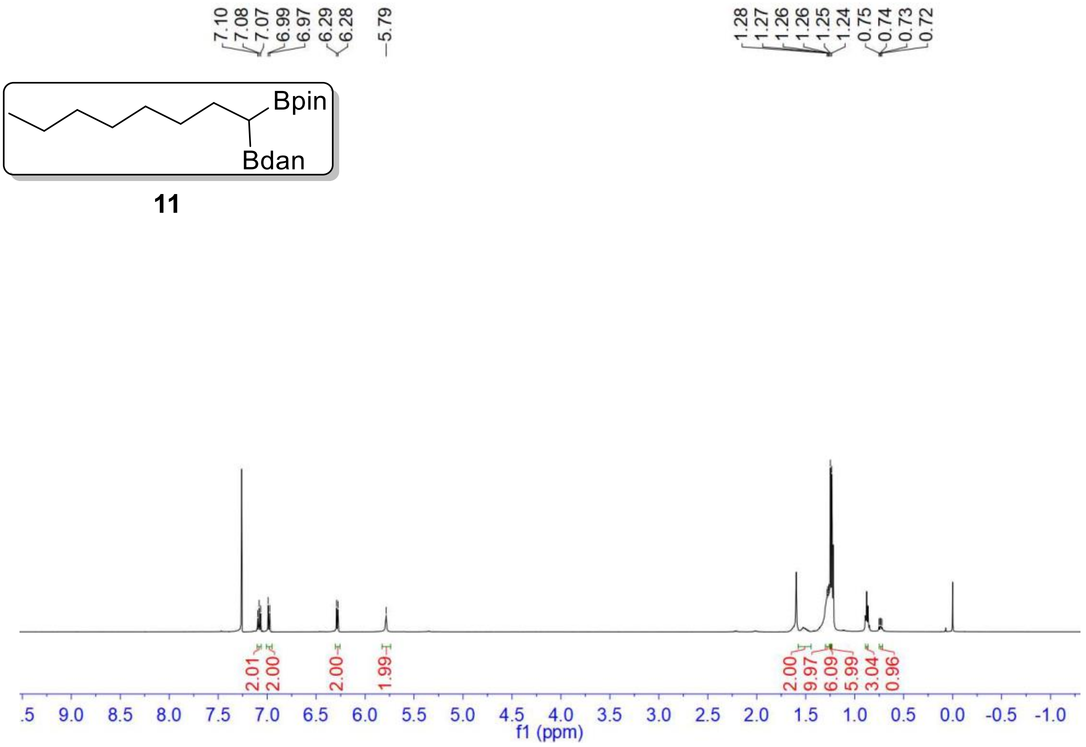

Supplementary Figure 40. ^{1}H NMR spectrum of compound 11

^{13}C NMR (126 MHz, room temperature, $CDCl_3$)

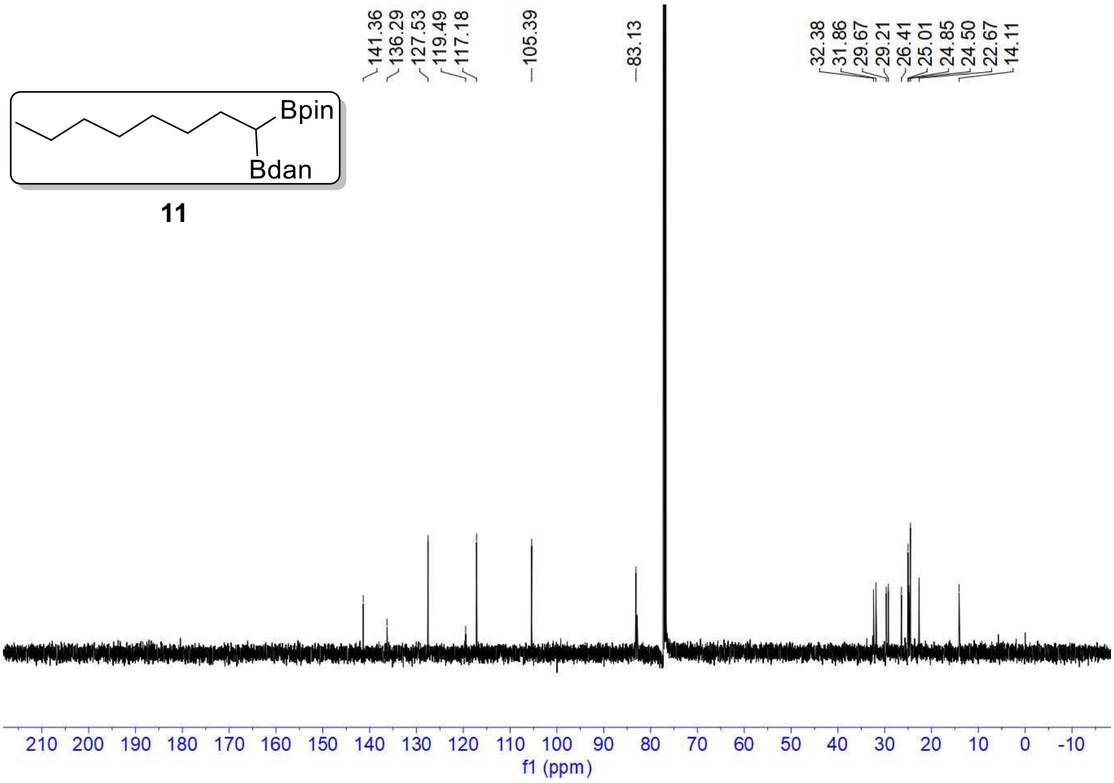

Supplementary Figure 41. ^{13}C NMR spectrum of compound 11

^{11}B NMR (160 MHz, room temperature, $CDCl_3$)

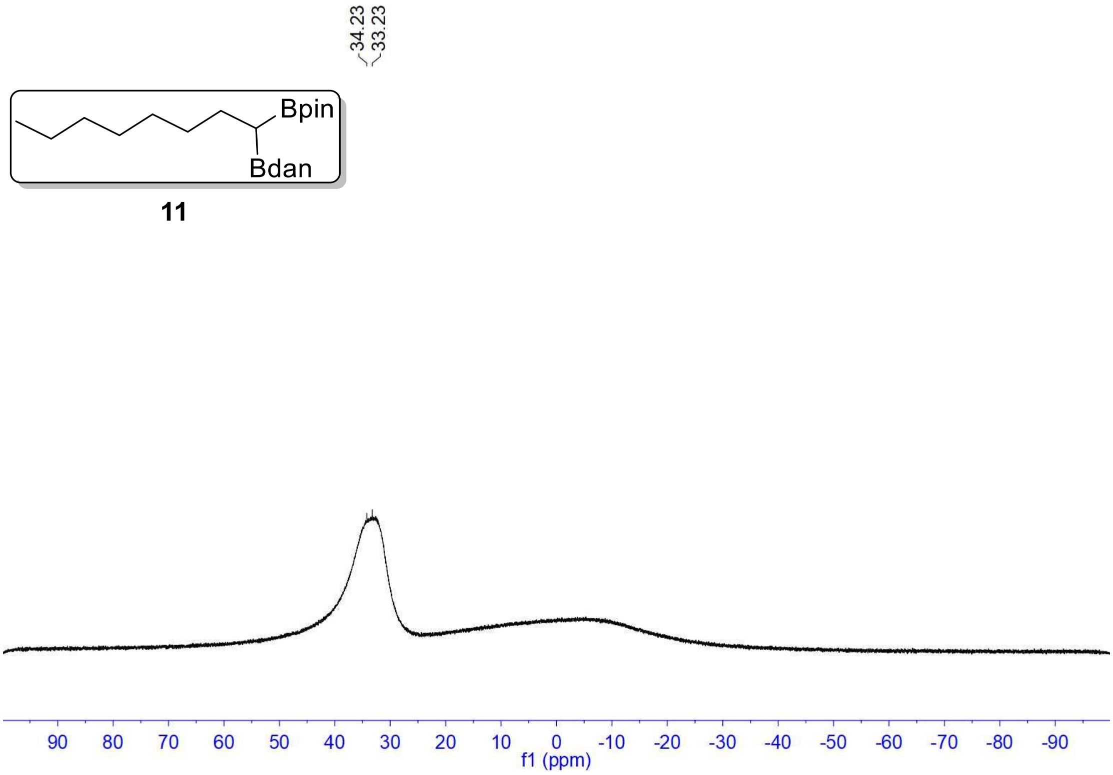

Supplementary Figure 42. ^{11}B NMR spectrum of compound 11

2-(1-(4,4,5,5-tetramethyl-1,3,2-dioxaborolan-2-yl)octyl)-2,3-dihydro-1H-naphtho[1,8-de][1,3,2]diazaborinine (12)

^{1}H NMR (500 MHz, room temperature, $CDCl_3$)

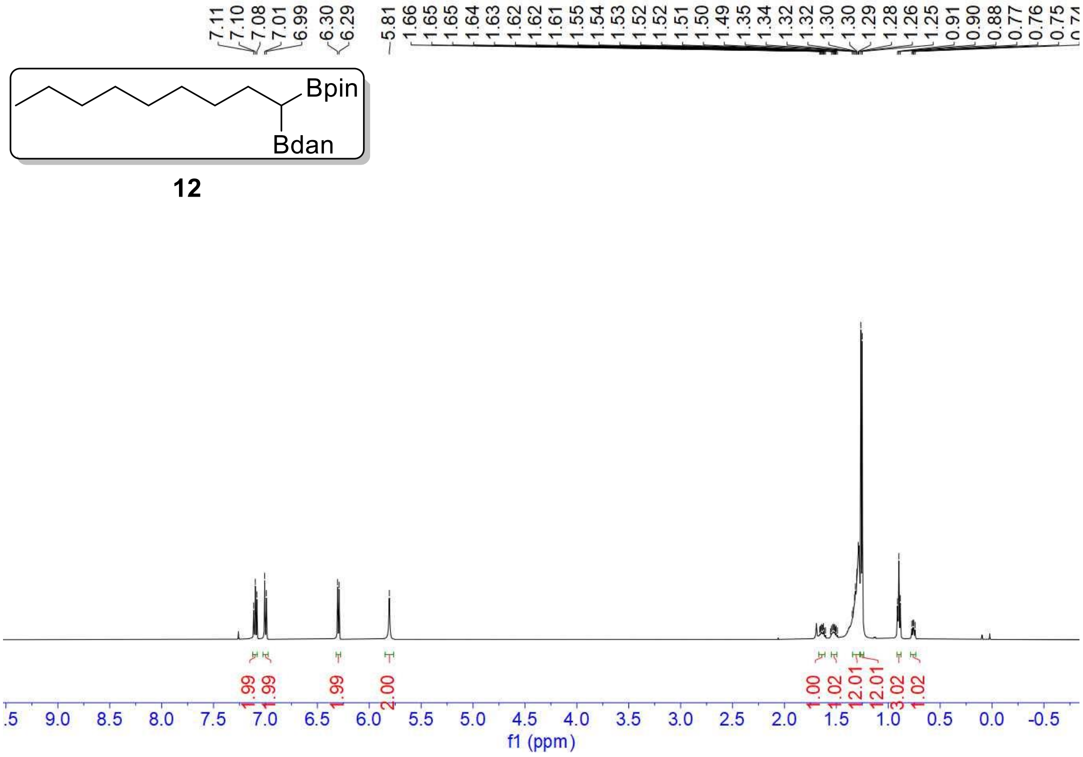

Supplementary Figure 43. ^{1}H NMR spectrum of compound 12

^{13}C NMR (126 MHz, room temperature, $CDCl_3$)

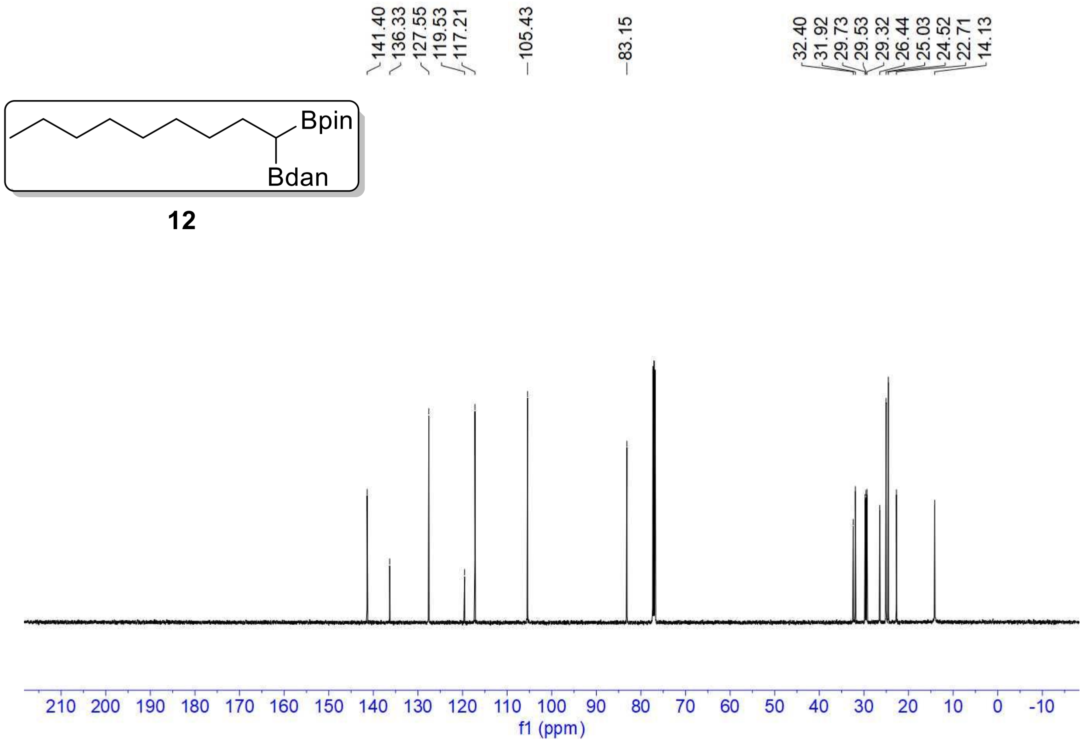

Supplementary Figure 44. ^{13}C NMR spectrum of compound 12

^{11}B NMR (160 MHz, room temperature, CDCl$_3$)

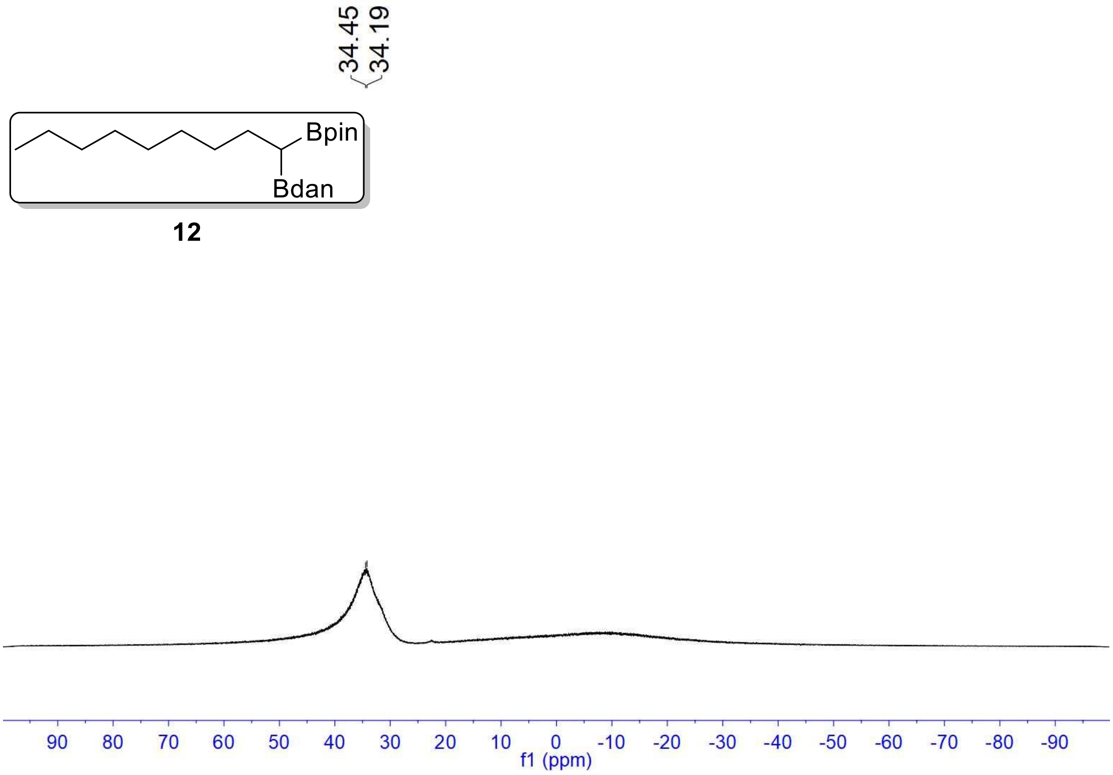

Supplementary Figure 45. ^{11}B NMR spectrum of compound 12

2-(5-methyl-1-(4,4,5,5-tetramethyl-1,3,2-dioxaborolan-2-yl)hexyl)-2,3-dihydro-1H-naphtho[1,8-de][1,3,2]diazaborinine (13)

^{1}H NMR (500 MHz, room temperature, CDCl$_3$)

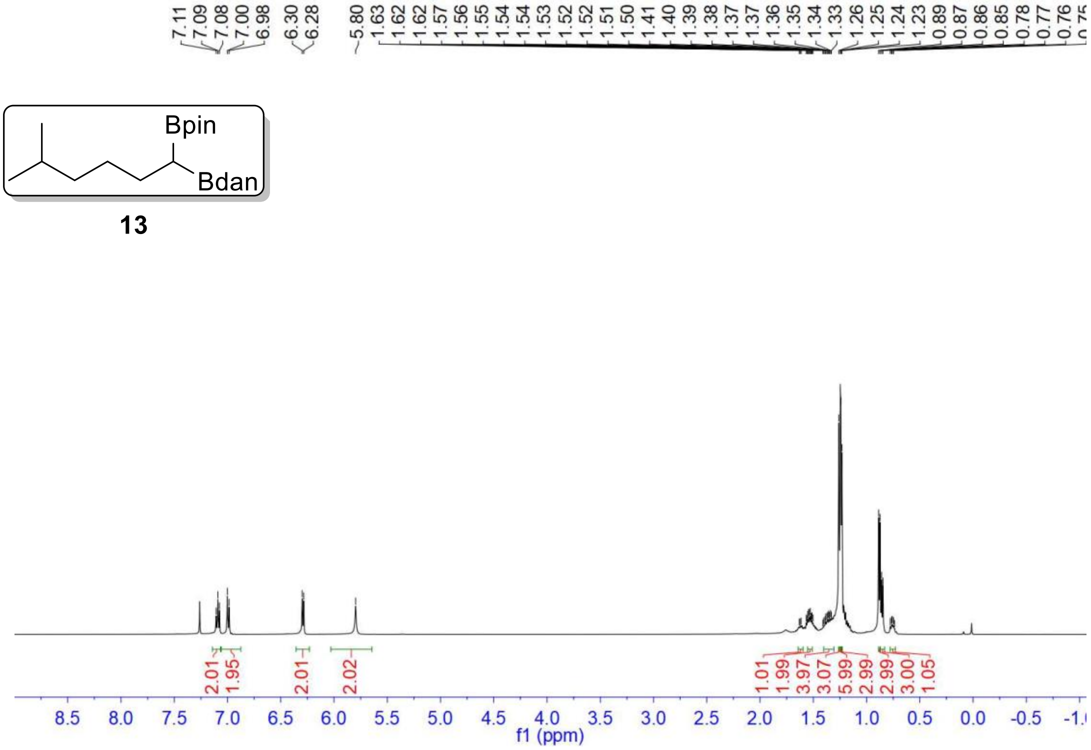

Supplementary Figure 46. ^{1}H NMR spectrum of compound 13

^{13}C NMR (126 MHz, room temperature, $CDCl_3$)

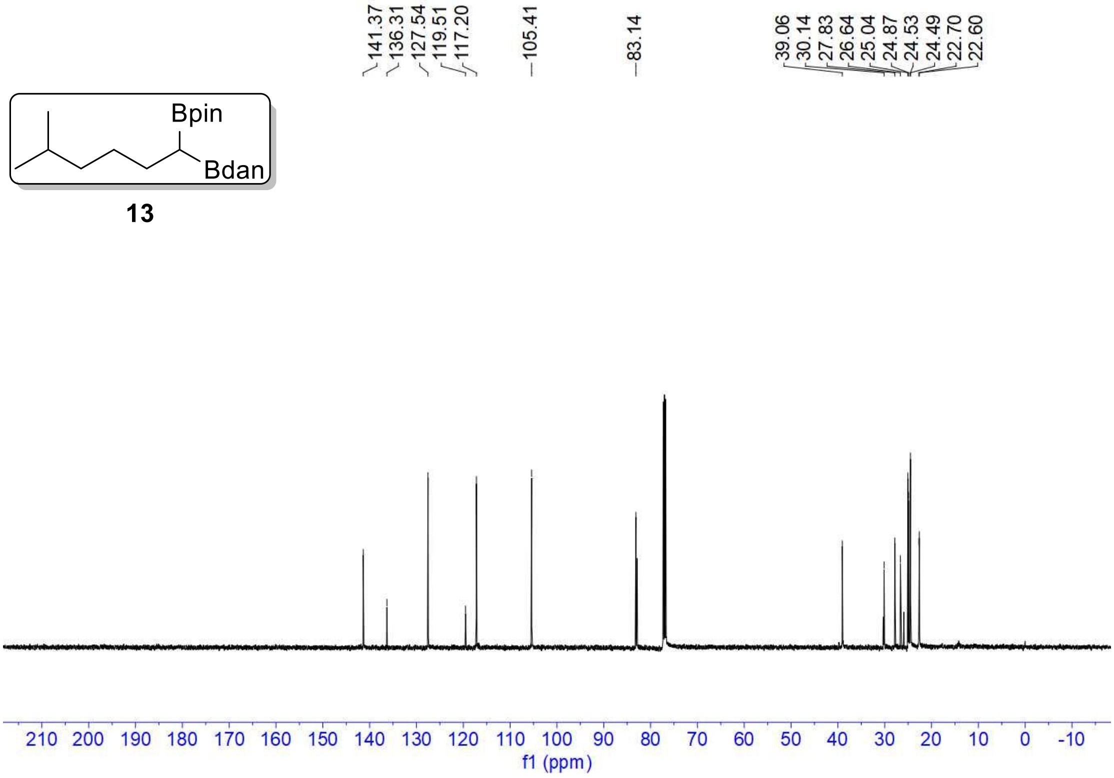

Supplementary Figure 47. ^{13}C NMR spectrum of compound 13

^{11}B NMR (160 MHz, room temperature, $CDCl_3$)

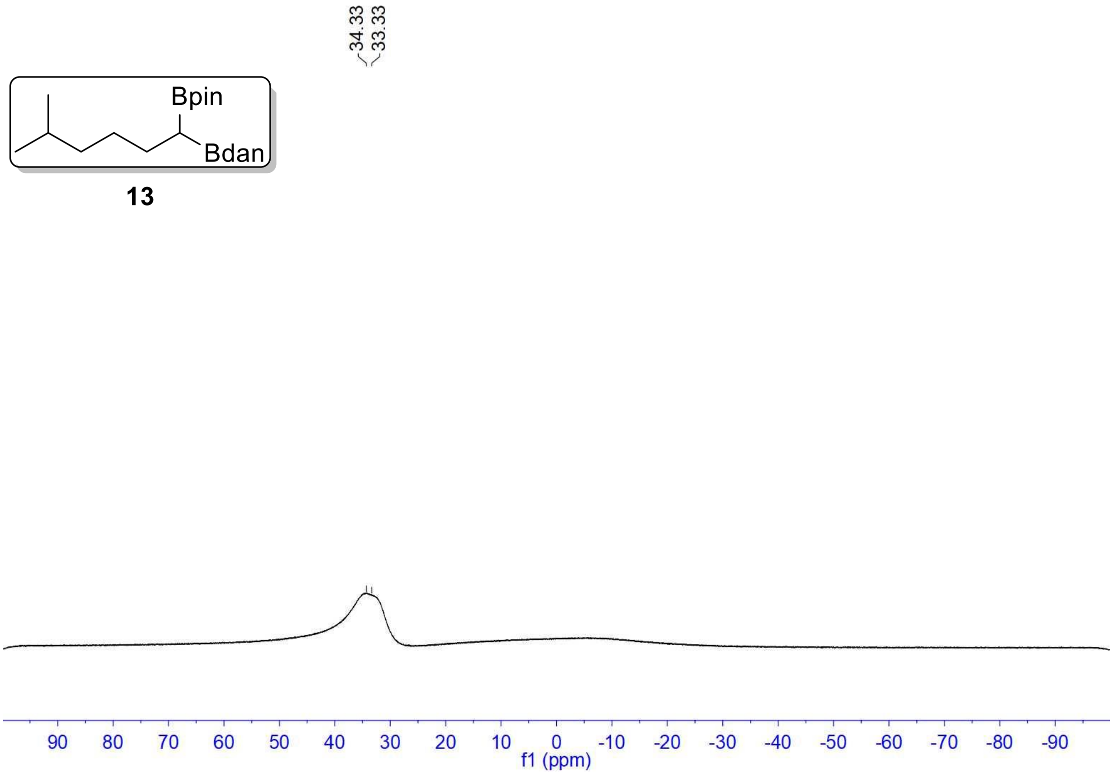

Supplementary Figure 48. ^{11}B NMR spectrum of compound 13

2-(3,3-dimethyl-1-(4,4,5,5-tetramethyl-1,3,2-dioxaborolan-2-yl)butyl)-2,3-dihydro-1H-naphtho[1,8-de][1,3,2]diazaborinine (14)

^{1}H NMR (500 MHz, room temperature, CDCl$_3$)

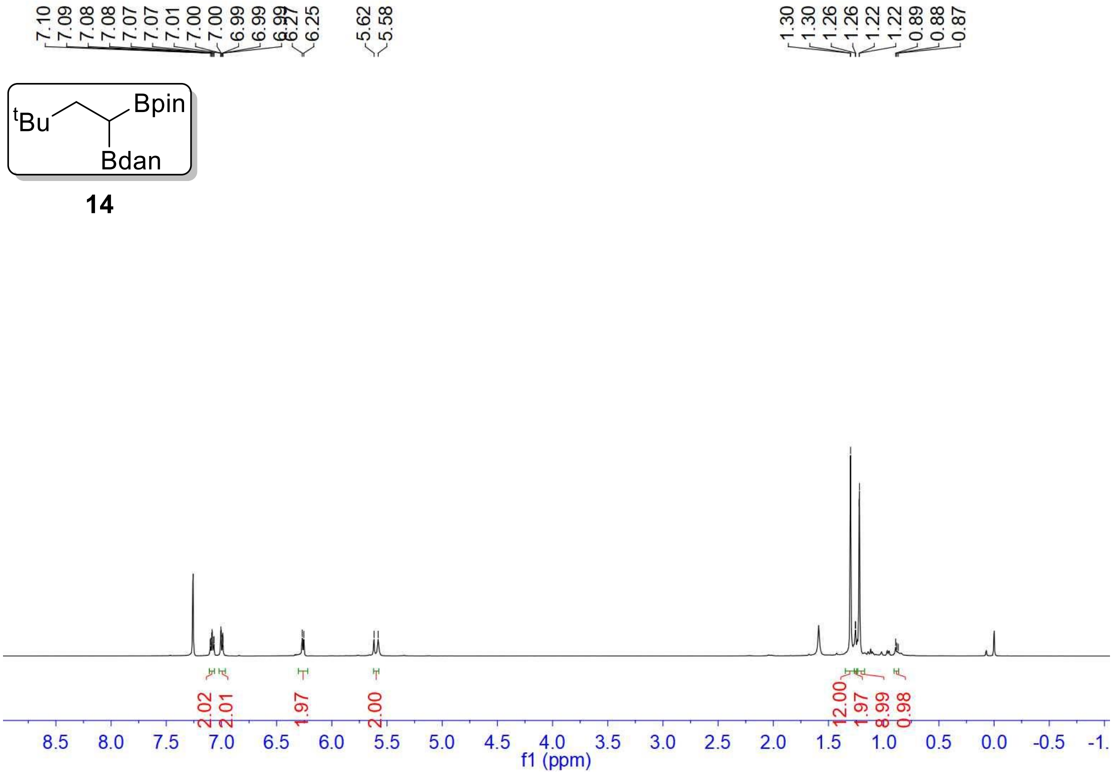

Supplementary Figure 49. ^{1}H NMR spectrum of compound 14

^{13}C NMR (126 MHz, room temperature, CDCl$_3$)

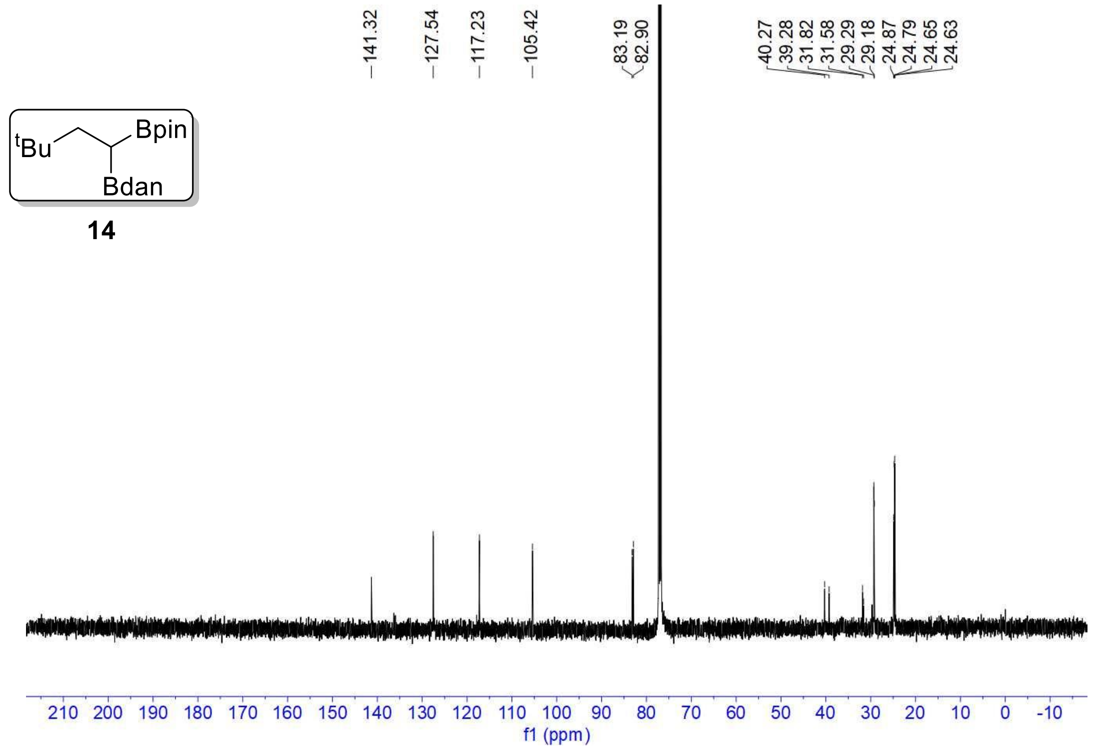

Supplementary Figure 50. ^{13}C NMR spectrum of compound 14

^{11}B NMR (160 MHz, room temperature, $CDCl_3$)

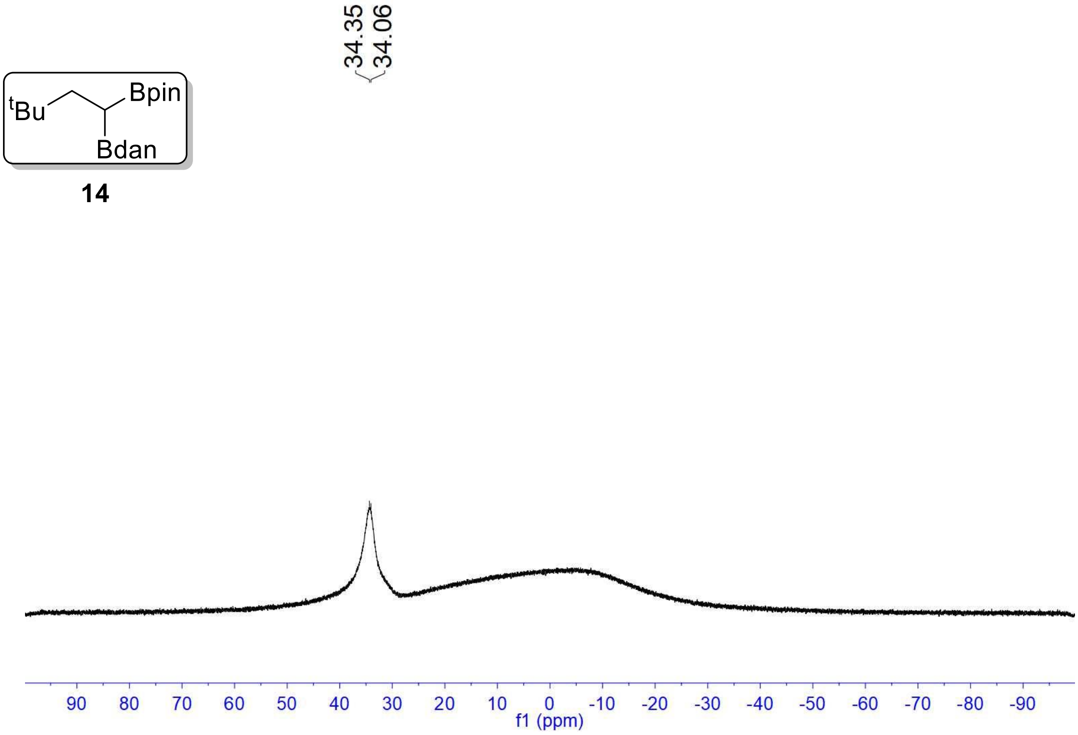

Supplementary Figure 51. ^{11}B NMR spectrum of compound 14

2-(6-((tert-butyldimethylsilyl)oxy)-1-(4,4,5,5-tetramethyl-1,3,2-dioxaborolan-2-yl)hexyl)-2,3-dihydro-1H-naphtho[1,8-de][1,3,2]diazaborinine (15)

^{1}H NMR (500 MHz, room temperature, $CDCl_3$)

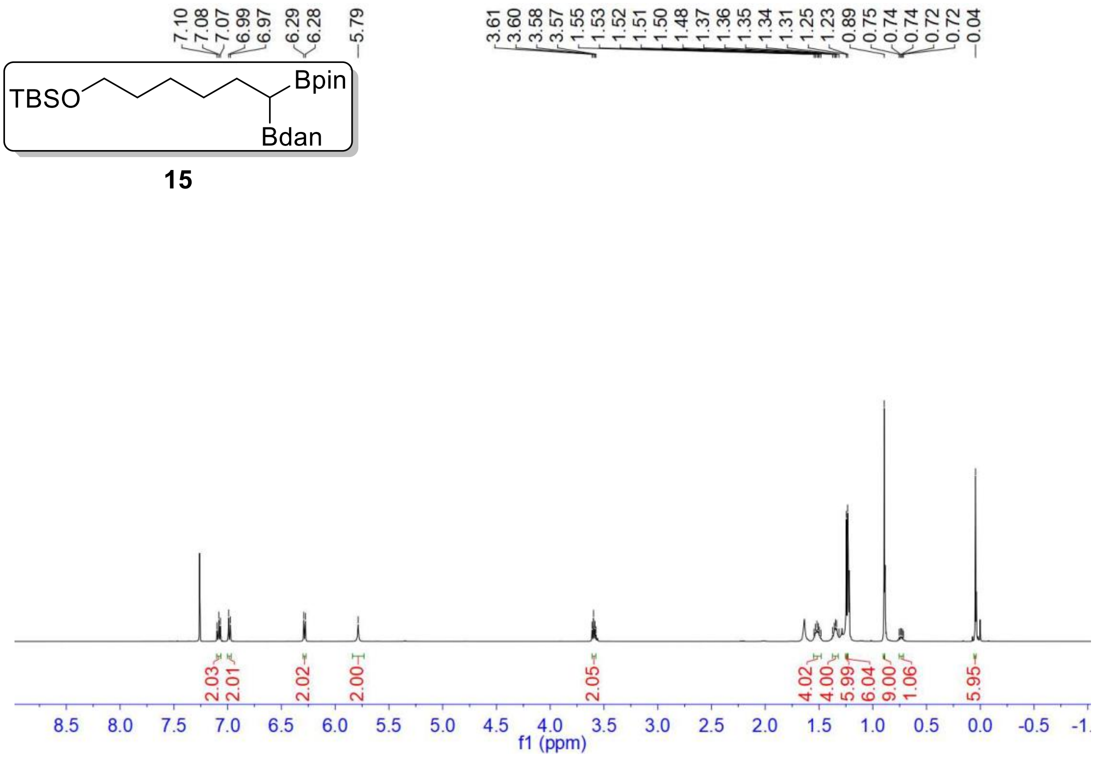

Supplementary Figure 52. ^{1}H NMR spectrum of compound 15

^{13}C NMR (126 MHz, room temperature, $CDCl_3$)

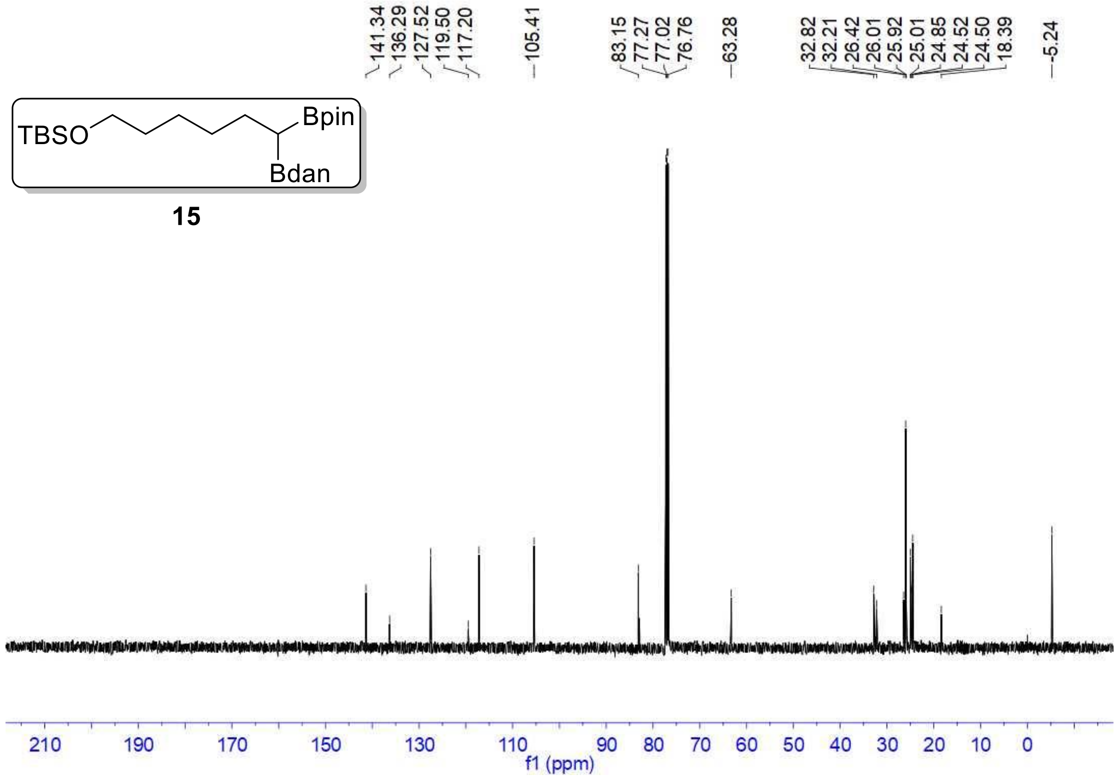

Supplementary Figure 53. ^{13}C NMR spectrum of compound 15

^{11}B NMR (160 MHz, room temperature, $CDCl_3$)

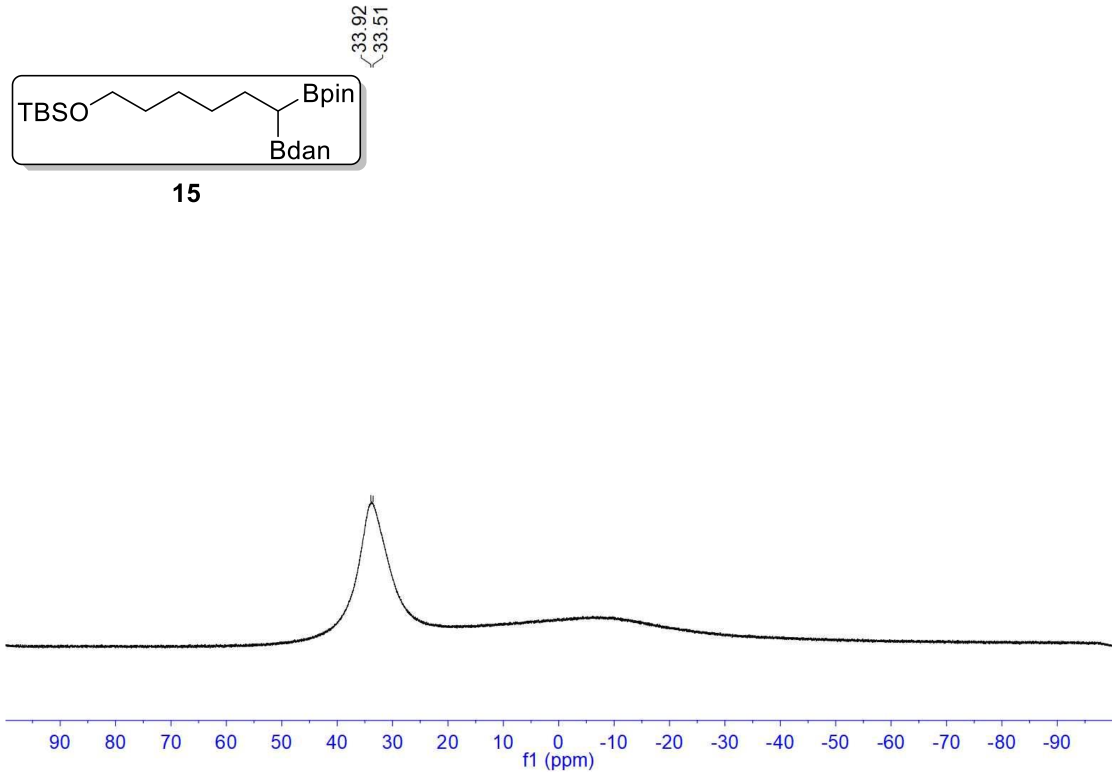

Supplementary Figure 54. ^{11}B NMR spectrum of compound 15

2-(4-((tert-butyldimethylsilyl)oxy)-1-(4,4,5,5-tetramethyl -1,3,2-dioxaborolan-2-yl)butyl)-2,3-dihydro-1H-naphtho[1,8-de][1,3,2]diazaborinine (16)

^{1}H NMR (500 MHz, room temperature, $CDCl_3$)

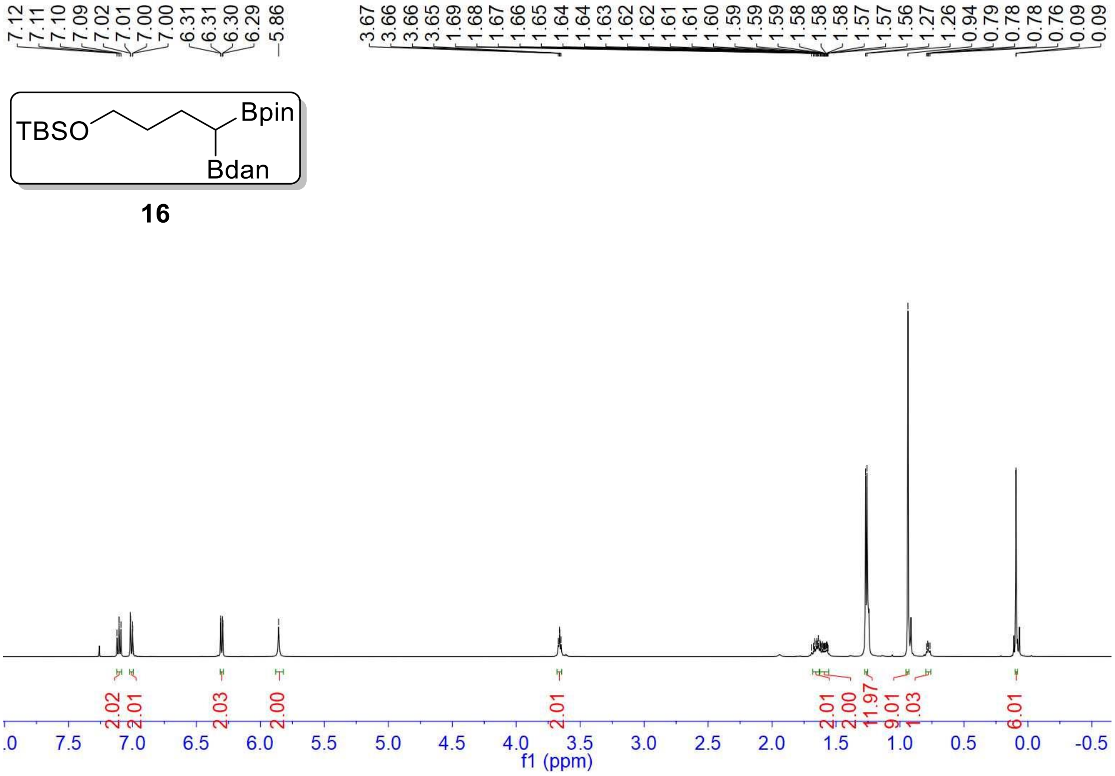

Supplementary Figure 55. ^{1}H NMR spectrum of compound 16

^{13}C NMR (126 MHz, room temperature, $CDCl_3$)

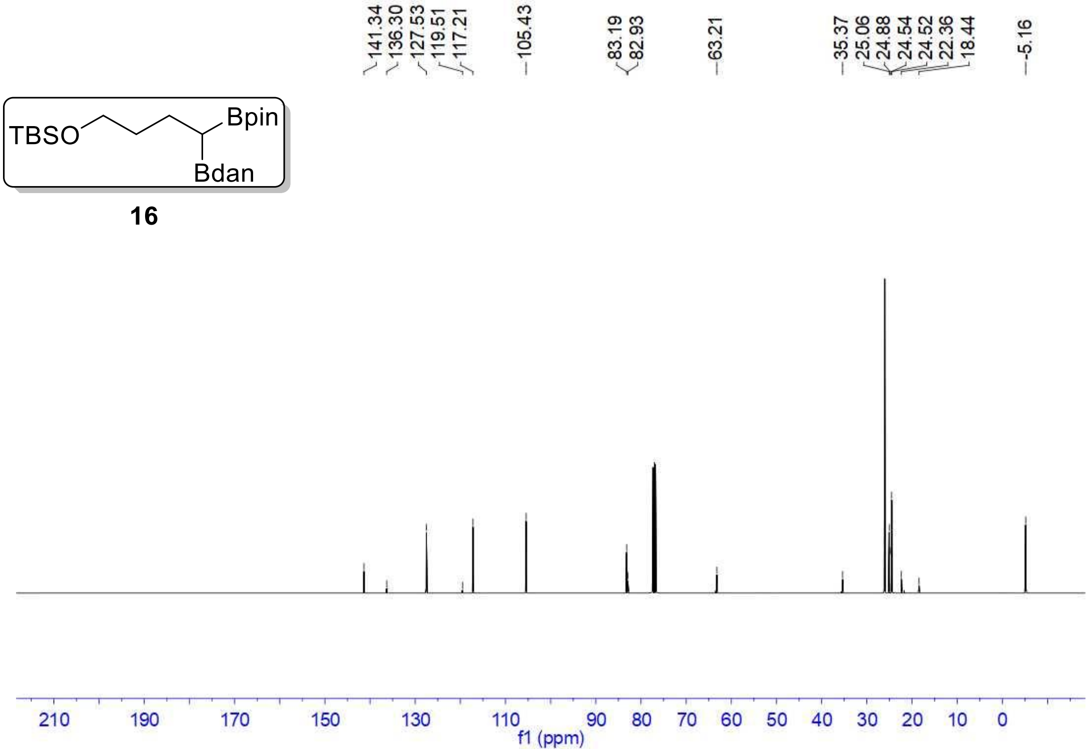

Supplementary Figure 56. ^{13}C NMR spectrum of compound 16

^{11}B NMR (160 MHz, room temperature, CDCl$_3$)

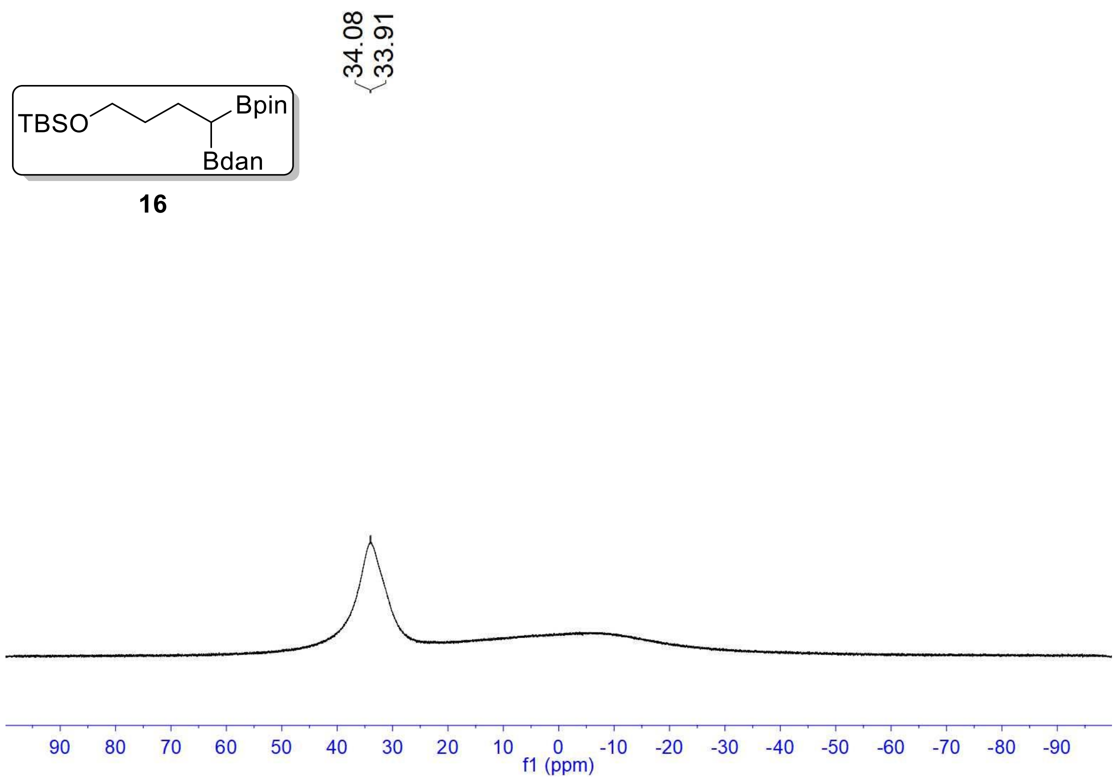

Supplementary Figure 57. ^{11}B NMR spectrum of compound 16

tert-butyl 4-(2-(1H-naphtho[1,8-de] [1,3,2] diazaborinin-2(3H)-yl) -2-(4,4,5,5-tetramethyl-1,3,2-dioxaborolan-2-yl) ethyl) piperidine-1-carboxylate (17)

^{1}H NMR (500 MHz, room temperature, CDCl$_3$)

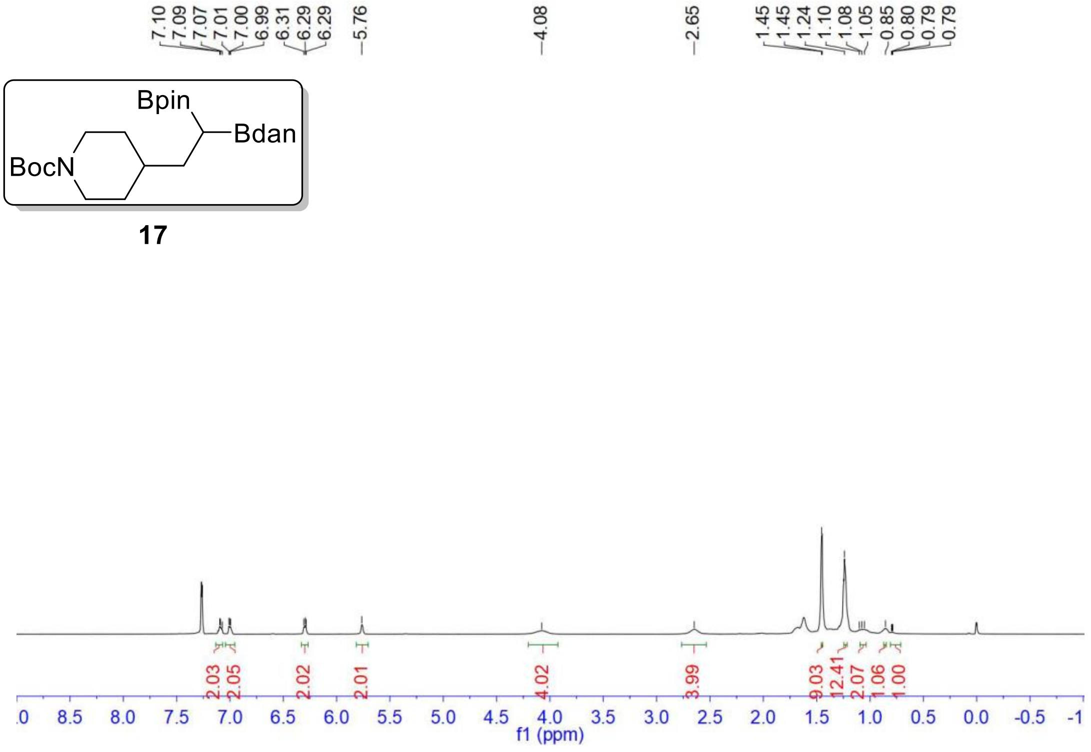

Supplementary Figure 58. ^{1}H NMR spectrum of compound 17

^{13}C NMR (126 MHz, room temperature, $CDCl_3$)

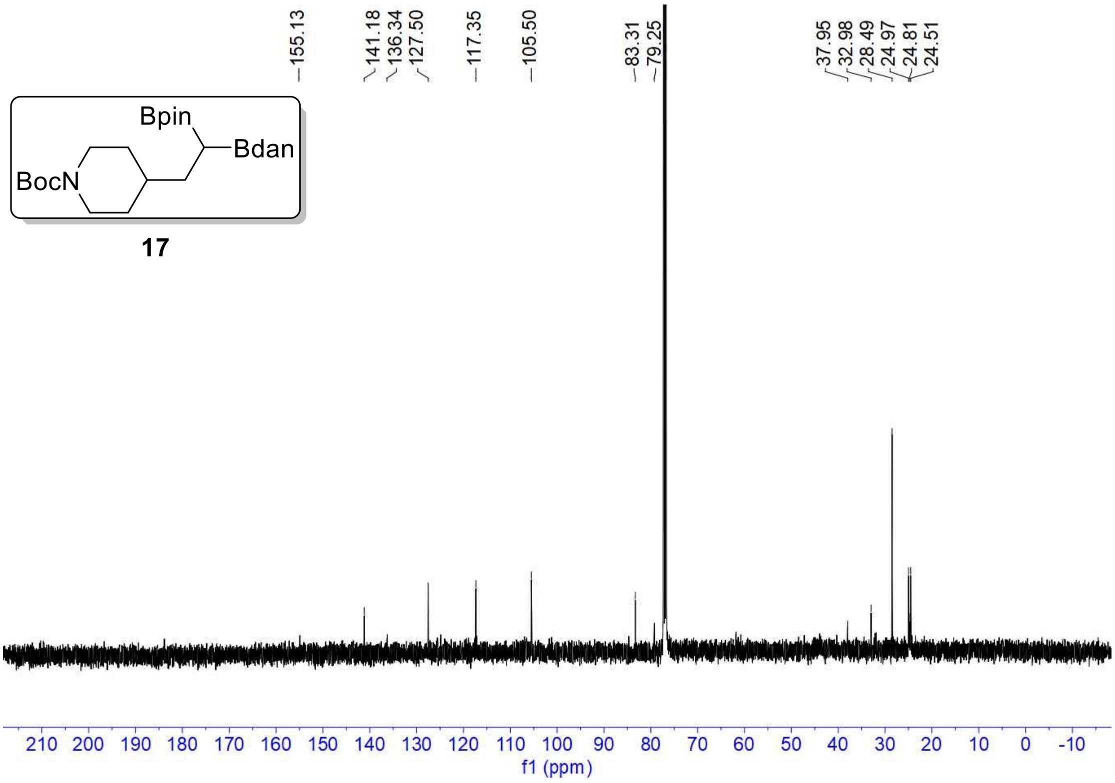

Supplementary Figure 59. ^{13}C NMR spectrum of compound 17

^{11}B NMR (160 MHz, room temperature, $CDCl_3$)

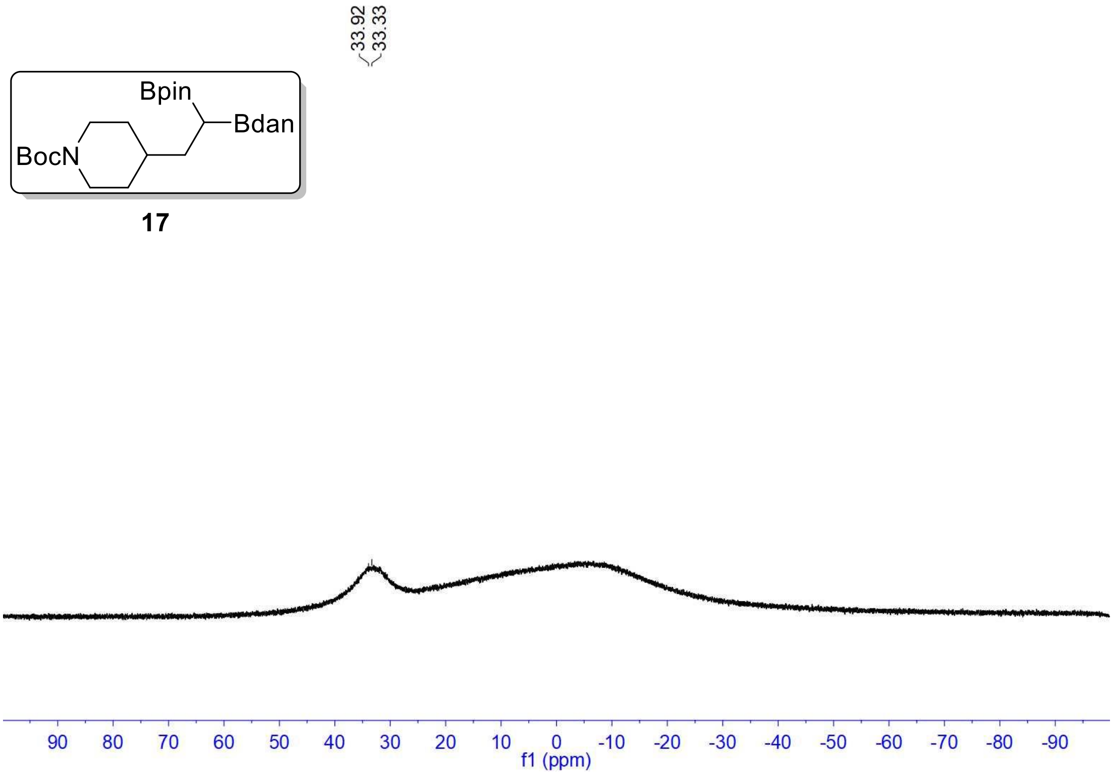

Supplementary Figure 60. ^{11}B NMR spectrum of compound 17

2-(2-(cyclohex-1-en-1-yl)-1-(4,4,5,5-tetramethyl- 1,3,2-dioxaborolan-2-yl)ethyl)-2,3-dihydro-1H-naphtho[1,8-de][1,3,2]diazaborinine (18)

^{1}H NMR (500 MHz, room temperature, $CDCl_3$)

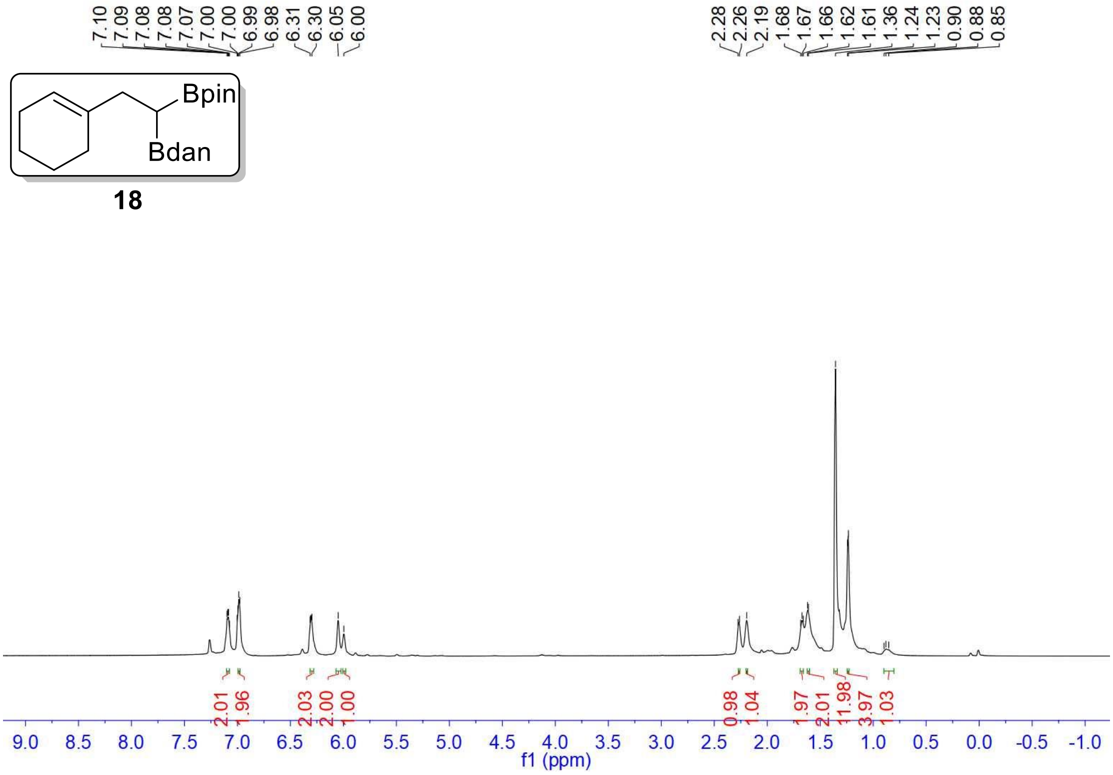

Supplementary Figure 61. ^{1}H NMR spectrum of compound 18

^{13}C NMR (126 MHz, room temperature, $CDCl_3$)

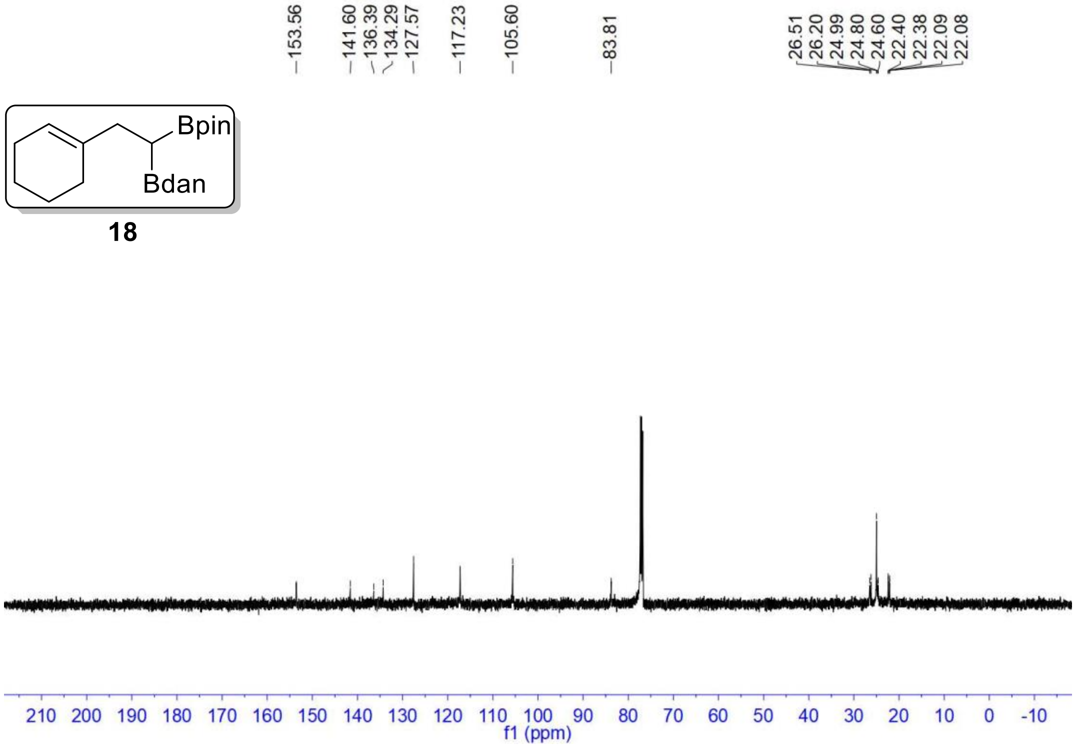

Supplementary Figure 62. ^{13}C NMR spectrum of compound 18

^{11}B NMR (160 MHz, room temperature, $CDCl_3$)

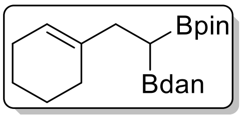

18

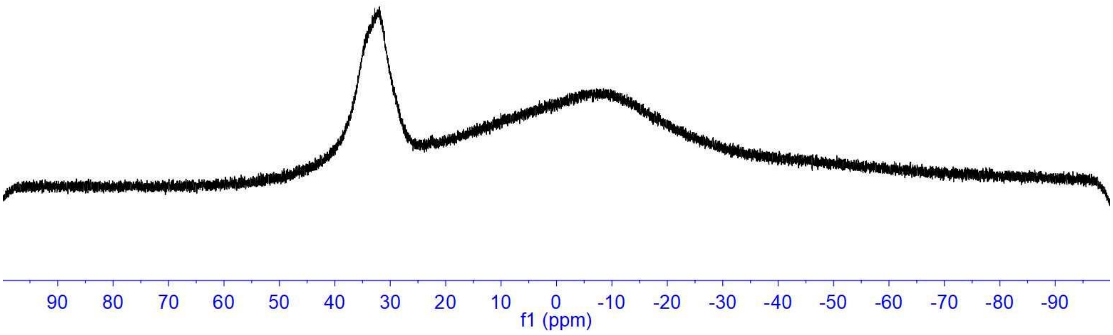

Supplementary Figure 63. ^{11}B NMR spectrum of compound 18

5-(1H-naphtho[1,8-de][1,3,2]diazaborinin-2(3H)-yl) -5-(4,4,5,5-tetramethyl-1,3,2-dioxaborolan-2-yl)pentyl propionate (19)

^{1}H NMR (500 MHz, room temperature, $CDCl_3$)

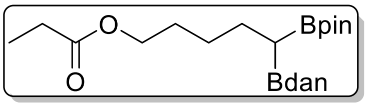

19

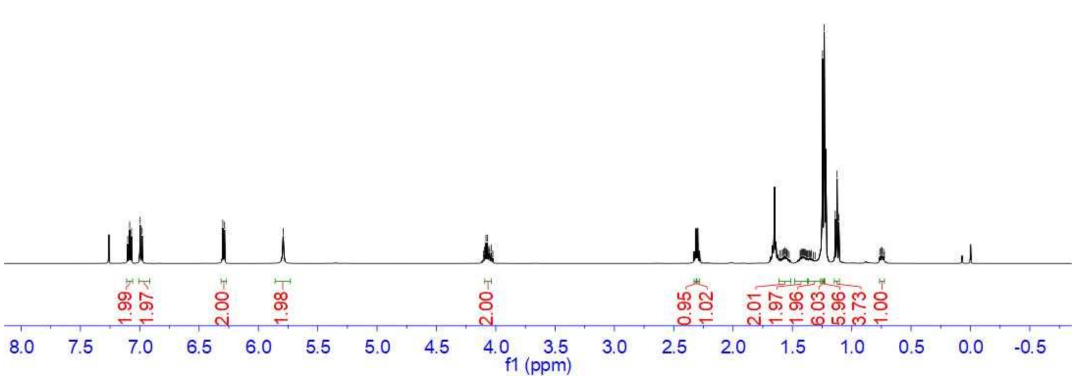

Supplementary Figure 64. ^{1}H NMR spectrum of compound 19

^{13}C NMR (126 MHz, room temperature, $CDCl_3$)

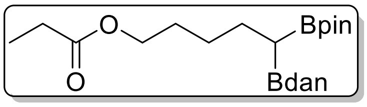

19

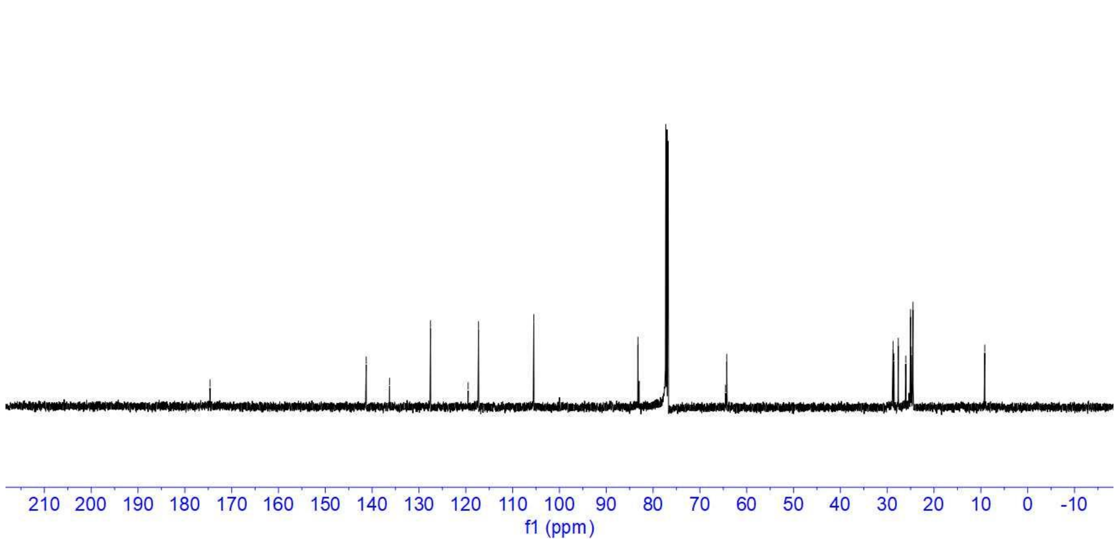

Supplementary Figure 65. ^{13}C NMR spectrum of compound 19

^{11}B NMR (160 MHz, room temperature, $CDCl_3$)

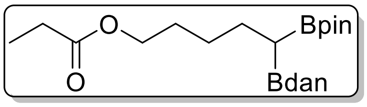

19

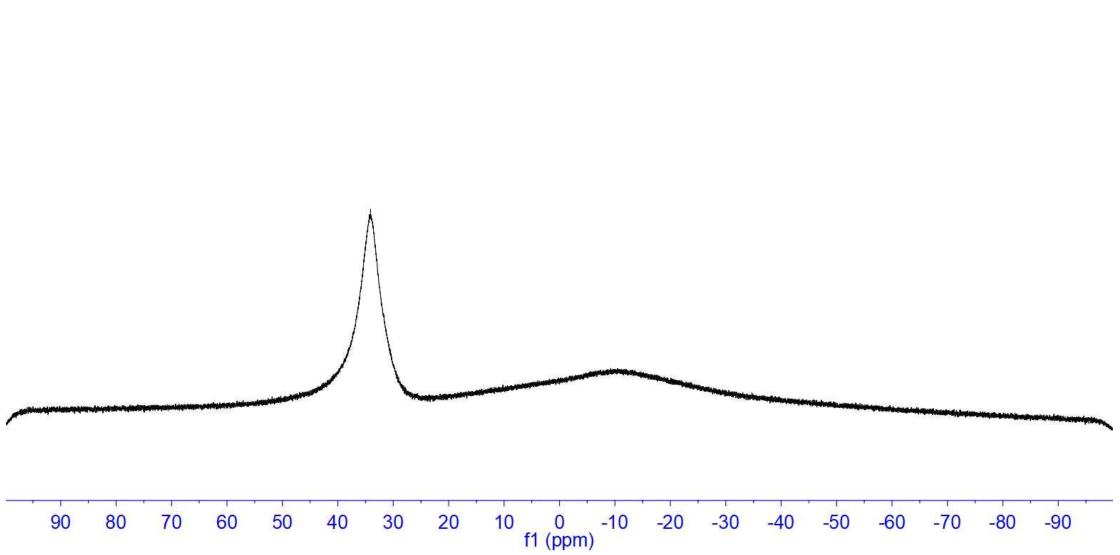

Supplementary Figure 66. ^{11}B NMR spectrum of compound 19

2-(4-((tetrahydro-2H-pyran-2-yl)oxy) -1-(4,4,5,5-tetramethyl-1,3,2-dioxaborolan-2-yl)butyl)-2,3-dihydro-1H-naphtho[1,8-de][1,3,2]diazaborinine (20)

^{1}H NMR (500 MHz, room temperature, $CDCl_3$)

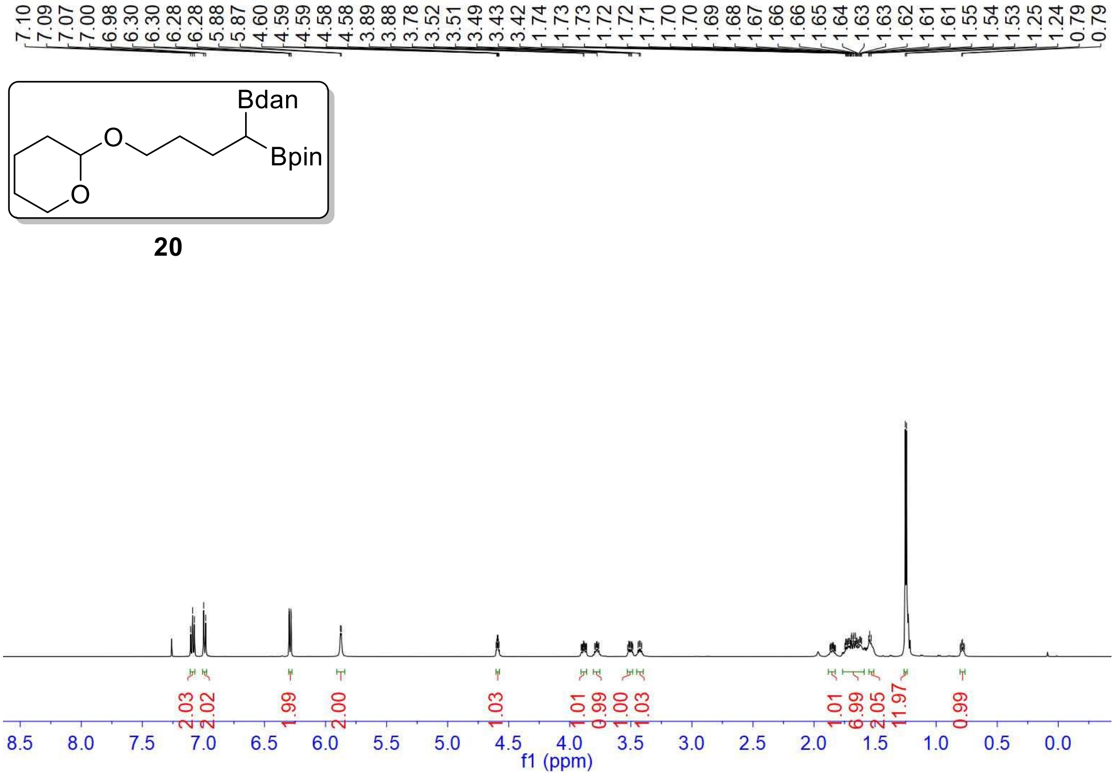

Supplementary Figure 67. ^{1}H NMR spectrum of compound 20

^{13}C NMR (126 MHz, room temperature, $CDCl_3$)

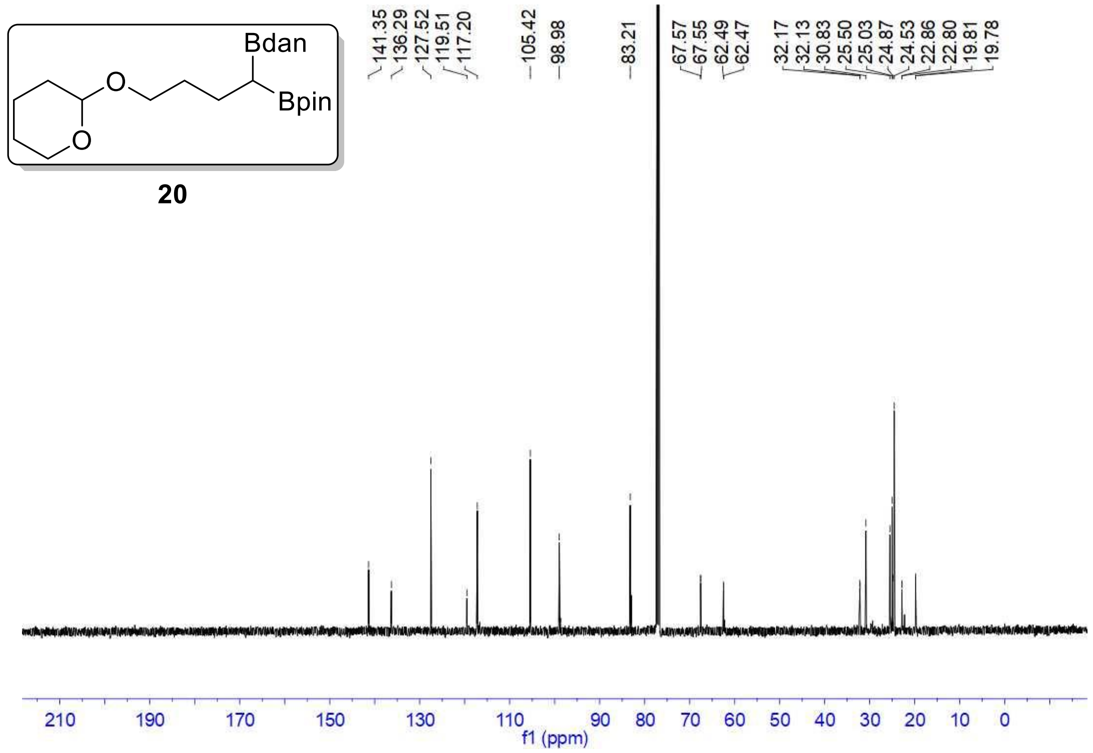

Supplementary Figure 68. ^{13}C NMR spectrum of compound 20

^{11}B NMR (160 MHz, room temperature, CDCl$_3$)

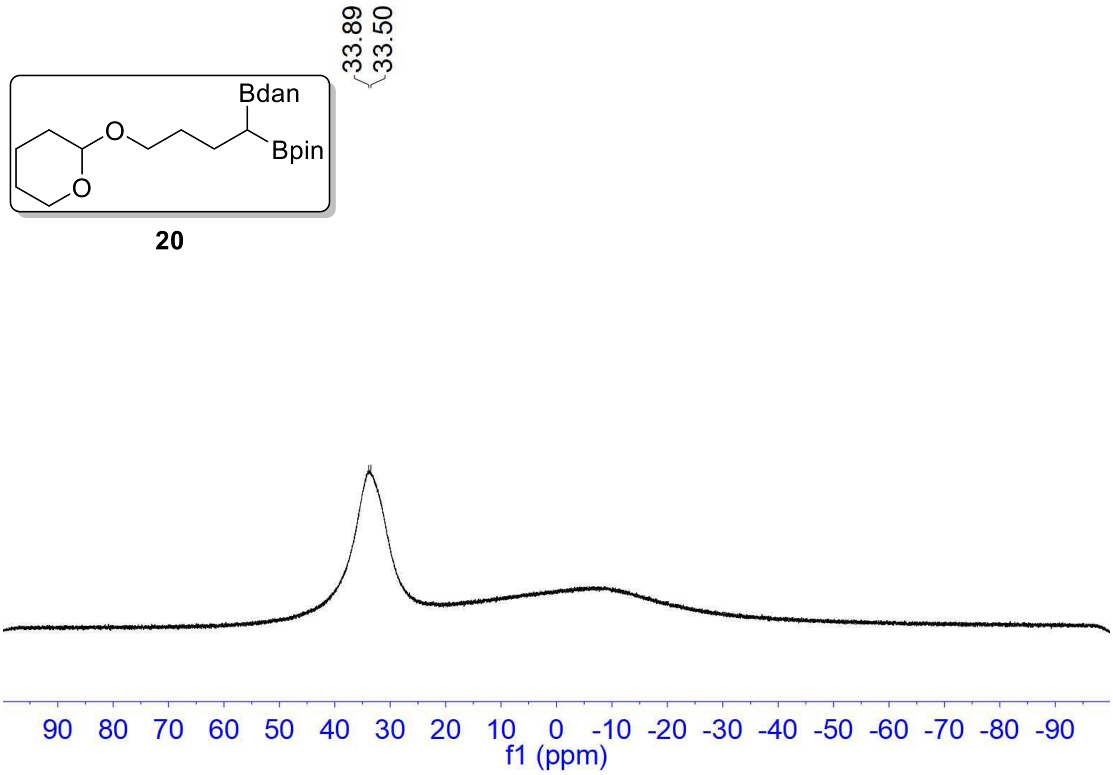

20

Supplementary Figure 69. ^{11}B NMR spectrum of compound 20

2-(3,3-diethoxy-1-(4,4,5,5-tetramethyl-1,3,2-dioxaborolan-2-yl)propyl)-2,3-dihydro-1H-naphtho[1,8-de][1,3,2]diazaborinine (21)

^{1}H NMR (500 MHz, room temperature, CDCl$_3$)

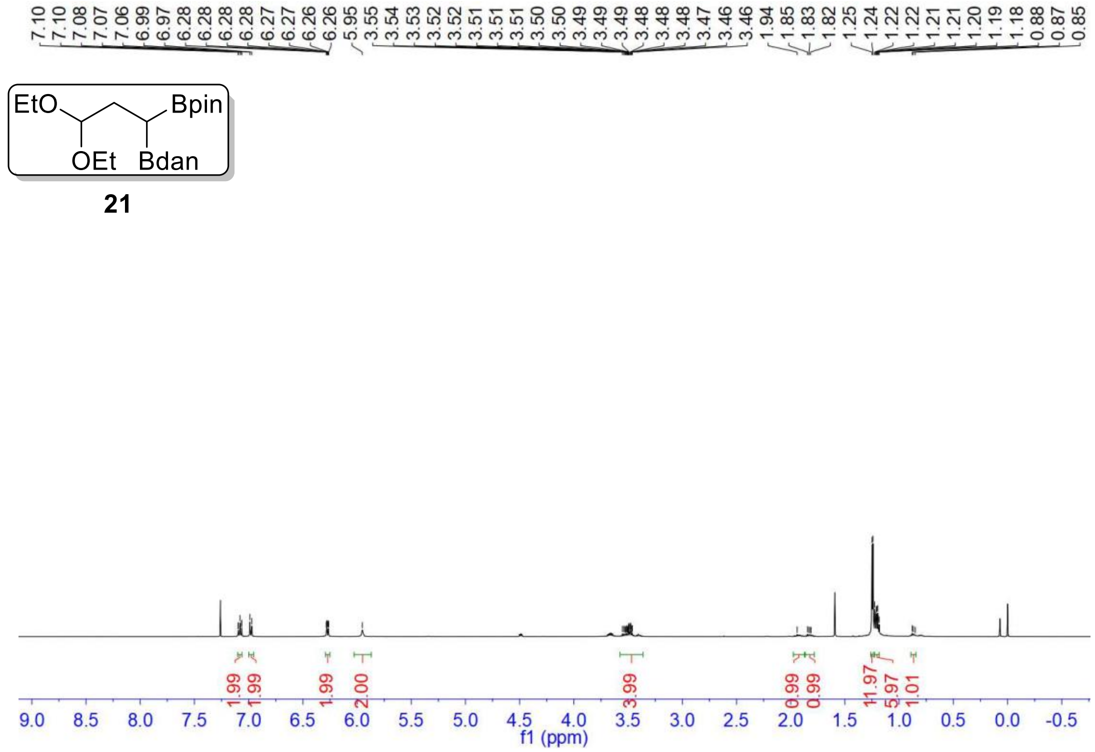

21

Supplementary Figure 70. ^{1}H NMR spectrum of compound 21

^{13}C NMR (126 MHz, room temperature, CDCl$_3$)

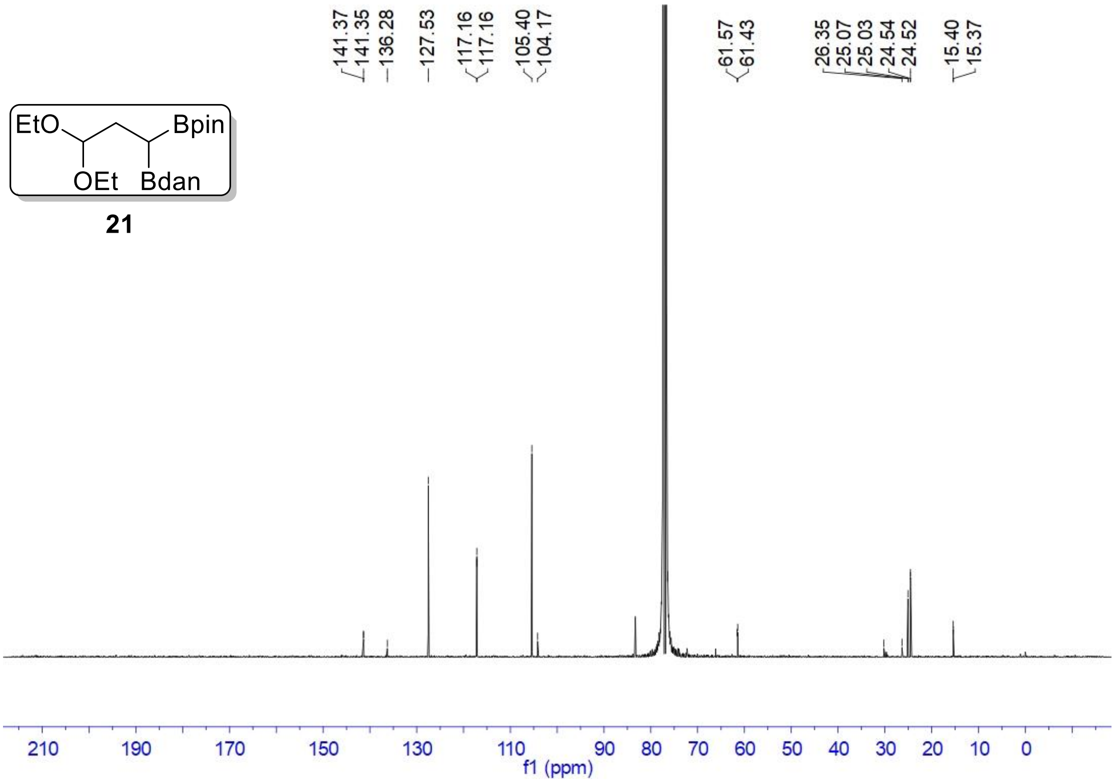

Supplementary Figure 71. ^{13}C NMR spectrum of compound 21

^{11}B NMR (160 MHz, room temperature, CDCl$_3$)

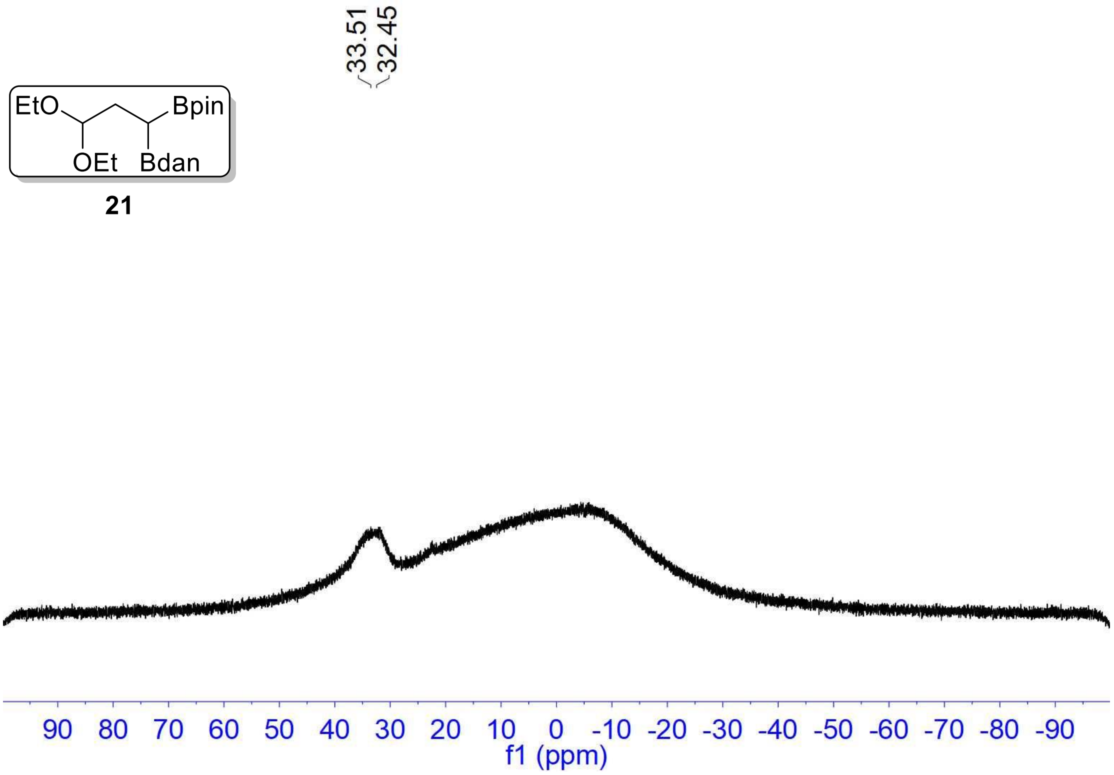

Supplementary Figure 72. ^{11}B NMR spectrum of compound 21

5-(1H-naphtho[1,8-de][1,3,2]diazaborinin-2(3H)-yl)-5-(4,4,5,5-tetramethyl-1,3,2-dioxaborolan-2-yl)pentyl (2E,4E)-hexa-2,4-dienoate (22)

^{1}H NMR (500 MHz, room temperature, CDCl$_3$)

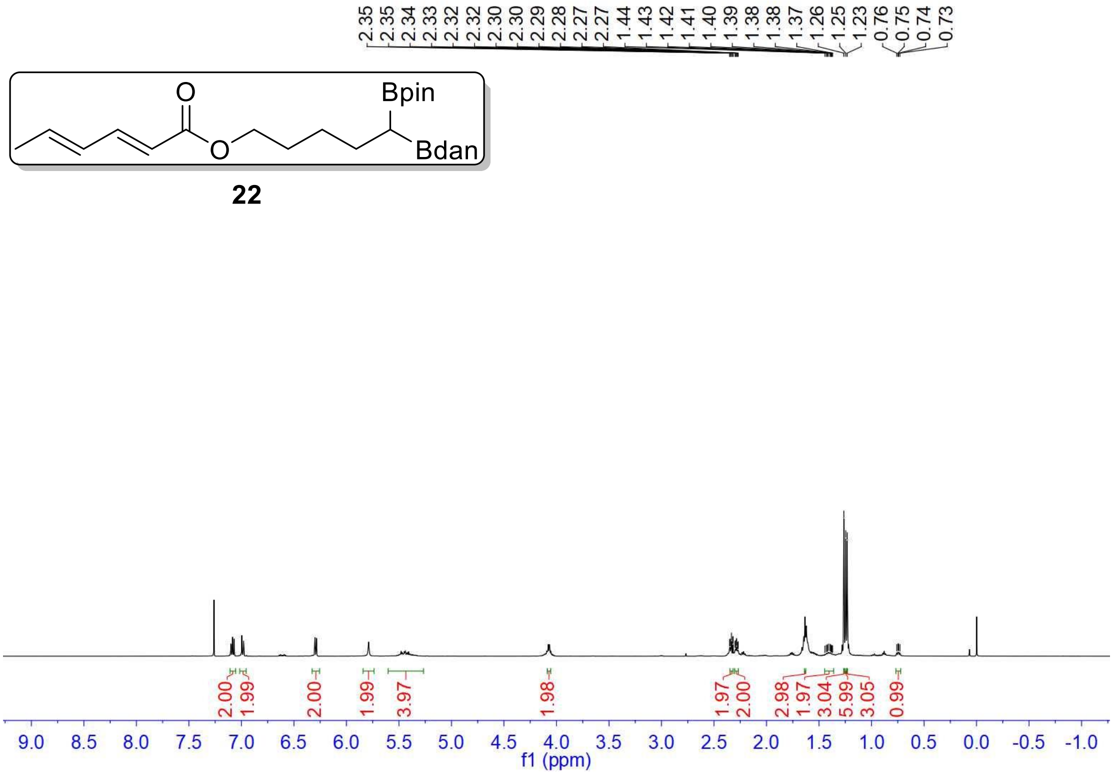

Supplementary Figure 73. ^{1}H NMR spectrum of compound 22

^{13}C NMR (126 MHz, room temperature, CDCl$_3$)

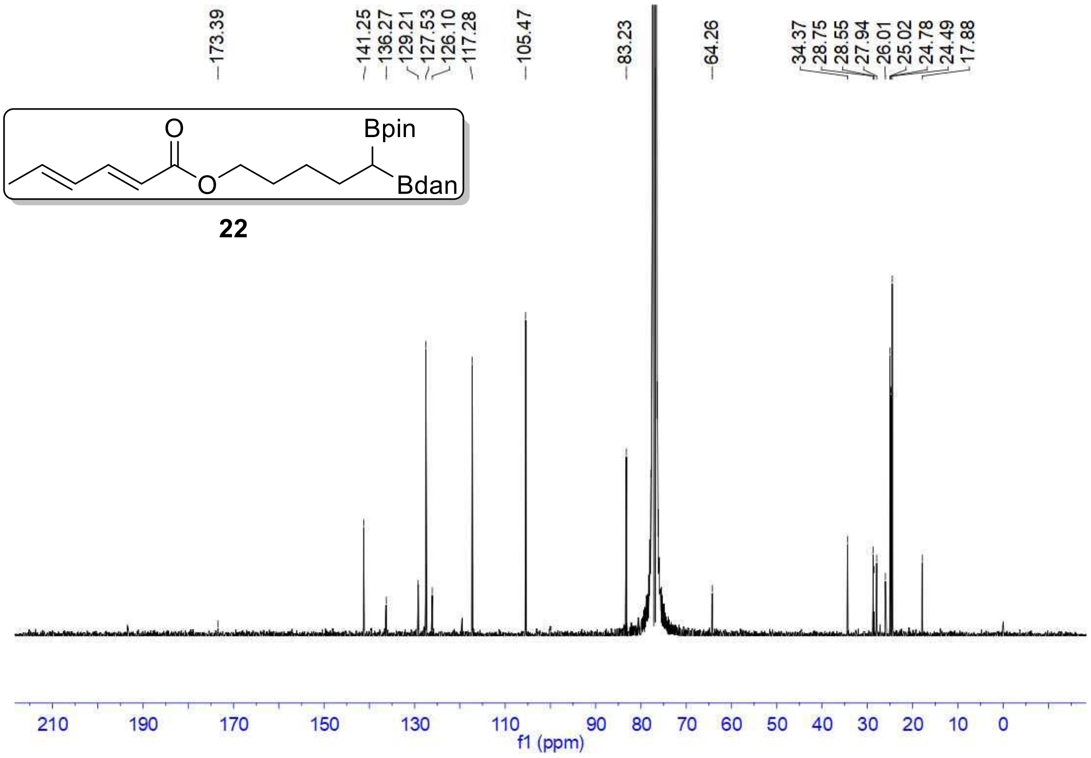

Supplementary Figure 74. ^{13}C NMR spectrum of compound 22

^{11}B NMR (160 MHz, room temperature, $CDCl_3$)

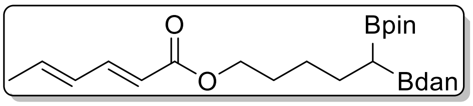

22

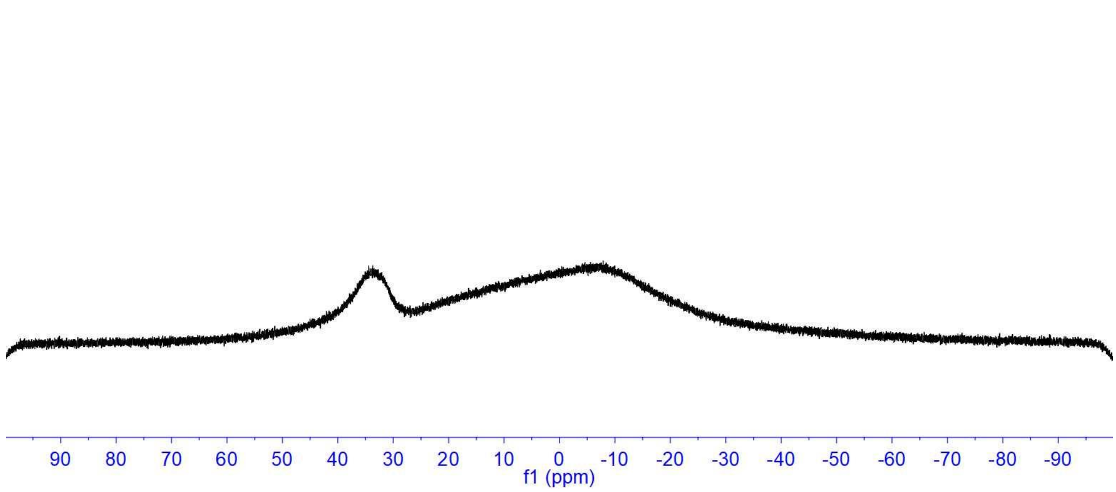

Supplementary Figure 75. ^{11}B NMR spectrum of compound 22

5-(1H-naphtho[1,8-de][1,3,2]diazaborinin-2(3H)-yl)-5-(4,4,5,5-tetramethyl-1,3,2-dioxaborolan-2-yl)pentyl 2-propylpentanoate (23)

^{1}H NMR (500 MHz, room temperature, $CDCl_3$)

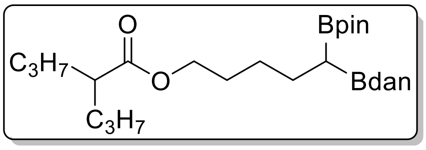

23

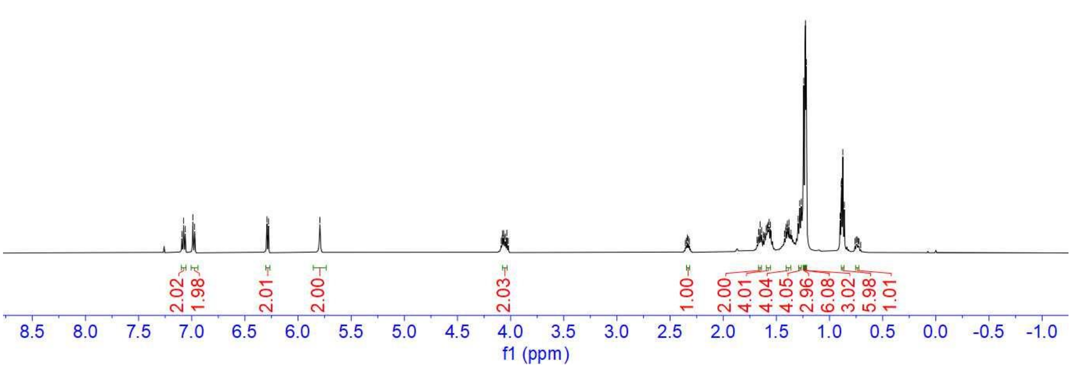

Supplementary Figure 76. ^{1}H NMR spectrum of compound 23

^{13}C NMR (126 MHz, room temperature, $CDCl_3$)

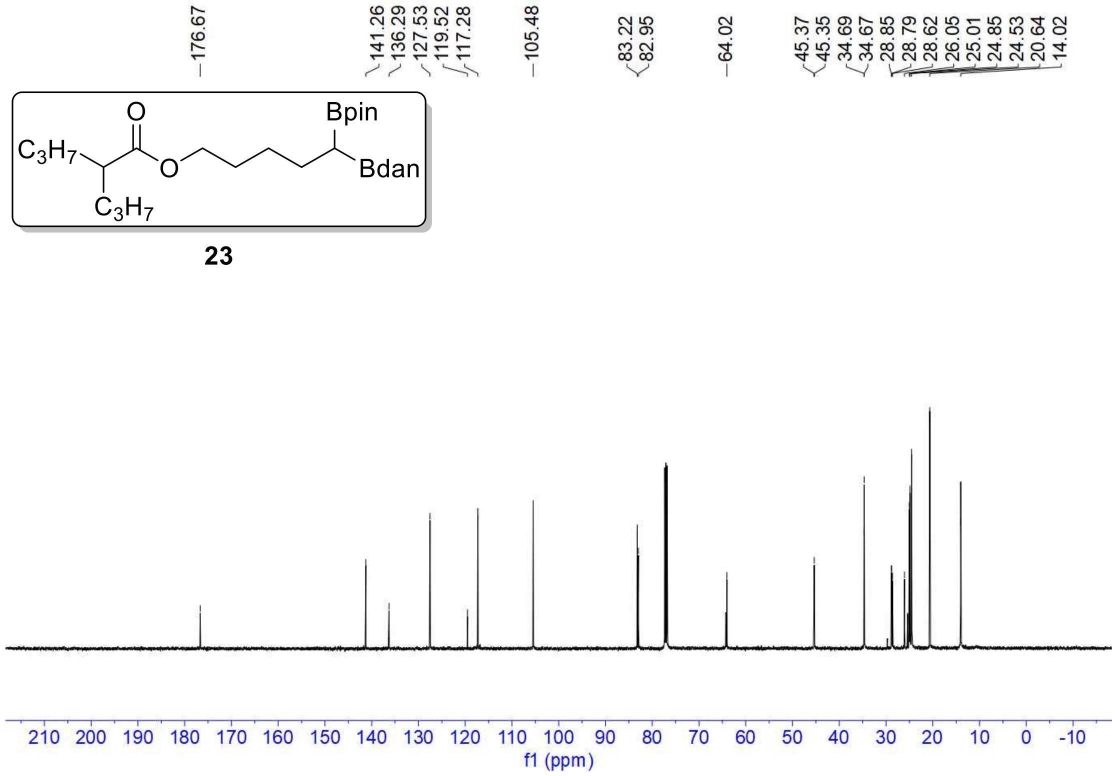

Supplementary Figure 77. ^{13}C NMR spectrum of compound 23

^{11}B NMR (160 MHz, room temperature, $CDCl_3$)

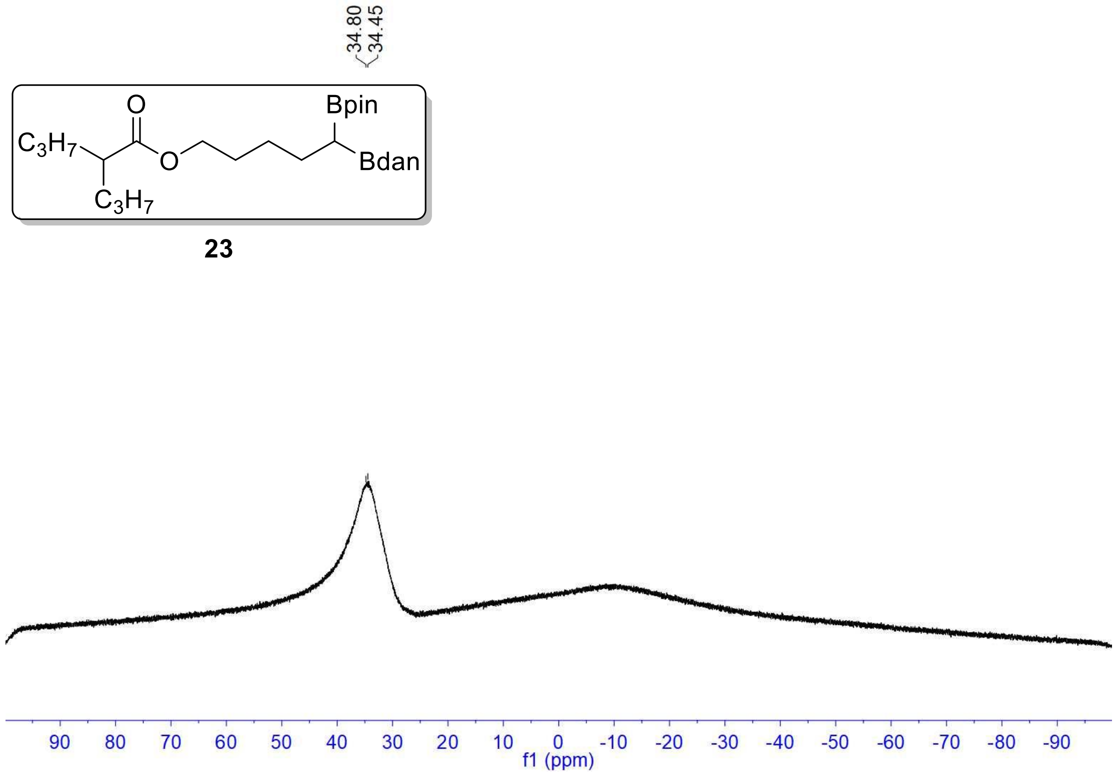

Supplementary Figure 78. ^{11}B NMR spectrum of compound 23

5-(1H-naphtho[1,8-de][1,3,2]diazaborinin-2(3H)-yl)-5-(4,4,5,5-tetramethyl-1,3,2-dioxaborolan-2-yl)pentyl 1-phenylcyclopentane-1-carboxylate (24)

^{1}H NMR (500 MHz, room temperature, CDCl$_3$)

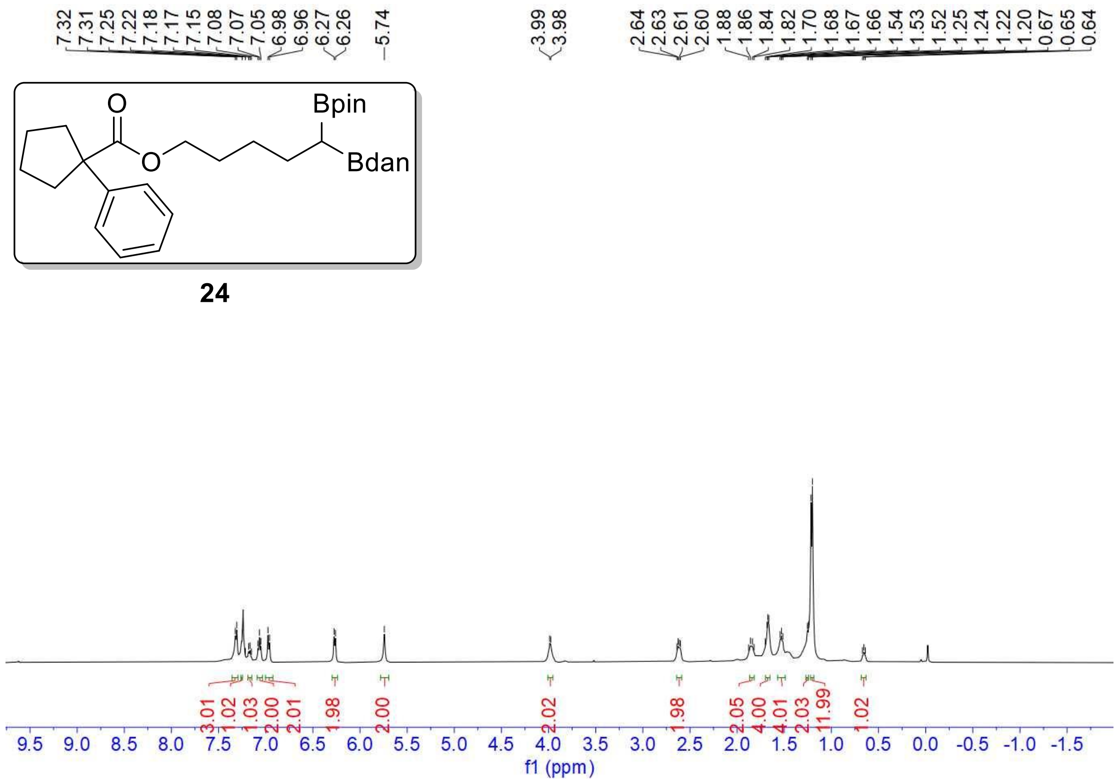

Supplementary Figure 79. ^{1}H NMR spectrum of compound 24

^{13}C NMR (126 MHz, room temperature, CDCl$_3$)

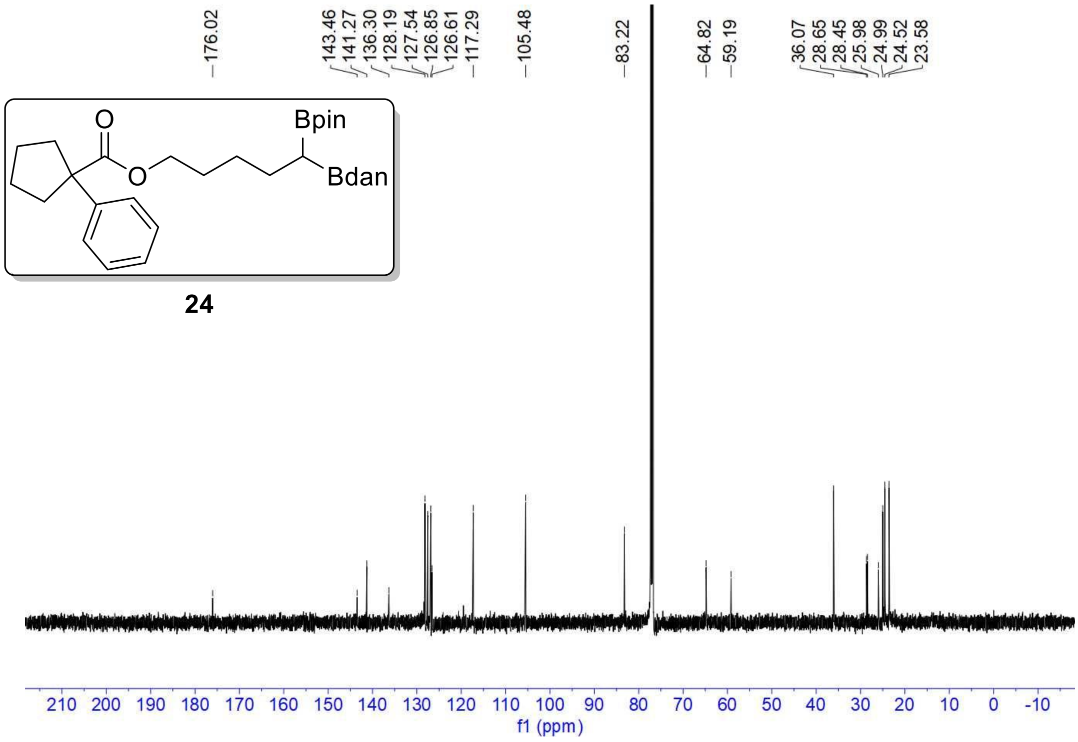

Supplementary Figure 80. ^{13}C NMR spectrum of compound 24

^{11}B NMR (160 MHz, room temperature, CDCl$_3$)

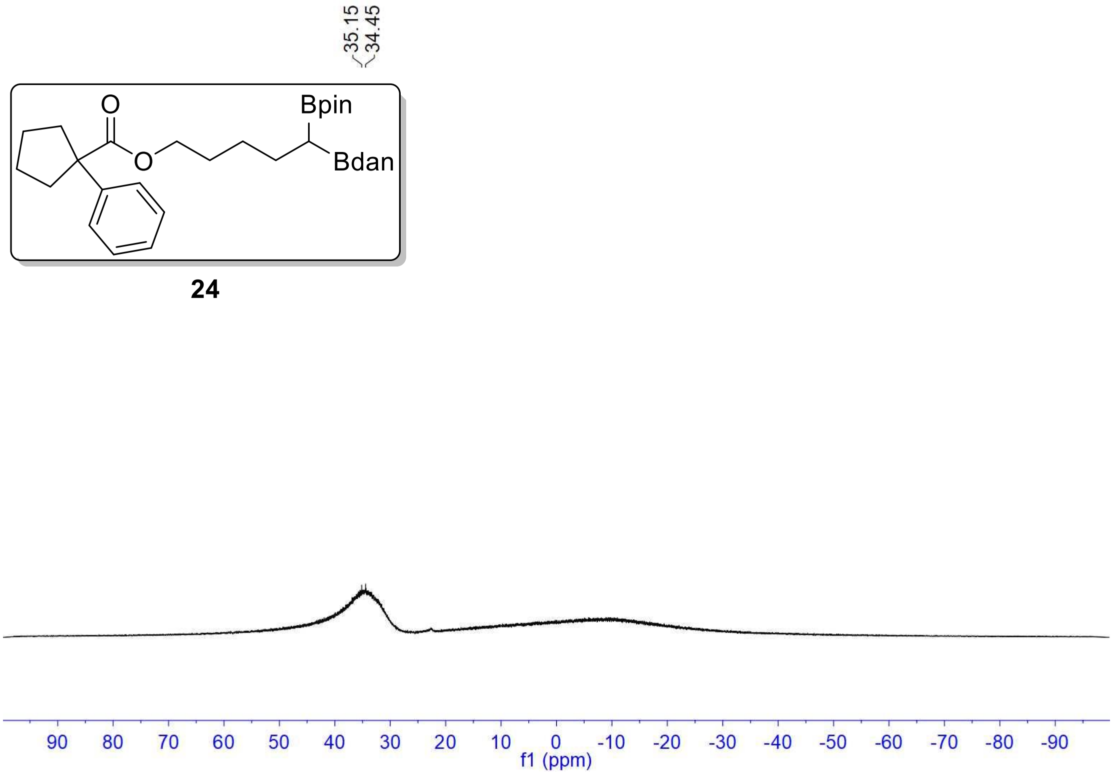

Supplementary Figure 81. ^{11}B NMR spectrum of compound 24

5-(1H-naphtho[1,8-de][1,3,2]diazaborinin-2(3H)-yl)-5-(4,4,5,5-tetramethyl-1,3,2-dioxaborolan-2-yl)pentyl (2R)-2-phenylpropanoate (25)

^{1}H NMR (500 MHz, room temperature, CDCl$_3$)

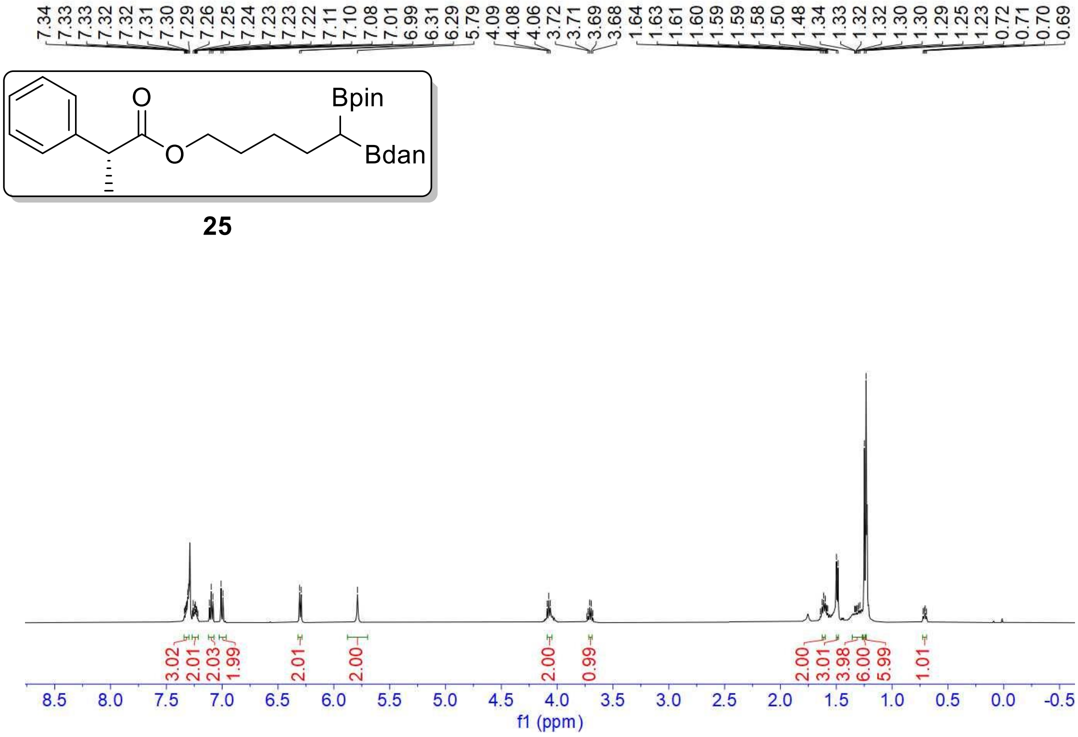

Supplementary Figure 82. ^{1}H NMR spectrum of compound 25

^{13}C NMR (126 MHz, room temperature, $CDCl_3$)

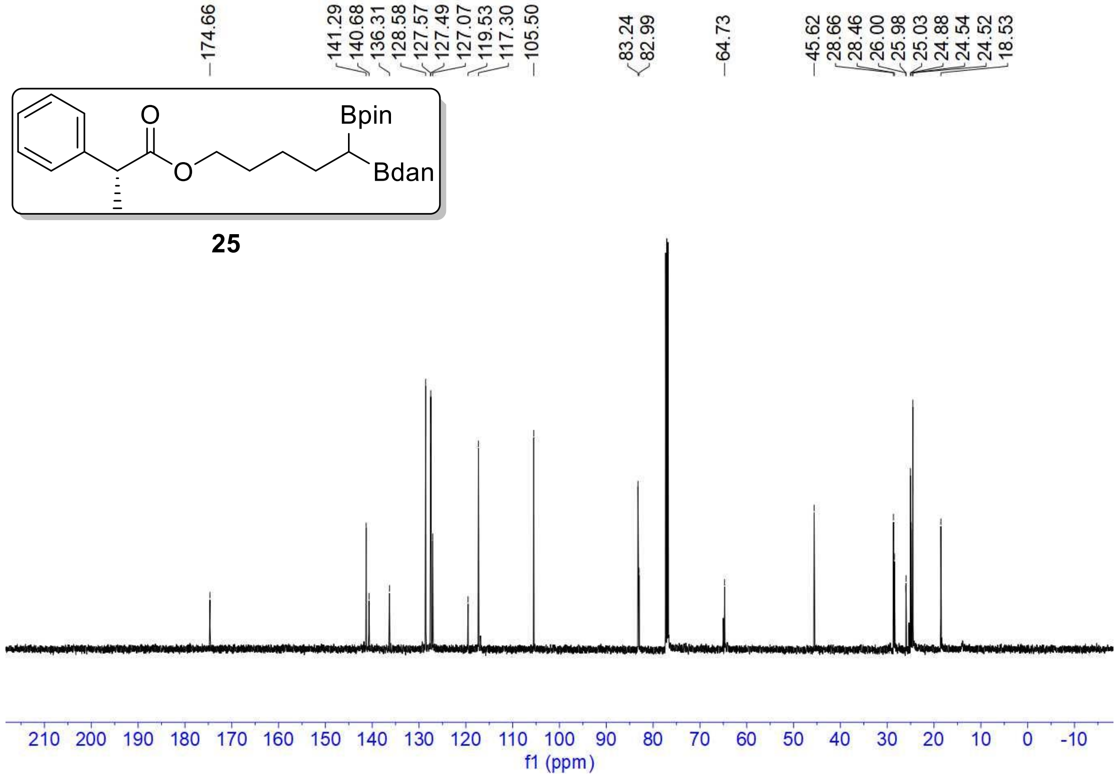

Supplementary Figure 83. ^{13}C NMR spectrum of compound 25

^{11}B NMR (160 MHz, room temperature, $CDCl_3$)

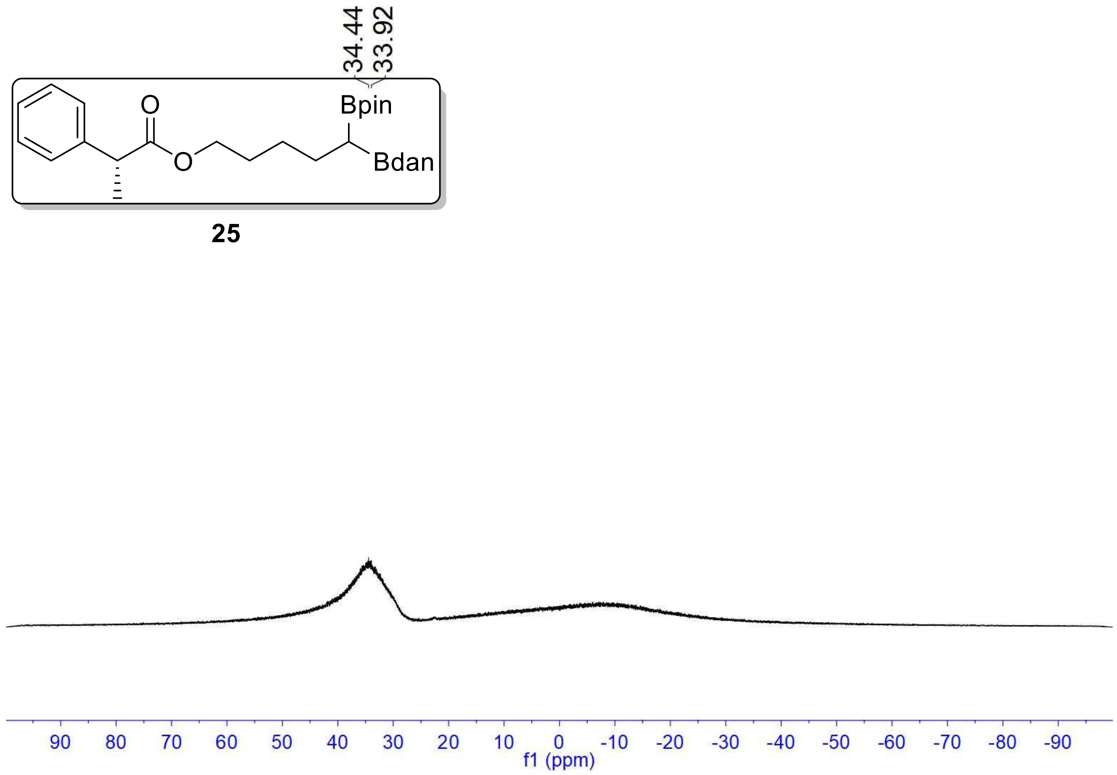

Supplementary Figure 84. ^{11}B NMR spectrum of compound 25

5-(1H-naphtho[1,8-de][1,3,2]diazaborinin-2(3H)-yl)-5-(4,4,5,5-tetramethyl-1,3,2-dioxaborolan-2-yl)pentyl benzoate (26)

^{1}H NMR (500 MHz, room temperature, $CDCl_3$)

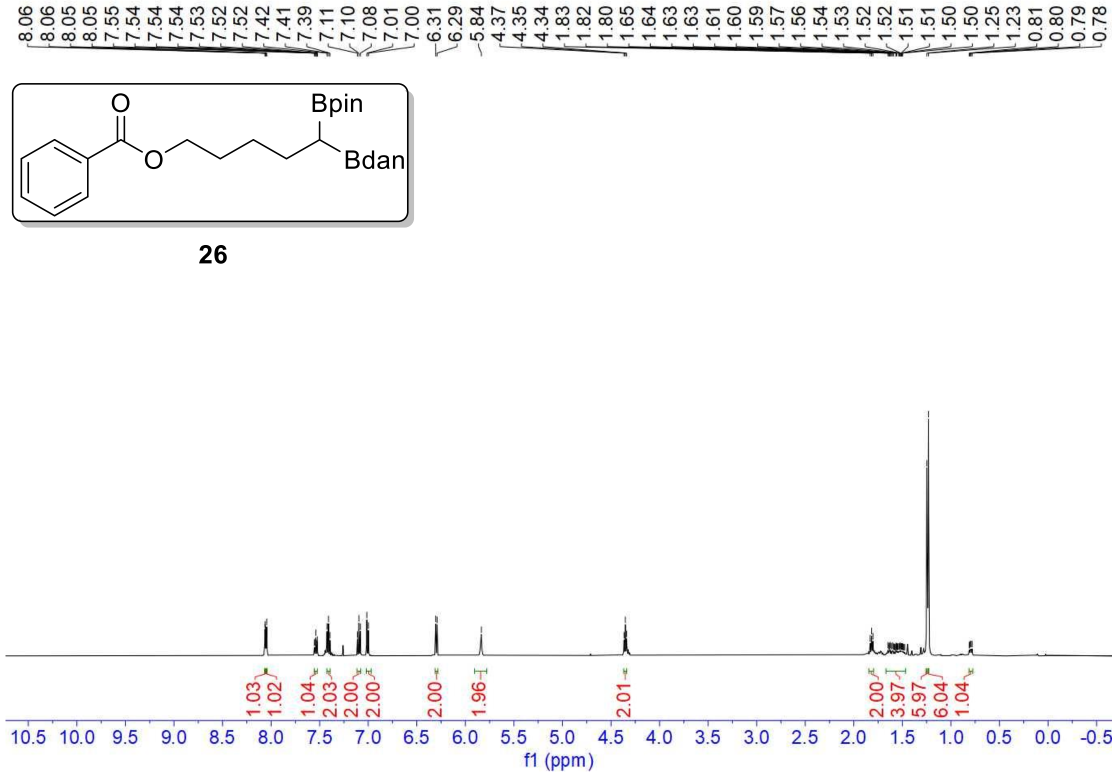

Supplementary Figure 85. ^{1}H NMR spectrum of compound 26

^{13}C NMR (126 MHz, room temperature, $CDCl_3$)

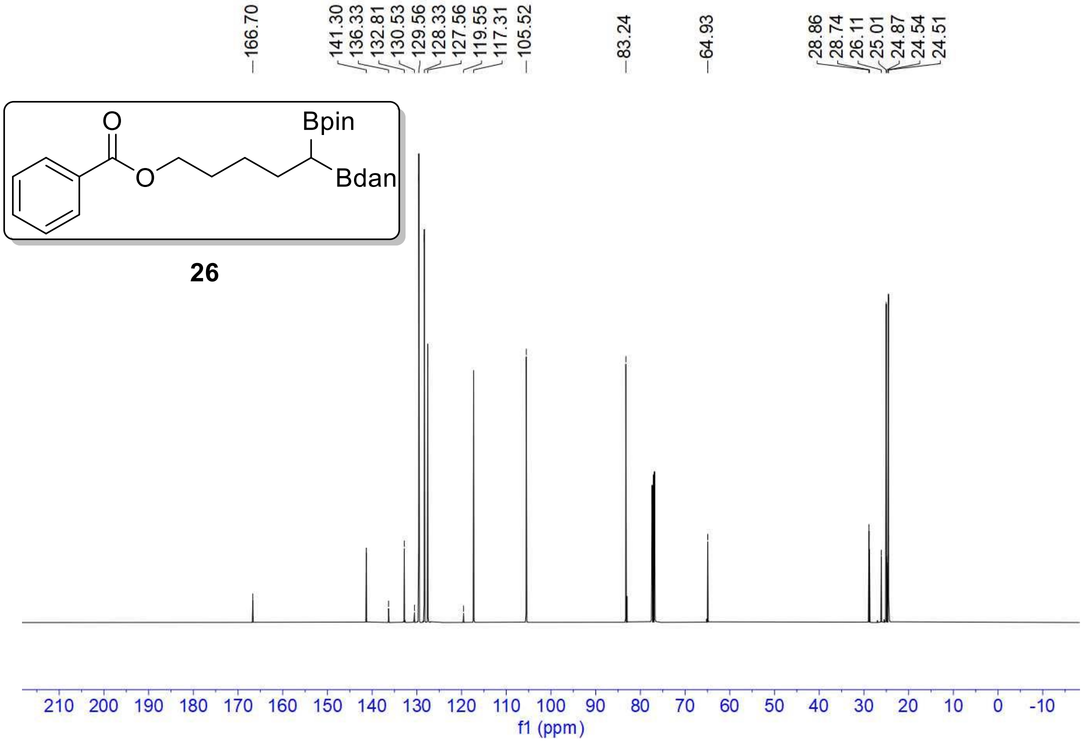

Supplementary Figure 86. ^{13}C NMR spectrum of compound 26

^{11}B NMR (160 MHz, room temperature, $CDCl_3$)

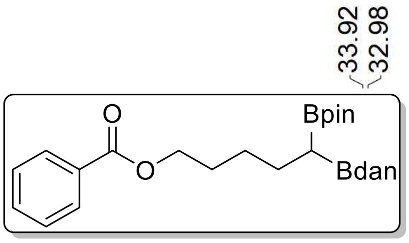

26

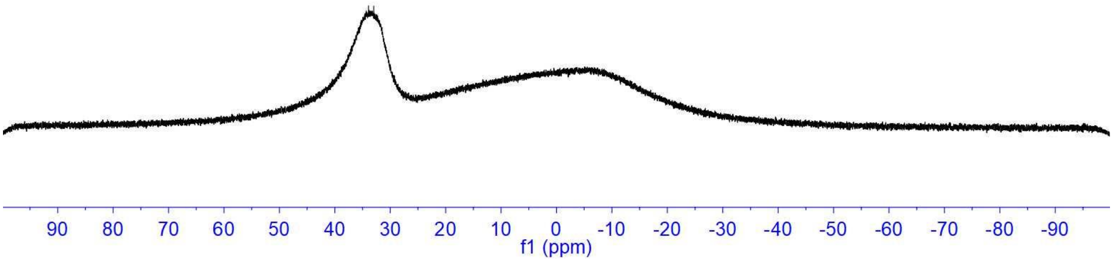

Supplementary Figure 87. [11]**B NMR spectrum of compound 26**

5-(1H-naphtho[1,8-de][1,3,2]diazaborinin-2(3H)-yl)-5-(4,4,5,5-tetramethyl-1,3,2-dioxaborolan-2-yl)pentyl (2S)-2-(4-isobutylphenyl)propanoate (27)

^{1}H NMR (500 MHz, room temperature, $CDCl_3$)

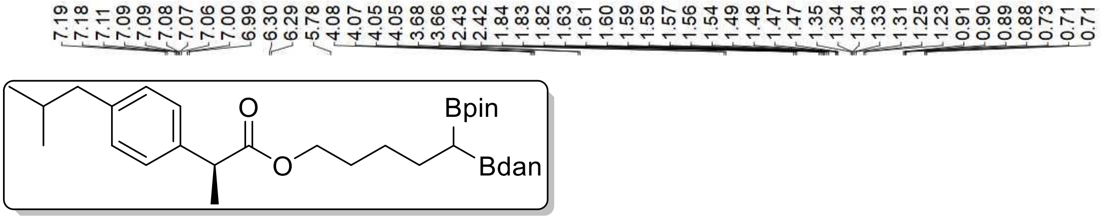

27

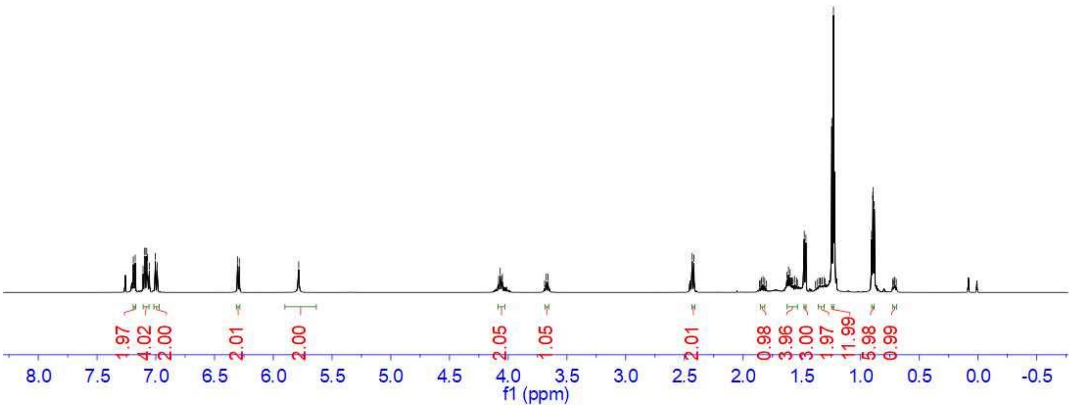

Supplementary Figure 88. [1]**H NMR spectrum of compound 27**

^{13}C NMR (126 MHz, room temperature, $CDCl_3$)

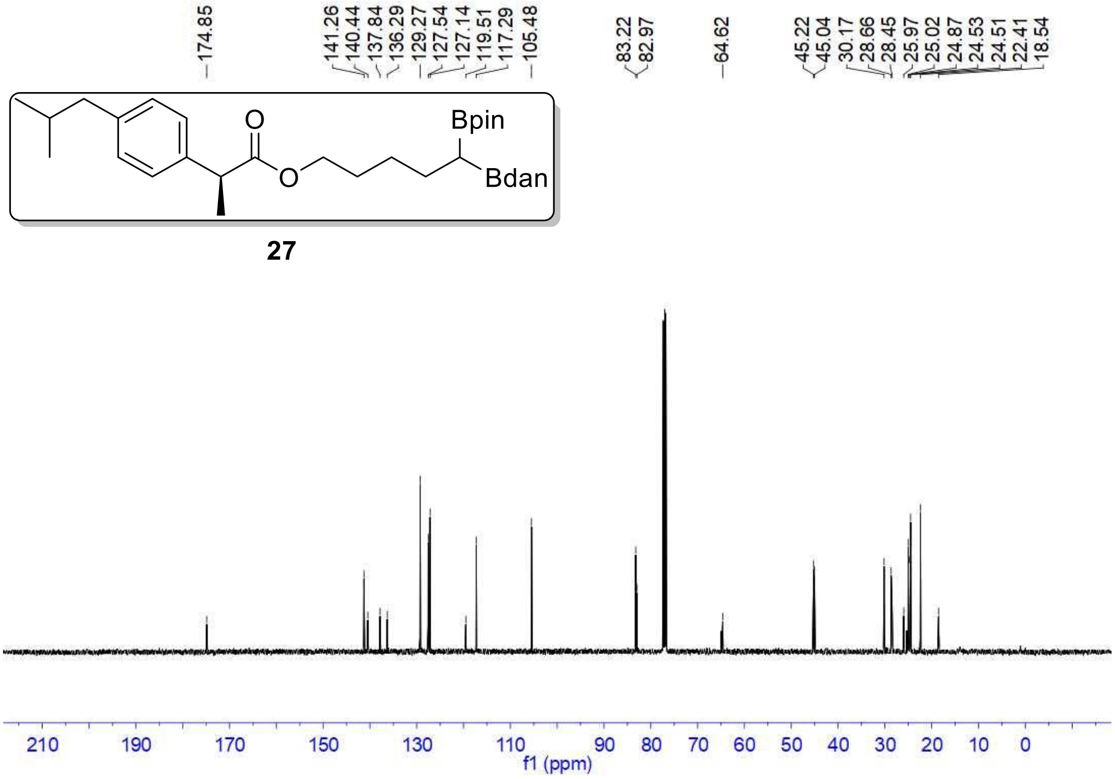

Supplementary Figure 89. ^{13}C NMR spectrum of compound 27

^{11}B NMR (160 MHz, room temperature, $CDCl_3$)

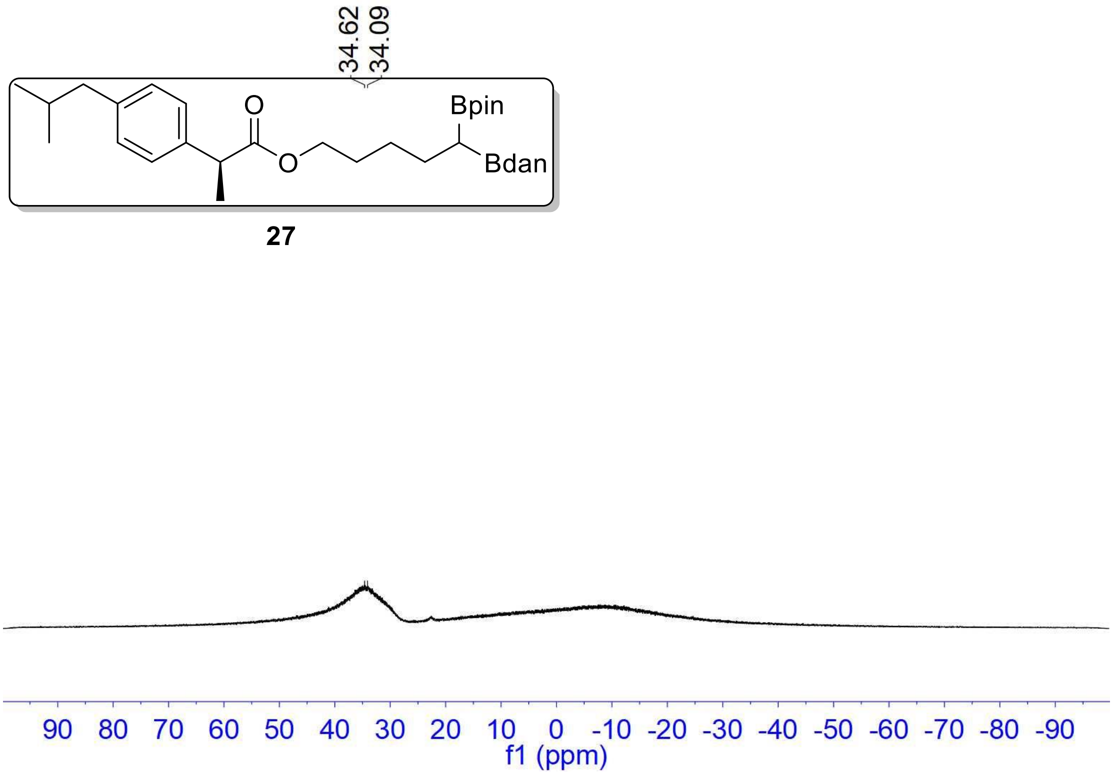

Supplementary Figure 90. ^{11}B NMR spectrum of compound 27

5-(1H-naphtho[1,8-de][1,3,2]diazaborinin-2(3H)-yl)-5-(4,4,5,5-tetramethyl-1,3,2-dioxaborolan-2-yl)pentyl (E)-2-methyl-3-phenylacrylate (28)

^{1}H NMR (500 MHz, room temperature, $CDCl_3$)

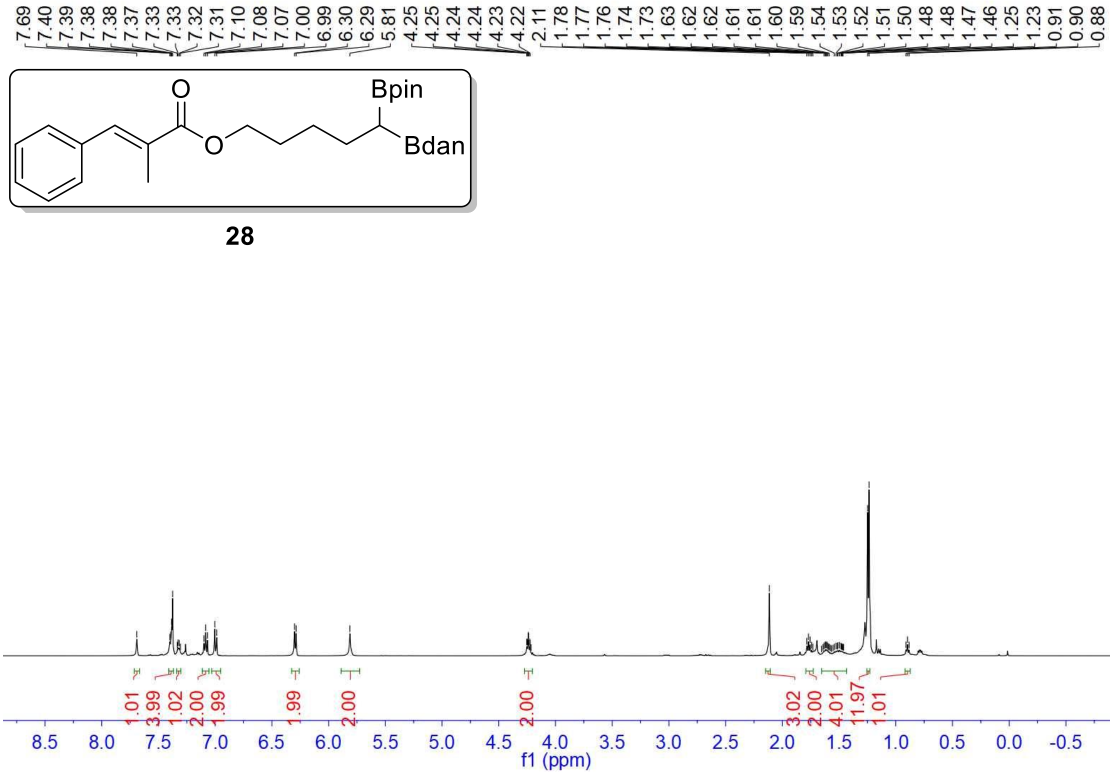

Supplementary Figure 91. ^{1}H NMR spectrum of compound 28

^{13}C NMR (126 MHz, room temperature, $CDCl_3$)

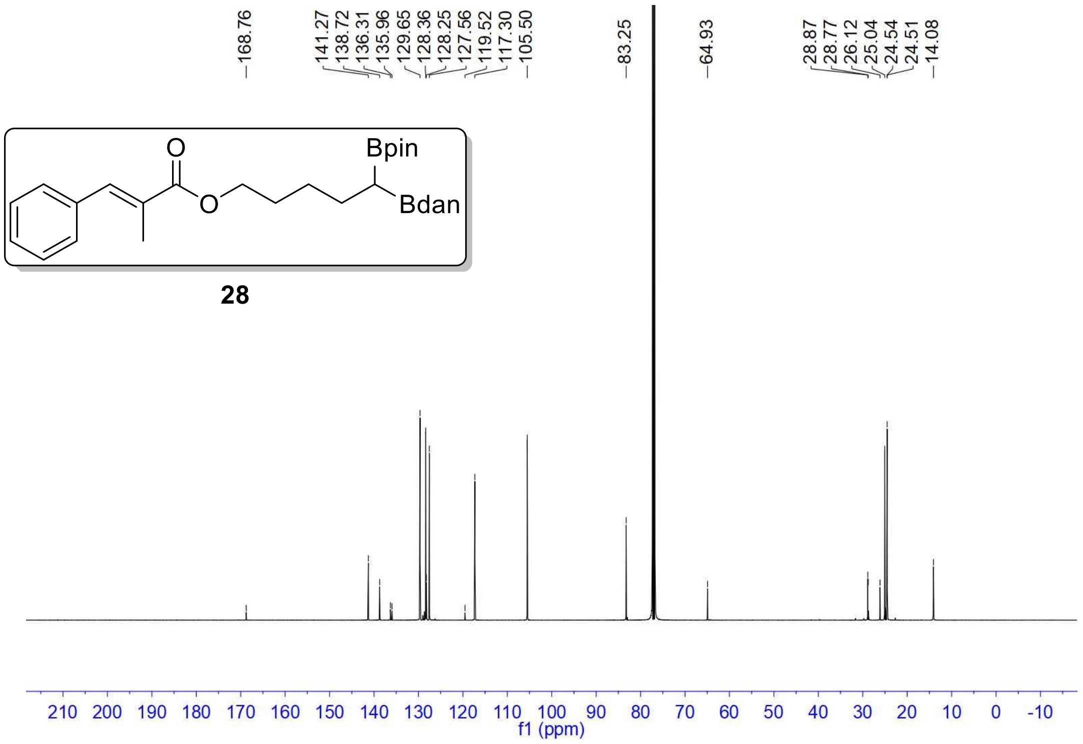

Supplementary Figure 92. ^{13}C NMR spectrum of compound 28

^{11}B NMR (160 MHz, room temperature, $CDCl_3$)

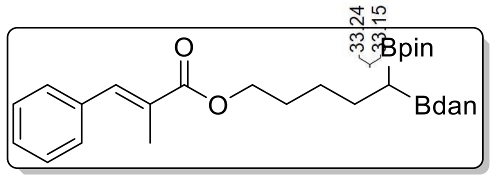

28

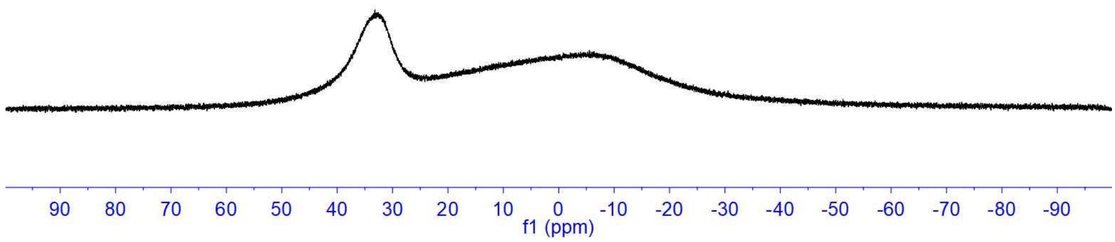

Supplementary Figure 93. ^{11}B NMR spectrum of compound 28

5-(1H-naphtho[1,8-de][1,3,2]diazaborinin-2(3H)-yl)-5-(4,4,5,5-tetramethyl-1,3,2-dioxaborolan-2-yl)pentyl (1R,2S,4R)-bicyclo[2.2.1]hept-5-ene-2-carboxylate (29)

^{1}H NMR (500 MHz, room temperature, $CDCl_3$)

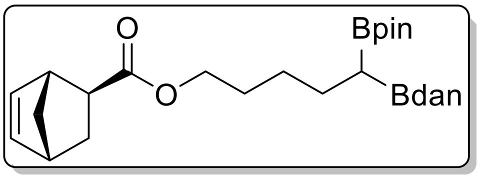

29

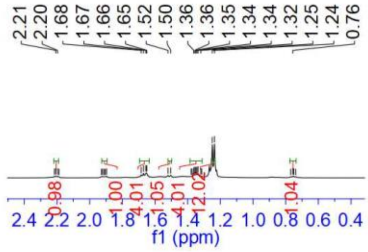

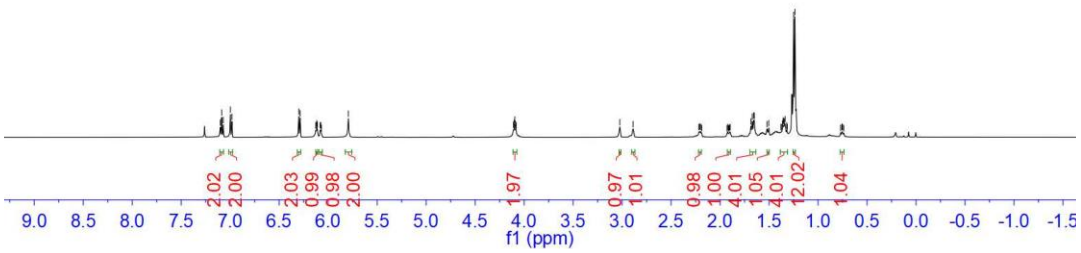

Supplementary Figure 94. ^{1}H NMR spectrum of compound 29

^{13}C NMR (126 MHz, room temperature, $CDCl_3$)

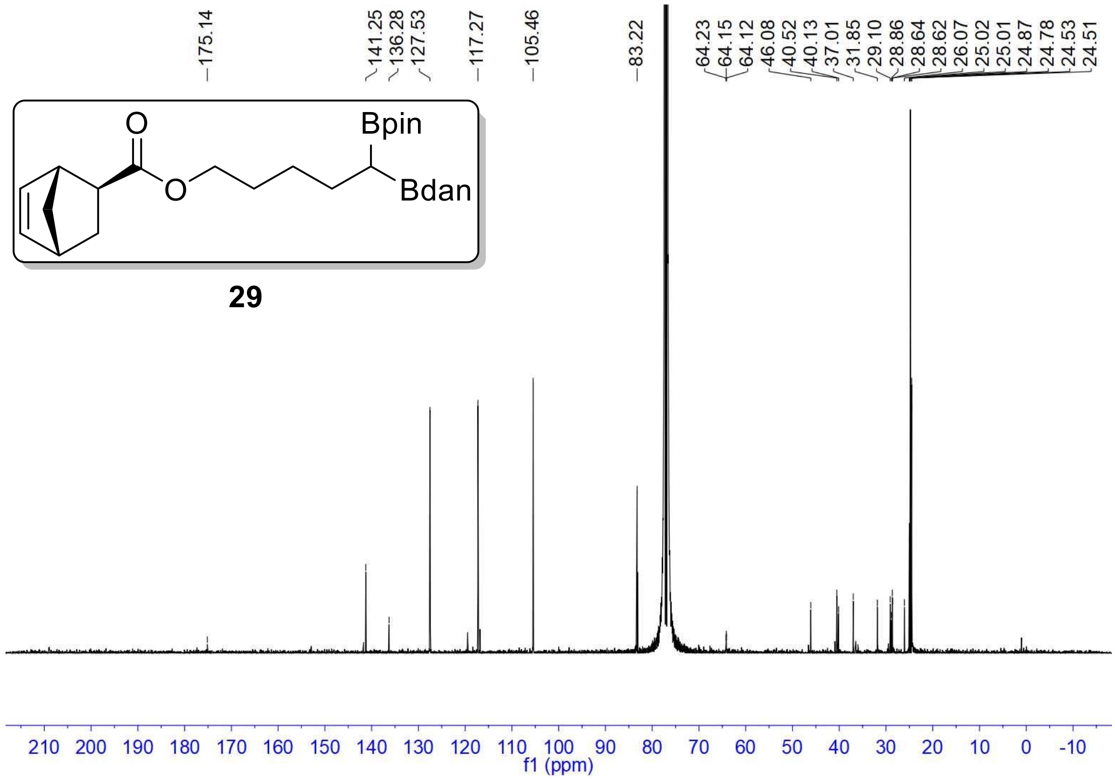

Supplementary Figure 95. ^{13}C NMR spectrum of compound 29

^{11}B NMR (160 MHz, room temperature, $CDCl_3$)

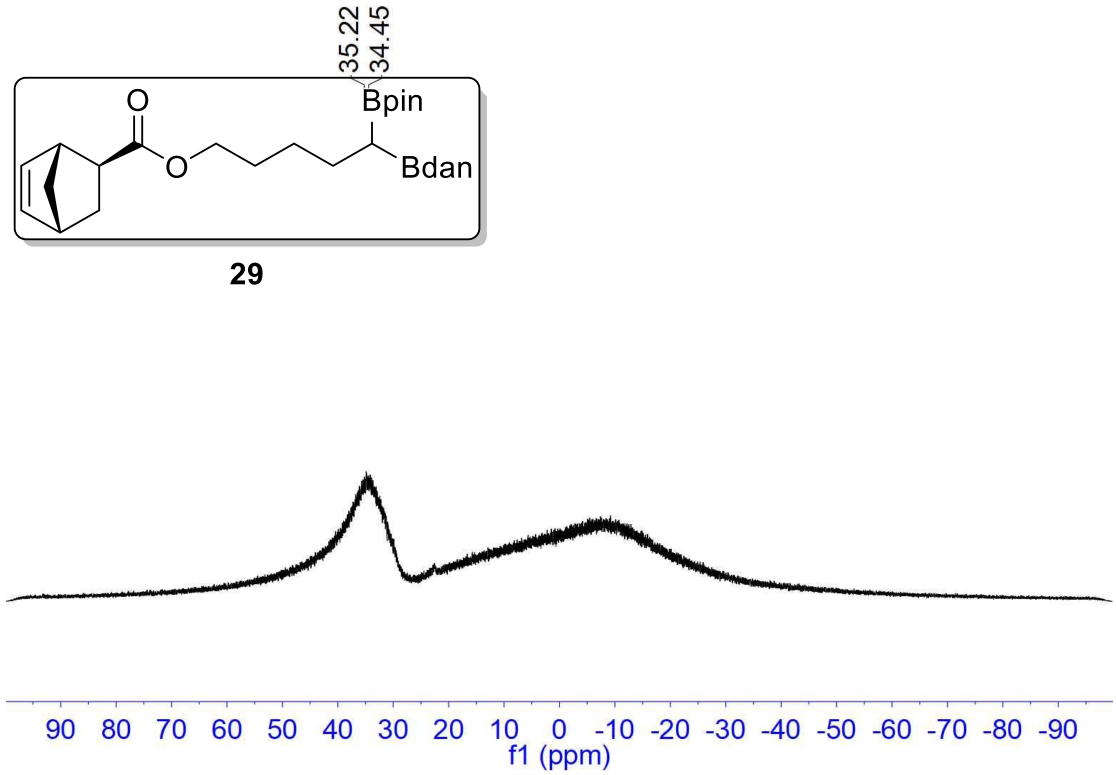

Supplementary Figure 96. ^{11}B NMR spectrum of compound 29

5-(1H-naphtho[1,8-de][1,3,2]diazaborinin-2(3H)-yl)-5-(4,4,5,5-tetramethyl-1,3,2-dioxaborolan-2-yl)pentyl 2,3-dihydrobenzo[b][1,4]dioxine-2-carboxylate (30)

^{1}H NMR (500 MHz, room temperature, CDCl$_3$)

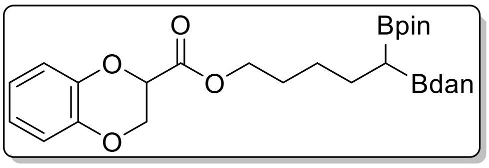

30

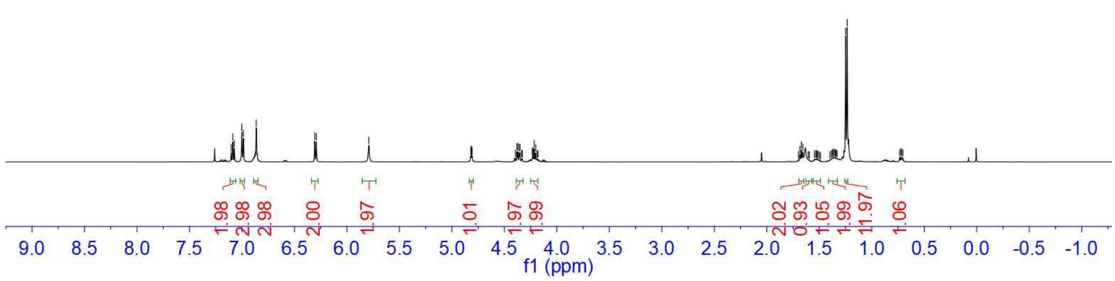

Supplementary Figure 97. ^{1}H NMR spectrum of compound 30

^{13}C NMR (126 MHz, room temperature, CDCl$_3$)

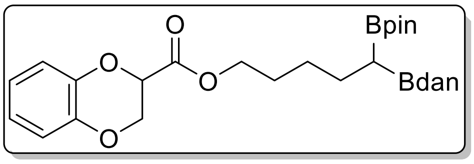

30

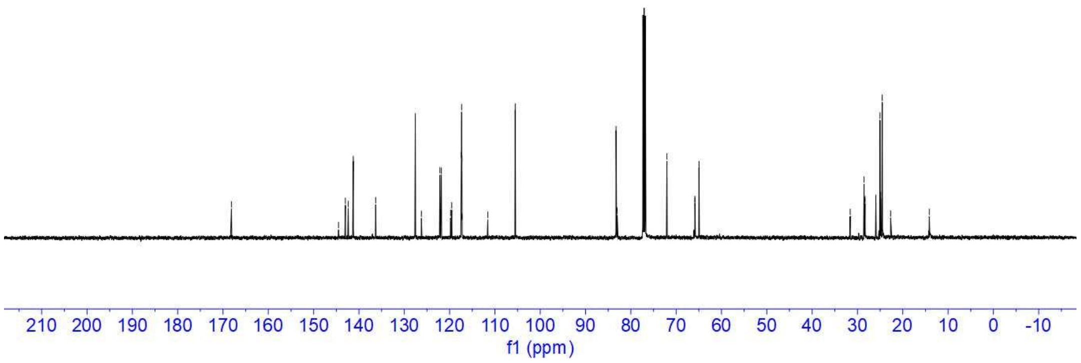

Supplementary Figure 98. ^{13}C NMR spectrum of compound 30

^{11}B NMR (160 MHz, room temperature, $CDCl_3$)

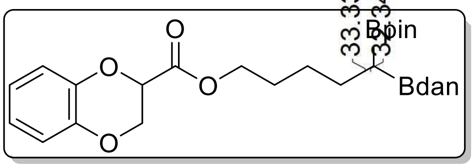

30

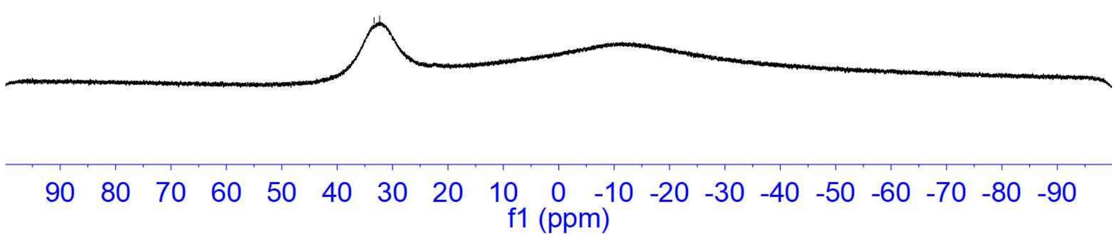

Supplementary Figure 99. ^{11}B NMR spectrum of compound 30

5-(1H-naphtho[1,8-de][1,3,2]diazaborinin-2(3H)-yl)-5-(4,4,5,5-tetramethyl-1,3,2-dioxaborolan-2-yl)pentyl 6-(3-(bicyclo[3.3.1]nonan-1-yl)-4-methoxyphenyl)-2-naphthoate (31)

^{1}H NMR (500 MHz, room temperature, $CDCl_3$)

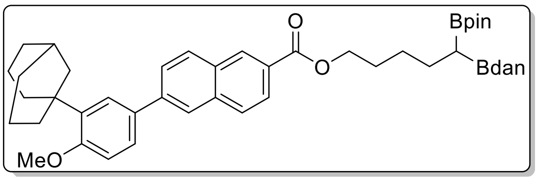

31

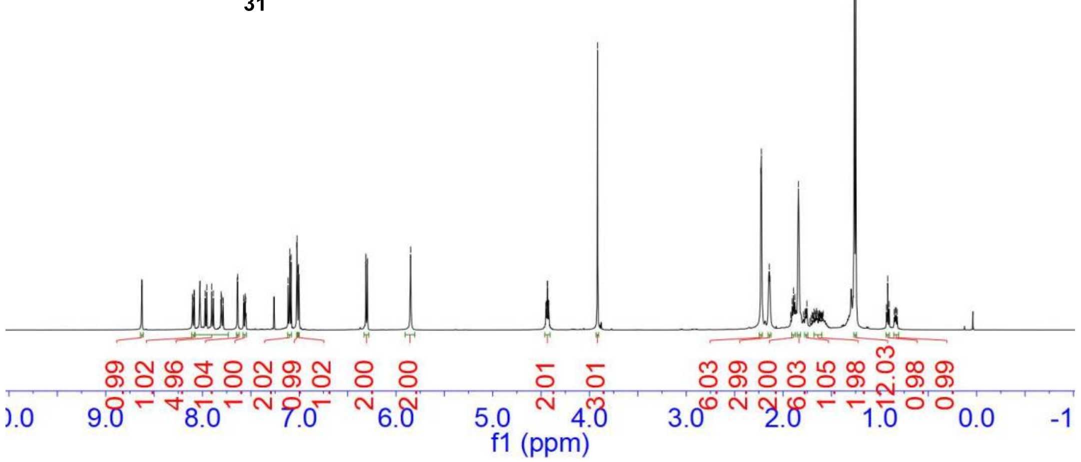

Supplementary Figure 100. ^{1}H NMR spectrum of compound 31

^{13}C NMR (126 MHz, room temperature, $CDCl_3$)

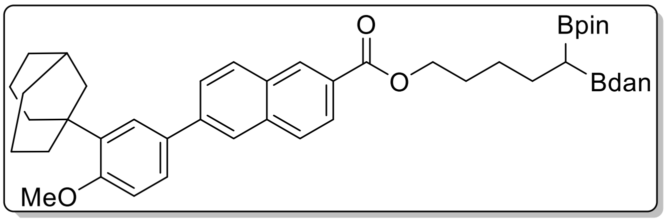

31

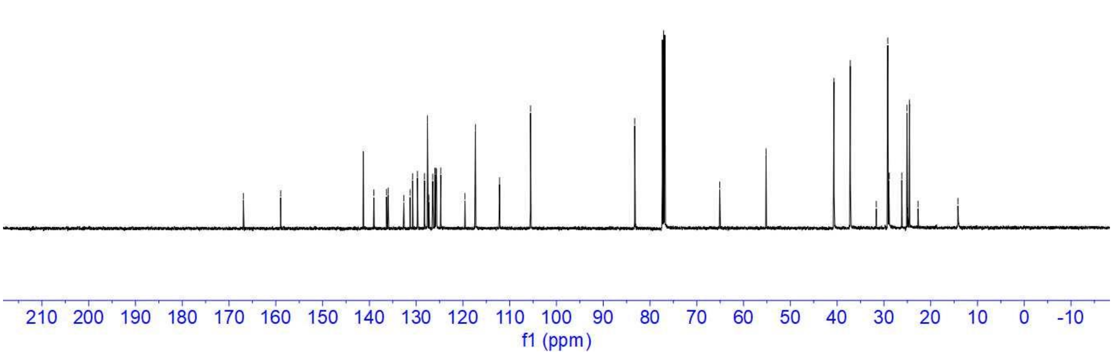

Supplementary Figure 101. [13]**C NMR spectrum of compound 31**

^{11}B NMR (160 MHz, room temperature, $CDCl_3$)

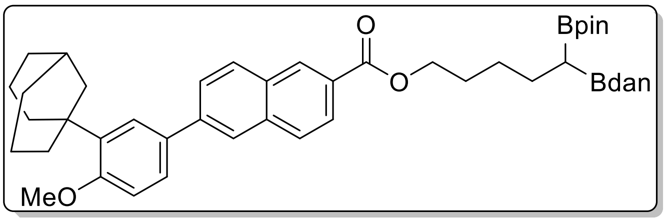

31

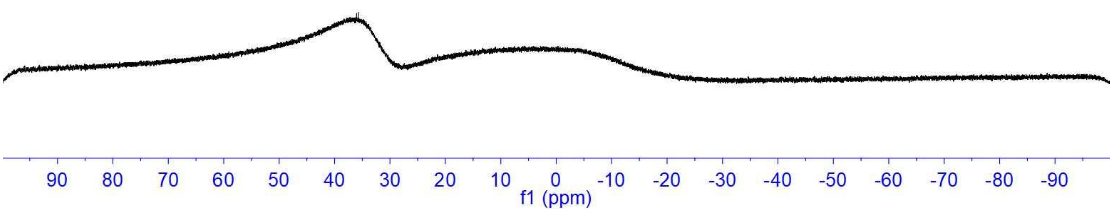

Supplementary Figure 102. [11]**B NMR spectrum of compound 31**

5-(1H-naphtho[1,8-de][1,3,2]diazaborinin-2(3H)-yl)-5-(4,4,5,5-tetramethyl-1,3,2-dioxaborolan-2-yl)pentyl (2R)-2-(6-methoxynaphthalen-2-yl)propanoate (32)

^{1}H NMR (500 MHz, room temperature, CDCl$_3$)

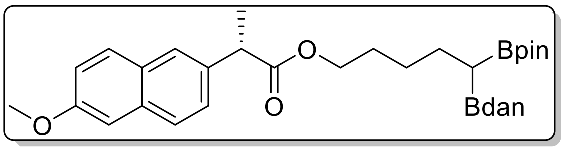

32

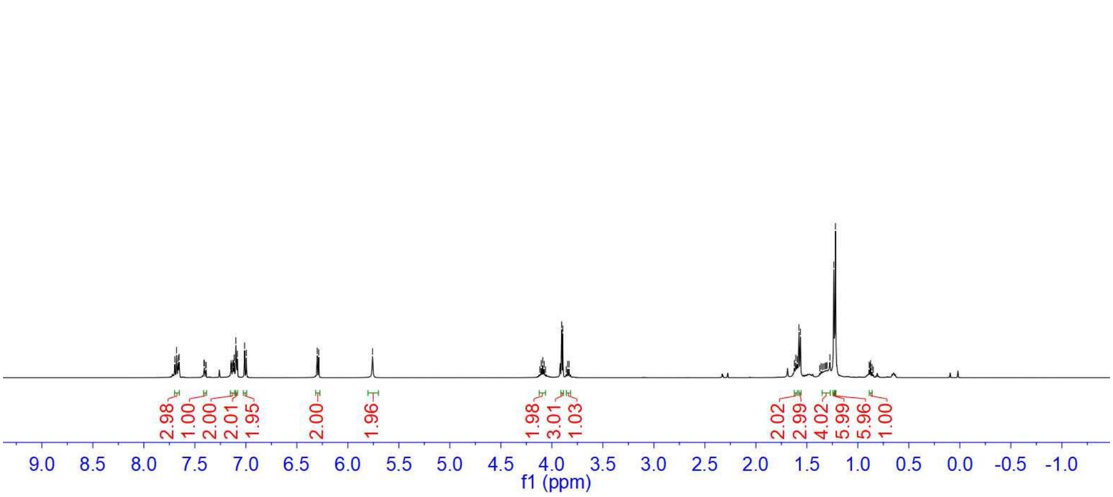

Supplementary Figure 103. ^{1}H NMR spectrum of compound 32

^{13}C NMR (126 MHz, room temperature, CDCl$_3$)

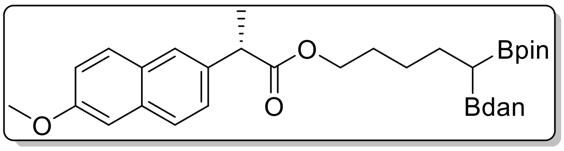

32

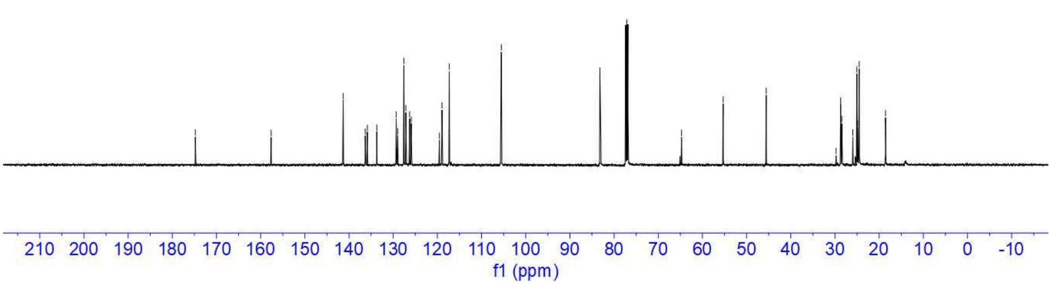

Supplementary Figure 104. ^{13}C NMR spectrum of compound 32

^{11}B NMR (160 MHz, room temperature, $CDCl_3$)

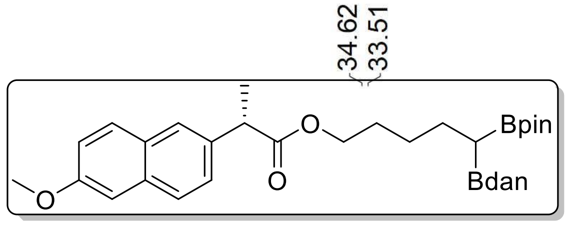

32

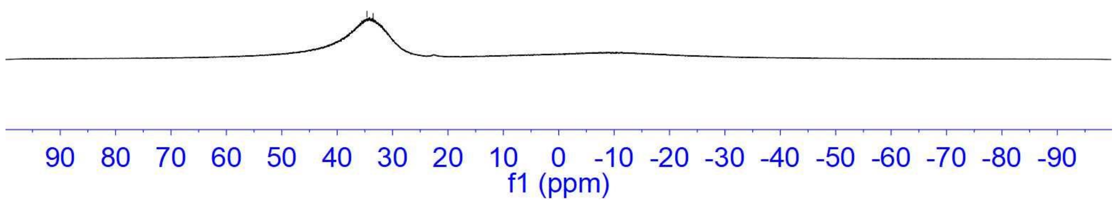

Supplementary Figure 105. ^{11}B NMR spectrum of compound 32

5-(1H-naphtho[1,8-de][1,3,2]diazaborinin-2(3H)-yl)-5-(4,4,5,5-tetramethyl-1,3,2-dioxaborolan-2-yl)pentyl 4-cyclohexylbenzoate (33)

^{1}H NMR (500 MHz, room temperature, $CDCl_3$)

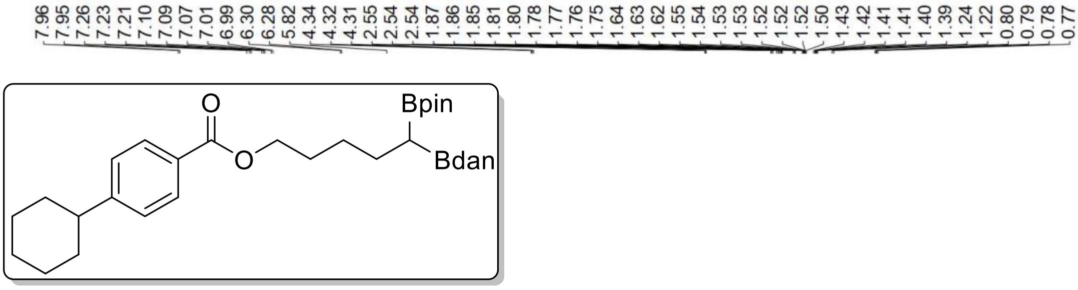

33

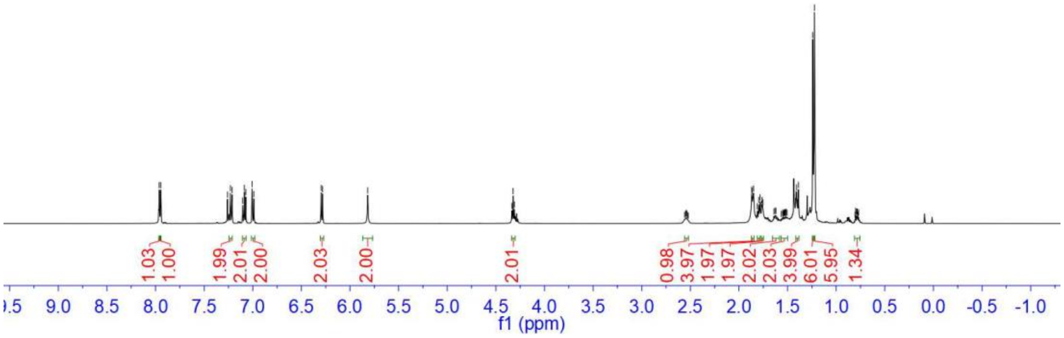

Supplementary Figure 106. ^{1}H NMR spectrum of compound 33

^{13}C NMR (126 MHz, room temperature, $CDCl_3$)

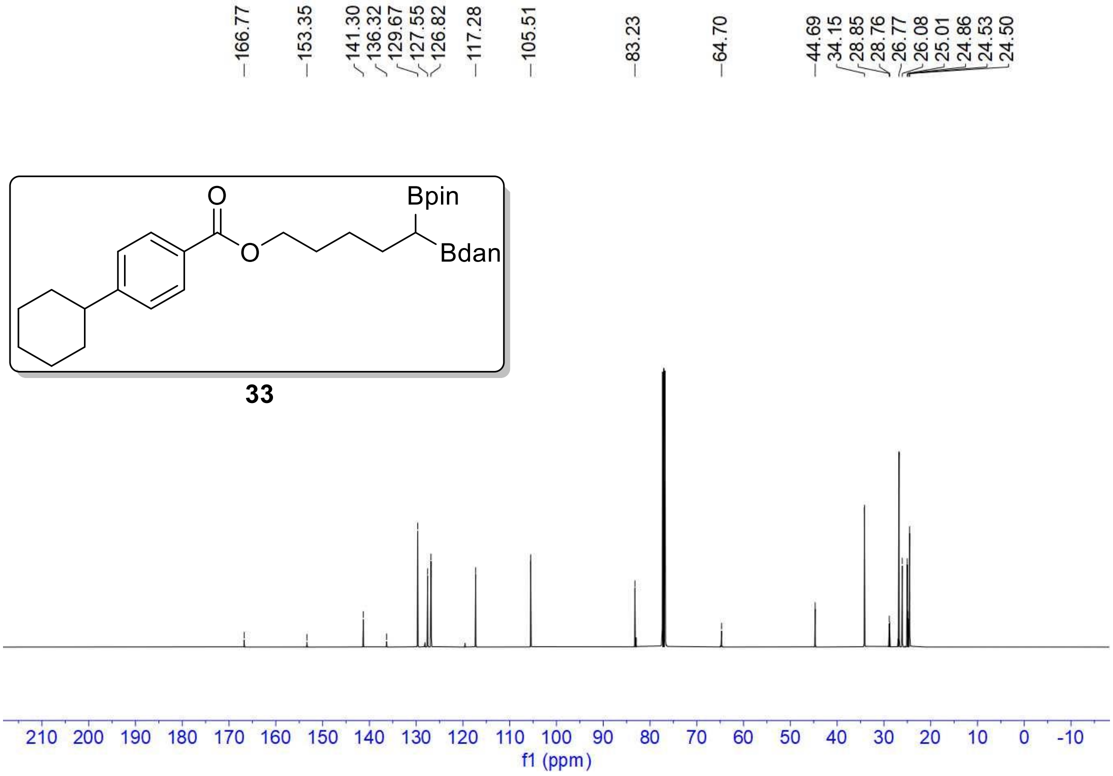

Supplementary Figure 107. ^{13}C NMR spectrum of compound 33

^{11}B NMR (160 MHz, room temperature, $CDCl_3$)

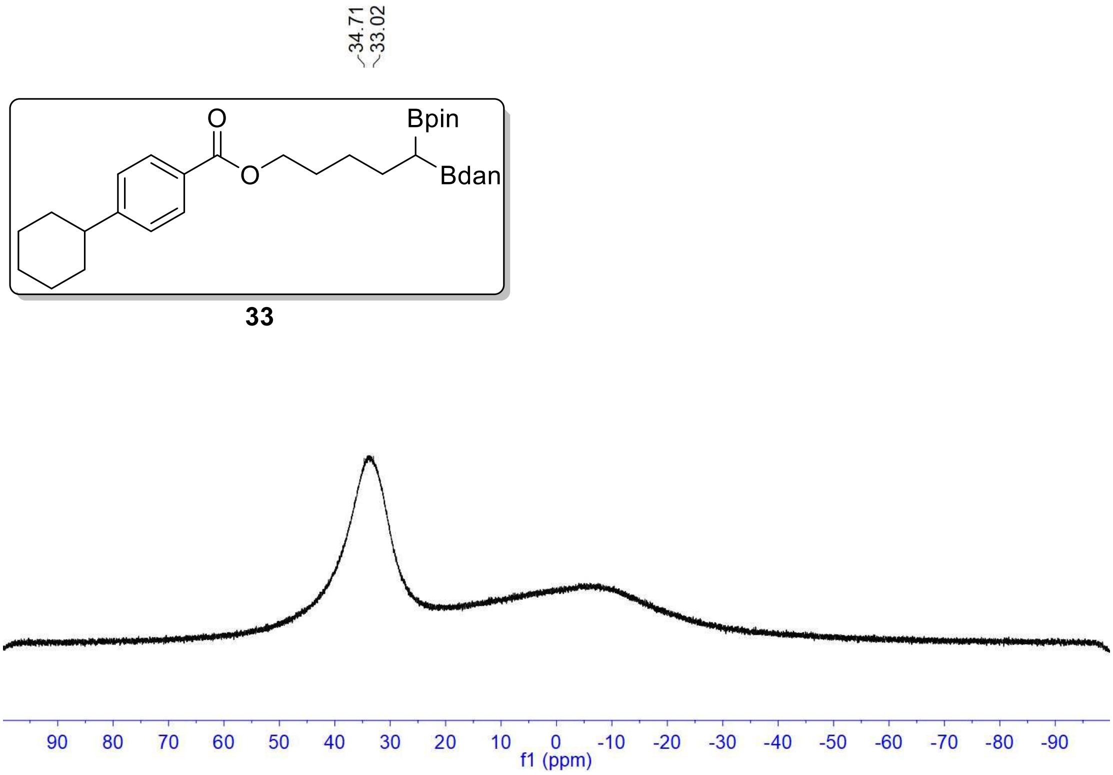

Supplementary Figure 108. ^{11}B NMR spectrum of compound 33

2-(2-phenyl-1-(4,4,5,5-tetramethyl-1,3,2-dioxaborolan-2-yl)ethyl)-2,3-dihydro-1H-naphtho[1,8-de][1,3,2]diazaborinine (34)

^{1}H NMR (500 MHz, room temperature, $CDCl_3$)

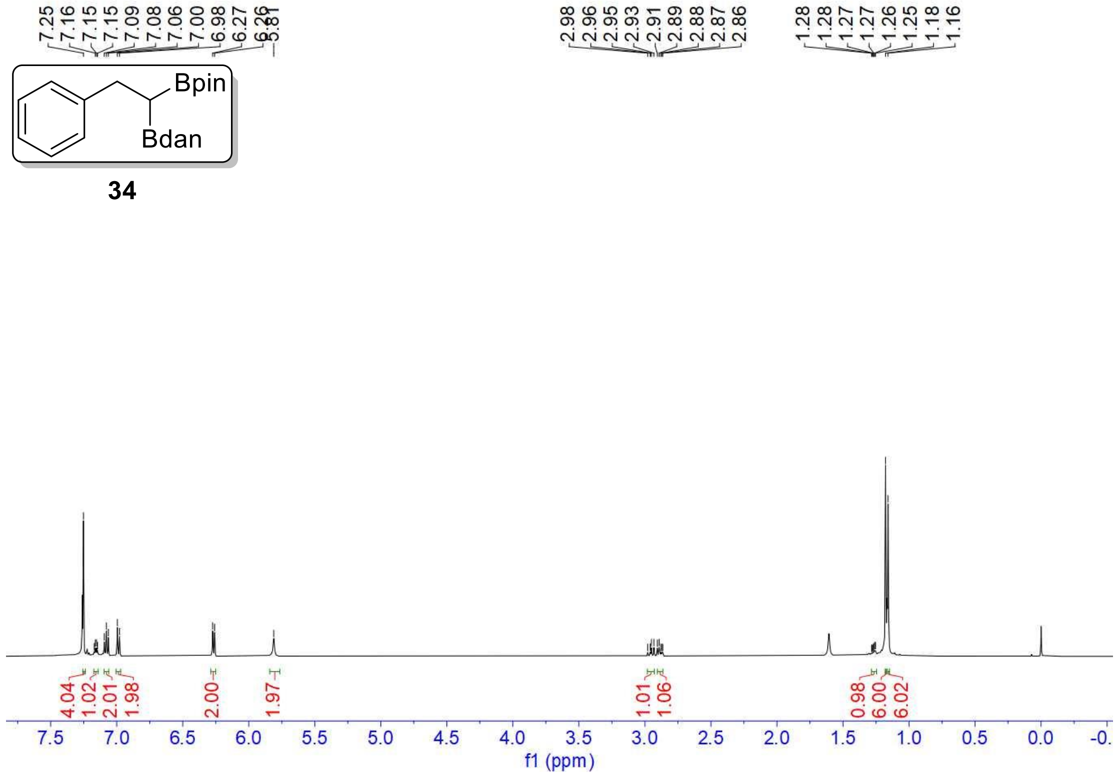

Supplementary Figure 109. ^{1}H NMR spectrum of compound 34

^{13}C NMR (126 MHz, room temperature, $CDCl_3$)

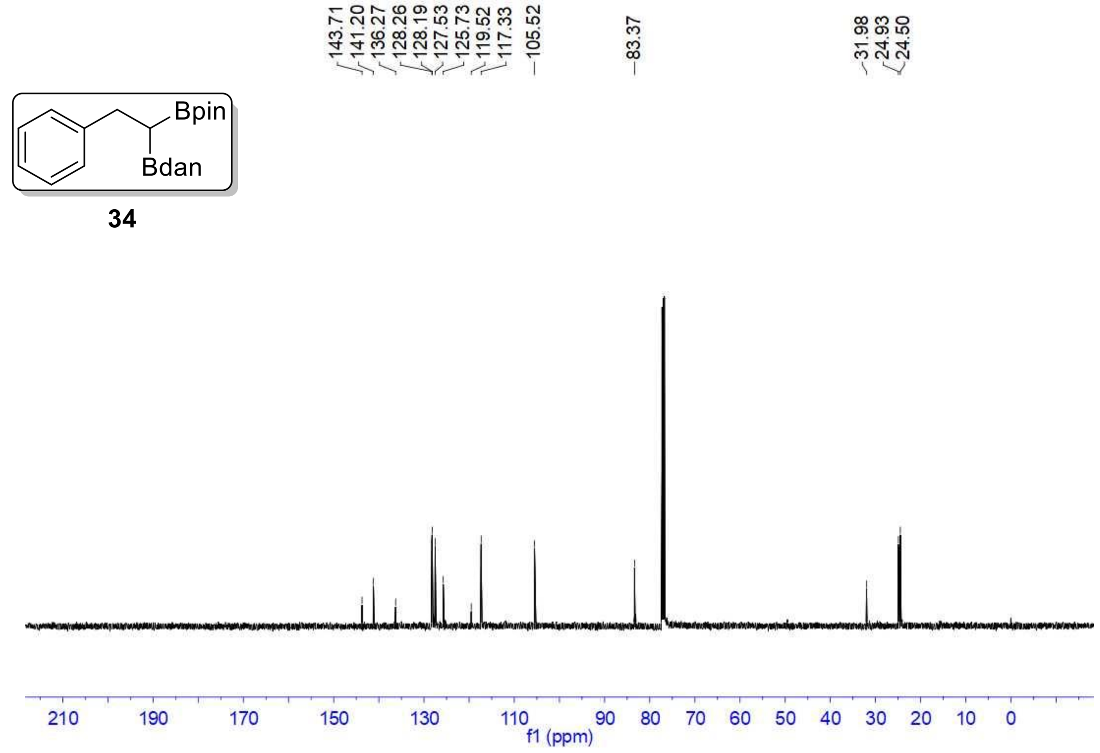

Supplementary Figure 110. ^{13}C NMR spectrum of compound 34

^{11}B NMR (160 MHz, room temperature, CDCl$_3$)

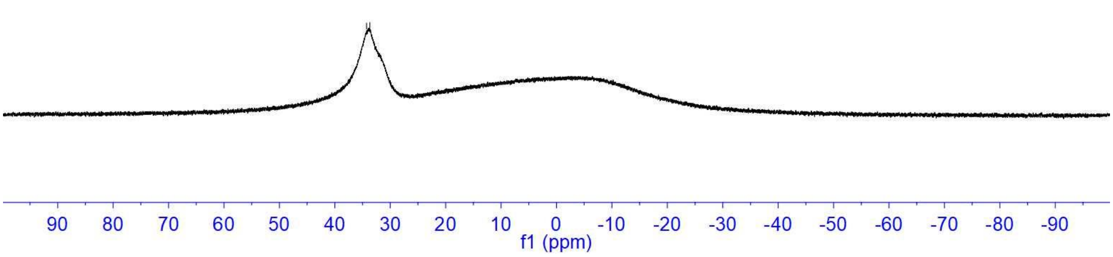

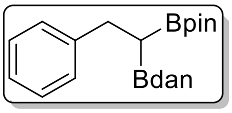

34

Supplementary Figure 111. ^{11}B NMR spectrum of compound 34

2-(1-(4,4,5,5-tetramethyl-1,3,2-dioxaborolan-2-yl)-2-(p-tolyl)ethyl)-2,3-dihydro-1H-naphtho[1,8-de][1,3,2]diazaborinine (35)

^{1}H NMR (500 MHz, room temperature, CDCl$_3$)

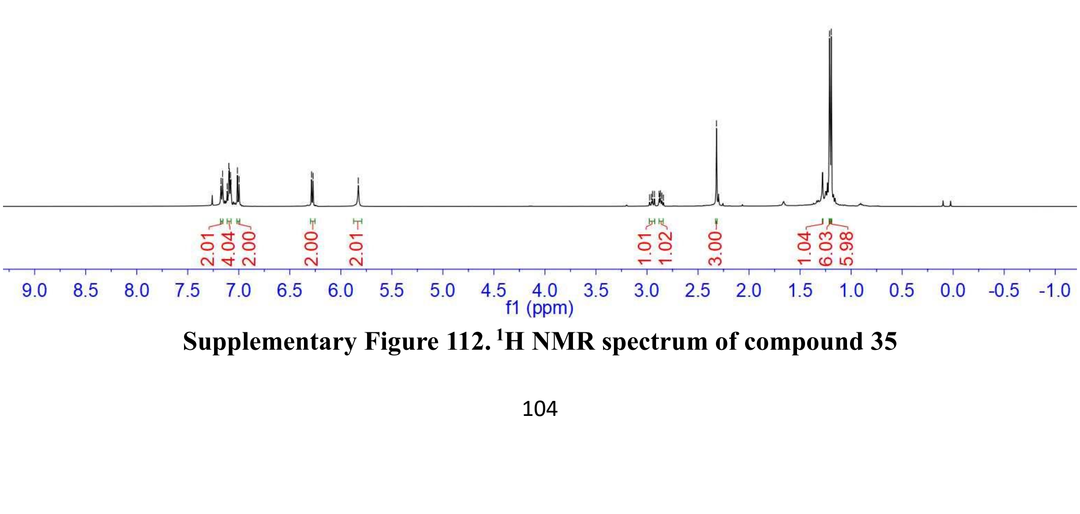

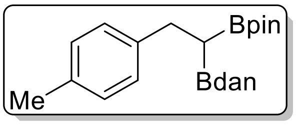

35

Supplementary Figure 112. ^{1}H NMR spectrum of compound 35

^{13}C NMR (126 MHz, room temperature, $CDCl_3$)

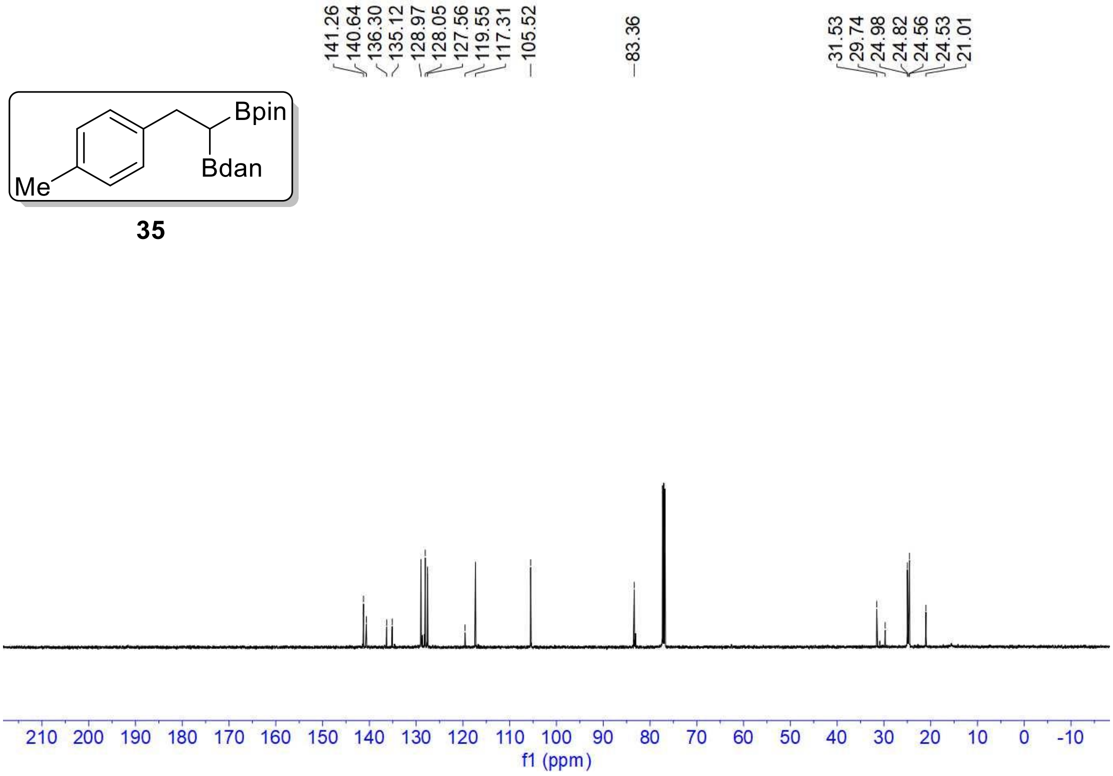

Supplementary Figure 113. ^{13}C NMR spectrum of compound 35

^{11}B NMR (160 MHz, room temperature, $CDCl_3$)

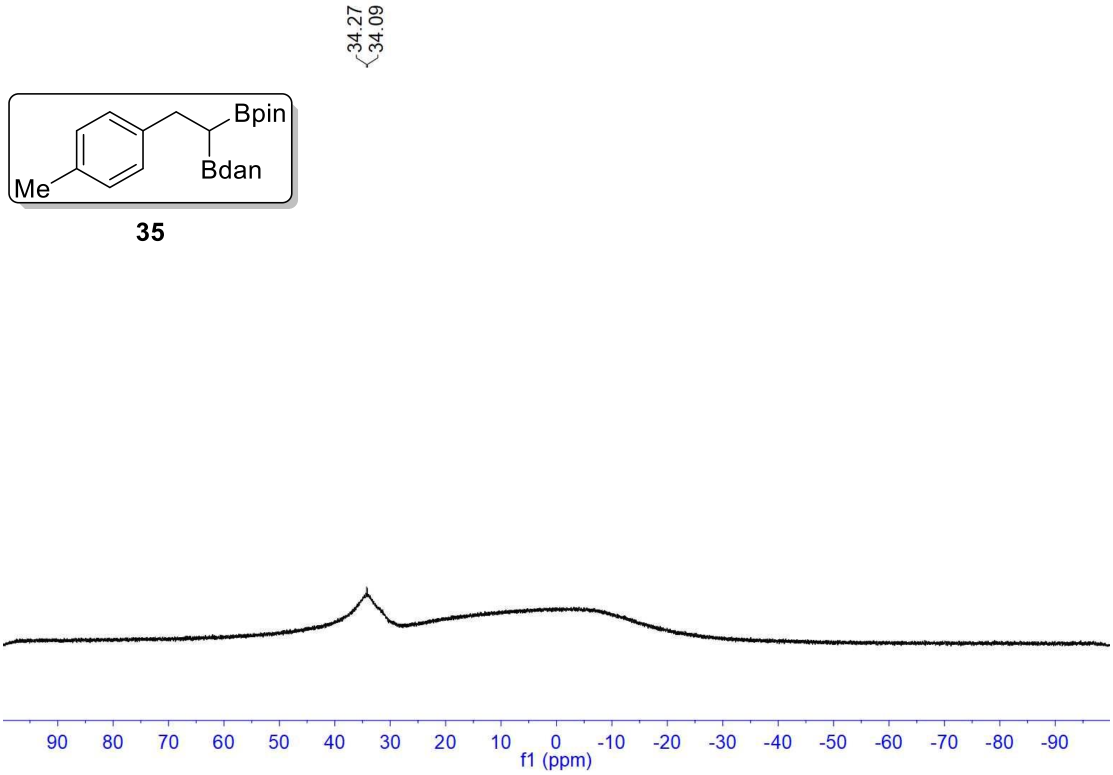

Supplementary Figure 114. ^{11}B NMR spectrum of compound 35

2-(2-(4-ethylphenyl)-1-(4,4,5,5-tetramethyl-1,3,2-dioxaborolan-2-yl)ethyl)-2,3-dihydro-1H-naphtho[1,8-de][1,3,2]diazaborinine (36)

^{1}H NMR (500 MHz, room temperature, CDCl$_3$)

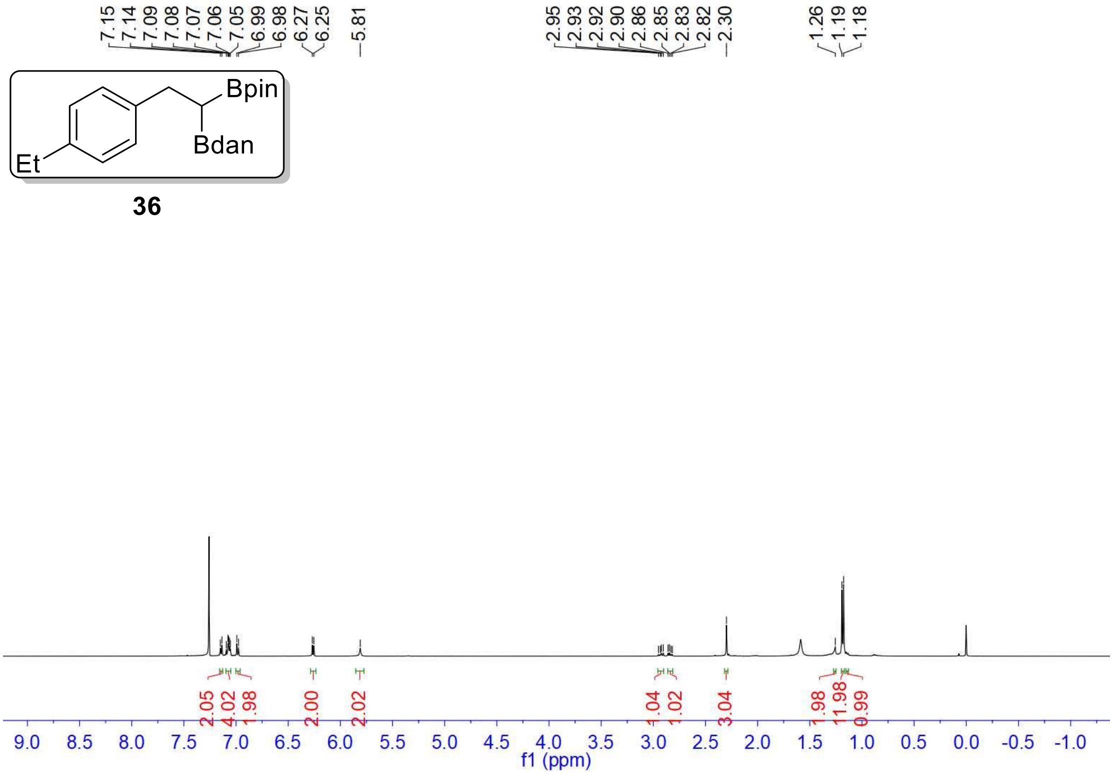

Supplementary Figure 115. ^{1}H NMR spectrum of compound 36

^{13}C NMR (126 MHz, room temperature, CDCl$_3$)

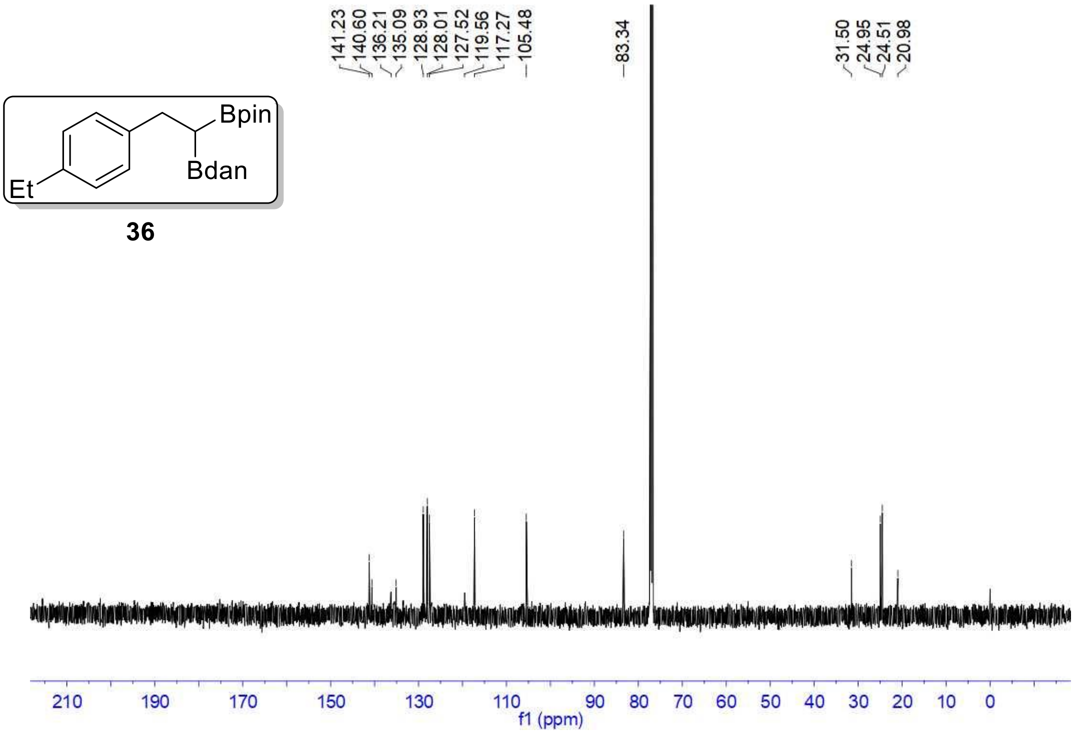

Supplementary Figure 116. ^{13}C NMR spectrum of compound 36

^{11}B NMR (160 MHz, room temperature, $CDCl_3$)

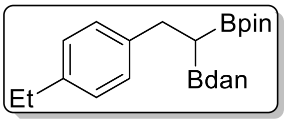

36

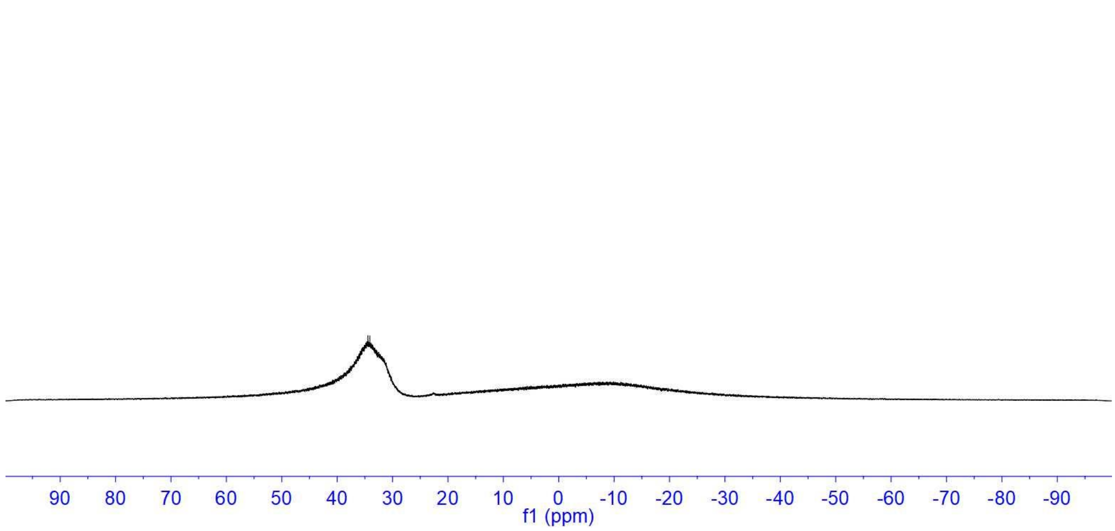

Supplementary Figure 117. ^{11}B NMR spectrum of compound 36

2-(2-(4-(tert-butyl)phenyl)-1-(4,4,5,5-tetramethyl-1,3,2-dioxaborolan-2-yl)ethyl)-2,3-dihydro-1H-naphtho[1,8-de][1,3,2]diazaborinine (37)

^{1}H NMR (500 MHz, room temperature, $CDCl_3$)

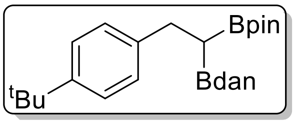

37

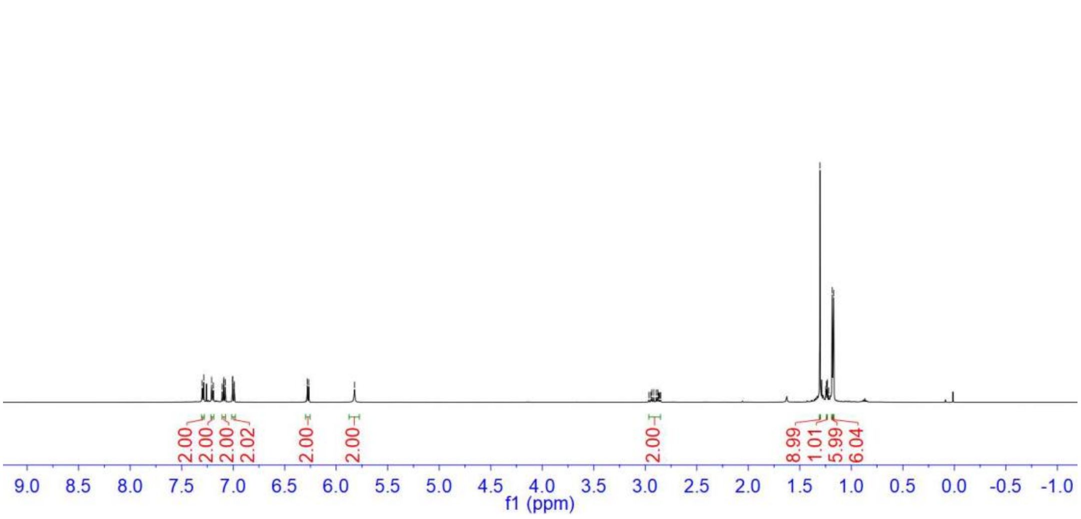

Supplementary Figure 118. ^{1}H NMR spectrum of compound 37

^{13}C NMR (126 MHz, room temperature, $CDCl_3$)

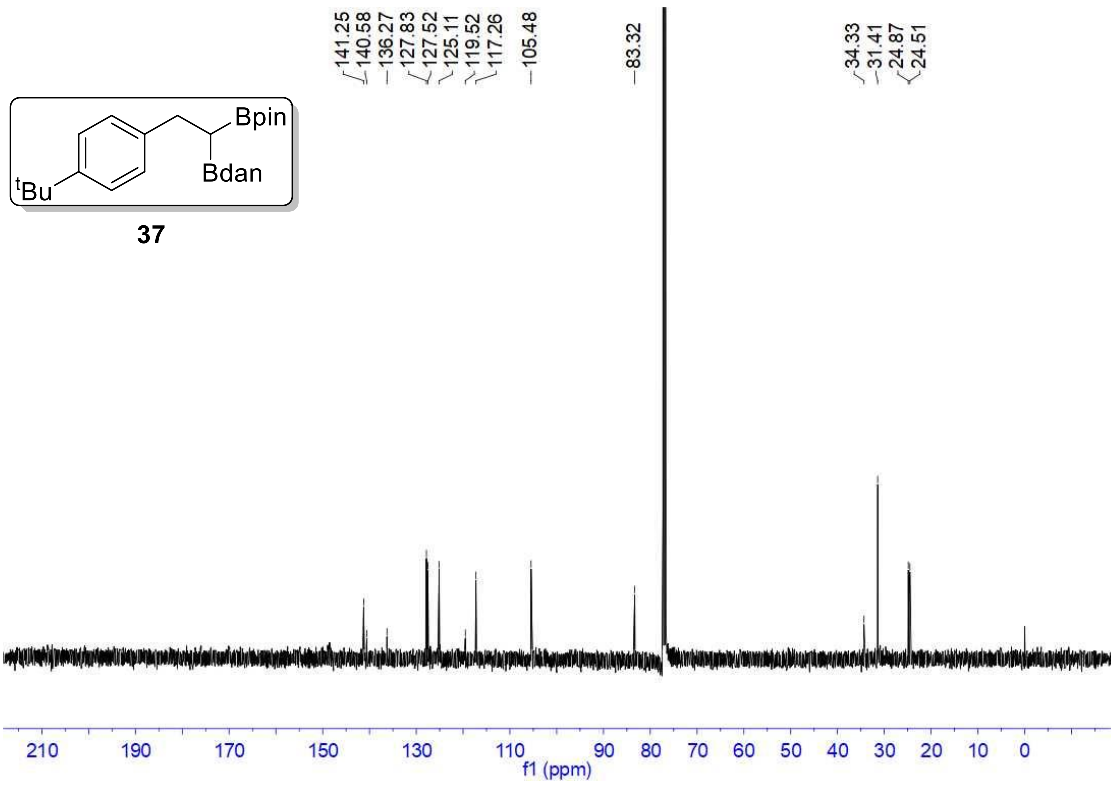

Supplementary Figure 119. ^{13}C NMR spectrum of compound 37

^{11}B NMR (160 MHz, room temperature, $CDCl_3$)

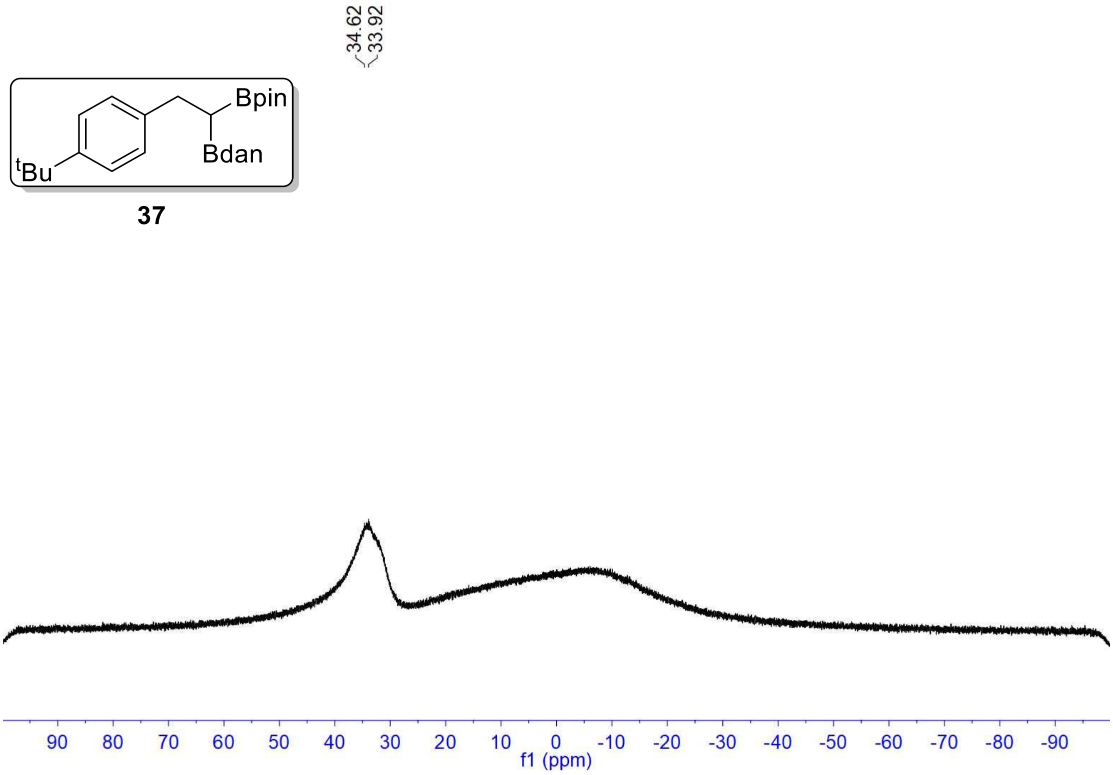

Supplementary Figure 120. ^{11}B NMR spectrum of compound 37

2-(2-(4-isopropylphenyl)-1-(4,4,5,5-tetramethyl-1,3,2-dioxaborolan-2-yl)ethyl)-2,3-dihydro-1H-naphtho[1,8-de][1,3,2]diazaborinine (38)

^{1}H NMR (500 MHz, room temperature, $CDCl_3$)

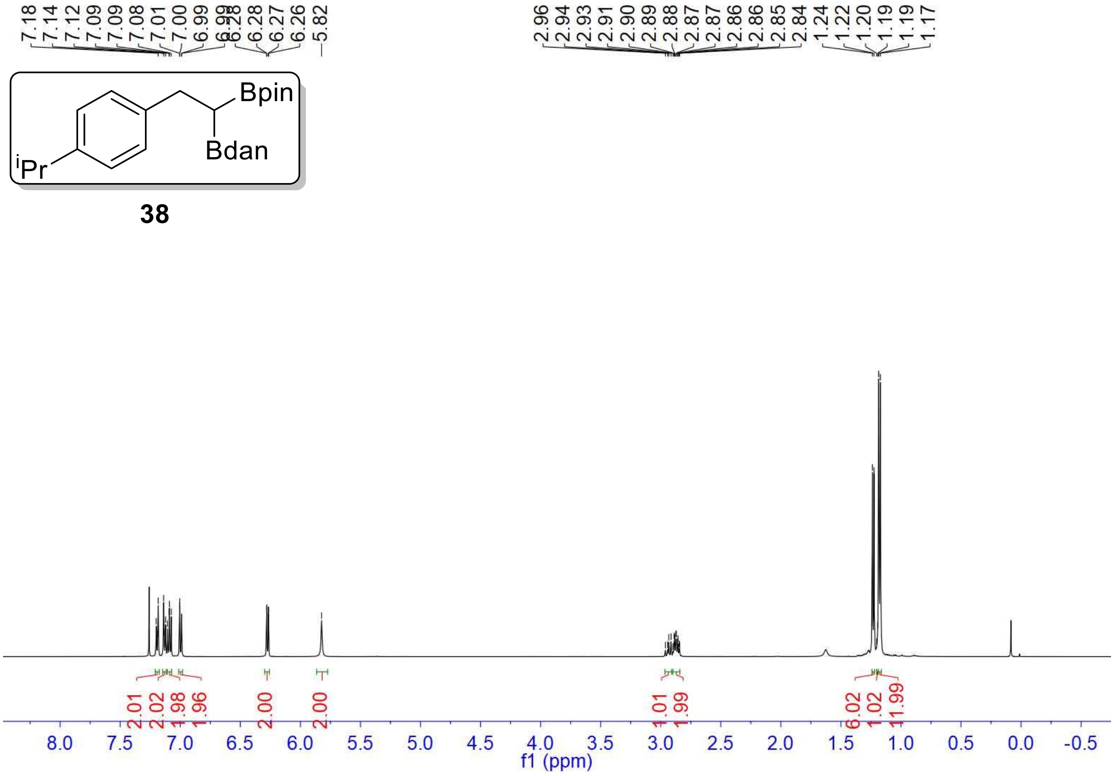

Supplementary Figure 121. ^{1}H NMR spectrum of compound 38

^{13}C NMR (126 MHz, room temperature, $CDCl_3$)

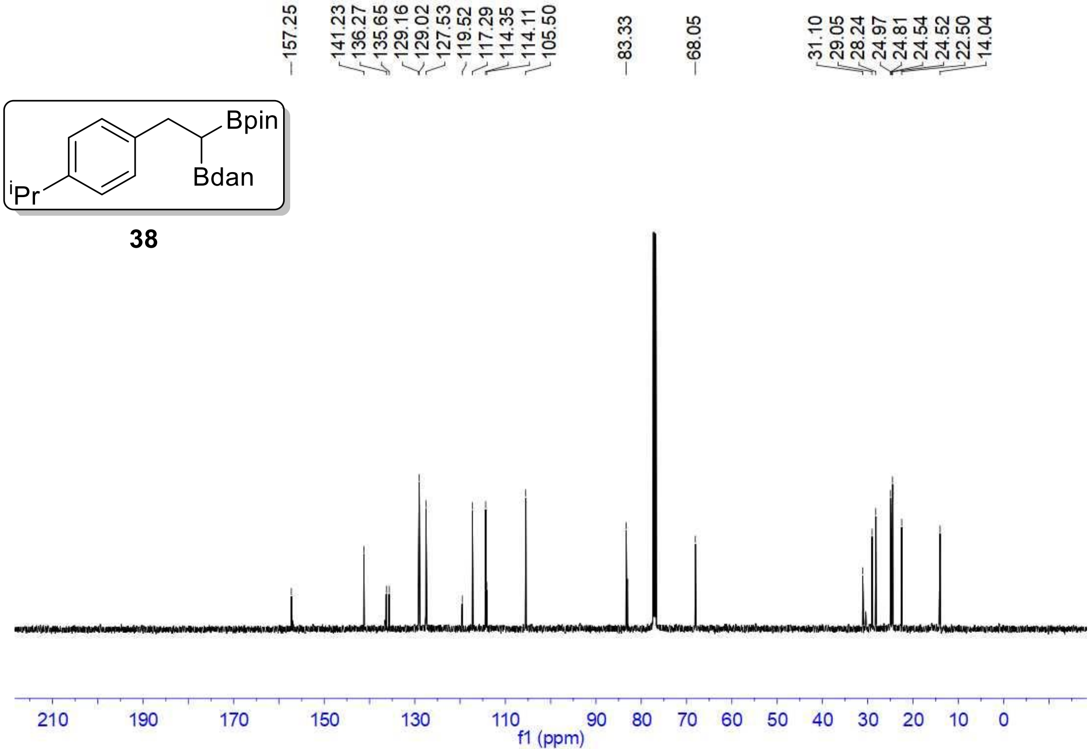

Supplementary Figure 122. ^{13}C NMR spectrum of compound 38

^{11}B NMR (160 MHz, room temperature, $CDCl_3$)

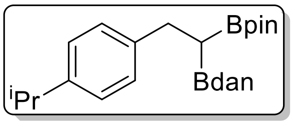

38

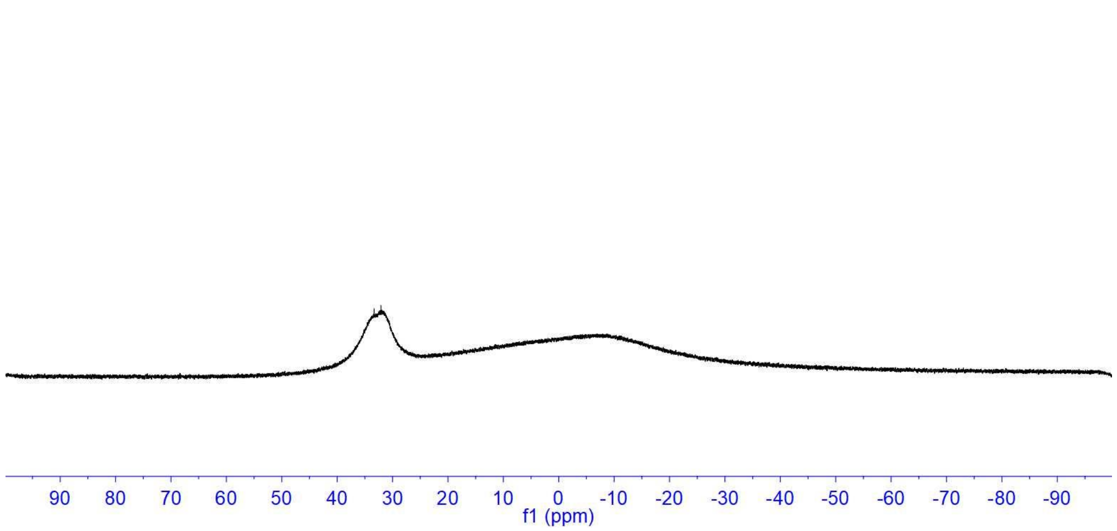

Supplementary Figure 123. ^{11}B NMR spectrum of compound 38

2-(2-(4-butylphenyl)-1-(4,4,5,5-tetramethyl-1,3,2-dioxaborolan-2-yl)ethyl)-2,3-dihydro-1H-naphtho[1,8-de][1,3,2]diazaborinine (39)

^{1}H NMR (500 MHz, room temperature, $CDCl_3$)

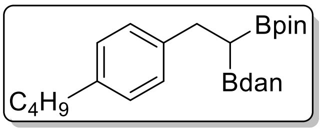

39

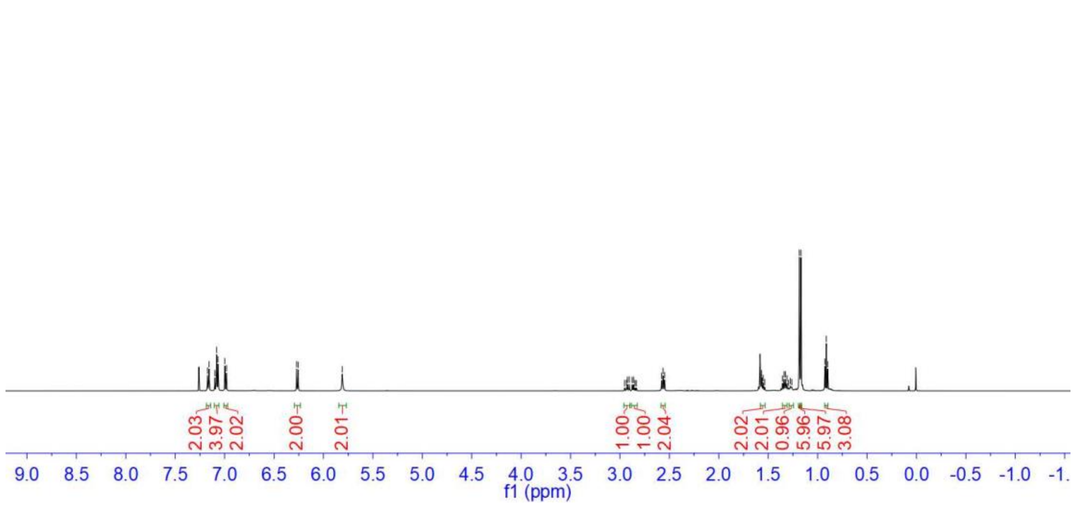

Supplementary Figure 124. ^{1}H NMR spectrum of compound 39

^{13}C NMR (126 MHz, room temperature, $CDCl_3$)

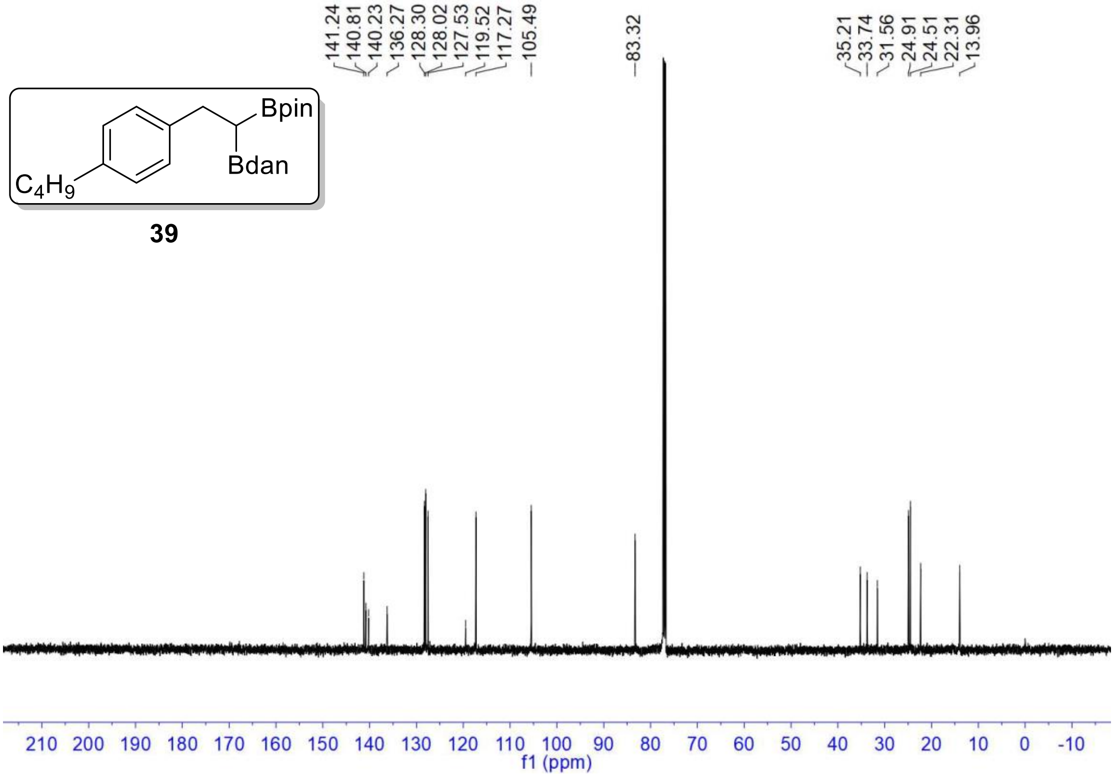

Supplementary Figure 125. ^{13}C NMR spectrum of compound 39

^{11}B NMR (160 MHz, room temperature, $CDCl_3$)

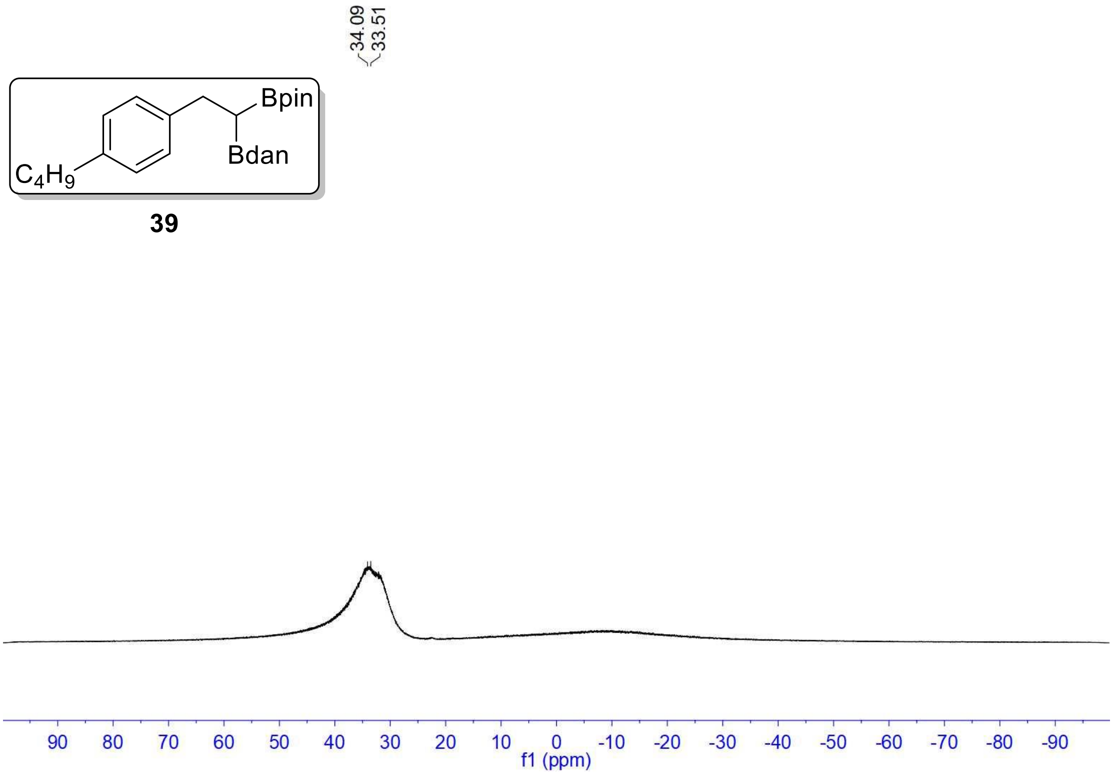

Supplementary Figure 126. ^{11}B NMR spectrum of compound 39

2-(2-(4-propylphenyl)-1-(4,4,5,5-tetramethyl-1,3,2-dioxaborolan-2-yl)ethyl)-2,3-dihydro-1H-naphtho[1,8-de][1,3,2]diazaborinine (40)

^{1}H NMR (500 MHz, room temperature, $CDCl_3$)

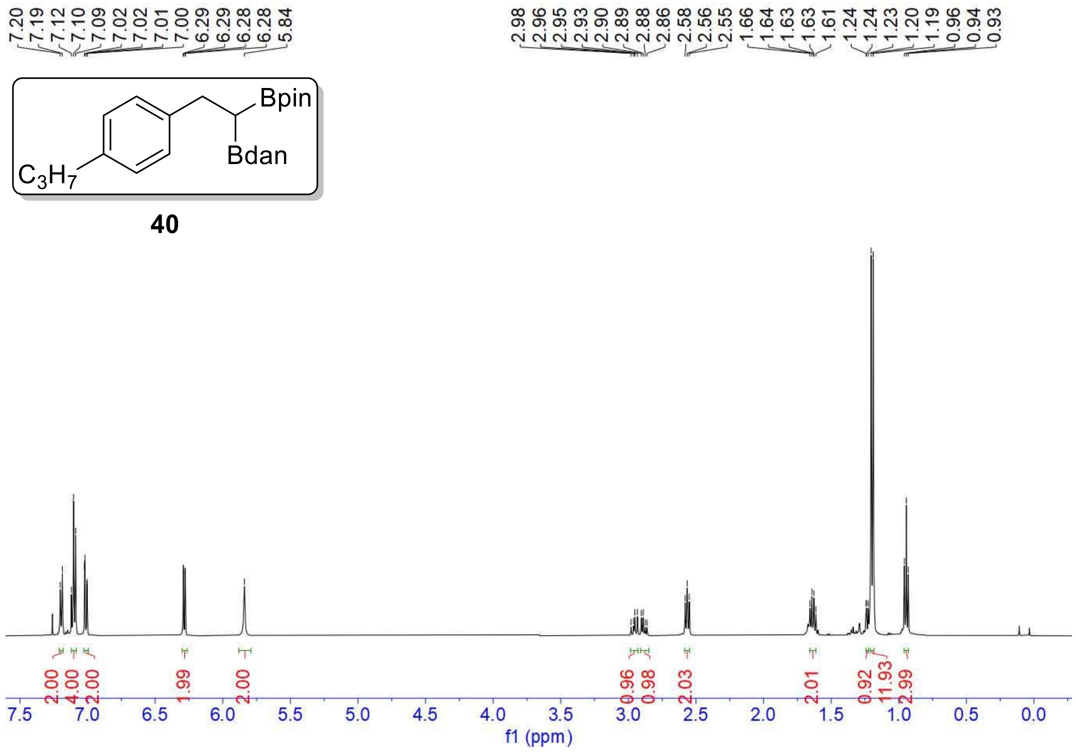

Supplementary Figure 127. ^{1}H NMR spectrum of compound 40

^{13}C NMR (126 MHz, room temperature, $CDCl_3$)

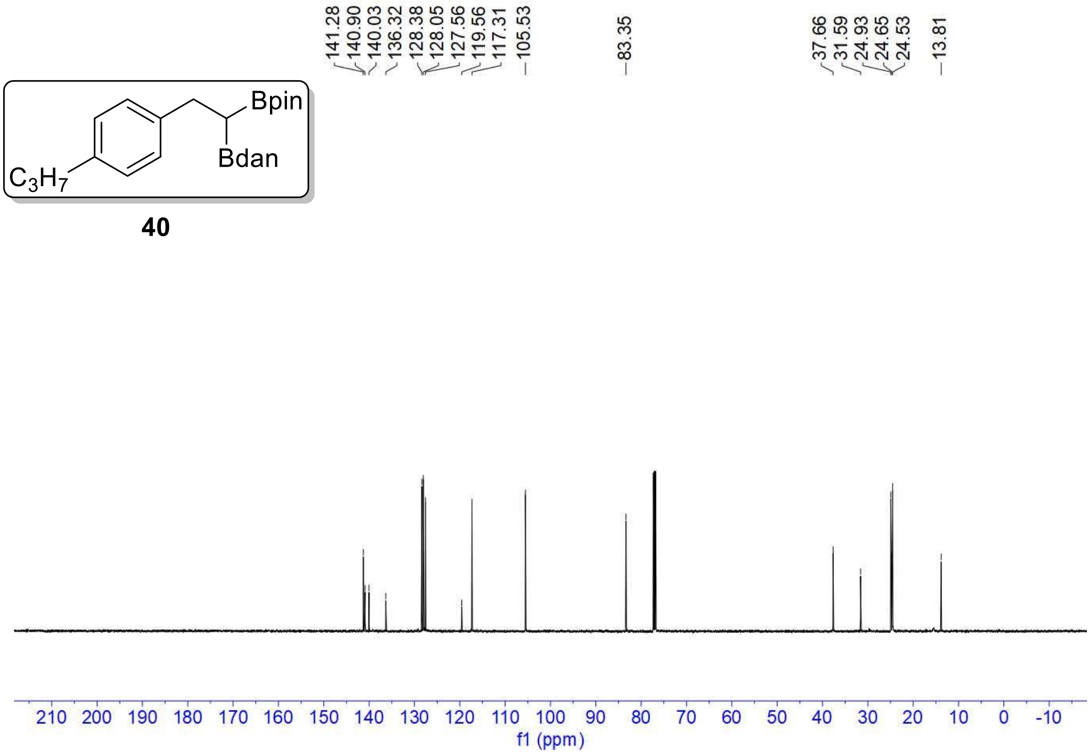

Supplementary Figure 128. ^{13}C NMR spectrum of compound 40

^{11}B NMR (160 MHz, room temperature, $CDCl_3$)

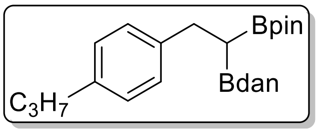

40

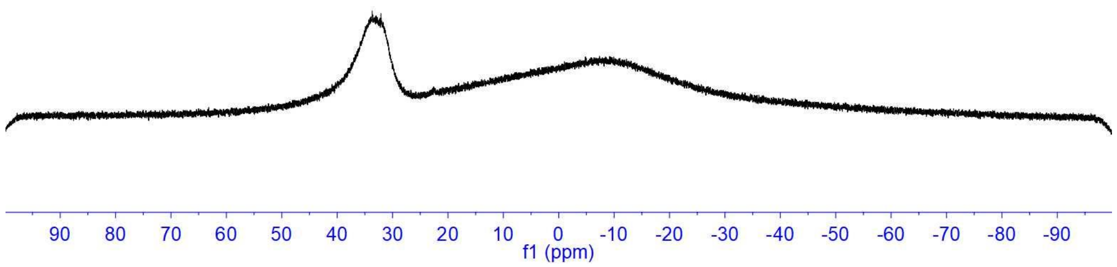

Supplementary Figure 129. ^{11}B NMR spectrum of compound 40

2-(2-([1,1'-biphenyl]-4-yl)-1-(4,4,5,5-tetramethyl-1,3,2-dioxaborolan-2-yl)ethyl)-2,3-dihydro-1H-naphtho[1,8-de][1,3,2]diazaborinine (41)

^{1}H NMR (500 MHz, room temperature, $CDCl_3$)

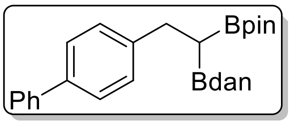

41

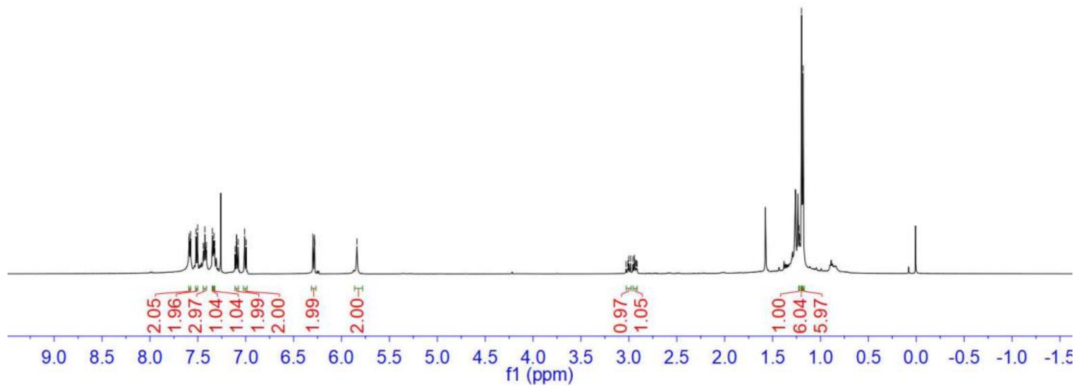

Supplementary Figure 130. ^{1}H NMR spectrum of compound 41

^{13}C NMR (126 MHz, room temperature, $CDCl_3$)

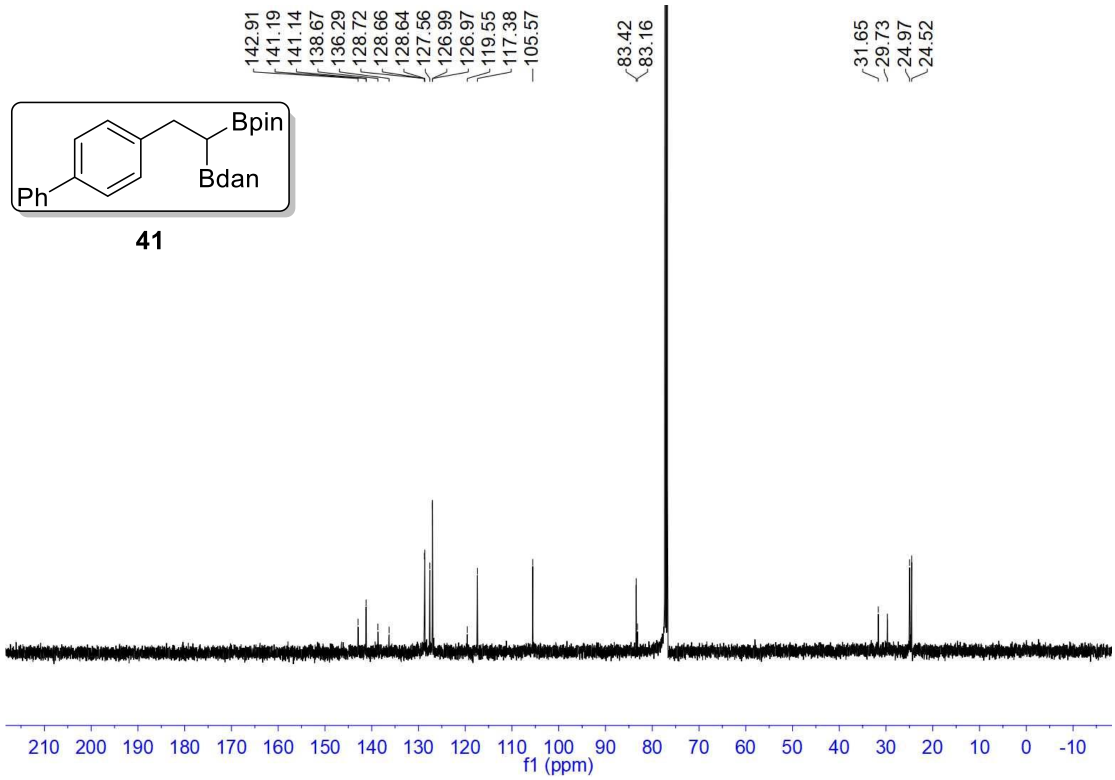

Supplementary Figure 131. ^{13}C NMR spectrum of compound 41

^{11}B NMR (160 MHz, room temperature, $CDCl_3$)

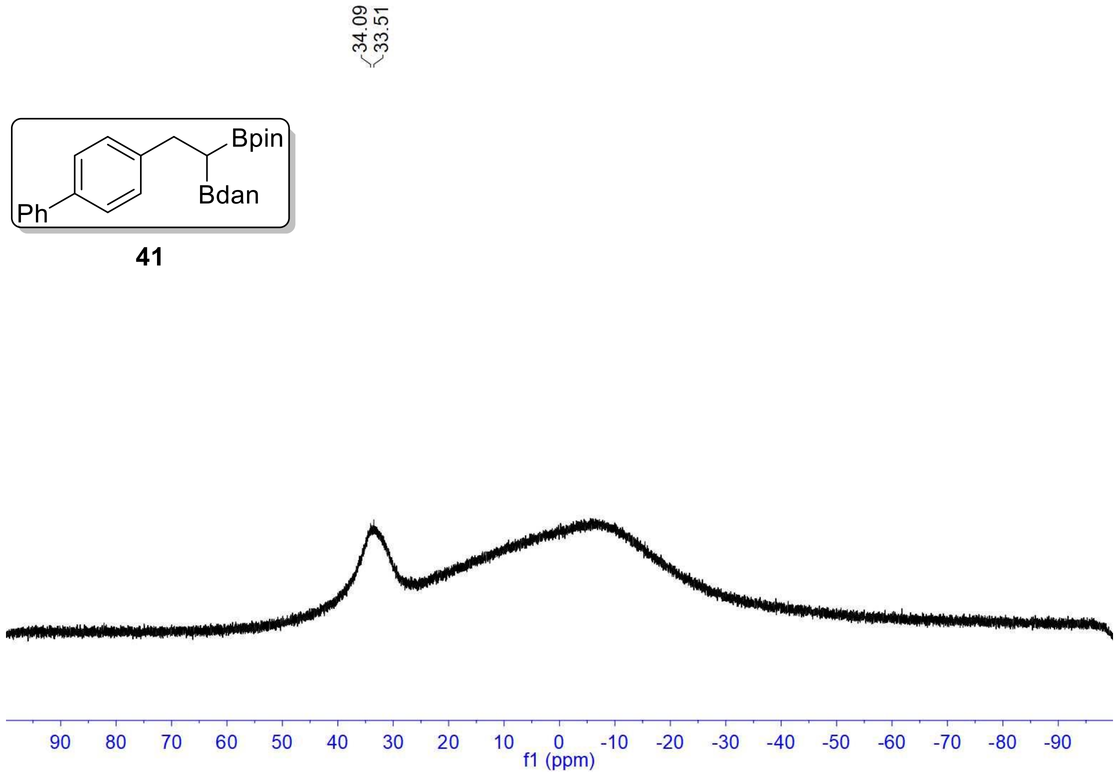

Supplementary Figure 132. ^{11}B NMR spectrum of compound 41

2-(2-(4-methoxyphenyl)-1-(4,4,5,5-tetramethyl-1,3,2-dioxaborolan-2-yl)ethyl)-2,3-dihydro-1H-naphtho[1,8-de][1,3,2]diazaborinine (42)

^{1}H NMR (500 MHz, room temperature, CDCl$_3$)

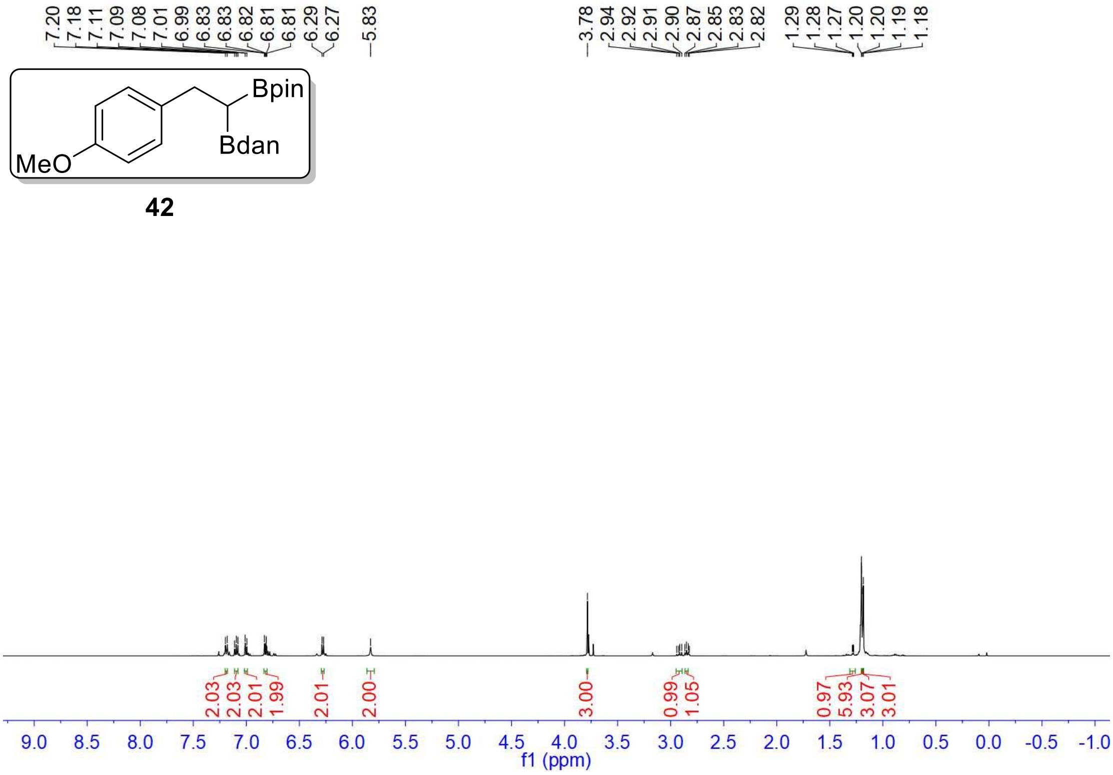

Supplementary Figure 133. ^{1}H NMR spectrum of compound 42

^{13}C NMR (126 MHz, room temperature, CDCl$_3$)

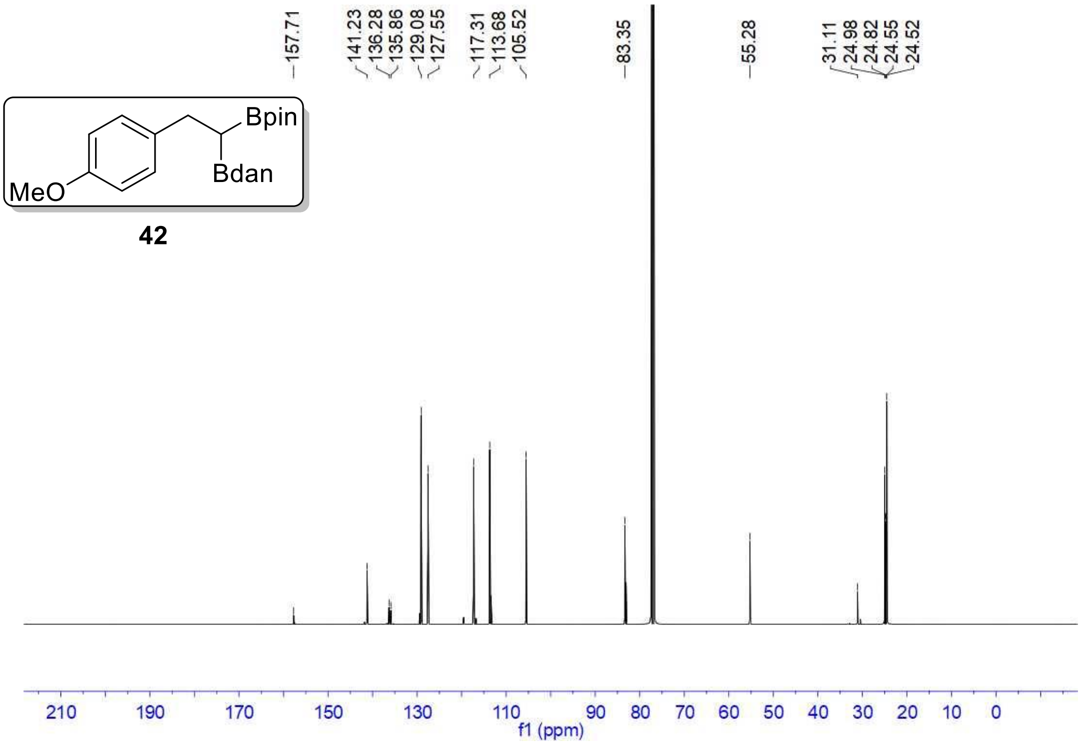

Supplementary Figure 134. ^{13}C NMR spectrum of compound 42

^{11}B NMR (160 MHz, room temperature, $CDCl_3$)

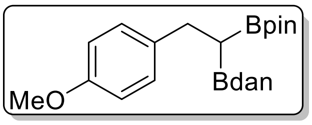

42

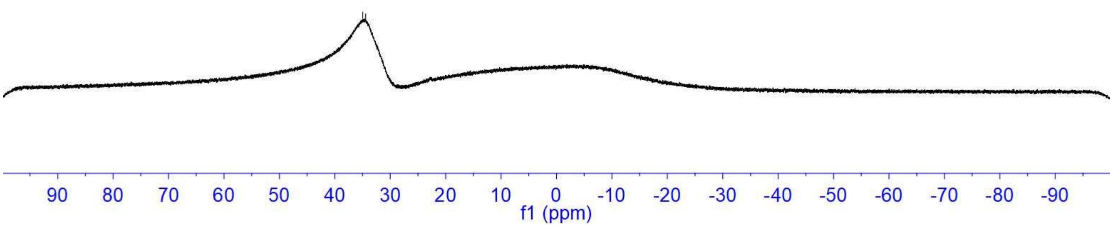

Supplementary Figure 135. ^{11}B NMR spectrum of compound 42

2-(2-(3-methoxyphenyl)-1-(4,4,5,5-tetramethyl-1,3,2-dioxaborolan-2-yl)ethyl)-2,3-dihydro-1H-naphtho[1,8-de][1,3,2]diazaborinine (43)

^{1}H NMR (500 MHz, room temperature, $CDCl_3$)

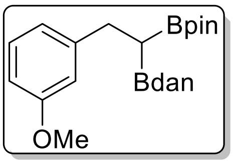

43

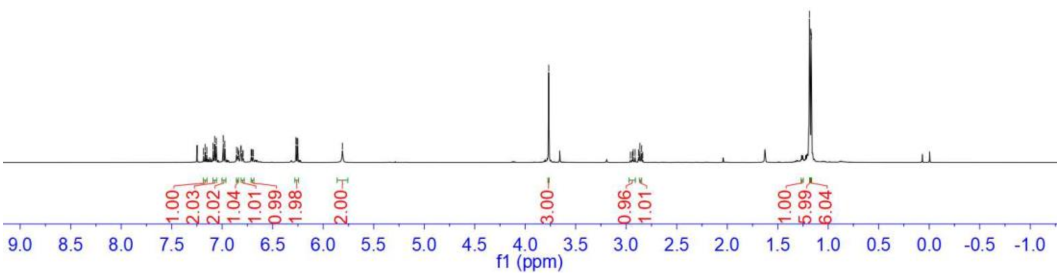

Supplementary Figure 136. ^{1}H NMR spectrum of compound 43

^{13}C NMR (126 MHz, room temperature, $CDCl_3$)

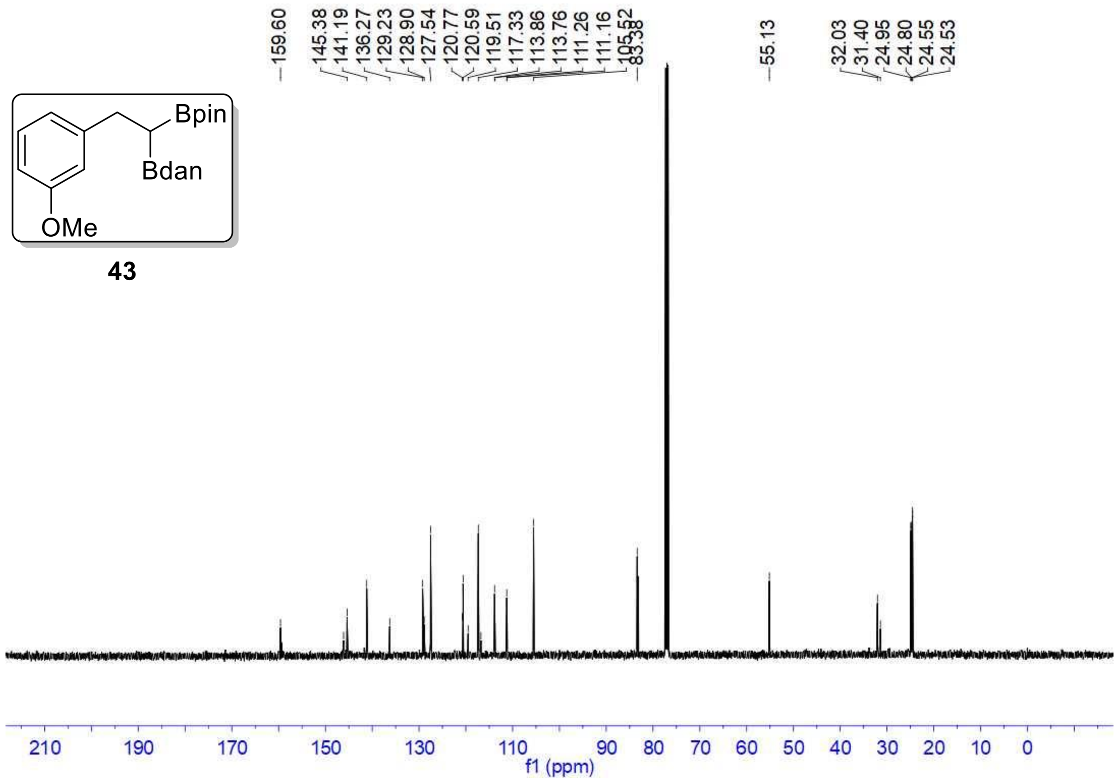

Supplementary Figure 137. ^{13}C NMR spectrum of compound 43

^{11}B NMR (160 MHz, room temperature, $CDCl_3$)

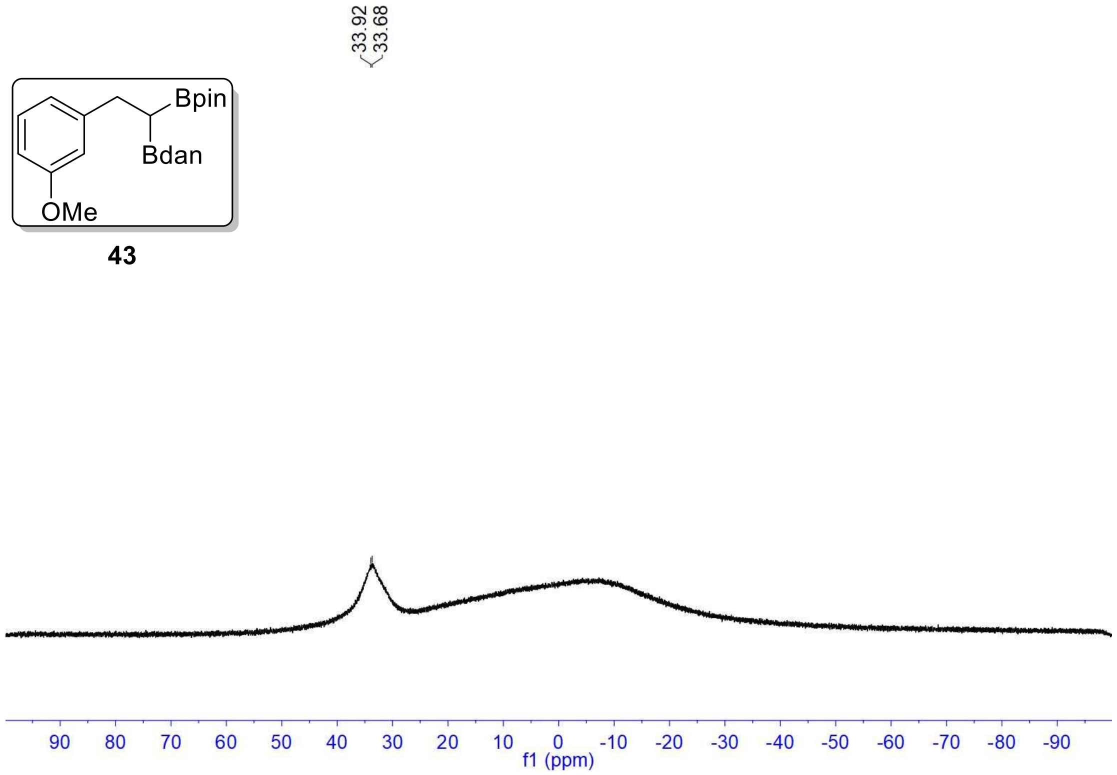

Supplementary Figure 138. ^{11}B NMR spectrum of compound 43

2-(2-(4-(pentyloxy)phenyl)-1-(4,4,5,5-tetramethyl-1,3,2-dioxaborolan-2-yl)ethyl)-2,3-dihydro-1H-naphtho[1,8-de][1,3,2]diazaborinine (44)

^{1}H NMR (500 MHz, room temperature, $CDCl_3$)

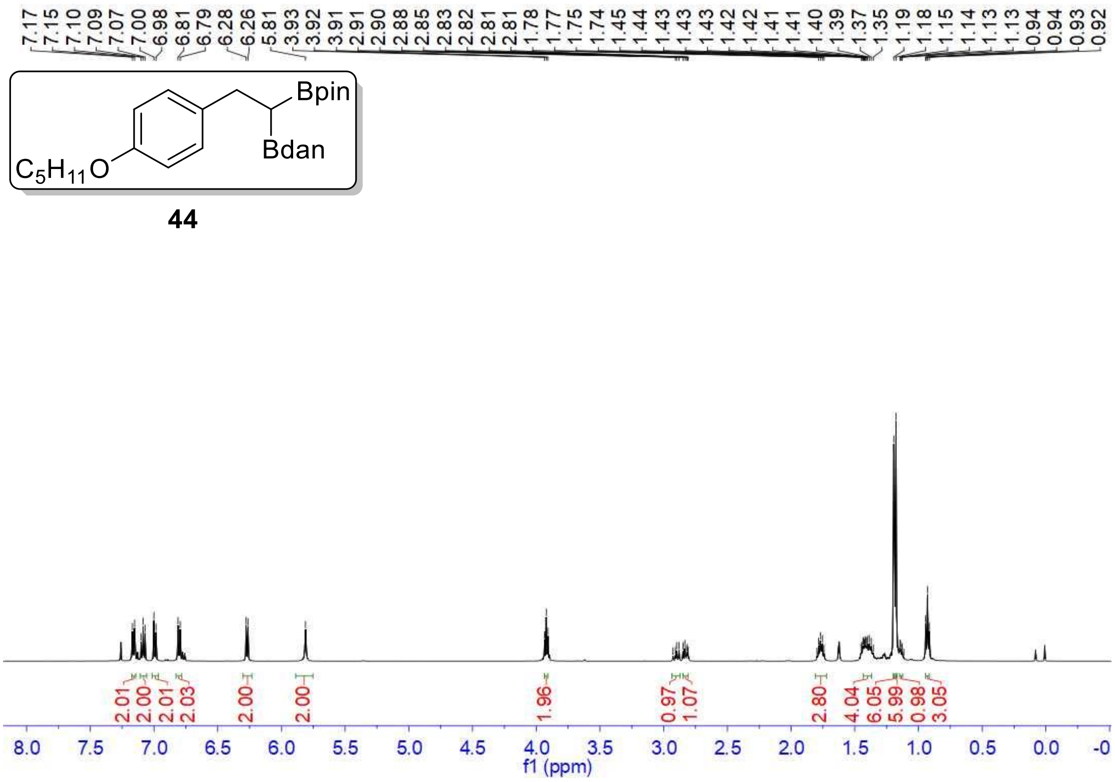

Supplementary Figure 139. ^{1}H NMR spectrum of compound 44

^{13}C NMR (126 MHz, room temperature, $CDCl_3$)

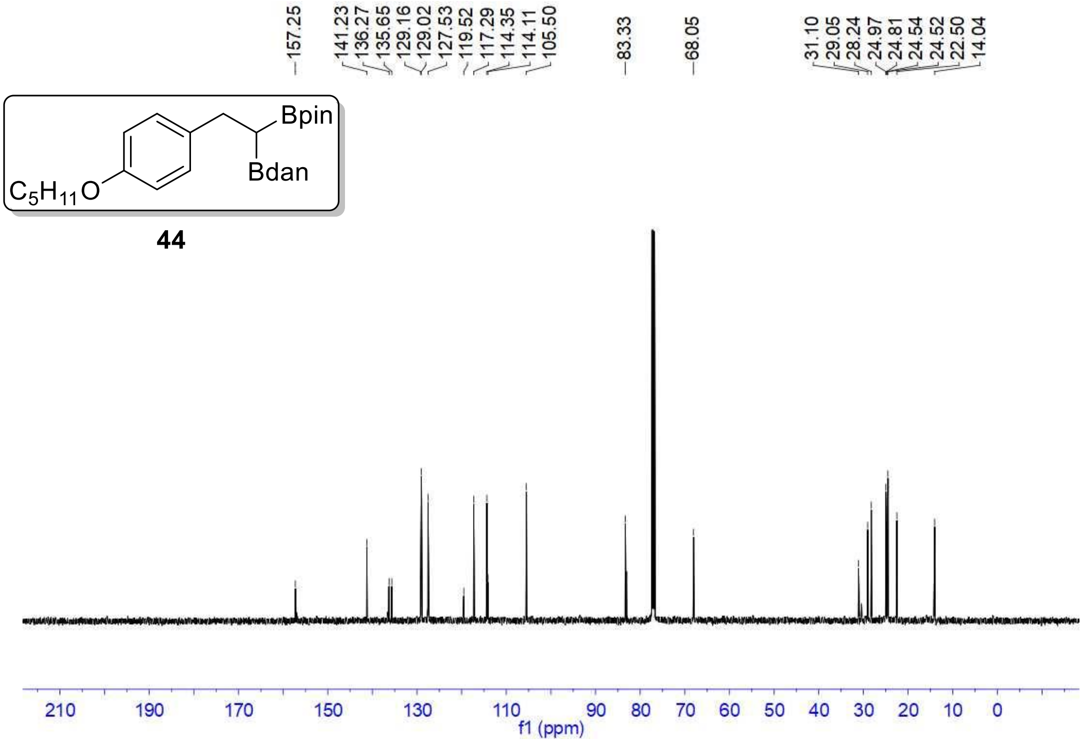

Supplementary Figure 140. ^{13}C NMR spectrum of compound 44

^{11}B NMR (160 MHz, room temperature, $CDCl_3$)

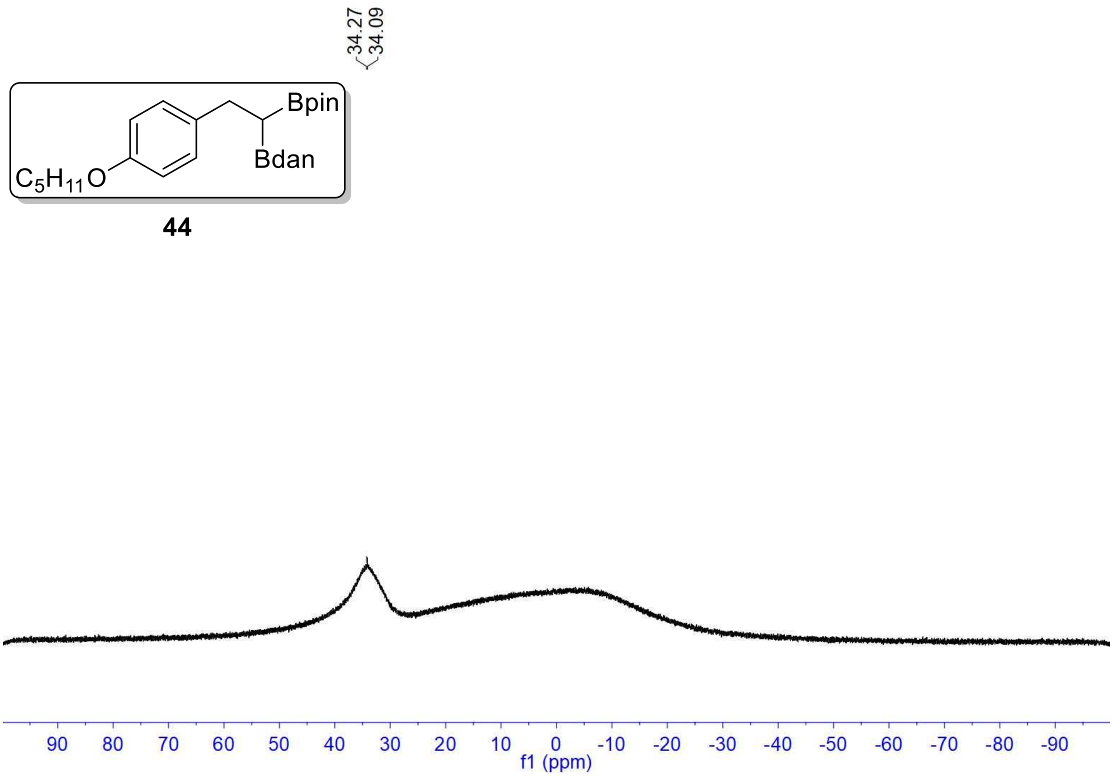

Supplementary Figure 141. ^{11}B NMR spectrum of compound 44

4-(2-(1H-naphtho[1,8-de][1,3,2]diazaborinin-2(3H)-yl)-2-(4,4,5,5-tetramethyl-1,3,2-dioxaborolan-2-yl)ethyl)-N,N-dimethylaniline (45)

^{1}H NMR (500 MHz, room temperature, $CDCl_3$)

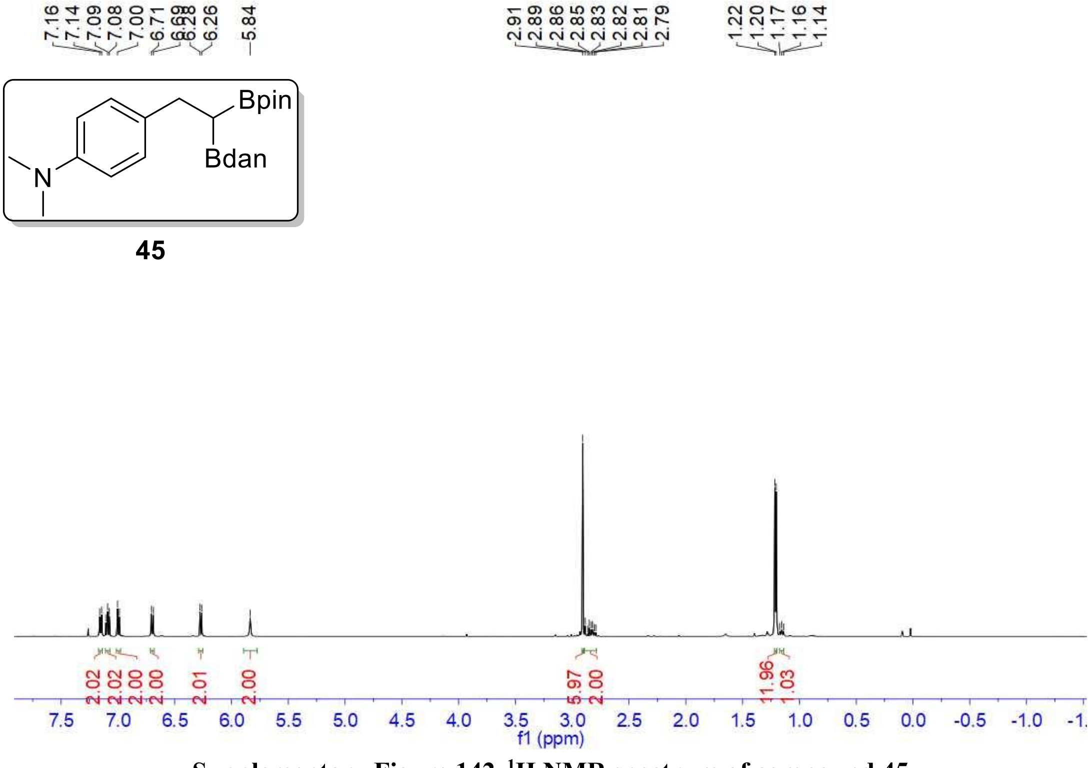

Supplementary Figure 142. ^{1}H NMR spectrum of compound 45

^{13}C NMR (126 MHz, room temperature, $CDCl_3$)

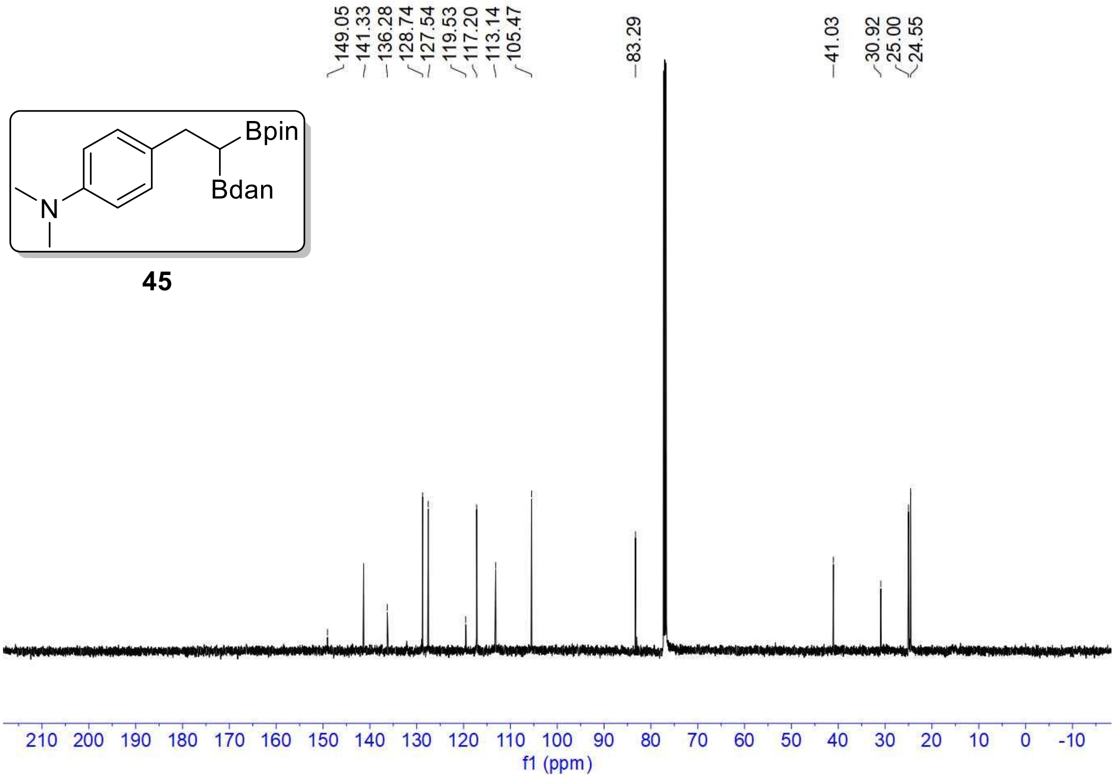

Supplementary Figure 143. ^{13}C NMR spectrum of compound 45

^{11}B NMR (160 MHz, room temperature, $CDCl_3$)

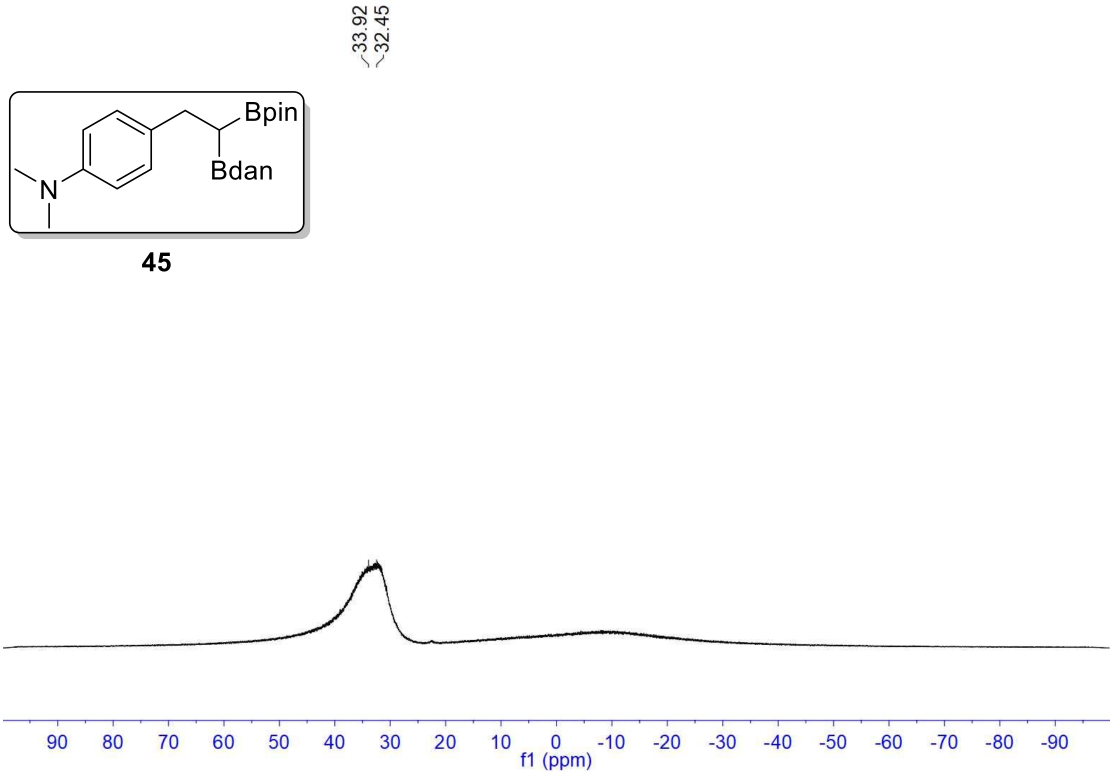

Supplementary Figure 144. ^{11}B NMR spectrum of compound 45

methyl 4-(2-(1H-naphtho[1,8-de][1,3,2]diazaborinin-2(3H)-yl) -2-(4,4,5,5-tetramethyl-1,3,2-dioxaborolan-2-yl)ethyl)benzoate (46)

^{1}H NMR (500 MHz, room temperature, CDCl$_3$)

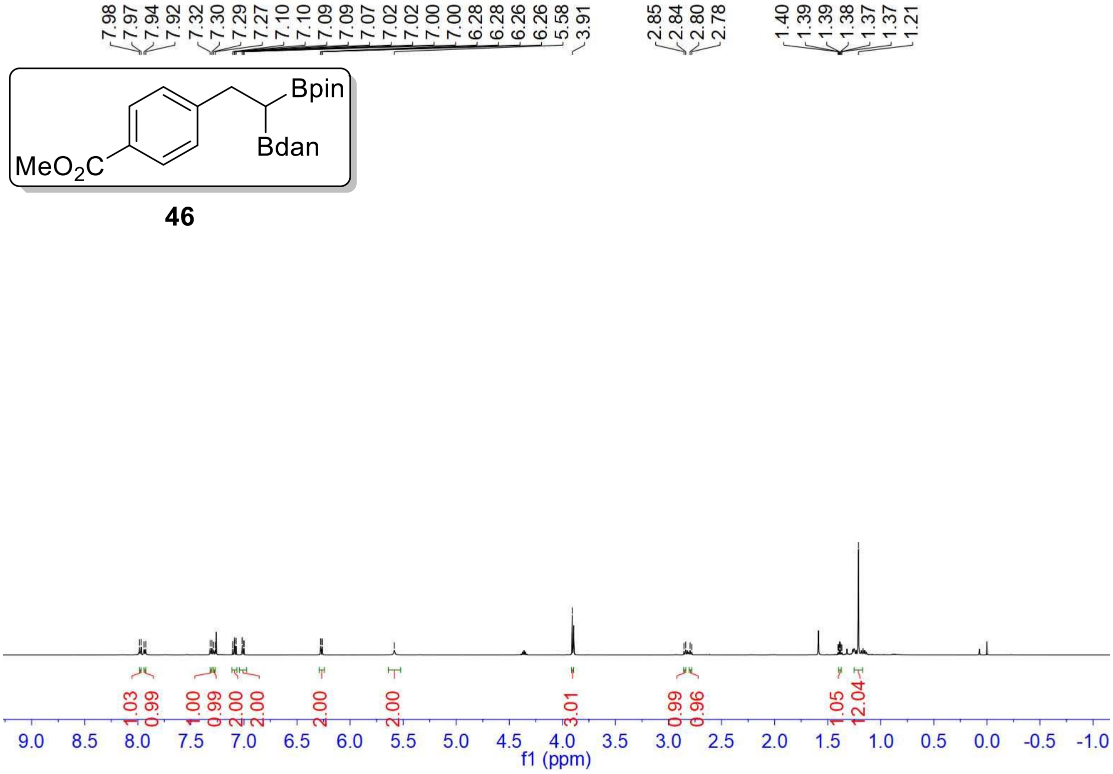

Supplementary Figure 145. ^{1}H NMR spectrum of compound 46

^{13}C NMR (126 MHz, room temperature, CDCl$_3$)

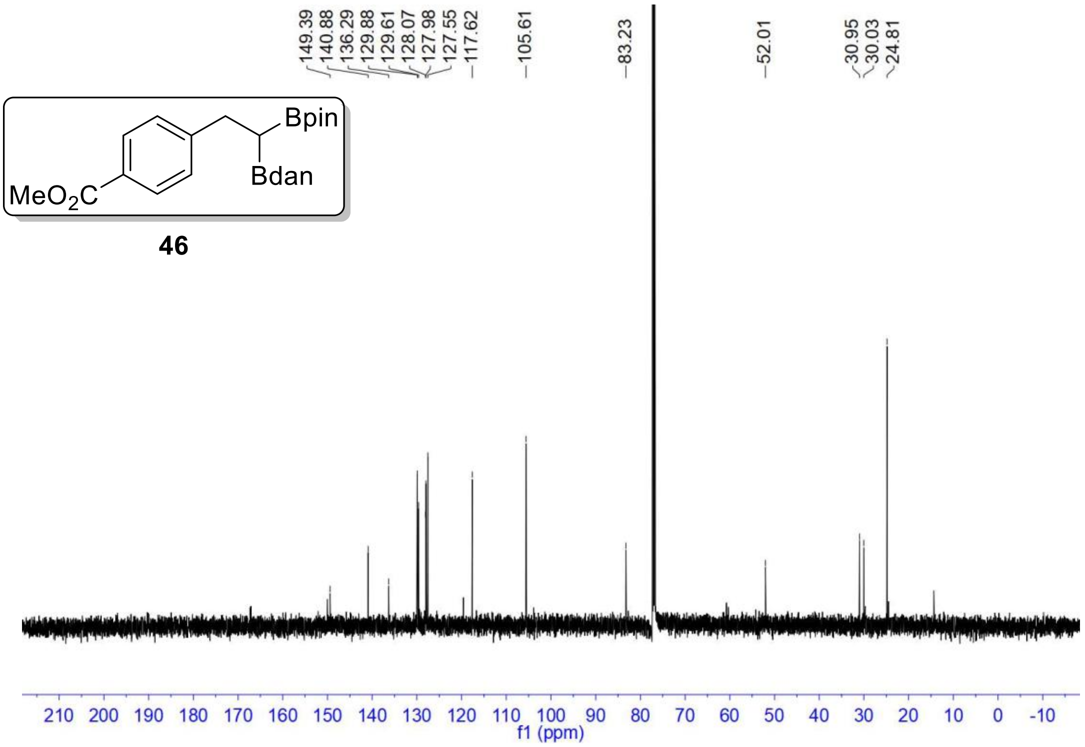

Supplementary Figure 146. ^{13}C NMR spectrum of compound 46

^{11}B NMR (160 MHz, room temperature, $CDCl_3$)

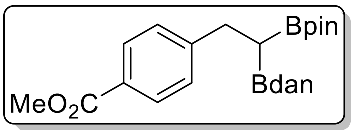

46

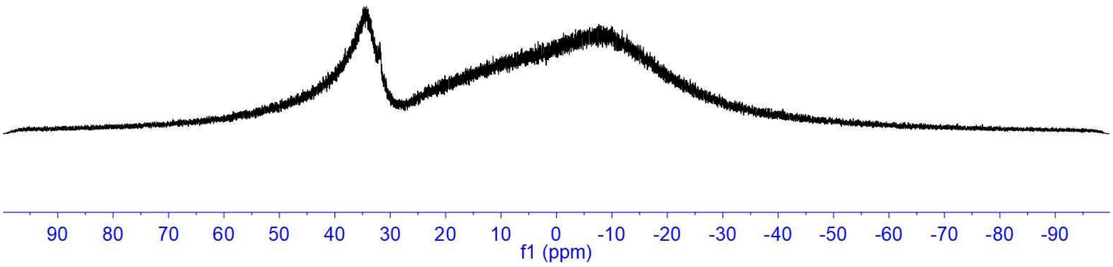

Supplementary Figure 147. ^{11}B NMR spectrum of compound 46

2-(1-(4,4,5,5-tetramethyl-1,3,2-dioxaborolan-2-yl)-2-(4-(trifluoromethoxy)phenyl)ethyl)-2,3-dihydro-1H-naphtho[1,8-de][1,3,2]diazaborinine (47)

^{1}H NMR (500 MHz, room temperature, $CDCl_3$)

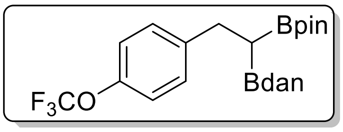

47

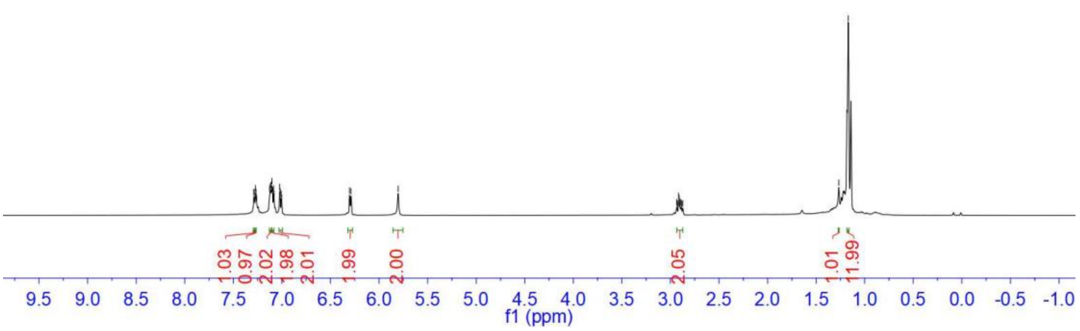

Supplementary Figure 148. ^{1}H NMR spectrum of compound 47

^{13}C NMR (126 MHz, room temperature, $CDCl_3$)

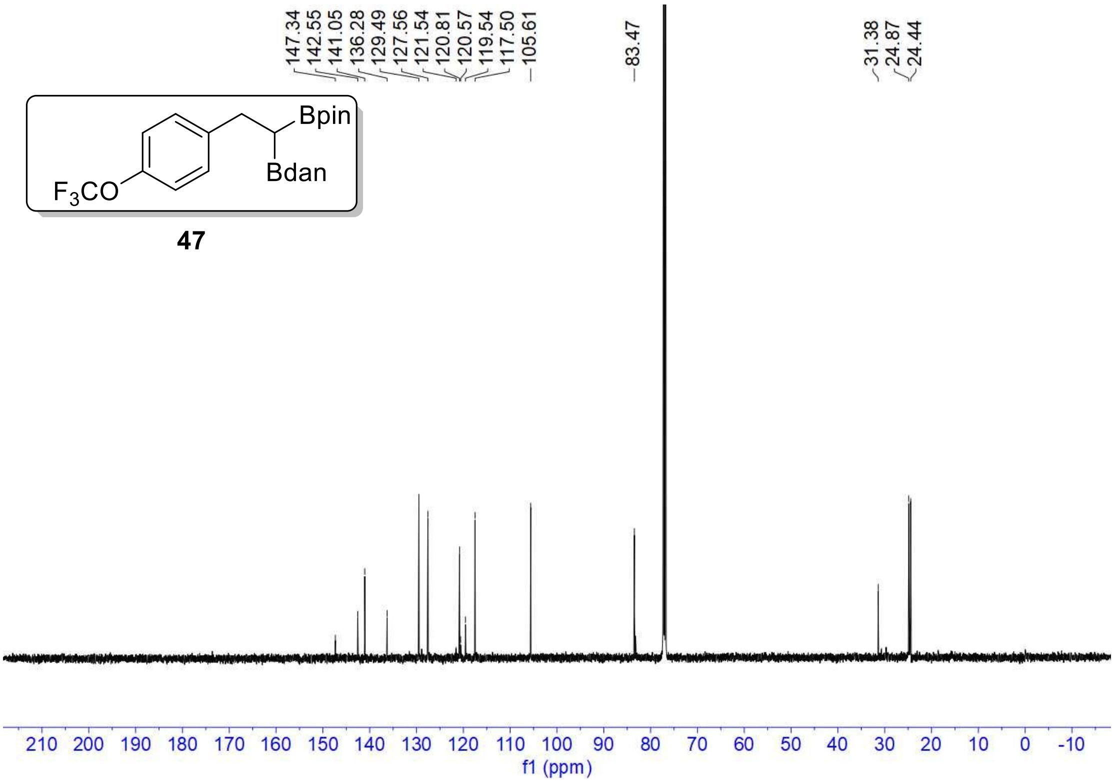

Supplementary Figure 149. ^{13}C NMR spectrum of compound 47

^{11}B NMR (160 MHz, room temperature, $CDCl_3$)

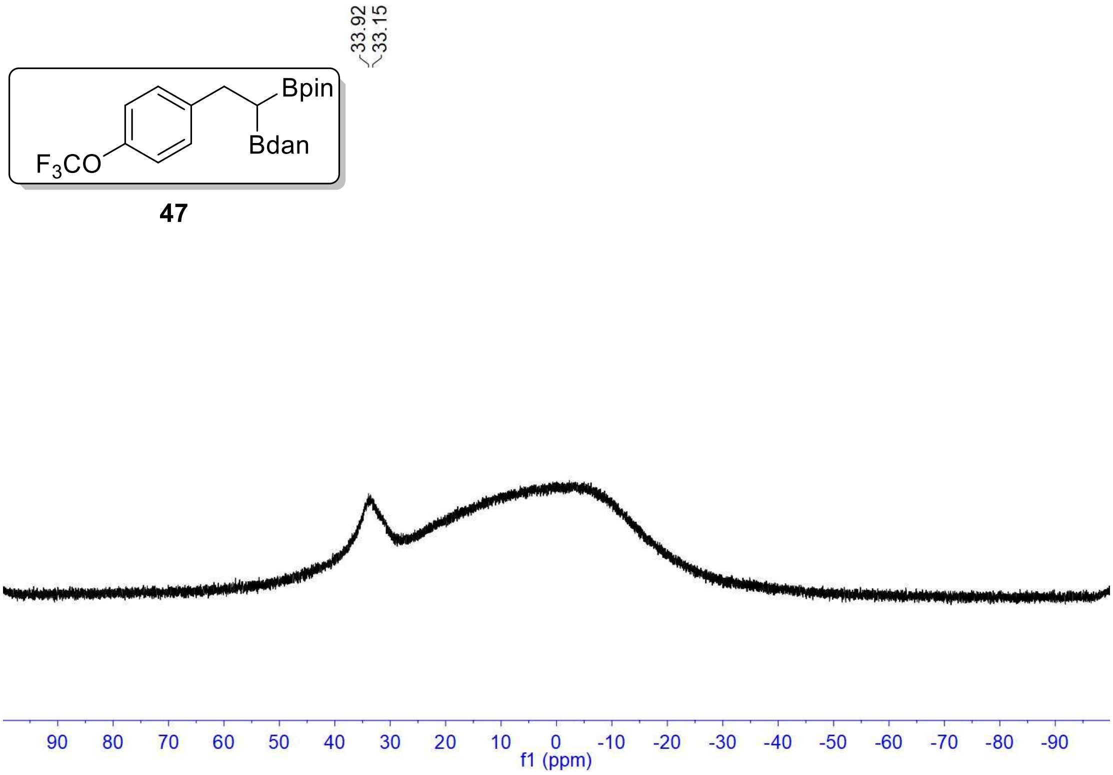

Supplementary Figure 150. ^{11}B NMR spectrum of compound 47

^{19}F NMR (126 MHz, room temperature, $CDCl_3$)

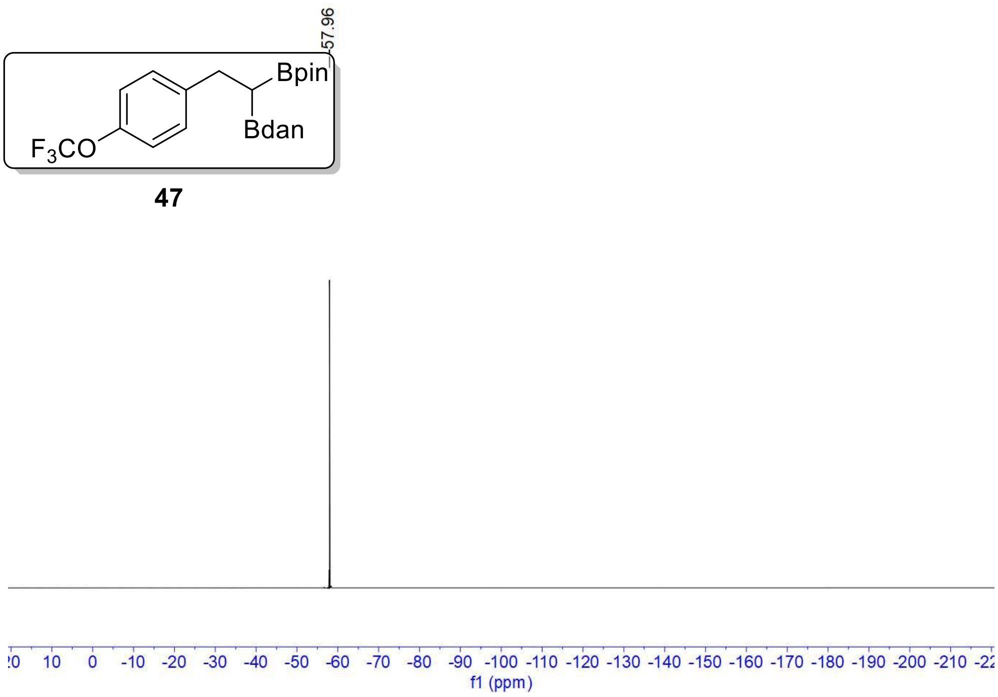

Supplementary Figure 151. ^{19}F NMR spectrum of compound 47

2-(2-(4-fluorophenyl)-1-(4,4,5,5-tetramethyl-1,3,2-dioxaborolan-2-yl) ethyl)-2,3-dihydro-1H-naphtho[1,8-de][1,3,2]diazaborinine (48)

1H NMR (500 MHz, room temperature, $CDCl_3$)

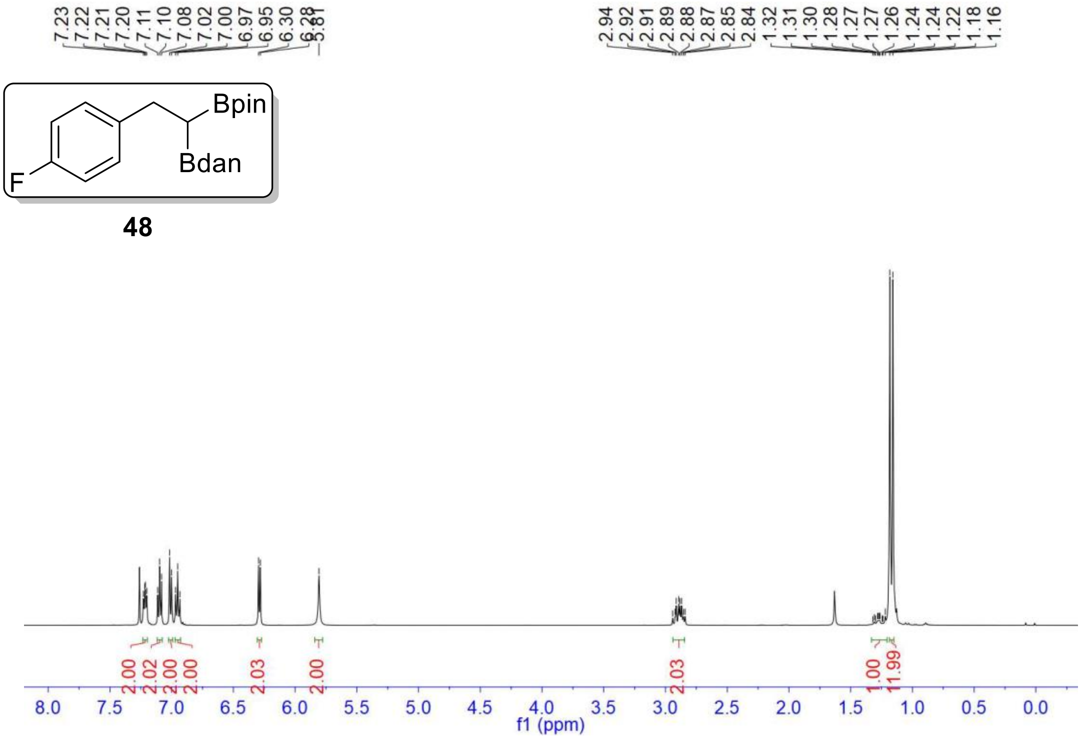

Supplementary Figure 152. ^{1}H NMR spectrum of compound 48

^{13}C NMR (126 MHz, room temperature, $CDCl_3$)

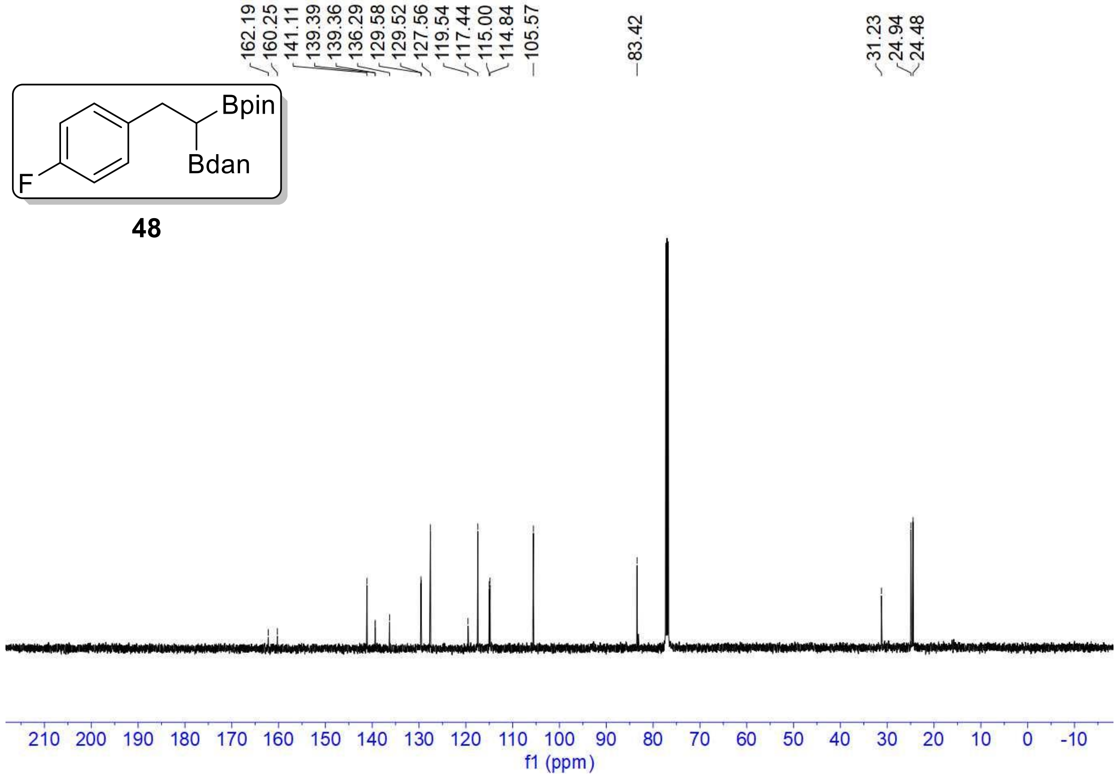

Supplementary Figure 153. ^{13}C NMR spectrum of compound 48

^{11}B NMR (160 MHz, room temperature, $CDCl_3$)

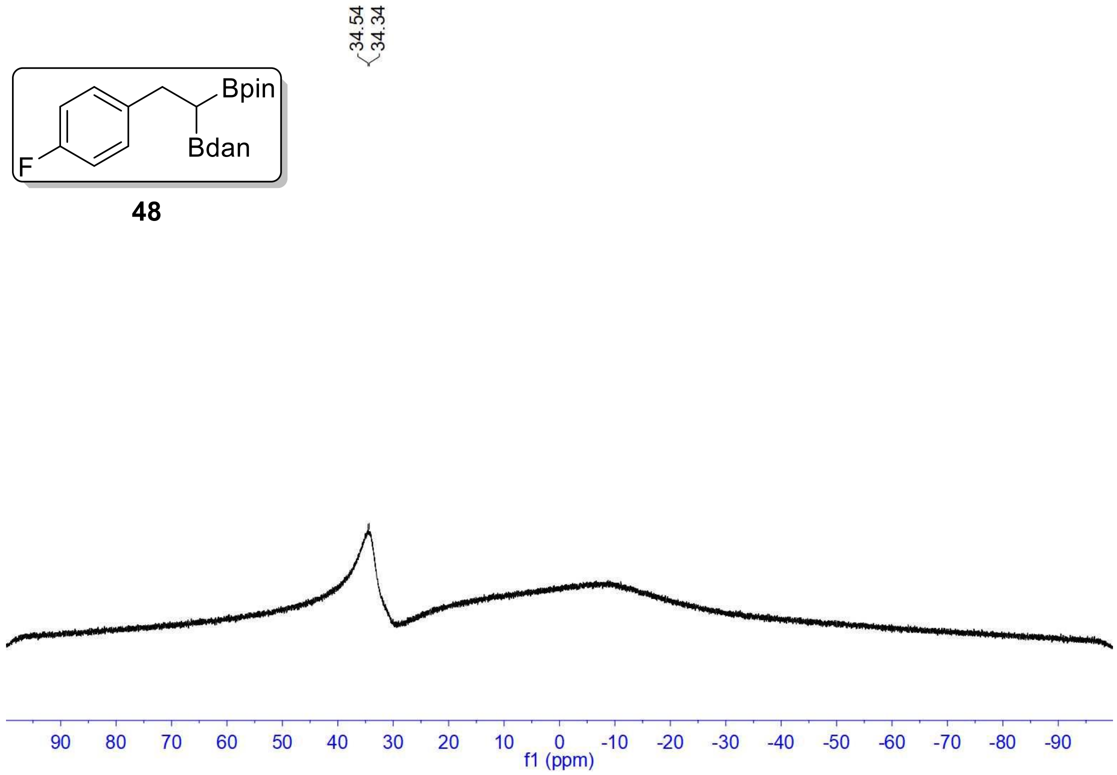

Supplementary Figure 154. ^{11}B NMR spectrum of compound 48

^{19}F NMR (126 MHz, room temperature, $CDCl_3$)

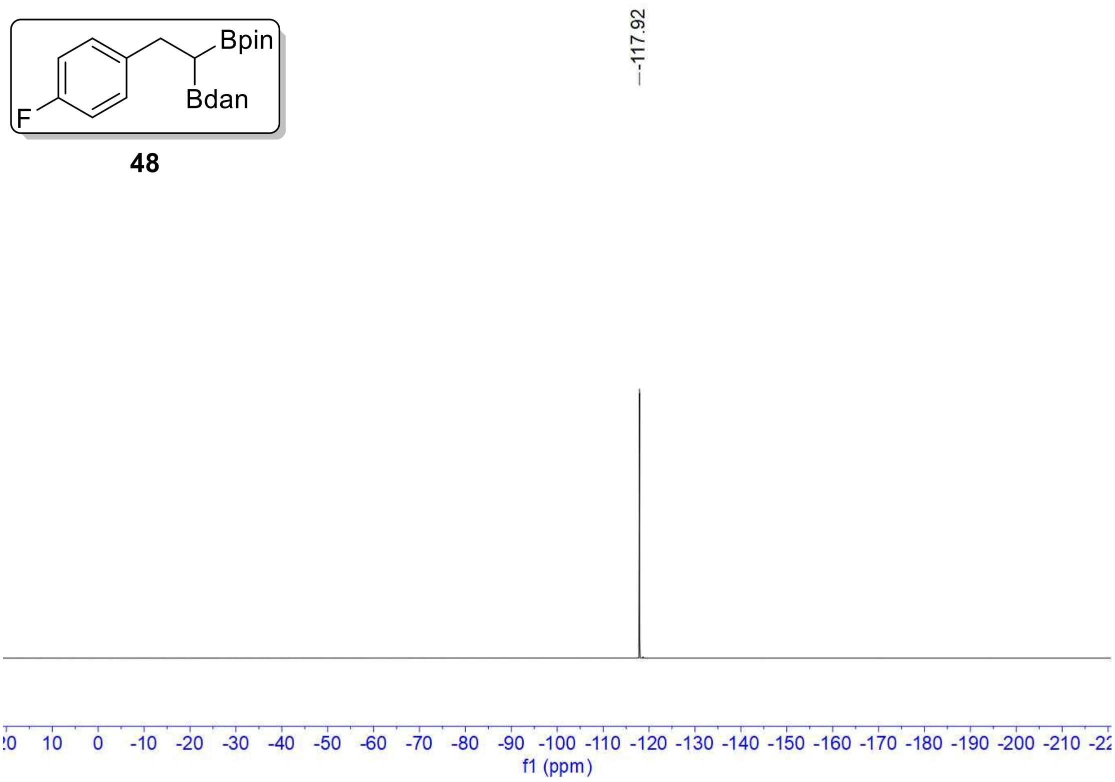

Supplementary Figure 155. ^{19}F NMR spectrum of compound 48

2-(2-(4-chlorophenyl)-1-(4,4,5,5-tetramethyl-1,3,2-dioxaborolan-2-yl)ethyl) -2,3-dihydro-1H-naphtho[1,8-de][1,3,2]diazaborinine (49)

^{1}H NMR (500 MHz, room temperature, $CDCl_3$)

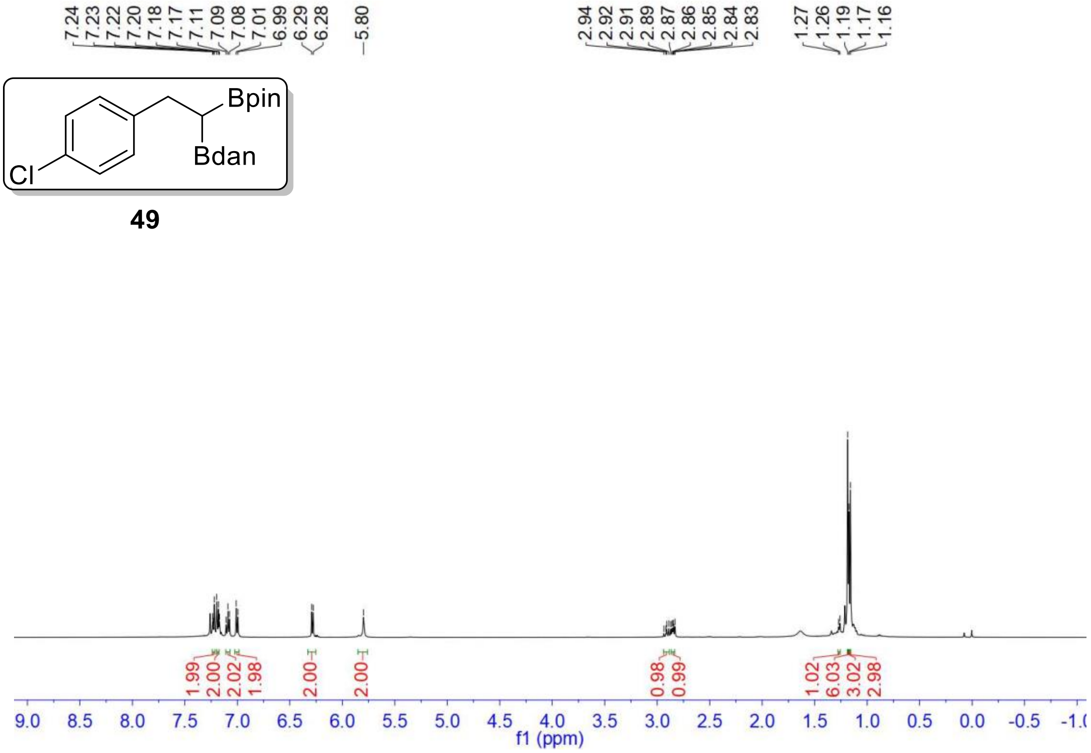

Supplementary Figure 156. ^{1}H NMR spectrum of compound 49

^{13}C NMR (126 MHz, room temperature, $CDCl_3$)

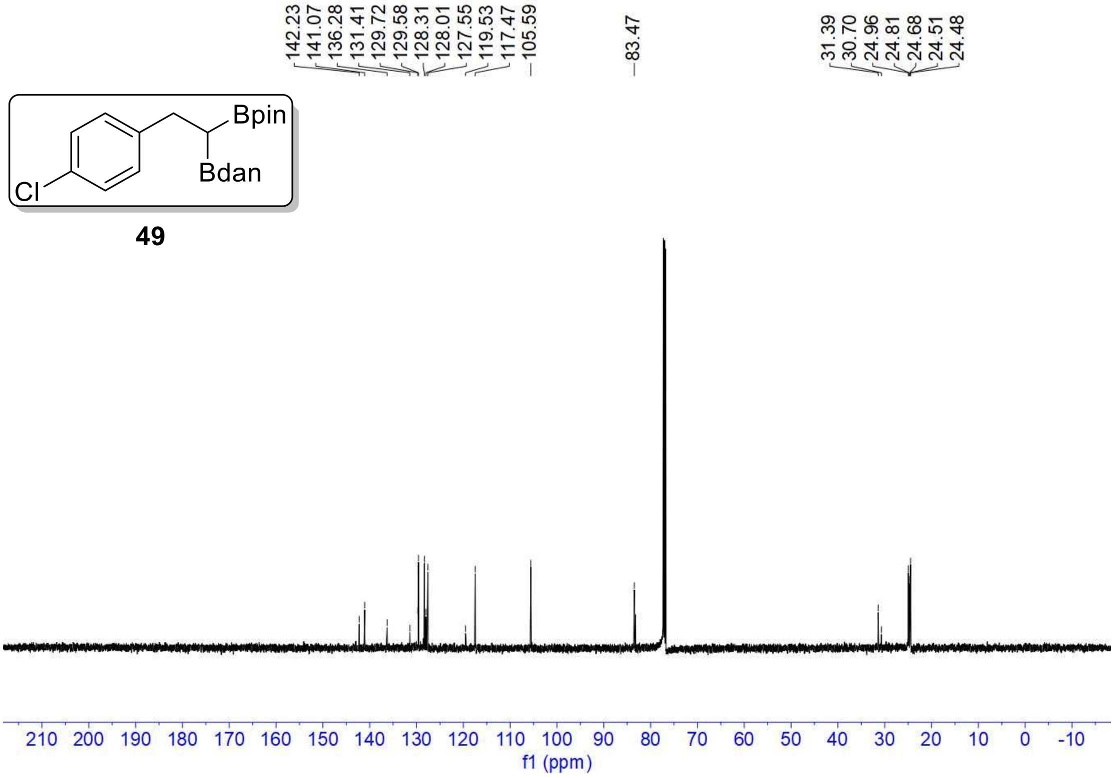

49

Supplementary Figure 157. ^{13}C NMR spectrum of compound 49

^{11}B NMR (160 MHz, room temperature, $CDCl_3$)

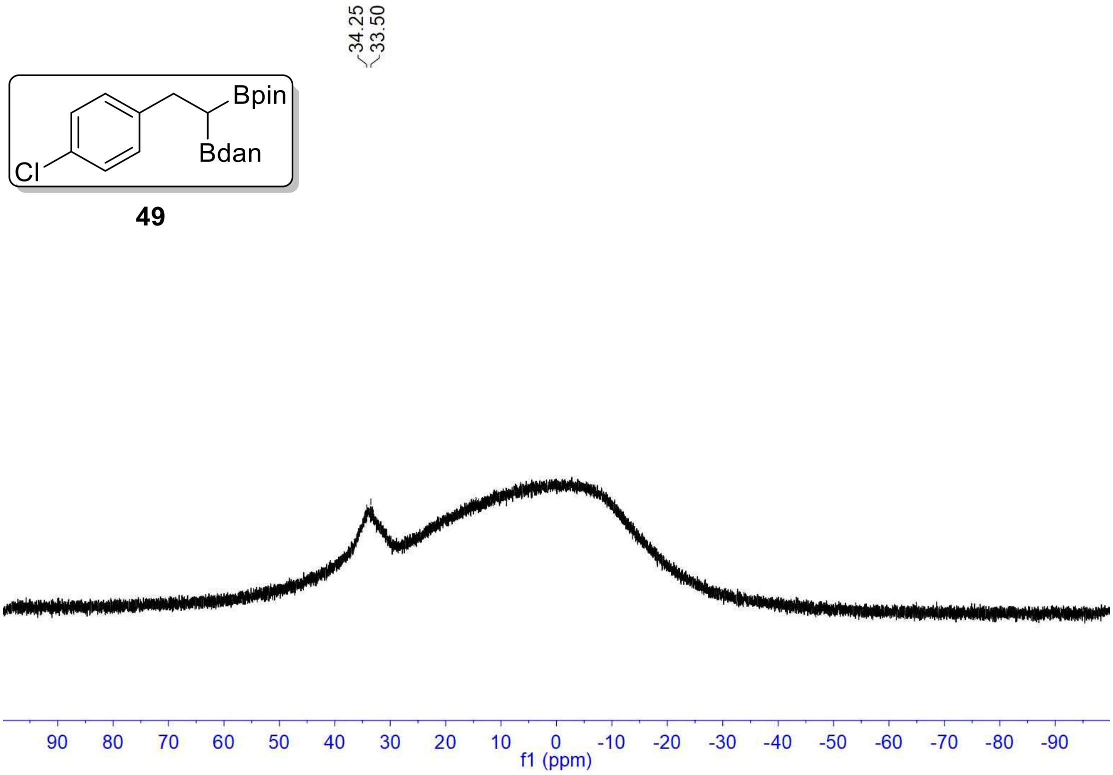

49

Supplementary Figure 158. ^{11}B NMR spectrum of compound 49

2-(1-(4,4,5,5-tetramethyl-1,3,2-dioxaborolan-2-yl)-2-(4-(trifluoromethyl)phenyl) ethyl)-2,3-dihydro-1H-naphtho[1,8-de][1,3,2]diazaborinine (50)

^{1}H NMR (500 MHz, room temperature, $CDCl_3$)

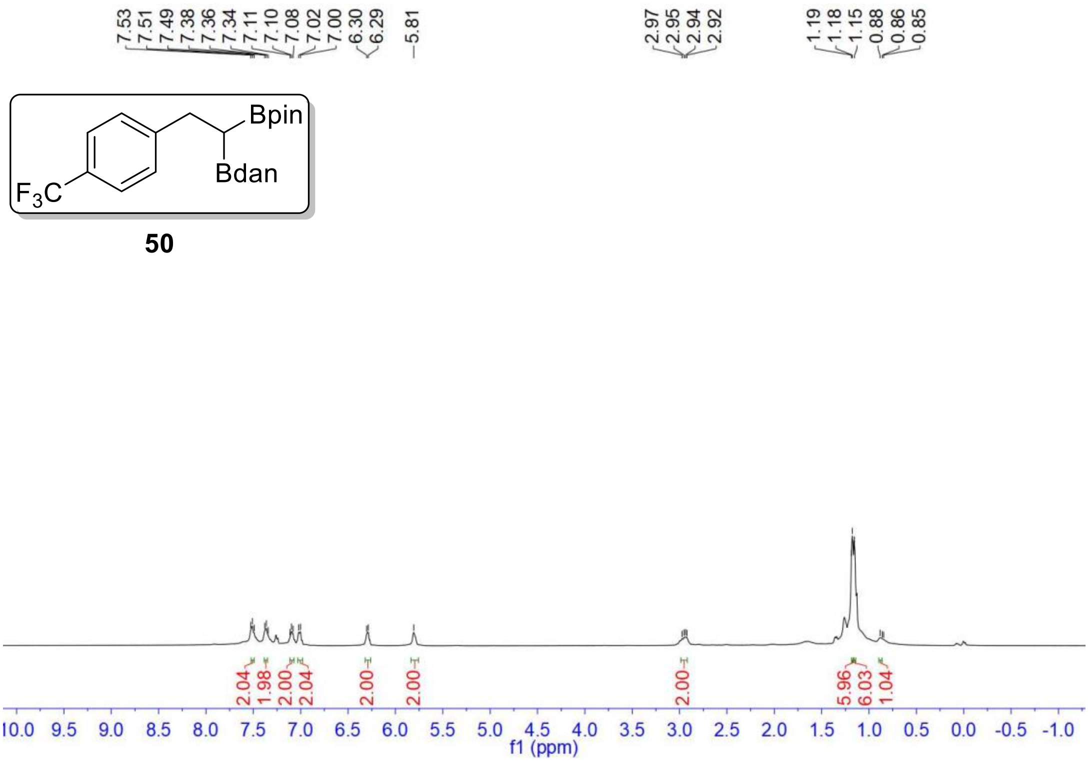

Supplementary Figure 159. ^{1}H NMR spectrum of compound 50

^{13}C NMR (126 MHz, room temperature, $CDCl_3$)

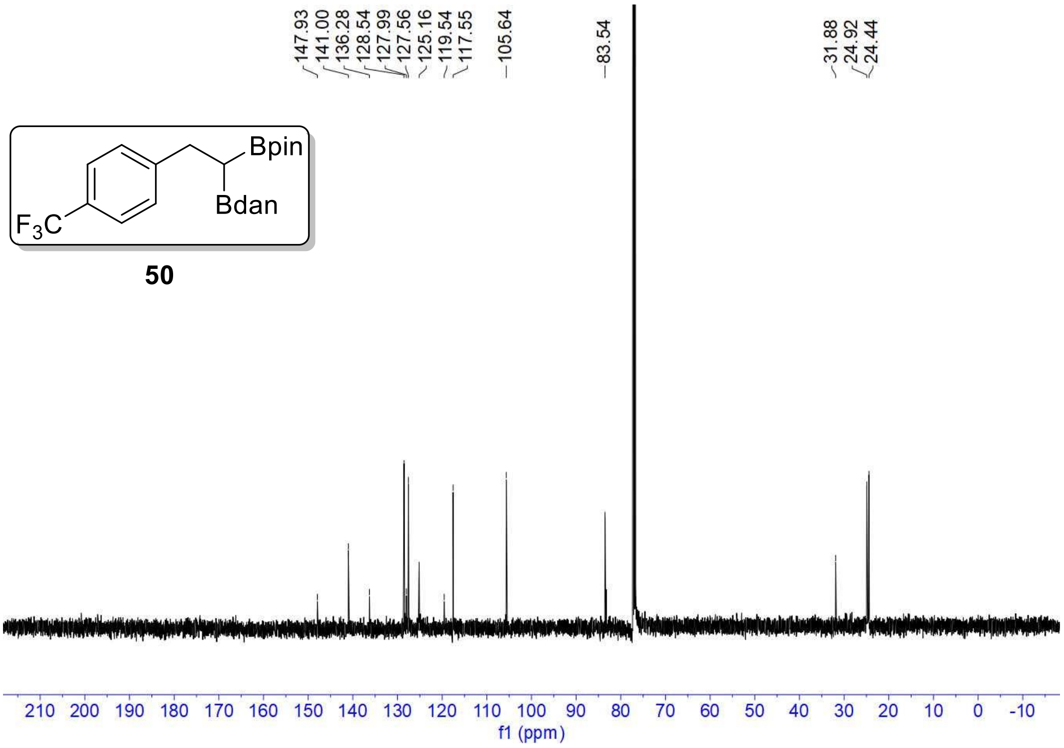

Supplementary Figure 160. ^{13}C NMR spectrum of compound 50

^{11}B NMR (160 MHz, room temperature, $CDCl_3$)

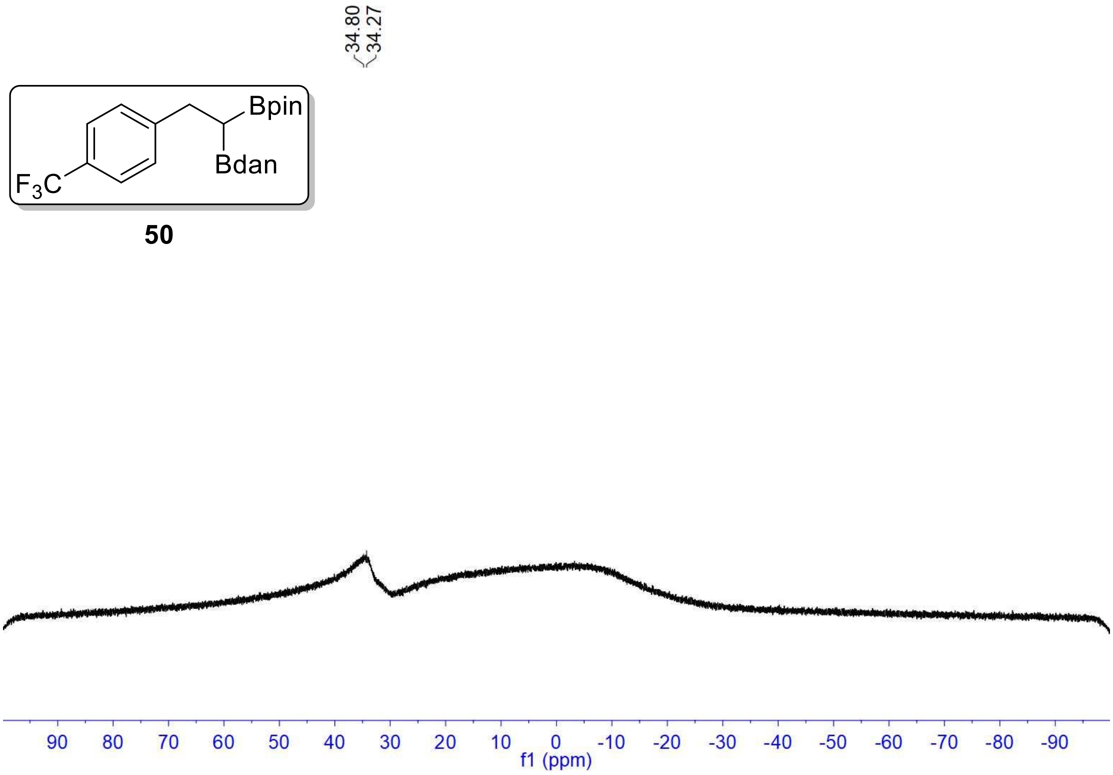

Supplementary Figure 161. ^{11}B **NMR spectrum of compound 50**

^{19}F NMR (126 MHz, room temperature, $CDCl_3$)

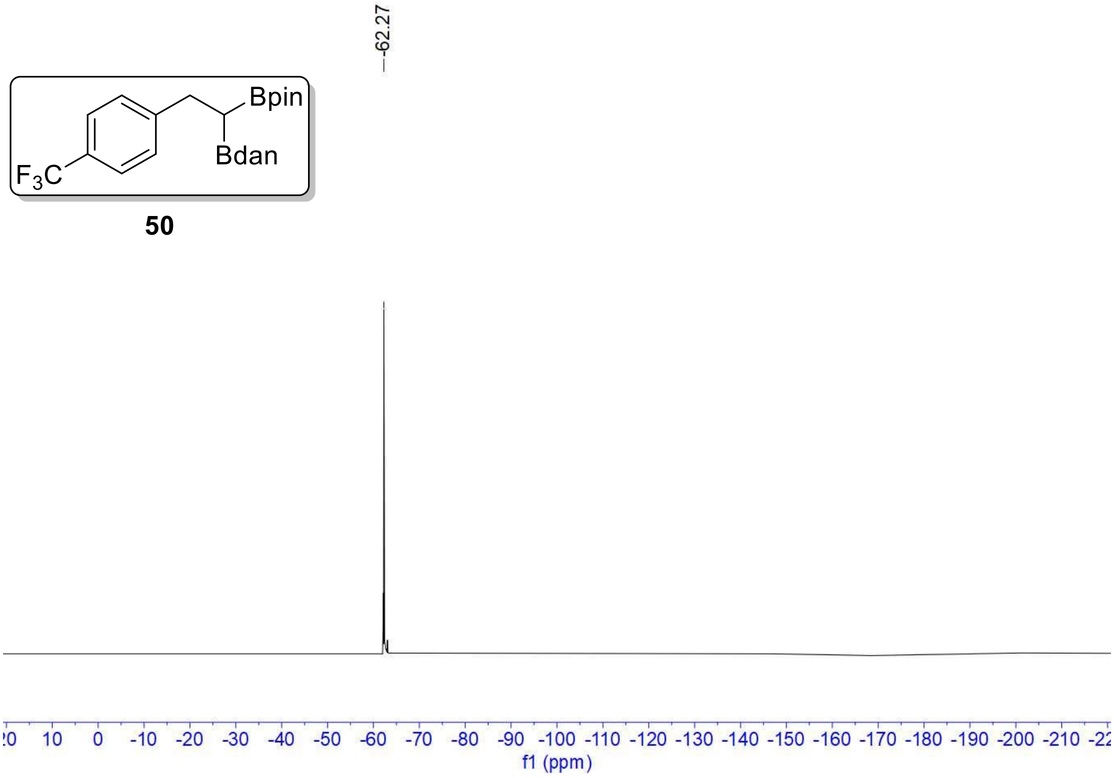

Supplementary Figure 162. ^{19}F **NMR spectrum of compound 50**

2-(1-(4,4,5,5-tetramethyl-1,3,2-dioxaborolan-2-yl)-2-(m-tolyl)ethyl)-2,3-dihydro-1H-naphtho[1,8-de][1,3,2]diazaborinine (51)

^{1}H NMR (500 MHz, room temperature, $CDCl_3$)

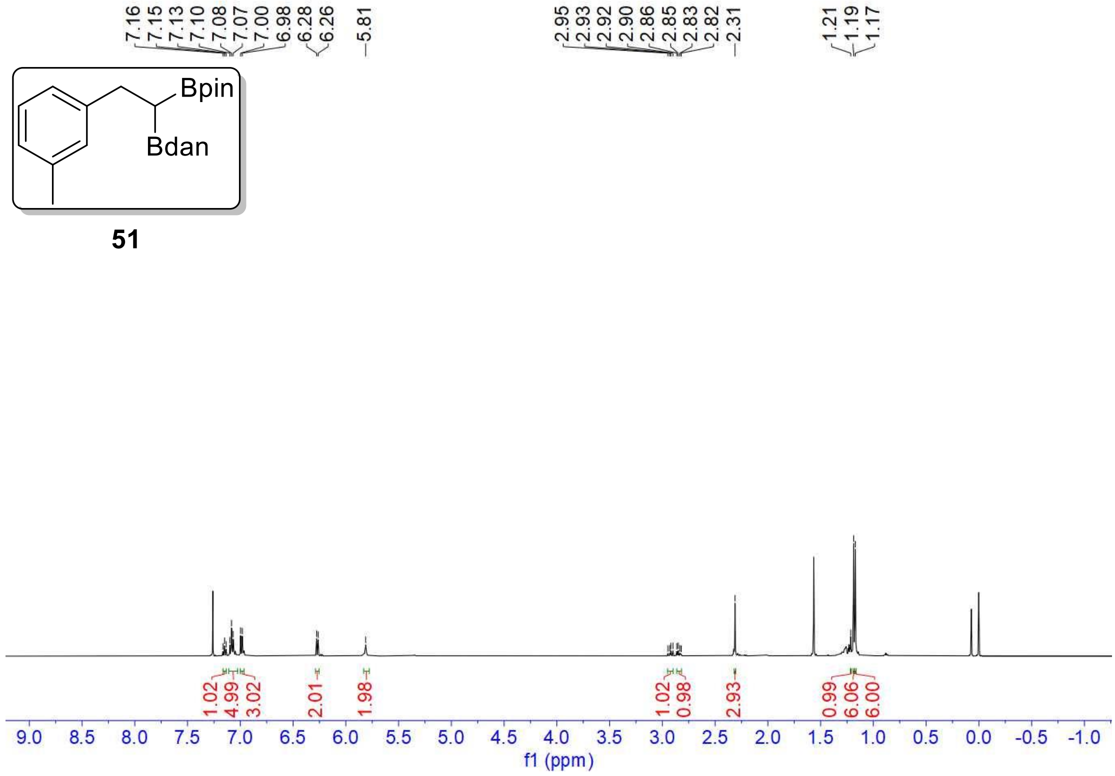

Supplementary Figure 163. ^{1}H NMR spectrum of compound 51

^{13}C NMR (126 MHz, room temperature, $CDCl_3$)

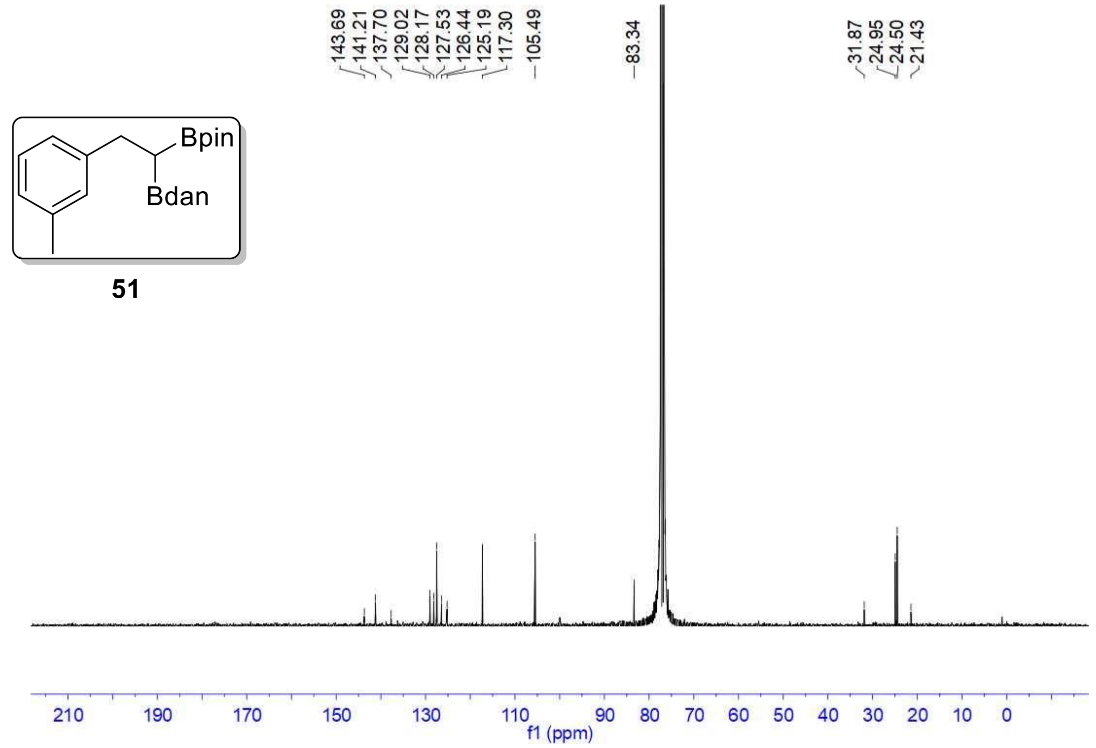

Supplementary Figure 164. ^{13}C NMR spectrum of compound 51

^{11}B NMR (160 MHz, room temperature, $CDCl_3$)

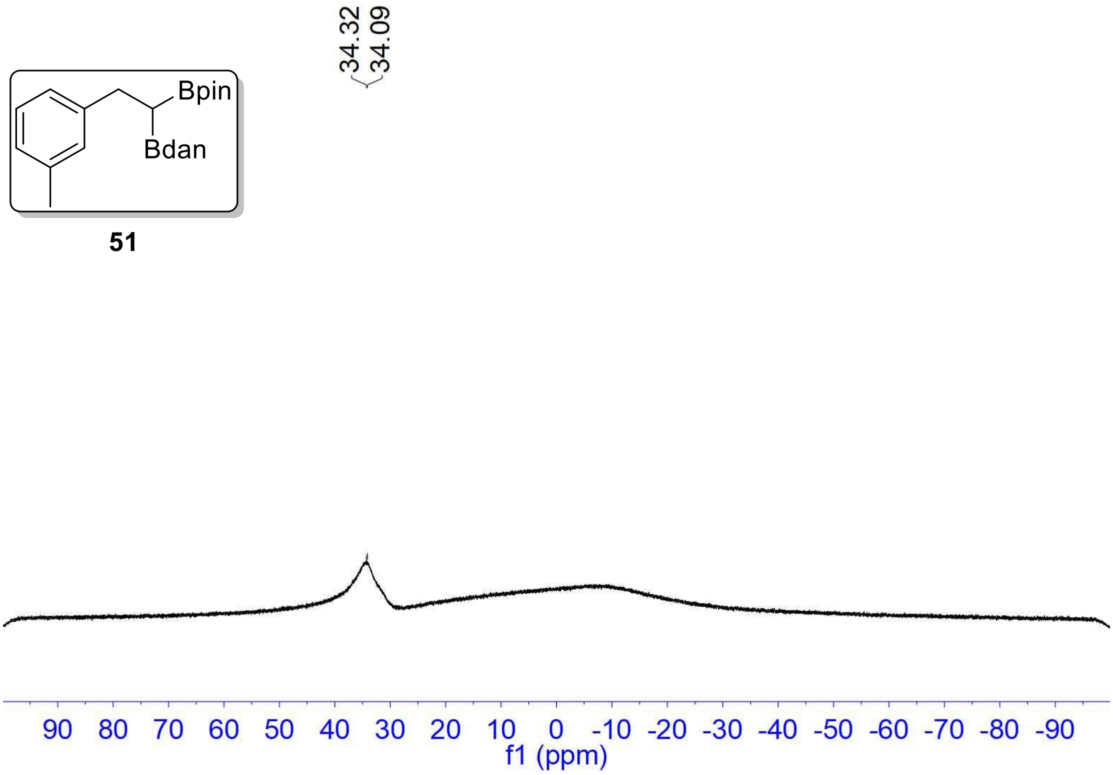

Supplementary Figure 165. ^{11}B NMR spectrum of compound 51

2-(2-(3,4-dimethylphenyl)-1-(4,4,5,5-tetramethyl-1,3,2-dioxaborolan-2-yl)ethyl)-2,3-dihydro-1H-naphtho[1,8-de][1,3,2]diazaborinine (52)

^{1}H NMR (500 MHz, room temperature, $CDCl_3$)

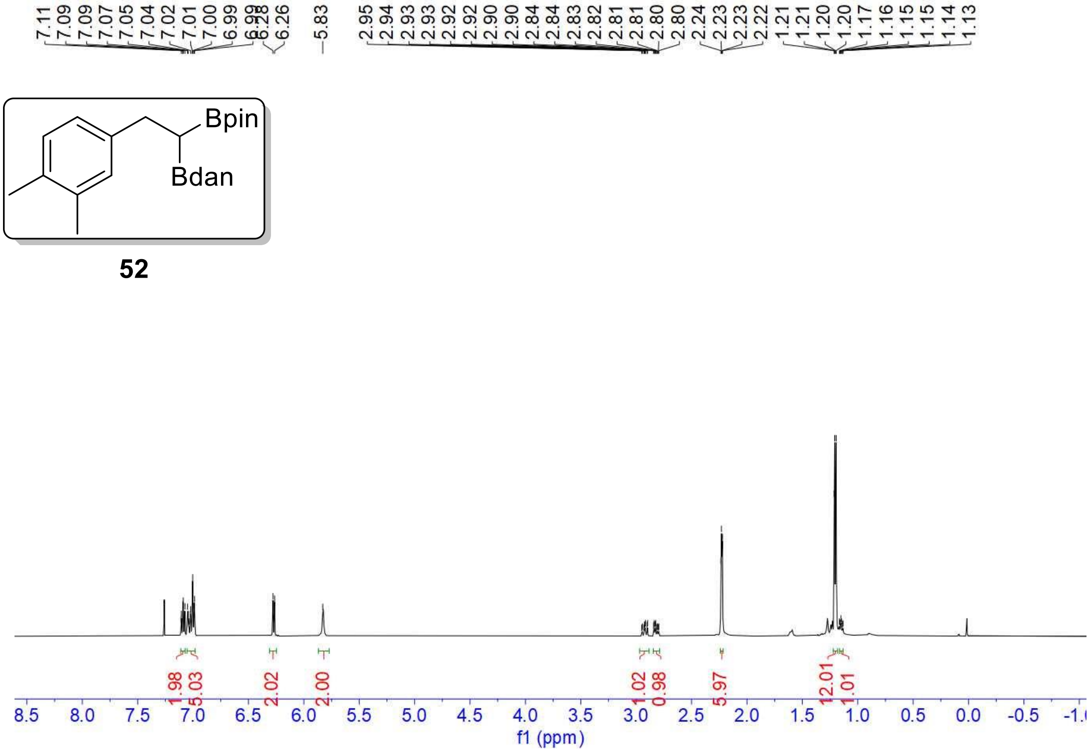

Supplementary Figure 166. ^{1}H NMR spectrum of compound 52

^{13}C NMR (126 MHz, room temperature, $CDCl_3$)

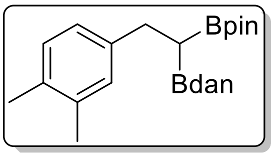

52

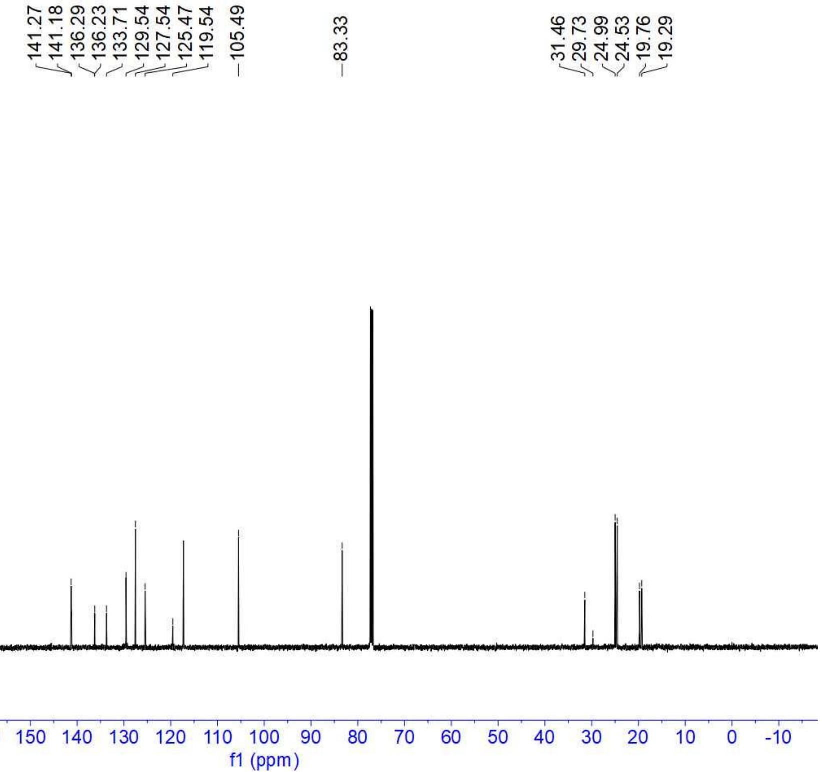

Supplementary Figure 167. ^{13}**C NMR spectrum of compound 52**

^{11}B NMR (160 MHz, room temperature, $CDCl_3$)

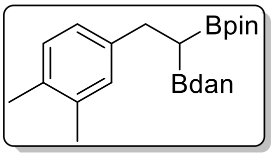

52

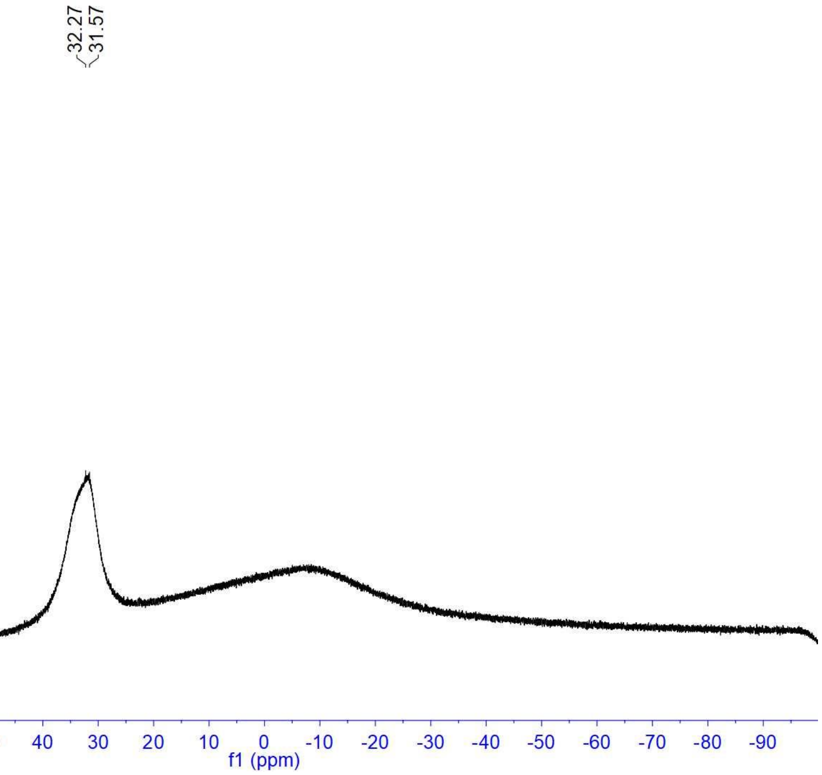

Supplementary Figure 168. ^{11}**B NMR spectrum of compound 52**

2-(2-(3,5-dimethylphenyl)-1-(4,4,5,5-tetramethyl-1,3,2-dioxaborolan-2-yl)ethyl)-2,3-dihydro-1H-naphtho[1,8-de][1,3,2]diazaborinine (53)

^{1}H NMR (500 MHz, room temperature, $CDCl_3$)

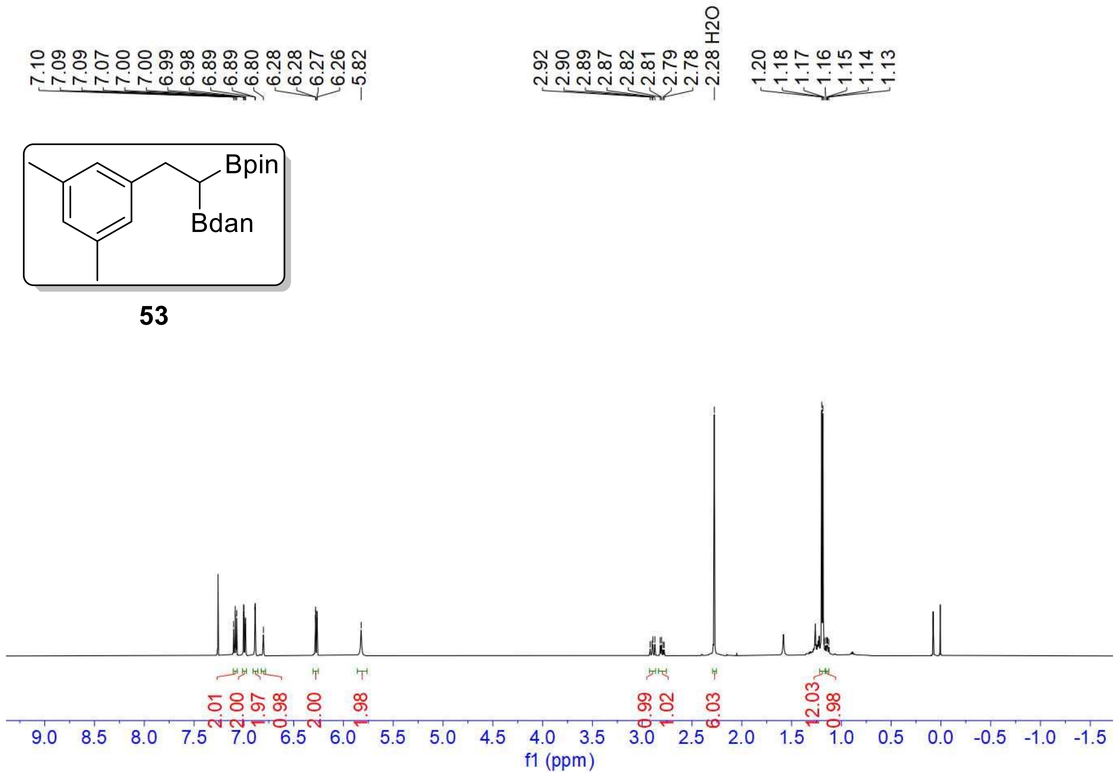

Supplementary Figure 169. ^{1}H NMR spectrum of compound 53

^{13}C NMR (126 MHz, room temperature, $CDCl_3$)

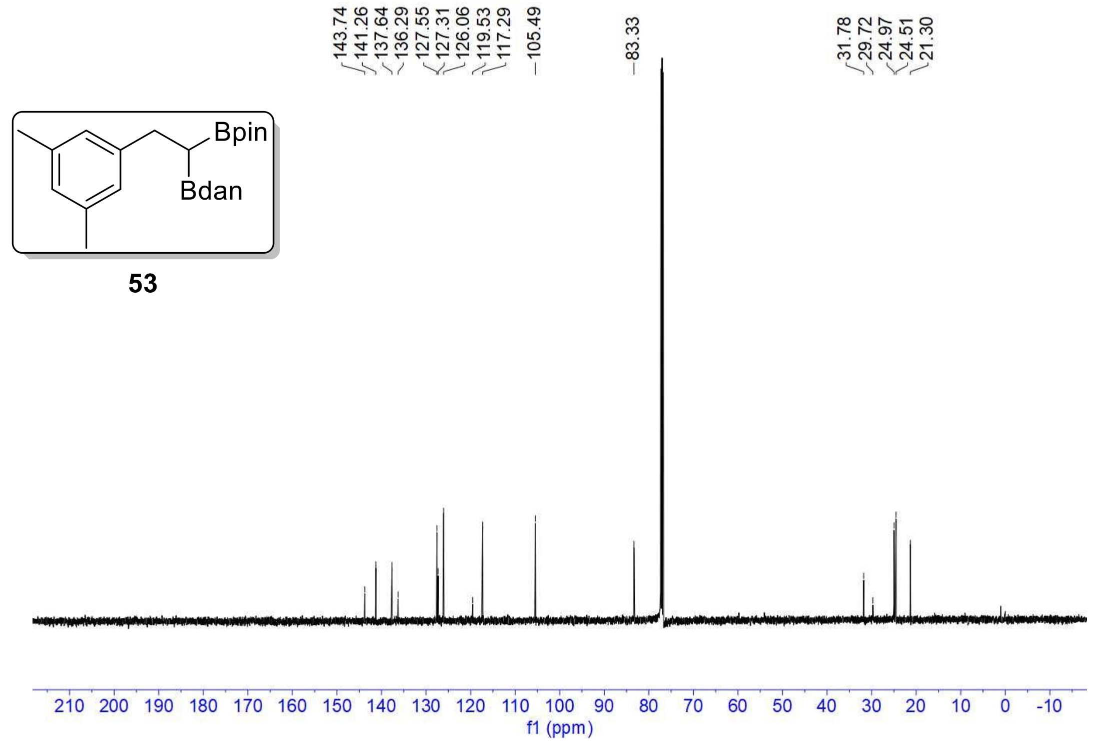

Supplementary Figure 170. ^{13}C NMR spectrum of compound 53

^{11}B NMR (160 MHz, room temperature, $CDCl_3$)

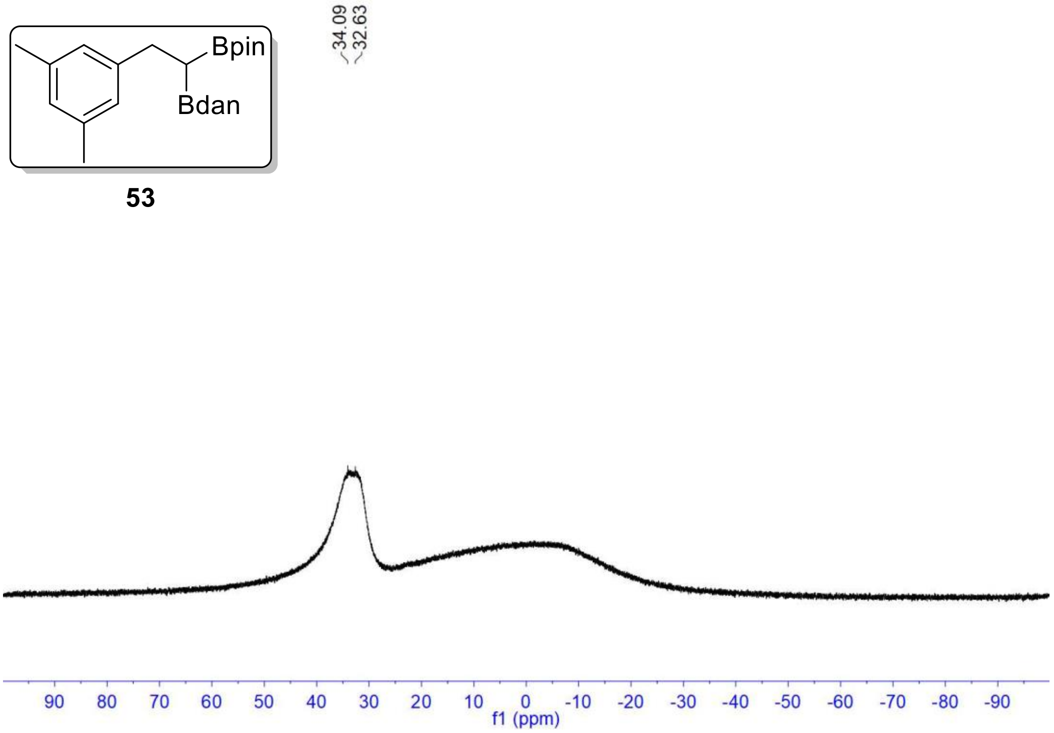

Supplementary Figure 171. ^{11}B NMR spectrum of compound 53

2-(2-(4,4-dimethylthiochroman-7-yl)-1-(4,4,5,5-tetramethyl-1,3,2-dioxaborolan-2-yl)ethyl)-2,3-dihydro-1H-naphtho[1,8-de][1,3,2]diazaborinine (54)

^{1}H NMR (500 MHz, room temperature, $CDCl_3$)

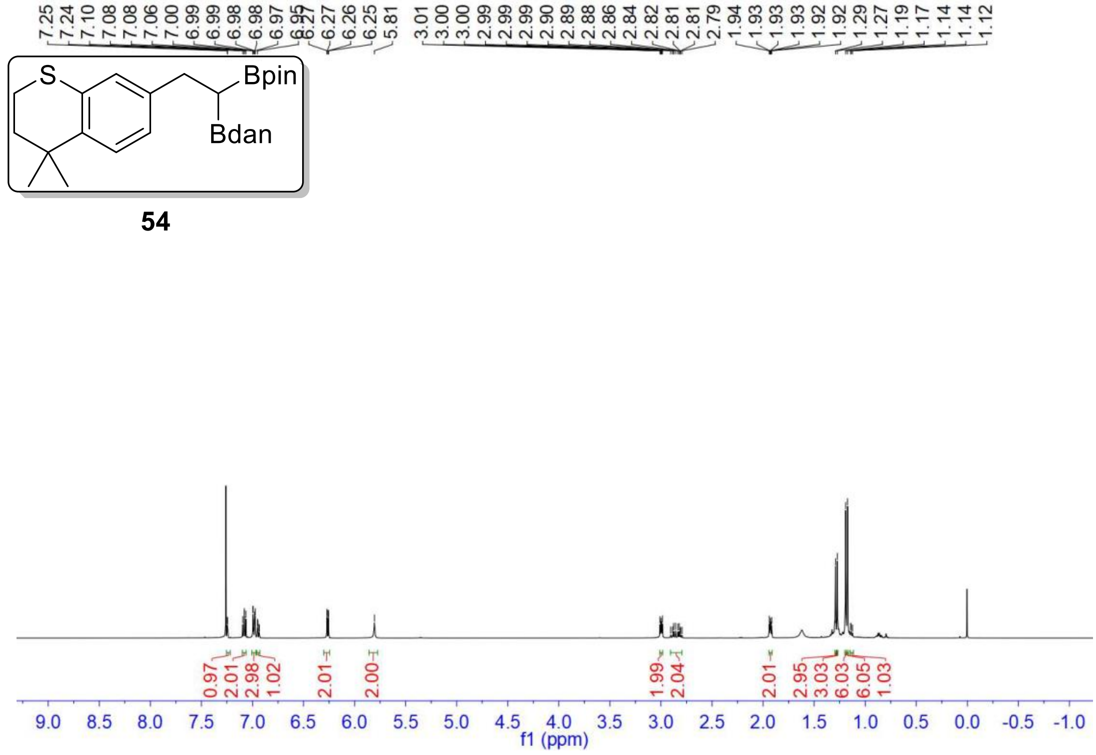

Supplementary Figure 172. ^{1}H NMR spectrum of compound 54

^{13}C NMR (126 MHz, room temperature, $CDCl_3$)

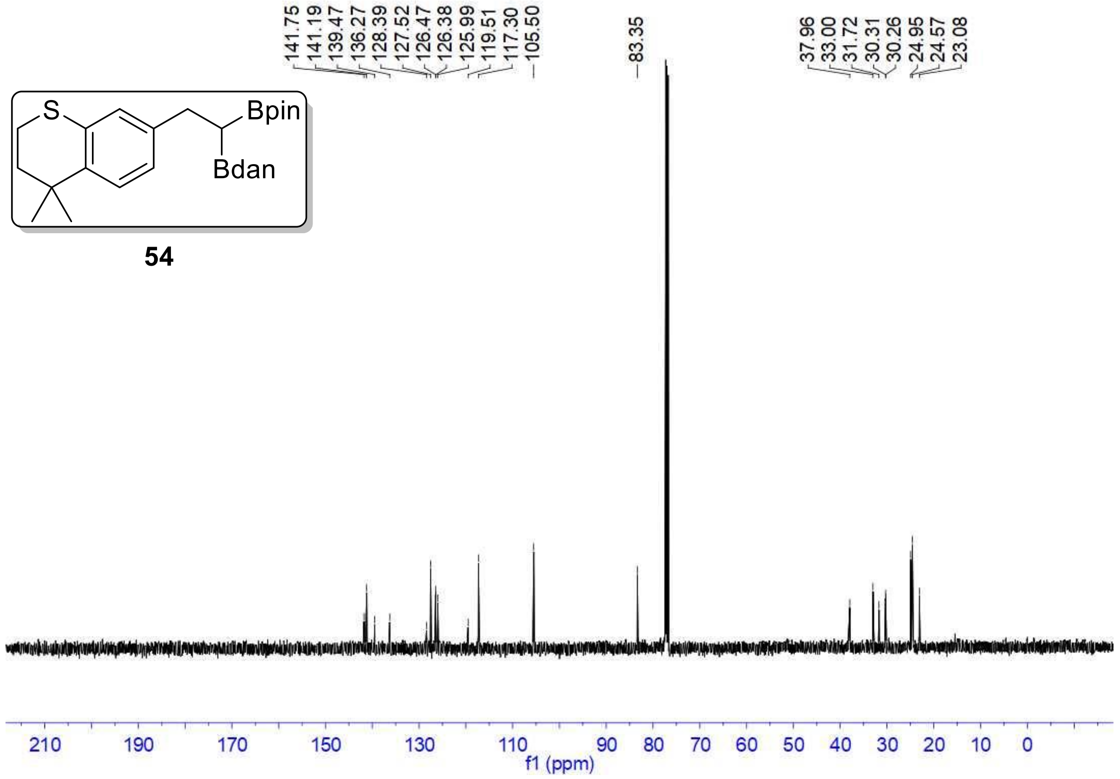

Supplementary Figure 173. ^{13}C NMR spectrum of compound 54

^{11}B NMR (160 MHz, room temperature, $CDCl_3$)

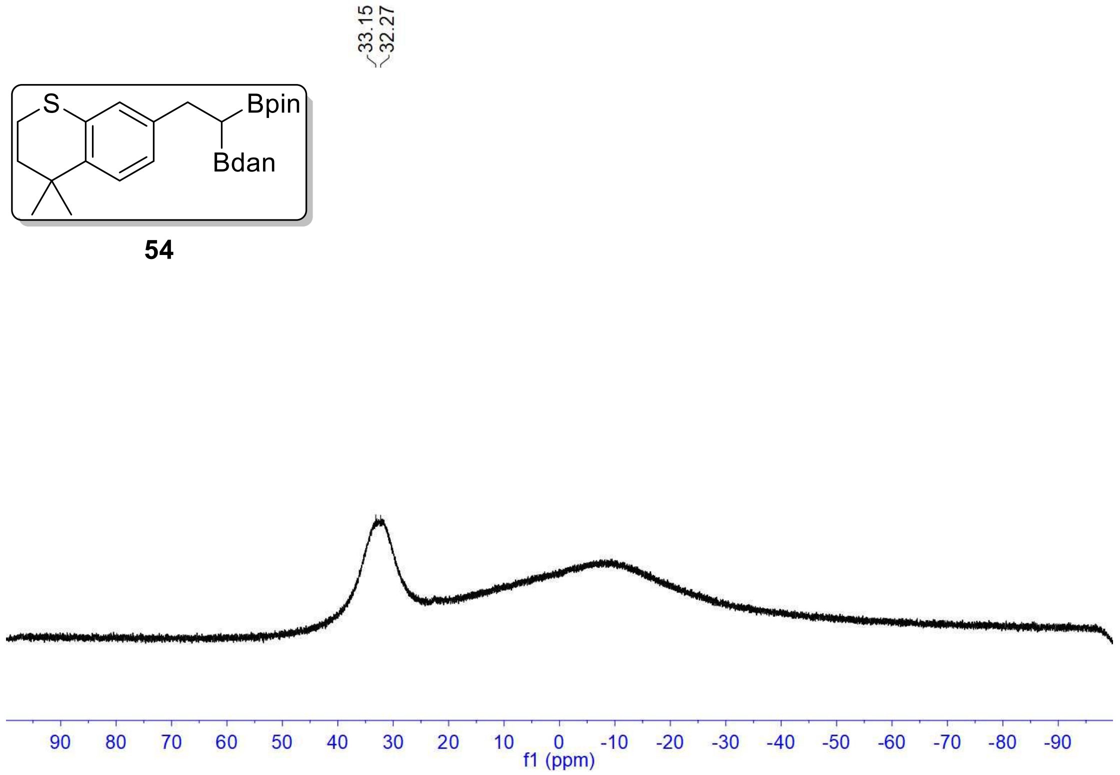

Supplementary Figure 174. ^{11}B NMR spectrum of compound 54

2-(1-(4,4,5,5-tetramethyl-1,3,2-dioxaborolan-2-yl)-2-(thiophen-3-yl)ethyl)-2,3-dihydro-1H-naphtho[1,8-de][1,3,2]diazaborinine (55)

^{1}H NMR (500 MHz, room temperature, CDCl$_3$)

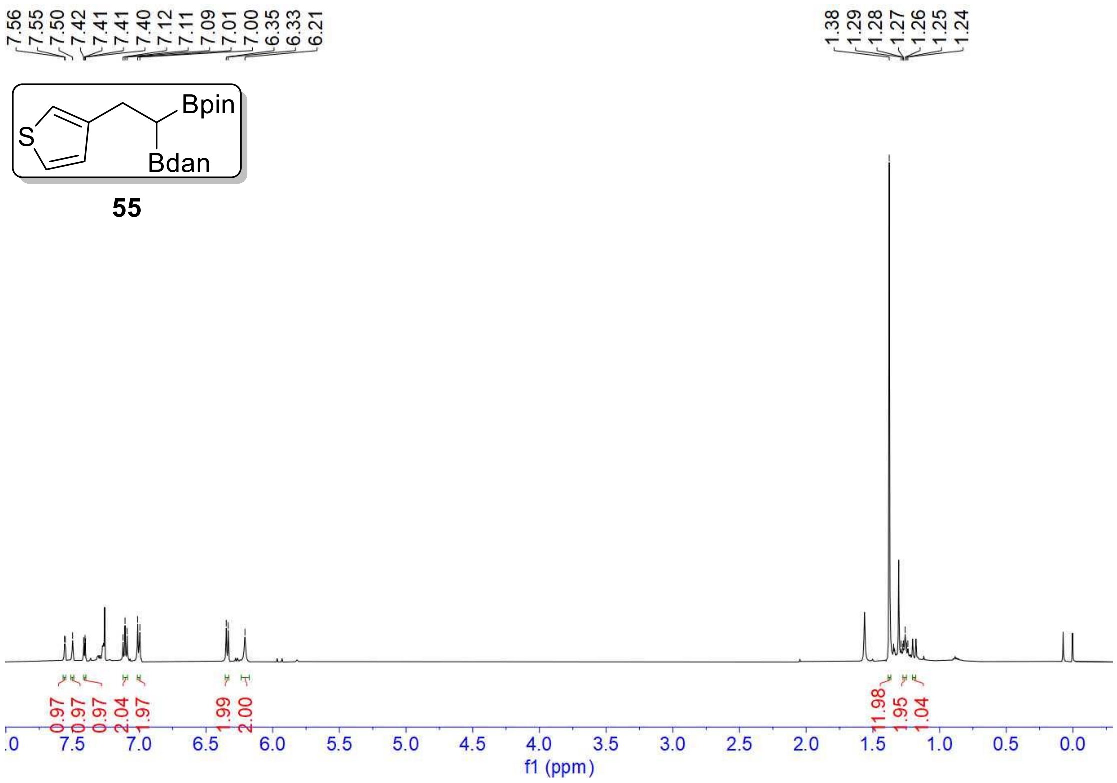

Supplementary Figure 175. ^{1}H NMR spectrum of compound 55

^{13}C NMR (126 MHz, room temperature, CDCl$_3$)

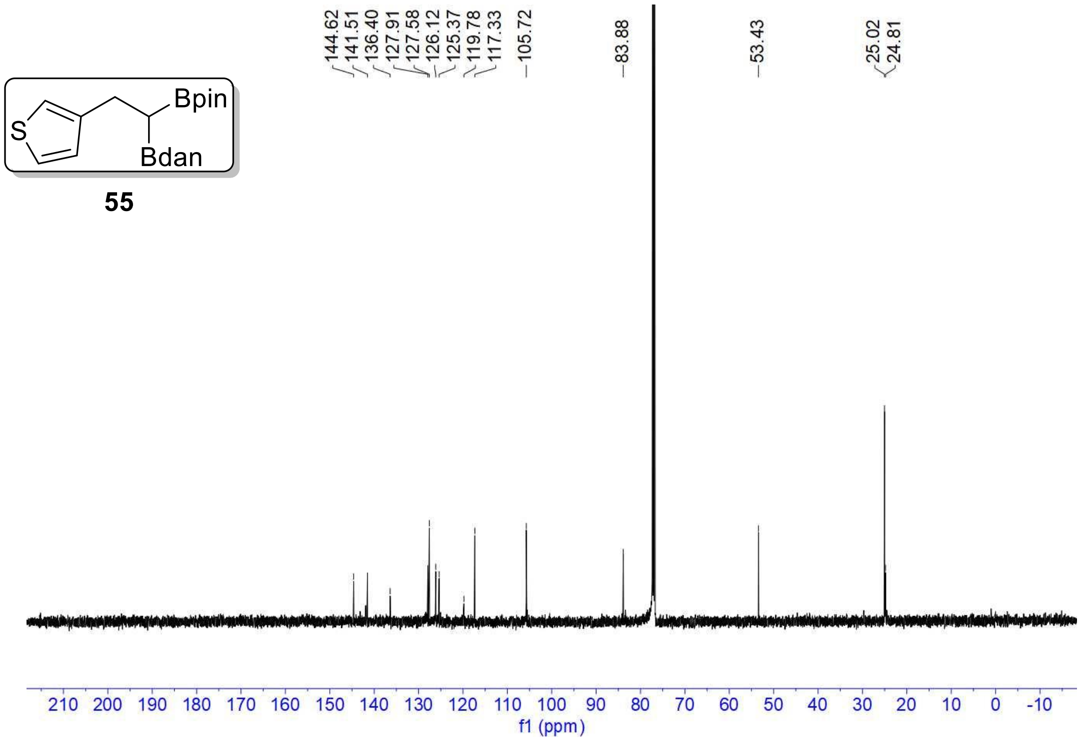

Supplementary Figure 176. ^{13}C NMR spectrum of compound 55

^{11}B NMR (160 MHz, room temperature, CDCl$_3$)

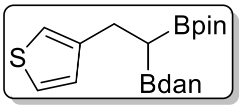

55

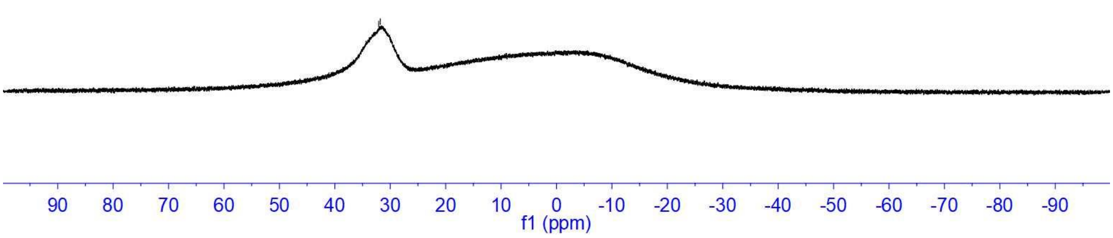

Supplementary Figure 177. ^{11}B NMR spectrum of compound 55

2-(2-(1H-phenalen-5-yl)-1-(4,4,5,5-tetramethyl-1,3,2-dioxaborolan-2-yl)ethyl)-2,3-dihydro-1H-naphtho[1,8-de][1,3,2]diazaborinine (56)

^{1}H NMR (500 MHz, room temperature, CDCl$_3$)

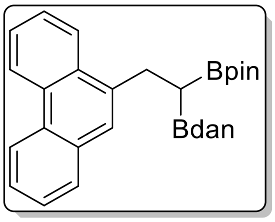

56

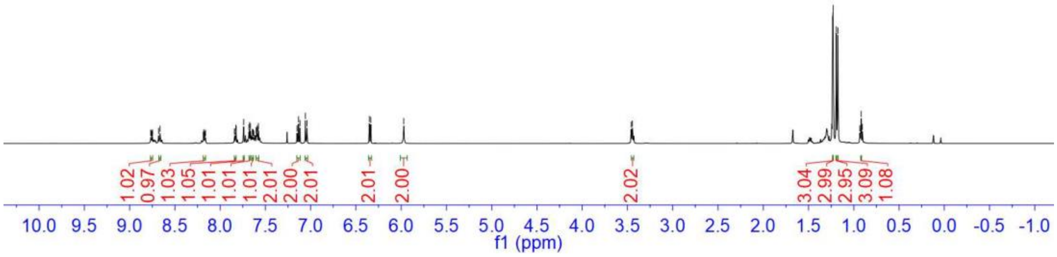

Supplementary Figure 178. ^{1}H NMR spectrum of compound 56

^{13}C NMR (126 MHz, room temperature, $CDCl_3$)

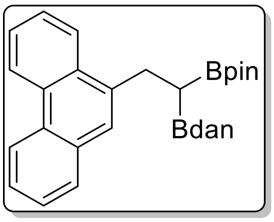

56

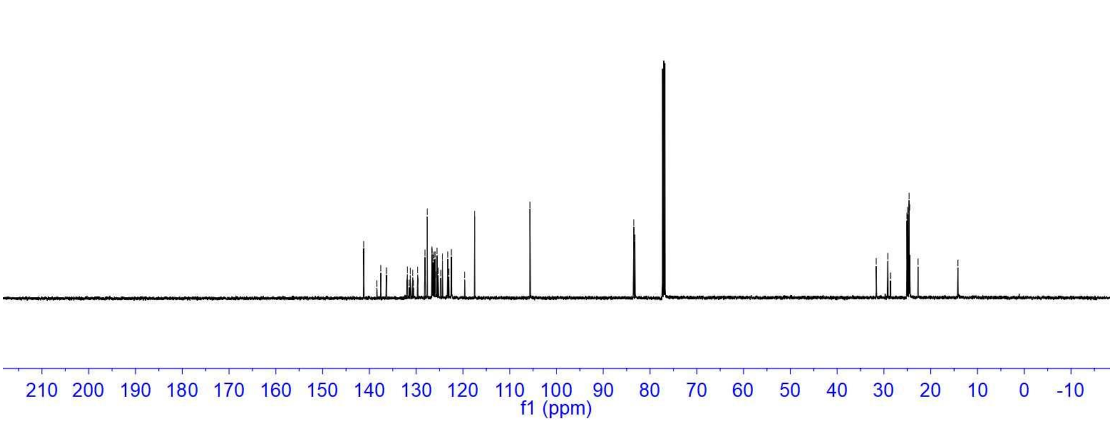

Supplementary Figure 179. ^{13}C NMR spectrum of compound 56

^{11}B NMR (160 MHz, room temperature, $CDCl_3$)

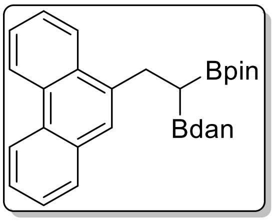

56

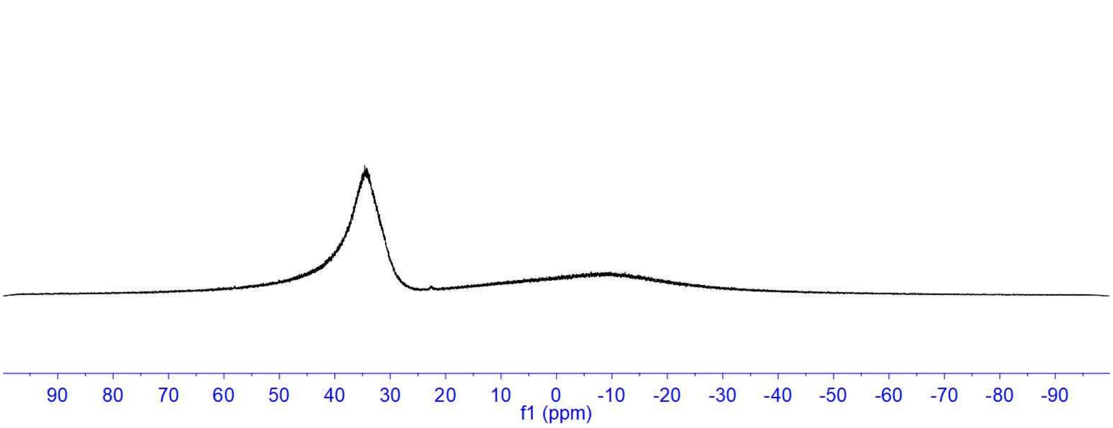

Supplementary Figure 180. ^{11}B NMR spectrum of compound 56

2-(2-(4-(9H-carbazol-9-yl)phenyl)-1-(4,4,5,5-tetramethyl-1,3,2-dioxaborolan-2-yl)ethyl)-2,3-dihydro-1H-naphtho[1,8-de][1,3,2]diazaborinine (57)

^{1}H NMR (500 MHz, room temperature, $CDCl_3$)

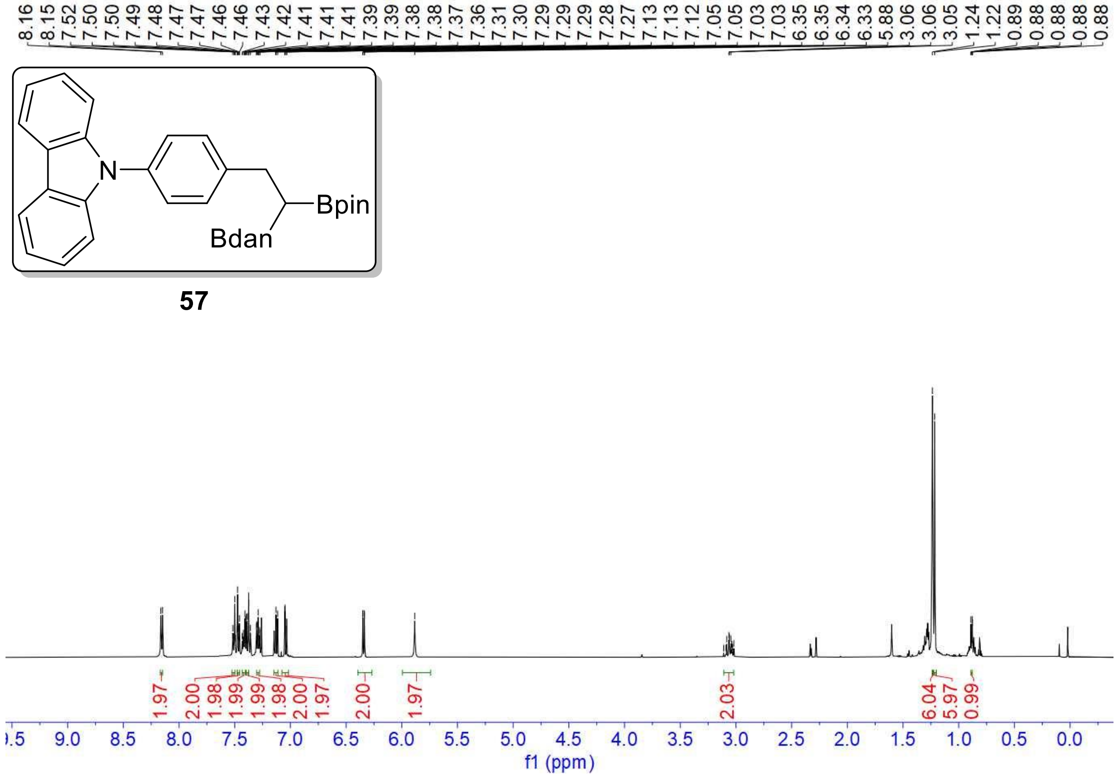

Supplementary Figure 181. ^{1}H NMR spectrum of compound 57

^{13}C NMR (126 MHz, room temperature, $CDCl_3$)

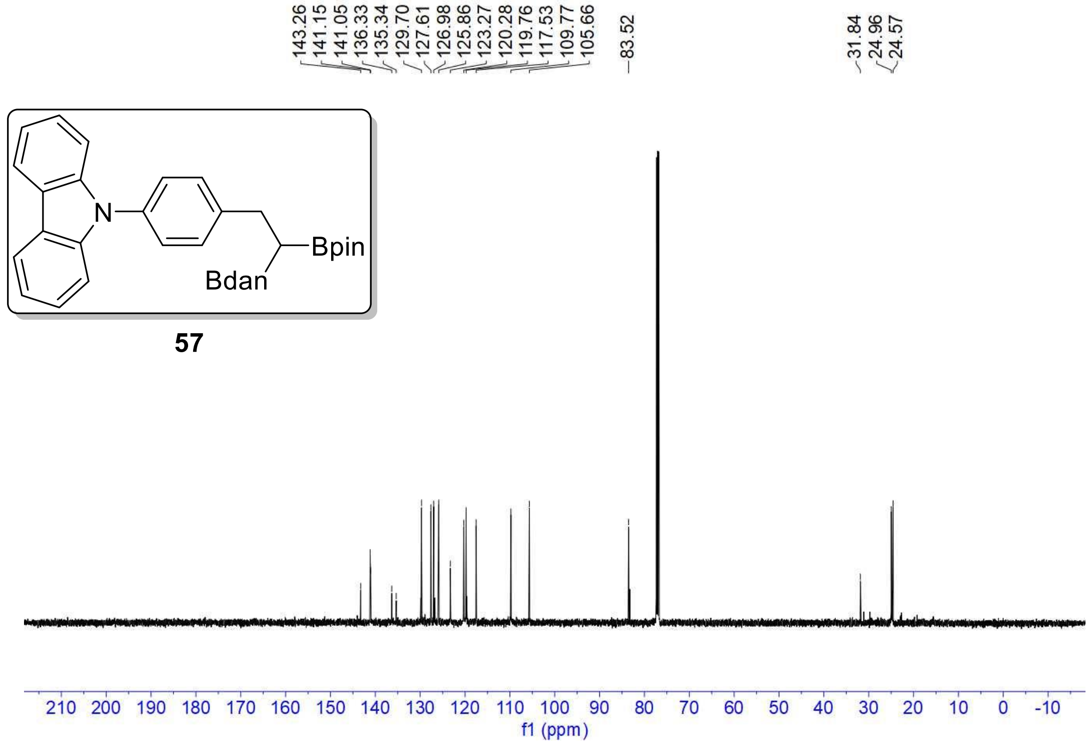

Supplementary Figure 182. ^{13}C NMR spectrum of compound 57

^{11}B NMR (160 MHz, room temperature, $CDCl_3$)

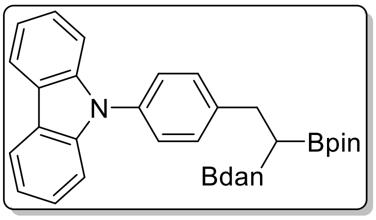

57

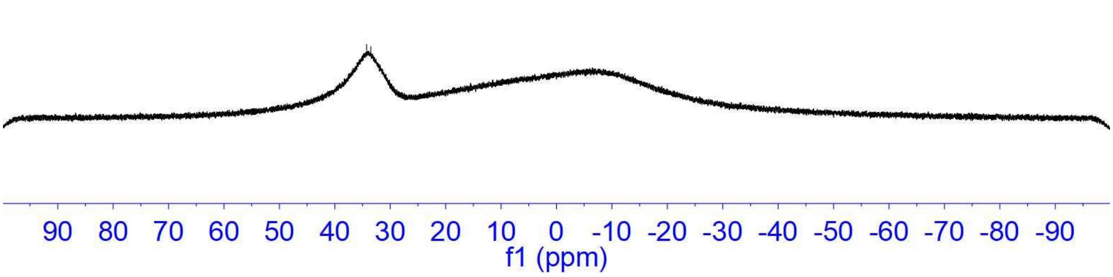

Supplementary Figure 183. ^{11}B NMR spectrum of compound 57

2-(2-(naphthalen-2-yl)-1-(4,4,5,5-tetramethyl-1,3,2-dioxaborolan-2-yl)ethyl)-2,3-dihydro-1H-naphtho[1,8-de][1,3,2]diazaborinine (58)

^{1}H NMR (500 MHz, room temperature, $CDCl_3$)

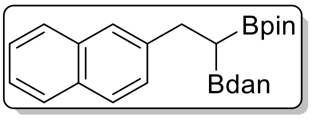

58

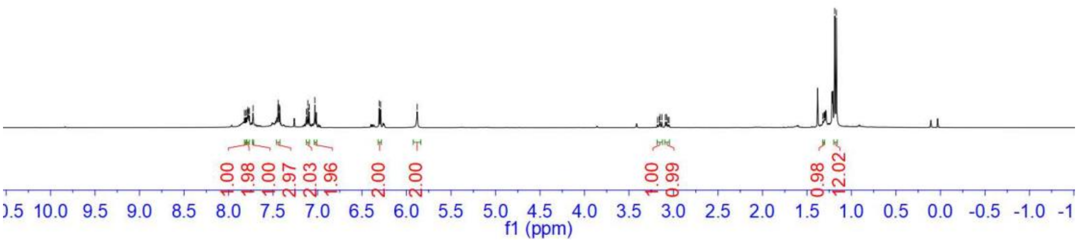

Supplementary Figure 184. ^{1}H NMR spectrum of compound 58

^{13}C NMR (126 MHz, room temperature, $CDCl_3$)

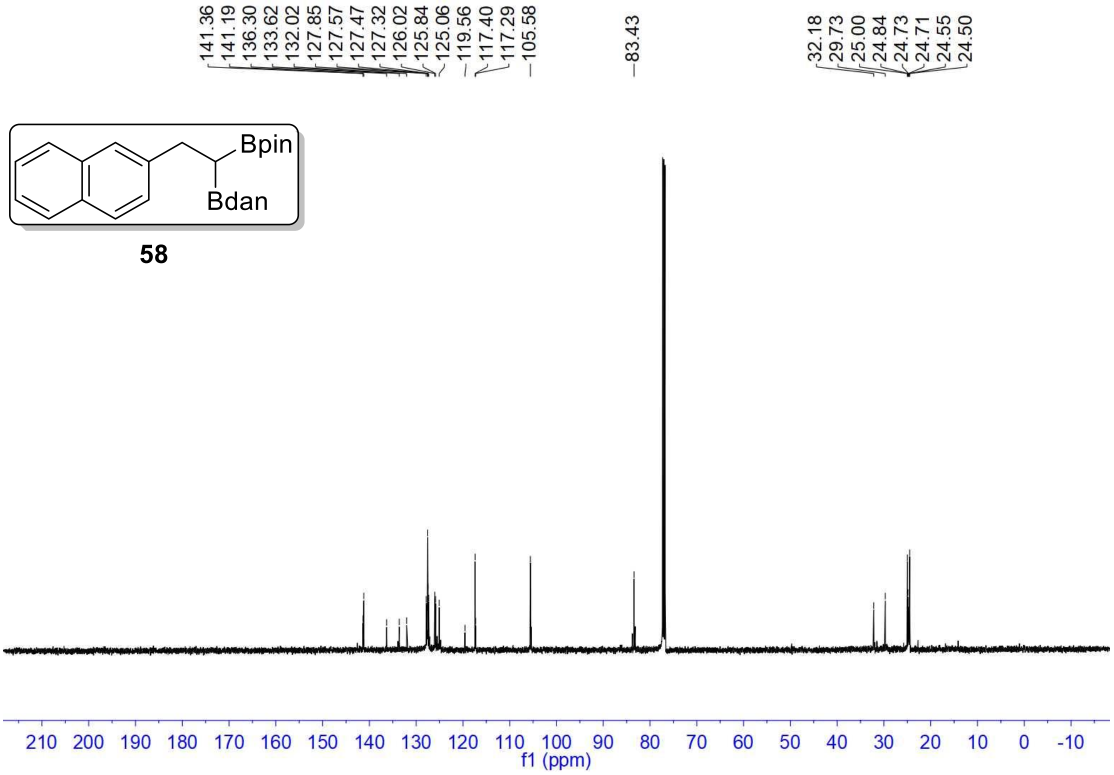

Supplementary Figure 185. ^{13}C NMR spectrum of compound 58

^{11}B NMR (160 MHz, room temperature, $CDCl_3$)

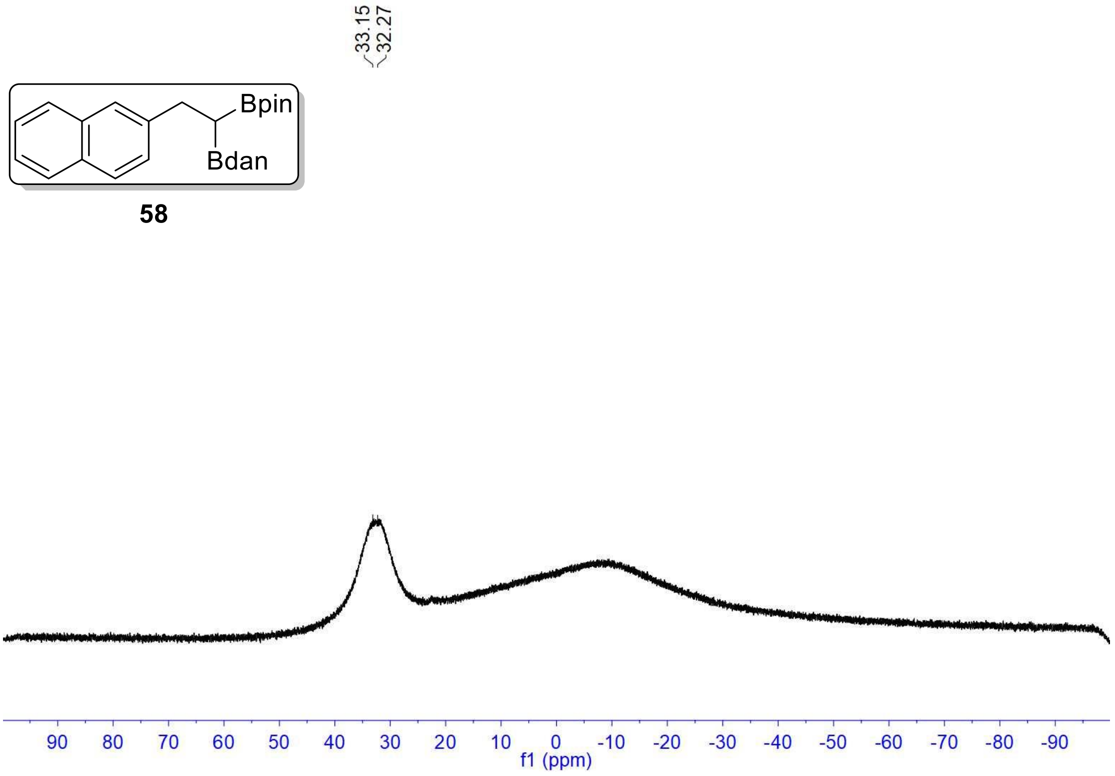

Supplementary Figure 186. ^{11}B NMR spectrum of compound 58

(R)-2-(4-phenyl-1-(4,4,5,5-tetramethyl-1,3,2-dioxaborolan-2-yl)butyl)-2,3-dihydro-1H-naphtho[1,8-de][1,3,2]diazaborinine (59)

^{1}H NMR (500 MHz, room temperature, CDCl$_3$)

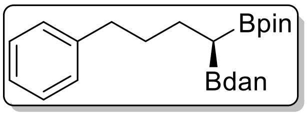

59

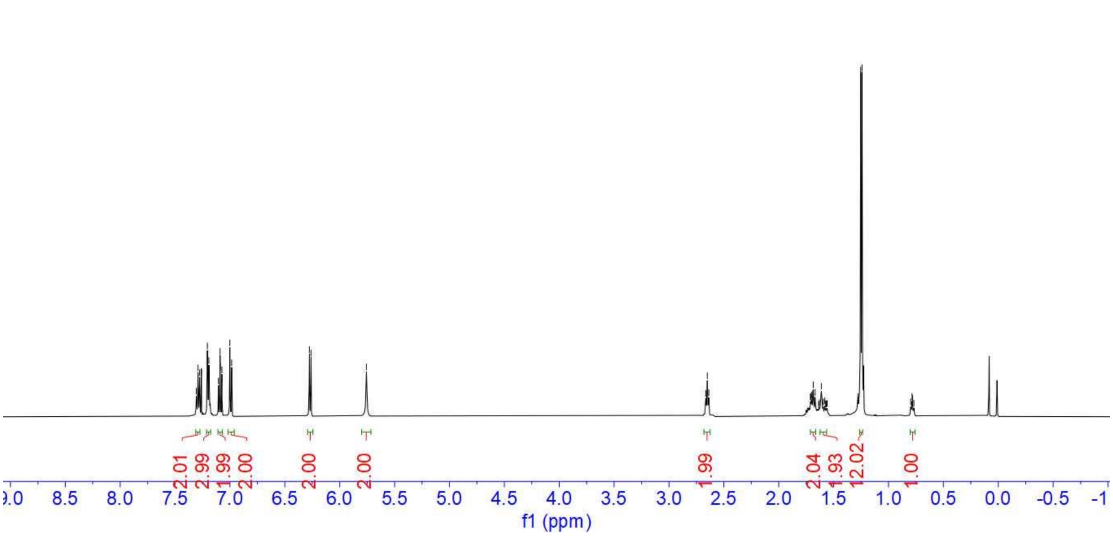

Supplementary Figure 187. ^{1}H NMR spectrum of compound 59

^{13}C NMR (126 MHz, room temperature, CDCl$_3$)

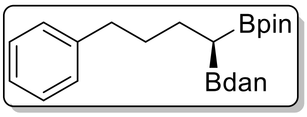

59

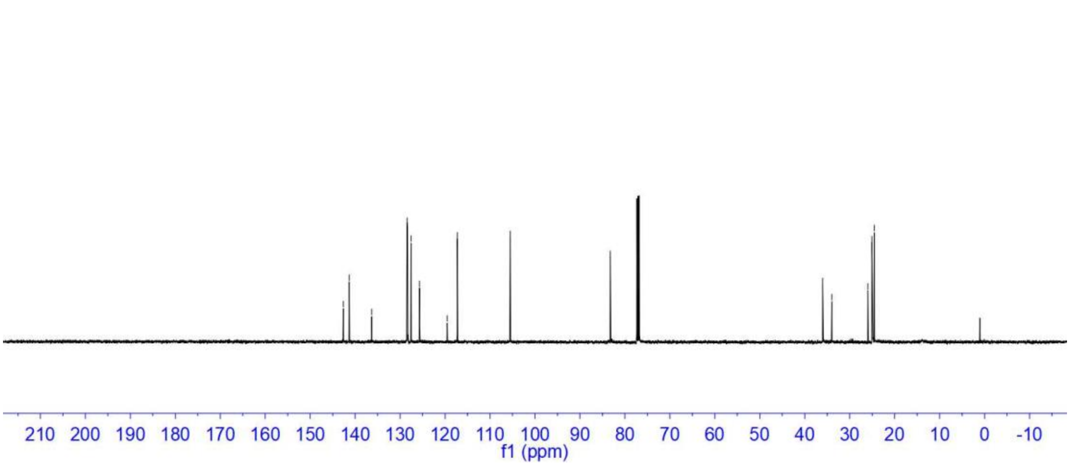

Supplementary Figure 188. ^{13}C NMR spectrum of compound 59

^{11}B NMR (160 MHz, room temperature, $CDCl_3$)

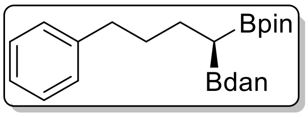

59

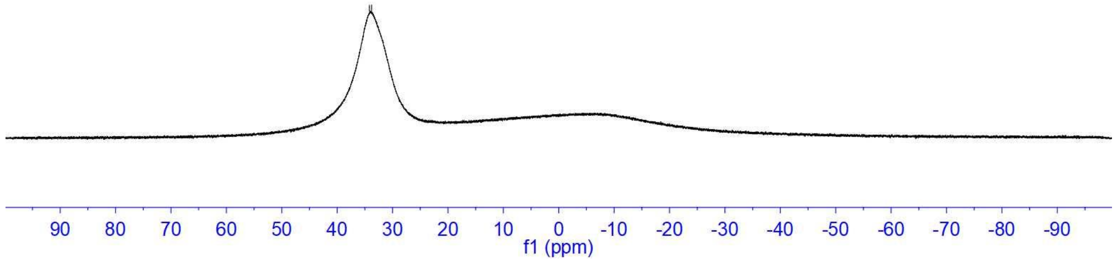

Supplementary Figure 189. [11]**B NMR spectrum of compound 59**

(R)-2-(3-phenyl-1-(4,4,5,5-tetramethyl-1,3,2-dioxaborolan-2-yl)propyl)-2,3-dihydro-1H-naphtho[1,8-de][1,3,2]diazaborinine (60)

^{1}H NMR (500 MHz, room temperature, $CDCl_3$)

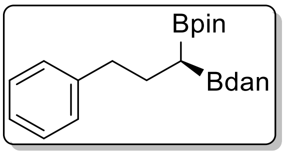

60

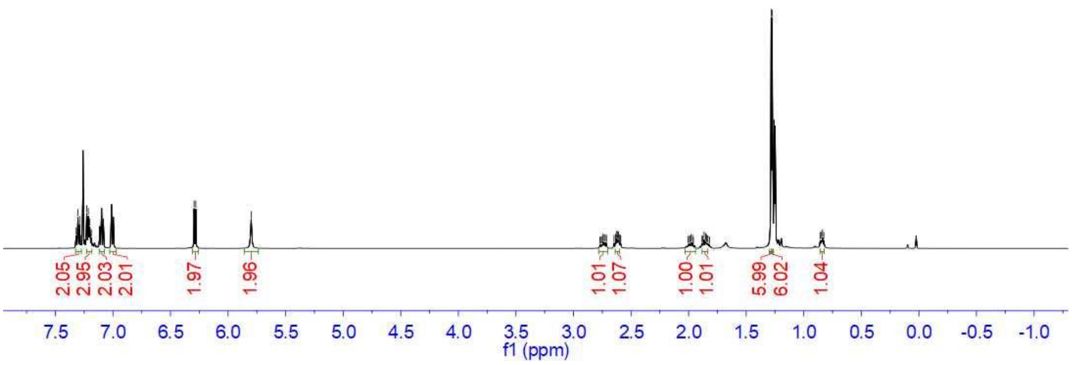

Supplementary Figure 190. [1]**H NMR spectrum of compound 60**

^{13}C NMR (126 MHz, room temperature, $CDCl_3$)

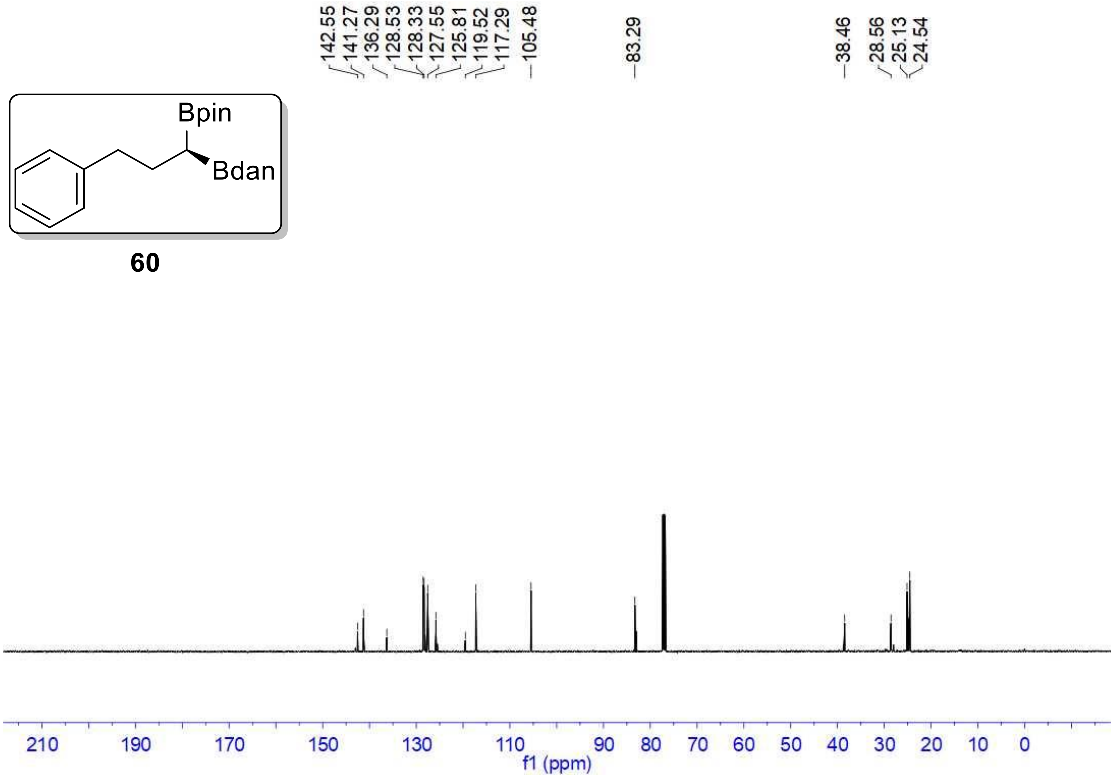

Supplementary Figure 191. ^{13}C **NMR spectrum of compound 60**

^{11}B NMR (160 MHz, room temperature, $CDCl_3$)

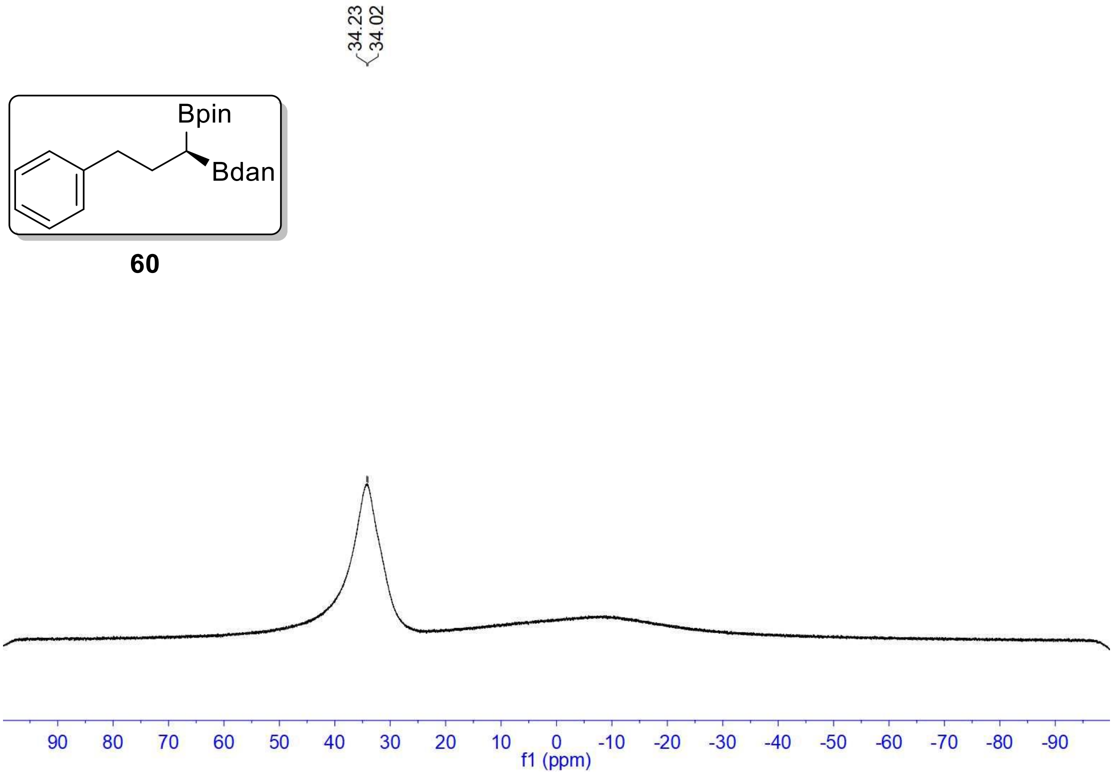

Supplementary Figure 192. ^{11}B **NMR spectrum of compound 60**

(R)-2-(2-cyclohexyl-1-(4,4,5,5-tetramethyl-1,3,2-dioxaborolan-2-yl)ethyl)-2,3-dihydro-1H-naphtho[1,8-de][1,3,2]diazaborinine (61)

^{1}H NMR (500 MHz, room temperature, CDCl$_3$)

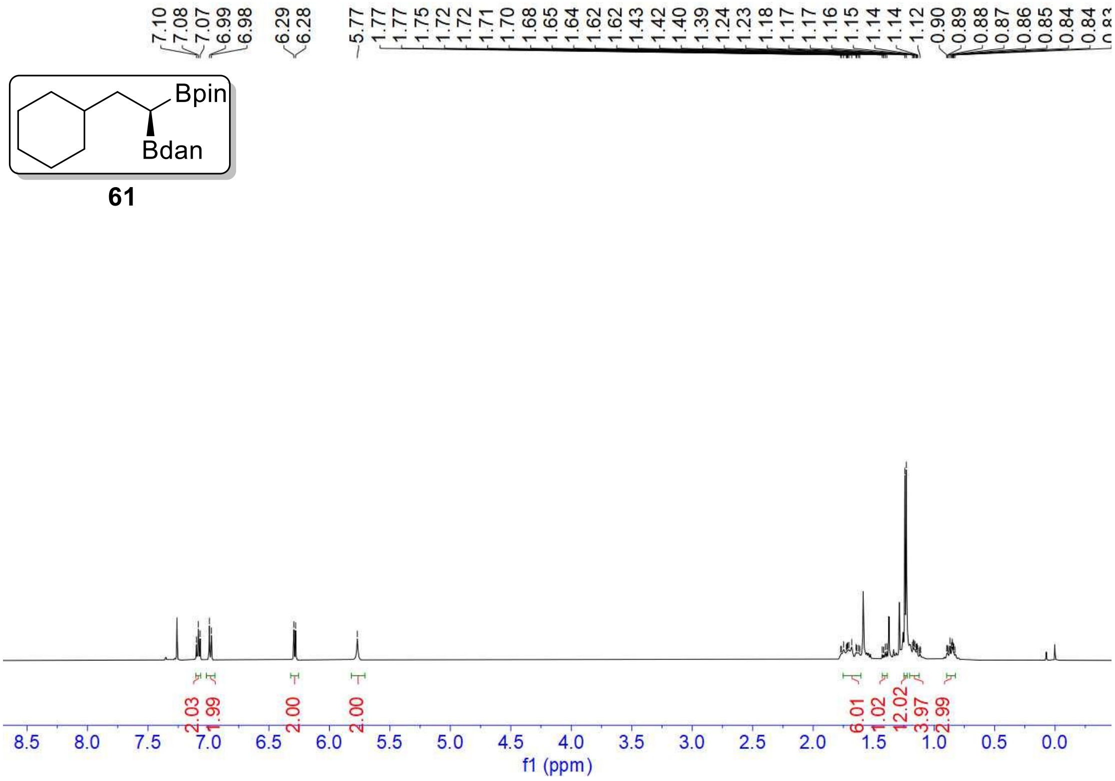

Supplementary Figure 193. ^{1}H NMR spectrum of compound 61

^{13}C NMR (126 MHz, room temperature, CDCl$_3$)

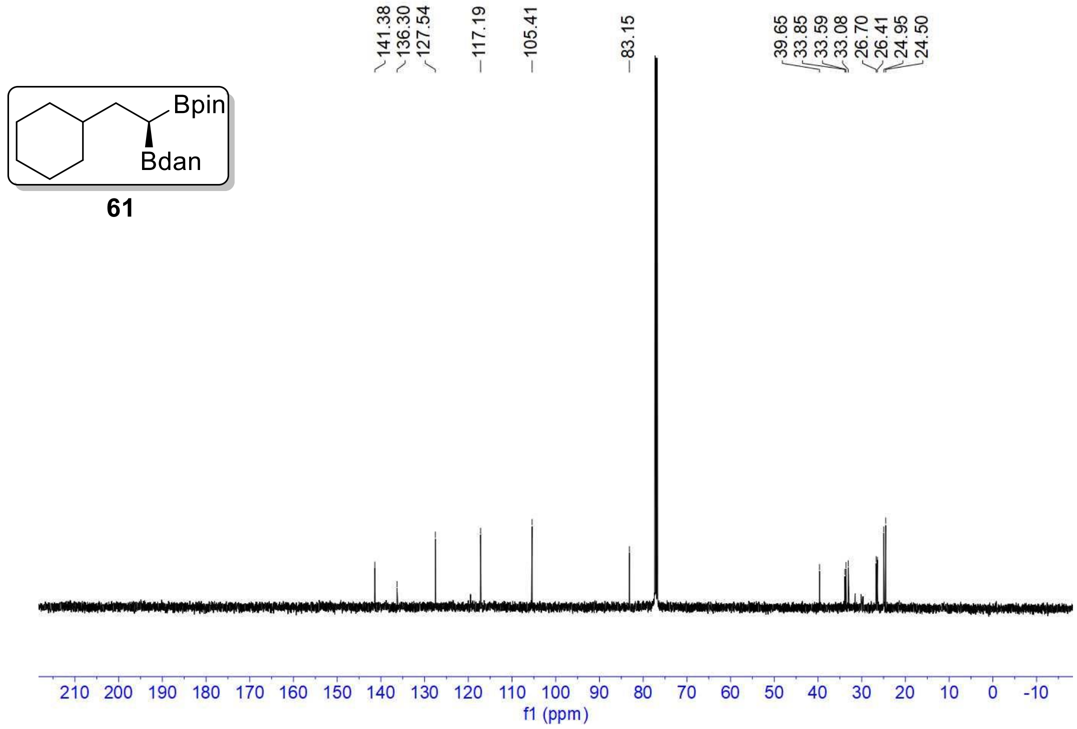

Supplementary Figure 194. ^{13}C NMR spectrum of compound 61

^{11}B NMR (160 MHz, room temperature, $CDCl_3$)

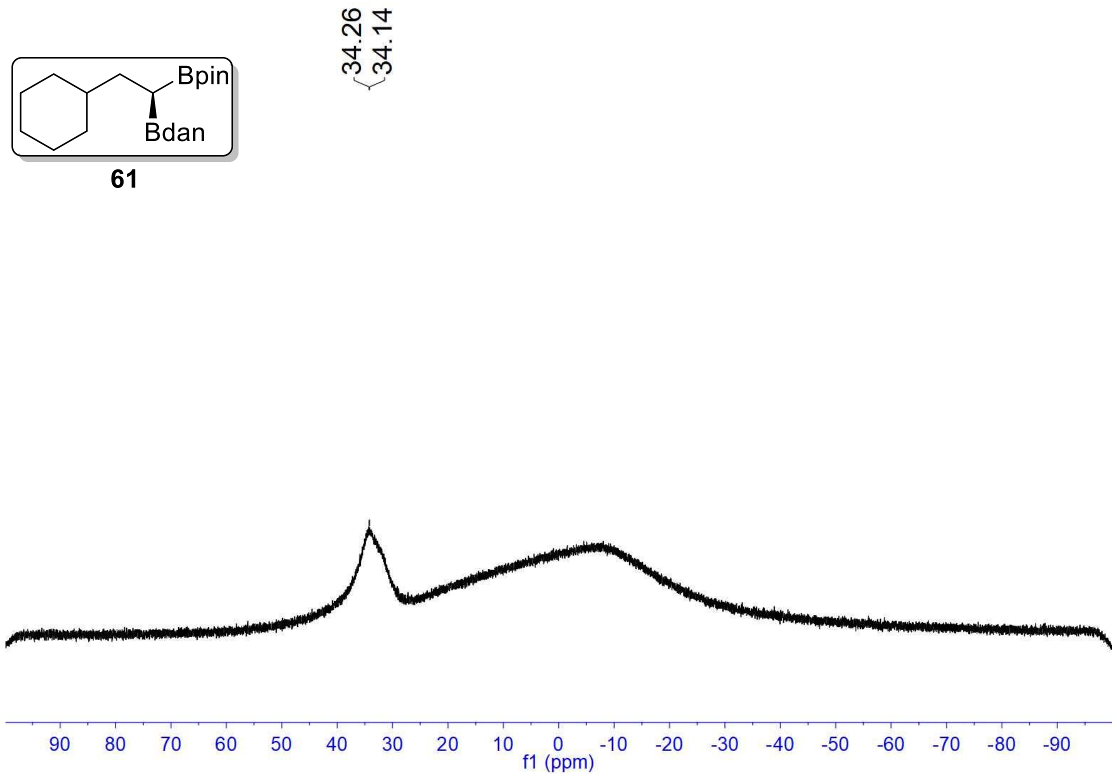

Supplementary Figure 195. ^{11}B NMR spectrum of compound 61

(R)-2-(2-cyclopentyl-1-(4,4,5,5-tetramethyl-1,3,2-dioxaborolan-2-yl)ethyl)-2,3-dihydro-1H-naphtho[1,8-de][1,3,2]diazaborinine (62)

^{1}H NMR (500 MHz, room temperature, $CDCl_3$)

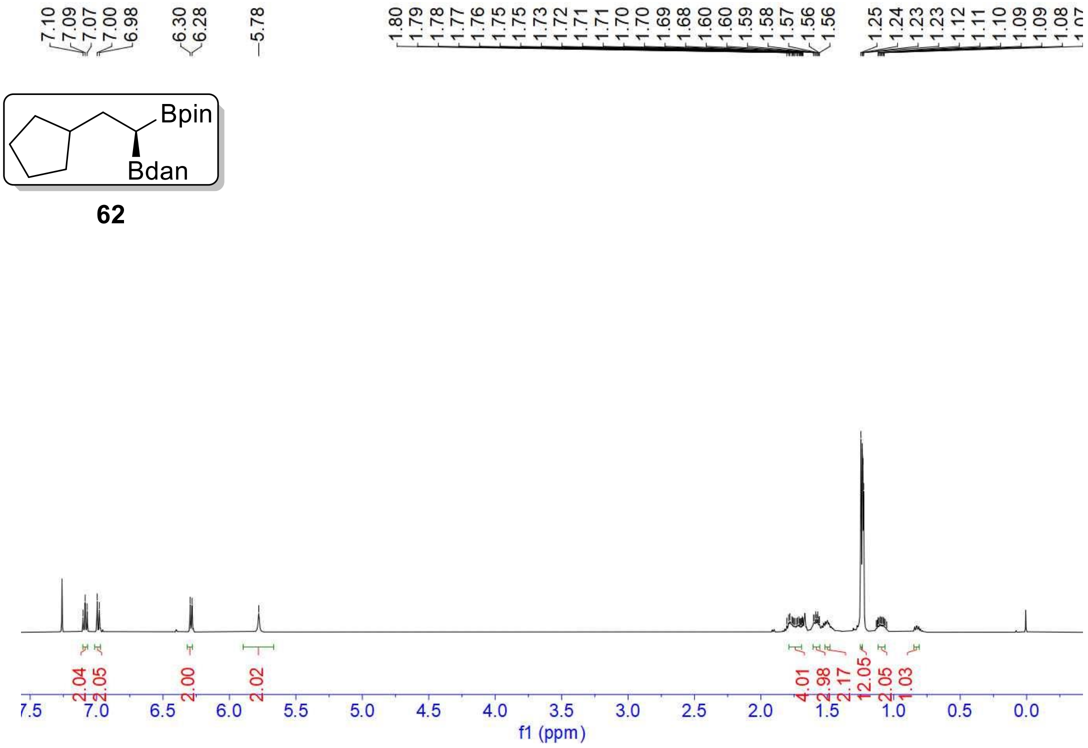

Supplementary Figure 196. ^{1}H NMR spectrum of compound 62

^{13}C NMR (126 MHz, room temperature, $CDCl_3$)

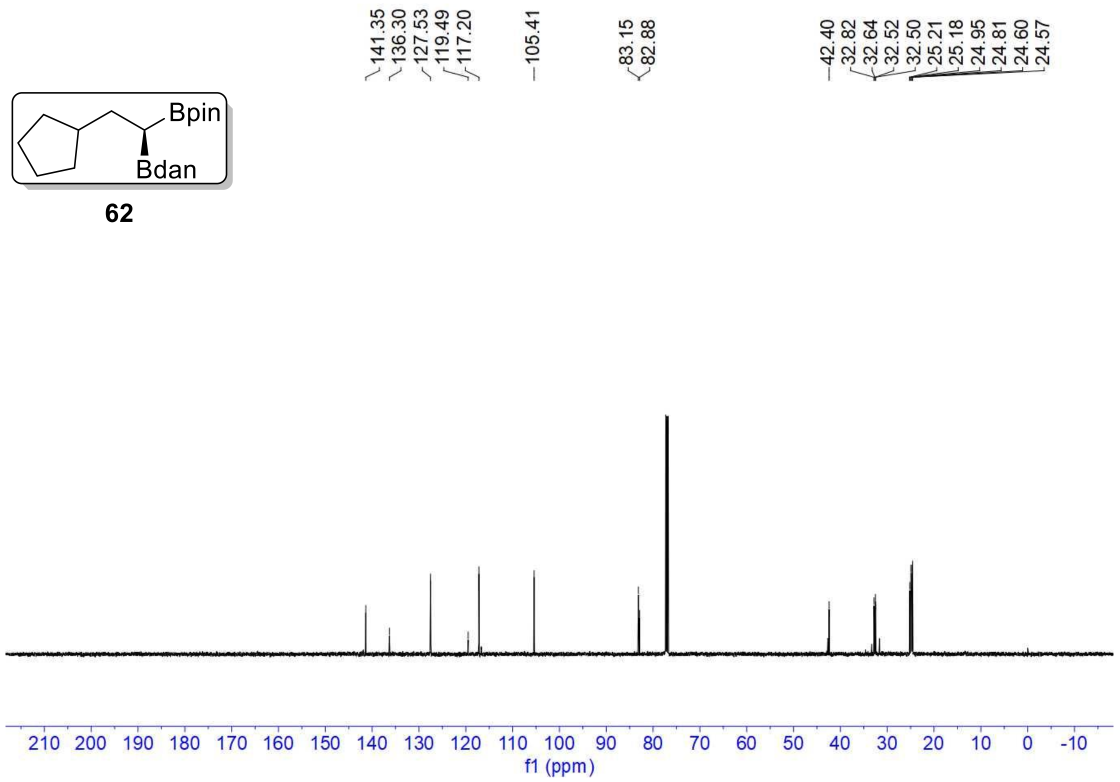

Supplementary Figure 197. ^{13}C NMR spectrum of compound 62

^{11}B NMR (160 MHz, room temperature, $CDCl_3$)

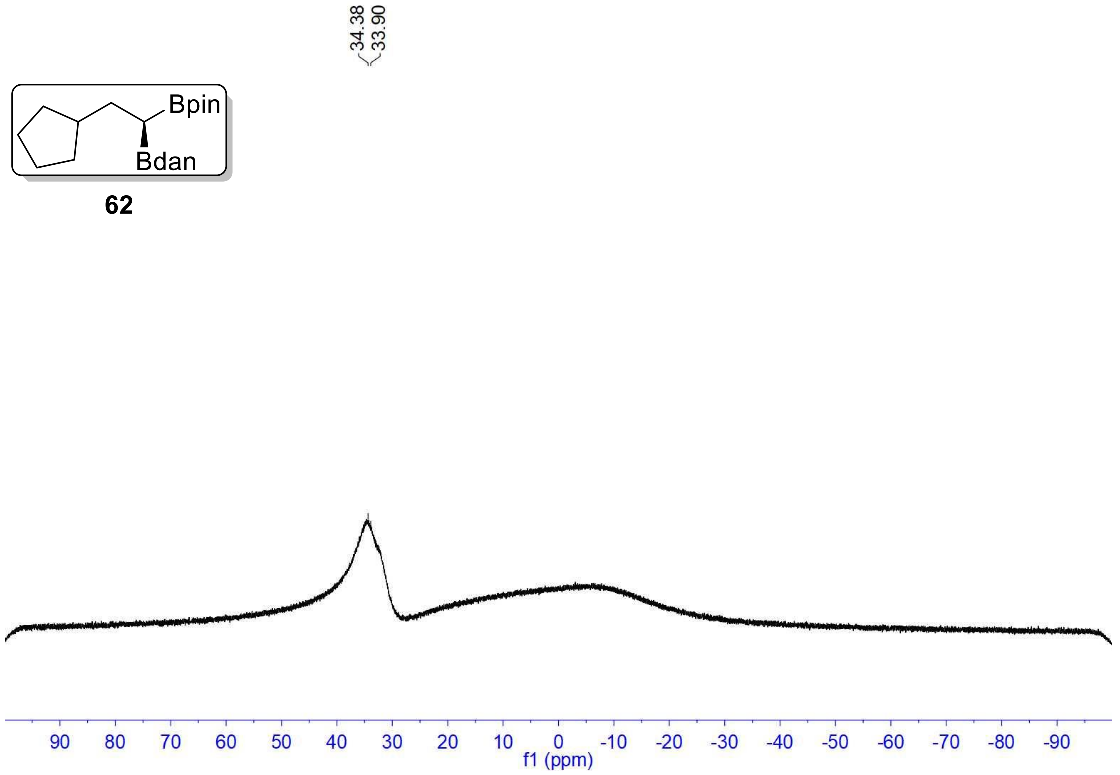

Supplementary Figure 198. ^{11}B NMR spectrum of compound 62

(R)-2-(2-cyclopropyl-1-(4,4,5,5-tetramethyl-1,3,2-dioxaborolan-2-yl)ethyl)-2,3-dihydro-1H-naphtho[1,8-de][1,3,2]diazaborinine (63)

^{1}H NMR (500 MHz, room temperature, $CDCl_3$)

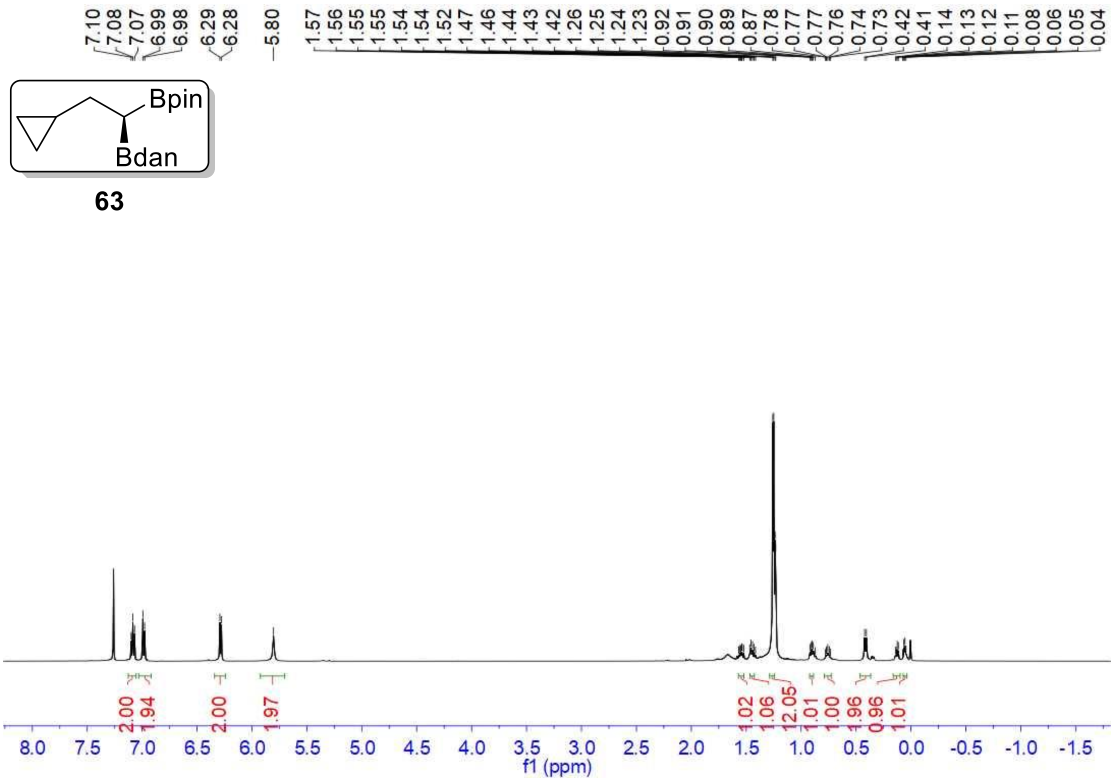

Supplementary Figure 199. ^{1}H NMR spectrum of compound 63

^{13}C NMR (126 MHz, room temperature, $CDCl_3$)

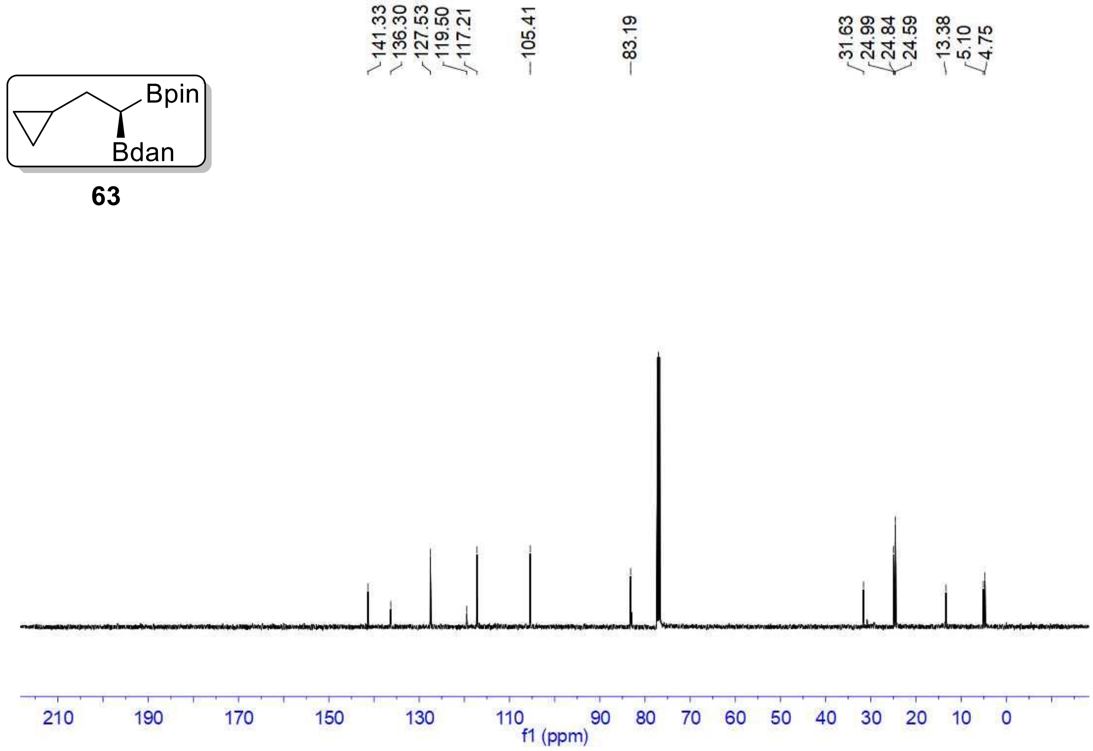

Supplementary Figure 200. ^{13}C NMR spectrum of compound 63

^{11}B NMR (160 MHz, room temperature, $CDCl_3$)

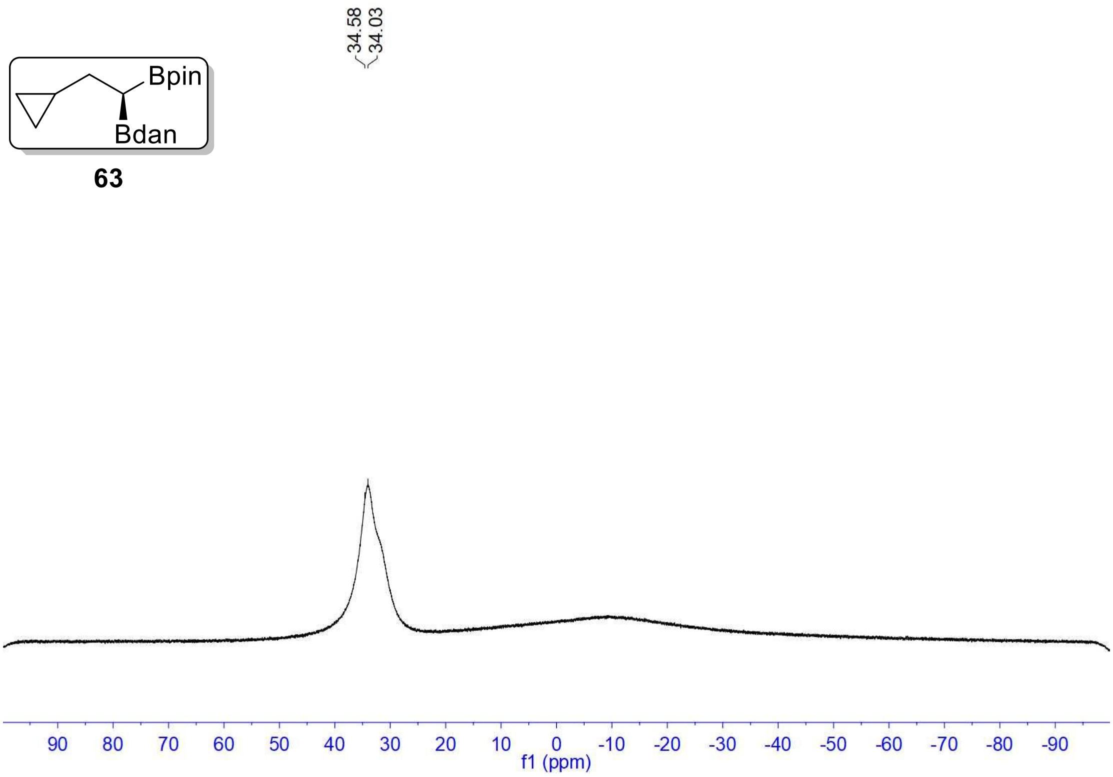

Supplementary Figure 201. ^{11}B NMR spectrum of compound 63

(R)-2-(3,3-dimethyl-1-(4,4,5,5-tetramethyl-1,3,2-dioxaborolan-2-yl)butyl)-2,3-dihydro-1H-naphtho[1,8-de][1,3,2]diazaborinine (64)

^{1}H NMR (500 MHz, room temperature, $CDCl_3$)

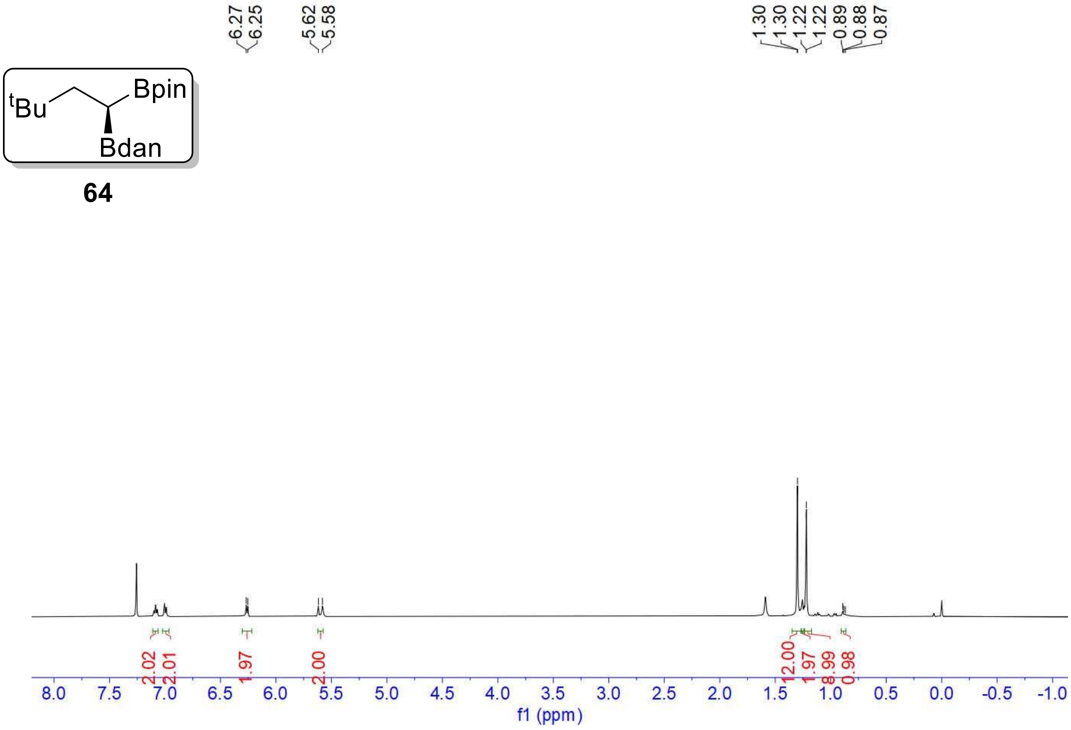

Supplementary Figure 202. ^{1}H NMR spectrum of compound 64

^{13}C NMR (126 MHz, room temperature, $CDCl_3$)

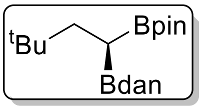

64

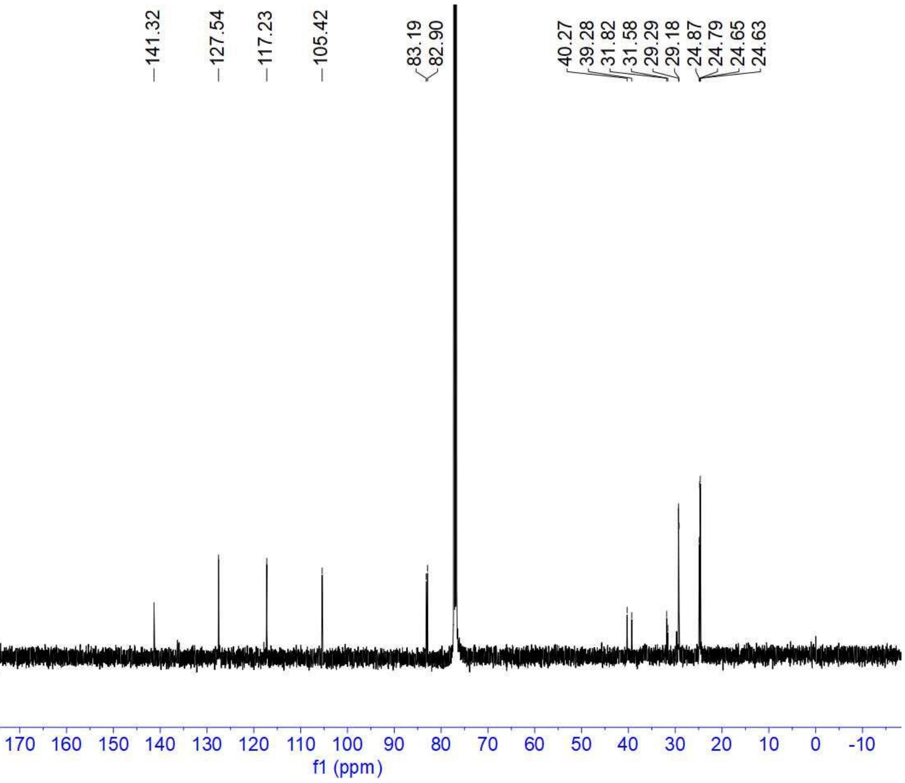

Supplementary Figure 203. ^{13}C NMR spectrum of compound 64

^{11}B NMR (160 MHz, room temperature, $CDCl_3$)

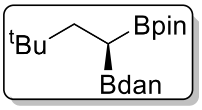

64

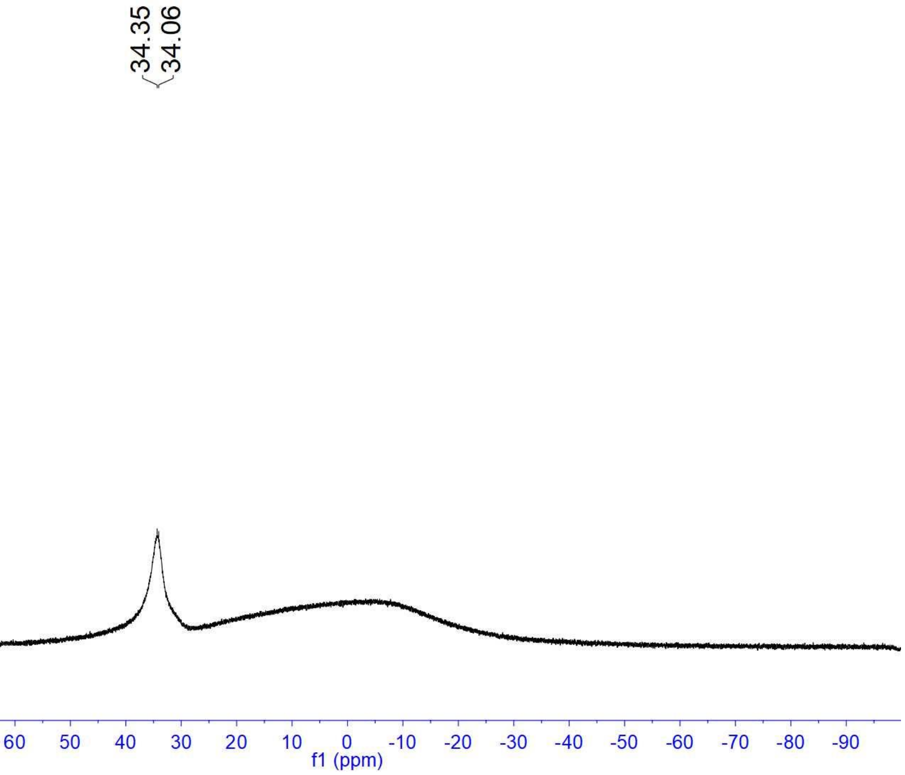

Supplementary Figure 204. ^{11}B NMR spectrum of compound 64

(R)-2-(1-(4,4,5,5-tetramethyl-1,3,2-dioxaborolan-2-yl)pentyl)-2,3-dihydro-1H-naphtho[1,8-de][1,3,2]diazaborinine (65)

^{1}H NMR (500 MHz, room temperature, $CDCl_3$)

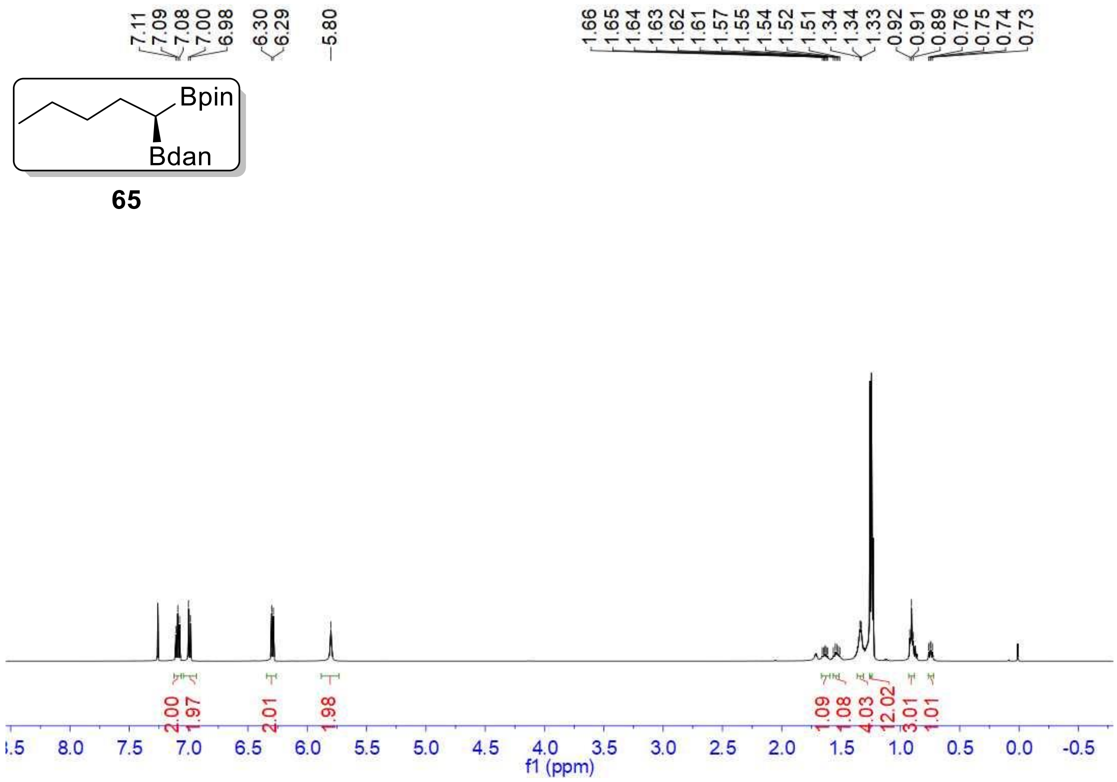

Supplementary Figure 205. ^{1}H NMR spectrum of compound 65

^{13}C NMR (126 MHz, room temperature, $CDCl_3$)

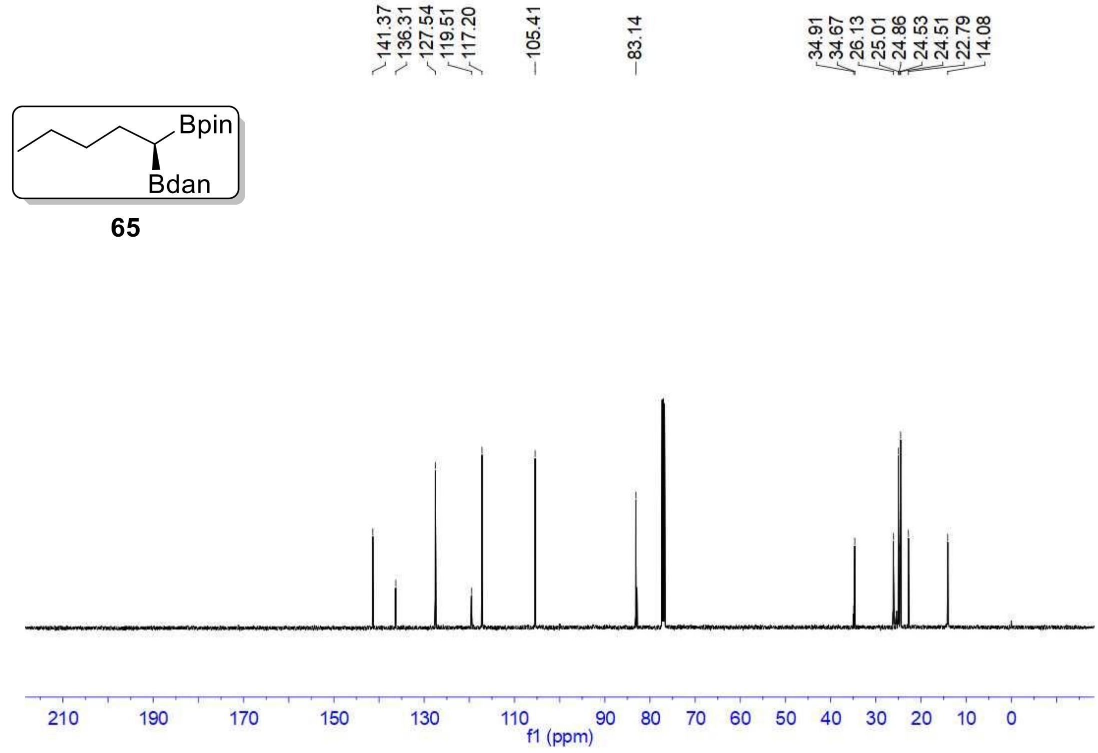

Supplementary Figure 206. ^{13}C NMR spectrum of compound 65

^{11}B NMR (160 MHz, room temperature, CDCl$_3$)

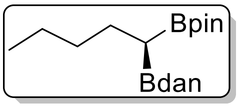

65

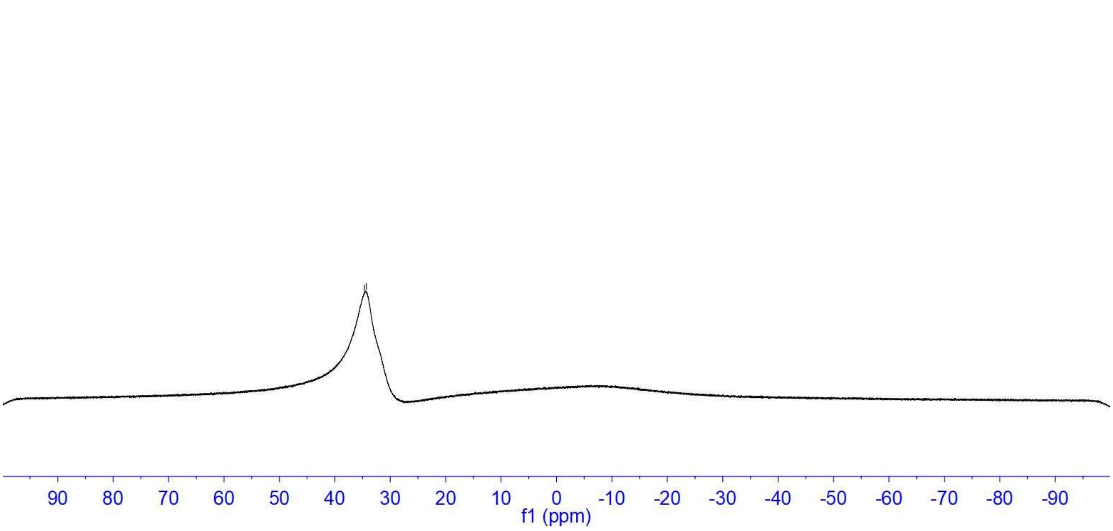

Supplementary Figure 207. ^{11}B NMR spectrum of compound 65

(R)-2-(1-(4,4,5,5-tetramethyl-1,3,2-dioxaborolan-2-yl)hexyl)-2,3-dihydro-1H-naphtho[1,8-de][1,3,2]diazaborinine (66)

^{1}H NMR (500 MHz, room temperature, CDCl$_3$)

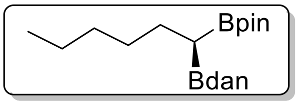

66

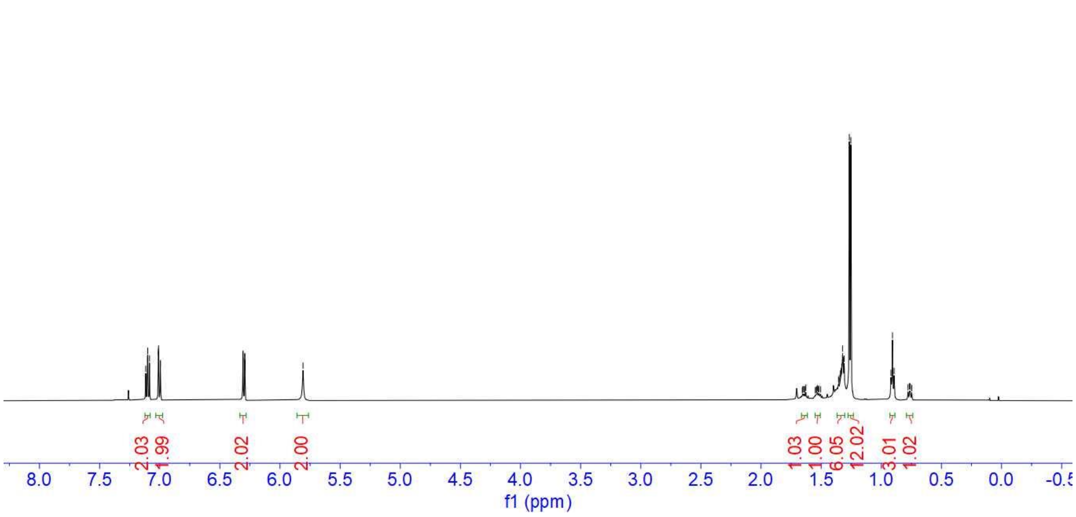

Supplementary Figure 208. ^{1}H NMR spectrum of compound 66

^{13}C NMR (126 MHz, room temperature, $CDCl_3$)

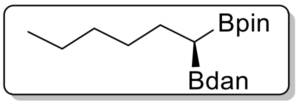

66

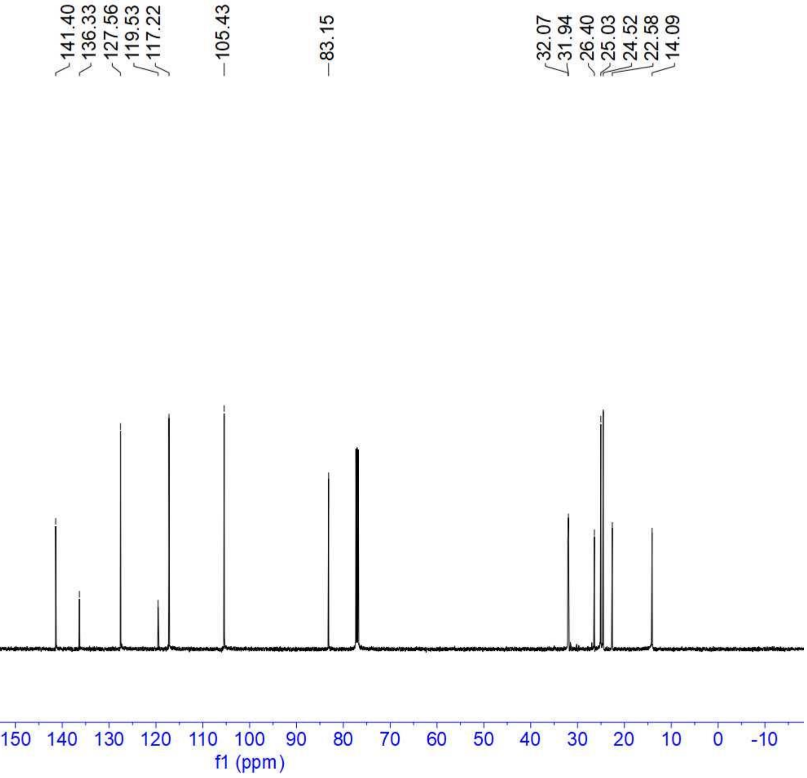

Supplementary Figure 209. ^{13}C NMR spectrum of compound 66

^{11}B NMR (160 MHz, room temperature, $CDCl_3$)

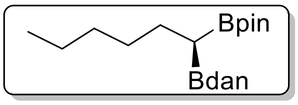

66

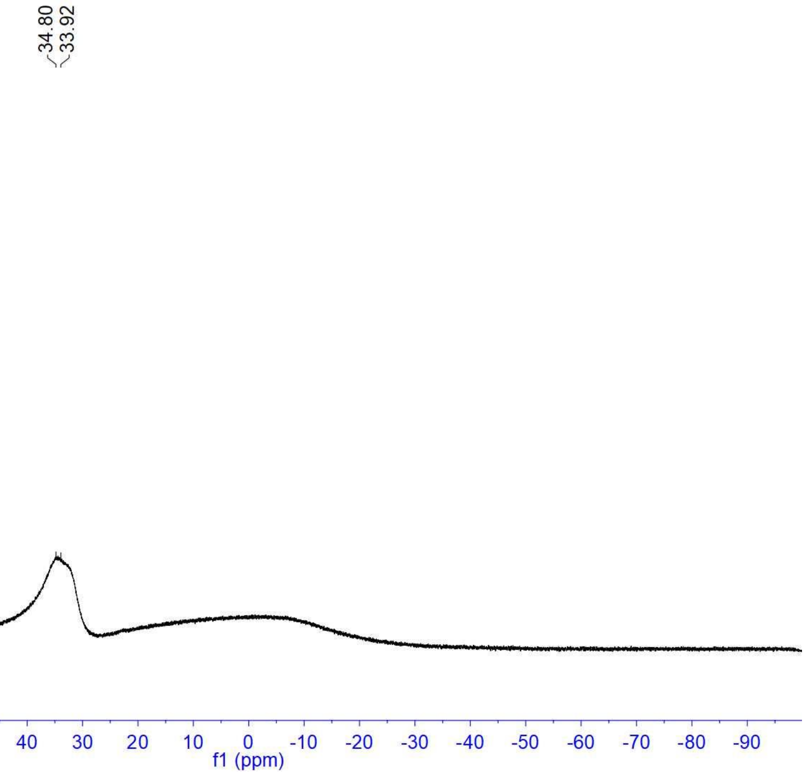

Supplementary Figure 210. ^{11}B NMR spectrum of compound 66

(R)-2-(1-(4,4,5,5-tetramethyl-1,3,2-dioxaborolan-2-yl)heptyl)-2,3-dihydro-1H-naphtho[1,8-de][1,3,2]diazaborinine (67)

^{1}H NMR (500 MHz, room temperature, $CDCl_3$)

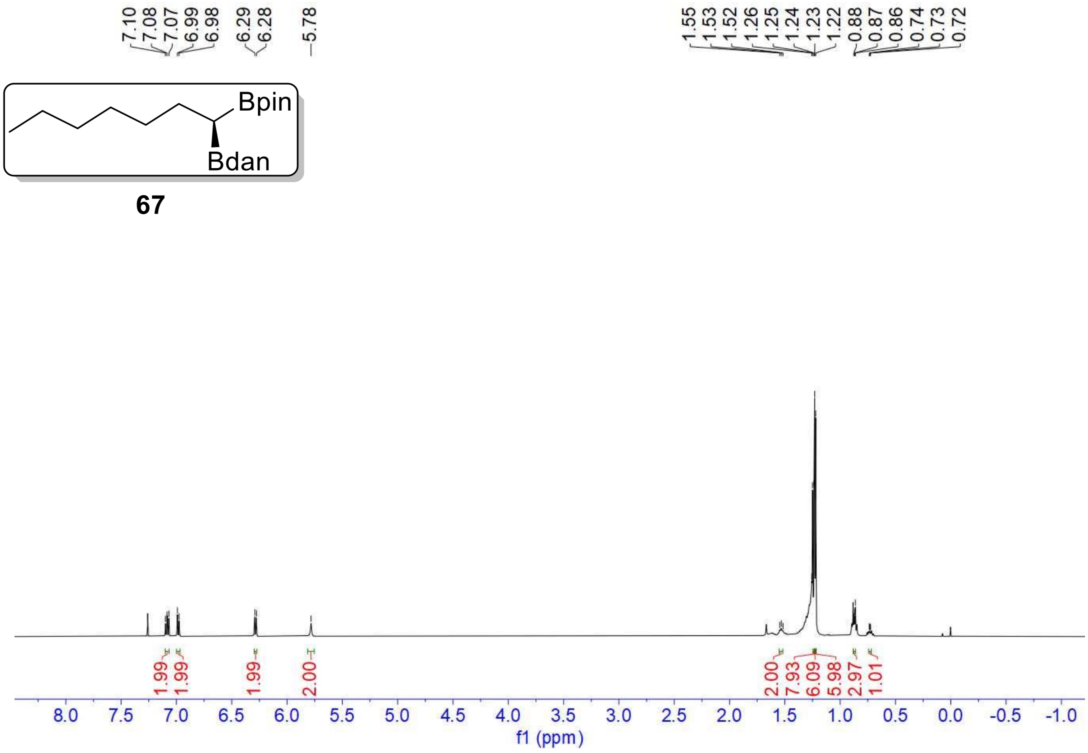

Supplementary Figure 211. ^{1}H NMR spectrum of compound 67

^{13}C NMR (126 MHz, room temperature, $CDCl_3$)

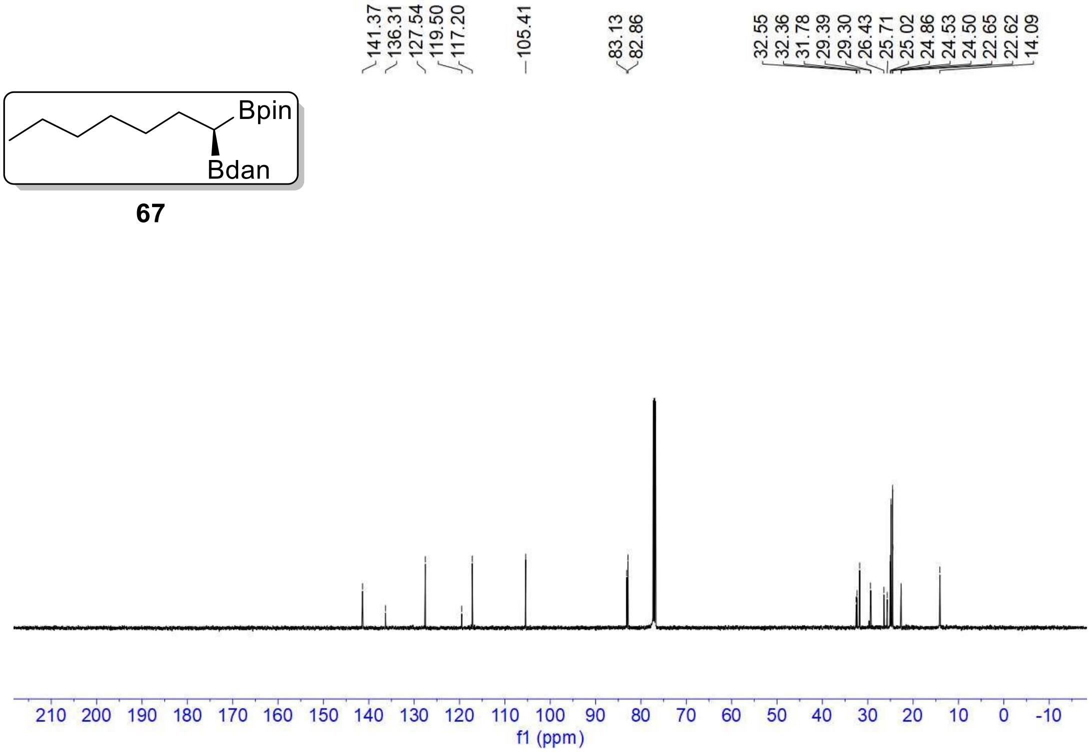

Supplementary Figure 212. ^{13}C NMR spectrum of compound 67

^{11}B NMR (160 MHz, room temperature, $CDCl_3$)

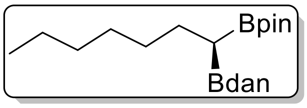

67

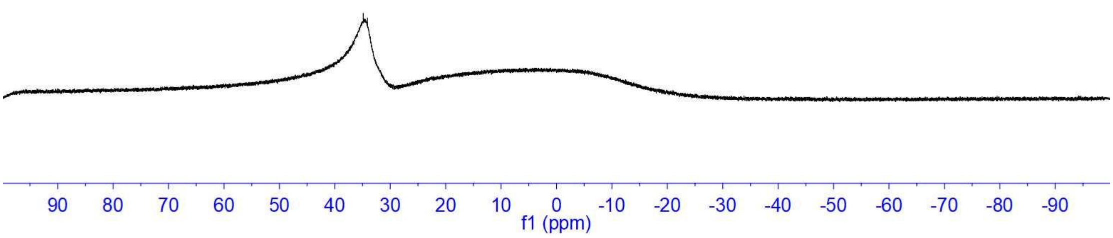

Supplementary Figure 213. ^{11}B NMR spectrum of compound 67

(R)-2-(1-(4,4,5,5-tetramethyl-1,3,2-dioxaborolan-2-yl)octyl)-2,3-dihydro-1H-naphtho[1,8-de][1,3,2]diazaborinine (68)

^{1}H NMR (500 MHz, room temperature, $CDCl_3$)

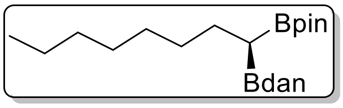

68

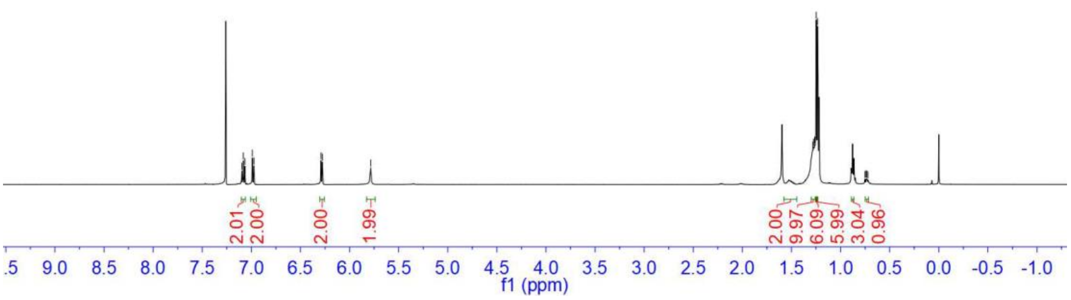

Supplementary Figure 214. ^{1}H NMR spectrum of compound 68

^{13}C NMR (126 MHz, room temperature, $CDCl_3$)

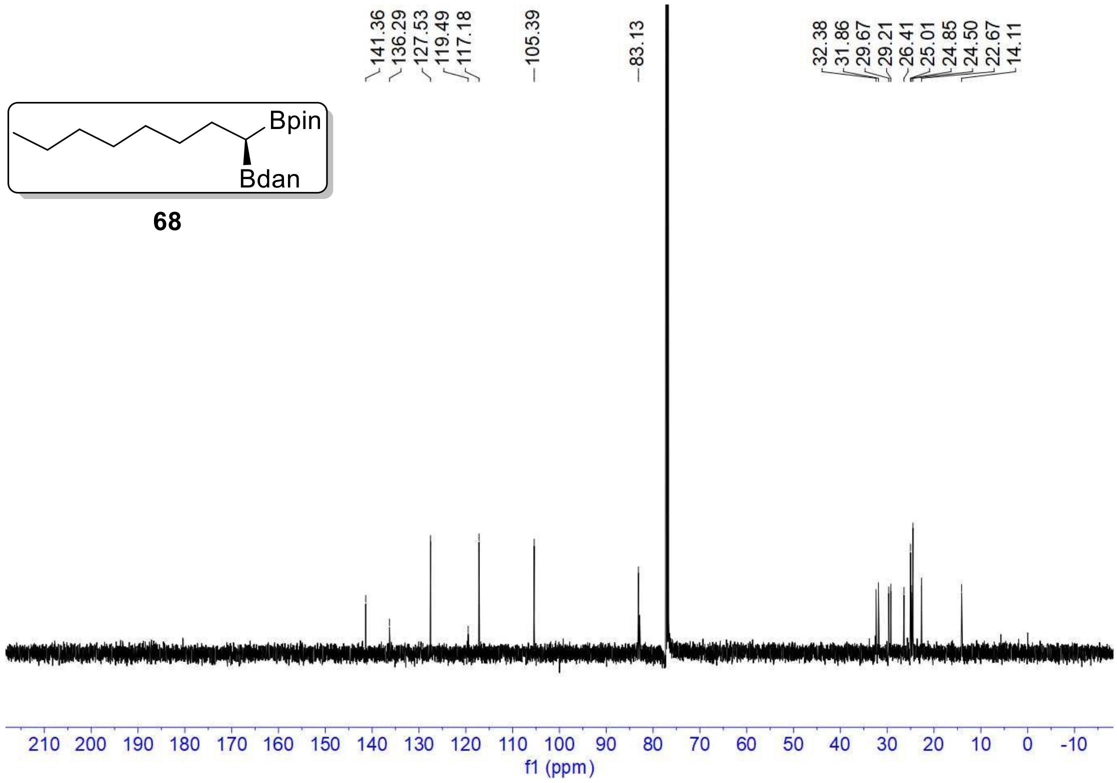

Supplementary Figure 215. ^{13}C NMR spectrum of compound 68

^{11}B NMR (160 MHz, room temperature, $CDCl_3$)

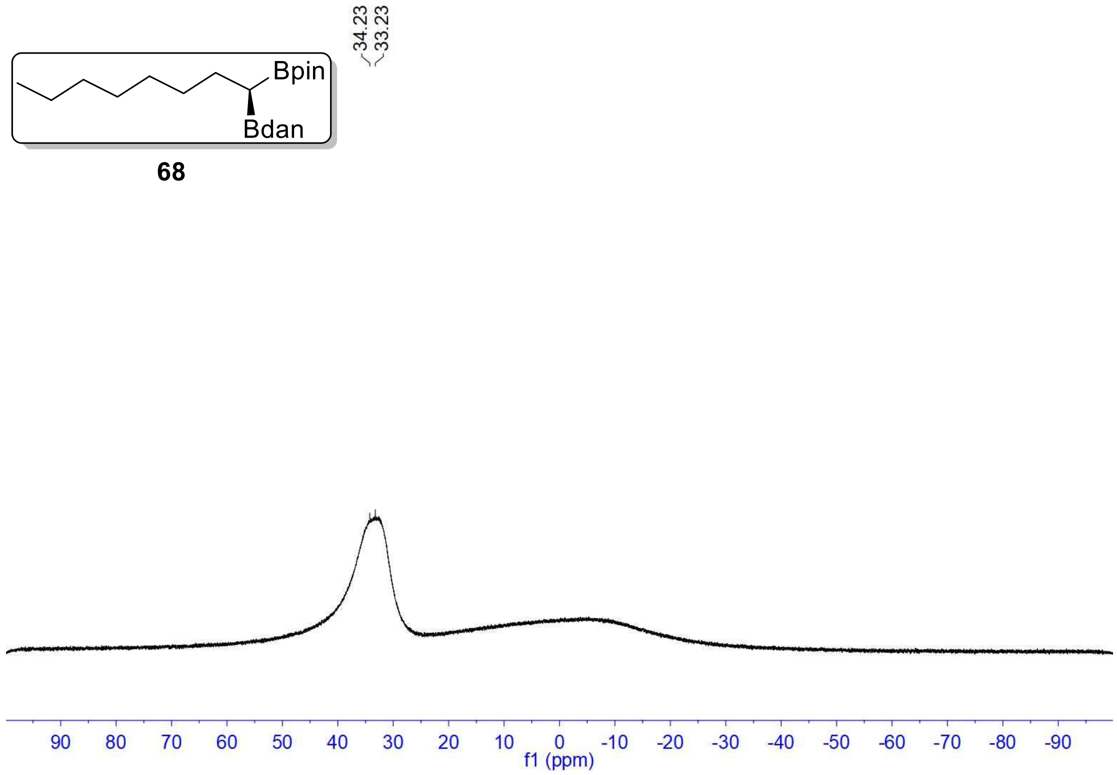

Supplementary Figure 216. ^{11}B NMR spectrum of compound 68

(R)-2-(1-(4,4,5,5-tetramethyl-1,3,2-dioxaborolan-2-yl)nonyl)-2,3-dihydro-1H-naphtho[1,8-de][1,3,2]diazaborinine (69)

^{1}H NMR (500 MHz, room temperature, $CDCl_3$)

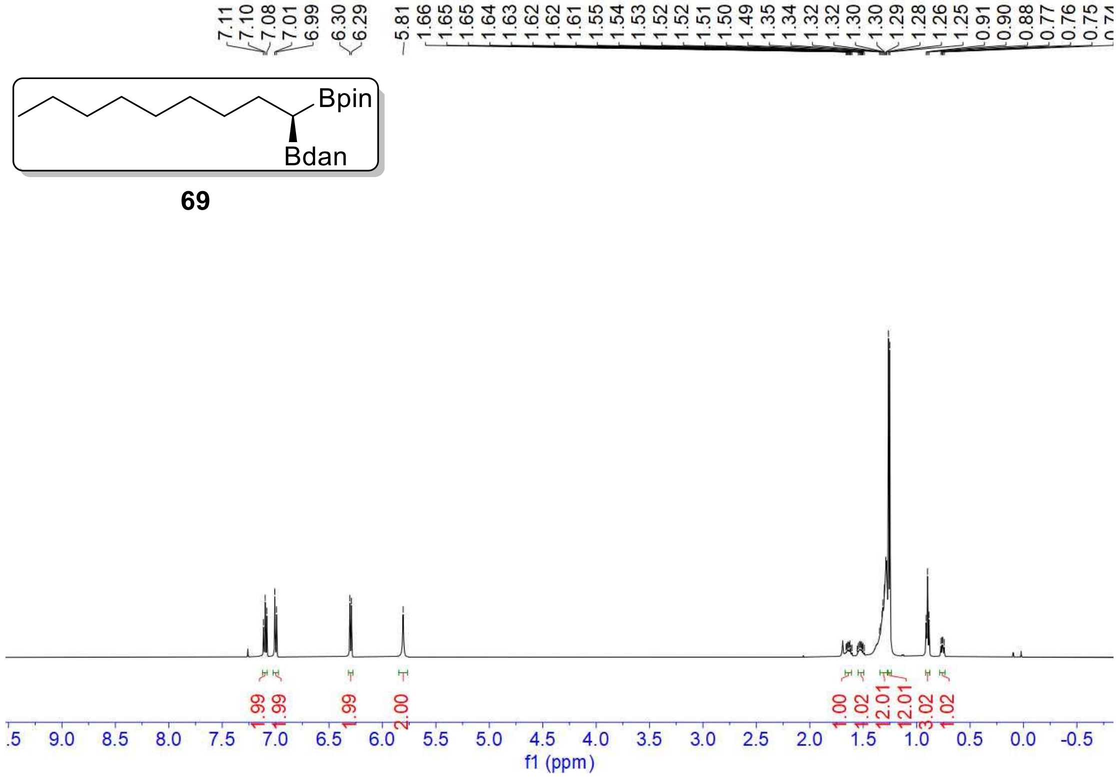

Supplementary Figure 217. ^{1}H NMR spectrum of compound 69

^{13}C NMR (126 MHz, room temperature, $CDCl_3$)

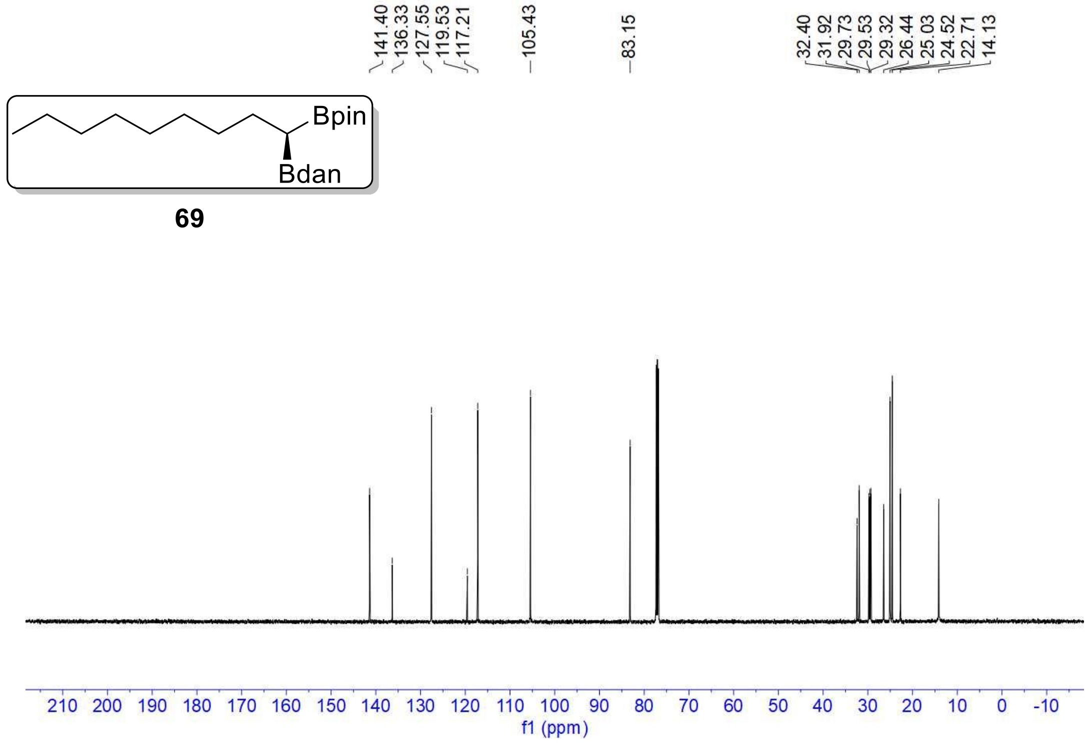

Supplementary Figure 218. ^{13}C NMR spectrum of compound 69

^{11}B NMR (160 MHz, room temperature, $CDCl_3$)

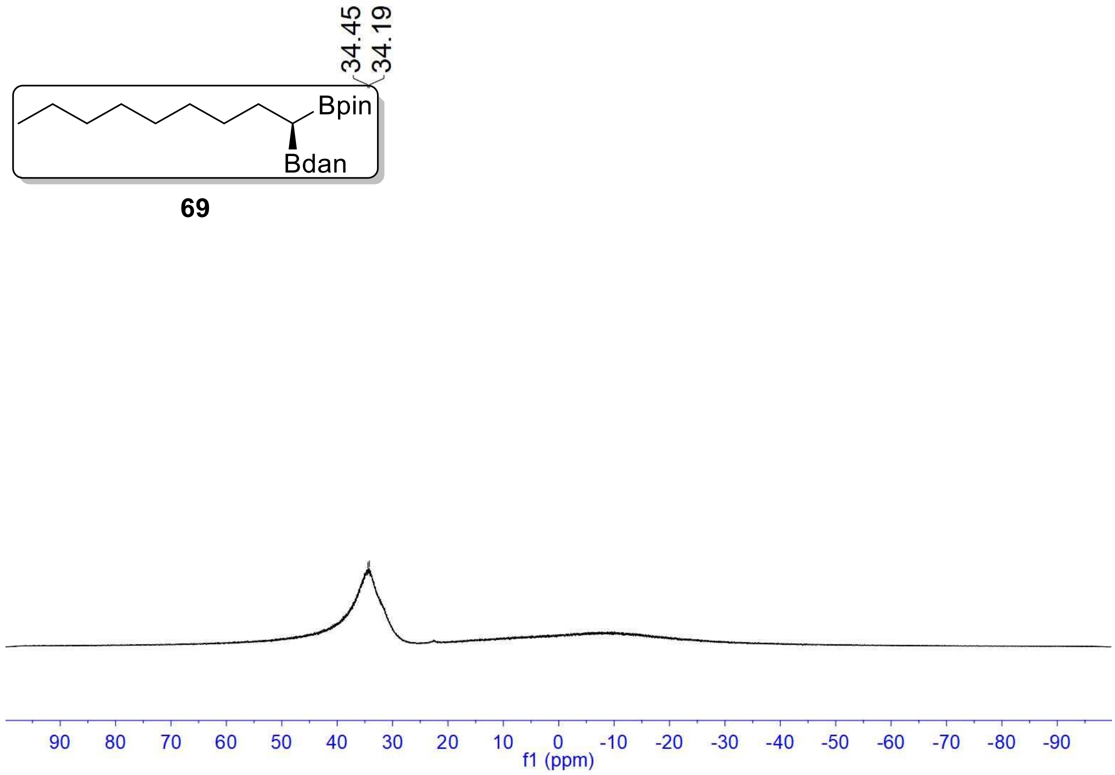

Supplementary Figure 219. ^{11}B NMR spectrum of compound 69

(R)-2-(5-methyl-1-(4,4,5,5-tetramethyl-1,3,2-dioxaborolan-2-yl)hexyl)-2,3-dihydro-1H-naphtho[1,8-de][1,3,2]diazaborinine (70)

^{1}H NMR (500 MHz, room temperature, $CDCl_3$)

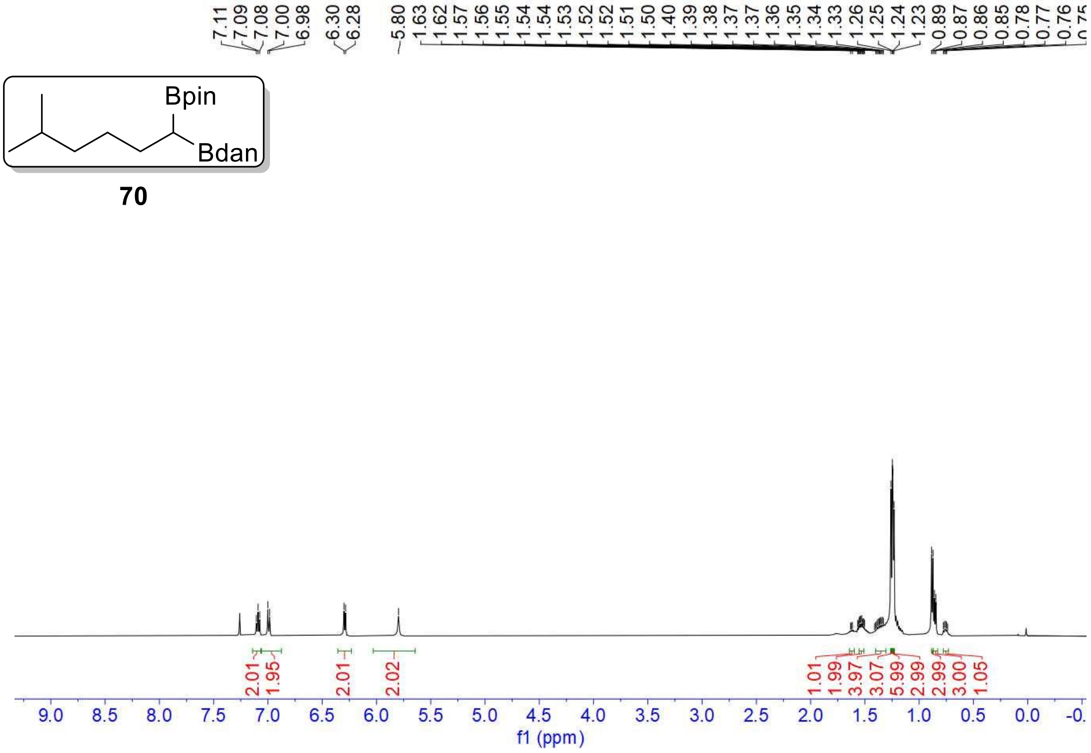

Supplementary Figure 220. ^{1}H NMR spectrum of compound 70

^{13}C NMR (126 MHz, room temperature, CDCl$_3$)

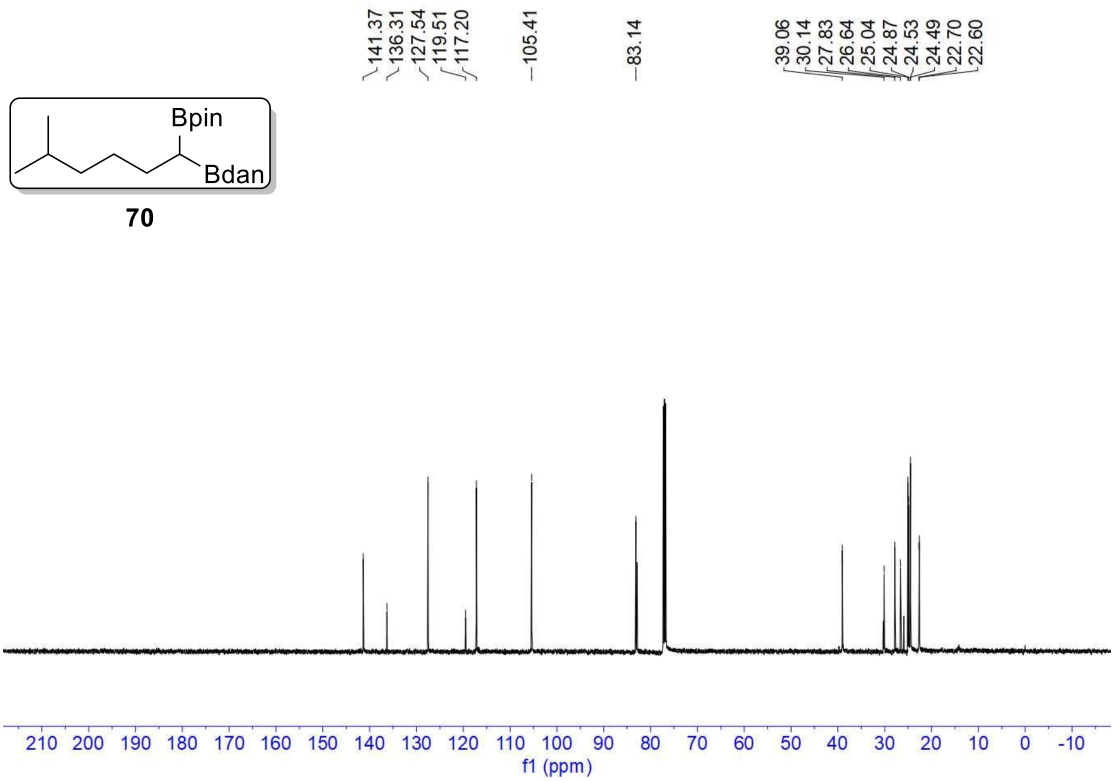

Supplementary Figure 221. ^{13}C NMR spectrum of compound 70

^{11}B NMR (160 MHz, room temperature, CDCl$_3$)

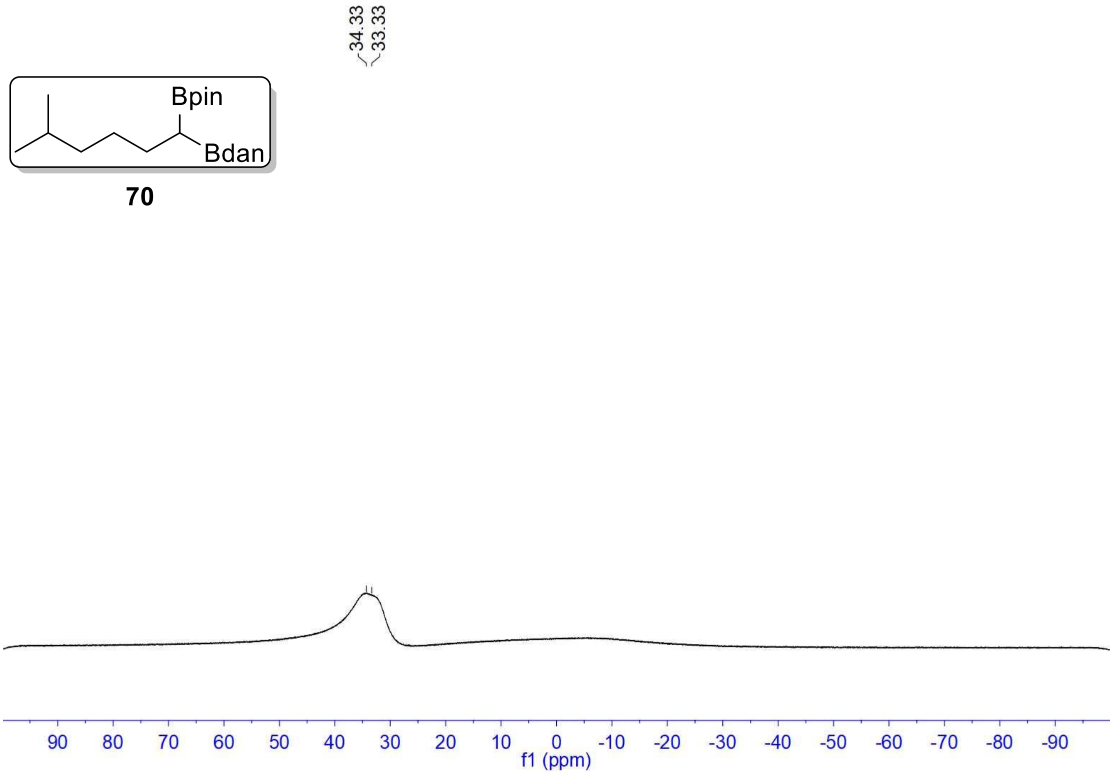

Supplementary Figure 222. ^{11}B NMR spectrum of compound 70

(R)-tert-butyl 4-(2-(1H-naphtho[1,8-de][1,3,2]diazaborinin-2(3H)-yl)-2-(4,4,5,5-tetramethyl-1,3,2-dioxaborolan-2-yl)ethyl)piperidine-1-carboxylate (71)

^{1}H NMR (500 MHz, room temperature, CDCl$_3$)

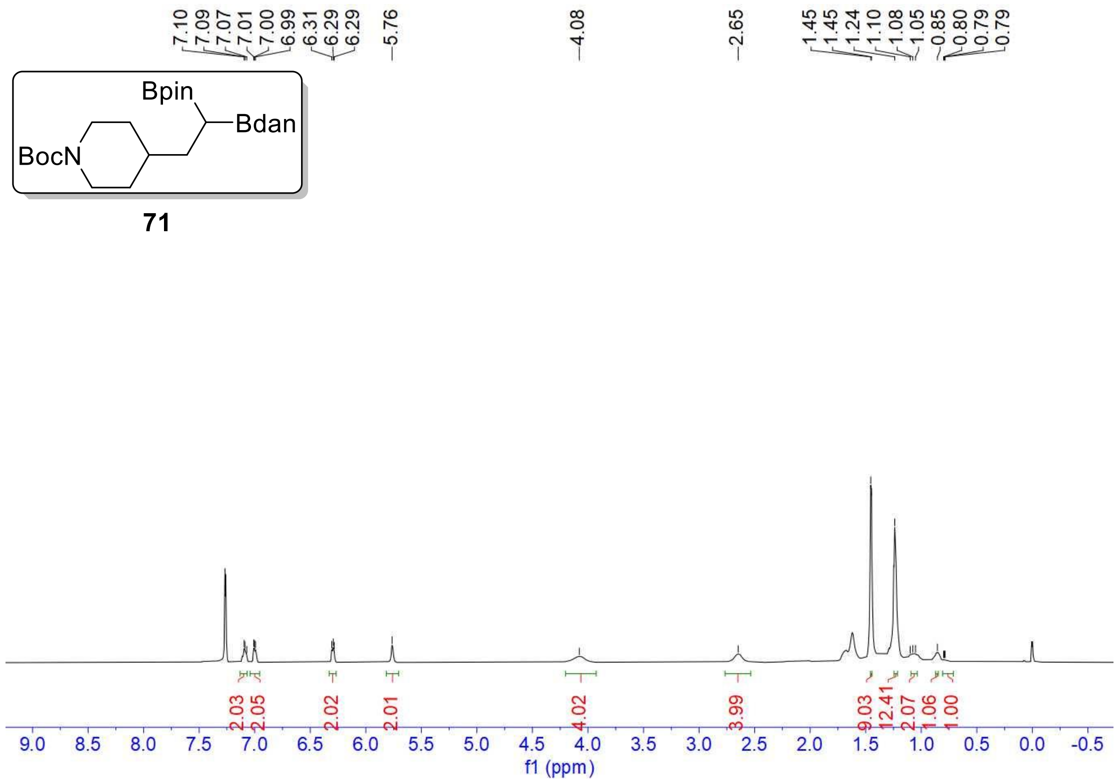

Supplementary Figure 223. ^{1}H NMR spectrum of compound 71

^{13}C NMR (126 MHz, room temperature, CDCl$_3$)

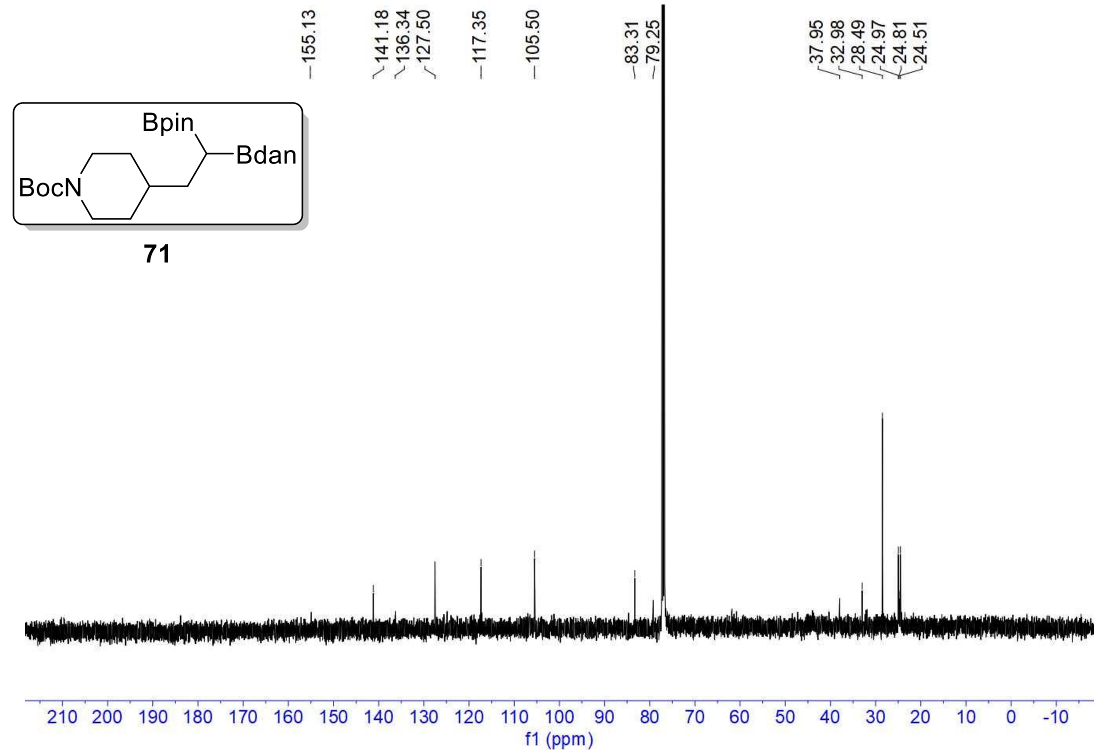

Supplementary Figure 224. ^{13}C NMR spectrum of compound 71

^{11}B NMR (160 MHz, room temperature, $CDCl_3$)

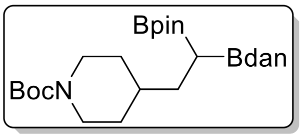

71

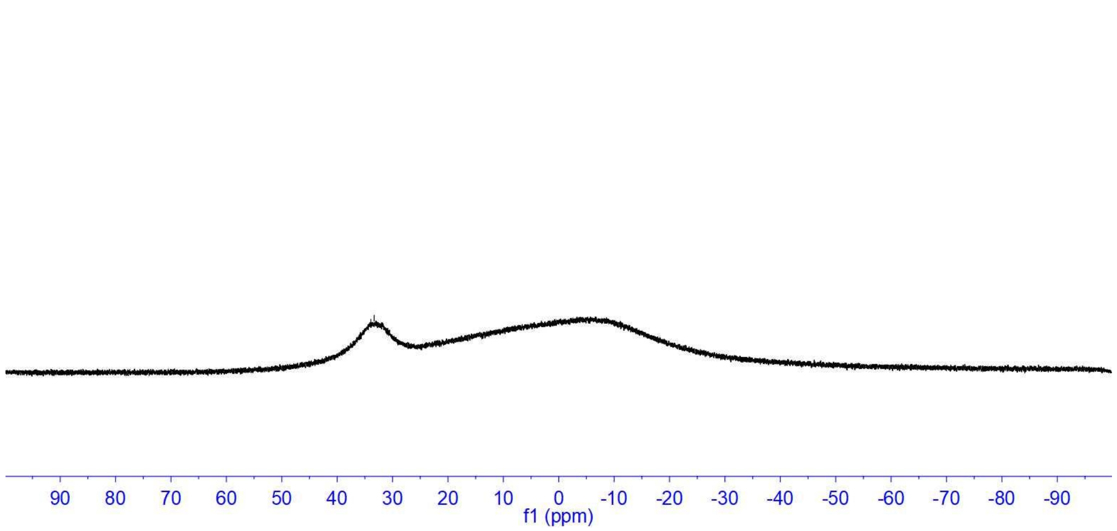

Supplementary Figure 225. ^{11}B NMR spectrum of compound 71

(R)-2-(4-((tert-butyldimethylsilyl)oxy)-1-(4,4,5,5-tetramethyl-1,3,2-dioxaborolan-2-yl)butyl)-2,3-dihydro-1H-naphtho[1,8-de][1,3,2]diazaborinine (72)

^{1}H NMR (500 MHz, room temperature, $CDCl_3$)

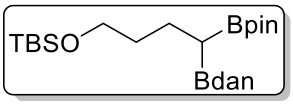

72

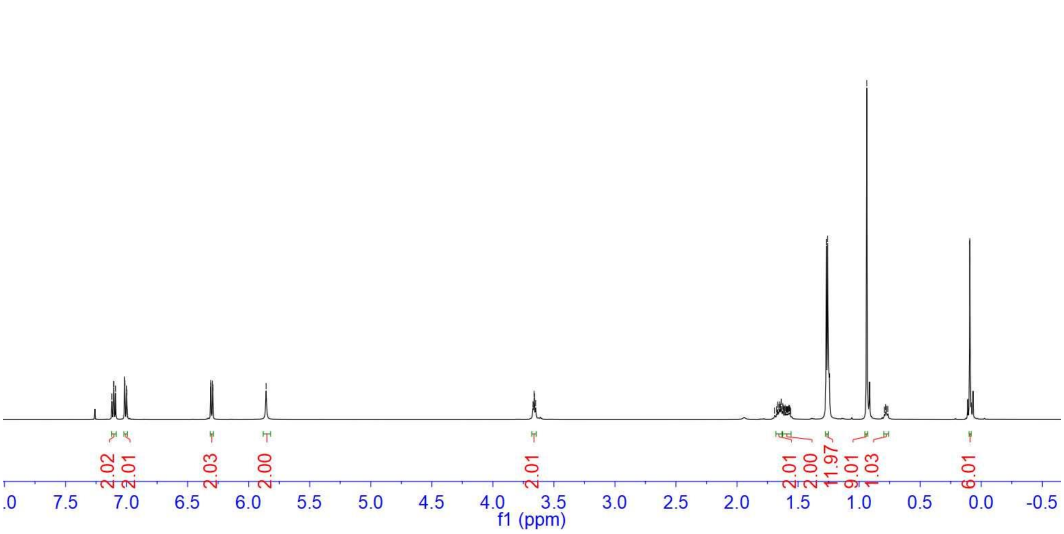

Supplementary Figure 226. ^{1}H NMR spectrum of compound 72

^{13}C NMR (126 MHz, room temperature, $CDCl_3$)

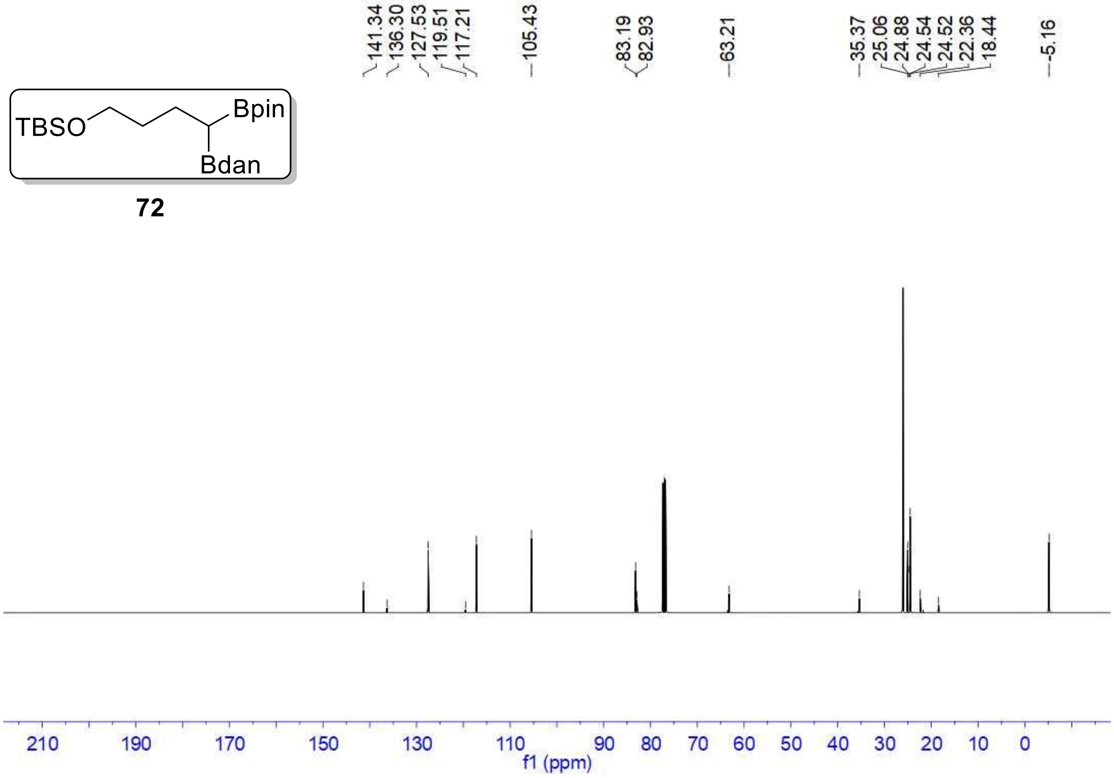

Supplementary Figure 227. ^{13}C NMR spectrum of compound 72

^{11}B NMR (160 MHz, room temperature, $CDCl_3$)

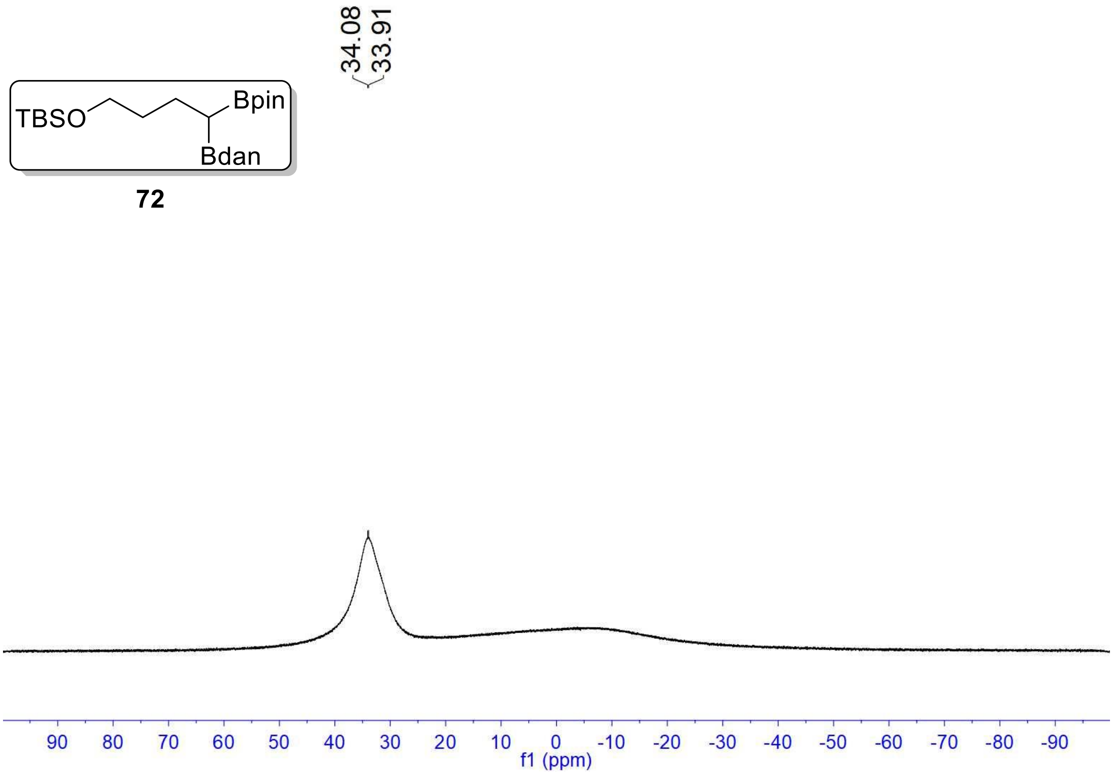

Supplementary Figure 228. ^{11}B NMR spectrum of compound 72

(R)-2-(6-((tert-butyldimethylsilyl)oxy)-1-(4,4,5,5-tetramethyl -1,3,2-dioxaborolan-2-yl)hexyl)-2,3-dihydro-1H-naphtho[1,8-de][1,3,2]diazaborinine (73)

^{1}H NMR (500 MHz, room temperature, $CDCl_3$)

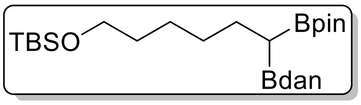

73

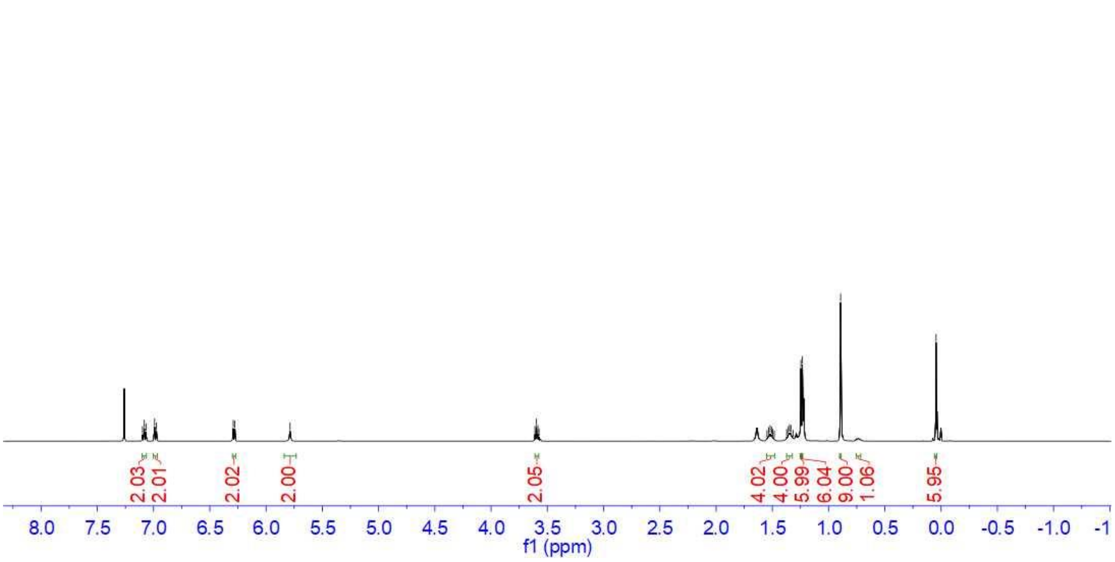

Supplementary Figure 229. ^{1}H NMR spectrum of compound 73

^{13}C NMR (126 MHz, room temperature, $CDCl_3$)

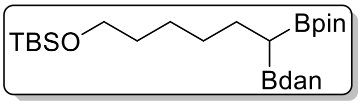

73

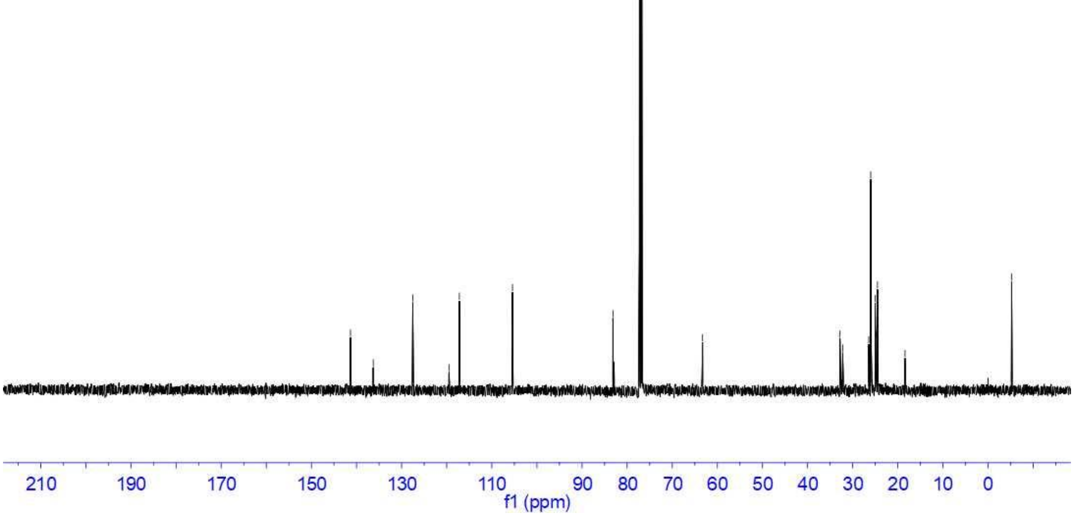

Supplementary Figure 230. ^{13}C NMR spectrum of compound 73

^{11}B NMR (160 MHz, room temperature, CDCl$_3$)

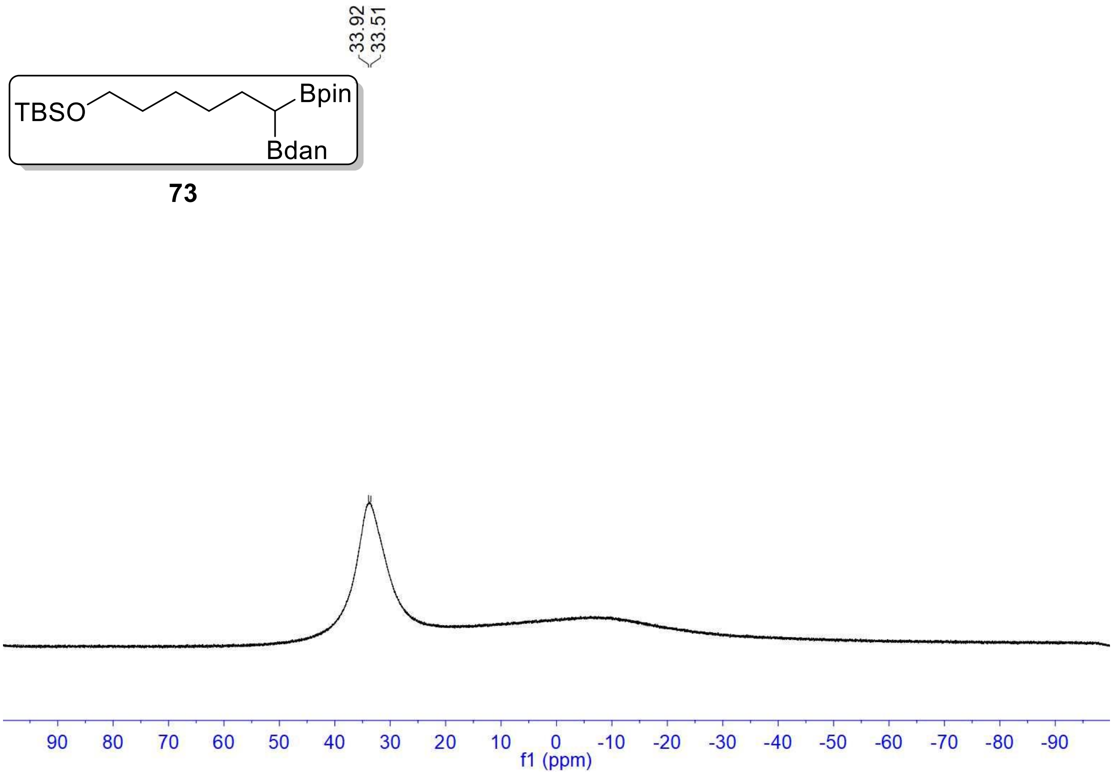

73

Supplementary Figure 231. ^{11}B NMR spectrum of compound 73

(R)-2-(5-((tert-butyldiphenylsilyl)oxy)-1-(4,4,5,5-tetramethyl-1,3,2-dioxaborolan-2-yl)pentyl)-2,3-dihydro-1H-naphtho[1,8-de][1,3,2]diazaborinine (74)

^{1}H NMR (500 MHz, room temperature, CDCl$_3$)

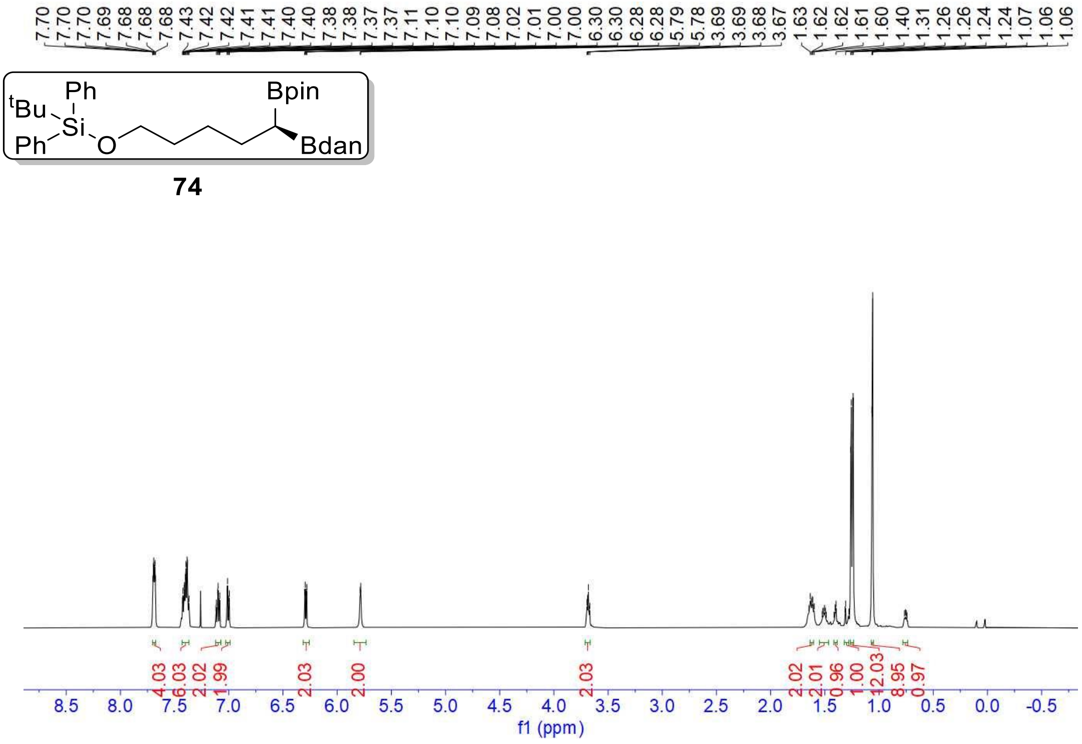

74

Supplementary Figure 232. ^{1}H NMR spectrum of compound 74

^{13}C NMR (126 MHz, room temperature, CDCl$_3$)

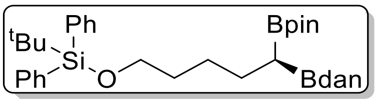

74

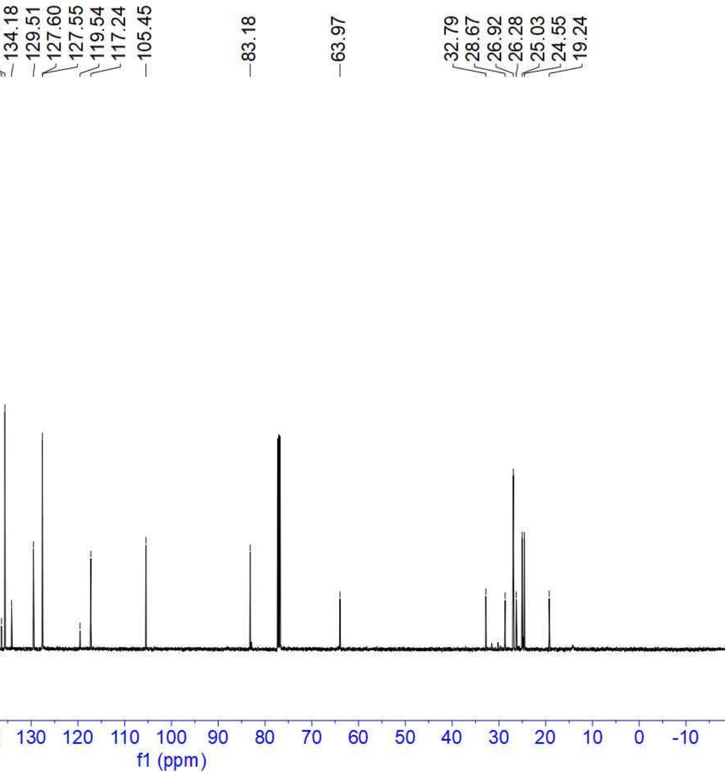

Supplementary Figure 233. ^{13}C NMR spectrum of compound 74

^{11}B NMR (160 MHz, room temperature, CDCl$_3$)

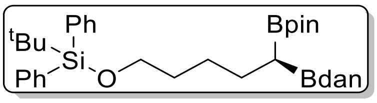

74

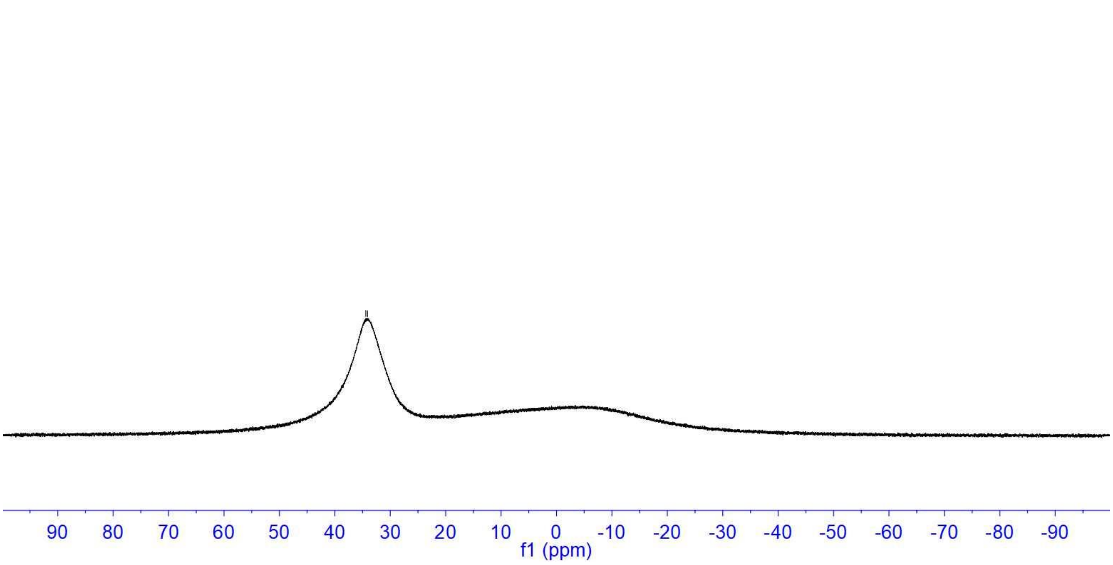

Supplementary Figure 234. ^{11}B NMR spectrum of compound 74

(R)-2-(1-(4,4,5,5-tetramethyl-1,3,2-dioxaborolan-2-yl)-5-((triisopropylsilyl)oxy)pentyl)-2,3-dihydro-1H-naphtho[1,8-de][1,3,2]diazaborinine (75)

^{1}H NMR (500 MHz, room temperature, $CDCl_3$)

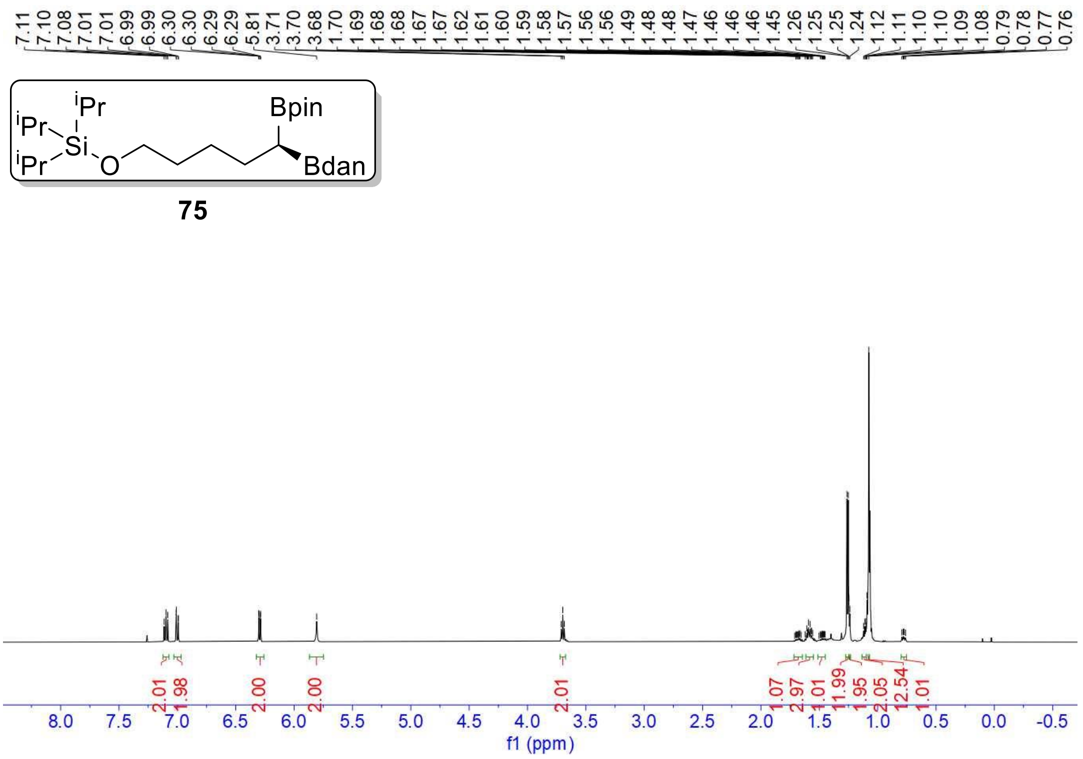

Supplementary Figure 235. ^{1}H NMR spectrum of compound 75

^{13}C NMR (126 MHz, room temperature, $CDCl_3$)

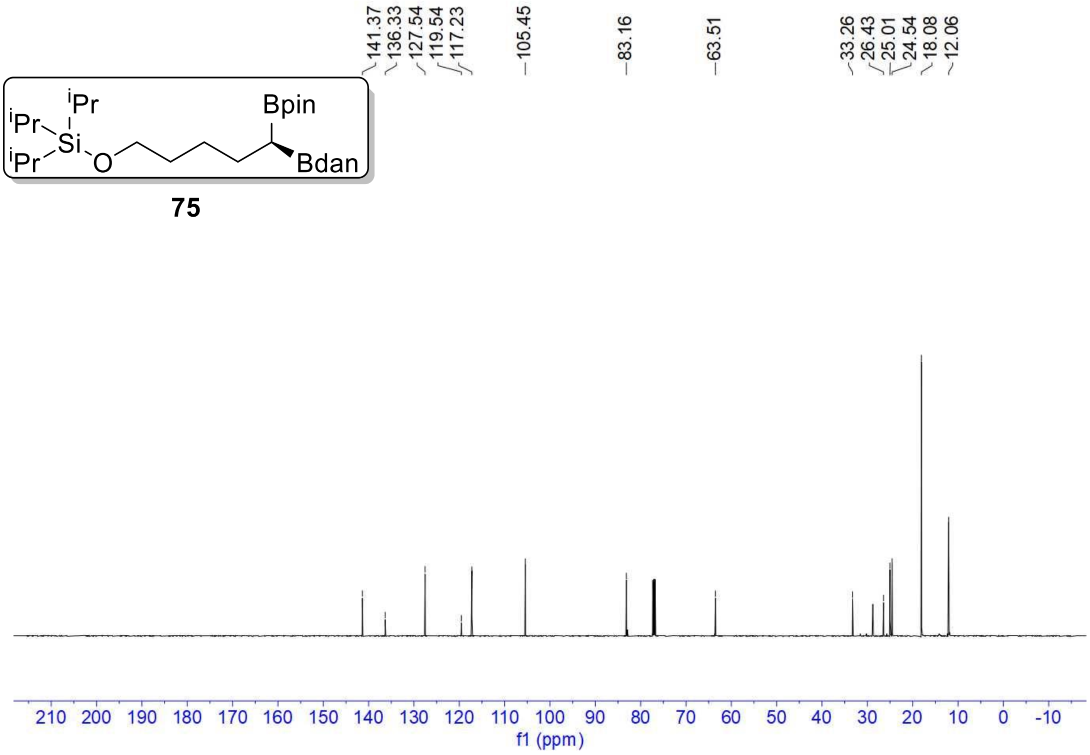

Supplementary Figure 236. ^{13}C NMR spectrum of compound 75

^{11}B NMR (160 MHz, room temperature, CDCl$_3$)

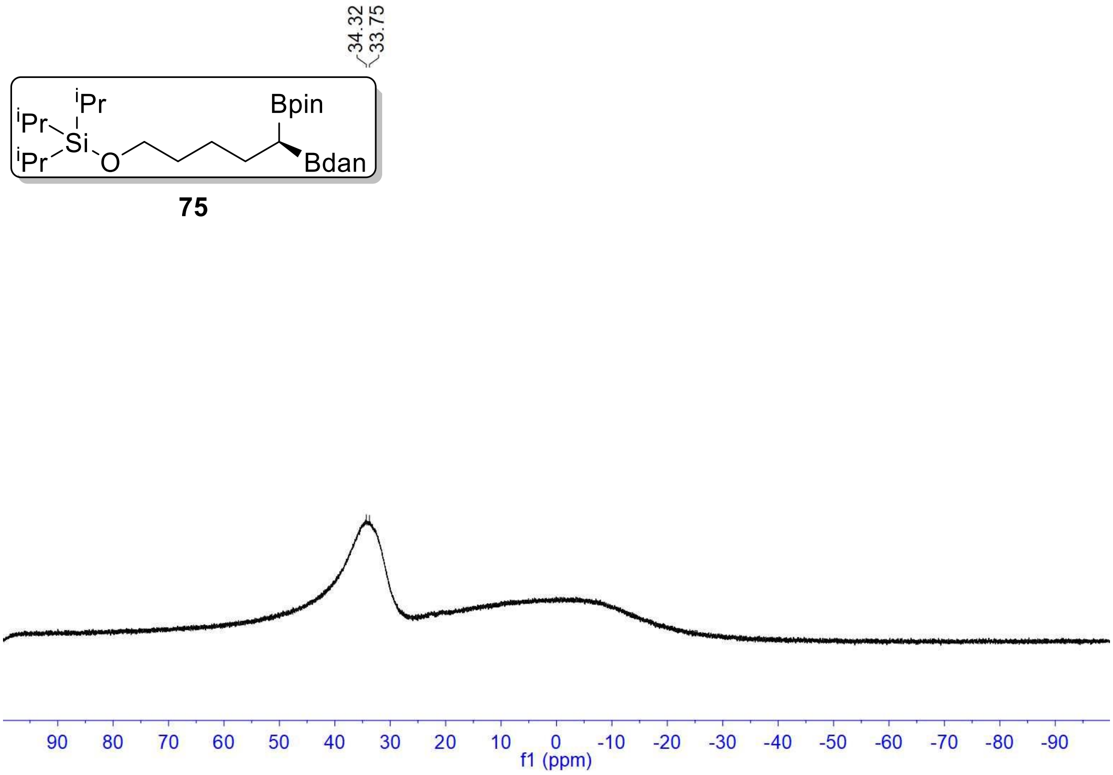

Supplementary Figure 237. ^{13}C NMR spectrum of compound 75

(R)-5-(1H-naphtho[1,8-de][1,3,2]diazaborinin-2(3H)-yl)-5-(4,4,5,5-tetramethyl-1,3,2-dioxaborolan-2-yl)pentyl propionate (76)

^{1}H NMR (500 MHz, room temperature, CDCl$_3$)

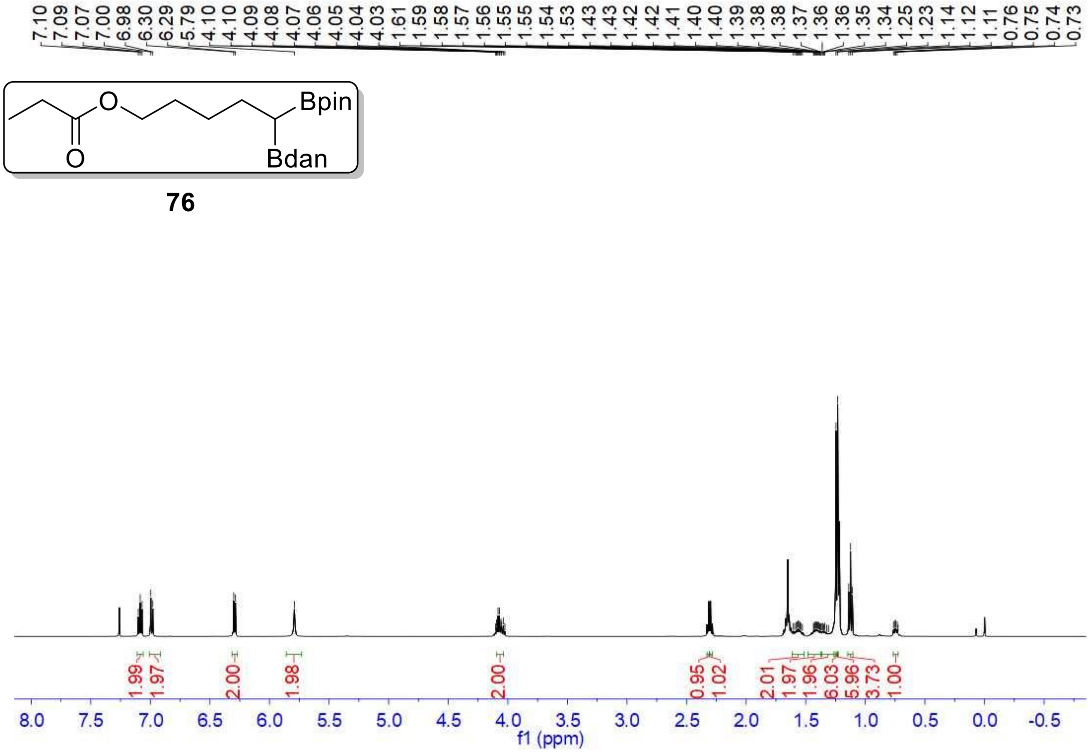

Supplementary Figure 238. ^{1}H NMR spectrum of compound 76

^{13}C NMR (126 MHz, room temperature, $CDCl_3$)

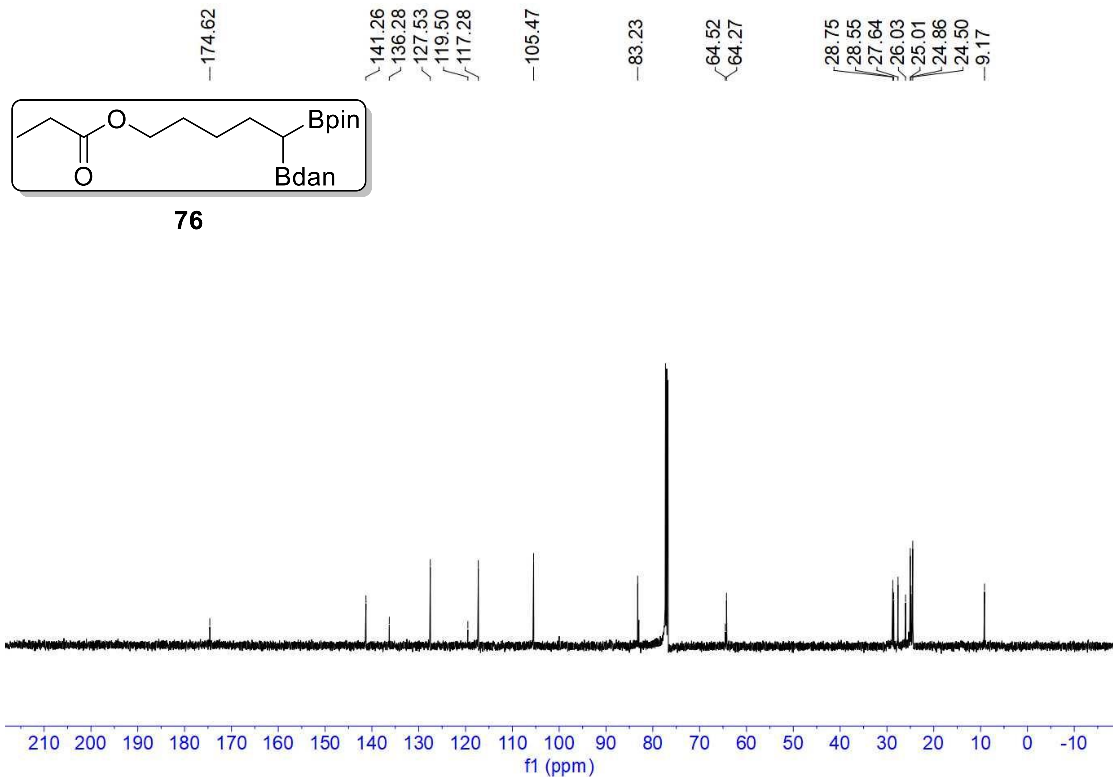

Supplementary Figure 239. ^{13}C NMR spectrum of compound 76

^{11}B NMR (160 MHz, room temperature, $CDCl_3$)

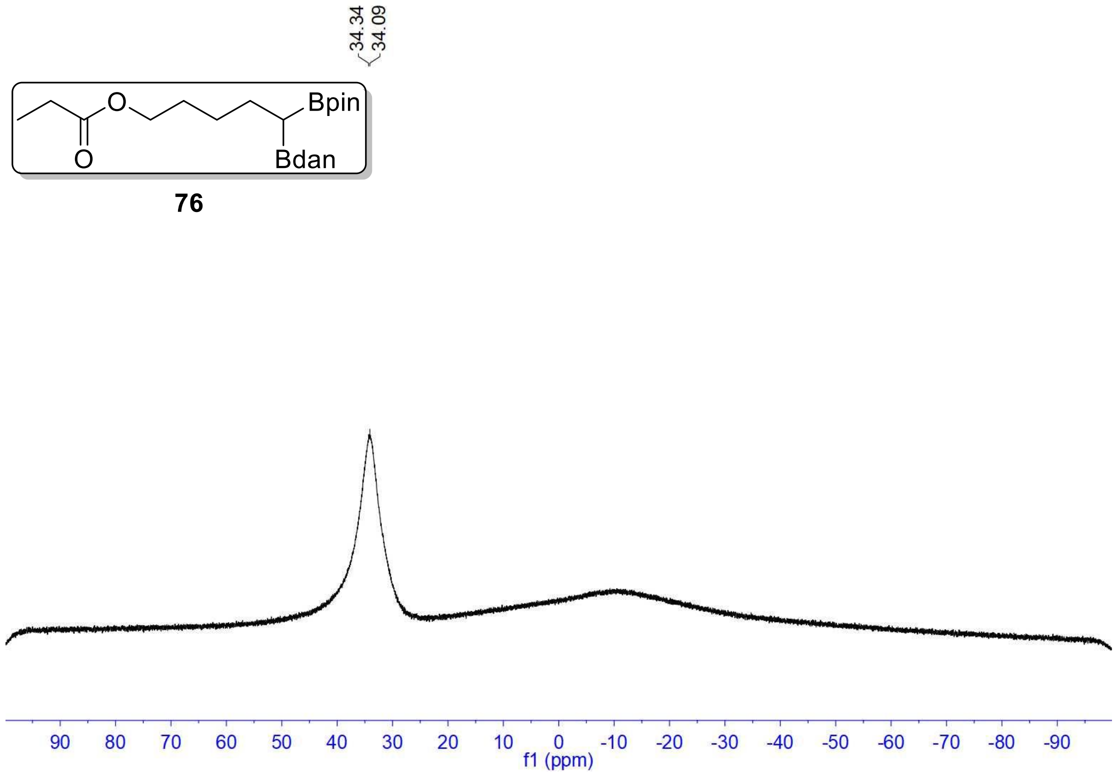

Supplementary Figure 240. ^{11}B NMR spectrum of compound 76

(R)-5-(1H-naphtho[1,8-de][1,3,2]diazaborinin-2(3H)-yl)-5-(4,4,5,5-tetramethyl-1,3,2-dioxaborolan-2-yl)pentyl (2E,4E)-hexa-2,4-dienoate (77)

^{1}H NMR (500 MHz, room temperature, CDCl$_3$)

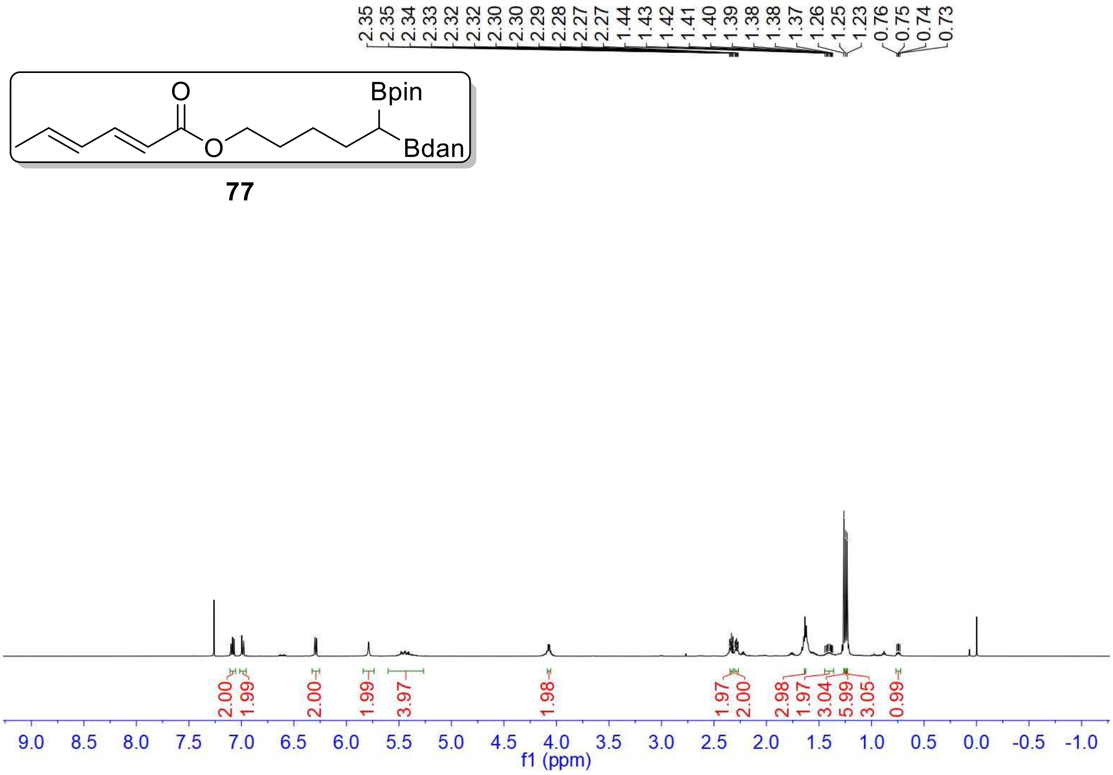

Supplementary Figure 241. ^{1}H NMR spectrum of compound 77

^{13}C NMR (126 MHz, room temperature, CDCl$_3$)

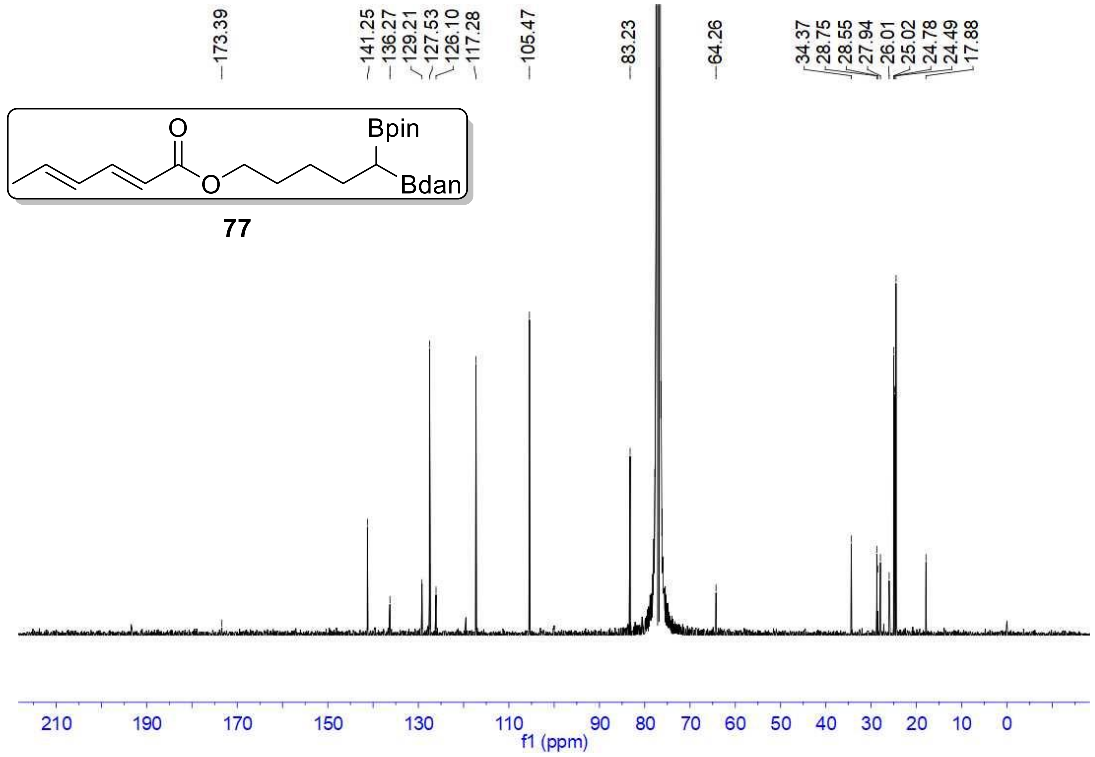

Supplementary Figure 242. ^{13}C NMR spectrum of compound 77

^{11}B NMR (160 MHz, room temperature, $CDCl_3$)

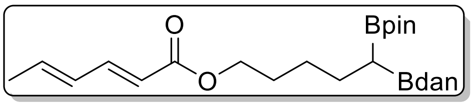

77

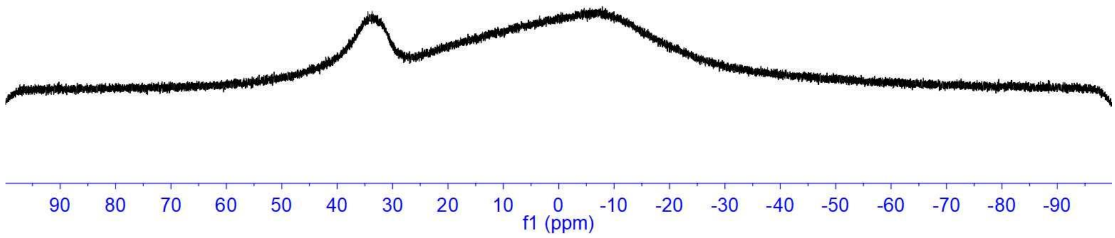

Supplementary Figure 243. ^{11}**B NMR spectrum of compound 77**

(R)-5-(1H-naphtho[1,8-de][1,3,2]diazaborinin-2(3H)-yl)-5-(4,4,5,5-tetramethyl-1,3,2-dioxaborolan-2-yl)pentyl 2-propylpentanoate (78)

^{1}H NMR (500 MHz, room temperature, $CDCl_3$)

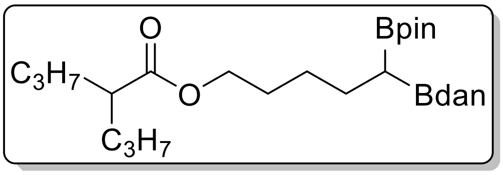

78

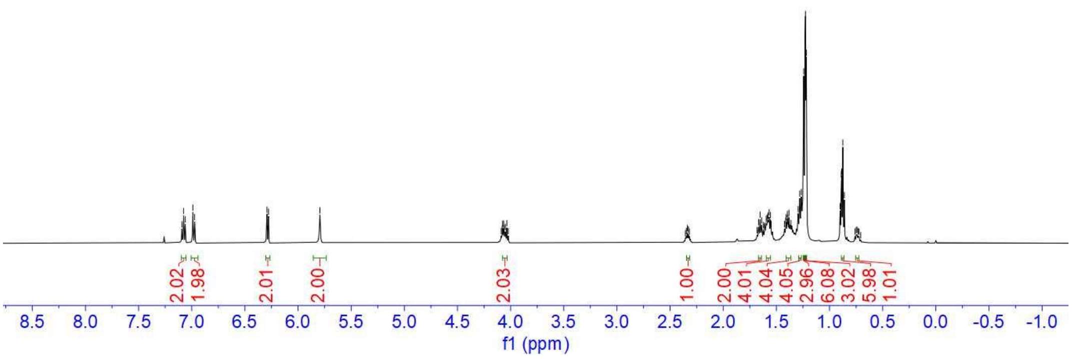

Supplementary Figure 244. ^{1}**H NMR spectrum of compound 78**

^{13}C NMR (126 MHz, room temperature, $CDCl_3$)

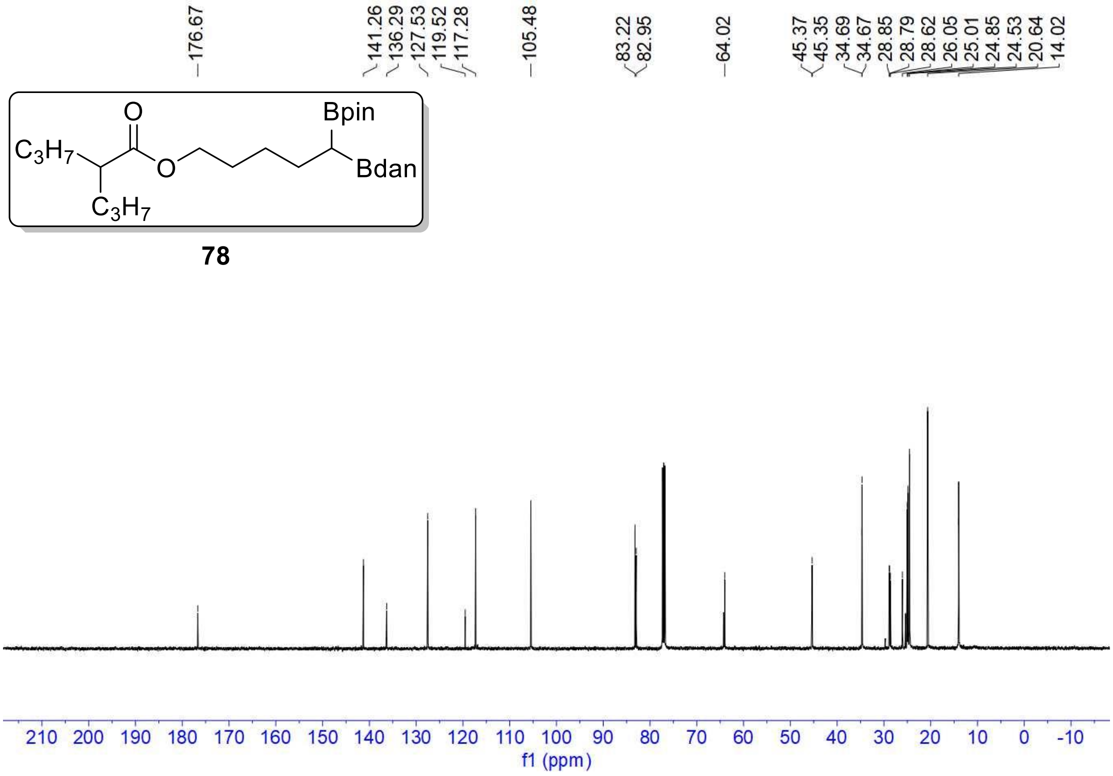

Supplementary Figure 245. ^{13}C **NMR spectrum of compound 78**

^{11}B NMR (160 MHz, room temperature, $CDCl_3$)

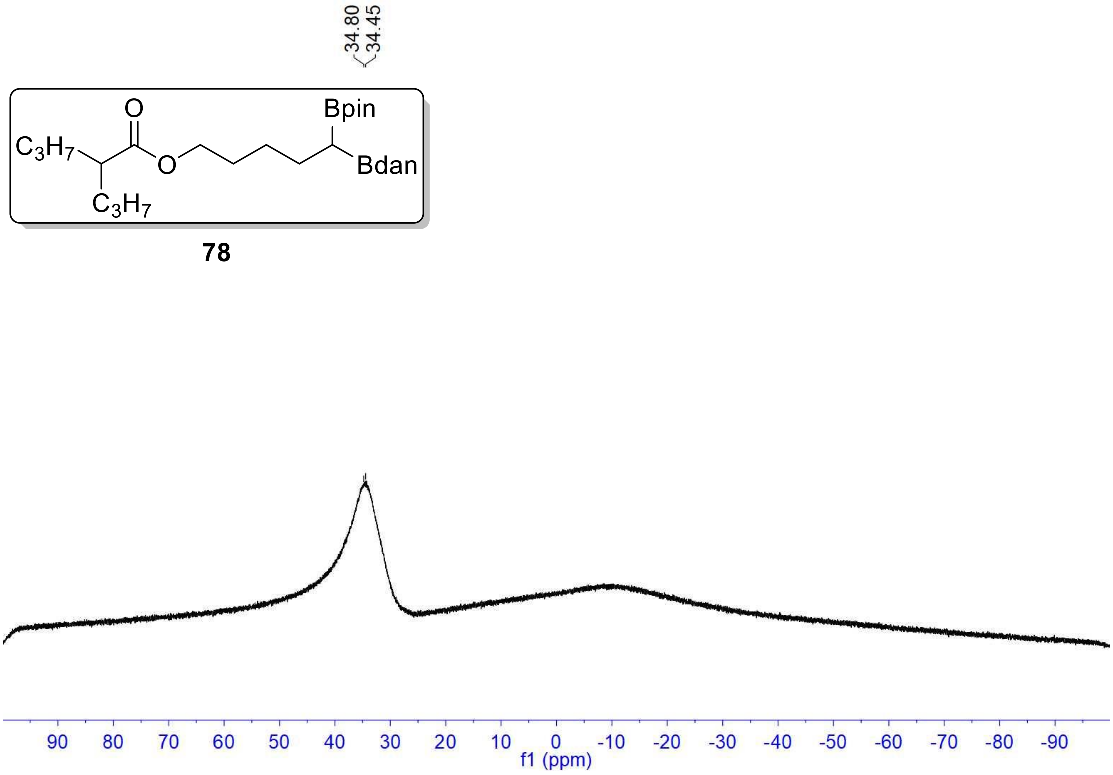

Supplementary Figure 246. ^{11}B **NMR spectrum of compound 78**

(R)-5-(1H-naphtho[1,8-de][1,3,2]diazaborinin-2(3H)-yl)-5-(4,4,5,5-tetramethyl-1,3,2-dioxaborolan-2-yl)pentyl benzoate (79)

^{1}H NMR (500 MHz, room temperature, CDCl$_3$)

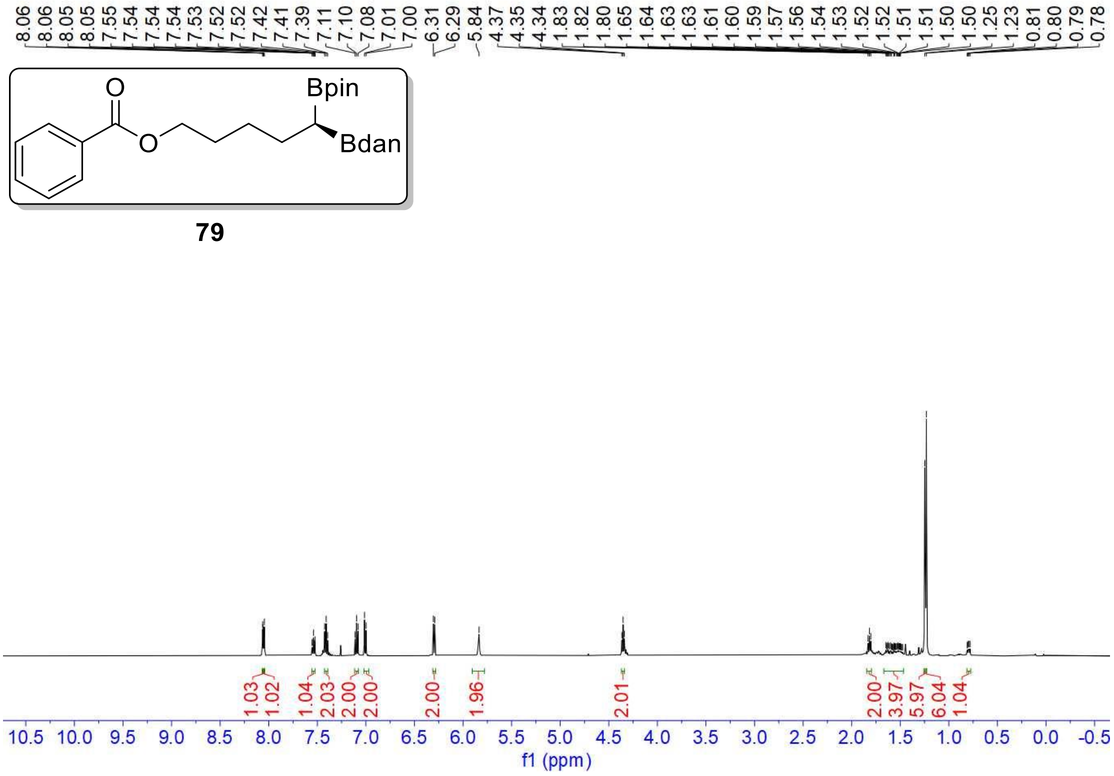

Supplementary Figure 247. ^{1}H NMR spectrum of compound 79

^{13}C NMR (126 MHz, room temperature, CDCl$_3$)

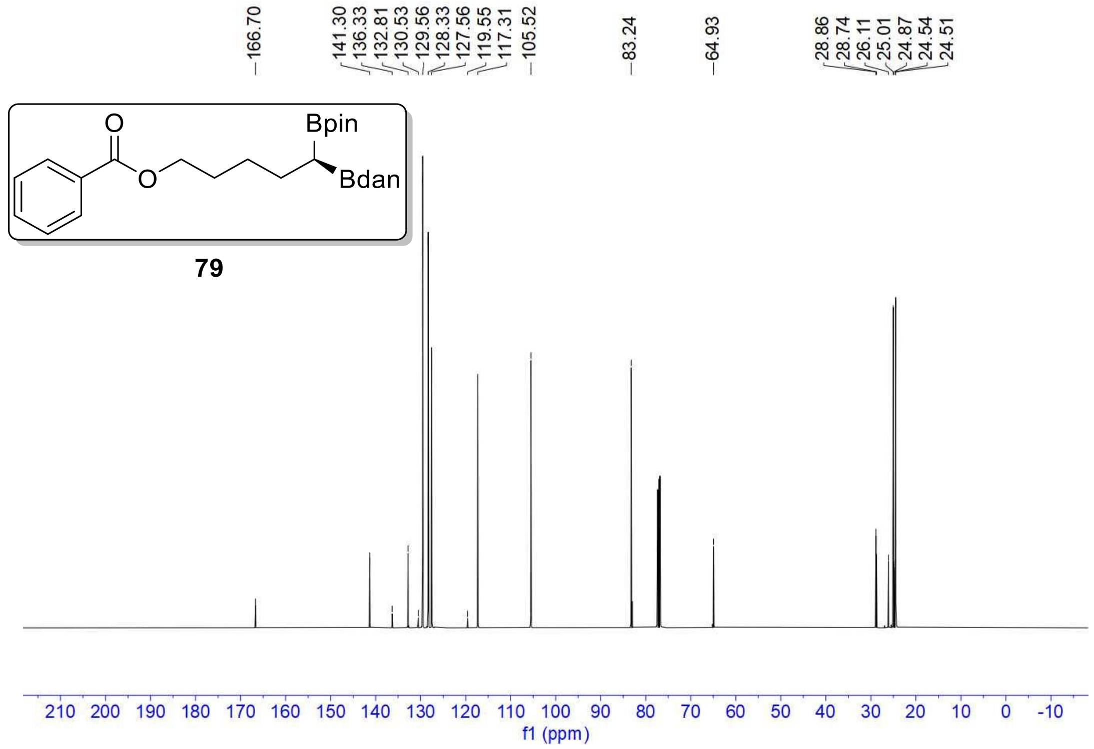

Supplementary Figure 248. ^{13}C NMR spectrum of compound 79

^{11}B NMR (160 MHz, room temperature, CDCl$_3$)

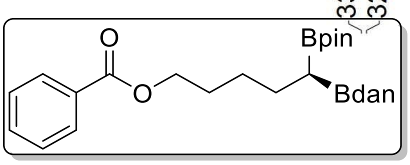

79

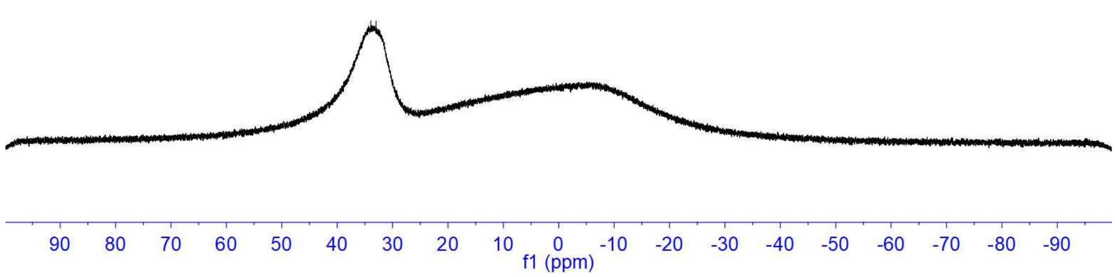

Supplementary Figure 249. ^{11}B NMR spectrum of compound 79

(R)-5-(1H-naphtho[1,8-de][1,3,2]diazaborinin-2(3H)-yl)-5-(4,4,5,5-tetramethyl-1,3,2-dioxaborolan-2-yl)pentyl 4-methylbenzoate (80)

^{1}H NMR (500 MHz, room temperature, CDCl$_3$)

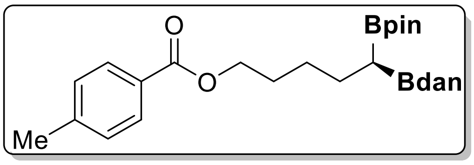

80

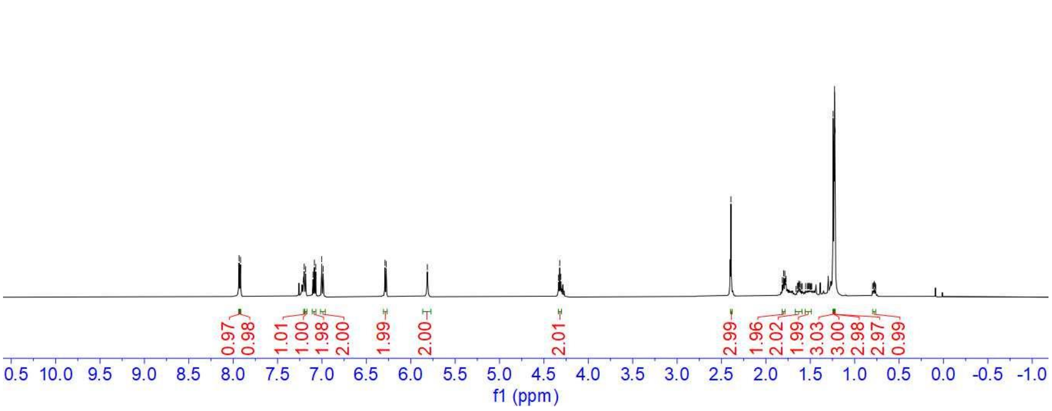

Supplementary Figure 250. ^{1}H NMR spectrum of compound 80

^{13}C NMR (126 MHz, room temperature, $CDCl_3$)

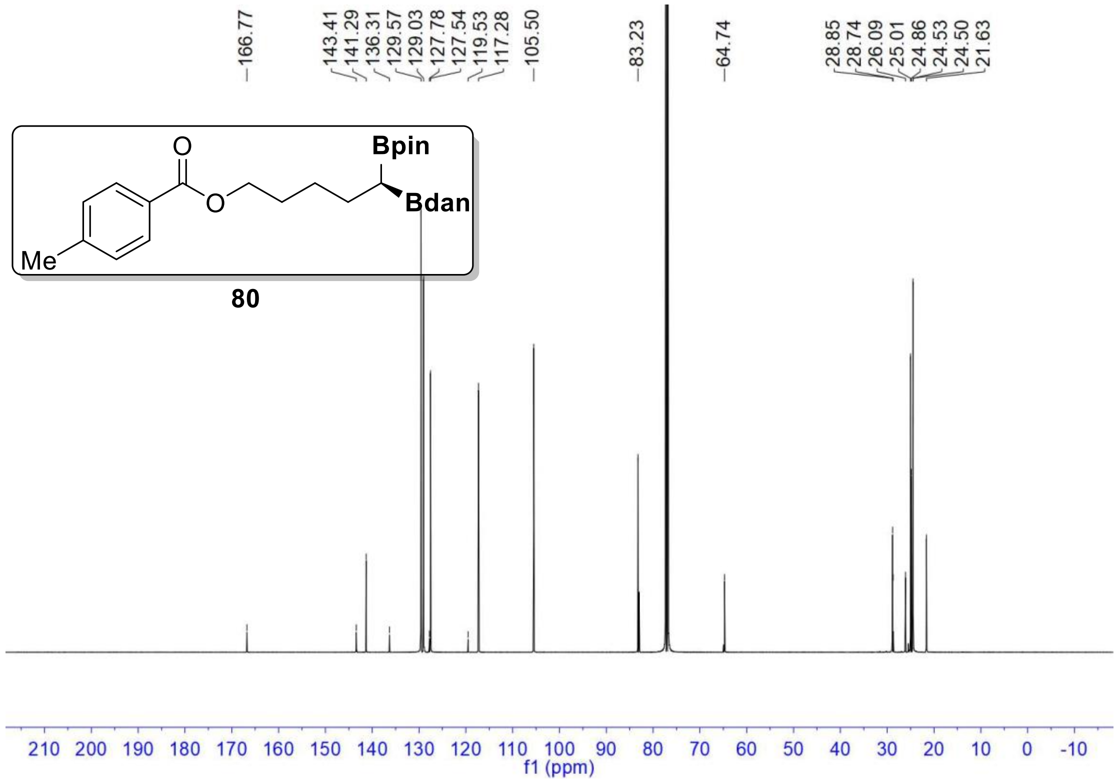

Supplementary Figure 251. ^{13}C NMR spectrum of compound 80

^{11}B NMR (160 MHz, room temperature, $CDCl_3$)

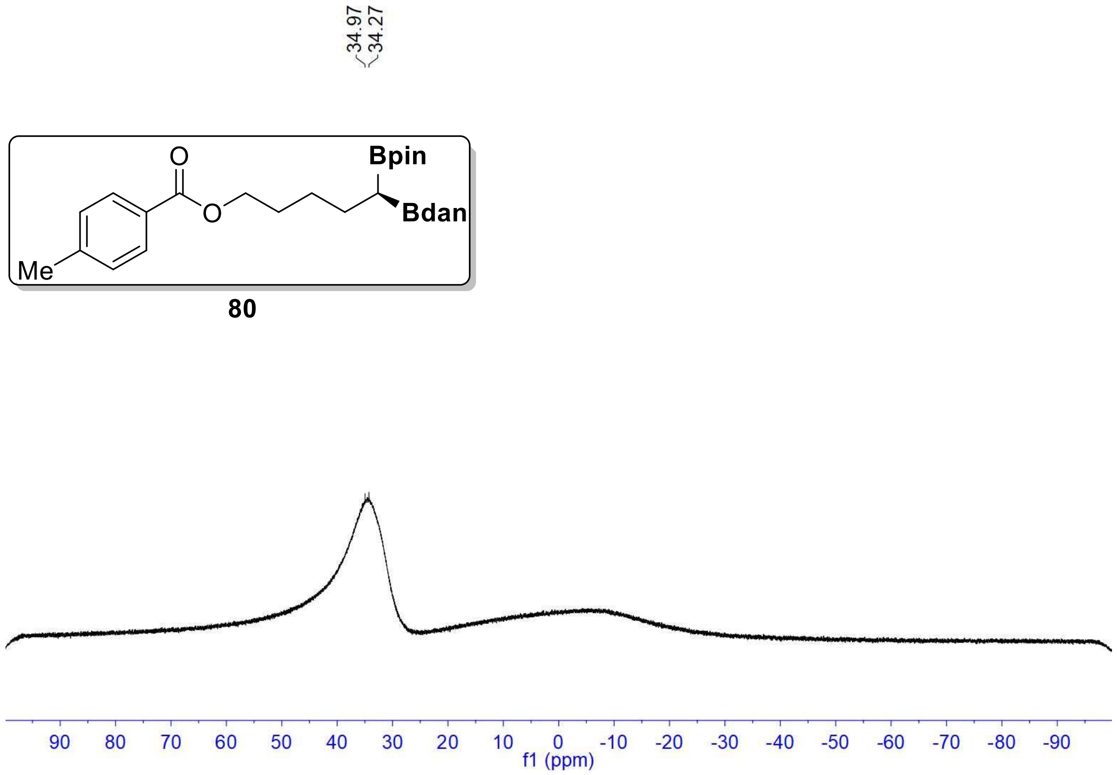

Supplementary Figure 252. ^{11}B NMR spectrum of compound 80

(R)-5-(1H-naphtho[1,8-de][1,3,2]diazaborinin-2(3H)-yl)-5-(4,4,5,5-tetramethyl-1,3,2-dioxaborolan-2-yl)pentyl 4-cyclohexylbenzoate (81)

^{1}H NMR (500 MHz, room temperature, CDCl$_3$)

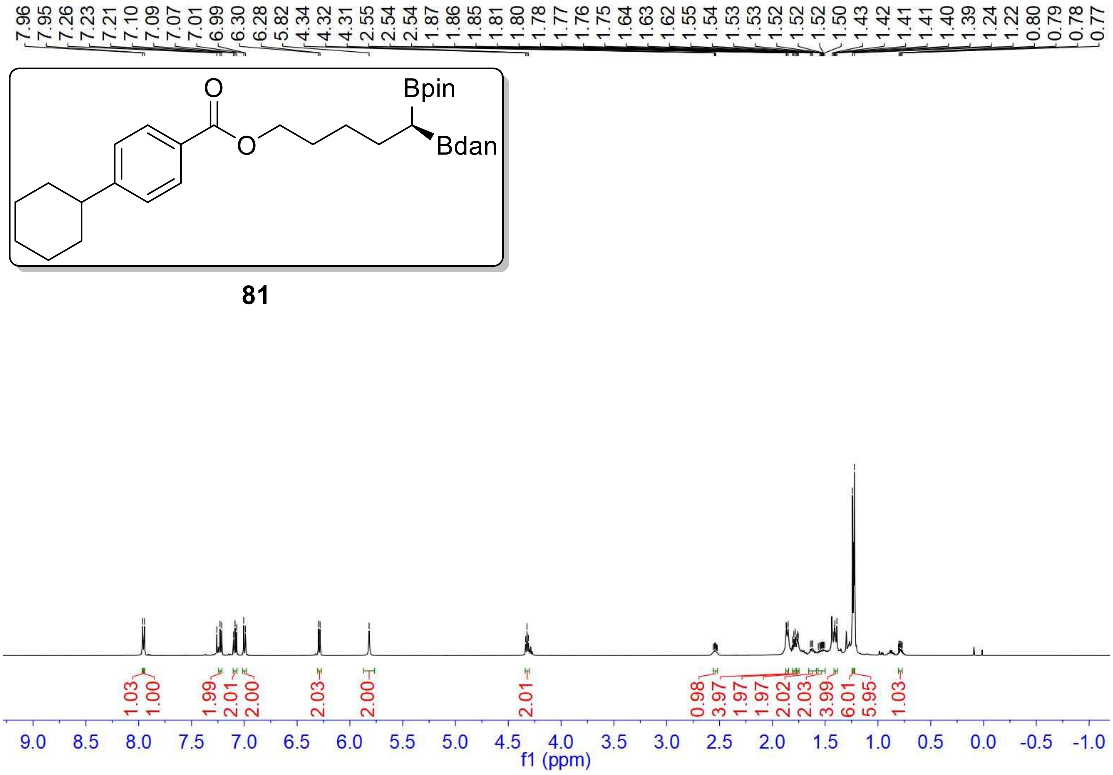

Supplementary Figure 253. ^{1}H NMR spectrum of compound 81

^{13}C NMR (126 MHz, room temperature, CDCl$_3$)

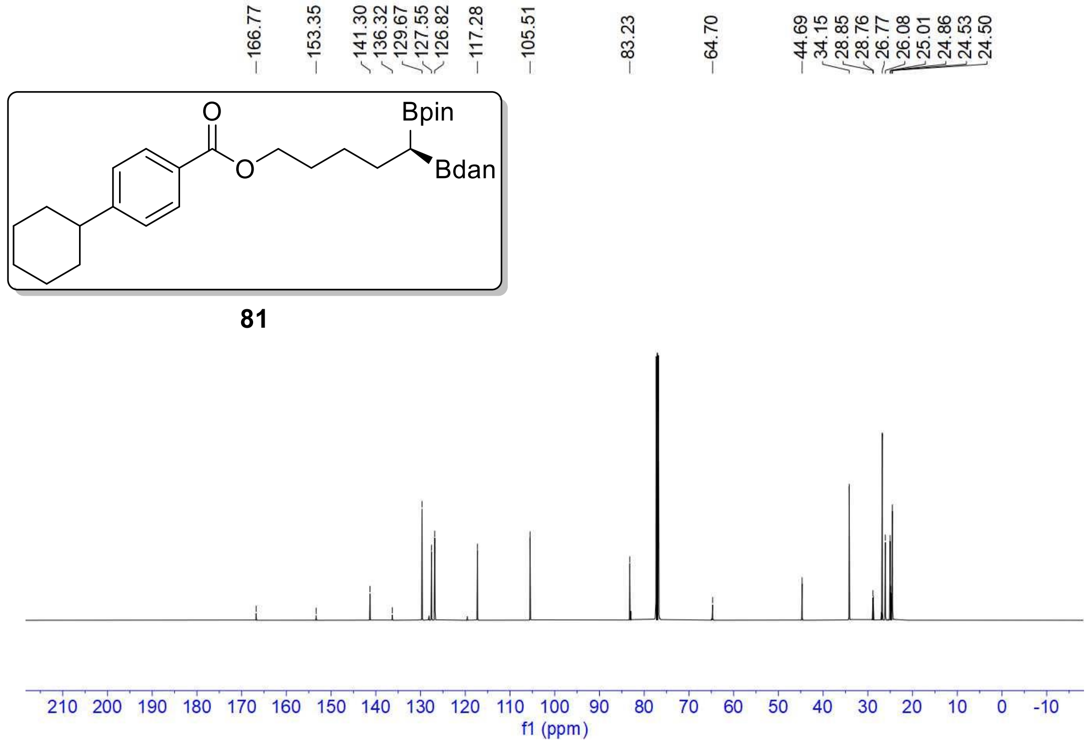

Supplementary Figure 254. ^{13}C NMR spectrum of compound 81

^{11}B NMR (160 MHz, room temperature, $CDCl_3$)

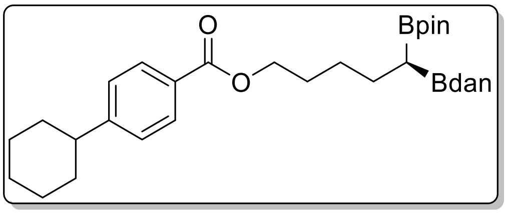

81

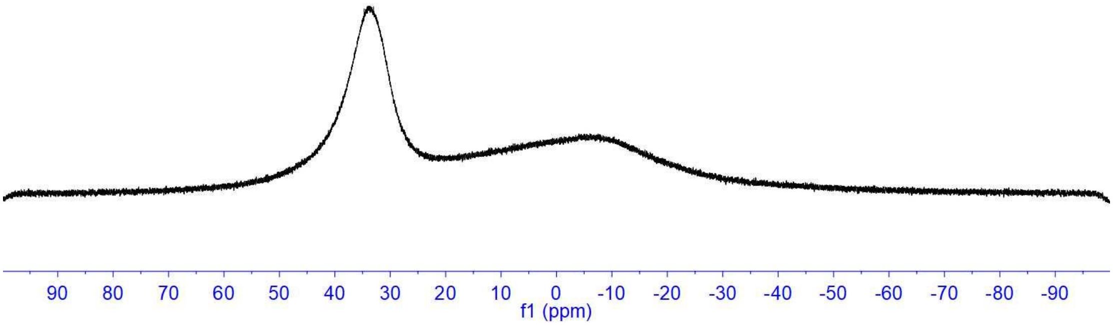

Supplementary Figure 255. ^{11}B NMR spectrum of compound 81

(R)-5-(1H-naphtho[1,8-de][1,3,2]diazaborinin-2(3H)-yl)-5-(4,4,5,5-tetramethyl-1,3,2-dioxaborolan-2-yl)pentyl (E)-2-methyl-3-phenylacrylate (82)

^{1}H NMR (500 MHz, room temperature, $CDCl_3$)

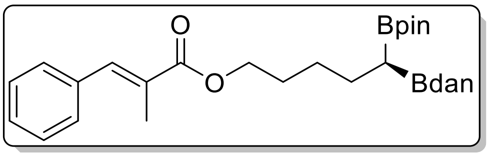

82

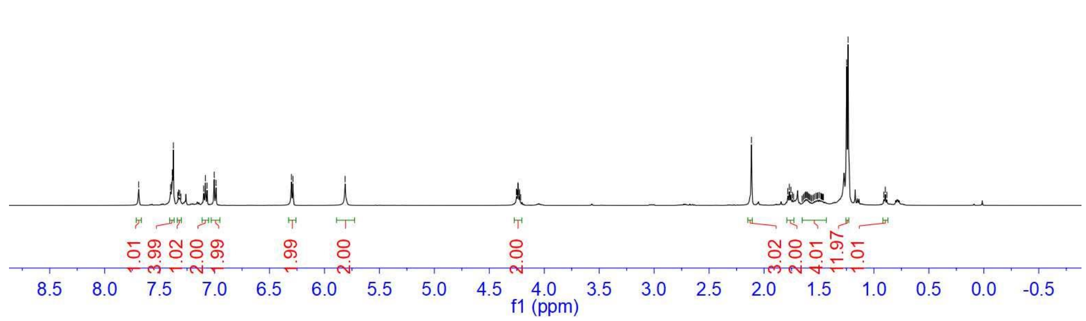

Supplementary Figure 256. ^{1}H NMR spectrum of compound 82

^{13}C NMR (126 MHz, room temperature, $CDCl_3$)

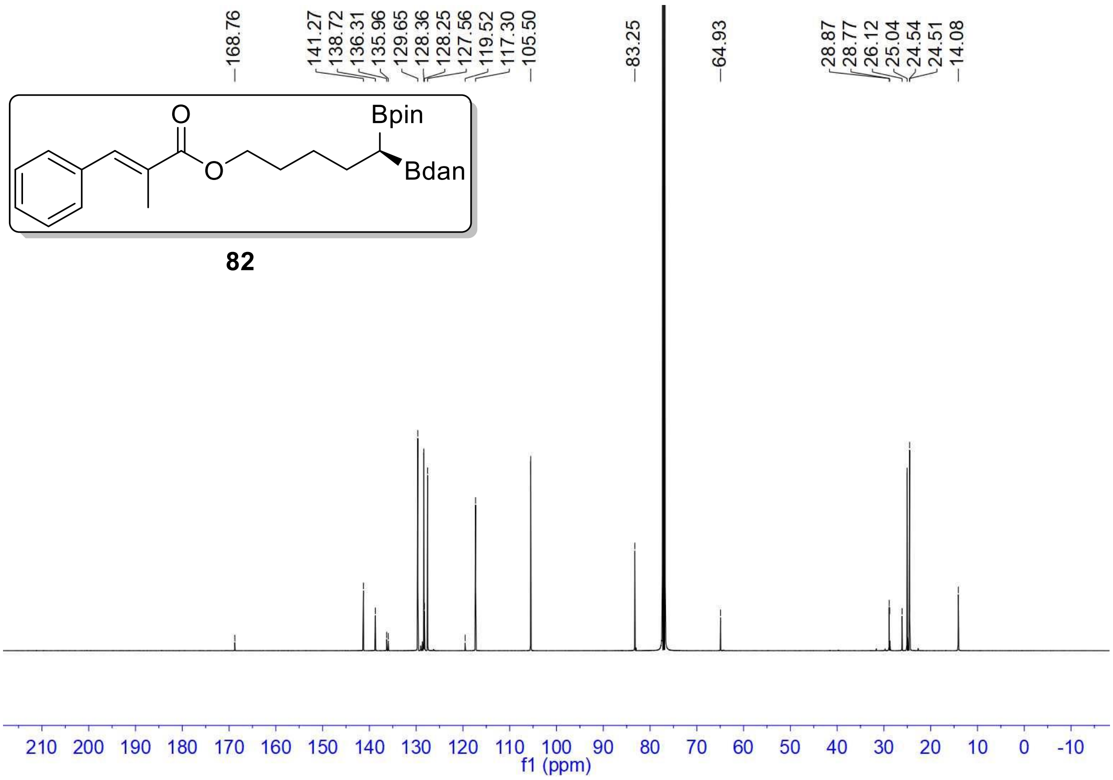

Supplementary Figure 257. ^{13}C NMR spectrum of compound 82

^{11}B NMR (160 MHz, room temperature, $CDCl_3$)

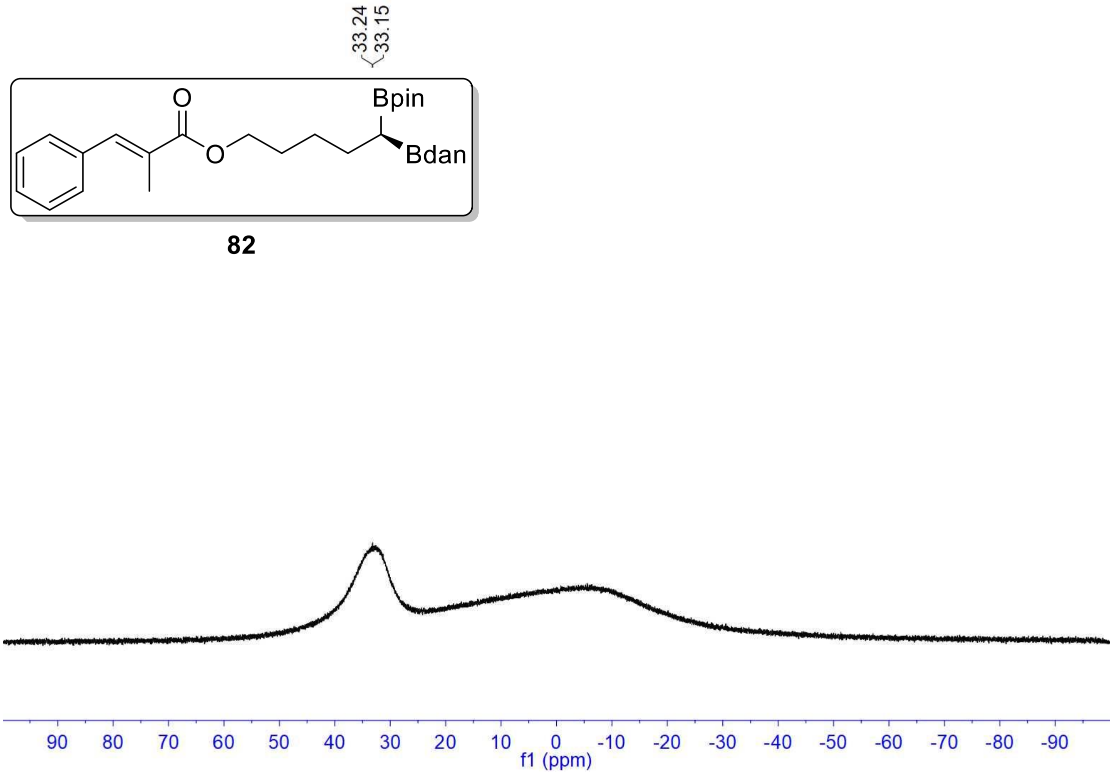

Supplementary Figure 258. ^{11}B NMR spectrum of compound 82

(R)-5-(1H-naphtho[1,8-de][1,3,2]diazaborinin-2(3H)-yl)-5-(4,4,5,5-tetramethyl-1,3,2-dioxaborolan-2-yl)pentyl (2R)-2-phenylpropanoate (83)

^{1}H NMR (500 MHz, room temperature, CDCl$_3$)

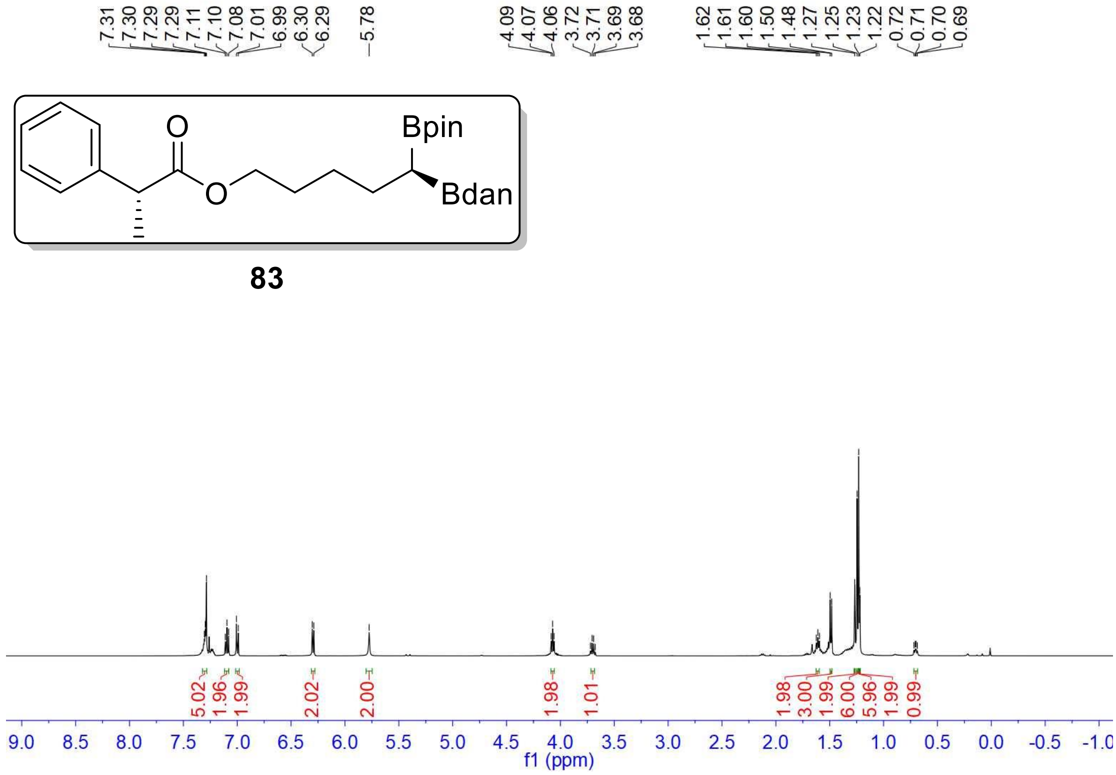

Supplementary Figure 259. ^{1}H NMR spectrum of compound 83

^{13}C NMR (126 MHz, room temperature, CDCl$_3$)

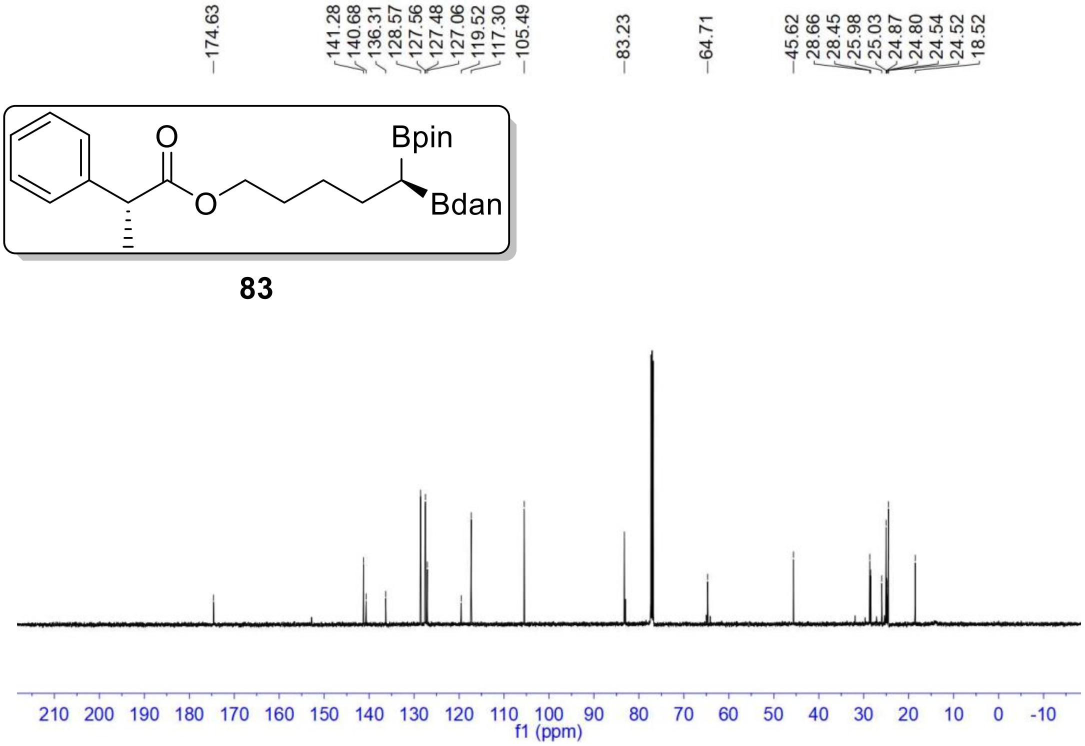

Supplementary Figure 260. ^{13}C NMR spectrum of compound 83

^{11}B NMR (160 MHz, room temperature, $CDCl_3$)

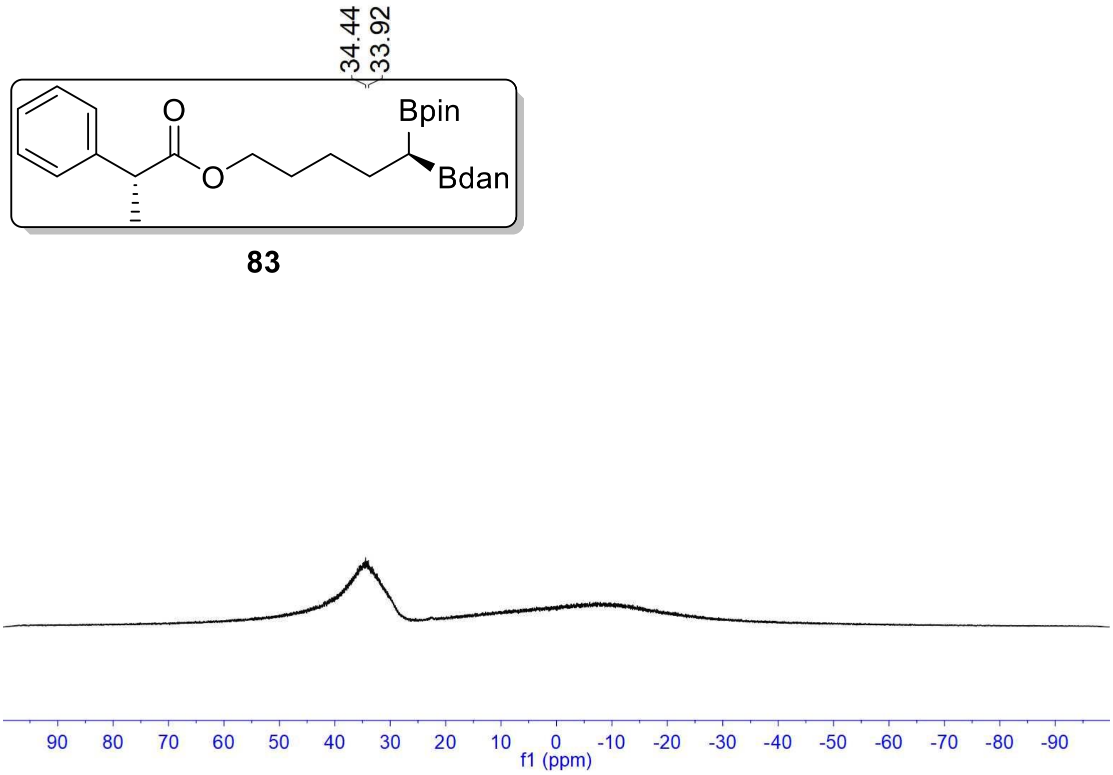

83

Supplementary Figure 261. ^{11}B NMR spectrum of compound 83

(R)-5-(1H-naphtho[1,8-de][1,3,2]diazaborinin-2(3H)-yl)-5-(4,4,5,5-tetramethyl-1,3,2-dioxaborolan-2-yl)pentyl (2S)-2-(4-isobutylphenyl)propanoate (84)

^{1}H NMR (500 MHz, room temperature, $CDCl_3$)

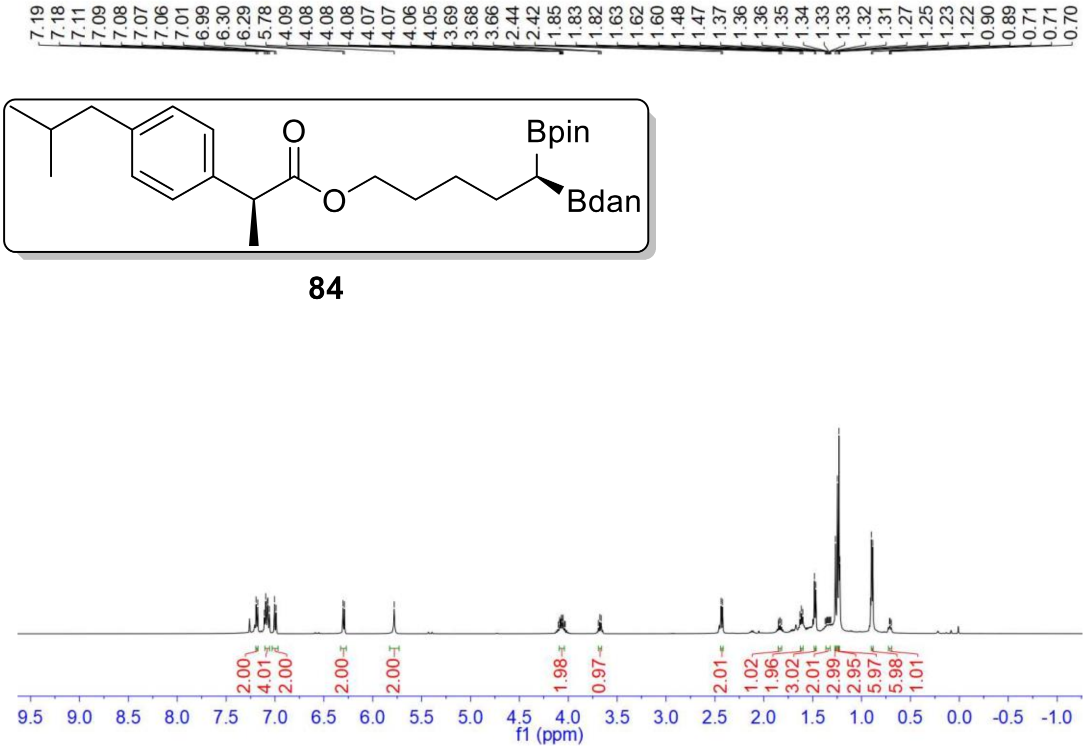

84

Supplementary Figure 262. ^{1}H NMR spectrum of compound 84

^{13}C NMR (126 MHz, room temperature, $CDCl_3$)

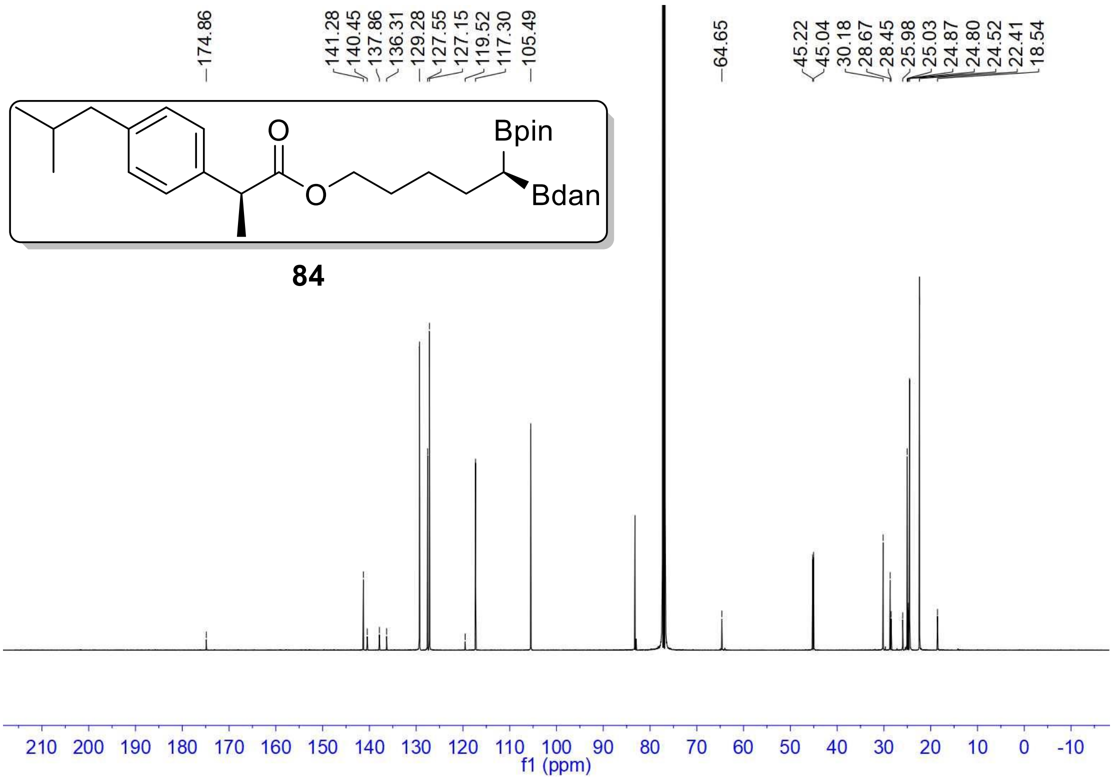

Supplementary Figure 263. ^{13}C NMR spectrum of compound 84

^{11}B NMR (160 MHz, room temperature, $CDCl_3$)

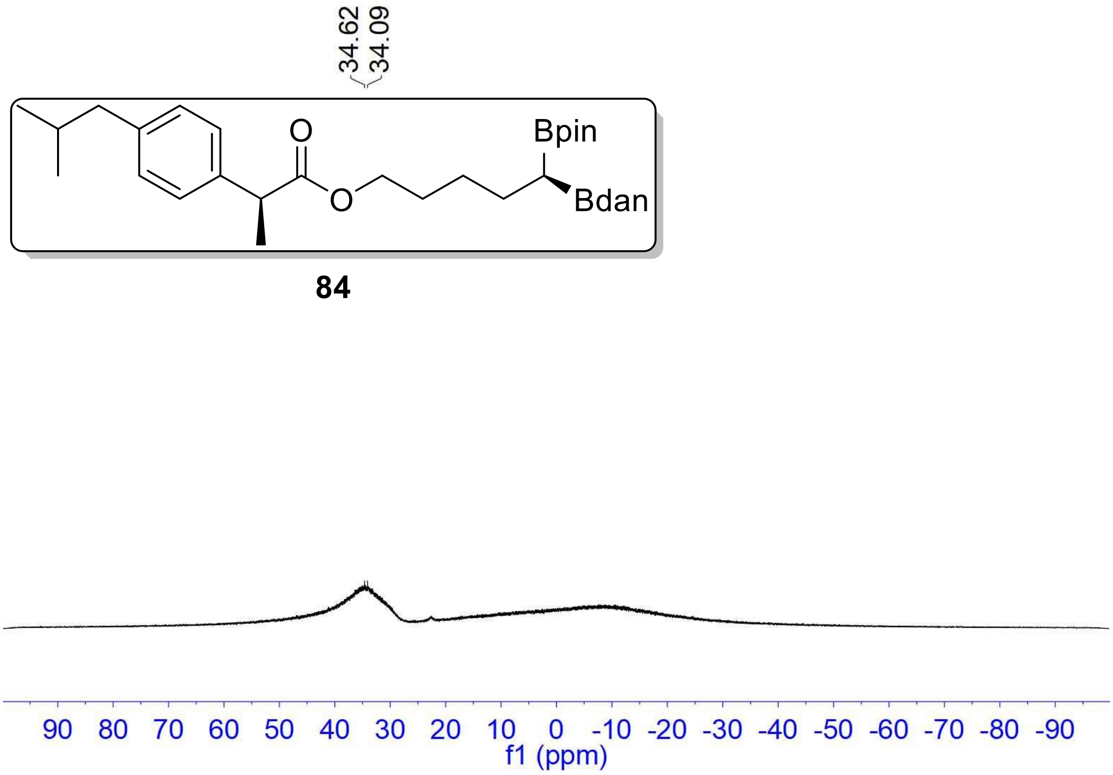

Supplementary Figure 264. ^{11}B NMR spectrum of compound 84

(R)-5-(1H-naphtho[1,8-de][1,3,2]diazaborinin-2(3H)-yl)-5-(4,4,5,5-tetramethyl-1,3,2-dioxaborolan-2-yl)pentyl (2R)-2-(6-methoxynaphthalen-2-yl)propanoate (85)

^{1}H NMR (500 MHz, room temperature, $CDCl_3$)

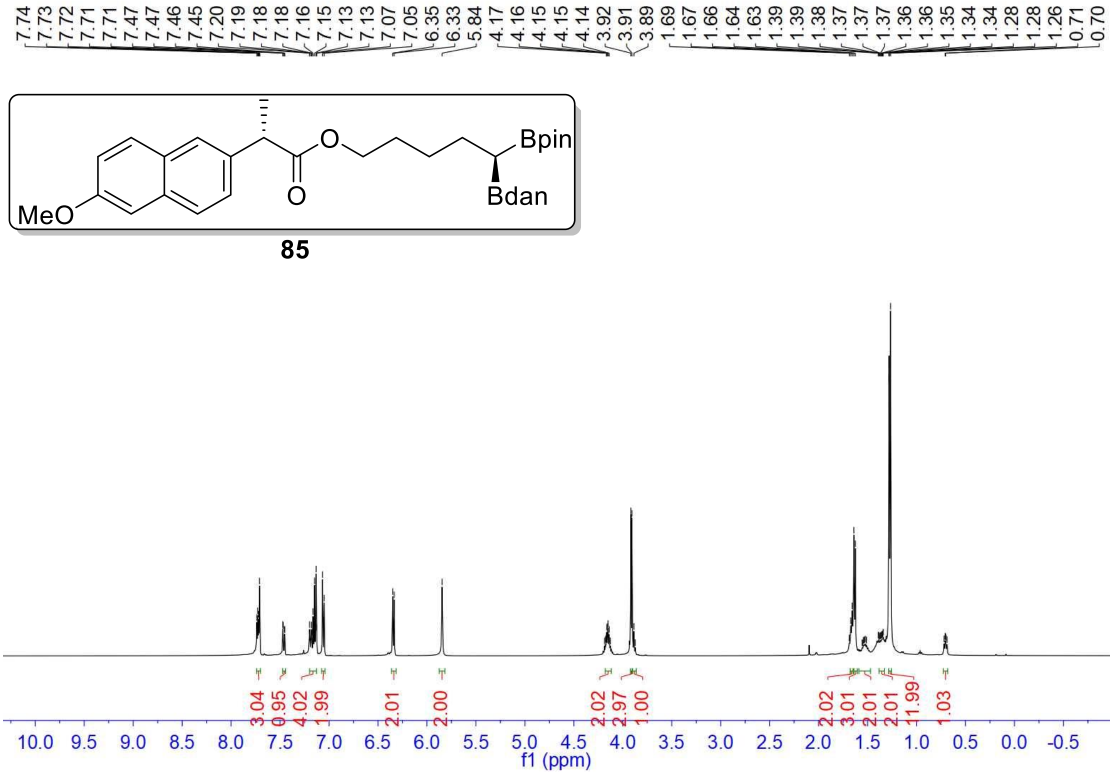

Supplementary Figure 265. ^{1}H NMR spectrum of compound 85

^{13}C NMR (126 MHz, room temperature, $CDCl_3$)

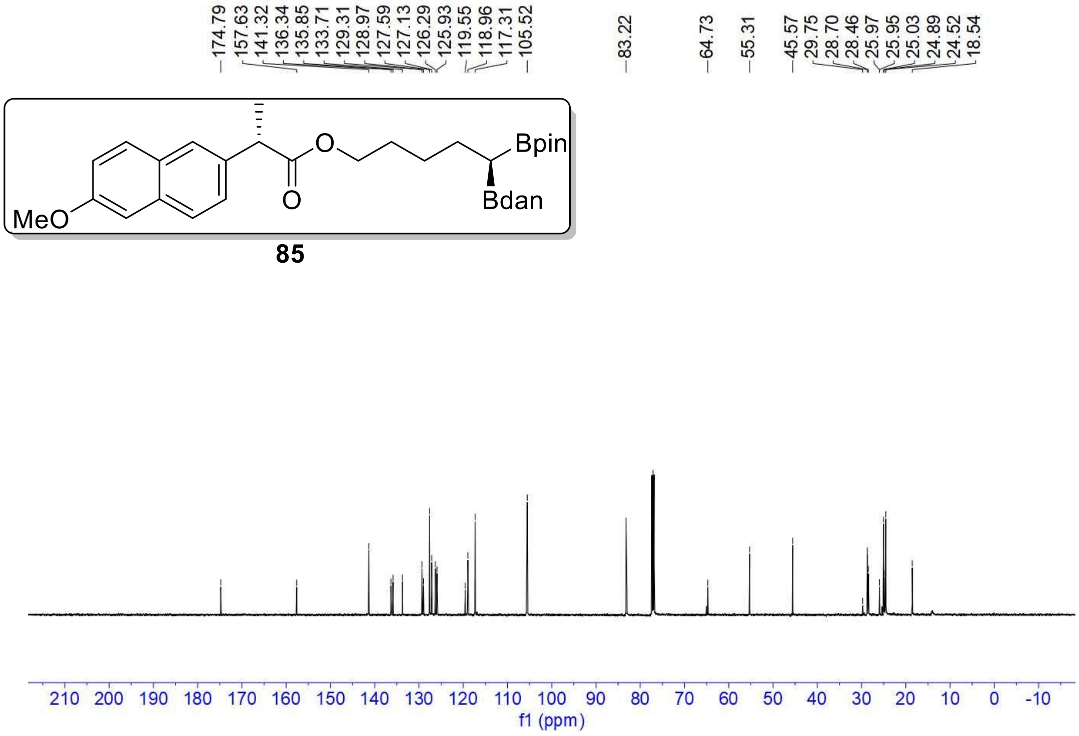

Supplementary Figure 266. ^{13}C NMR spectrum of compound 85

^{11}B NMR (160 MHz, room temperature, $CDCl_3$)

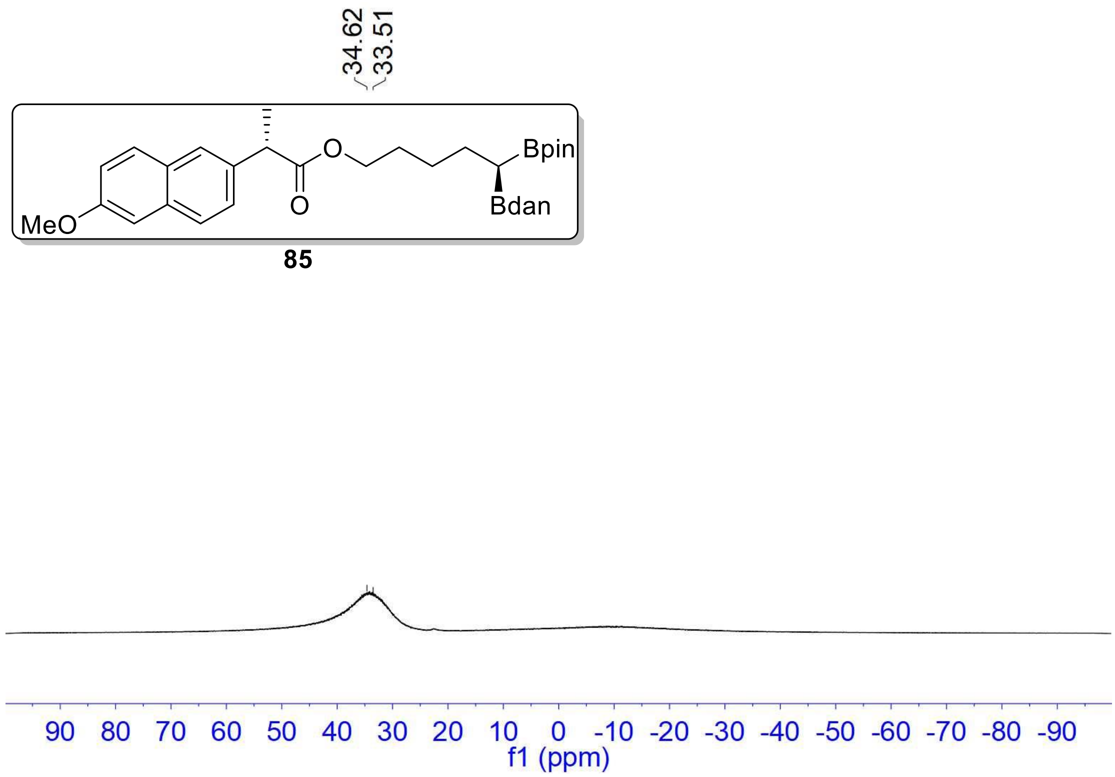

Supplementary Figure 267. ^{11}B NMR spectrum of compound 85

(R)-5-(1H-naphtho[1,8-de][1,3,2]diazaborinin-2(3H)-yl)-5-(4,4,5,5-tetramethyl-1,3,2-dioxaborolan-2-yl)pentyl (1R,2S,4R)-bicyclo[2.2.1]hept-5-ene-2-carboxylate (86)

^{1}H NMR (500 MHz, room temperature, $CDCl_3$)

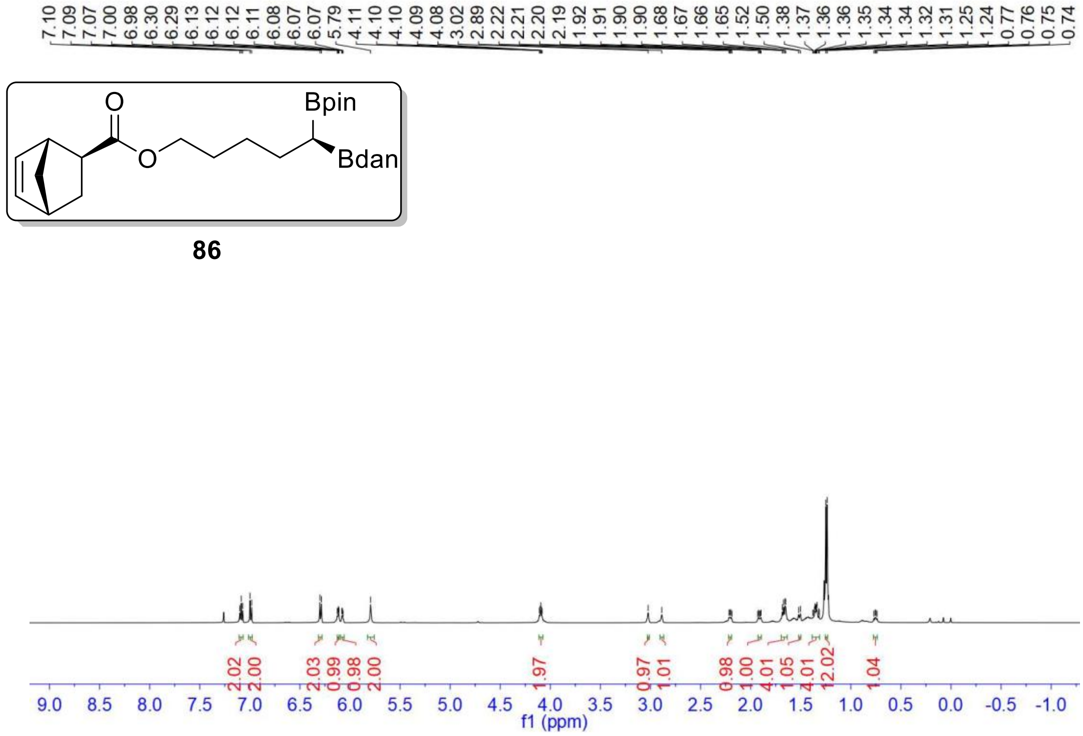

Supplementary Figure 268. ^{1}H NMR spectrum of compound 86

^{13}C NMR (126 MHz, room temperature, $CDCl_3$)

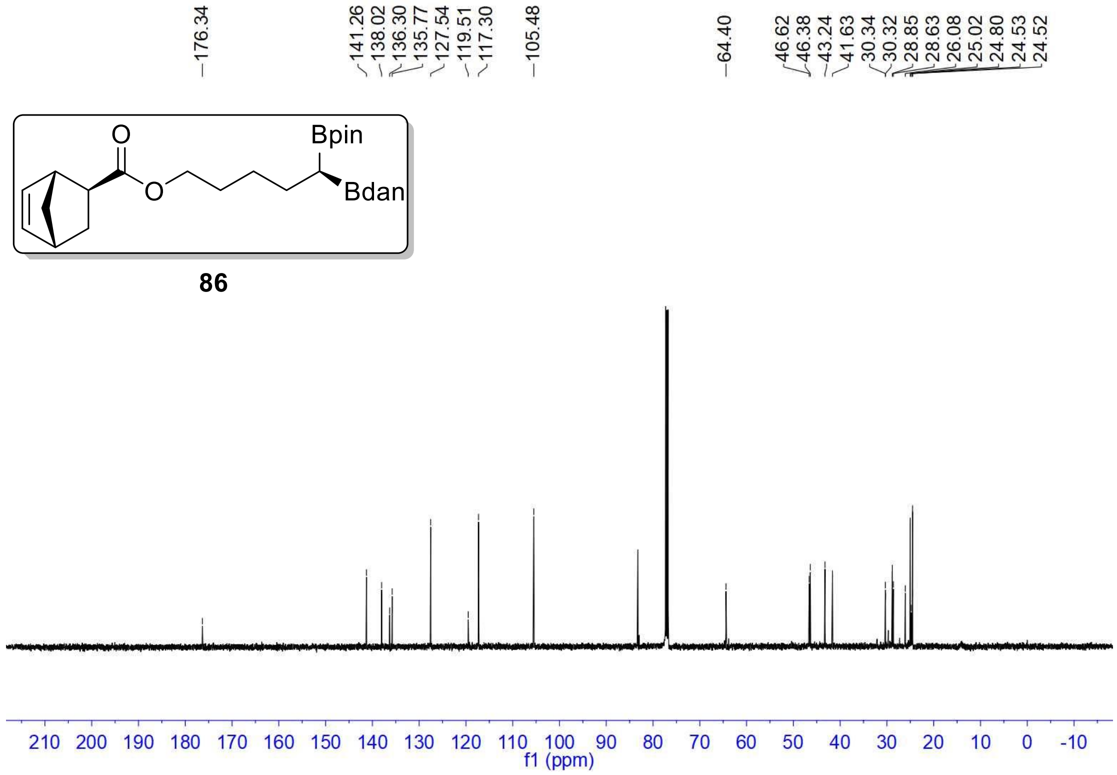

Supplementary Figure 269. ^{13}C NMR spectrum of compound 86

^{11}B NMR (160 MHz, room temperature, $CDCl_3$)

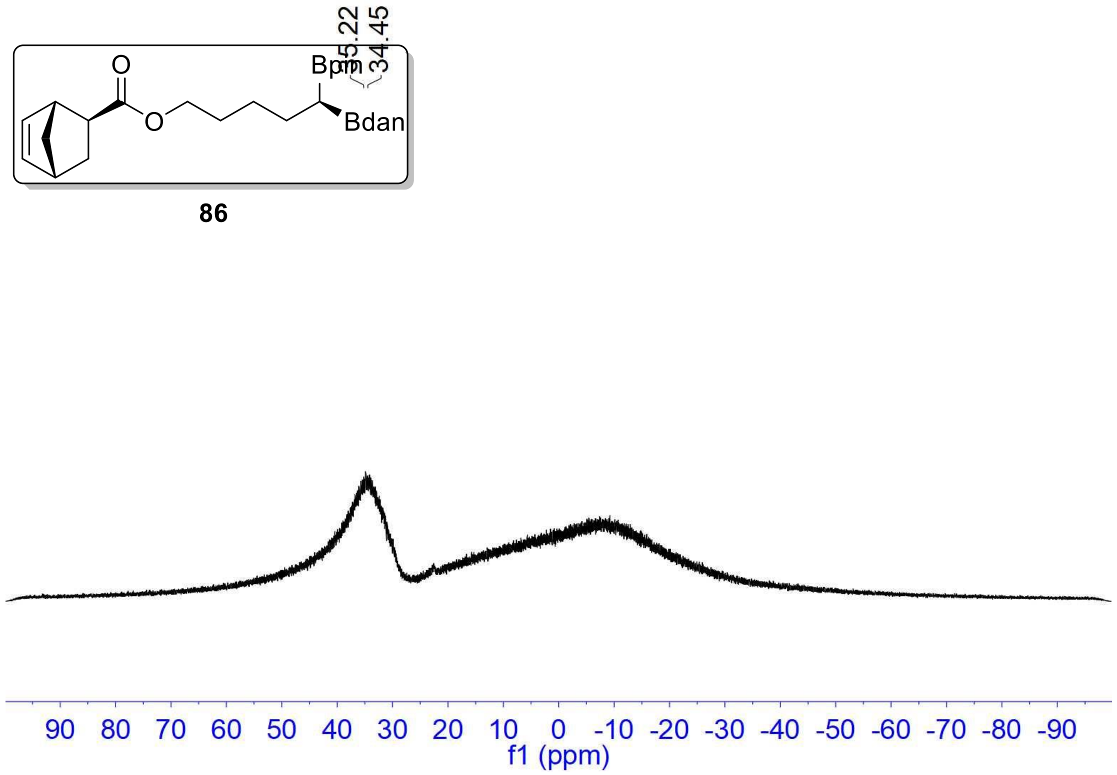

Supplementary Figure 270. ^{11}B NMR spectrum of compound 86

(R)-2-(2-phenyl-1-(4,4,5,5-tetramethyl-1,3,2-dioxaborolan-2-yl)ethyl)-2,3-dihydro-1H-naphtho[1,8-de][1,3,2]diazaborinine (87[a])

^{1}H NMR (500 MHz, room temperature, $CDCl_3$)

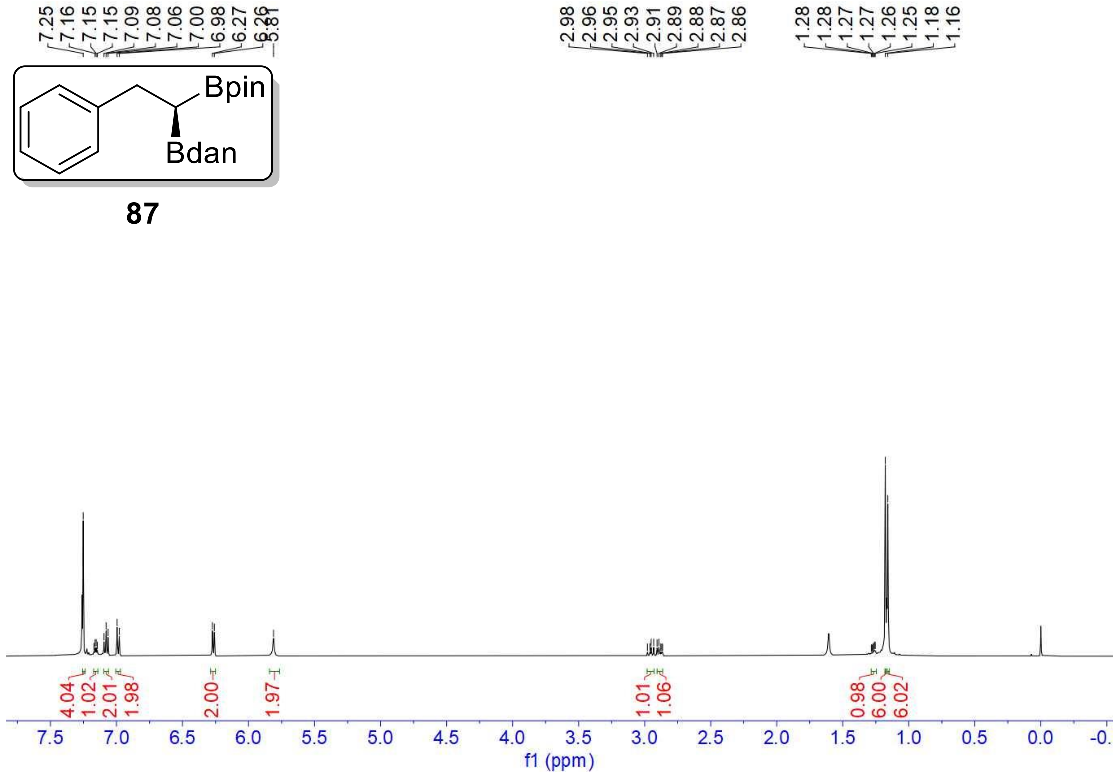

Supplementary Figure 271. ^{1}H NMR spectrum of compound 87[a]

^{13}C NMR (126 MHz, room temperature, $CDCl_3$)

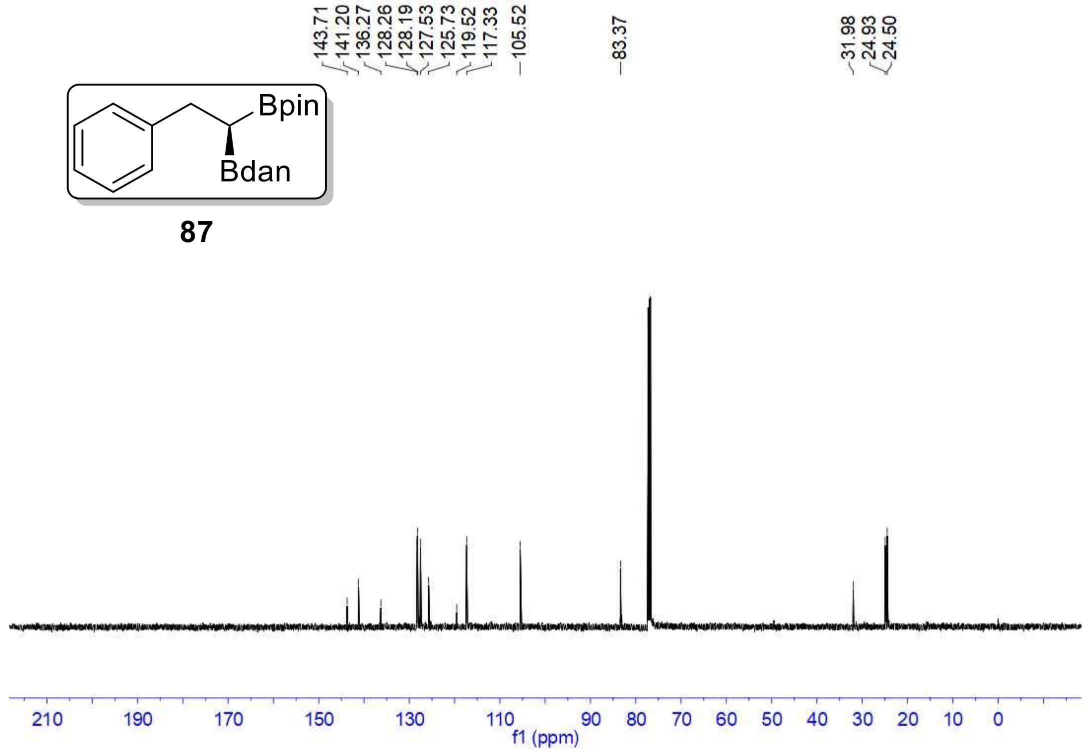

Supplementary Figure 272. ^{13}C NMR spectrum of compound 87[a]

^{11}B NMR (160 MHz, room temperature, $CDCl_3$)

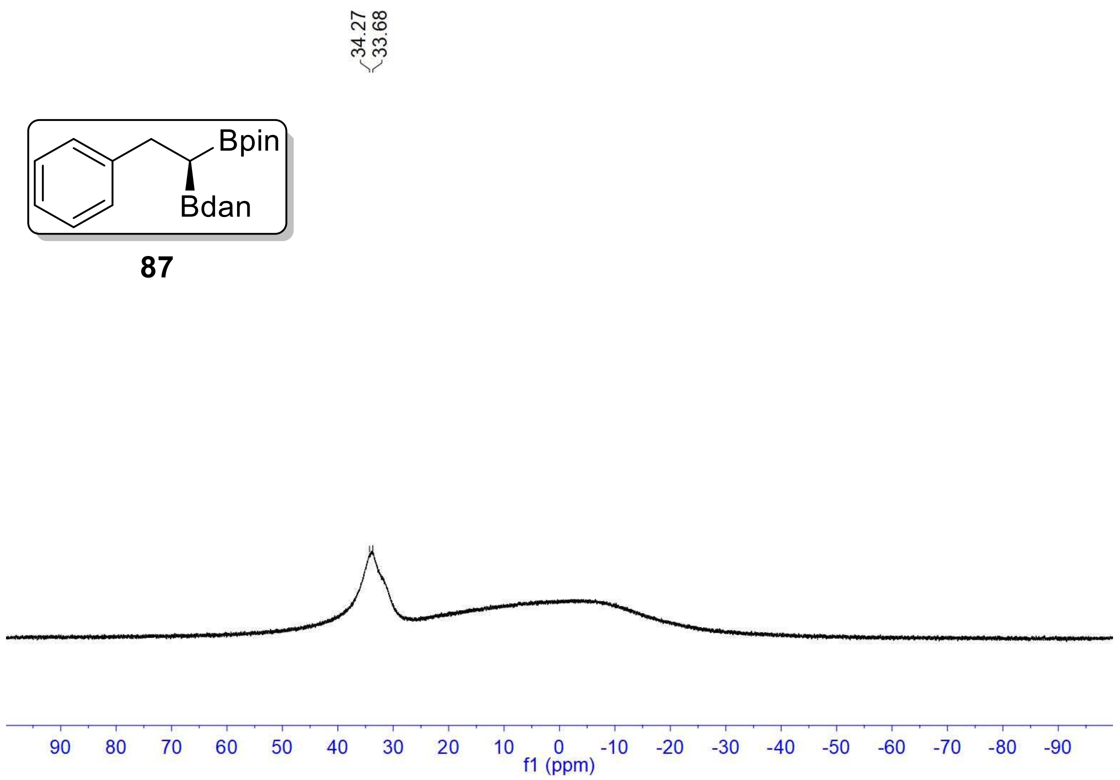

Supplementary Figure 273. ^{11}B NMR spectrum of compound 87[a]

(S)-2-(2-phenyl-1-(4,4,5,5-tetramethyl-1,3,2-dioxaborolan-2-yl)ethyl)-2,3-dihydro-1H-naphtho[1,8-de][1,3,2]diazaborinine (87[b])

^{1}H NMR (500 MHz, room temperature, $CDCl_3$)

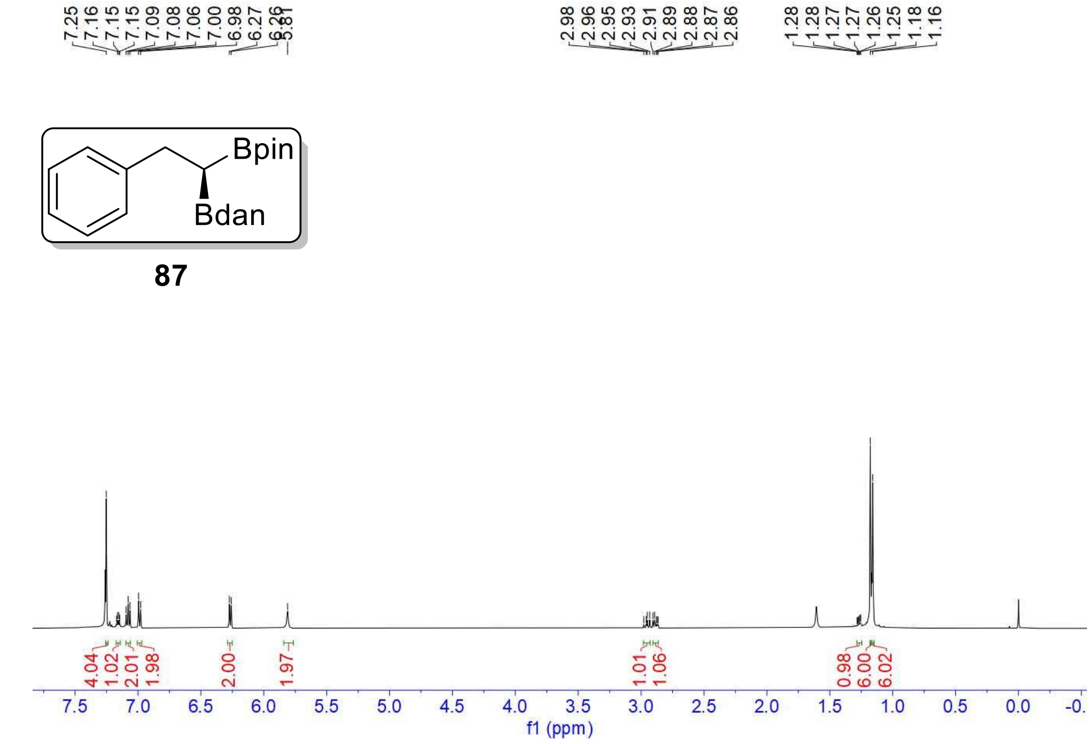

Supplementary Figure 274. ^{1}H NMR spectrum of compound 87[b]

^{13}C NMR (126 MHz, room temperature, $CDCl_3$)

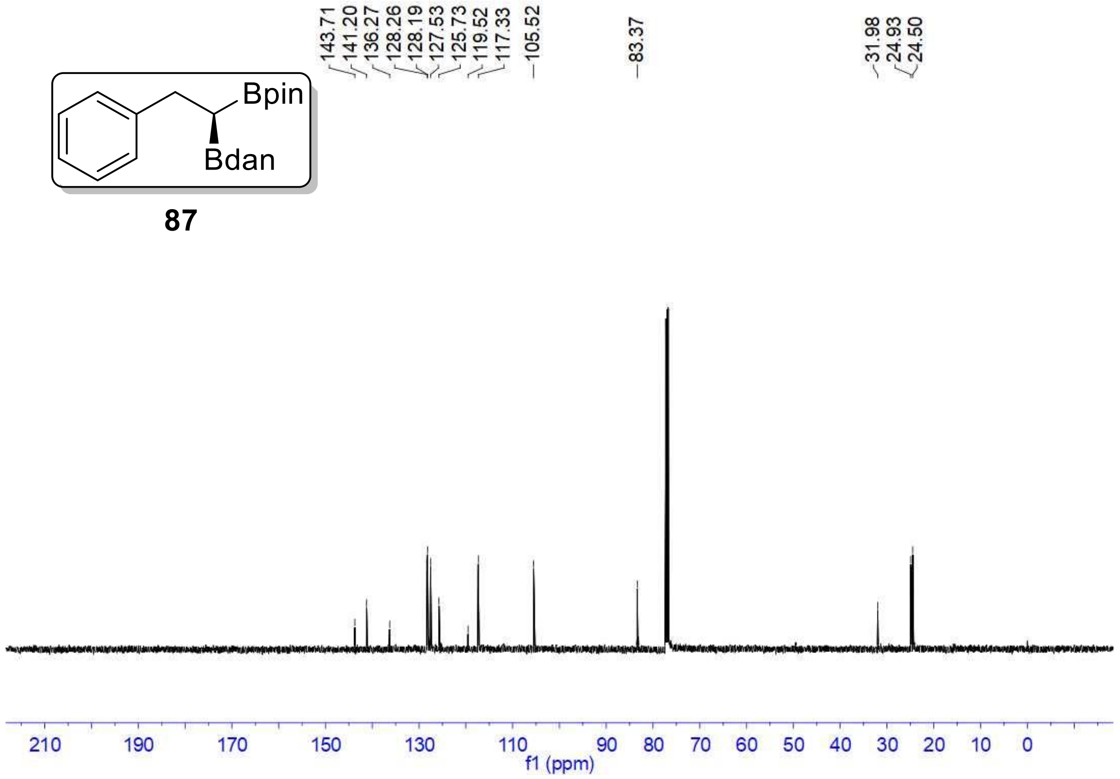

Supplementary Figure 275. ^{13}C NMR spectrum of compound 87[b]

^{11}B NMR (160 MHz, room temperature, $CDCl_3$)

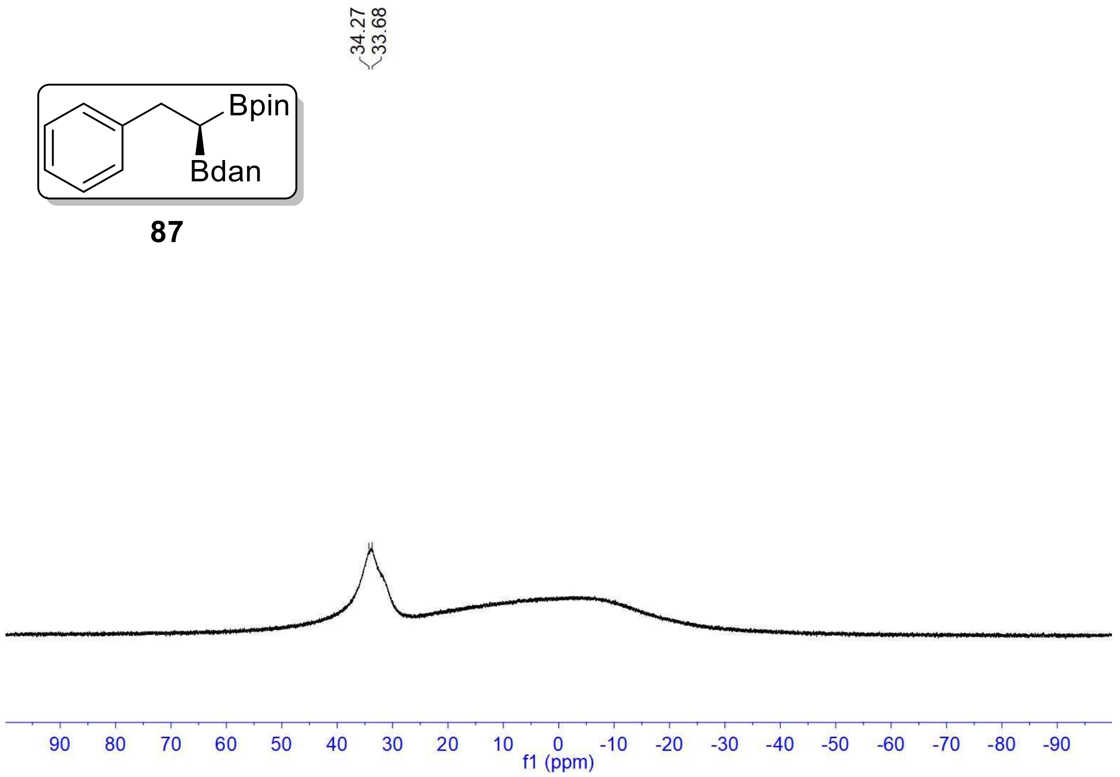

Supplementary Figure 276. ^{11}B NMR spectrum of compound 87[b]

(S)-2-(1-(4,4,5,5-tetramethyl-1,3,2-dioxaborolan-2-yl)-2-(p-tolyl)ethyl)-2,3-dihydro-1H-naphtho[1,8-de][1,3,2]diazaborinine (88)

^{1}H NMR (500 MHz, room temperature, $CDCl_3$)

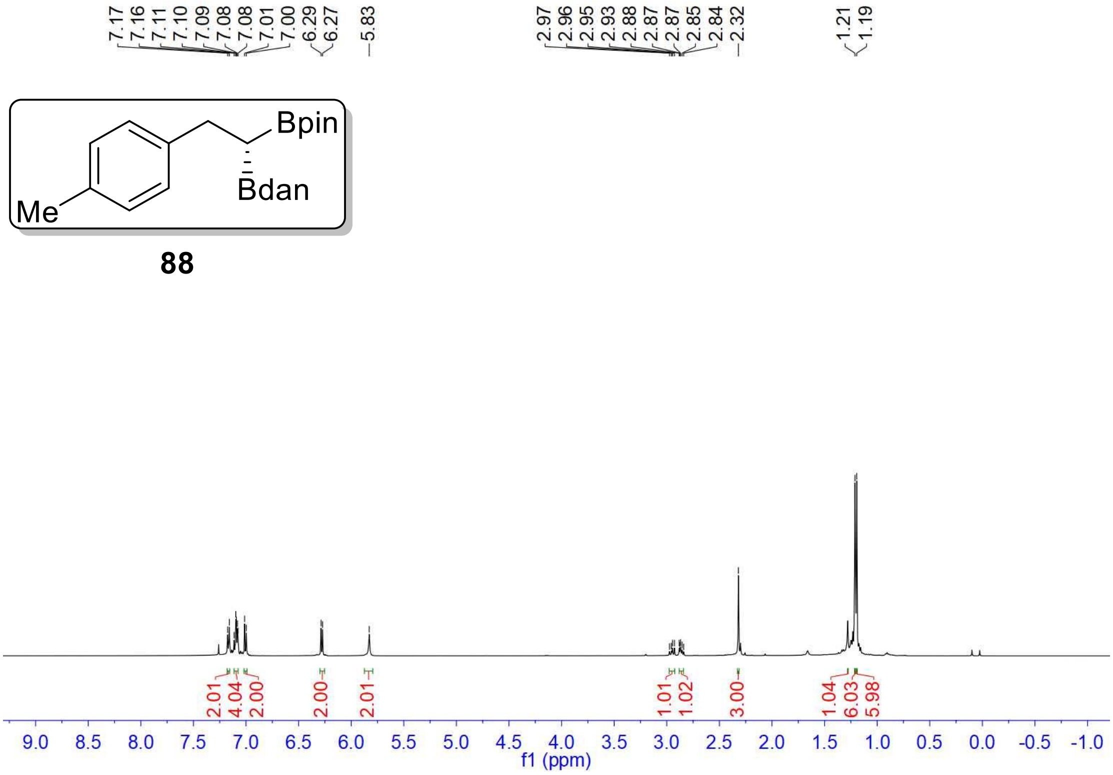

Supplementary Figure 277. ^{1}H NMR spectrum of compound 88

^{13}C NMR (126 MHz, room temperature, $CDCl_3$)

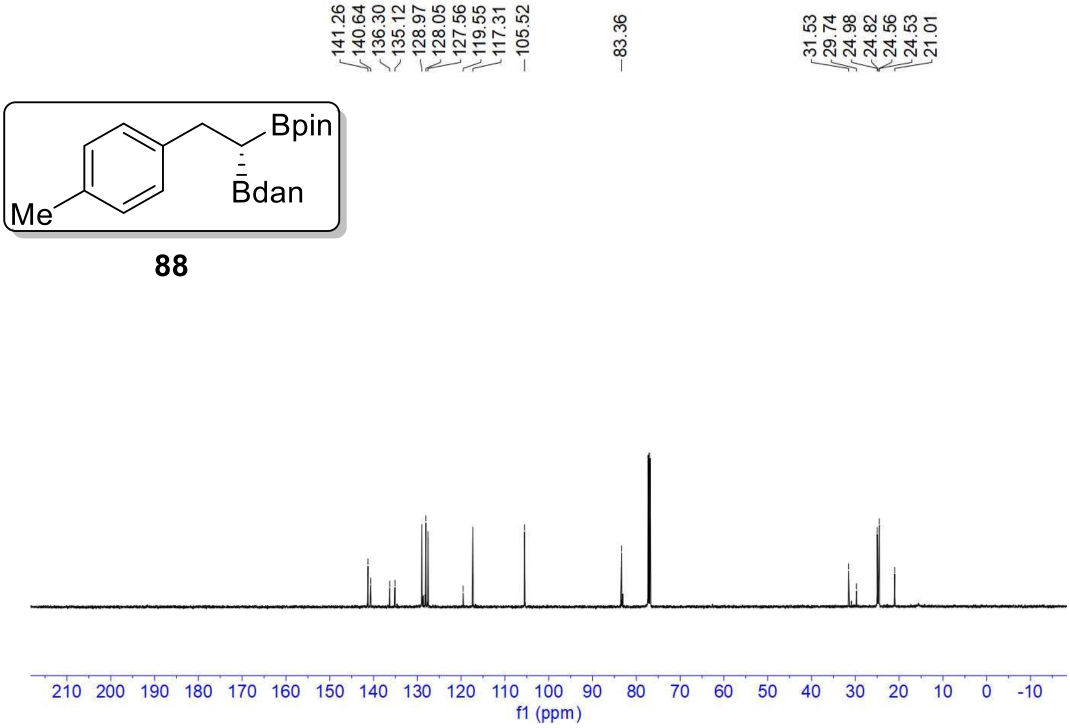

Supplementary Figure 278. ^{13}C NMR spectrum of compound 88

^{11}B NMR (160 MHz, room temperature, CDCl$_3$)

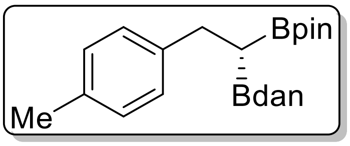

88

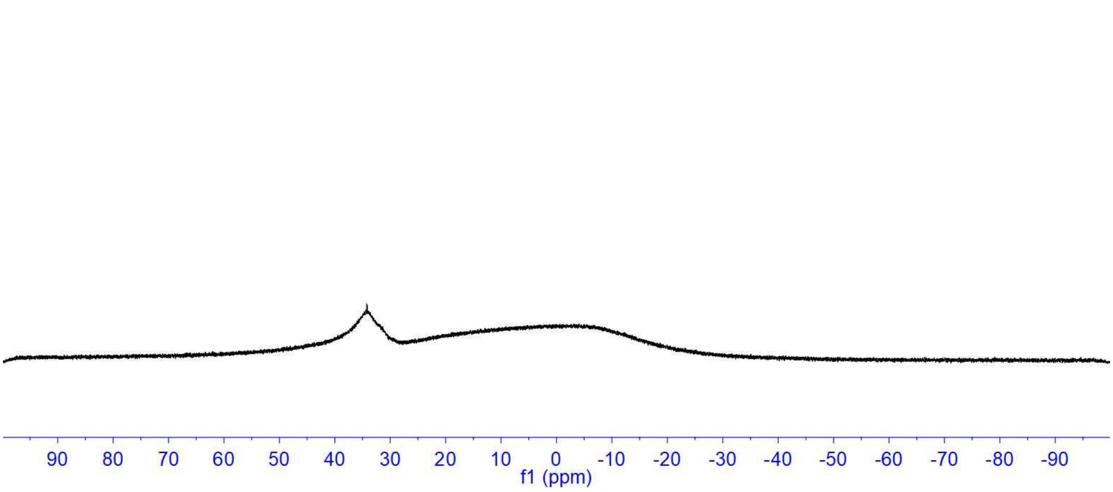

Supplementary Figure 279. ^{11}B NMR spectrum of compound 88

(S)-2-(1-(4,4,5,5-tetramethyl-1,3,2-dioxaborolan-2-yl)-2-(m-tolyl)ethyl)-2,3-dihydro-1H-naphtho[1,8-de][1,3,2]diazaborinine (89)

^{1}H NMR (500 MHz, room temperature, CDCl$_3$)

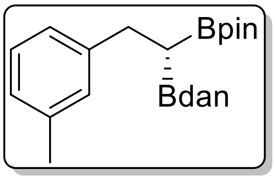

89

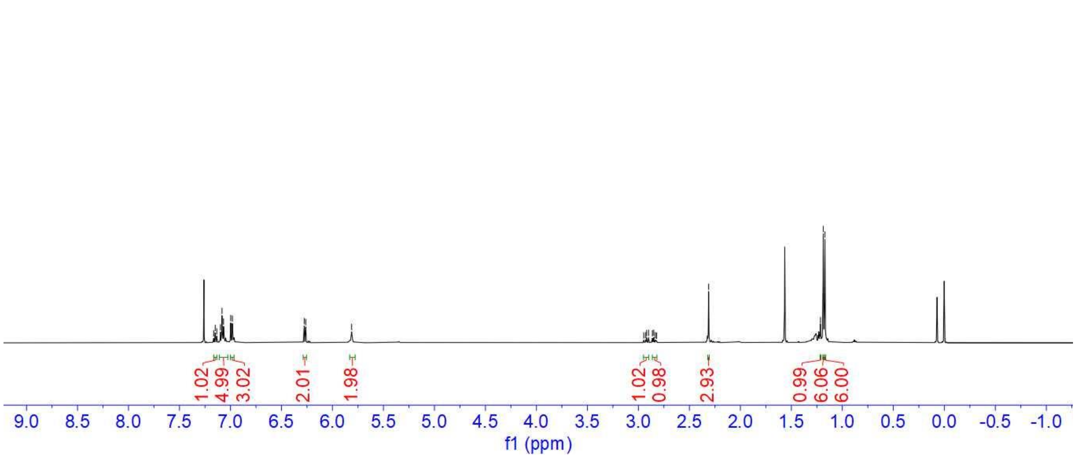

Supplementary Figure 280. ^{1}H NMR spectrum of compound 89

^{13}C NMR (126 MHz, room temperature, $CDCl_3$)

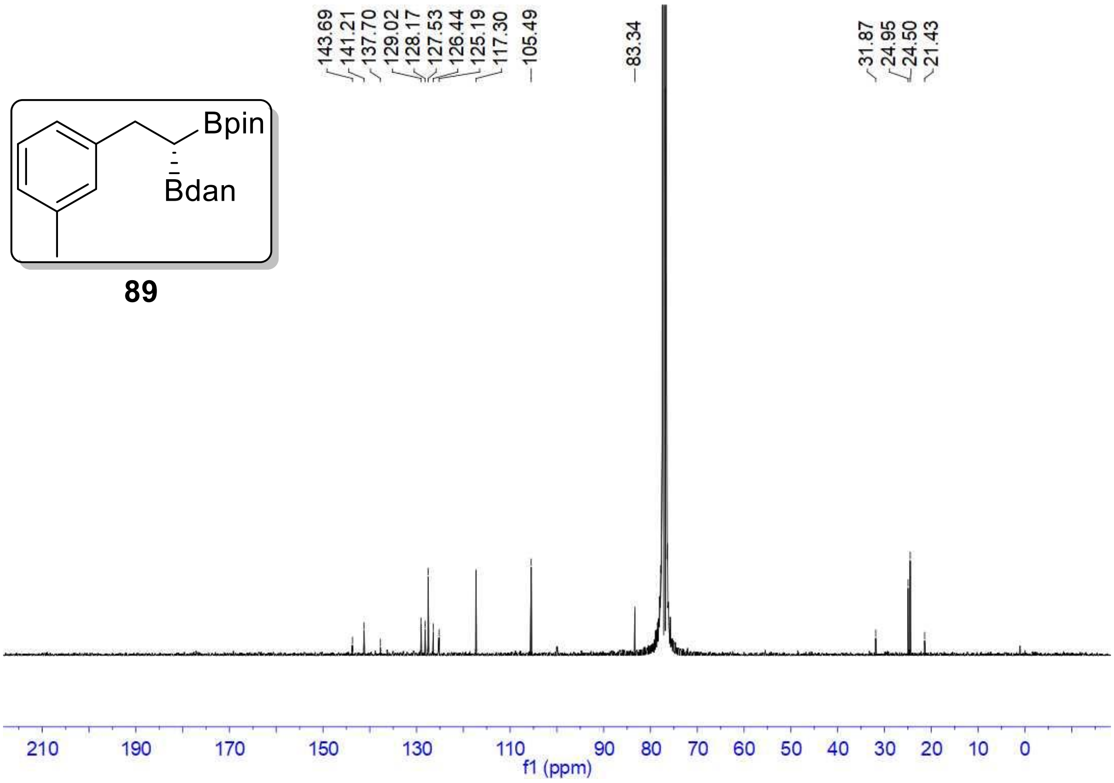

Supplementary Figure 281. ^{13}**C NMR spectrum of compound 89**

^{11}B NMR (160 MHz, room temperature, $CDCl_3$)

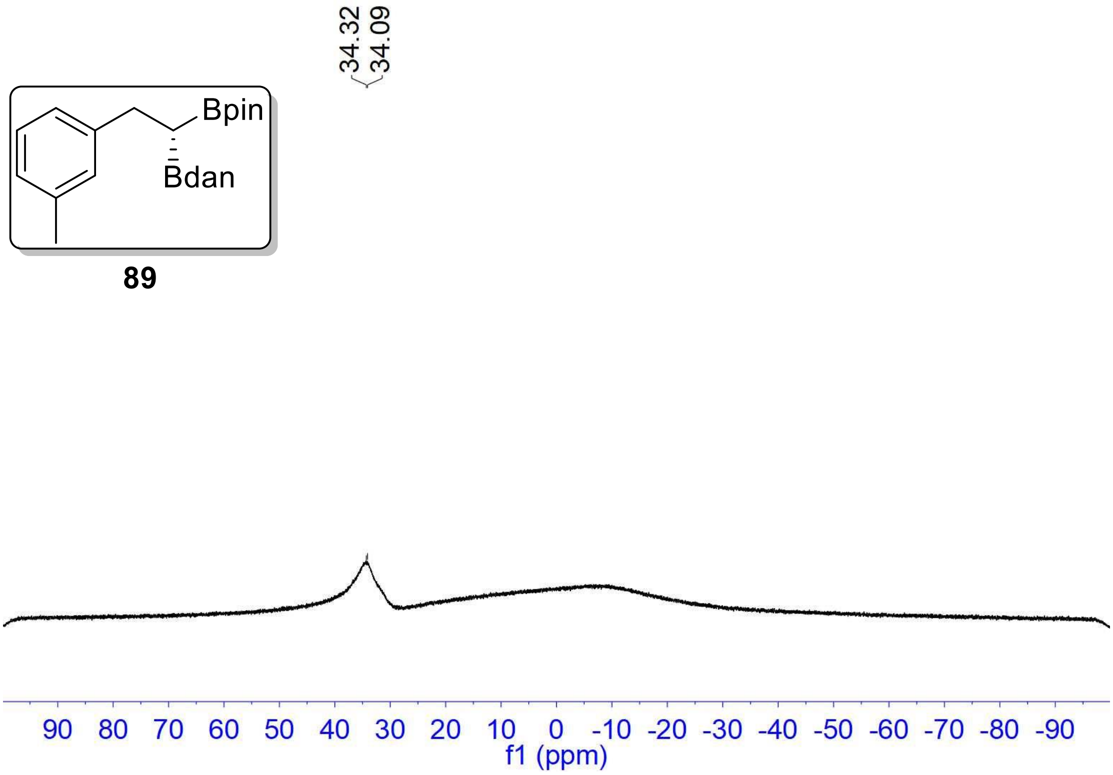

Supplementary Figure 282. ^{13}**C NMR spectrum of compound 89**

(S)-2-(2-(4-ethylphenyl)-1-(4,4,5,5-tetramethyl-1,3,2-dioxaborolan-2-yl)ethyl)-2,3-dihydro-1H-naphtho[1,8-de][1,3,2]diazaborinine (90)

^{1}H NMR (500 MHz, room temperature, $CDCl_3$)

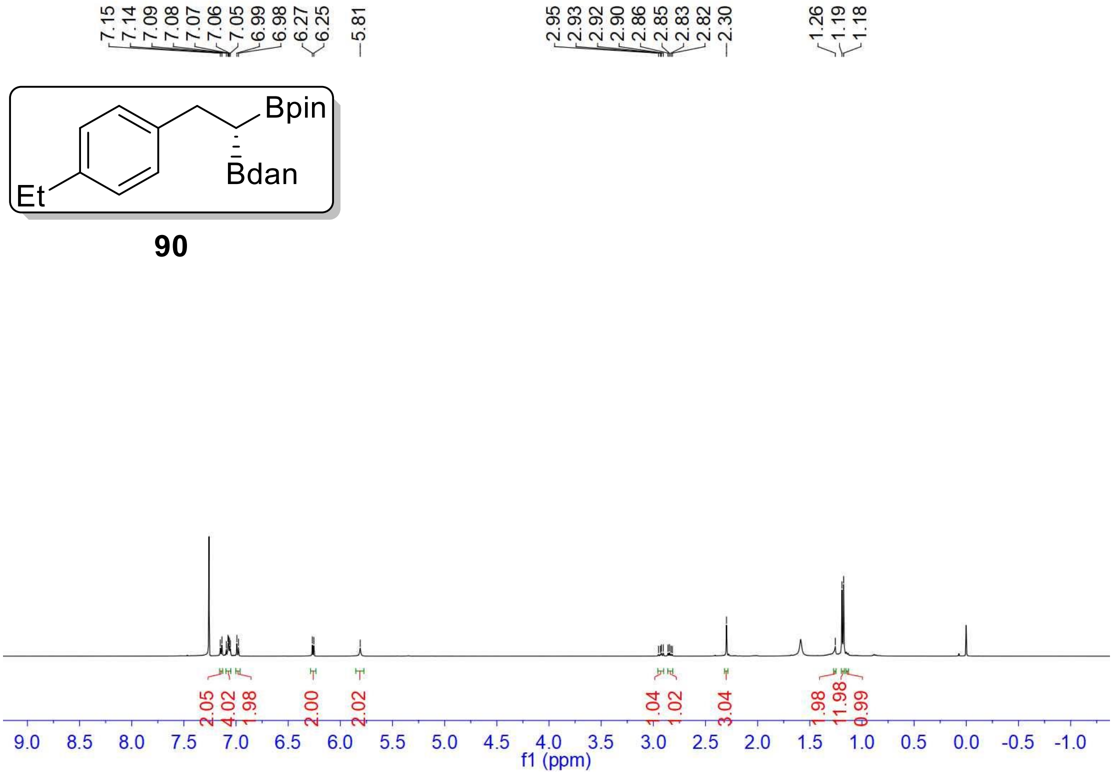

Supplementary Figure 283. ^{1}H NMR spectrum of compound 90

^{13}C NMR (126 MHz, room temperature, $CDCl_3$)

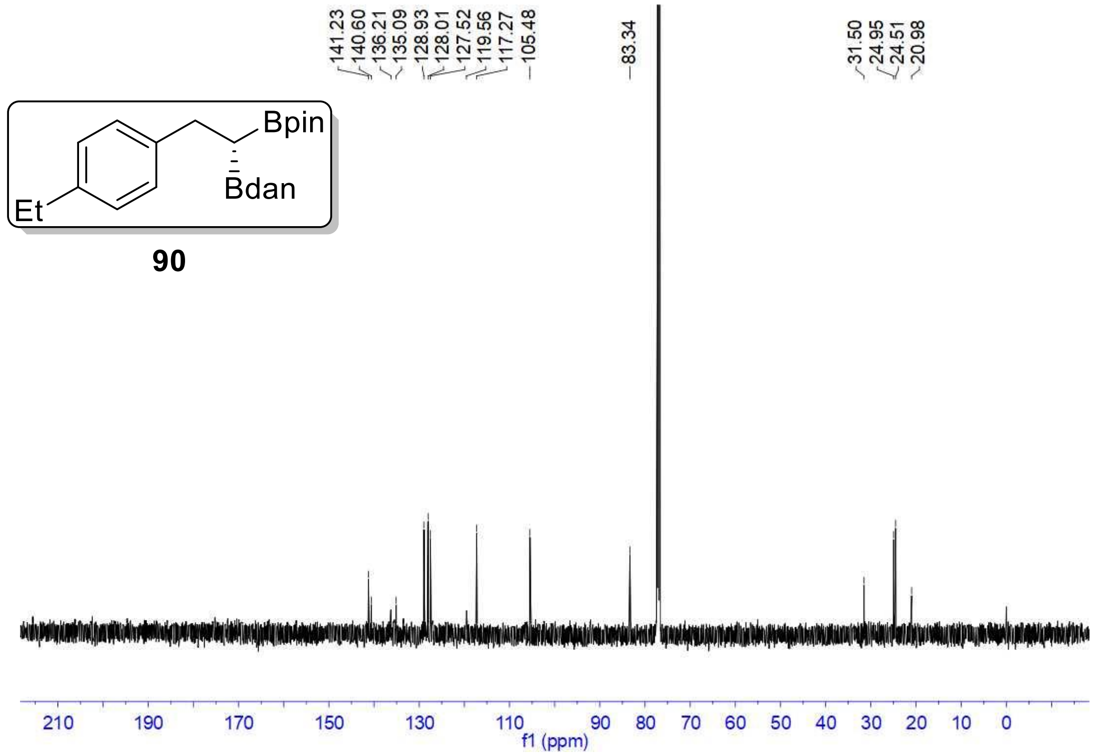

Supplementary Figure 284. ^{13}C NMR spectrum of compound 90

^{11}B NMR (160 MHz, room temperature, $CDCl_3$)

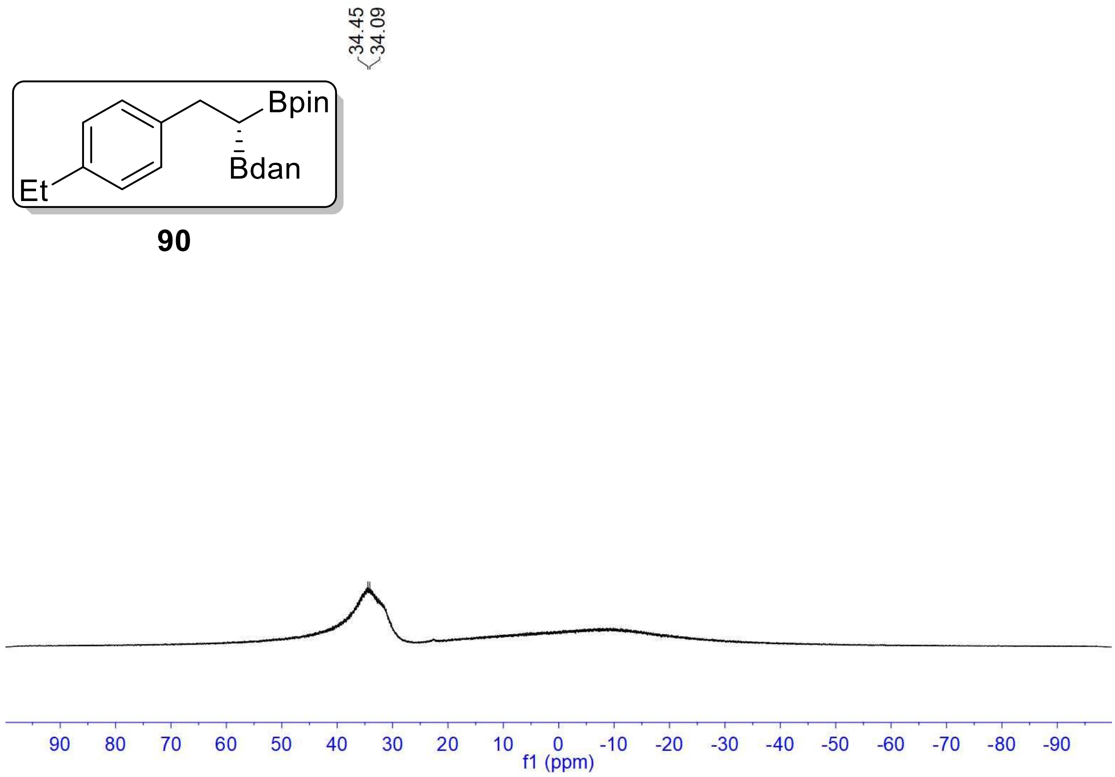

Supplementary Figure 285. ^{11}B NMR spectrum of compound 90

(S)-2-(2-(4-propylphenyl)-1-(4,4,5,5-tetramethyl-1,3,2-dioxaborolan-2-yl)ethyl)-2,3-dihydro-1H-naphtho[1,8-de][1,3,2]diazaborinine (91)

^{1}H NMR (500 MHz, room temperature, $CDCl_3$)

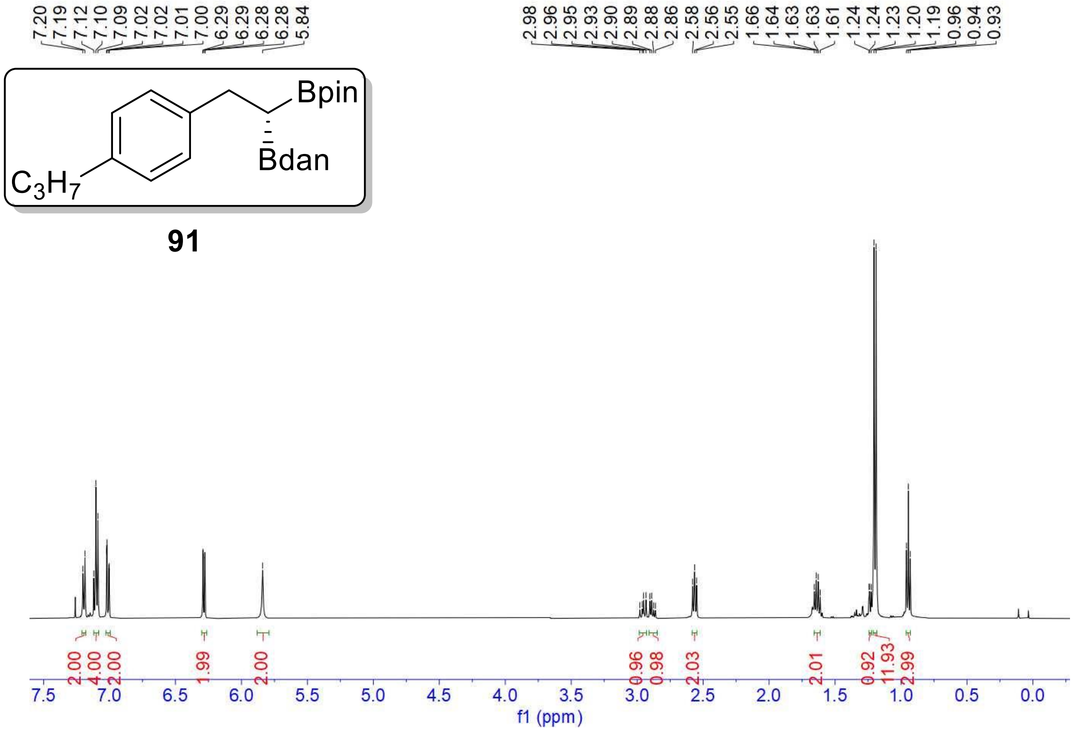

Supplementary Figure 286. ^{1}H NMR spectrum of compound 91

^{13}C NMR (126 MHz, room temperature, $CDCl_3$)

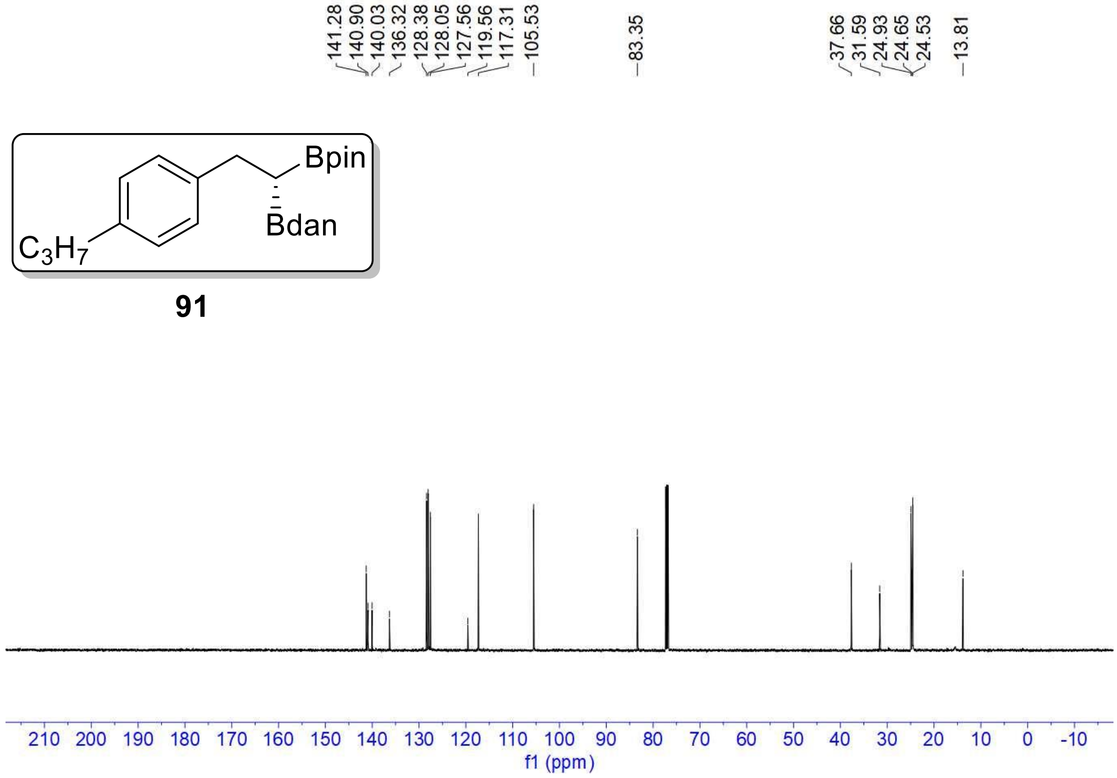

Supplementary Figure 287. ^{13}C NMR spectrum of compound 91

^{11}B NMR (160 MHz, room temperature, $CDCl_3$)

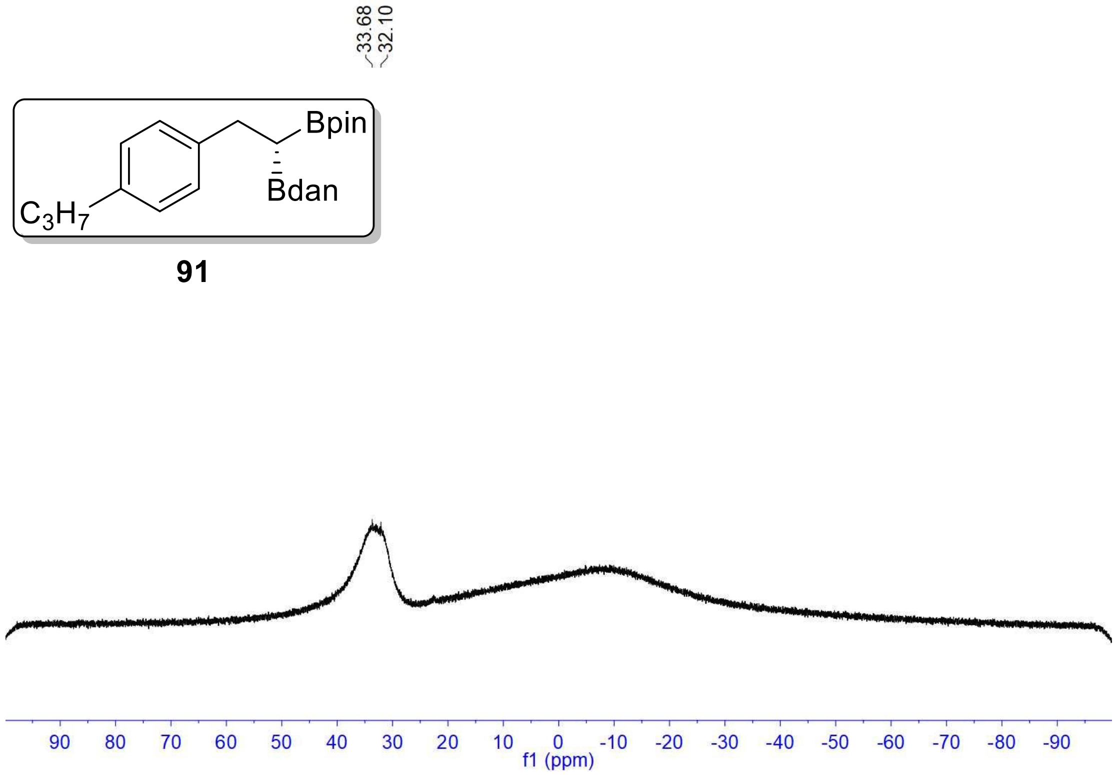

Supplementary Figure 288. ^{11}B NMR spectrum of compound 91

2-(4-phenylbutyl-1-d)-2,3-dihydro-1H-naphtho[1,8-de][1,3,2]diazaborinine (97)

^{1}H NMR (500 MHz, room temperature, $CDCl_3$)

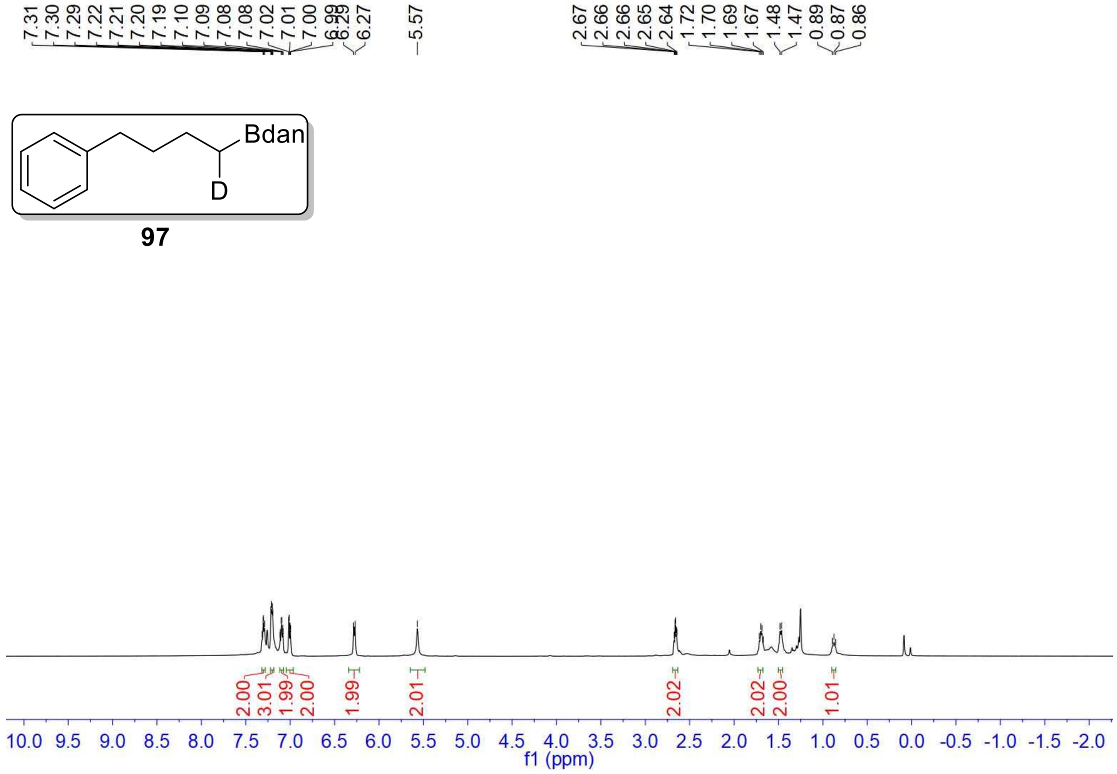

Supplementary Figure 289. ^{1}H NMR spectrum of compound 97

^{13}C NMR (126 MHz, room temperature, $CDCl_3$)

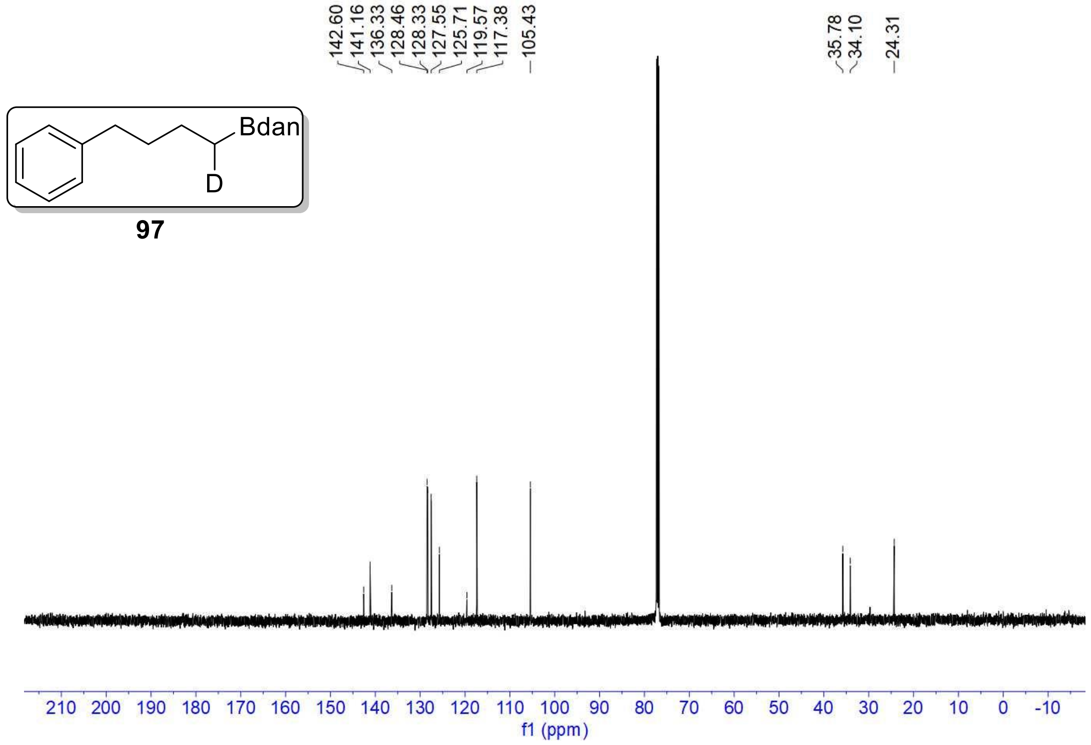

Supplementary Figure 290. ^{13}C NMR spectrum of compound 97

^{11}B NMR (160 MHz, room temperature, CDCl$_3$)

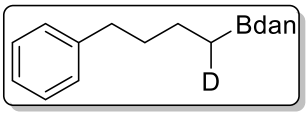

97

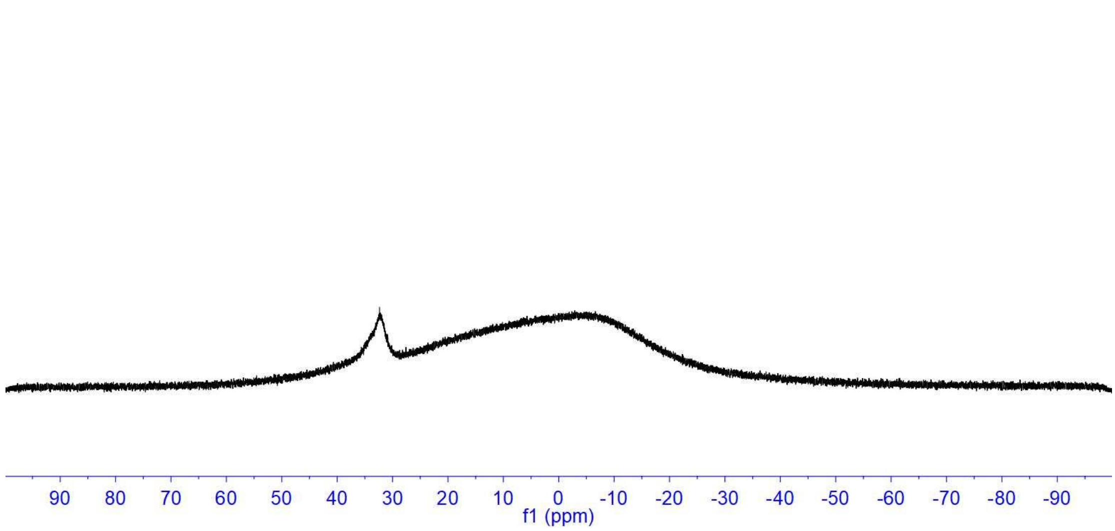

Supplementary Figure 291. ^{11}B NMR spectrum of compound 97

4.2. Chiral HPLC charts

5-(1H-naphtho[1,8-de][1,3,2]diazaborinin-2(3H)-yl)-5-(4,4,5,5-tetramethyl-1,3,2-dioxaborolan-2-yl)pentyl (2R)-2-phenylpropanoate (25)

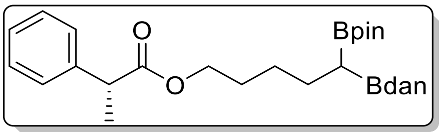

25

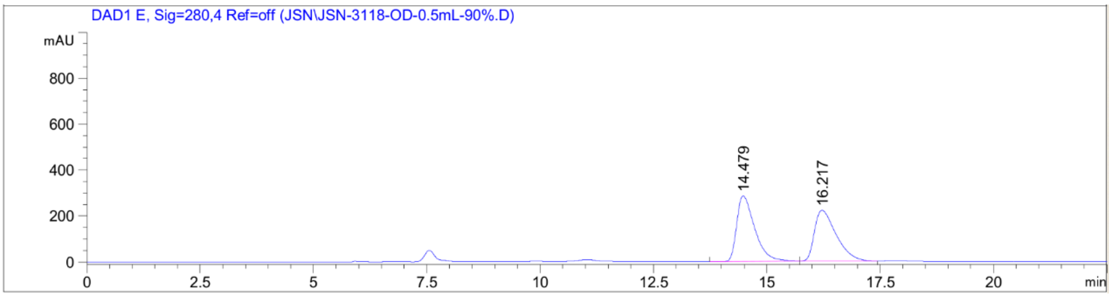

Peak #	RetTime [min]	Type	Width [min]	Area [mAU*s]	Height [mAU]	Area %
1	14.479	BV	0.4217	7917.75928	285.34573	51.6164
2	16.217	VB	0.5039	7421.85010	222.10002	48.3836

Supplementary Figure 292. HPLC of compound rac-25

5-(1H-naphtho[1,8-de][1,3,2]diazaborinin-2(3H)-yl)-5-(4,4,5,5-tetramethyl-1,3,2-dioxaborolan-2-yl)pentyl (2S)-2-(4-isobutylphenyl)propanoate (27)

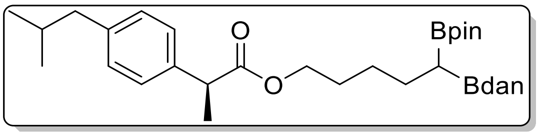

27

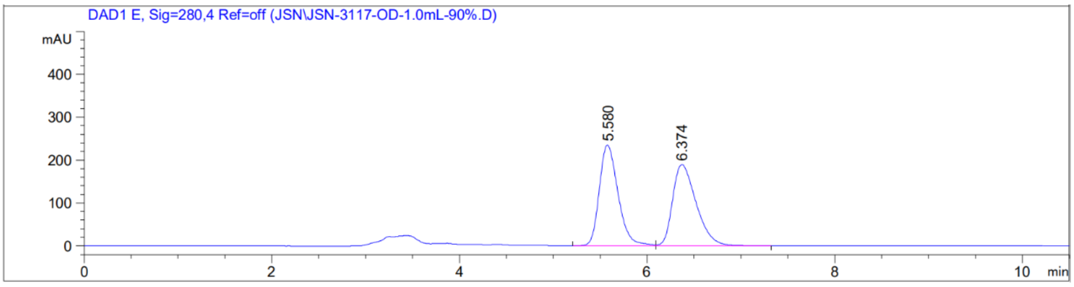

Peak #	RetTime [min]	Type	Width [min]	Area [mAU*s]	Height [mAU]	Area %
1	5.580	BV	0.2161	3261.42554	234.66853	49.7827
2	6.374	VB	0.2671	3289.90015	188.98360	50.2173

Supplementary Figure 293. HPLC of compound rac-27

5-(1H-naphtho[1,8-de][1,3,2]diazaborinin-2(3H)-yl)-5-(4,4,5,5-tetramethyl-1,3,2-dioxaborolan-2-yl)pentyl (1R,2S,4R)-bicyclo[2.2.1]hept-5-ene-2-carboxylate (29)

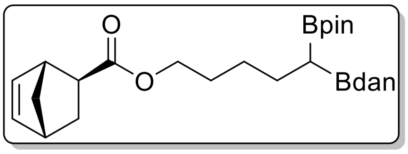

29

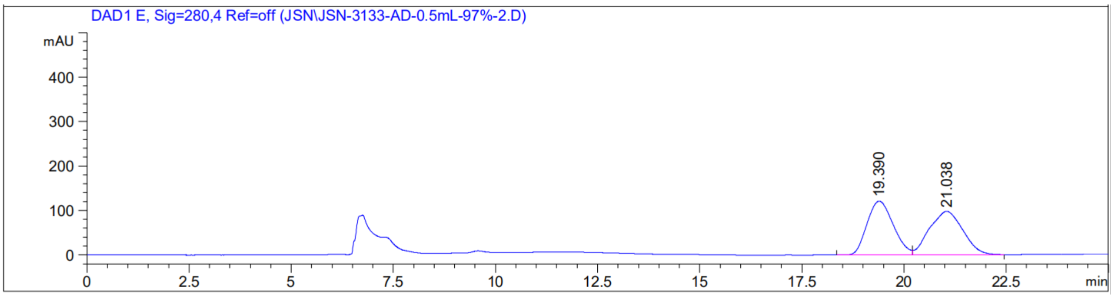

Peak #	RetTime [min]	Type	Width [min]	Area [mAU*s]	Height [mAU]	Area %
1	19.390	BV	0.6860	5390.60742	120.95336	49.4058
2	21.038	VB	0.8115	5520.27441	97.65661	50.5942

Supplementary Figure 294. HPLC of compound rac-29

5-(1H-naphtho[1,8-de][1,3,2]diazaborinin-2(3H)-yl)-5-(4,4,5,5-tetramethyl-1,3,2-dioxaborolan-2-yl)pentyl (2S)-2-(6-methoxynaphthalen-2-yl)propanoate (32)

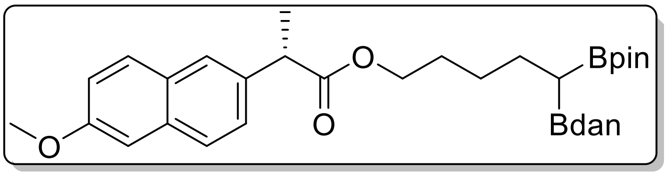

32

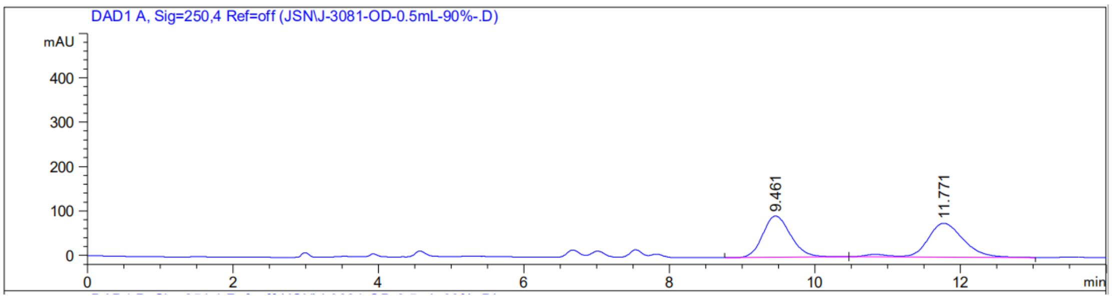

Peak #	RetTime [min]	Type	Width [min]	Area [mAU*s]	Height [mAU]	Area %

1	9.461	BB	0.4124	2476.59546	93.07779	48.3490
2	11.771	VB R	0.5116	2645.73657	76.08044	51.6510

Supplementary Figure 295. HPLC of compound rac-32

(R)-2-(4-phenyl-1-(4,4,5,5-tetramethyl-1,3,2-dioxaborolan-2-yl)butyl)-2,3-dihydro-1H-naphtho[1,8-de][1,3,2]diazaborinine (59)

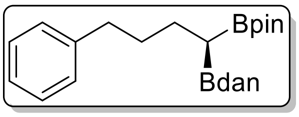

59

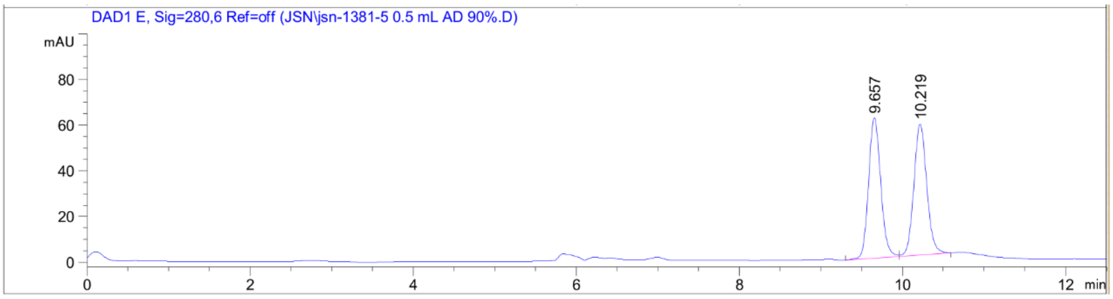

Peak #	RetTime [min]	Type	Width [min]	Area [mAU*s]	Height [mAU]	Area %
1	9.657	BV	0.1584	634.51373	61.46228	50.2326
2	10.219	VB	0.1701	628.63837	57.26398	49.7674

Supplementary Figure 296. HPLC of compound rac-59

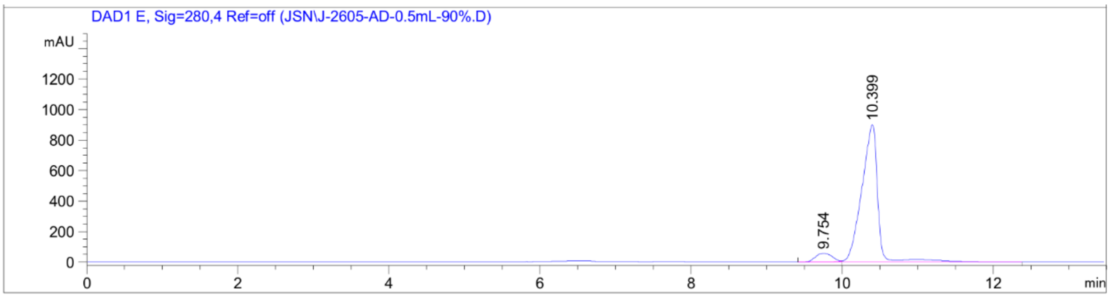

Peak #	RetTime [min]	Type	Width [min]	Area [mAU*s]	Height [mAU]	Area %
1	9.754	BV E	0.2525	878.77509	56.79230	6.1422
2	10.399	VV R	0.2079	1.34284e4	900.02173	93.8578

Supplementary Figure 297. HPLC of compound (R)-59

(R)-2-(3-phenyl-1-(4,4,5,5-tetramethyl-1,3,2-dioxaborolan-2-yl)propyl)-2,3-dihydro-1H-naphtho[1,8-de][1,3,2]diazaborinine (60)

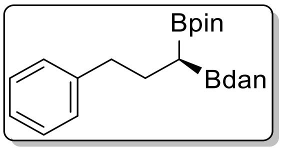

60

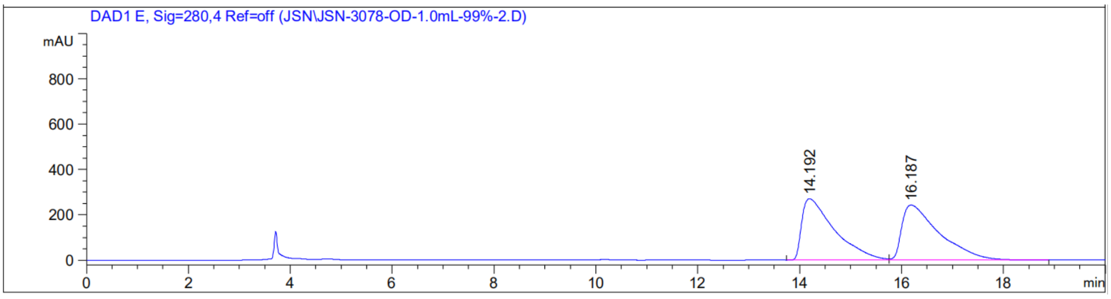

Peak #	RetTime [min]	Type	Width [min]	Area [mAU*s]	Height [mAU]	Area %
1	14.192	BV	0.6532	1.20894e4	270.63831	49.3313
2	16.187	VB	0.7475	1.24172e4	242.46112	50.6687

Supplementary Figure 298. HPLC of compound rac-59

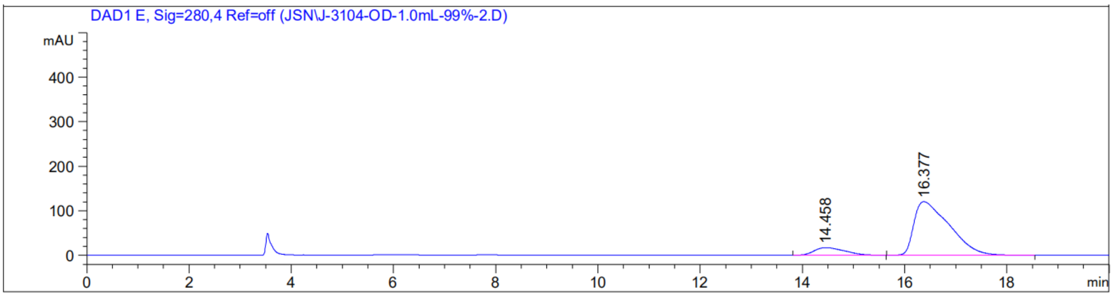

Peak #	RetTime [min]	Type	Width [min]	Area [mAU*s]	Height [mAU]	Area %
1	14.458	BB	0.5947	694.97015	17.00724	10.7639
2	16.377	BB	0.6848	5761.53369	120.29792	89.2361

Supplementary Figure 299. HPLC of compound (R)-60

(R)-2-(2-cyclohexyl-1-(4,4,5,5-tetramethyl-1,3,2-dioxaborolan-2-yl)ethyl)-2,3-dihydro-1H-naphtho[1,8-de][1,3,2]diazaborinine (61)

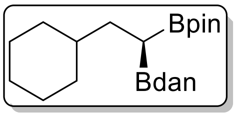

61

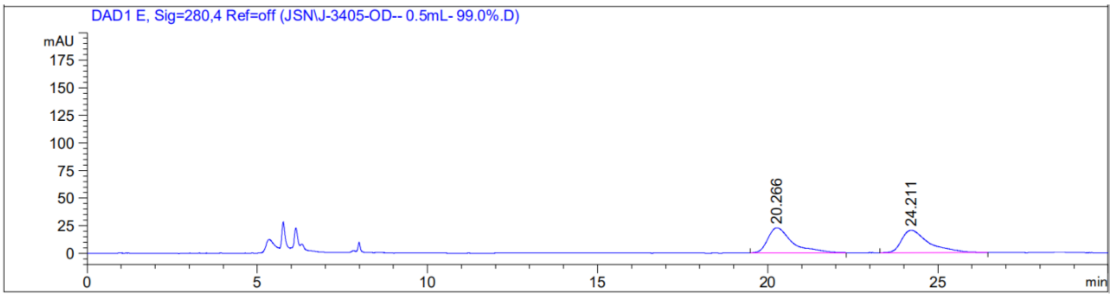

Peak #	RetTime [min]	Type	Width [min]	Area [mAU*s]	Height [mAU]	Area %
1	20.266	BB	0.6590	1131.40186	22.71955	49.8423
2	24.211	BB	0.6948	1138.56152	20.47389	50.1577

Supplementary Figure 300. HPLC of compound rac-61

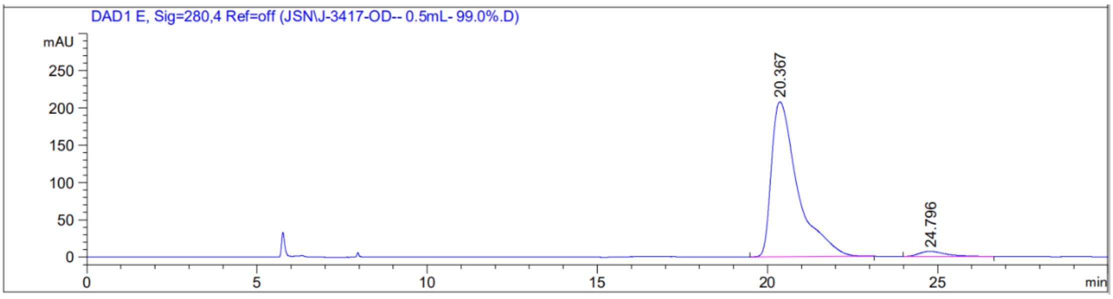

Peak #	RetTime [min]	Type	Width [min]	Area [mAU*s]	Height [mAU]	Area %
1	20.367	BB	0.7810	1.11588e4	207.63017	96.6070
2	24.796	BB	0.6633	391.91779	7.04206	3.3930

Supplementary Figure 301. HPLC of compound (R)-61

(R)-2-(2-cyclopentyl-1-(4,4,5,5-tetramethyl-1,3,2-dioxaborolan-2-yl)ethyl)-2,3-dihydro-1H-naphtho[1,8-de][1,3,2]diazaborinine (62)

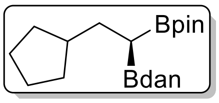

62

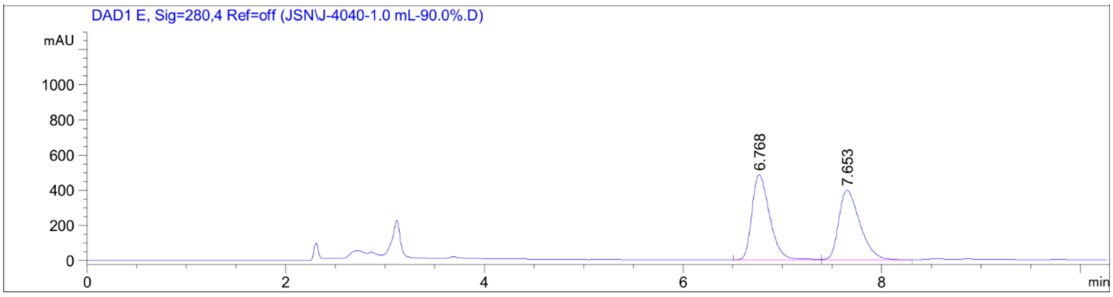

Peak #	RetTime [min]	Type	Width [min]	Area [mAU*s]	Height [mAU]	Area %

1	6.768	BV	0.1905	5982.47119	483.11530	50.6590
2	7.653	VB	0.2259	5826.82031	395.34924	49.3410

Supplementary Figure 302. HPLC of compound rac-62

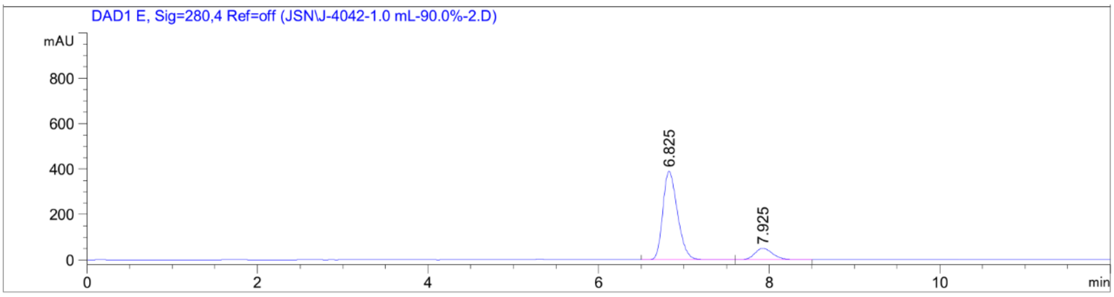

Peak #	RetTime [min]	Type	Width [min]	Area [mAU*s]	Height [mAU]	Area %
1	6.825	BB	0.1853	4650.33105	389.37024	86.3160
2	7.925	BB	0.2291	737.23468	50.26014	13.6840

Supplementary Figure 303. HPLC of compound (R)-62

(R)-2-(2-cyclopropyl-1-(4,4,5,5-tetramethyl-1,3,2-dioxaborolan-2-yl)ethyl)-2,3-dihydro-1H-naphtho[1,8-de][1,3,2]diazaborinine (63)

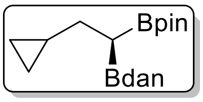

63

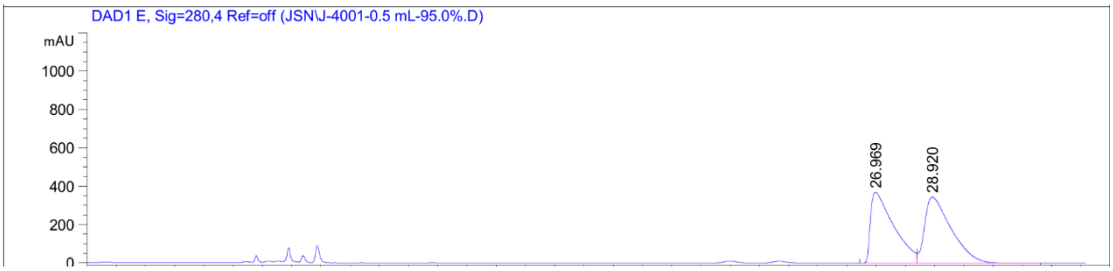

Peak #	RetTime [min]	Type	Width [min]	Area [mAU*s]	Height [mAU]	Area %
1	26.969	BV	0.7811	2.03004e4	369.41107	48.2878
2	28.920	VB	0.9216	2.17400e4	344.77774	51.7122

Supplementary Figure 304. HPLC of compound rac-63

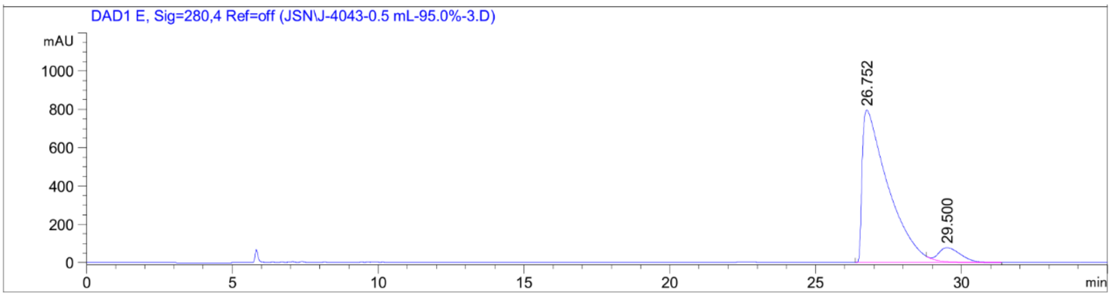

Peak #	RetTime [min]	Type	Width [min]	Area [mAU*s]	Height [mAU]	Area %
1	26.752	BV R	0.8714	4.93415e4	795.76880	92.8582
2	29.500	VB E	0.7887	3794.87378	73.70837	7.1418

Supplementary Figure 305. HPLC of compound (R)-63

(R)-2-(3,3-dimethyl-1-(4,4,5,5-tetramethyl-1,3,2-dioxaborolan-2-yl)butyl)-2,3-dihydro-1H-naphtho[1,8-de][1,3,2]diazaborinine (64)

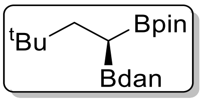

64

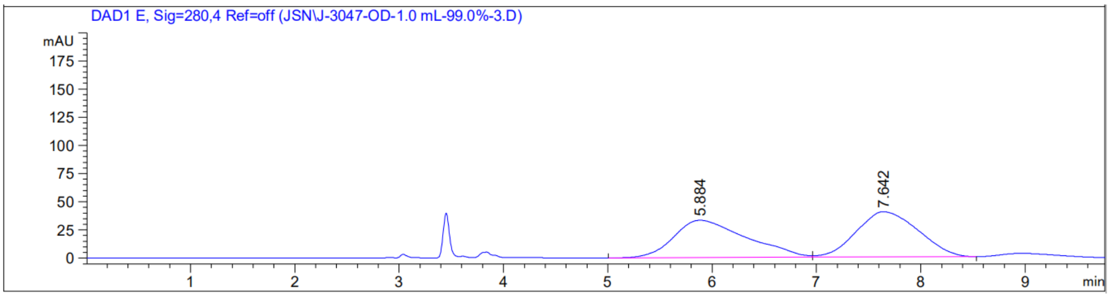

Peak #	RetTime [min]	Type	Width [min]	Area [mAU*s]	Height [mAU]	Area %
1	5.884	BV	0.6954	1606.15625	33.14324	49.2433
2	7.642	VB	0.6748	1655.52087	40.17491	50.7567

Supplementary Figure 306. HPLC of compound rac-64

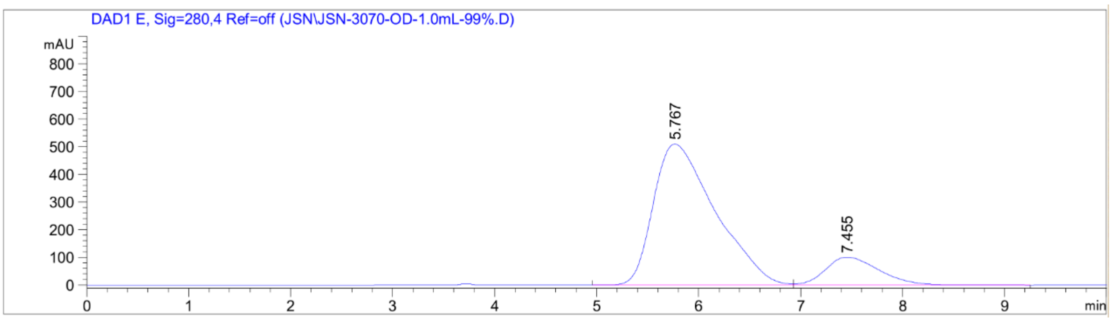

Peak #	RetTime [min]	Type	Width [min]	Area [mAU*s]	Height [mAU]	Area %

1	5.767	BV	0.6388	2.14327e4	509.44110	85.2609
2	7.455	VB	0.5604	3705.09912	101.37244	14.7391

Supplementary Figure 307. HPLC of compound (R)-64

(R)-2-(1-(4,4,5,5-tetramethyl-1,3,2-dioxaborolan-2-yl)pentyl)-2,3-dihydro-1H-naphtho[1,8-de][1,3,2]diazaborinine (65)

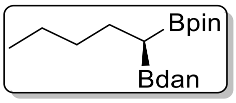

65

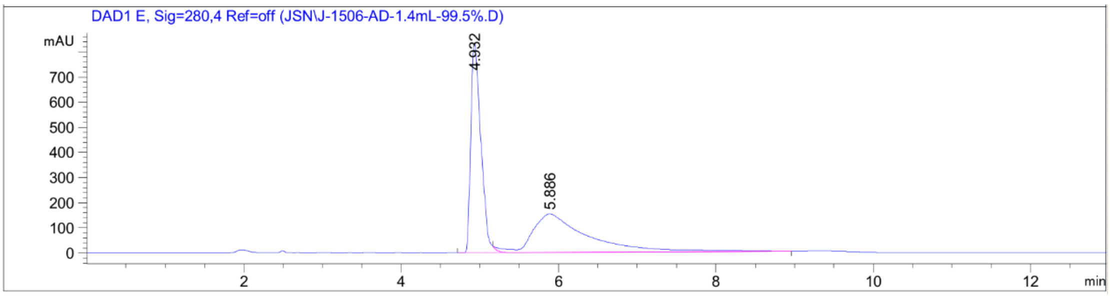

Peak #	RetTime [min]	Type	Width [min]	Area [mAU*s]	Height [mAU]	Area %
1	4.932	BV R	0.1340	7444.99023	835.30438	49.5970
2	5.886	VB E	0.6997	7565.97461	153.32700	50.4030

Supplementary Figure 308. HPLC of compound rac-65

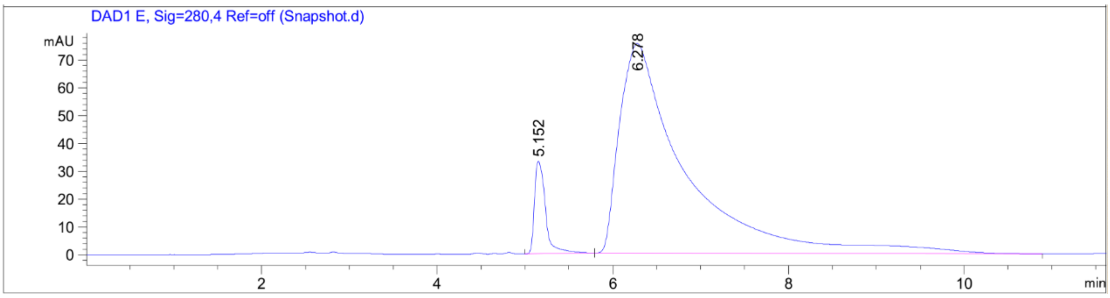

Peak #	RetTime [min]	Type	Width [min]	Area [mAU*s]	Height [mAU]	Area %
1	5.152	BB	0.1397	290.87671	33.37503	6.8202
2	6.278	BB	0.7386	3974.03784	75.43893	93.1798

Supplementary Figure 309. HPLC of compound (R)-65

(R)-2-(1-(4,4,5,5-tetramethyl-1,3,2-dioxaborolan-2-yl)hexyl)-2,3-dihydro-1H-naphtho[1,8-de][1,3,2]diazaborinine (66)

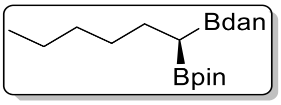

66

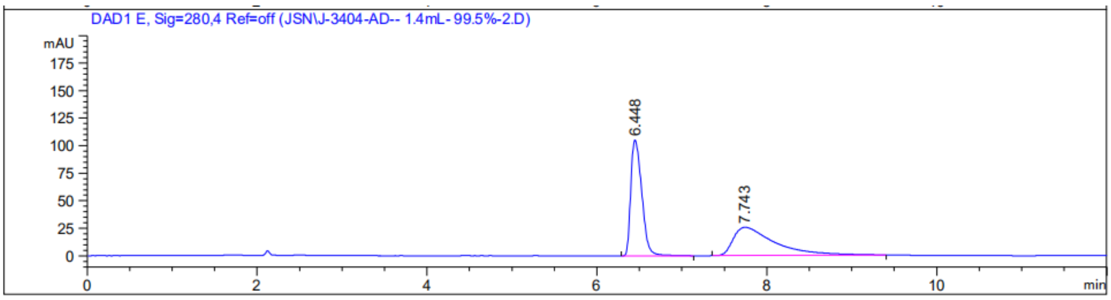

Peak #	RetTime [min]	Type	Width [min]	Area [mAU*s]	Height [mAU]	Area %
1	6.448	BB	0.1454	949.18066	105.08267	51.9872
2	7.743	BB	0.4763	876.61676	25.67881	48.0128

Supplementary Figure 310. HPLC of compound rac-66

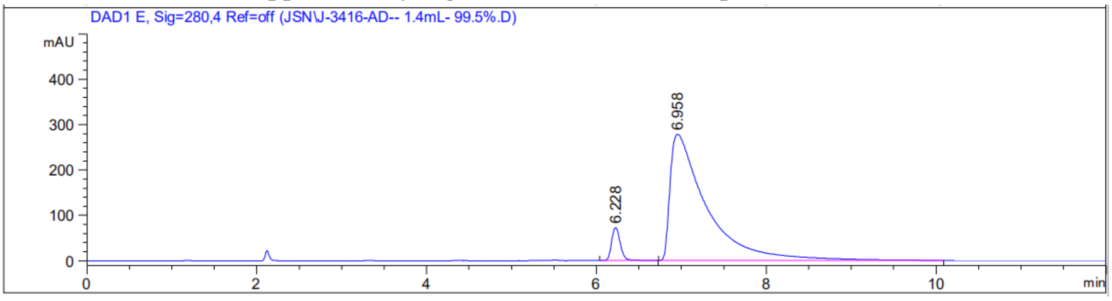

Peak #	RetTime [min]	Type	Width [min]	Area [mAU*s]	Height [mAU]	Area %
1	6.228	BB	0.1139	521.11084	72.43766	5.9008
2	6.958	BB	0.4184	8310.04102	278.30270	94.0992

Supplementary Figure 311. HPLC of compound (R)-66

(R)-2-(1-(4,4,5,5-tetramethyl-1,3,2-dioxaborolan-2-yl)heptyl)-2,3-dihydro-1H-naphtho[1,8-de][1,3,2]diazaborinine (67)

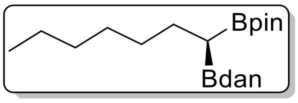

67

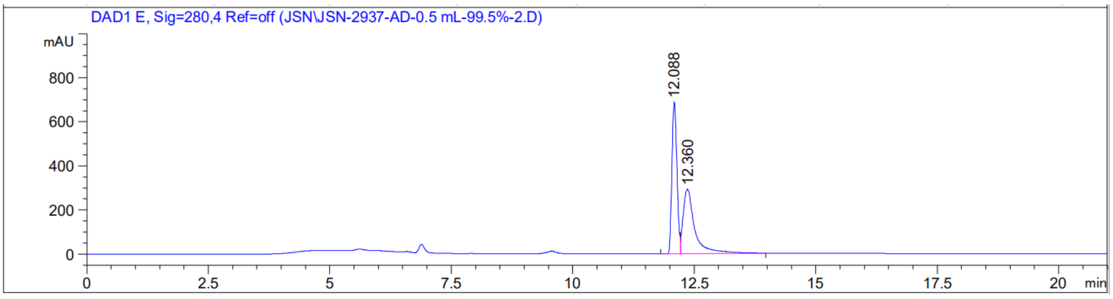

Peak #	RetTime [min]	Type	Width [min]	Area [mAU*s]	Height [mAU]	Area %
1	12.088	BV	0.1138	4940.35498	687.47717	50.4191
2	12.360	VB	0.2439	4858.22803	292.27377	49.5809

Supplementary Figure 312. HPLC of compound rac-67

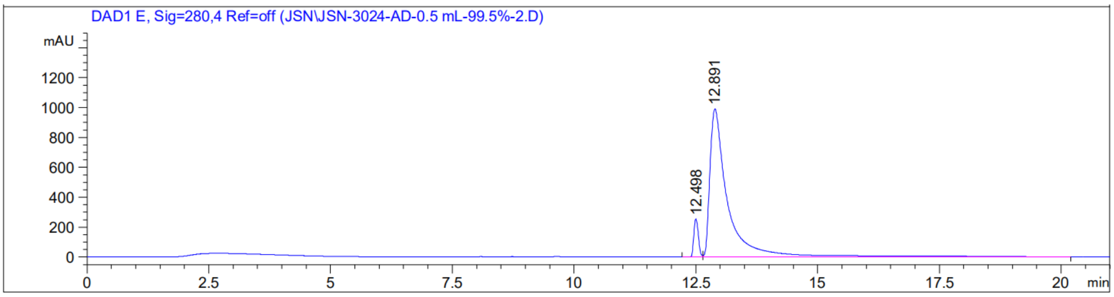

Peak #	RetTime [min]	Type	Width [min]	Area [mAU*s]	Height [mAU]	Area %
1	12.498	BV	0.1085	1654.26111	252.24028	6.0511
2	12.891	VB	0.3686	2.56840e4	990.24756	93.9489

Supplementary Figure 313. HPLC of compound (R)-67

(R)-2-(1-(4,4,5,5-tetramethyl-1,3,2-dioxaborolan-2-yl)octyl)-2,3-dihydro-1H-naphtho[1,8-de][1,3,2]diazaborinine (68)

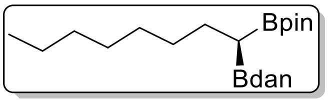

68

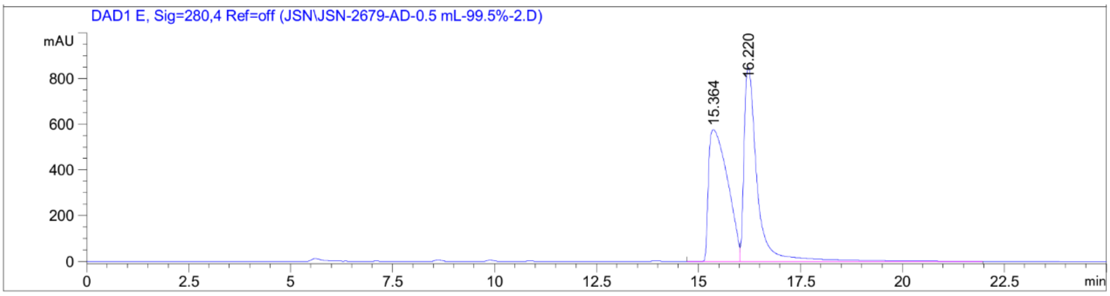

Peak #	RetTime [min]	Type	Width [min]	Area [mAU*s]	Height [mAU]	Area %

1	15.364	BV	0.5678	1.86789e4	576.88287	49.5291
2	16.220	VB	0.3354	1.90341e4	849.55981	50.4709

Supplementary Figure 314. HPLC of compound rac-68

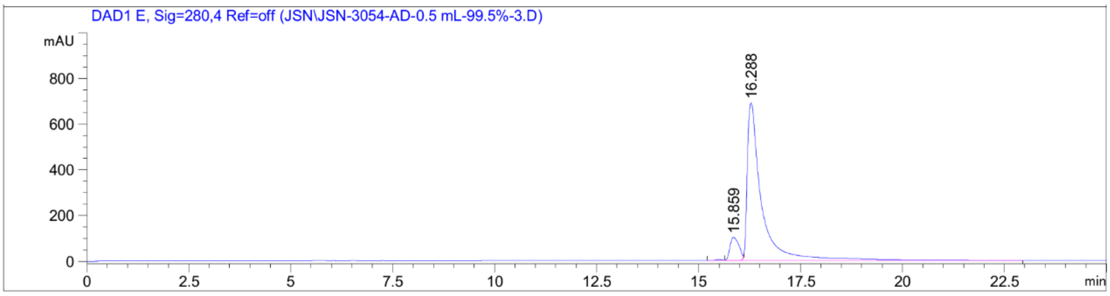

Peak #	RetTime [min]	Type	Width [min]	Area [mAU*s]	Height [mAU]	Area %
1	15.859	VV E	0.2648	1564.93420	101.00799	8.3759
2	16.288	VB R	0.3503	1.71188e4	690.36987	91.6241

Supplementary Figure 315. HPLC of compound (R)-68

(R)-2-(1-(4,4,5,5-tetramethyl-1,3,2-dioxaborolan-2-yl)octyl)-2,3-dihydro-1H-naphtho[1,8-de][1,3,2]diazaborinine (69)

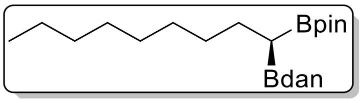

69

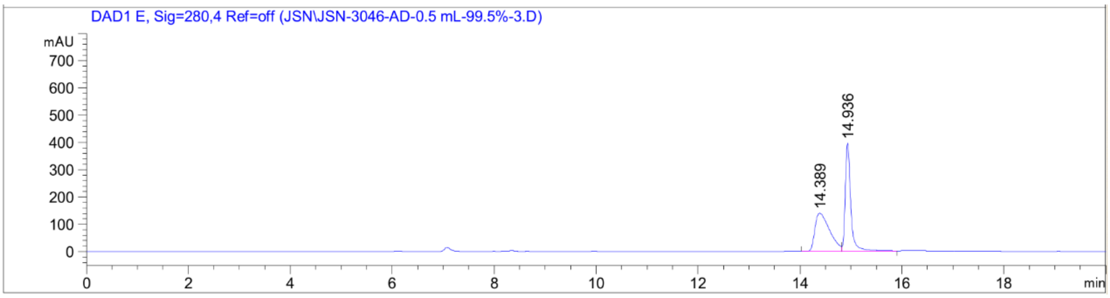

Peak #	RetTime [min]	Type	Width [min]	Area [mAU*s]	Height [mAU]	Area %
1	14.389	BV	0.3112	2783.11621	139.21651	48.6469
2	14.936	VB	0.1128	2937.93481	394.90619	51.3531

Supplementary Figure 316. HPLC of compound rac-69

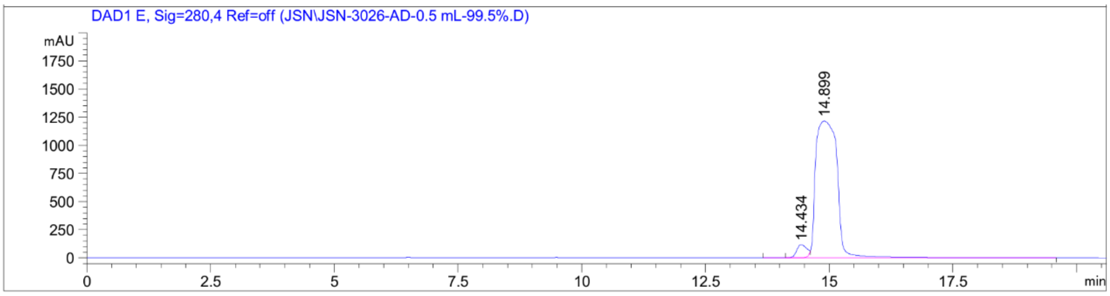

Peak #	RetTime [min]	Type	Width [min]	Area [mAU*s]	Height [mAU]	Area %
1	14.434	VV E	0.2141	1596.14941	116.32363	4.1939
2	14.899	VB R	0.4983	3.64622e4	1214.45532	95.8061

Supplementary Figure 317. HPLC of compound (R)-69

(R)-2-(5-methyl-1-(4,4,5,5-tetramethyl-1,3,2-dioxaborolan-2-yl)hexyl)-2,3-dihydro-1H-naphtho[1,8-de][1,3,2]diazaborinine (70)

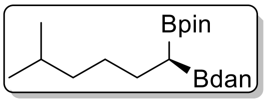

70

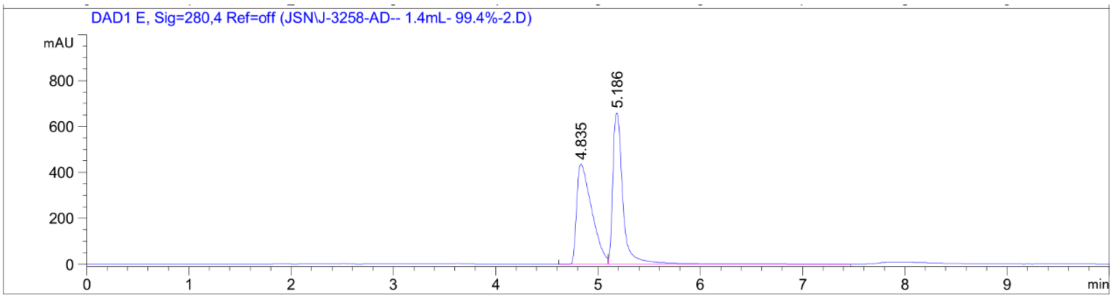

Peak #	RetTime [min]	Type	Width [min]	Area [mAU*s]	Height [mAU]	Area %
1	4.835	BV	0.1567	4442.48340	436.79803	49.2399
2	5.186	VB	0.1069	4579.63916	660.33038	50.7601

Supplementary Figure 318. HPLC of compound rac-70

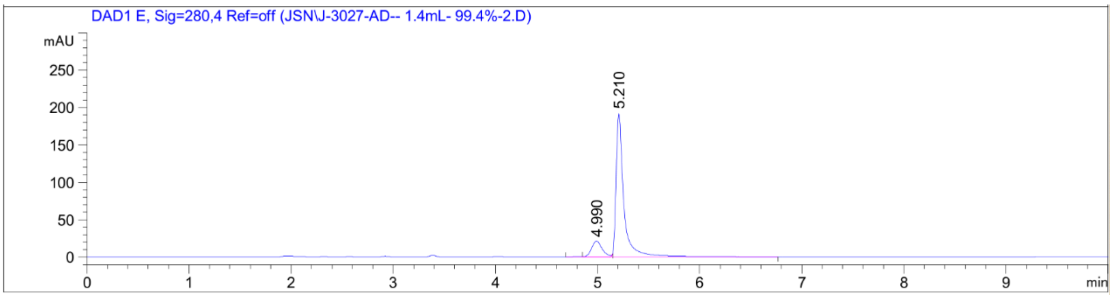

Peak #	RetTime [min]	Type	Width [min]	Area [mAU*s]	Height [mAU]	Area %

1	4.990	VV E	0.1086	149.74039	21.14183	12.9321
2	5.210	VB R	0.0753	1008.15424	192.02641	87.0679

Supplementary Figure 319. HPLC of compound (R)-70

(R)-tert-butyl 4-(2-(1H-naphtho[1,8-de][1,3,2]diazaborinin-2(3H)-yl)-2-(4,4,5,5-tetramethyl-1,3,2-dioxaborolan-2-yl)ethyl)piperidine-1-carboxylate (71)

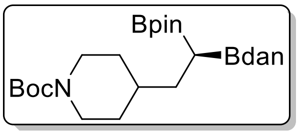

71

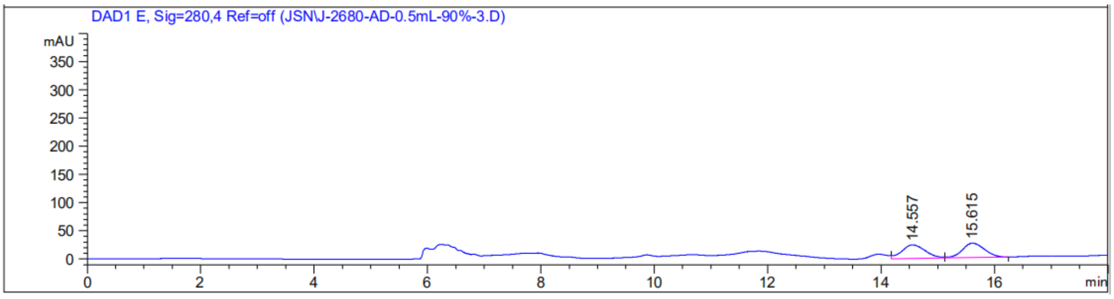

Peak #	RetTime [min]	Type	Width [min]	Area [mAU*s]	Height [mAU]	Area %
1	14.557	VV	0.4437	682.01819	23.84302	49.5257
2	15.615	VB	0.4212	695.08228	25.56347	50.4743

Supplementary Figure 320. HPLC of compound rac-71

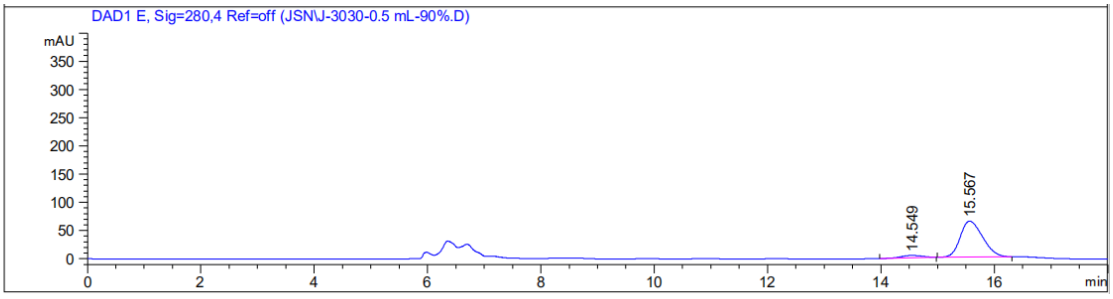

Peak #	RetTime [min]	Type	Width [min]	Area [mAU*s]	Height [mAU]	Area %
1	14.549	BB	0.4119	112.36375	4.28607	6.0284
2	15.567	BB	0.4306	1751.53357	63.73475	93.9716

Supplementary Figure 321. HPLC of compound (R)-71

(R)-2-(4-((tert-butyldimethylsilyl)oxy)-1-(4,4,5,5-tetramethyl-1,3,2-dioxaborolan-2-yl)butyl)-2,3-dihydro-1H-naphtho[1,8-de][1,3,2]diazaborinine (72)

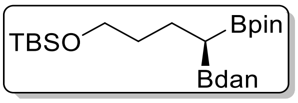

72

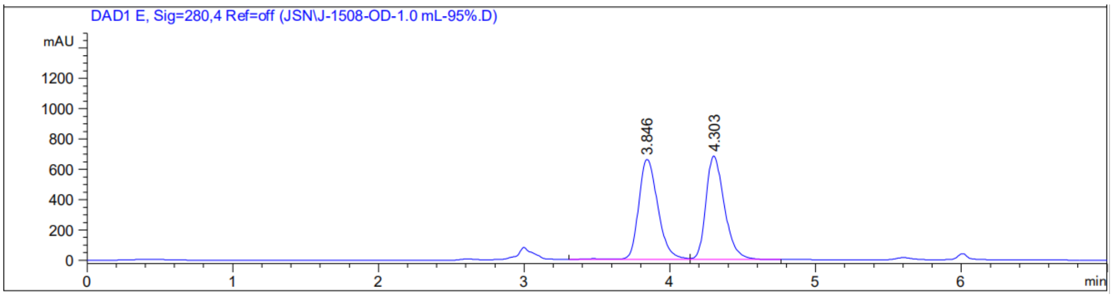

Peak #	RetTime [min]	Type	Width [min]	Area [mAU*s]	Height [mAU]	Area %
1	3.846	VV R	0.1403	6057.28271	659.75140	50.1609
2	4.303	VB	0.1346	6018.42969	684.03479	49.8391

Supplementary Figure 322. HPLC of compound rac-72

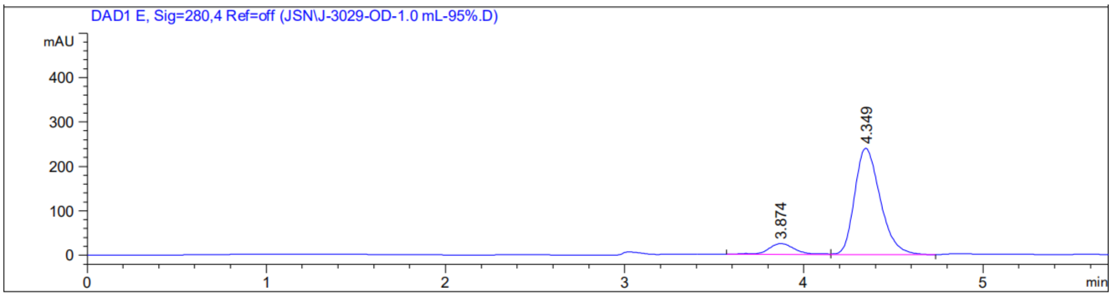

Peak #	RetTime [min]	Type	Width [min]	Area [mAU*s]	Height [mAU]	Area %
1	3.874	VV R	0.1544	250.04866	24.26586	9.5449
2	4.349	VB	0.1535	2369.64746	239.29808	90.4551

Supplementary Figure 323. HPLC of compound (R)-72

(R)-2-(6-((tert-butyldimethylsilyl)oxy)-1-(4,4,5,5-tetramethyl-1,3,2-dioxaborolan-2-yl)hexyl)-2,3-dihydro-1H-naphtho[1,8-de][1,3,2]diazaborinine (73)

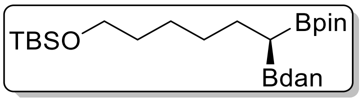

73

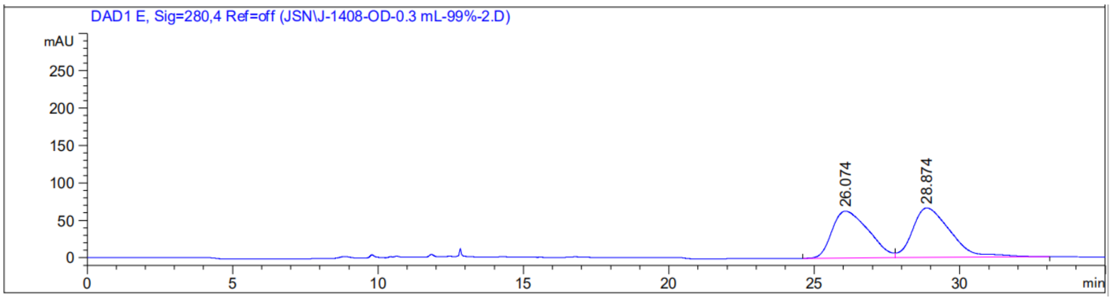

Peak #	RetTime [min]	Type	Width [min]	Area [mAU*s]	Height [mAU]	Area %
1	26.074	BV	1.2310	5440.52539	62.65578	47.6667
2	28.874	VB	1.2352	5973.15967	66.16544	52.3333

Supplementary Figure 324. HPLC of compound rac-73

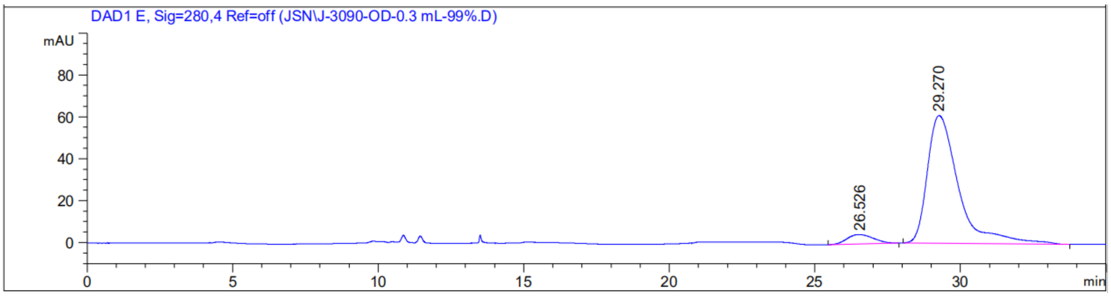

Peak #	RetTime [min]	Type	Width [min]	Area [mAU*s]	Height [mAU]	Area %
1	26.526	BB	0.7359	285.30493	4.56008	5.9039
2	29.270	BB	1.1142	4547.15088	60.96195	94.0961

Supplementary Figure 325. HPLC of compound (R)-73

(R)-2-(5-((tert-butyldiphenylsilyl)oxy)-1-(4,4,5,5-tetramethyl-1,3,2-dioxaborolan-2-yl)pentyl)-2,3-dihydro-1H-naphtho[1,8-de][1,3,2]diazaborinine (74)

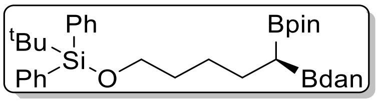

74

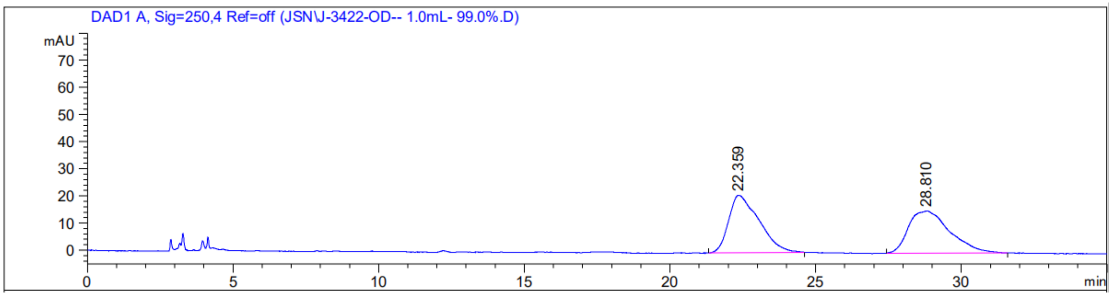

Peak #	RetTime [min]	Type	Width [min]	Area [mAU*s]	Height [mAU]	Area %
1	22.359	BB	0.8592	1536.34949	21.20619	49.8997
2	28.810	BB	1.1670	1542.52344	15.58082	50.1003

Supplementary Figure 326. HPLC of compound rac-74

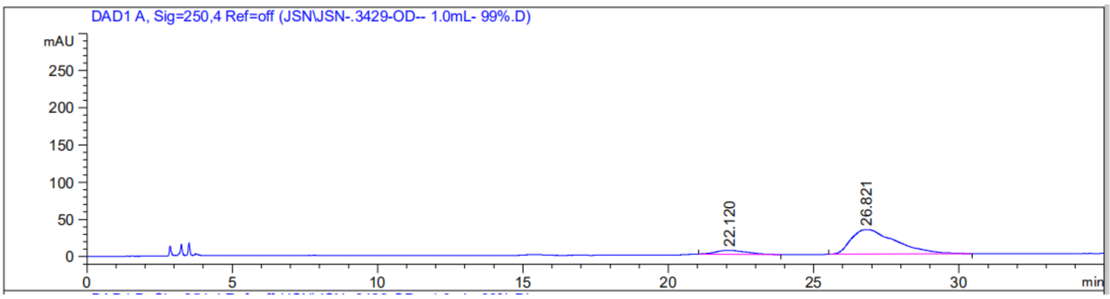

Peak #	RetTime [min]	Type	Width [min]	Area [mAU*s]	Height [mAU]	Area %
1	22.120	BB	0.8378	382.55521	5.36582	9.8158
2	26.821	BB	1.2536	3514.78198	32.86849	90.1842

Supplementary Figure 327. HPLC of compound (R)-74

(R)-2-(1-(4,4,5,5-tetramethyl-1,3,2-dioxaborolan-2-yl)-5-((triisopropylsilyl)oxy)pentyl)-2,3-dihydro-1H-naphtho[1,8-de][1,3,2]diazaborinine (75)

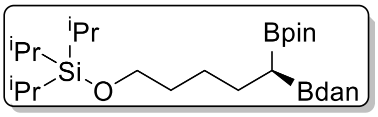

75

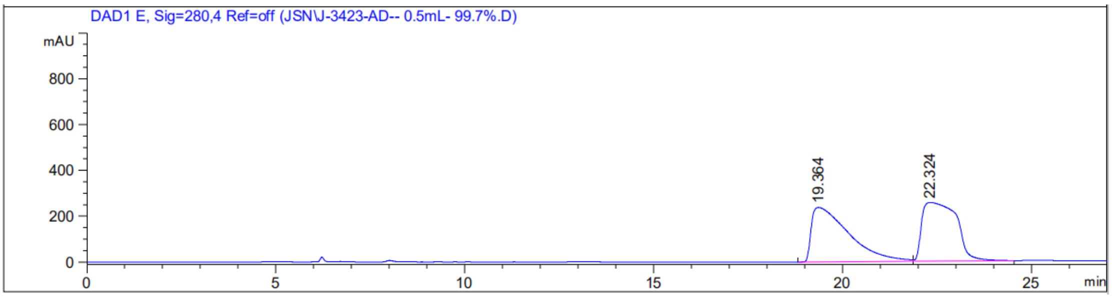

Peak #	RetTime [min]	Type	Width [min]	Area [mAU*s]	Height [mAU]	Area %
1	19.364	BV	1.0287	1.63515e4	237.65356	49.9824
2	22.324	VB	1.0670	1.63630e4	256.24603	50.0176

Supplementary Figure 328. HPLC of compound rac-75

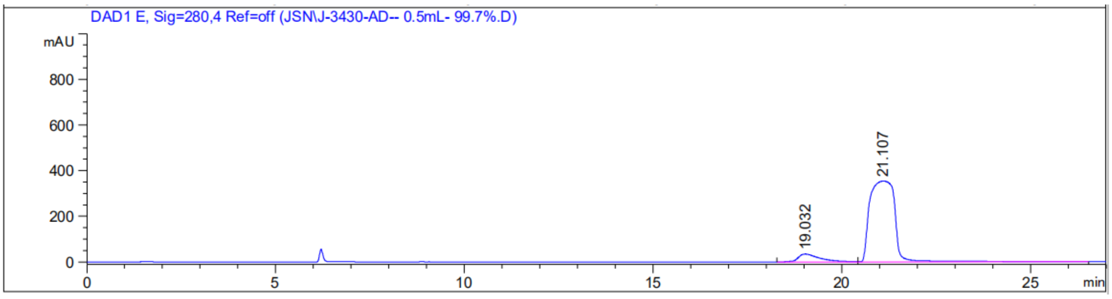

Peak #	RetTime [min]	Type	Width [min]	Area [mAU*s]	Height [mAU]	Area %

1	19.032	BV	0.6016	1473.51355	35.39914	8.1744
2	21.107	VB	0.7746	1.65523e4	354.57391	91.8256

Supplementary Figure 329. HPLC of compound (R)-75

(R)-5-(1H-naphtho[1,8-de][1,3,2]diazaborinin-2(3H)-yl)-5-(4,4,5,5-tetramethyl-1,3,2-dioxaborolan-2-yl)pentyl propionate (76)

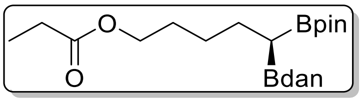

76

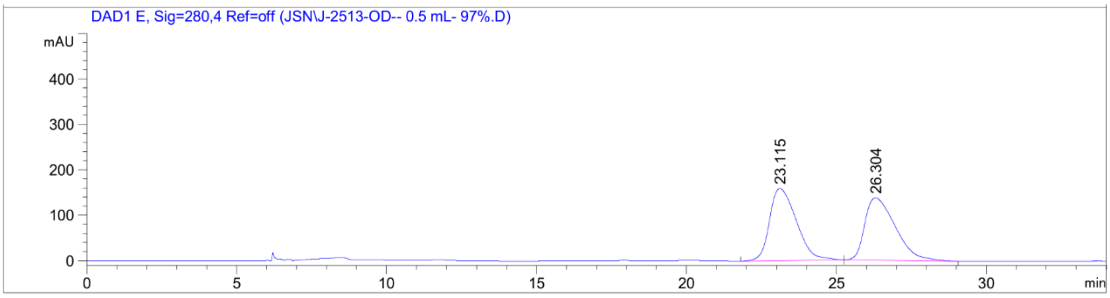

Peak #	RetTime [min]	Type	Width [min]	Area [mAU*s]	Height [mAU]	Area %
1	23.115	BB	0.9577	9865.75195	158.60326	50.3779
2	26.304	BB	1.0479	9717.75684	137.27243	49.6221

Supplementary Figure 330. HPLC of compound rac-76

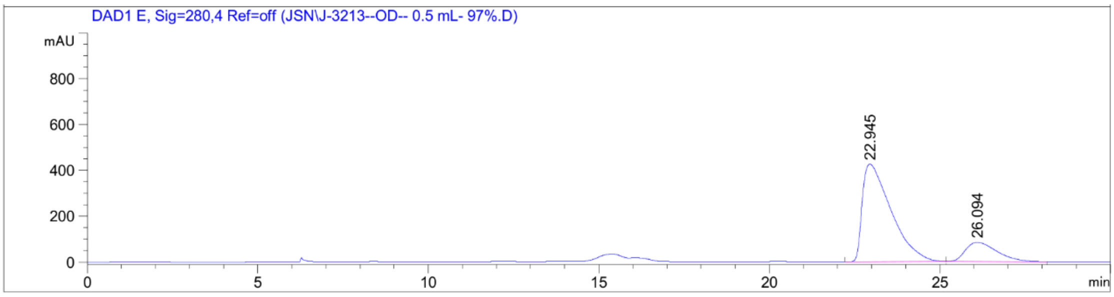

Peak #	RetTime [min]	Type	Width [min]	Area [mAU*s]	Height [mAU]	Area %
1	22.945	BB	0.8712	2.49334e4	425.77872	89.6176
2	26.094	BBA	0.7264	2888.58594	62.59844	10.3824

Supplementary Figure 331. HPLC of compound (R)-76

(R)-5-(1H-naphtho[1,8-de][1,3,2]diazaborinin-2(3H)-yl)-5-(4,4,5,5-tetramethyl-1,3,2-dioxaborolan-2-yl)pentyl (2E,4E)-hexa-2,4-dienoate (77)

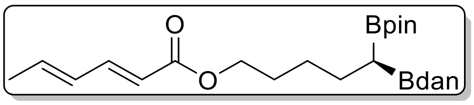

77

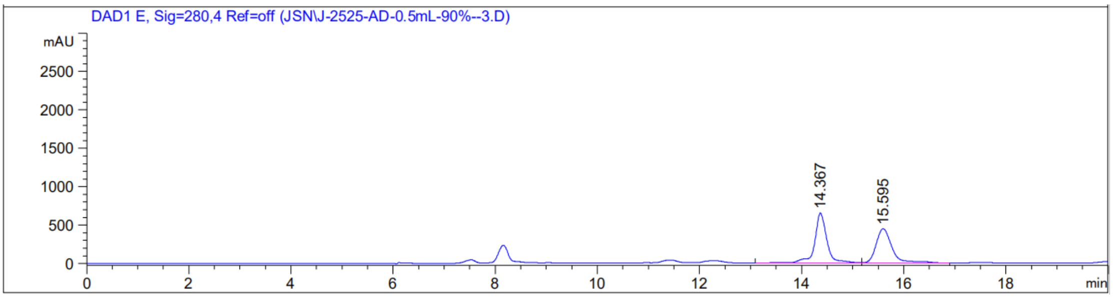

Peak #	RetTime [min]	Type	Width [min]	Area [mAU*s]	Height [mAU]	Area %
1	14.367	BV	0.2502	1.08570e4	652.27118	52.9041
2	15.595	VB	0.3335	9664.98828	444.89221	47.0959

Supplementary Figure 332. HPLC of compound rac-77

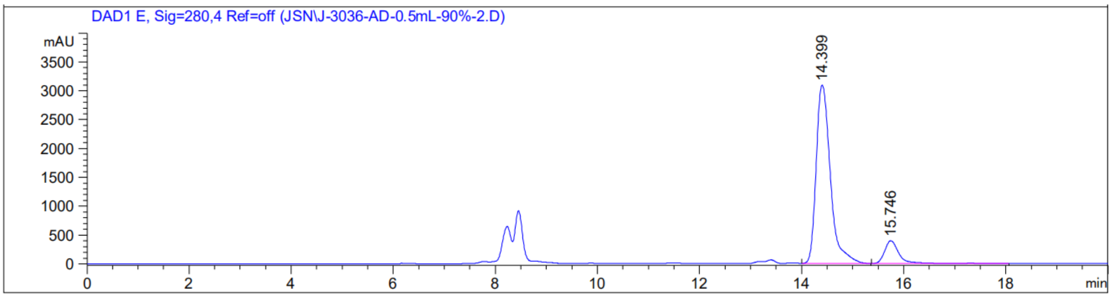

Peak #	RetTime [min]	Type	Width [min]	Area [mAU*s]	Height [mAU]	Area %
1	14.399	VB	0.2968	5.95971e4	3093.34424	88.4176
2	15.746	BV R	0.2928	7806.97803	393.98853	11.5824

Supplementary Figure 333. HPLC of compound (R)-77

(R)-5-(1H-naphtho[1,8-de][1,3,2]diazaborinin-2(3H)-yl)-5-(4,4,5,5-tetramethyl-1,3,2-dioxaborolan-2-yl)pentyl 2-propylpentanoate (78)

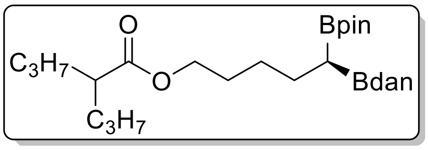

78

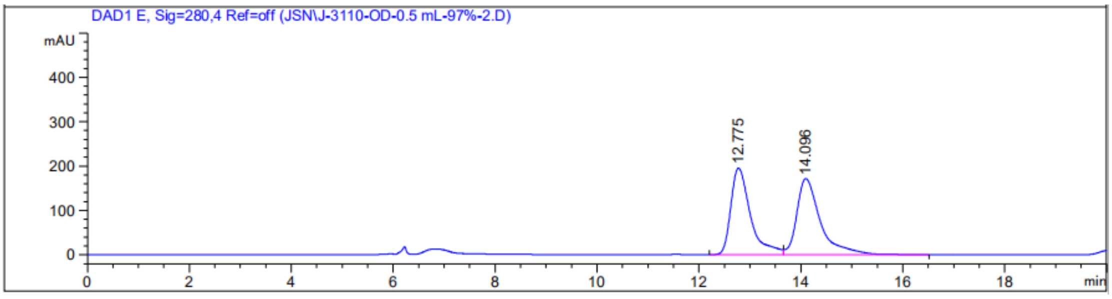

Peak #	RetTime [min]	Type	Width [min]	Area [mAU*s]	Height [mAU]	Area %
1	12.775	BV	0.4045	5219.79346	194.85825	48.6710
2	14.096	VB	0.4808	5504.84521	171.35628	51.3290

Supplementary Figure 334. HPLC of compound rac-78

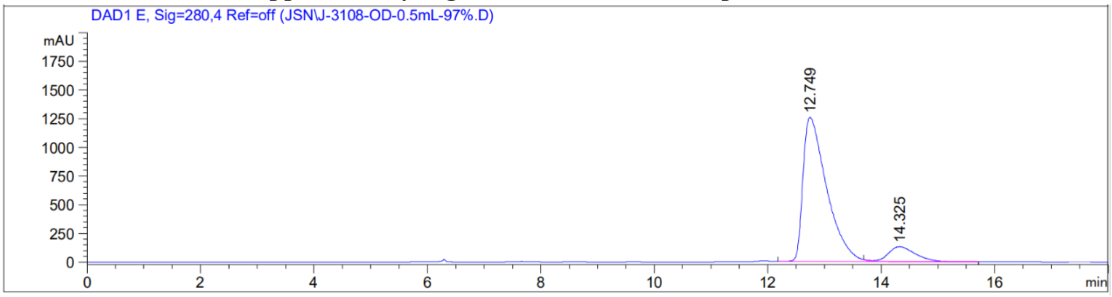

Peak #	RetTime [min]	Type	Width [min]	Area [mAU*s]	Height [mAU]	Area %
1	12.749	BV R	0.4360	3.63390e4	1254.20691	89.4230
2	14.325	VB E	0.5080	4298.18896	129.89571	10.5770

Supplementary Figure 335. HPLC of compound (R)-78

(R)-5-(1H-naphtho[1,8-de][1,3,2]diazaborinin-2(3H)-yl)-5-(4,4,5,5-tetramethyl-1,3,2-dioxaborolan-2-yl)pentyl benzoate (79)

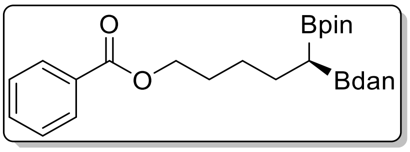

79

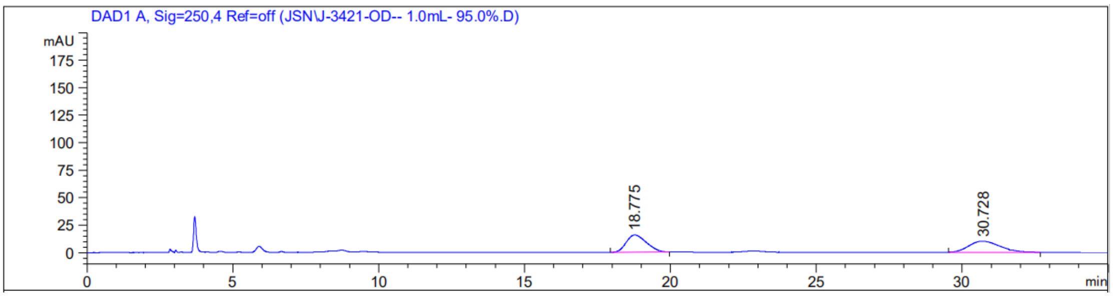

Peak #	RetTime [min]	Type	Width [min]	Area [mAU*s]	Height [mAU]	Area %

1	18.775	BB	0.6103	760.43848	15.66695	50.6188
2	30.728	BB	0.8727	741.84705	10.05539	49.3812

Supplementary Figure 336. HPLC of compound rac-79

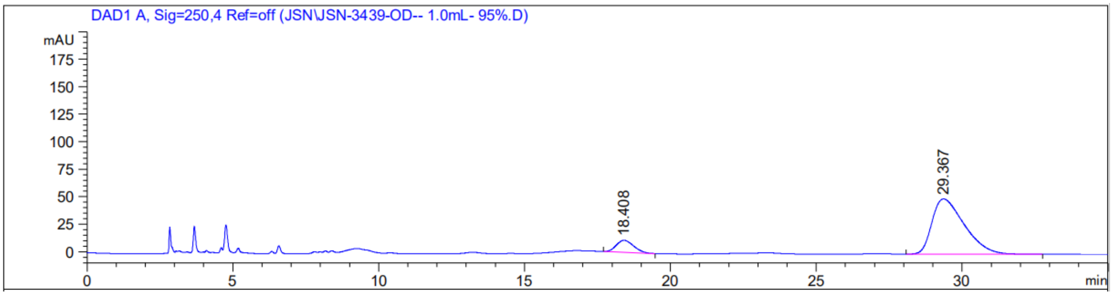

Peak #	RetTime [min]	Type	Width [min]	Area [mAU*s]	Height [mAU]	Area %
1	18.408	BB	0.6327	451.63766	10.91542	10.4569
2	29.367	BB	1.1192	3867.39209	50.17798	89.5431

Supplementary Figure 337. HPLC of compound (R)-79

(R)-5-(1H-naphtho[1,8-de][1,3,2]diazaborinin-2(3H)-yl)-5-(4,4,5,5-tetramethyl-1,3,2-dioxaborolan-2-yl)pentyl 4-methylbenzoate (80)

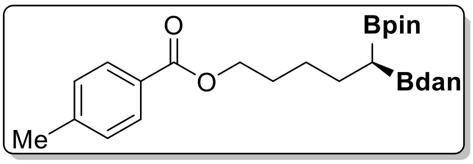

80

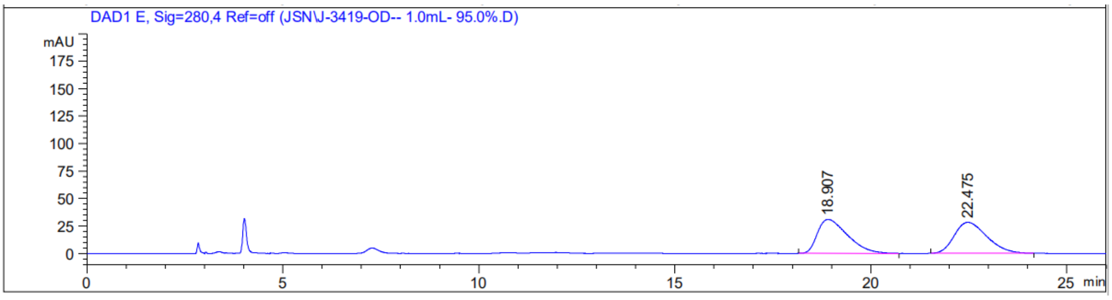

Peak #	RetTime [min]	Type	Width [min]	Area [mAU*s]	Height [mAU]	Area %
1	18.907	BB	0.7182	1655.19128	30.93835	50.2387
2	22.475	BB	0.6986	1639.46509	28.26812	49.7613

Supplementary Figure 338. HPLC of compound rac-80

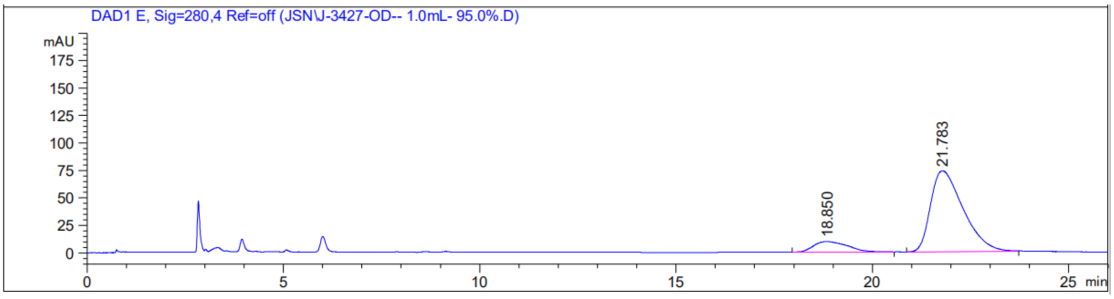

Peak #	RetTime [min]	Type	Width [min]	Area [mAU*s]	Height [mAU]	Area %
1	18.850	BB	0.7130	574.25592	9.55793	11.9572
2	21.783	BB	0.8210	4228.35547	73.51833	88.0428

Supplementary Figure 339. HPLC of compound (R)-80

(R)-5-(1H-naphtho[1,8-de][1,3,2]diazaborinin-2(3H)-yl)-5-(4,4,5,5-tetramethyl-1,3,2-dioxaborolan-2-yl)pentyl 4-cyclohexylbenzoate (81)

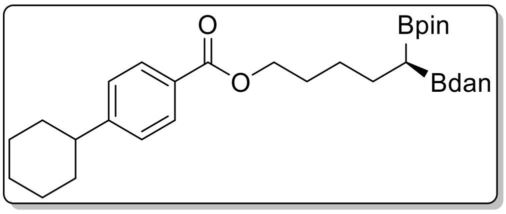

81

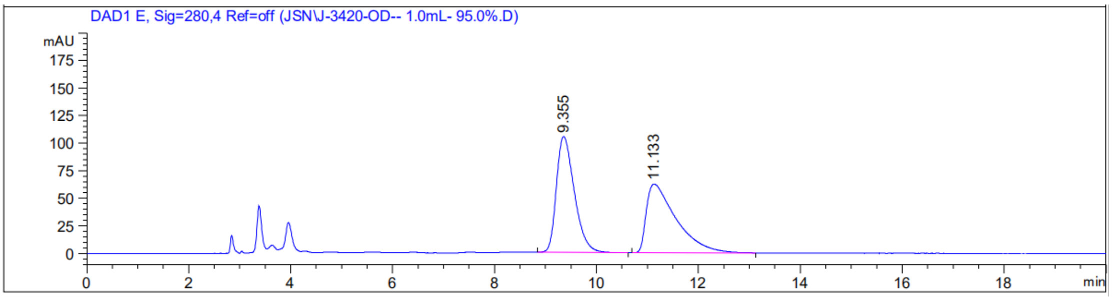

Peak #	RetTime [min]	Type	Width [min]	Area [mAU*s]	Height [mAU]	Area %
1	9.355	BB	0.3707	2561.59302	104.98648	50.3330
2	11.133	BB	0.5717	2527.70117	62.24888	49.6670

Supplementary Figure 340. HPLC of compound rac-81

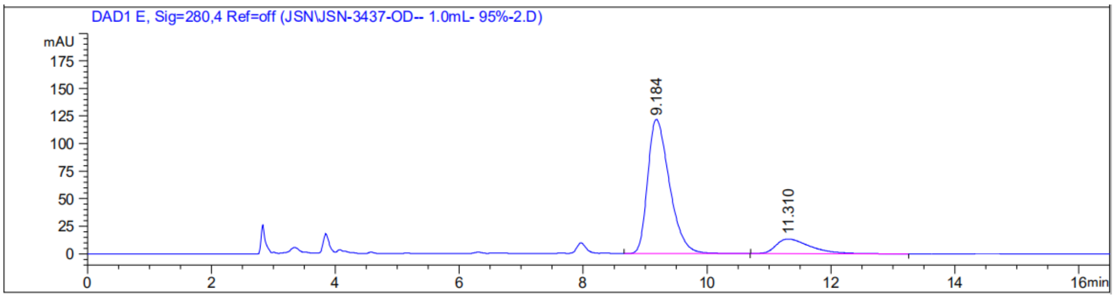

Peak #	RetTime [min]	Type	Width [min]	Area [mAU*s]	Height [mAU]	Area %

1	9.184	BB	0.3745	2967.78223	121.71830	85.3946
2	11.310	BB	0.5770	507.59317	13.30732	14.6054

Supplementary Figure 341. HPLC of compound (R)-81

(R)-5-(1H-naphtho[1,8-de][1,3,2]diazaborinin-2(3H)-yl)-5-(4,4,5,5-tetramethyl-1,3,2-dioxaborolan-2-yl)pentyl (E)-2-methyl-3-phenylacrylate (82)

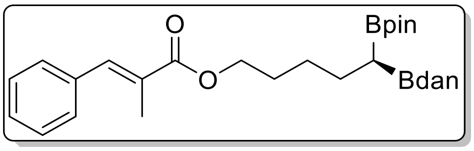

82

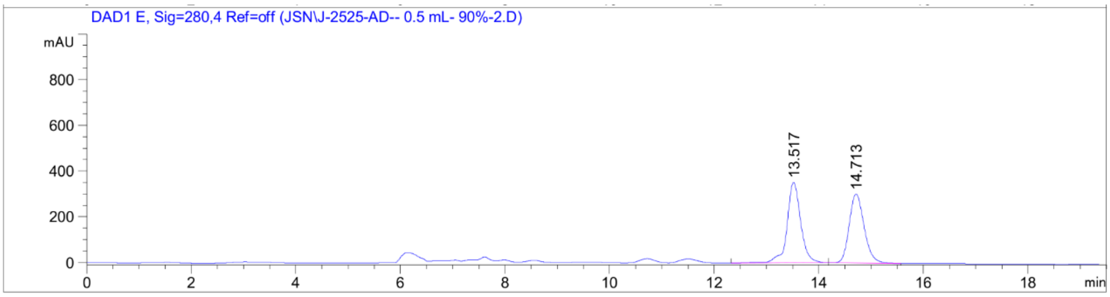

Peak #	RetTime [min]	Type	Width [min]	Area [mAU*s]	Height [mAU]	Area %
1	13.517	BB	0.2736	6394.96436	352.56653	51.6419
2	14.713	BB	0.3076	5988.32373	301.63293	48.3581

Supplementary Figure 342. HPLC of compound rac-82

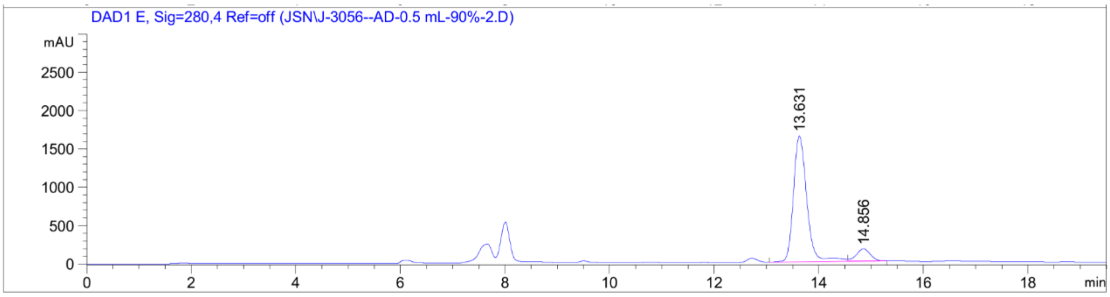

Peak #	RetTime [min]	Type	Width [min]	Area [mAU*s]	Height [mAU]	Area %
1	13.631	BV R	0.2658	2.92452e4	1641.33691	90.7317
2	14.856	VB E	0.2862	2987.40381	161.22876	9.2683

Supplementary Figure 343. HPLC of compound (R)-82

(R)-2-(2-phenyl-1-(4,4,5,5-tetramethyl-1,3,2-dioxaborolan-2-yl)ethyl)-2,3-dihydro-1H-naphtho[1,8-de][1,3,2]diazaborinine (87[a])

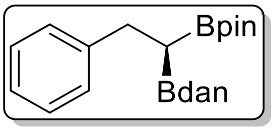

87[a]

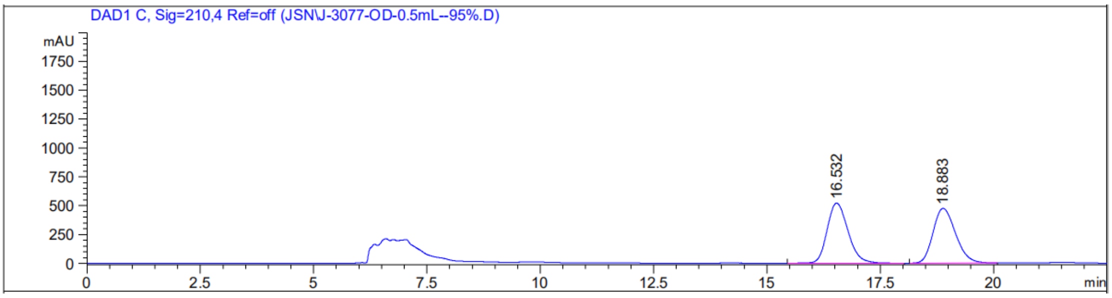

Peak #	RetTime [min]	Type	Width [min]	Area [mAU*s]	Height [mAU]	Area %
1	16.532	BB	0.4985	1.67143e4	520.72620	50.5286
2	18.883	BB	0.5303	1.63646e4	474.55447	49.4714

Supplementary Figure 344. HPLC of compound rac-87[a]

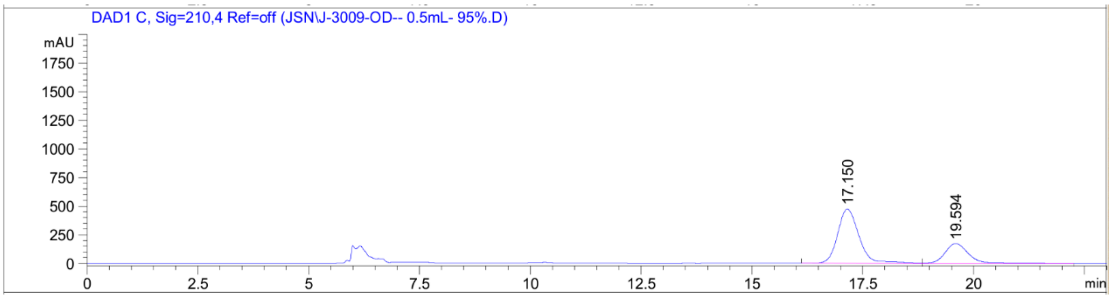

Peak #	RetTime [min]	Type	Width [min]	Area [mAU*s]	Height [mAU]	Area %
1	17.150	BV	0.5168	1.59323e4	473.17288	71.0814
2	19.594	VB	0.5692	6481.83154	173.76414	28.9186

Supplementary Figure 345. HPLC of compound (R)- 87[a]

(S)-2-(2-phenyl-1-(4,4,5,5-tetramethyl-1,3,2-dioxaborolan-2-yl)ethyl)-2,3-dihydro-1H-naphtho[1,8-de][1,3,2]diazaborinine (87[b])

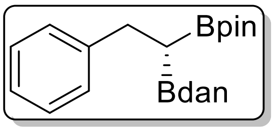

87[b]

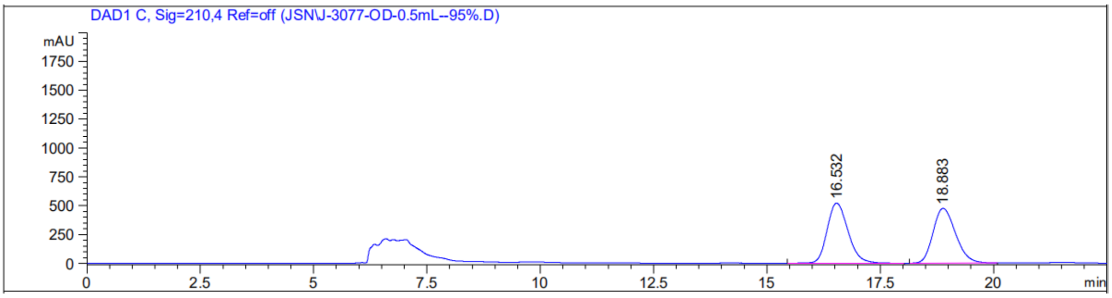

Peak #	RetTime [min]	Type	Width [min]	Area [mAU*s]	Height [mAU]	Area %
1	16.532	BB	0.4985	1.67143e4	520.72620	50.5286
2	18.883	BB	0.5303	1.63646e4	474.55447	49.4714

Supplementary Figure 346. HPLC of compound rac-87[b]

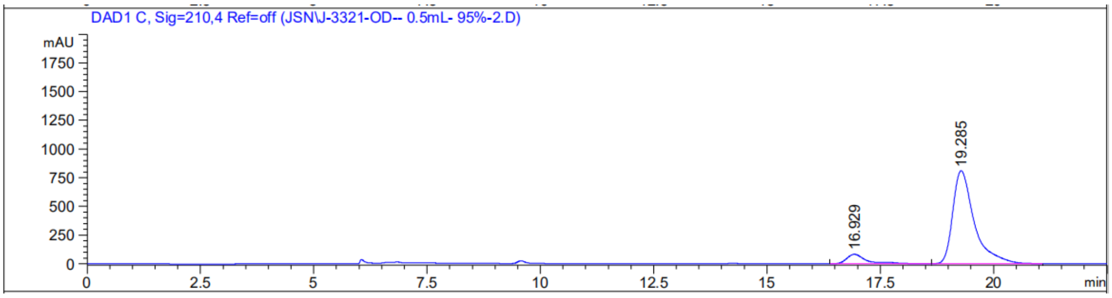

Peak #	RetTime [min]	Type	Width [min]	Area [mAU*s]	Height [mAU]	Area %
1	16.929	BV R	0.4081	2588.28394	83.44131	8.8532
2	19.285	BB	0.4917	2.66472e4	810.23145	91.1468

Supplementary Figure 347. HPLC of compound (S)- 87[b]

(S)-2-(1-(4,4,5,5-tetramethyl-1,3,2-dioxaborolan-2-yl)-2-(p-tolyl)ethyl)-2,3-dihydro-1H-naphtho[1,8-de][1,3,2]diazaborinine (88)

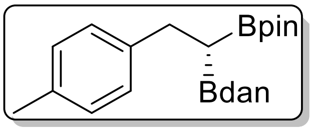

88

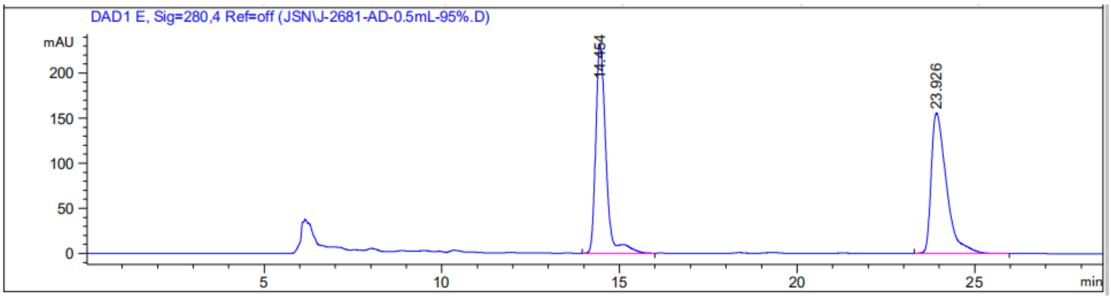

Peak #	RetTime [min]	Type	Width [min]	Area [mAU*s]	Height [mAU]	Area %

1	14.454	BV R	0.2911	4615.70898	231.86812	49.9542
2	23.926	BB	0.4531	4624.17871	155.42926	50.0458

Supplementary Figure 348. HPLC of compound rac-88

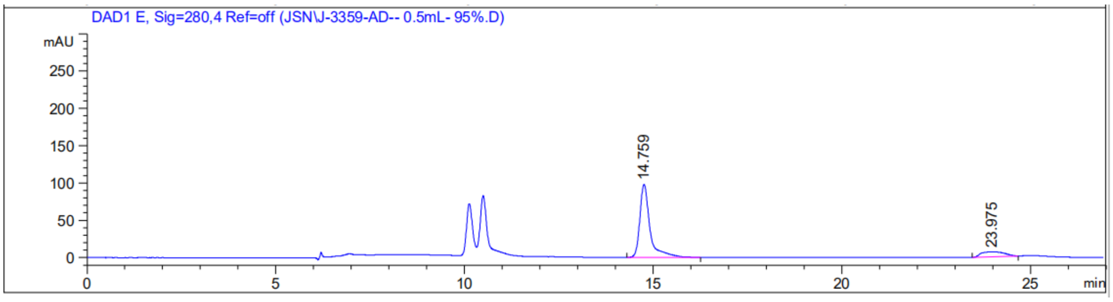

Peak #	RetTime [min]	Type	Width [min]	Area [mAU*s]	Height [mAU]	Area %
1	14.759	BB	0.2792	1841.42896	97.99349	86.4962
2	23.975	BB	0.5068	287.48306	6.77800	13.5038

Supplementary Figure 349. HPLC of compound (S)- 88

(S)-2-(1-(4,4,5,5-tetramethyl-1,3,2-dioxaborolan-2-yl)-2-(m-tolyl)ethyl)-2,3-dihydro-1H-naphtho[1,8-de][1,3,2]diazaborinine (99)

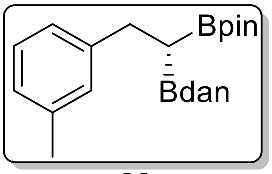

89

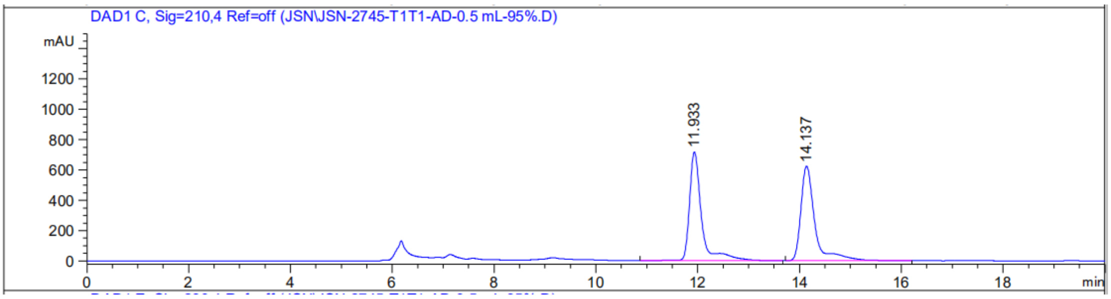

Peak #	RetTime [min]	Type	Width [min]	Area [mAU*s]	Height [mAU]	Area %
1	11.933	BV R	0.2246	1.19339e4	716.41162	49.9321
2	14.137	BV R	0.2673	1.19664e4	623.34894	50.0679

Supplementary Figure 350. HPLC of compound rac-89

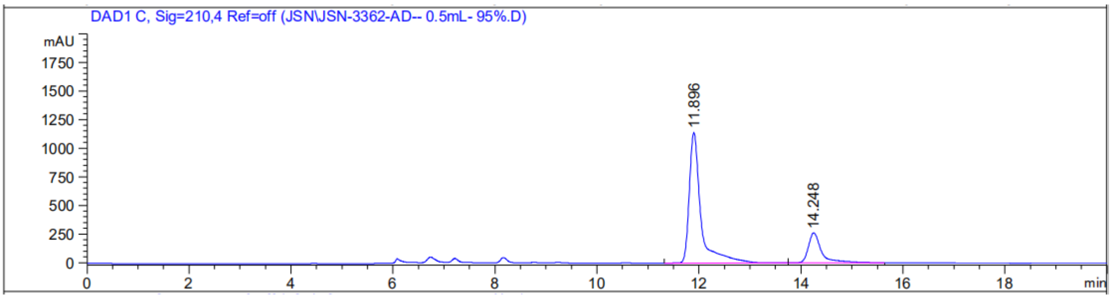

Peak #	RetTime [min]	Type	Width [min]	Area [mAU*s]	Height [mAU]	Area %
1	11.896	BB	0.2335	1.81295e4	1140.01892	79.4929
2	14.248	BB	0.2643	4676.94727	261.96622	20.5071

Supplementary Figure 351. HPLC of compound (S)- 89

(S)-2-(2-(4-ethylphenyl)-1-(4,4,5,5-tetramethyl-1,3,2-dioxaborolan-2-yl)ethyl)-2,3-dihydro-1H-naphtho[1,8-de][1,3,2]diazaborinine (90)

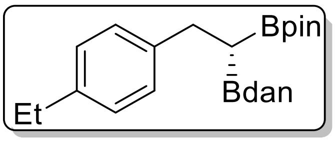

90

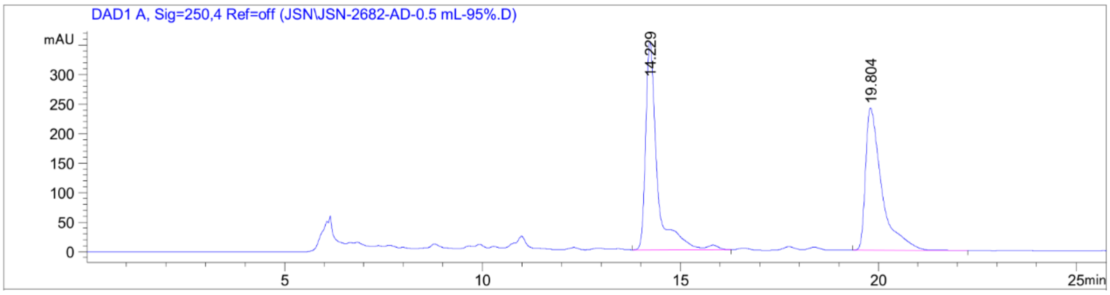

Peak #	RetTime [min]	Type	Width [min]	Area [mAU*s]	Height [mAU]	Area %
1	14.229	BV R	0.2951	7262.18848	352.94122	51.3728
2	19.804	BB	0.4171	6874.05029	240.75574	48.6272

Supplementary Figure 352. HPLC of compound rac-90

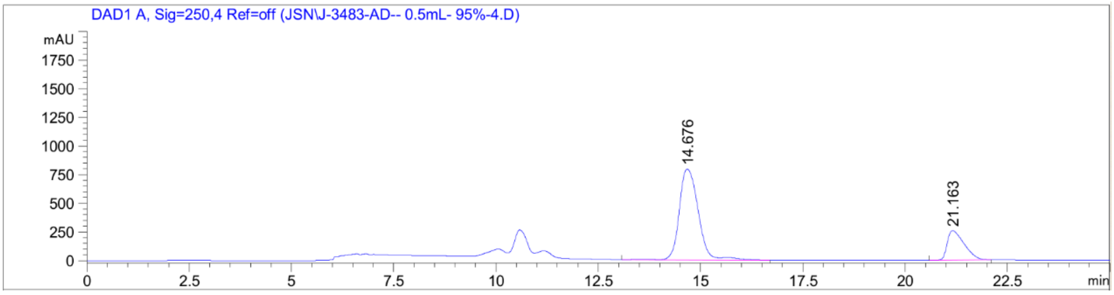

Peak #	RetTime [min]	Type	Width [min]	Area [mAU*s]	Height [mAU]	Area %

1	14.676	VV R	0.4974	2.56564e4	790.87378	77.7908
2	21.163	BB	0.4387	7324.85742	256.82422	22.2092

Supplementary Figure 353. HPLC of compound (S)- 90

(R)-2-(2-(4-propylphenyl)-1-(4,4,5,5-tetramethyl-1,3,2-dioxaborolan-2-yl)ethyl)-2,3-dihydro-1H-naphtho[1,8-de][1,3,2]diazaborinine (91)

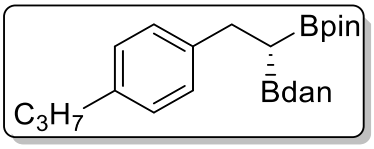

91

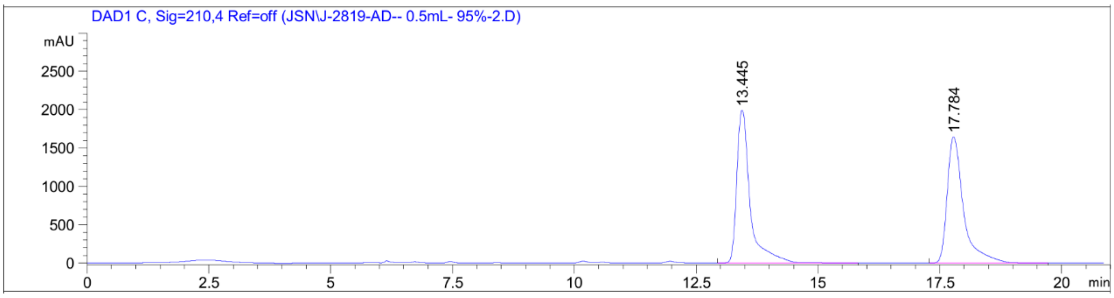

Peak #	RetTime [min]	Type	Width [min]	Area [mAU*s]	Height [mAU]	Area %
1	13.445	BB	0.2871	3.80495e4	1990.14209	49.7613
2	17.784	BB	0.3443	3.84146e4	1645.87903	50.2387

Supplementary Figure 354. HPLC of compound rac-91

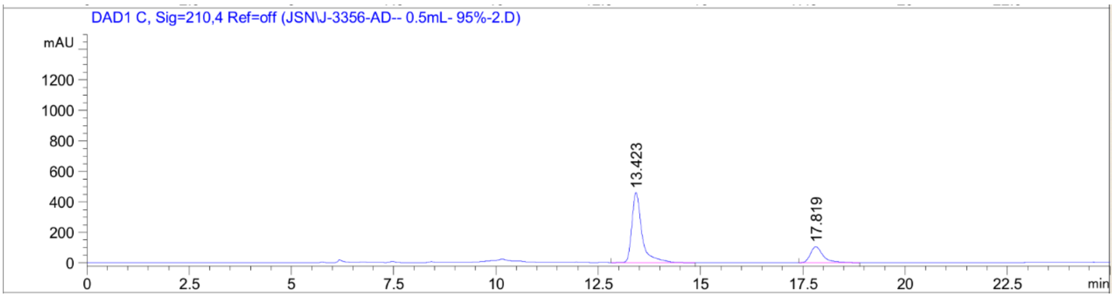

Peak #	RetTime [min]	Type	Width [min]	Area [mAU*s]	Height [mAU]	Area %
1	13.423	BB	0.2572	7999.01611	459.16885	77.2766
2	17.819	BB	0.3268	2352.13037	106.02388	22.7234

Supplementary Figure 355. HPLC of compound (S)- 91

5. Supplementary References

1. Kuang, Z., Mai, S., Yang, K. & Song, Q. Synthesis of anti-vicinal diboronates from diarylethynes and B_2pin_2. *Science Bulletin*. **64**, 1685-1690 (2019).

2. Feng, X., Jeon, H. & Yun, J. Regio- and Enantioselective Copper(I)-Catalyzed Hydroboration of Borylalkenes: Asymmetric Synthesis of 1,1-Diborylalkanes. *Angew. Chem. Int. Ed*. **52**, 3989–3992 (2013).

3. Gaussian 16, Revision A.03, Frisch, M. J.; Trucks, G. W.; Schlegel, H. B.; Scuseria, G. E.; Robb, M. A.; Cheeseman, J. R.; Scalmani, G.; Barone, V.; Petersson, G. A.; Nakatsuji, H.; Li, X.; Caricato, M.; A. Marenich,V.; Bloino, J.; Janesko, B. G.; Gomperts, R.; Mennucci, B.; Hratchian, H. P.; Ortiz, J. V.; Izmaylov, A. F.; Sonnenberg, J. L.; Williams-Young, D.; Ding, F.; Lipparini, F.; Egidi, F.; Goings, J.; Peng, B.; Petrone, A.; Henderson, T.; Ranasinghe, D.; Zakrzewski, V. G.; Gao, J.; Rega, N.; Zheng, G.; Liang, W.; Hada, M.; Ehara, M.; Toyota, K.; Fukuda, R.; Hasegawa, J.; Ishida, M.; Nakajima, T.; Honda, Y.; Kitao, O.; Nakai, H.; Vreven, T.; Throssell, K.; Montgomery, J. A.; Peralta, J. E.; Ogliaro, F.; Bearpark, M. J.; Heyd, J. J.; Brothers, E. N.; K. Kudin, N.; Staroverov, V. N.; Keith, T. A.; Kobayashi, R.; Normand, J.; Raghavachari, K.; Rendell, A. P.; Burant, J. C.; Iyengar, S. S.; Tomasi, J.; Cossi, M.; Millam, J. M.; Klene, M.; Adamo, C.; Cammi, R.; Ochterski, J. W.; Martin, R. L.; Morokuma, K.; Farkas, O.; Foresman, J. B.; Fox, D. J. Gaussian, Inc., Wallingford CT, 2016.

4. Lee, C., Yang, W. & Parr, R. G. Development of the Colle-Salvetti correlation-energy formula into a functional of the electron density. *Phys. Rev. B.* **37**, 785-789 (1988).

5. Becke, A. D. Density-functional exchange-energy approximation with correct asymptotic behavior. *Phys. Rev. A.* **38**, 3098 (1988).

6. Miehlich, B., Savin, A., Stoll, H. & Preuss, H. Results obtained with the correlation energy density functionals of Becke and Lee, Yang and Parr. *Chem. Phys. Lett.* **157**, 200-206 (1989).

7. Becke, A. D. Density-functional thermochemistry. III. The role of exact exchange. *J. Chem. Phys.* **98**, 5648-5652 (1993).

8. Grimme, S., Antony, S., Ehrlich, S. & Krieg, S. A consistent and accurate ab initio parametrization of density functional dispersion correction (dft-d) for the 94 elements H-Pu. *J. Chem. Phys.* **132**, 154104 (2010).

9. Roy, L. E., Hay, P. J. & Martin, R. L. Revised basis sets for the LANL effective core potentials. *J. Chem. Theory Comput.* **4**, 1029-1031 (2008).

10. Zhao, Y. & Truhlar, D. G. A new local density functional for main-group thermochemistry, transition metal bonding, thermochemical kinetics, and noncovalent interactions. *J. Chem. Phys.* **125**, 194101 (2006).

11. Marenich, A. V., Cramer, C. J. & Truhlar, D. G. Universal solvation model based on solute electron density and on a continuum model of the solvent defined by the bulk dielectric constant and atomic surface tensions. *J. Phys. Chem. B.* **113**, 6378-6396 (2009).

12. Weaver, J. H. & Frederikse, H. P. R. Crc handbook of chemistry and physics. *CRC Press. Boca Raton.* **76**, 12-156 (1977).

13. Chai, J.-D. & Head-Gordon, M. Long-range corrected hybrid density functionals with damped atom-atom dispersion corrections. *Phys. Chem. Chem. Phys.* **10**, 6615-6620 (2008).

14. Li, H. & Hall, M. B. Role of the chemically con-innocent ligand in the catalytic formation of hydrogen and carbon dioxide from methanol and water with the metal as the spectator. *J. Am. Chem. Soc.* **137**, 12330-12342 (2015).

15. Jiang, Y.-Y., Yan, L., Yu, H.-Z., Zhang, Q. & Fu, Y. Mechanism of vanadium-catalyzed selective C-O and C-C cleavage of lignin model compound. *ACS Catal.* **6**, 4399-4410 (2016).

16. Yu, J.-L., Zhang, S.-Q. & Hong, X. Mechanisms and origins of chemo- and regioselectivities of Ru(II)-catalyzed decarboxylative C-H alkenylation of aryl

carboxylic acids with alkynes: A computational study. *J. Am. Chem. Soc.* **139**, 7224-7243 (2017).

17. Legault, C. Y. CYLview, 1.0b, Universite de Sherbrooke: Quebec, Canada, 2009; http://www.cylview.org.
